# Functional Prediction of Hypothetical Transcription Factors of *Escherichia coli* K-12 Based on Expression Data

**DOI:** 10.1016/j.csbj.2018.03.003

**Published:** 2018-03-27

**Authors:** Emanuel Flores-Bautista, Carenne Ludeña Cronick, Anny Rodriguez Fersaca, Mario Alberto Martinez-Nuñez, Ernesto Perez-Rueda

**Affiliations:** a Facultad de Ingenieria Química, Universidad Autónoma de Yucatán, Mexico; b Departamento de Ciencias Básicas, Universidad Jorge Tadeo Lozano, Bogotá, Colombia; c Facultad de Ciencias, Universidad Antonio Nariño, Bogotá, Colombia; d Laboratorio de Ecogenómica, Unidad Académica de Ciencias y Tecnología de Yucatán, Facultad de Ciencias, UNAM, Mérida, Yucatán, Mexico; e Instituto de Investigaciones en Matemáticas Aplicadas y en Sistemas, Universidad Nacional Autónoma de México, Unidad Académica Yucatán, C.P. 97302 Mérida, Yucatán, Mexico; f Departamento de Ingenieria Celular y Biocatálisis, Instituto de Biotecnología, UNAM, Cuernavaca C.P. 62210, Morelos, Mexico

**Keywords:** Transcription factor, *Escherichia coli*, Gene expression, Hypothetical proteins, Spectral clustering

## Abstract

The repertoire of 304 DNA-binding transcription factors (TFs) in *Escherichia coli* K-12 has been described recently, with 196 TFs experimentally characterized and 108 proteins predicted by sequence comparisons. Based on 303 expression profile patterns retrieved from the Colombos database 12 clusters were identified, including hypothetical and experimentally characterized TFs, using a spectral clustering algorithm based on a 3NN graph built using 14 principal components that represent 65% of the variance of the expression data. In a posterior step, clusters were characterized in terms of their associated overrepresented functions, based on KEGG, Supfam annotations and Pfam assignments among other functional categories using an enrichment test, reinforcing the notion that the identified clusters are functionally similar among them. Based on these data, the we identified 12 clusters in which hypothetical and known TFs share similar regulatory and physiological functions, such as module associations of toxin-antitoxin (TA) systems with DNA repair mechanisms, amino acid biosynthesis, and carbon metabolism/transport, among others. This analysis has increased our knowledge about gene regulation in *E. coli* K-12 and can be further expanded to other organisms.

## 1 Introduction

In recent years, the amount of information associated with biological data has increased exponentially, and it has also increased the number of protein and DNA sequences with no evident functions. In this regard, although experimental determinations of protein functions are the most reliable way to characterize proteins of unknown activity, it is a challenge to conduct experiments for the large number of proteins predicted so far. A common strategy to determine functions and guide experimentalists is to compare sequences and structures between experimentally determined proteins and hypothetical ones. However, the gap between proteins with an experimentally determined function and those with still-unknown function is rapidly increasing [1]. A recent study suggested that >40% of known proteins lack any annotation in public databases, although many are evolutionarily conserved and probably play important biological roles [2].

*Escherichia coli* K-12 strain MG1655 represents one of the most important model organisms in biology. Its chromosome is composed of a 4.6-MB circular, negatively supercoiled DNA molecule that contains 4679 genes. Although *E. coli* K-12 MG1655 represents an archetype for molecular biology because of the large amount of experimental information that researchers have accumulated for this organism, only two-thirds of its protein-encoding genes are associated with an assigned function in the HAMAP database [3], demonstrating the necessity of finding approaches to identify probable functions associated with the protein repertoire.

An important element associated with gene expression in this bacterium corresponds to DNA-binding transcription factors (TFs), which provide the ability to contend with environmental changes by blocking (via negative regulation) or allowing (via positive regulation) access of the RNA polymerase (RNAP) to promoters [[4], [5], [6]]. Previous analyses identified 304 TFs that could regulate gene expression in *E. coli* [7]; of these, 196 TFs have been experimentally characterized, whereas 108 correspond to predictions based on sequence comparisons [8]. In this work, in order to elucidate the diverse regulatory functions associated with hypothetical TFs, clustering analyses based on 303 expression profile pattern data were performed using a spectral clustering algorithm based on a 3NN graph. We describe a workflow to retrieve the enriched pathways and biological processes from the resulting clusters of coexpressed genes, based on the target genes deposited on the RegulonDB and Ecocyc databases. In our analysis, we identified 12 clusters in which hypothetical and known TFs share similar regulatory and physiological functions.

## 2 Material and Methods

### 2.1 Identification of DNA-binding TFs

A total of 196 TFs have been experimentally characterized, and this information has been deposited in RegulonDB [8] and Ecocyc [9]; these TFs were used as seeds in BLASTP searches against the complete proteome of *E. coli*. E-values of ≤1e−6 and a coverage of 70% were considered for further analysis. In addition, TFs were retrieved that were specifically associated with *E. coli* K-12 and for which information has been deposited in the DBD, HAMAP [3], Superfamily DB [10], or PFAM [11] databases. Finally, those TFs were scrutinized to assess their domain organization by using the Superfamily and PFAM database assignments [10], with an E-value at ≤10^−3^ to be considered as significant. In addition, superfamily domains were associated to functional categories.

### 2.2 Expression Data Pre-processing

All statistical analyses conducted in this study considered the expression of 291 out 304 genes over 303 expression profiles (See Supplementary material Table S1) obtained from the Colombos database [12]. 13 TFs were not included in the dataset because they did not contain robust information concerning expression pattern. In brief, the Colombos database is a compendium on expression by bacterial organisms, as it combines expression information from different microarray platforms and experiments. The compendium also incorporates annotations for both genes and experimental conditions. These heterogeneous data are integrated to allow interactive browsing and queries of the compendium, not only for specific genes or experiments but also for metabolic pathways, as well as transcriptional regulation mechanisms, and other related topics.

**Fig. 1:**
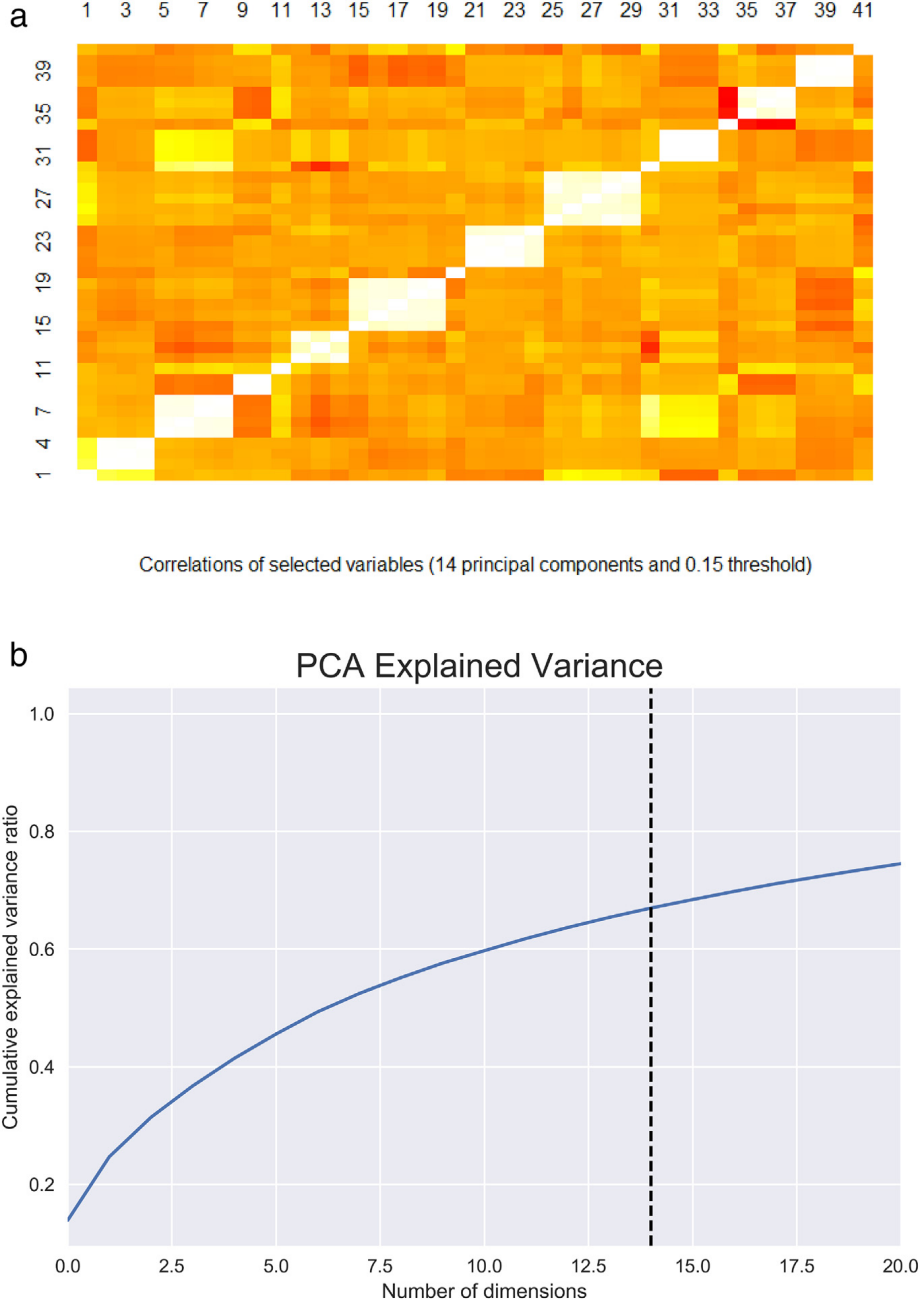
a) Cummulative proportion of explained variance as a function of the number of principal components. Dashed line corresponds to 16 components accounting for 68% of total variance. In X-axis is the number of dimensions and in Y-axis is the cumulative variance ratio. b) Heatmap of absolute value of correlations of projections of original attributes over the first 14 principal components, accounting for over 65% of the overall variance. Red is equivalent to low correlations and white to high correlations.

In order to select the most informative attributes, i.e. expression values that explain a high percentage of the overall variance, a principal component analysis (PCA) was performed, using the program *prcomp* from the package *stats* in the R statistical program. PCA is a linear dimensionality reduction technique that uses a linear combination of the variables to maximize the variance in a high-dimensional dataset. In our analysis, each principal component is a linear combination of all conditions of the expression data. Attributes were selected based on the value of projections over the first 14 components accounting for 65% of the overall variance. For this a two-step procedure was used. First, a threshold value was established by visual inspection of the correlation heatmap. A good threshold was assumed to provide a significant contrast among selected variables (based on correlations). Visually, this produces a block diagonal image with high contrasts with out-of-the-diagonal points. If the threshold is too small, contrasts decrease. If the threshold is too big few points are selected. Based on this procedure a threshold of 0.15 was selected (see Fig. 1). Second, to exclude highly correlated points only one point was selected per block. Namely, points were selected in such a way as to assure that correlations among them were lower than 0.9. With this procedure 16 attributes were selected: ×67, ×71, ×79, ×138, ×315, ×434, ×468, ×498, ×516, ×535, ×971, ×1499, ×1911, ×1976, ×1835, and ×2364. It is however important to stress the linear nature of PCA. Although a powerful method for dimension reduction, it does not necessarily provide a robust clustering procedure if clusters are not radial in nature. That is, defined by correlation-based similarities.

### 2.3 Cluster Identification

Spectral clustering relies on k-means clustering of the smallest eigenvectors of the Laplacian or normalized Laplacian of a “similarity” graph associated with an ensemble of points [13]. We define the Laplacian *L* value considering a weight matrix *W*, to be *L* = *D* − *A*, or *L*_*W*_ = *D* − *W*, where *D* is the degree matrix and *A* is the adjacency matrix of a graph. The normalized Laplacian is then defined according to whether the nonsymmetric or symmetric version is considered: *L*_*N*_ = *D*(*I* − *D*^−1^*W*) or *L*_*NS*_ = *D*^−1/2^*LD*^−1/2^. The smallest eigenvectors correspond to the smallest eigenvalues (excluding the first, which is zero) of the considered Laplacian. Effectiveness of the method is based on two key observations. First, typically, the associated graph is based on nonlinear methods such as nearest neighbors (NN) thus ensuring a nonlinear embedding of the original data set into an appropriate feature space. Second, eigenvectors associated to the (second) smallest eigenvalues choose the directions minimizing a functional, which can be interpreted as a continuous version of the mincut problem. That is, eliminating the smallest amount of edges in order to obtain a nonconnected graph, where connected components are then interpreted as clusters. Efficiency of the proposed method is increased by prior linear dimension reduction using a PCA. Next, spectral clustering was used and was based on the selected 16 attributes with *specClust* in the package *kknn*, using the nearest-neighbor standard (weak)-associated graph with the 3 nearest neighbors and the symmetric normalized Laplacian, *L*_*NS*_. However, results did not vary when we considered more complex nearest-neighbor structures (number of *NN* values considered ranged from 3 to 10). The number of clusters was selected by (local) minimization of the total within the sum of squares (WSS) for clustering 2 to 90 clusters. The first major descent of this metric occurred at k = 6 clusters. Enriching this clustering the second occurred for k = 12 clusters. In order to study stability of proposed solutions, 500 random trials of the spectral clustering were considered obtaining a coefficient of variation of 0,1%, indicating a strong stability of the solution. For the final clustering scheme, a ratio of between sums of squares to the total sum of squares of 70.1% was achieved. The obtained clusters are shown in Table 1, along with their individual WSS values and the list of TFs in each cluster. Clusters were relatively uniform in size and in spread, as measured by the WSS.

**Table 1:** Clusters of TFs identified by similar profile patterns of expression.

Cluster	Experimentally characterized		Hypothetical TFs	N	WSS
	Strong	Weak			
C1	AraC, CsgD, FeaR, GadX, GalS, LsrR, MelR, Mlc, RclR (YkgD)	AbgR, CdaR, FucR, MhpR, SrlR (GutR), HcaR, MtlR, PrpR, RhaR,TdcA, YiaJ, YeiL	YahB, YbiI, YneJ, YgeV, YgfI, YihL, ChpS, SfsA (MalQ), DmlR (YeaT), LgoR (yjjM), YebK (HexR)	32	11.81
C2	GalR, LrhA, NarP, RelE, RelB, PuuR, MqsA,LexA, McbR (YncC), SoxR, YefM	GlcC, Hha, MalI, MntR, NsrR, UidR,	YdjF, YeeY, YgjM (HigA), YgiT, YjgJ	22	5.43
C3	AlaS, ArgR, BaeR, CpxR, CysB, FruR, NhaR, NikR, NrdR, PepA, PurR, SdiA, TreR, TrpR, TyrR, UxuR, YehT, YjiE (HypT), YqhC,	AllR, AppY, BglJ, DeoR, EbgR, FabR, IdnR, LacI, UhpA,	YcaN, YdiA (PpsR), YfeR, YgbI, YggD (FumE), YhaJ, YidZ	35	10.88
C4	Ada, CadC, ChbR, GlpR, DpiA, IhfB, HipB, GlrR (YfhA), PhoB, RutR, SlyA, TtdR, YdeO,	Crl, CreB, DhaR, IlvY, PerR, SfsB	DicC (regulated by DicA), (regulated by RcsB-BglJ), YbcL, YbcM, YbeF, YbhD, YdaS, YddM, YnfL, YdhB, YfhH, YqeH, YhjB, YjhI, YjjQ, YjjJ	34	9.13
C5	CaiF, Cbl, CytR, FlhDC, Hns, HupA, HupB, IhfA, Lrp, NarL, GlnG, StpA,	CsiR, EutR, MalT,	OgrK, YpdC, YphH,	19	8.52
C6	CspA, MarA, UlaR, ComR (YcfQ),	MarR, NemR (YdhM)	CspH, CspG, YdfH, YbaO	10	2.13
C7	AidB, GadE, PutA,	BolA, LldR,	CspD, DctR, YhjC, YiaG, YjdC	10	1.59
C8	AgaR, DicA, ArgP, EvgA, ExuR, FadR, FNR, Fur, IscR, GadW, NagC, NanR, PdhR, Rob, RstA, UvrY, YcgE (BluR) MlrA	GcvA, GntR, KdgR,	YbaQ, YciT (DeoT), YfgA (RodZ)	25	3.14
C9	AdiY, ArcA, CueR, HdfR, LeuO, Nac, OmpR, OxyR, PhoP, PgrR (YcjZ), PspF, RcsA, RcsB, SoxS, YqjI YeaM (NimR),	AsnC, BetI, DsdC, LysR, PspC, PspF, YfaX(RhmR)	YafC, YbiH, YeiE, YieP, YtfH, YijO	28	7.15
C10	AtoC, Crp, CusR, DcuR, FhlA, Fis, KdpE, MprA, SgrR, RhaS, XylR	AllS, AlpA, ArsR, EnvR, EnvY, HycA, NadR, GutM, HyfR, PhnF, RbsR, RpiR (AlsR), RtcR, TdcR,	YbdO, CspE, YmfL, YdiP, YqeI, YgeH, YidL, YidP, FrvR, FimZ (YbcA), SgcR (YjhJ), SlmA (YicB), DgoR (YidW)	38	9.93
C11	AcrR, IclR, ModE, RcnR, TorR, YdcN, YedW, YegW, RcdA (YbjK), YahA (PdeL)	Cnu	YagI, YbfE, CspI, CspB, CspF	16	3.44
C12	BirA, BasR, CynR, MetR, MngR, MurR (YfeT), PaaX,	AscG, FrlR (yhfR), NorR, XapR, MazE	YafN, YcjW, YdcQ, YdcR, YeiI, YfiE, YiaU, YihW, YjhU, YjiR	22	3.4

Columns are as follow: Cluster number, known TFs (strong and weak evidences) and hypothetical TFs; number of TFs per cluster; and individual (within) sum of squares (WSS).

### 2.4 Functional Classes of the Regulated Genes

To evaluate the associations between the functional categories and their corresponding clusters, we used one-tailed Fisher's Exact Test (FET). FET is based on the hypergeometric probability and can be used to calculate the significance, or P-value of the overlap between two independent datasets. We set statistical significance at a P-value of <0.045. Together with FET, we also determine the False Discovery Rate (FDR) of the tests to account for Type I errors. Multiple-testing corrections were performed using the Benjamini and Hochberg step-up false-discovery rate (FDR)-controlling procedure to calculate adjusted P-values. All analyses were performed using the R software [14].

## 3 Results

### 3.1 Regulatory Mechanism Associated With Hypothetical TFs in the Bacterium *E. coli* K-12

TFs were defined as DNA-binding proteins needed to activate or repress the transcription of a gene, but TFs are themselves part of neither the RNAP core nor the holoenzyme [15]. Therefore, sigma factors were not considered TFs in this study. Based on the information deposited in RegulonDB and Ecocyc, our literature search, and our sequence analyses, 196 TFs were experimentally characterized and 108 predicted TFs were identified. The 196 experimentally characterized TFs regulate a total of 1807 genes out 4679; that corresponds to 38.6% of the total genes in *E. coli* K-12, and represents 4490 regulatory interactions according RegulonDB, reinforcing the notion that it is one the best known organisms described so far, in terms of gene regulation.

Based on information deposited in RegulonDB and Ecocyc, we determined that around 16% of the total number of hypothetical TFs is regulated by 25 different regulatory proteins, among them global regulators, such as Crp and HNS, and sigma factors, like RpoS and RpoD. In detail, four global regulators (Crp, Fis, Fnr and HNS) are regulating the expression of 9 hypothetical TFs (SfsA, YjjQ, CspD, CspE, CspI, DctR, LgoR, MalQ, and YjjQ), suggesting their integration on well-known regulons. In addition, 8 hypothetical TFs are regulated by one protein, 7 hypothetical TFs are regulated by two proteins, and 2 hypothetical TFs are regulated by three or more proteins, like CspD and DctR. In this regard, the most plausible explanation is that hypothetical regulators belong to regulons already described, reinforcing the notion of recruitment of new elements of previously identified regulatory networks, however further evidences are necessary.

### 3.2 Identification and Consistency of Functional Clusters Based on Similar Expression Patterns

Previous analyses describing the important role of coregulation in the regulatory network of *E. coli* K-12 have been reviewed elsewhere [16], and the analyses showed that the interplay of TFs in a regulatory region will determine expression. In this regard, it is reasonable to ask whether similar expression profiles also suggest common regulatory processes. If this hypothesis is true, hypothetical TFs could be associated with functional categories that would be posteriorly experimentally corroborated. Based on this assumption, PCA and spectral clustering algorithms were applied. 12 clusters with similar expression patterns were identified, yielding hypothetical and well-known TFs in each group or cluster. Moreover, the well-known TFs included regulated genes with similar physiological functions, suggesting that, in functional terms the obtained clusters are robust. In order to evaluate the consistency of our clusters, we compared the 11 modules using the EcoMAC expression dataset, and 12 modules using the COLOMBOS dataset (including non-coenriched TFs) recently identified by Fang et al. [17] against clusters identified in this work at the target gene level. From this, we identified a functional enrichment in 5 out 11 modules for at least one cluster from the EcoMAC dataset (Supplementary material Table S2). All these comparisons represent a significant biological coherence. Interestingly, in both methods the toxin-antitoxin (TA) systems are clustered (Cluster 2 and Module 6, P-value < 0.001, Fisher Exact Test) with oxidative stress response and DNA-repair TFs, that is consistent with the functional roles of the TA systems. Another interesting functional insight was that of the co-clustering of multistress response TFs (Cluster 9 and Module 1, P-value < 0.001), which represent a functional relationship that has been previously described [7,17]. We also performed an enrichment analysis on the COLOMBOS dataset modules and similar results were obtained. Overall, these results suggest a functional relationship between the expression patterns within the modules of the regulatory network of *E. coli* K-12, that can be verified by similar approaches. In what follows, we describe the most relevant clusters identified.

### 3.3 Clusters Included Genes With Common Regulatory Processes

#### 3.3.1 Carbohydrate Metabolism Cluster (Cluster 1)

**Fig. 2:**
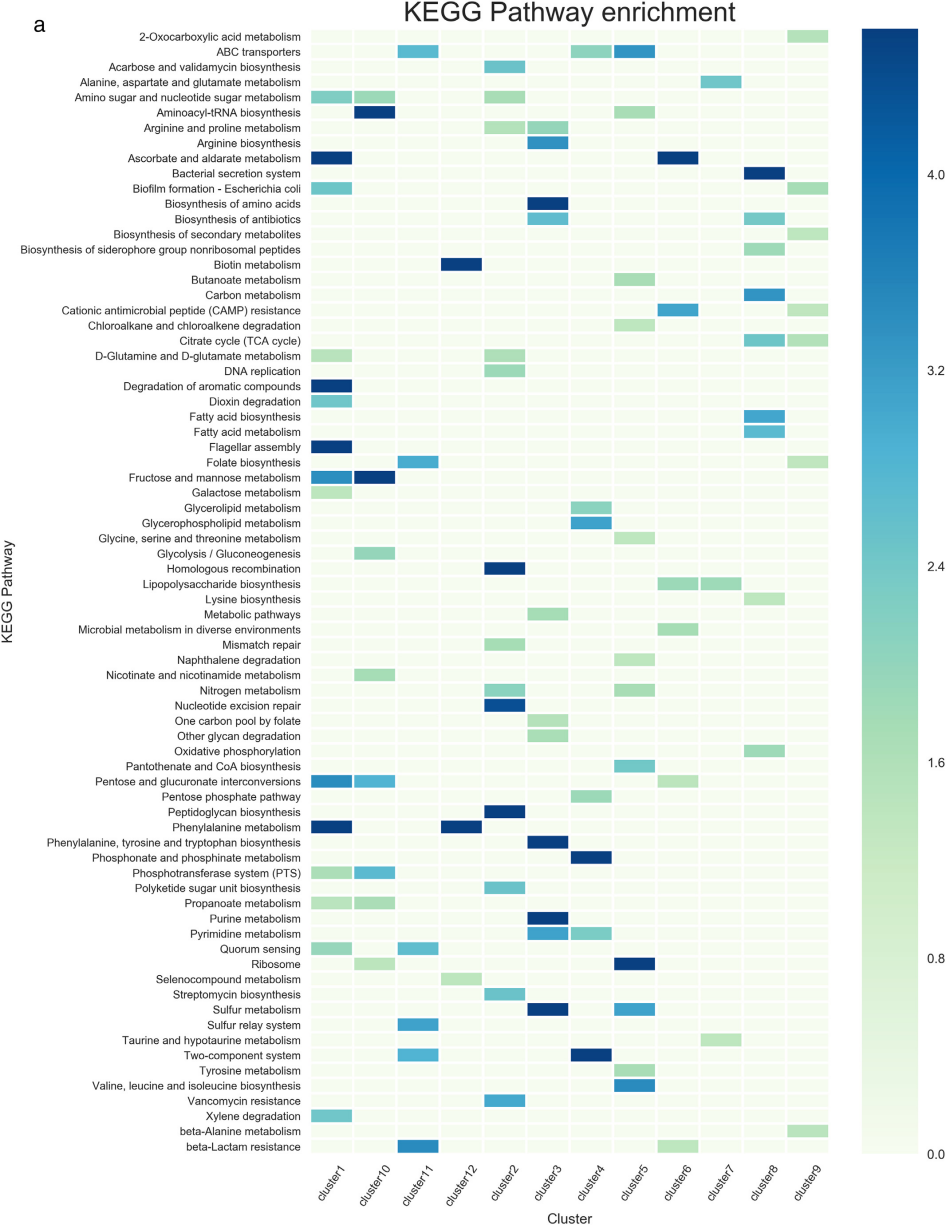

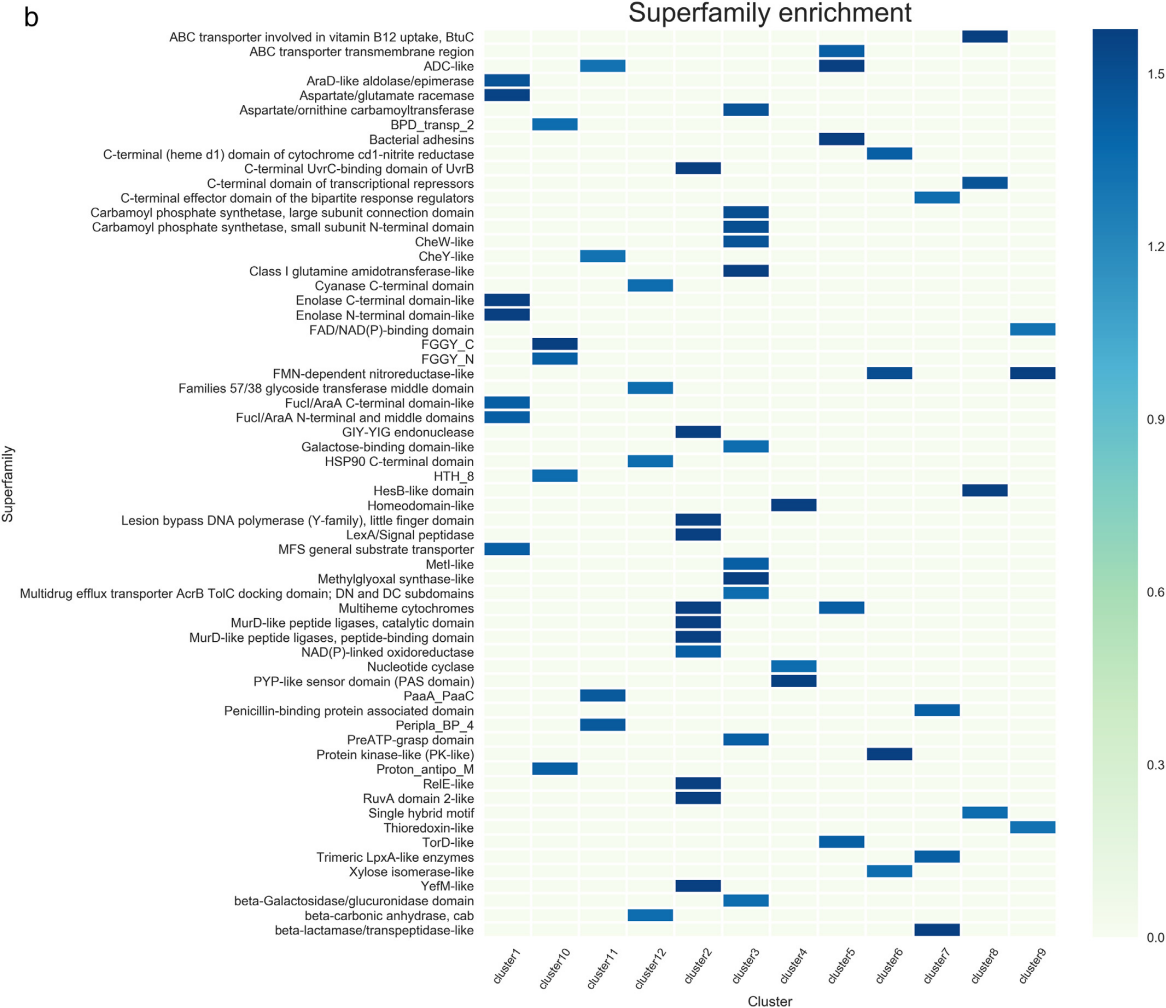

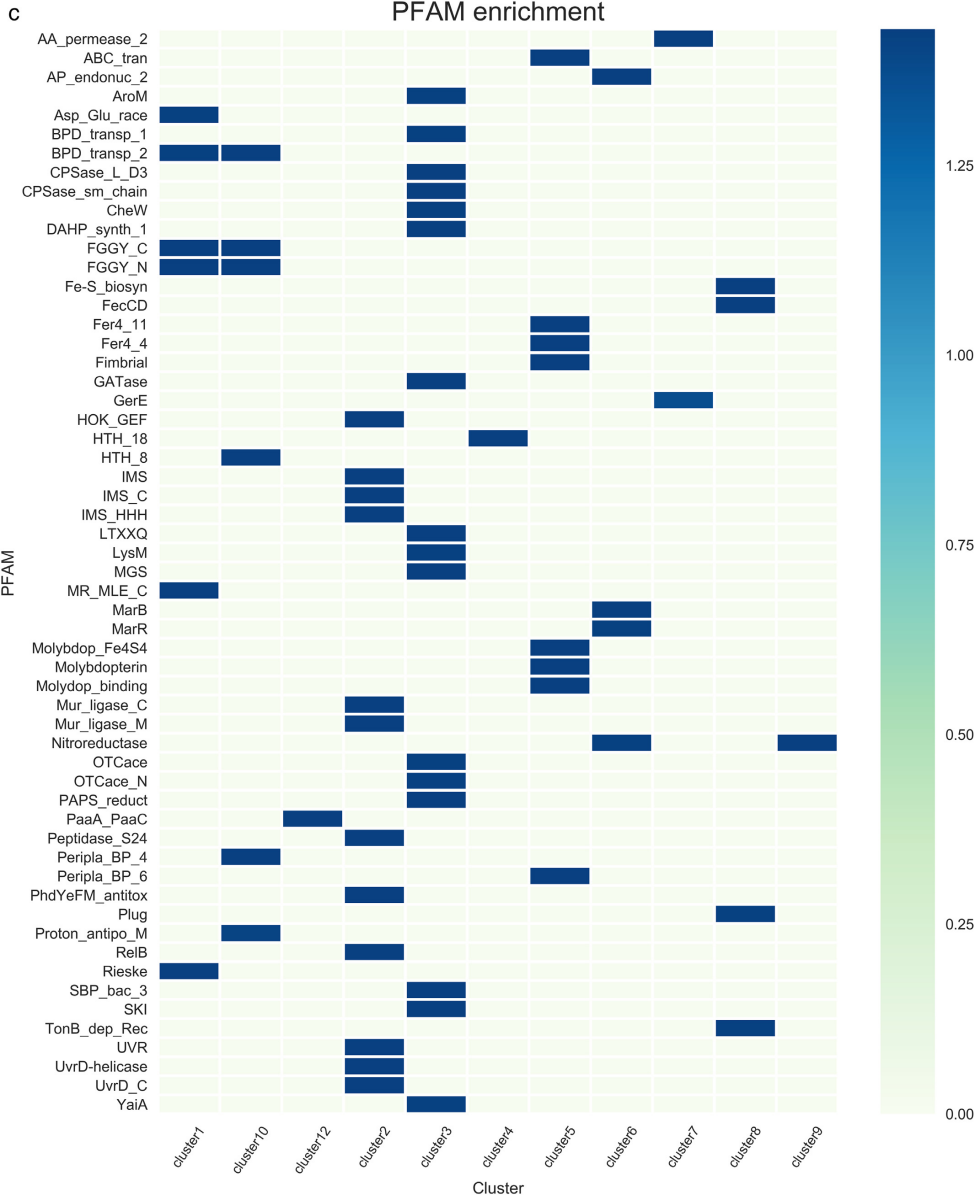
Functional assignments based on a) KEGG, b) SUPFAM and c) Pfam annotations. Functional assignments were evaluated per cluster. Only enriched functions were plotted as a heatmap. Colorbar represents −log P-value with Benjamini Hochberg correction.

In cluster 1, 21 TFs that have been experimentally characterized, such as AraC and MelR, and 11 hypothetical TFs identified by sequence comparisons were included. These 32 TFs were identified to have similar expression patterns according to the Colombos database and cluster analysis. In this cluster, members of the LysR and AraC/XylS families are overrepresented (P = 0.056 and 0.0078, respectively), showing evolutionary consistency in terms of the protein members associated with this group. In order to evaluate functional coherence, 321 genes regulated by these 21 TFs experimentally described were analyzed. Based on KEGG Pathway annotation and Supfam functional categories annotations we found carbohydrate metabolism (Fructose and mannose, Amino sugar and nucleotide sugar, Propanoate, and Galactose metabolism, among others); Cell motility (Flagellar assembly); and Cellular community (Biofilm formation and Quorum sensing) systems enriched with adjusted P-values below 0.045 (See Table 2). These data correlates with the Pfam domains enriched in the target genes regulated by the experimentally TFs, such as those AraC_binding domain (arabinose binding), FGGY_C and FGGY_N related to carbohydrate kinase family, and Aldolase_II, among others (Table 2 and Fig. 2). These tests suggest a functional relationship between carbohydrate (AraC-arabinose, FucR-fucose, RhaR-rhamnose, and GalS-galactose) transport and metabolism regulons. Therefore, based on the expression patterns, similar regulatory processes, and physiological functions, we suggest that hypothetical TFs associated with this cluster participate in carbohydrate metabolism.

**Table 2:** Functional characterization of clusters.

Cluster no.	Number of target genes	Regulatory mechanism	KEGG	Supfam	PFAM
C1	321	–	Carbohydrate metabolism; amino acid metabolism; metabolism of other amino acids	Metabolism - amino acids; carbohydrate; energy	FGGY_C;MR_MLE_C;Rieske; FGGY_N;Asp_Glu_race; BPD_transp_2; Aldolase_II;AraC_binding;
C2	272	Repressor	Replication and repair; Metabolism of other amino acids; drug resistance: antimicrobial	Information - DNA replication-repair; metabolism - other enzymes, redox; Processes_EC - toxins-defense; Processes_IC - proteases	Mur_ligase_C; Mur_ligase_M; HOK_GEF; IMS; IMS_C; IMS_HHH; Peptidase_S24; PhdYeFM_antitox; RelB; UVR; UvrD_C; UvrD-helicase
C3	371	Repressor	Amino acid metabolism; energy metabolism; nucleotide metabolism; glycan biosynthesis and metabolism; metabolism of cofactors and vitamins	Metabolism - amino acids, carbohydrate, coenzyme, transferases; Processes_IC – transport	DAHP_synth_1; GATase; LTXXQ; MGS; SBP_bac_3; SKI;CPSase_L_D3; CPSase_sm_chain; PAPS_reduct
C4	177	Activator	Lipid metabolism; membrane transport; signal transduction	Information - DNA replication-repair; regulation - signal transduction	HTH_18
C5	869	Repressor	Carbohydrate metabolism; xenobiotics biodegradation and metabolism; energy metabolism; amino acid metabolism; translation; membrane transport	General – general; metabolism – redox; Processes_EC - cell adhesion; Processes_IC – protein modification, transport	Fimbrial; molybdopterin; Molydop_binding; Molybdop_Fe4S4; Fer4_11; ABC_tran; Peripla_BP_6; Fer4_4
C6	51	Activator	Metabolism; carbohydrate metabolism; glycan biosynthesis and metabolism; drug resistance: antimicrobial	Metabolism – carbohydrate, E-transfer; regulation - kinases-phosphatases	AA_permease_2; GerE
C7	47	Activator	Amino acid metabolism; glycan biosynthesis and metabolism; metabolism of other amino acids	Information - DNA replication-repair; metabolism - other enzymes;	AA_permease_2; ABC_tran
C8	745	Dual	Energy metabolism; Lipid metabolism; amino acid metabolism; metabolism of terpenoids and membrane transport; polyketides	Information – transcription; metabolism - coenzyme other; Processes_IC - transport	FecCD; Fe-S_biosyn; Plug; TonB_dep_Rec; N_methyl
C9	474	Activator	Metabolism of other amino acids	Metab - energy transfer	BPD_transp_2; Peripla_BP_4; FGGY_C; HTH_8; FGGY_N; Proton_antipo_M
C10	939	Activator	Carbohydrate metabolism; metabolism of cofactors and vitamins; translation membrane transport	Information - DNA replication-repair; metabolism – nucleotide; other enzymes, transferases	Nitroreductase
C11	86	–	Drug resistance: antimicrobial; folding, sorting and degradation; membrane transport; signal transduction; cellular community - prokaryotes	Metab - redox	–
C12	64	Repressor	Metabolism of cofactors and vitamins; metabolism of other amino acids	Metabolism - other enzymes Processes_IC - protein modification	PaaA_PaaC

Columns are as follows: cluster number; number of target genes per cluster; enriched regulatory roles associated to target genes; functions according to Supfam, KEGG and Pfam. A P-value <0.045 were considered as threshold.

#### 3.3.2 Biosynthesis and Stress Metabolism Cluster (Cluster 2)

A total of 22 different TFs were included in cluster 2: 17 TFs experimentally described and 5 TFs identified by sequence comparisons. It is interesting that members of the GalR/LacI family (P-value = 0.0515), such as GalR and MalI, were identified as predominant. Based on RegulonDB information, 82% of the 272 target genes associated with the experimentally described dataset are negatively regulated (enrichment corrected P-value equal to 1.42E−36). Therefore, this cluster involves genes that are mainly repressed, and they are probably expressed in the absence of the TF in a holo-conformation, as has been previously suggested [18]. In functional terms, toxin-antitoxin (TA) system was found to be enriched in this cluster. Interestingly, this functional module has previously been described [19,20]. Toxins are activated upon amino acid starvation, and degraded by proteases. Moreover there is a functional link between superoxide response, DNA damage, and proteases module and TA regulons in this cluster. In addition, genes devoted to DNA-replication repair and transcription (information processes) were also identified as enriched in Supfam and KEGG functional annotations. Finally, PFAM assignments identified domains associated to Mur_ligase, peptidase, RelB, helicases and Antitoxin Phd_YefM, type II toxin-antitoxin system, associated to DNA-repair and stress responses. Table 2 and Fig. 2. Altogether, PFAM, Supfam, KEGG annotations suggest common regulatory processes related to the genes regulated by the experimentally characterized TFs, and suggesting that the five hypothetical TFs could be associated with regulation of biosynthetic metabolism or stress responses in a negative fashion, as is true for most of the regulatory processes associated with well-known TFs.

#### 3.3.3 Multiple Types of Stress Response and Anaerobic Metabolism Cluster (Cluster 9)

In cluster 9, 21 experimental and 7 hypothetical TFs were included (see Table 2 and Fig. 2). These proteins were mainly classified as members of the LysR (P-value = 0.002415236), AraC/XylS (P-value = 0.05321101), and OmpR (P-value = 0.05058147) families. In general, a total of 57% of the regulated genes are associated with positive regulation (P-value = 0.006887). Those regulated genes are mainly associated to metabolism of cofactors and vitamins and other amino acids, and cellular community processes (KEGG annotations). These functions correlate with the SUPFAM annotation (Metabolism E-transfer). Finally, PFAM assignments identified domains associated to the nitroreductase family that comprises a group of FMN- or FAD-dependent and NAD(P)H-dependent enzymes able to metabolize nitrosubstituted compounds [21] Table 2.

Therefore, it is interesting that the oxidative stress response OxyR and SoxS regulons, the GadEWX regulons [22,23] for acid stress response, and other stress-related genes (such as the low-Mg^2+^-sensitive PhoPQ two-component system, efflux system-related channel TolC, and acid stress chaperones HdeAB) clustered together. In this context of ROS and low pH signals, the shutdown of aerobic respiration (through ArcA) while NADH concentrations are sustained forces *E. coli* to overproduce oxidoreductases to prevent metabolic collapse [23,24]. For example, activation of *zwf* links glycolysis to the pentose phosphate pathway; also, it generates its finest ROS-resistant aconitase, AcnA (more stable against ROS than AcnB), which is activated by SoxS [[23], [24], [25]]. ROS-detoxifying enzyme-related genes, like *sodA*, *ahpCF*, and *katG*, are also associated with the TFs included in this cluster.

Cluster 9 also contains proteins related to metal transport, such as MntH for Mn^2+^, ZinT for Zn^2+^, and MgtA for Mg^2+^ transport, DNA repair enzymes Dps and Nfo, Fe-S cluster regeneration proteins TrxC and GrxA, and the *suf* operon product. An interesting fact is that the operon *gadEWX* was associated with TFs included in this cluster, suggesting cross-talk for regulation of transcription in the context of acid tolerance and ROS resistance. This result can be explained by MgtA being activated by both SoxR and PhoP TFs. Moreover, cross-stress protection is a phenomenon that arises in evolutionary scenarios where one stress signal provides fitness for another stressor [26]; this interconnectedness provides robustness to bacterial populations. Indeed, these regulons were identified in a cluster comprising genes for resistance to antibiotics, ROS, and organic solvents [7]. Thus, these results show that stress response genes have several expression modules that are coordinated robustly across multiple strains. All of these findings are consistent with data from the RegulonDB Gensor Units database [8]. In summary, this module may have evolved due to the requirements of certain proteins for specific metal ions, like SodA and AroF (which require Mn^2+^), TrxC, MepM, and MetE (which require Zn^2+^), and finally housekeeping enzymes like DNA polymerases and kinase (which require Mg^2+^) [23].

#### 3.3.4 Global Regulation Cluster (Cluster 10)

Cluster 10 includes 38 different TFs (see Table 2 and Fig. 2). Of these, 25 TFs experimentally described are regulating the expression of 939 genes, with the global regulators Crp and Fis the best representatives; in addition, the cluster includes 13 hypothetical TFs. The most prominent families of these regulators correspond to AraC/XylS (P-value = 0.06001661), OmpR (P-value = 0.01137062), and EBP (P-value = 0.00319223). In this cluster, regulated genes associated to TF of the cluster are preferentially activated (P-value of 1.54E−48). Based on an enrichment analysis we identified diverse regulated genes devoted to carbohydrate metabolism, mainly associated to Fructose, Pentose and Glycolysis/Gluconeogenesis, membrane transport and translation processes (KEGG annotations). Therefore, we suggest that hypothetical TFs could be involved in regulate genes devoted to carbohydrate metabolism, and transport across membrane. These data are consistent with PFAM domains identified in the dataset, such as those associated to transmembrane transport (Proton-conducting membrane transporter), Periplasmic_BP_4 and membrane transport according PFAM, with P-values of 0.0035, 0.0077 and 0.00073 respectively. In addition, two hypothetical TFs belonging to the two-component system of YqeI/YgeH were identified as being located within a cryptic genomic island corresponding to a type III secretion system, the ETT2. YgeH is homologous to a master regulator of HilA in *Salmonella enterica* serovar Typhimurium, and YqeI is a MarT homolog, which is a member of the Spi-3 pathogenicity island [27]. Both regulators are clustered with other two-component system regulators, reinforcing their probable role in oxoacid metabolic processes. Despite there still being some unidentified ETT2 TFs that could be involved in chemotaxis to increase pathogenicity [28], it is not certain to what signal(s) or histidine kinase(s) these TFs respond.

### 3.4 Concluding Remarks

In this work, by applying spectral clustering to the *E. coli* K-12 global expression data from the Colombos database, we have shown that TFs with similar expression profile patterns could regulated common processes together hypothetical regulators and this is a strategy to determine functions. Large-scale functional analysis with the KEGG, Supfam and Pfam databases provided an automated and statistically robust classification of the genes into clusters with similar physiological roles. For instance, superoxide dismutase SodA detoxifies superoxide anion and is activated by GadW and SoxS [22,23]. In turn, GadW is activated by PhoP clustered together with SoxS; thus, it is interesting to speculate whether the acid and ROS stress response modules display cross-resistance. Moreover, GadW and SoxS are members of the AraC/XylS family, consistent with our observations of overrepresentation of certain regulatory families inside the clusters.

**Fig. 3:**
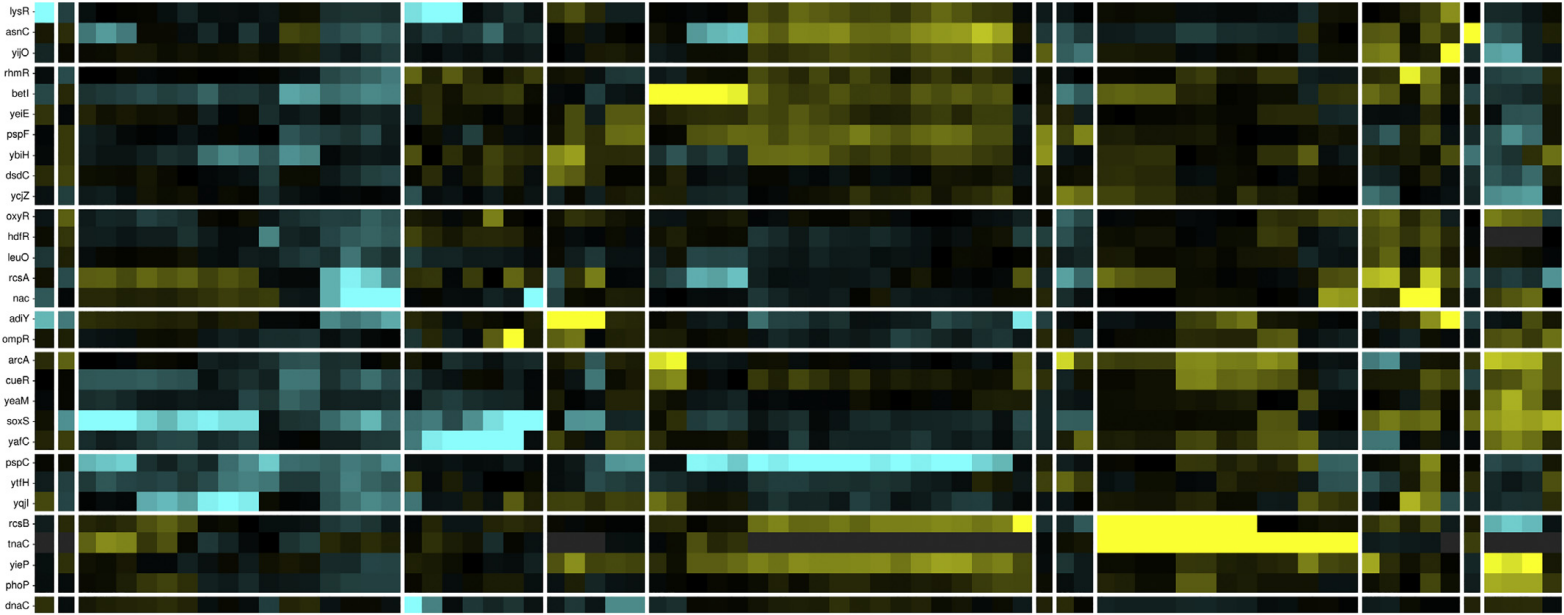
Heatmap of cluster 9 TFs (TFs for multiple types of stress response and anaerobic metabolism). Experimental and well-known TFs were mapped into Colombos, and their expression patterns are displayed.

Additionally, there are some interesting functional implications of the putative TFs in the clusters, especially for processes that are beneficial in coping with environmental changes. For example NimR, an AraC/XylS-like regulator that confers resistance to the antibacterial agent nitroimidazole [29], was clustered together with SoxS, a member of the same superfamily, and YafC (Fig. 3), a TF probably involved in resistance to ionizing radiation [30]. These findings therefore suggest that the repertoire of global stress response proteins is potentially larger than previously known, allowing our method to expand the known repertoire of genes associated with this function. Moreover, we also found interesting results related to metabolism. The YfhH putative regulator has a SIS domain (Pfam ID PF01380) that plays role in phosphosugar regulation. It is located in a genomic region next to PgpC, an enzyme that catalyzes the dephosphorylation of PGP, an essential phospholipid of the inner and outer membranes of *E. coli* K-12, suggesting that there might be a functional relationship with this membrane lipid.

Finally, the compilation and analysis of regulatory elements in *E. coli* have led us to better understand the regulatory network organization of this bacterium. Although TFs are the most extensively used elements in regulatory networks, the extended repertoire of other regulatory mechanisms has resulted in a significant increase in the versatility of the network, as it accurately modulates gene expression. Altogether, this analysis shows a strategy for functional assigment to TFs, provides new clues about the *E. coli* genetic regulation network, and such information can be determined for other organisms through gene expression databases.

The following are the supplementary data related to this article.

Supplementary material Image 1

Supplementary material Table S1 Profile expression pattern associated to hypothetical and experimental Transcription Factors. Supplementary material Table S1

Supplementary material Table S2 Comparison of clusters identified in this work against previous analysis. Supplementary material Table S2

## References

[1] Watson J.D. (2007). Towards fully automated structure-based function prediction in structural genomics: a case study. J Mol Biol.

[2] Konc J. (2013). Structure-based function prediction of uncharacterized protein using binding sites comparison. PLoS Comput Biol.

[3] Pedruzzi I. (2015). HAMAP in 2015: updates to the protein family classification and annotation system. Nucleic Acids Res.

[4] Martinez-Antonio A. (2006). Internal-sensing machinery directs the activity of the regulatory network in *Escherichia coli*. Trends Microbiol.

[5] Miroslavova N.S., Busby S.J. (2006). Investigations of the modular structure of bacterial promoters. Biochem Soc Symp.

[6] Wall M.E. (2004). Design of gene circuits: lessons from bacteria. Nat Rev Genet.

[7] Perez-Rueda E. (2015). The functional landscape bound to the transcription factors of *Escherichia coli* K-12. Comput Biol Chem.

[8] Gama-Castro S. (2011). RegulonDB version 7.0: transcriptional regulation of *Escherichia coli* K-12 integrated within genetic sensory response units (Gensor units). Nucleic Acids Res.

[9] Keseler I.M. (2017). The EcoCyc database: reflecting new knowledge about *Escherichia coli* K-12. Nucleic Acids Res.

[10] Wilson D. (2009). SUPERFAMILY—sophisticated comparative genomics, data mining, visualization and phylogeny. Nucleic Acids Res.

[11] Punta M. (2012). The Pfam protein families database. Nucleic Acids Res.

[12] Moretto M. (2016). COLOMBOS v3.0: leveraging gene expression compendia for cross-species analyses. Nucleic Acids Res.

[13] Luxur U.v. (2007). A tutorial on spectral clustering. Stat Comput.

[14] R, R.-p.D.C.T (2011). A language and environment for statistical computing.

[15] Zhou D., Yang R. (2006). Global analysis of gene transcription regulation in prokaryotes. Cell Mol Life Sci.

[16] Martinez-Antonio A. (2003). Environmental conditions and transcriptional regulation in *Escherichia coli*: a physiological integrative approach. Biotechnol Bioeng.

[17] Fang X. (2017). Global transcriptional regulatory network for *Escherichia coli* robustly connects gene expression to transcription factor activities. Proc Natl Acad Sci U S A.

[18] Balderas-Martinez Y.I. (2013). Transcription factors in *Escherichia coli* prefer the holo conformation. PLoS One.

[19] Balaban N.Q. (2004). Bacterial persistence as a phenotypic switch. Science.

[20] Page R., Peti W. (2016). Toxin-antitoxin systems in bacterial growth arrest and persistence. Nat Chem Biol.

[21] Etkind P. (1992). Pertussis outbreaks in groups claiming religious exemptions to vaccinations. Am J Dis Child.

[22] Seo S.W. (2015). Decoding genome-wide GadEWX-transcriptional regulatory networks reveals multifaceted cellular responses to acid stress in *Escherichia coli*. Nat Commun.

[23] Seo S.W. (2015). Genome-wide reconstruction of OxyR and SoxRS transcriptional regulatory networks under oxidative stress in *Escherichia coli* K-12 MG1655. Cell Rep.

[24] Kotte O. (2010). Bacterial adaptation through distributed sensing of metabolic fluxes. Mol Syst Biol.

[25] Varghese S. (2003). Contrasting sensitivities of *Escherichia coli* aconitases a and B to oxidation and iron depletion. J Bacteriol.

[26] Dragosits M. (2013). Evolutionary potential, cross-stress behavior and the genetic basis of acquired stress resistance in *Escherichia coli*. Mol Syst Biol.

[27] Ren C.P. (2004). The ETT2 gene cluster, encoding a second type III secretion system from *Escherichia coli*, is present in the majority of strains but has undergone widespread mutational attrition. J Bacteriol.

[28] Ashida H. (2011). Bacteria and host interactions in the gut epithelial barrier. Nat Chem Biol.

[29] Ogasawara H. (2015). Role of transcription factor NimR (YeaM) in sensitivity control of *Escherichia coli* to 2‑nitroimidazole. FEMS Microbiol Lett.

[30] Byrne R.T. (2014). *Escherichia coli* genes and pathways involved in surviving extreme exposure to ionizing radiation. J Bacteriol.

