# Supplementary material for: Functional Prediction of Hypothetical Transcription Factors of Escherichia coli K-12 Based on Expression Data
Source: Comput Struct Biotechnol J. 2018 Mar 27;16:157–66. doi: 10.1016/j.csbj.2018.03.003 (PMC6055005; doi:10.1016/j.csbj.2018.03.003)
Supplement: Supplementary file 1 — Supplementary material [file mmc1.docx]

**Supplementary material**

**Cluster 3**

In cluster 3, 28 well-known TFs and 7 hypothetical TFs were identified (see Table II). These proteins are mainly members of the LysR (P-value of 0.08773154), LuxR/UhpA (P-value of 0.1153141), and GalR/LacI (P-value of 3E-04) families. Of these TFs, 63% were identified as mainly negatively regulated (P-value = 4.12E-7)**.** The most enriched process identified in KEGG and SUPFAM annotations is for amino acid biosynthesis pathways (P-value of 8.15E-11), including genes associated with tryptophan, alanine, arginine, cysteine, glutamine, and glutamate biosynthesis. In addition, genes encoding the arginine succinyltransferase pathway for arginine catabolism (*ast*) and the Shikimate pathway for the biosynthesis of aromatic amino acids (*aro* operon) were also considered regulated by TFs and included in this cluster. Furthermore, the TFs PurR and DeoR, which are associated with nucleotide metabolism and carbohydrate transport and metabolism, respectively (LacI, TreR [trehalose], EbgR [galactose], UhpA [hexose phosphate tranporter]) were also present in this cluster. These overrepresented functions together PFAM domains enriched and associated to amino acid metabolism (AroM and LysM, among others) reinforce the notion of functional consistence in this cluster. This finding suggests that mostly negatively regulated biosynthetic processes are included in this cluster; therefore, hypothetical TFs could be associated with regulation of similar processes. In this regard, it has been suggested that biosynthetic genes are negatively regulated as a consequence of their requirements in the cell and that they obey the demand theory [31, 32]**.**

**Cluster 4**

In cluster 4, 19 experimentally characterized TFs and 15 hypothetical TFs were included (see Table II), classified as members of the LysR (P-value of 0.07623485), AraC/XylS (P-value of 0.1049294), OmpR (P-value of 0.09063483), LuxR/UhpA (P-value of 0.1036863), and HTH_3 (P-value of 0.02427164) familes. The most relevant observation associated with these TFs is that 57.6% of its 177 genes are positively regulated (P-value of 2.51E-02). In addition, it was found that the lipid, nucleotide metabolism and metabolism of other amino acids, and regulation of signal transductions are overrepresented using KEGG and SUPFAM functional annotations. Table II.

**Cluster 5**

Cluster 5 contains 16 known and 3 hypothetical TFs, including diverse global regulators NarL, Lrp (leucine response protein), FlhCD (for flagellar assembly), IHF (integration host factor), and HNS. The target genes from this cluster are negatively regulated (P-value of 4.86E-09). One of the most enriched biological processes is for generation of precursor metabolites and energy (P-value < 0.045), as classified by KEGG and Supfam; whereas Pfam domains are mainly devoted to transport mechanisms (P-value <0.045). In general, this cluster represents diverse biological processes associated with the global regulators. This finding is consistent with results from previous studies where global regulators were found clustered together [7].

**Cluster 6**

Cluster 6 has 6 experimentally described TFs and **4** hypothetical TFs and members of two families are significant, TetR/AcrR (P-value = 0.004) and cold shock response (P-value = 9.38E-05). The most relevant observation for these TFs is that 60% of the 51 regulated genes are positively regulated (P-value = 3.96E-02, suggesting that hypothetical TFs also play a relevant role in gene expression by an activation process. From the KEGG enrichment pathways, drug resistance: antimicrobial (P-value = 3.55E-02) mainly influenced by MarA, and ascorbate metabolism by UlaR (P-value = 3.21E-02), were identified. Moreover, it is important to note that three cold shock regulatory proteins (CspA/GH) were clustered together. Additionally, from the TetR regulatory family we found that the putative regulatory protein ComR (YcfQ) binds copper and represses ComC, an outer membrane protein involved in biofilm formation and stress response [33, 34]. In addition, it was found that four PFAM domains are overrepresented: MarB, MarR, AP_endonuc_2 and Nitroreductase (Table II).

**Cluster 7**

Cluster 7 has 5 experimentally identified TFs and 5 hypothetical TFs. Of the target genes, 85% are positively regulated (P-value = 9.42E-06). Members of the LuxR/UhpA family are overrepresentated (P=0.01348206), from which GadE, an activator of glutamate-dependent acid resistance, stands out. In addition, in this cluster we included PutA, a moonlighting protein involved in proline degradation at the cytoplasmic membrane and as a putative operon repressor, BolA, which controls cell morphology, and AidB, which is involved in DNA repair. Finally, the metabolism of amino acids and other enzymes were enriched in this cluster, with KEGG and SUPFAM functional annotations.

**Cluster 8**

Cluster 8 contains 22 experimentally characterized and 3 hypothetical TFs (see Table II), all of which are classified as members of the GntR (P-value = 0.01140669), HTH_3 (P-value = 0.008343787), and LuxR/UhpA (P-value = 0.01348206) families. A total of 745 regulated genes are associated with the 20 TFs for which the global regulator Fnr was identified. Of these, 53% of the genes are regulated with both positive and negative mechanisms (dual regulation) (P-value = 7.94E-05). This cluster is involved mostly with membrane transport, and metabolism of terpenoids and polyketides, among others (KEGG annotations) with PFAM domains also involved in similar processes, such as TonB_dep_Rec (Table II).

**Cluster 11**

Cluster 11 comprises 11 experimental and 5 hypothetical TFs, which together control 86 TGs, with 54% for activation, 46% involved in dual regulation, and none with a repression mechanism. The most relevant biological processes are organic cyclic compound metabolism and phenylacetate/xenobiotic catabolism (KEGG) and metabolism redox (Supfam). Like Cluster 6, we found three cold shock proteins clustered together (CspB/F/I).

**Cluster 12**

Cluster 12 is one of the smallest clusters, comprising 64 target genes, regulated by 12 known and 10 putative TFs. This cluster has a slight overrepresentation of the GntR regulatory family (P-value = 0.03199464). Metabolism of other amino acids and other enzymes are associated with this cluster, due to the PaaX and BirA regulators. Another interesting TF in this cluster is NorR, which is involved in nitric oxide reduction and detoxification in response to reactive nitrogen species. In this module, the most enriched function is repression (P-value = 6.89E-03) and the Pfam PaaA_PaaC domain (P-value 2.16E-03).

**References**

**[31]** M.A. Savageau, Demand theory of gene regulation. II. Quantitative application to the lactose and maltose operons of Escherichia coli, Genetics **149** (4), 1998, 1677–1691.

**[32]** M.A. Savageau, Demand theory of gene regulation. I. Quantitative development of the theory, Genetics **149** (4), 1998, 1665–1676.

**[33]** M. Mermod, et al., The copper-inducible ComR (YcfQ) repressor regulates expression of ComC (YcfR), which affects copper permeability of the outer membrane of Escherichia coli, Biometals **25** (1), 2012, 33–43.

**[34]** X.S. Zhang, et al., YcfR (BhsA) influences Escherichia coli biofilm formation through stress response and surface hydrophobicity, J Bacteriol **189** (8), 2007, 3051–3062.
