# Supplementary material for: Functional Prediction of Hypothetical Transcription Factors of Escherichia coli K-12 Based on Expression Data
Source: Comput Struct Biotechnol J. 2018 Mar 27;16:157–66. doi: 10.1016/j.csbj.2018.03.003 (PMC6055005; doi:10.1016/j.csbj.2018.03.003)
Supplement: Supplementary material Table S1 — Profile expression pattern associated to hypothetical and experimental Transcription Factors. [file mmc2.pdf]

		Test descript	test_E-TABM	test_E-TABM	test_E-MEXP	test_E-MEXP
		Reference de	ref_E-TABM-	ref_E-TABM-	test_E-MEXP	test_E-MEXP
		Experiment_	E-TABM-103	E-TABM-103	E-MEXP-926	E-MEXP-926
		Data source	ArrayExpress	ArrayExpress	ArrayExpress	ArrayExpress
		Platform	affymetrix	affymetrix	affymetrix	affymetrix
		Condition properties: valu		TIME:90	83972:1,VR5	83972:1,VR5
LocusTag	Gene name	Geneid/Cont	7	8	67	71
b0020	nhaR_Exp	19	0.22819	-0.17681	-0.787492	-0.747549
b0034	caiF_Exp	33	0.23921	0.052925	-0.266336	0.338307
b0064	araC_Exp	62	-0.47112	-0.45864	-0.27398	-0.988168
b0069	sgrR_Exp	67	-0.15713	-0.11075	-0.56114	-0.81084
b0076	leuO_Exp	74	-0.10545	0.0095883	0.265272	0.531406
b0080	fruR_Exp	77	-0.1734	-0.21614	-0.394929	0.336332
b0113	pdhR_Exp	108	0.56183	-0.2611	1.27341	3.6988
b0146	sfsA_hyp	141	-0.17246	-0.41675	0.435795	0.27701
b0162	cdaR_Exp	157	0.11852	-0.029368	0.0826484	0.0812009
b0208	yafC_hyp	197	-0.089282	-0.2281	0.238706	1.02949
b0232	yafN_hyp	222	-0.18512	-0.21105	-1.59649	-1.21862
b0240	crl_hyp	230	-0.8522	-0.9495	0.231488	0.342868
b0254	perR_hyp	247	-0.11701	-0.026235	0.318459	0.330396
b0272	yagI_hyp	265	0.29265	-0.084612	0.0513304	-0.0170462
b0294	matA_hyp	287	-0.17507	-0.085599	2.24335	0.670976
b0305	ykgD_hyp	300	-0.14842	0.018772	0.195056	0.208995
b0313	betI_Exp	307	-0.30119	-0.3599	1.4845	1.9938
b0315	yahA_hyp	309	0.039761	-0.067697	0.62067	-0.873867
b0316	yahB_hyp	310	-0.22359	0.0025129	-0.176225	-0.138556
b0330	prpR_Exp	323	-0.18839	-0.014177	-0.309423	-0.398986
b0338	cynR_Exp	330	-0.16225	-0.13549	-2.10515	-1.21018
b0345	lacI_Exp	337	-0.11788	-0.149	-0.36437	0.087042
b0346	mhpR_Exp	338	-0.50622	-0.61697	-3.50263	-2.02466
b0394	mak_Exp	383	-0.037578	-0.1603	0.71982	-0.2939
b0399	phoB_Exp	387	0.2958	-0.14989	0.388952	0.449661
b0413	nrdR_Exp	401	0.037486	-0.048897	0.166128	0.844289
b0435	bolA_Exp	423	-0.089772	-0.33634	0.78315	1.3162
b0440	hupB_Exp	428	-0.22963	-0.44195	-0.719618	-1.26757
b0447	ybaO_hyp	435	0.051743	-0.24207	0.037925	3.8727
b0460	hha_hyp	447	0.01721	-0.47379	-0.410578	2.36407
b0464	acrR_Exp	451	-0.24921	-0.20112	0.0854749	0.818918
b0483	ybaQ_hyp	470	-0.16849	-0.65531	-1.48658	0.861412

b0487	cueR_Exp	474	0.12972	-0.12333	-1.26965	0.116984
b0504	allS_Exp	491	0.1203	0.1168	-0.0491765	0.535507
b0506	allR_Exp	493	0.26952	0.022806	-0.189191	-0.23517
b0535	fimZ_hyp	521	-0.27099	-0.099246	0.241044	-0.0238235
b0545	ybcL_hyp	531	0.048294	0.026122	0.198025	0.0493473
b0546	ybcM_hyp	532	0.017649	-0.16354	0.0775428	0.0542591
b0564	appY_Exp	555	0.067015	0.18475	0.0639107	0.124887
b0566	envY_Exp	557	0.10018	-0.050187	-0.0137531	-0.0385672
b0571	cusR_Exp	562	0.054354	-0.12032	0.115816	0.199677
b0603	ybdO_hyp	597	-0.040708	0.059854	-0.0198956	-0.201135
b0620	dpiA_Exp	613	-0.15091	-0.033128	0.270811	0.519809
b0623	cspE_hyp	616	-0.26151	-0.51818	-0.459701	-0.0527988
b0629	ybeF_hyp	621	0.065715	-0.034293	-0.230278	-0.433736
b0676	nagC_Exp	657	0.086203	-0.17333	0.154709	0.611817
b0683	fur_Exp	664	0.037303	-0.34018	-0.0774842	1.62792
b0685	ybfE_hyp	667	0.53731	-0.20724	0.0877444	2.46474
b0694	kdpE_Exp	677	-0.028324	-0.039307	0.00118002	-0.0407981
b0730	mngR_Exp	713	0.12762	-0.10816	-2.18209	-1.05797
b0761	modE_Exp	738	0.086802	-0.21159	-0.319533	0.407486
b0768	ybhD_hyp	745	-0.10643	0.025701	0.301237	0.328683
b0796	ybiH_hyp	774	-0.2765	-0.19948	0.104572	1.03023
b0803	ybiI_hyp	781	0.26513	0.05935	0.716397	2.53637
b0817	mntR_Exp	795	0.23004	-0.22839	-0.0670237	0.802941
b0840	deoR_Exp	818	-0.016614	-0.098068	0.0651404	0.435217
b0846	ybjK_hyp	824	-0.0057302	-0.0333	-0.228856	0.0940017
b0880	cspD_hyp	858	-0.96799	-0.66827	-1.30578	-1.30676
b0889	lrp_Exp	866	-0.16909	-0.55541	-0.565234	-1.5905
b0900	ycaN_hyp	877	0.095252	-0.11532	-0.390474	-0.699208
b0912	ihfB_Exp	889	0.039003	-0.18324	-0.55178	-1.07745
b0989	cspH_hyp	967	0.020058	0.18918	0.344222	5.76891
b0990	cspG_hyp	968	0.046986	-0.15601	0.120786	6.82637
b0995	torR_Exp	974	-0.079716	-0.044481	0.00222562	0.194301
b1013	rutR_Exp	992	-0.050338	-0.12058	0.7903	0.777732
b1014	putA_Exp	993	-0.43954	-0.54852	1.62943	-0.275862
b1040	csgD_Exp	1015	0.043073	-0.12427	0.104434	0.40438
b1111	ycfQ_hyp	1085	0.16745	0.031254	0.097699	3.60142
b1130	phoP_Exp	1104	-0.15221	-0.30356	-0.553823	0.0645982
b1147	ymfL_hyp	1120	0.41538	-0.1958	0.0934952	0.0531516
b1162	ycgE_hyp	1136	-0.16645	-0.14066	-0.589167	2.0525
b1187	fadR_Exp	1163	0.21034	-0.089343	0.068603	1.03342
b1201	dhaR_Exp	1177	-0.23002	-0.037822	-0.295871	-0.207412
b1221	narL_Exp	1200	-0.052033	0.5311	-0.241907	-0.463682

b1237	hns_Exp	1214	-0.30124	-0.58257	-0.232172	-0.164649
b1275	cysB_Exp	1252	0.23933	-0.16006	-0.679754	1.89028
b1284	yciT_hyp	1263	0.27465	0.03163	-0.366037	0.947203
b1299	puuR_hyp	1279	0.047996	-0.0085309	-2.71889	-1.40424
b1303	pspF_Exp	1283	0.24974	0.15893	0.380965	1.37803
b1306	pspC_hyp	1286	0.27152	0.16958	0.393036	-0.213966
b1320	ycjW_hyp	1300	0.093642	0.080131	-1.19604	-0.763198
b1323	tyrR_Exp	1303	0.12249	0.031946	-0.29997	-0.138875
b1328	ycjZ_hyp	1309	-0.0065486	0.0055402	-0.0341476	0.447981
b1334	fnr_Exp	1315	0.093766	-0.2278	-0.526283	1.30132
b1339	abgR_hyp	1320	0.049254	0.045011	-0.603488	-0.260918
b1357	ydaS_hyp	1339	0.034753	0.07209	-0.0181312	-0.0937886
b1384	feaR_Exp	1366	0.04234	-0.060569	-1.2617	-0.712941
b1399	paaX_Exp	1381	0.19179	-0.10689	-2.01563	-1.30385
b1422	ydcI_hyp	1403	-0.31317	-0.35	-0.53319	1.0537
b1434	ydcN_hyp	1415	0.18395	-0.03554	-0.502437	1.16159
b1438	ydcQ_hyp	1419	0.10401	-0.02302	-1.15428	-0.695379
b1439	ydcR_hyp	1420	0.15299	-0.19636	-2.26264	-0.990638
b1450	yncC_Exp	1432	0.047318	0.083427	-1.1155	-0.71756
b1477	yddM_hyp	1458	0.068164	-0.20097	0.825437	0.319666
b1499	ydeO_Exp	1480	-0.29504	-0.03054	0.530793	-0.0589356
b1507	hipA_Exp	1488	0.054561	0.00048154	-1.3328	-0.51941
b1508	hipB_Exp	1489	-0.033969	-0.23074	1.32804	1.63907
b1512	lsrR_Exp	1493	0.086911	-0.28488	-1.53659	-0.959221
b1526	yneJ_hyp	1507	0.17013	-0.11405	-0.282457	1.30802
b1530	marR_Exp	1511	0.24136	-0.11133	-0.226207	5.81692
b1531	marA_Exp	1512	0.0063502	-0.3354	0.738124	5.61655
b1540	ydfH_hyp	1522	0.071181	-0.0080944	-0.200606	2.55363
b1552	cspI_hyp	1535	0.0023404	0.01832	0.38079	-0.15387
b1557	cspB_hyp	1540	0.19942	0.038146	0.040655	0.030638
b1558	cspF_hyp	1541	0.19191	0.022839	0.28245	-0.10181
b1563	relE_Exp	1546	0.15455	0.011405	0.0097547	0.069917
b1564	relB_Exp	1547	0.066381	-0.10233	0.15597	0.059569
b1569	dicC_hyp	1552	0.10718	-0.22006	0.14806	-0.12116
b1570	dicA_Exp	1553	0.17424	-0.13186	0.12654	0.079329
b1594	mlc_hyp	1576	-0.29198	-0.41778	-0.393775	0.0535089
b1595	ynfL_hyp	1577	0.022293	0.12835	-0.243678	-0.167043
b1608	rstA_Exp	1591	-0.0090901	-0.083625	0.15002	-0.134965
b1618	uidR_Exp	1601	0.024276	-0.068769	-0.254704	2.21387
b1620	malI_Exp	1603	-0.019586	0.033971	0.476932	2.97161
b1625	cnu_hyp	1609	0.21665	-0.26217	0.679361	0.972004
b1642	slyA_Exp	1626	-0.10247	-0.26437	-0.232543	0.221364

b1649	ydhM_hyp	1633	-0.15413	-0.077929	0.0233242	4.07014
b1658	purR_Exp	1643	0.38502	0.073949	-0.28286	-0.949926
b1659	ydhB_hyp	1644	0.12388	-0.13118	-0.206676	0.884606
b1696	ydiP_hyp	1679	0.25577	-0.040669	-0.147928	0.029985
b1703	ydiA_hyp	1686	-0.007547	0.080161	0.342172	-0.587717
b1712	ihfA_Exp	1695	0.060315	-0.1374	0.264093	-0.685076
b1735	chbR_Exp	1718	0.38811	0.097991	0.0923947	0.617344
b1770	ydjF_hyp	1753	0.071768	-0.085374	-0.192734	0.748823
b1790	yeaM_hyp	1773	0.042908	-0.095965	-0.540191	0.831122
b1799	yeaT_hyp	1782	-0.39687	-0.15628	-1.18679	-0.767695
b1823	cspC_hyp	1806	-0.32524	-0.12883	-0.0324731	-0.297657
b1827	kdgR_Exp	1811	0.099164	-0.18508	-0.172558	0.714457
b1853	yebK_hyp	1837	-0.20602	-0.33524	-0.933668	-0.812884
b1891	flhC_Exp	1875	-0.22848	-0.11605	-1.60505	-0.344723
b1892	flhD_Exp	1876	-0.46443	-0.20974	-1.61633	0.784365
b1914	uvrY_hyp	1894	-0.0099431	-0.18566	0.420392	1.56498
b1916	sdiA_Exp	1896	-0.063189	-0.38083	0.00256154	0.410988
b1951	rcsA_Exp	1929	-0.054951	-0.036437	1.54388	-0.0400491
b1969	yedW_hyp	1944	0.16312	-0.08305	0.204744	2.11634
b1987	cbl_Exp	1957	-0.083762	0.034348	-1.7636	-1.25973
b1988	nac_Exp	1958	-0.15089	0.049186	-0.359989	0.92116
b2015	yeeY_hyp	1988	-0.19317	0.20257	0.222089	3.7538
b4539	yoeB_Exp	1990	0.079642	-0.016387	-0.71642	1.1151
b2017	yefM_Exp	1991	-0.20959	0.053114	0.103282	2.93635
b2079	baeR_Exp	2053	-0.096259	-0.18083	-0.28896	0.69247
b2082	ogrK_hyp	2056	0.1834	-0.082917	0.0331554	0.222458
b2101	yegW_hyp	2073	-0.07785	0.035948	0.294179	1.19075
b2105	rcnR_Exp	2077	-0.0054793	0.004528	-0.576394	-0.848982
b2125	yehT_hyp	2096	0.017882	-0.13615	-0.704221	0.338997
b2127	mlrA_Exp	2098	0.00046255	0.13572	0.7717	2.4807
b2151	galS_Exp	2122	-0.17018	-0.36688	-0.214806	0.368096
b2157	yeiE_hyp	2128	0.0034918	-0.099241	0.355408	0.85285
b2160	yeiI_hyp	2131	0.11548	0.16095	-2.46575	-1.63346
b2163	yeiL_Exp	2134	0.079762	-0.012459	-0.117347	0.442119
b2193	narP_Exp	2163	0.27889	0.29334	-0.298318	1.6739
b2213	ada_Exp	2184	0.17134	0.035423	0.19505	-0.153141
b2217	rcsB_Exp	2188	-0.053511	-0.074573	-0.504917	0.0209398
b2220	atoC_Exp	2191	0.098272	-0.0271	-0.45827	-0.253346
b2248	yfaX_hyp	2220	0.045723	0.053019	0.60385	0.256233
b2289	lrhA_Exp	2262	0.048544	-0.087628	-0.914631	2.03798
b2364	dsdC_Exp	2337	-0.20781	-0.1366	-0.668356	-0.644855
b2369	evgA_Exp	2342	-0.22568	-0.24396	0.34487	3.7562

b2382	ypdC_hyp	2355	0.24439	0.027258	0.261153	0.514995
b2405	xapR_Exp	2371	-0.065708	-0.018971	-1.09422	-0.560344
b2409	yfeR_hyp	2375	0.14894	-0.0064269	-0.0680061	0.450237
b2427	yfeT_hyp	2394	-0.12867	-0.34655	-0.855596	-0.865983
b2437	eutR_hyp	2404	0.069858	0.039944	-0.215605	-0.0949398
b2491	hyfR_Exp	2460	0.13641	-0.093957	-0.0608592	0.0449566
b2516	yfgA_hyp	2485	-0.050751	-0.21584	-3.0214	-1.85234
b2531	iscR_Exp	2500	-0.12017	-1.074	-0.115815	1.9719
b2537	hcaR_Exp	2506	-0.26522	-0.25361	-2.49357	-1.36771
b2550	yphH_hyp	2519	0.065079	0.026667	-0.411287	-0.47488
b2554	yfhA_hyp	2523	-0.037874	-0.019287	-0.0070398	-0.367098
b2561	yfhH_hyp	2530	-0.0036431	-0.066538	0.3974	0.328839
b2577	yfiE_hyp	2546	-0.010118	-0.11433	-1.89099	-1.30021
b2624	alpA_Exp	2586	-0.0025008	0.026621	-0.0156666	0.154342
b2664	csiR_Exp	2618	-0.026548	0.27758	-0.255669	-0.442111
b2667	ygaV_hyp	2621	0.0076737	0.02294	-0.388056	1.25936
b2669	stpA_Exp	2623	0.3544	-0.2266	0.513699	-0.976866
b2684	mprA_Exp	2637	0.10093	-0.090912	0.498033	1.65347
b2697	alaS_Exp	2645	0.0086957	-0.39768	-0.0552952	-0.297201
b2706	gutM_Exp	2654	-0.29414	-0.088748	0.0620095	0.0917456
b2707	srlR_Exp	2655	-0.11036	-0.11597	-0.224211	-0.762819
b2709	norR_Exp	2657	0.0523	-0.11982	-1.53766	-0.745092
b2714	ascG_Exp	2662	0.097054	0.0052365	-2.38724	-1.28646
b2725	hycA_hyp	2673	-0.08136	0.032255	0.542484	0.0434557
b2731	fhlA_Exp	2679	0.097117	0.047439	-0.759299	-0.539787
b2735	ygbI_hyp	2683	-0.12679	-0.0056654	-0.0868793	1.1708
b2783	mazE_hyp	2730	-0.14594	-0.20567	-3.73592	-2.55695
b2805	fucR_Exp	2752	-0.69177	-0.49835	-0.0496721	-0.290848
b2808	gcvA_Exp	2755	0.14729	-0.048198	0.251294	2.51744
b2837	galR_Exp	2782	-0.2329	0.062818	0.123673	1.87805
b2839	lysR_Exp	2784	0.017239	0.016957	0.346148	0.463345
b2846	yqeH_hyp	2791	-0.36838	-0.036065	-0.174844	-0.307253
b2847	yqeI_hyp	2792	0.018846	0.11564	-0.159025	-0.59168
b2852	ygeH_hyp	2797	-0.35491	0.045402	0.0141835	-0.179839
b2869	ygeV_hyp	2810	-0.2168	-0.043201	0.550777	1.48504
b2916	argP_Exp	2855	0.045294	-0.034863	-0.393144	0.296444
b2921	ygfI_hyp	2860	-0.13836	0.034343	0.13627	0.9092
b2929	yggD_hyp	2868	-0.051044	-0.0046291	-0.381198	0.290398
b2980	glcC_Exp	2917	-0.48366	-0.18122	-0.281009	1.90453
b3010	yqhC_Exp	2944	-0.023927	0.006346	-0.17409	0.47173
b3021	ygiT_hyp	2954	-0.095321	0.025209	-1.79373	-1.28951
b3022	mqsR_Exp	2955	-0.15932	-0.04308	-2.97431	-2.06402

b3025	qseB_Exp	2958	-0.028345	0.10691	0.0611333	-0.265238
b3060	ttdR_Exp	2992	-0.13787	0.16092	0.624959	0.95459
b3071	yqjI_Exp	3002	-0.13556	0.0335	-0.0071011	0.173049
b3075	ebgR_hyp	3006	-0.23416	0.01923	-0.068756	0.652823
b3082	ygjM_hyp	3013	-0.11217	0.0895	0.549334	0.998458
b3094	exuR_Exp	3025	-0.23716	0.041989	-0.0461332	3.01031
b3105	yhaJ_hyp	3036	-0.21627	-0.081614	0.0623572	-0.532718
b3118	tdcA_Exp	3047	-0.035734	0.18235	0.429749	1.61611
b3119	tdcR_Exp	3048	-0.37328	0.2432	0.873756	0.0833455
b3131	agaR_Exp	3058	-0.13714	-0.17895	-0.24825	0.63515
b3188	sfsB_hyp	3113	-0.38134	0.13921	0.00255025	0.661217
b3226	nanR_Exp	3151	-0.18742	-0.028117	0.0176127	1.23585
b3237	argR_Exp	3162	0.38086	0.036701	-0.589159	-0.185679
b3243	aaeR_hyp	3168	-0.206	0.10566	0.234922	0.298595
b3261	fis_Exp	3185	0.43093	-0.32974	-0.0993056	-1.12651
b3264	envR_Exp	3188	-0.075127	-0.054771	0.811015	0.314778
b3292	zntR_Exp	3209	-0.33023	-0.084331	0.0445818	2.72587
b3357	crp_Exp	3275	-0.31603	0.00037224	0.221354	0.273152
b3375	frlR_hyp	3292	-0.25831	0.23854	-2.80538	-1.98657
b3405	ompR_Exp	3322	-0.40705	-0.17175	0.71562	1.44649
b3418	malT_Exp	3335	-0.67622	-0.25248	-2.07086	-1.69508
b3422	rtcR_Exp	3338	-0.056141	0.11165	-0.004407	-0.333845
b3423	glpR_Exp	3339	0.072314	0.046477	0.268185	-0.348567
b3438	gntR_Exp	3353	-0.027394	0.085767	0.133695	2.98639
b3481	nikR_Exp	3397	-0.23888	0.20352	0.278251	0.411042
b3501	arsR_Exp	3418	-0.15064	0.10053	-0.878131	-0.59514
b3507	dctR_hyp	3424	-0.2932	0.15041	1.12217	1.11442
b3512	gadE_Exp	3429	-0.11054	0.54653	3.28768	1.76636
b3515	gadW_Exp	3432	-0.39521	0.09591	-2.71344	-2.48936
b3516	gadX_Exp	3433	-0.14424	0.042922	-2.35356	-1.76761
b3520	yhjB_hyp	3437	-0.36205	0.12327	0.335632	0.556123
b3521	yhjC_hyp	3438	-0.26758	-0.028815	0.486768	0.0829869
b3555	yiaG_hyp	3472	-0.32675	0.20629	0.807266	1.10954
b3556	cspA_Exp	3473	0.76253	-0.69977	-0.142651	2.45989
b3569	xylR_Exp	3489	-0.14724	0.085985	-0.731111	-0.592399
b3574	yiaJ_Exp	3494	-0.34543	0.16545	-0.395519	1.93756
b3585	yiaU_hyp	3507	-0.28398	0.04186	-1.31444	-0.774511
b3601	mtlR_Exp	3527	-0.024678	0.14828	0.00309594	0.339029
b3604	lldR_Exp	3531	-1.3903	-1.2116	-2.76423	-1.52552
b3641	slmA_hyp	3568	-0.17629	0.2794	0.606397	-0.43678
b3669	uhpA_Exp	3597	0.030366	0.28582	-0.958681	-0.0218078
b3680	yidL_hyp	3610	-0.36512	0.25968	0.671615	0.392627

b3684	yidP_hyp	3613	-0.4978	0.32399	0.0697418	-0.238451
b4479	dgoR_hyp	3624	-0.013292	0.083509	0.273	0.70801
b3702	dnaA_Exp	3631	0.17869	0.099158	-0.635155	0.102797
b3711	yidZ_hyp	3641	-0.3969	0.27322	0.347426	1.14637
b3743	asnC_Exp	3673	-0.16967	-0.085931	-0.276392	0.286965
b3753	rbsR_Exp	3683	-0.33364	0.0046144	0.383027	-1.17E-05
b3755	yieP_hyp	3685	-0.090893	0.26764	0.397701	1.3049
b4480	hdfR_Exp	3686	-0.27884	-0.013759	0.49619	0.54845
b3773	ilvY_Exp	3695	-0.033507	0.13627	-0.0924062	1.43132
b3828	metR_Exp	3744	0.030973	0.0452	-0.92943	-1.2475
b3868	glnG_Exp	3776	0.059834	0.057311	-2.40014	-1.36253
b3872	yihL_hyp	3780	-0.12133	-0.15942	-0.0632968	-0.318822
b3884	yihW_hyp	3792	-0.21516	0.041495	-2.53082	-1.52277
b3897	frvR_hyp	3805	-0.12215	0.13526	0.268949	0.0263362
b3905	rhaS_Exp	3813	-0.2371	0.19955	0.746281	0.0369855
b3906	rhaR_Exp	3814	-0.23815	0.13698	0.407793	0.24311
b3912	cpxR_Exp	3820	-0.3871	-0.097046	0.272201	0.392951
b3934	cytR_Exp	3841	-0.25673	0.023158	-0.453824	0.275306
b3938	metJ_Exp	3845	0.14398	0.17553	-0.659935	-0.340898
b3954	yijO_hyp	3860	-0.25775	0.010681	-0.040559	0.0856099
b3961	oxyR_Exp	3867	-0.038634	-0.048606	0.0535559	-0.194138
b3963	fabR_Exp	3869	-0.40892	-0.26297	0.488012	0.680069
b3973	birA_Exp	3875	-0.023639	0.048163	-2.6059	-1.8937
b4000	hupA_Exp	3898	-0.46086	-0.48504	-0.0944282	-1.12389
b4004	zraR_Exp	3902	0.071892	-0.049487	-0.0712165	-0.18437
b4018	iclR_Exp	3912	0.070663	-0.14742	0.0692961	1.06513
b4043	lexA_Exp	3938	-0.16857	-0.16893	-0.0728906	1.2935
b4046	zur_Exp	3941	-0.12722	0.084149	-1.1092	-0.60934
b4062	soxS_Exp	3958	-0.21073	-0.17135	0.604036	0.0967811
b4063	soxR_Exp	3959	-0.2525	0.25264	0.737723	2.90366
b4089	rpiR_Exp	3986	0.014506	0.21915	-0.102471	-0.27724
b4102	phnF_hyp	3999	-0.28718	0.25596	0.769514	0.162249
b4113	basR_hyp	4009	-0.33518	0.10301	-3.803	-2.9805
b4116	adiY_Exp	4012	-0.23161	0.25311	1.00607	1.50949
b4118	melR_Exp	4014	-0.17176	0.098503	0.419881	0.863905
b4124	dcuR_Exp	4020	-0.1727	0.03813	0.22841	-0.375907
b4133	cadC_Exp	4030	-0.16943	0.16336	0.677982	1.59552
b4135	yjdC_hyp	4032	-0.4823	-0.0008719	0.622246	0.562471
b4178	nsrR_Exp	4074	-0.056421	-0.45729	-0.128748	2.46691
b4187	aidB_Exp	4083	0.087271	0.2894	0.632814	0.352353
b4191	ulaR_Exp	4087	-0.22315	0.1292	0.0359301	2.31883
b4212	ytfH_hyp	4108	0.04235	0.11256	0.316639	4.10447

b4224	chpS_hyp	4119	0.11219	-0.031434	0.238727	0.274168
b4241	treR_Exp	4135	-0.12544	0.00092796	-0.159693	0.135368
b4251	yjgJ_hyp	4143	-0.057798	-0.021386	-1.32474	-1.09717
b4260	pepA_Exp	4152	-0.070254	-0.053804	0.0721383	-0.381246
b4264	idnR_Exp	4156	0.051248	0.13044	-0.111166	-0.0444471
b4295	yjhU_hyp	4186	-0.040704	0.087772	-2.85415	-2.0098
b4299	yjhI_hyp	4190	-0.34018	0.14492	-0.38397	-0.444738
b4300	sgcR_hyp	4191	-0.34331	0.17161	-0.27308	-0.481728
b4324	uxuR_Exp	4219	0.04755	0.0577	0.156041	1.32967
b4327	yjiE_hyp	4222	-0.077568	-0.040642	-0.34774	0.47839
b4340	yjiR_hyp	4234	-0.14625	0.022548	-1.31532	-0.978
b4357	yjjM_hyp	4250	-0.68441	0.20499	-0.305305	-0.270852
b4365	yjjQ_hyp	4258	-0.19135	0.13179	0.634431	0.747892
b4366	bglJ_Exp	4259	-0.45748	0.15533	0.73861	0.89074
b4385	yjjJ_hyp	4277	0.059229	0.017903	0.0147635	0.140031
b4390	nadR_Exp	4282	-0.028538	0.060392	0.115995	-0.126877
b4393	trpR_Exp	4285	-0.0026662	-0.074585	0.229905	-0.390419
b4396	rob_Exp	4288	-0.11336	0.1493	-0.10453	0.894747
b4398	creB_Exp	4290	0.056076	0.016058	0.313527	-0.254681
b4401	arcA_Exp	4293	-0.48201	-0.12348	0.608591	2.53192

test_E-MEXP	test_E-MEXP	test_E-MEXP	test_E-MEXP	test_E-MEXP	test_E-MEXP	test_E-MEXP
test_E-MEXP	test_E-MEXP	test_E-MEXP	test_E-MEXP	test_E-MEXP	ref_E-MEXP-	ref_E-MEXP-
E-MEXP-926	E-MEXP-926	E-MEXP-926	E-MEXP-926	E-MEXP-926	E-MEXP-137(	E-MEXP-137(
ArrayExpress	ArrayExpress	ArrayExpress	ArrayExpress	ArrayExpress	ArrayExpress	ArrayExpress
affymetrix	affymetrix	affymetrix	affymetrix	affymetrix	affymetrix	affymetrix
83972:1,VR5	83972:1,VR5	HUMAN_URI	HUMAN_URI	HUMAN_URI	relA_b2784:-	relA_b2784:-
72	73	79	80	81	138	139
-0.477536	-0.480511	0.06699	-0.04313	0.207997	0.21113	1.3555
0.0897533	0.846272	0.0395003	-0.040418	0.036057	-1.8856	-1.9379
-0.789014	-0.770101	-0.233127	-0.474816	-0.109717	0.53026	0.42847
-0.676467	-0.700357	-0.506644	-0.486856	-0.571365	-0.44591	-0.42627
0.393352	0.0845188	-0.115609	-0.0079763	-0.0472185	0.030524	0.17006
0.295885	0.413315	0.984083	1.06646	1.12437	-0.41491	-0.19681
3.66525	3.71775	3.7027	3.82286	3.97689	-1.5222	-0.62276
0.244173	0.413563	1.15469	1.41979	1.16227	-0.56541	-0.66532
0.175065	-0.0542832	0.0402616	0.133539	0.122439	0.18205	0.68683
0.990498	1.08282	0.895291	1.05761	1.1442	0.039675	-0.31412
-1.06949	-1.12738	1.12204	1.17984	0.794692	1.0431	1.3829
0.117823	0.533467	0.774455	0.771315	1.03419	1.0673	2.1662
0.191888	0.226308	-0.0799646	-0.0665332	-0.141434	0.19811	0.048667
0.0761114	0.118639	-0.135702	0.00642362	-0.15087	0.93563	1.9945
0.601947	0.0188528	-0.182224	-0.334661	-0.253965	NaN	NaN
0.248278	0.0975606	-0.10563	0.0100308	-0.039474	-0.10815	0.096825
1.9232	1.9544	0.17096	-0.43313	0.16447	1.4095	1.406
-0.698481	-1.2327	-2.05049	-1.89162	-2.03955	1.5842	1.9109
-0.0800854	-0.225194	0.298	0.180916	0.125138	-0.15341	-0.50178
-0.399845	-0.407249	-0.116812	-0.252618	-0.173222	-0.51145	-0.33203
-1.13271	-0.95246	0.0636389	0.0103584	-0.0187512	0.14653	0.032198
0.25159	-0.33368	0.083537	-0.18016	-0.050408	-0.065583	-0.43592
-1.86736	-1.26546	0.336475	0.216578	0.346835	0.12863	-0.037378
-0.3975	-0.041785	-0.20605	-0.11111	0.012785	NaN	NaN
0.638442	0.17422	0.0947148	-0.130288	-0.290933	0.75735	0.39415
0.554516	1.23003	0.512422	0.559887	0.595427	0.1803	0.33783
1.05	1.7844	1.2086	1.6457	1.2356	0.26892	-0.13938
-1.35472	-0.819666	-0.812829	-0.5353	-0.489386	-0.24911	-0.91727
3.3205	4.7776	3.429	3.936	3.814	0.37837	0.40726
2.49526	2.69632	2.03219	1.93767	2.01638	1.0336	1.7592
0.648185	0.500879	1.15782	0.922579	1.1389	0.8974	0.9584
0.886186	1.13086	0.201709	0.141979	0.168566	-0.35942	0.17106

0.035343	0.707784	1.03762	1.45621	1.41479	0.37601	0.88729
0.614346	0.656706	0.44034	0.629939	0.688321	-0.31379	0.087009
-0.0461627	-0.212147	0.535843	0.339391	0.645702	-0.033098	-0.14271
-0.11845	-0.0569474	0.0687072	-0.0387799	0.164413	-0.10022	0.15986
0.039667	0.0479519	0.0906693	-0.0186355	-0.0383898	0.81041	0.60597
-0.0710555	-0.0897294	0.00403393	-0.0820595	0.00074599	1.3117	1.329
0.156512	-0.0387552	0.0673836	0.00037737	0.0207937	-0.43569	-0.20809
-0.296673	-0.267554	0.325522	0.0734551	0.0286686	-0.42439	-0.31717
0.0428035	0.410805	0.28167	0.271023	0.183944	0.0084156	0.20317
-0.151937	-0.132528	0.372788	0.238889	0.132155	-0.25773	-0.057824
0.516974	0.34539	-0.0550233	0.159957	0.0299239	0.60749	1.0033
-0.0380378	0.429201	1.09347	1.14637	1.49626	-0.6676	-2.0225
-0.417547	-0.539077	0.00819656	-0.0988903	-0.144067	0.42418	0.37087
0.569643	0.322236	0.55118	0.142742	0.276543	-1.4832	-1.3543
1.5584	1.66976	1.78955	1.73953	1.74697	0.26832	0.17277
2.62778	1.51029	1.15144	1.12002	0.943401	1.2182	1.0406
-0.229469	0.301195	-0.0695362	-0.253825	-0.229252	0.067341	0.074856
-1.10703	-0.719322	0.263639	0.304619	0.157007	0.42824	0.35297
0.362098	-0.17472	0.00991067	-0.152471	-0.253741	0.36501	0.23586
0.236514	0.274251	0.0559469	-0.0325384	0.226254	-0.014755	0.3561
0.655559	1.42176	1.48751	1.85769	1.44818	0.34938	0.65448
2.61554	2.63742	0.131253	0.421816	0.117747	0.2717	-0.54952
0.729953	1.16947	0.900181	1.26594	1.22306	0.96192	0.84061
0.603331	0.227625	0.498221	0.425642	0.451027	0.12994	0.1023
0.215413	0.13151	0.0195138	0.0471282	0.00813037	0.74377	-0.0085761
-1.1703	-0.426554	1.68452	1.58526	2.12845	0.50952	-0.41627
-1.77958	-1.26977	-0.958919	-1.0346	-0.887942	-0.30708	-0.52348
-0.567846	-0.678165	0.047111	-0.123028	-0.041234	0.53844	0.21415
-1.03513	-0.580348	-0.354196	-0.0631574	-0.108701	0.8392	1.0009
5.71221	5.33786	4.47019	3.92462	4.27866	1.9888	1.6985
7.14377	7.41575	6.13794	5.32021	5.69119	2.4081	2.5359
0.0591515	0.450058	-0.0740896	-0.0003188	-0.0339176	0.82364	0.6486
0.672959	0.587629	0.235778	-0.0127011	0.144375	0.52367	-0.28174
-0.301996	-0.164926	0.792655	0.695881	0.836939	-0.15103	-0.53804
0.475757	0.242182	0.204521	0.0957786	0.111741	0.50014	0.60054
3.58964	3.42033	2.72085	2.67496	2.53039	0.48201	0.37236
-0.172388	0.484675	0.214886	0.520367	0.404197	0.36102	-0.41054
-0.131208	0.138505	-0.229901	-0.0899095	-0.179703	-0.19344	-0.34656
1.94578	1.97303	2.06158	2.17323	2.03083	0.21815	0.13165
1.12245	0.510321	1.43682	1.33823	1.47143	0.74935	0.58262
0.0931742	-0.220198	-0.267455	-0.328585	-0.476244	0.42927	0.81039
-0.32343	-0.289225	-0.738715	-0.690945	-0.686678	0.65998	0.74677

-0.22674	-0.259107	0.0286918	0.0687656	0.0277596	0.59582	0.83828
2.08629	1.77961	1.78504	1.90093	1.74425	0.98717	0.93656
1.10092	0.866308	1.16691	0.572718	0.707122	0.56131	-0.052205
-1.59404	-1.20168	0.071838	0.0376711	0.0667908	1.2295	1.2258
1.15163	1.69633	0.216345	0.191717	0.0313851	0.5221	0.313
-0.29648	0.196604	2.25528	2.31397	2.74804	1.4897	1.3706
-0.749591	-0.812006	-0.523195	-0.487381	-0.706563	0.32423	-0.3509
-0.171978	-0.136102	-0.41363	-0.402722	-0.31981	0.44385	0.3496
0.703622	1.24382	0.755779	0.800674	0.783114	0.53229	0.19521
1.22056	1.67798	1.53779	1.64904	1.68484	0.67193	0.52298
-0.122595	-0.296772	0.089013	0.0470018	0.0629197	0.00064263	-0.2015
-0.0820762	-0.0347798	-0.21231	-0.149343	-0.189048	1.0861	0.89866
-0.750401	-0.807312	0.276417	0.0755181	0.32601	0.060382	-0.2394
-1.19412	-1.15447	-0.279636	-0.49878	-0.298169	0.85736	-0.0076573
0.91261	1.2959	0.84378	0.75949	0.9463	NaN	NaN
1.52523	1.13797	0.377297	0.544016	0.423694	0.76956	0.90025
-0.640986	-0.62604	1.29898	1.21647	1.14677	1.435	0.32679
-1.12897	-0.384956	0.146566	0.22822	0.07425	0.3053	0.95766
-0.69171	-0.86808	0.40704	0.2525	0.19425	1.9987	1.9327
0.463816	0.339828	0.178251	0.072758	0.304838	1.2176	1.2141
-0.053086	-0.132881	-0.209715	-0.124575	-0.190748	0.36861	0.27535
-0.62479	-0.5766	0.37204	0.31497	0.20334	NaN	NaN
1.21356	1.62471	-0.0513631	0.0167668	-0.0444896	1.1317	0.89954
-0.974957	-0.835445	1.00815	0.909361	0.963861	0.81545	0.34773
1.18517	0.891193	0.976657	0.997125	0.99788	0.61786	0.2093
5.98022	6.10744	5.43558	5.72194	5.44308	0.88899	0.5054
5.92657	5.14601	4.98819	4.90063	4.81652	0.51861	0.74593
2.42554	2.00842	2.50682	2.33655	2.40017	1.0474	1.0383
0.01361	0.065792	-0.14733	-0.12058	-0.1542	1.6328	0.97168
0.018699	0.011065	0.058935	0.062476	0.055508	2.7689	2.1383
0.045686	-0.24032	-0.25786	-0.21143	-0.15154	3.182	3.0241
-0.09663	-0.0078558	-0.21393	-0.011488	-0.1079	2.9089	3.1136
-0.12765	0.027916	-0.036807	0.095142	0.071624	2.8973	2.7456
-0.069642	-0.022277	-0.16805	-0.20971	-0.13719	1.2258	0.79153
-0.073917	-0.031822	-0.0052498	0.077586	-0.061712	0.58665	-0.27834
-0.0946474	0.580631	-0.184659	-0.082089	-0.0697456	-0.1648	0.29421
-0.0971597	0.0332319	-0.23145	-0.0903462	-0.159204	0.58172	0.4124
-0.123197	-0.442482	-0.371821	-0.306828	-0.664244	-1.1469	-2.1859
1.88342	2.72951	1.06785	1.21295	1.25434	0.9985	0.64845
2.74955	3.5127	1.54864	2.03001	1.93205	0.71457	0.97703
0.688241	0.414293	-0.140799	-0.295051	-0.311723	1.5476	1.3111
0.489359	0.59367	1.15529	1.20606	1.31528	1.0122	1.3154

3.92342	3.2864	2.46671	2.09016	2.2787	-0.096598	0.039543
-0.639213	-1.13661	-0.90899	-0.841359	-0.942366	1.7057	1.7581
0.780018	0.431152	0.0365123	-0.0803763	-0.0091071	0.25777	-0.089684
0.0362614	0.06071	-0.290664	-0.201025	-0.2304	-6.96E-05	0.0012495
-0.596407	-0.486369	-0.292206	-0.309654	-0.383294	-0.92112	-1.7908
-0.666773	-0.0899303	0.290748	0.408687	0.484709	0.37891	0.38301
0.472919	0.579398	-0.0607345	0.122265	-0.0977017	-0.046817	0.46439
0.514455	1.08016	0.39036	0.535674	0.364472	1.6021	1.9012
0.493881	0.405937	0.792062	0.769286	0.953891	0.33192	0.69406
-0.673393	-0.827791	-0.405905	-0.275129	-0.43401	0.021993	-0.43816
-0.0350381	0.719132	-0.54847	-0.535128	-0.31944	NaN	NaN
0.142699	1.07272	0.251697	0.231773	0.15427	0.65692	0.57781
-0.809434	-0.864433	-0.897488	-0.822267	-0.896427	0.088342	-0.76355
-0.37316	-0.095943	-1.12662	-0.88958	-0.936191	-2.2442	-2.3233
0.552552	0.41339	-0.378046	-0.487858	-0.202519	-2.5244	-2.7105
1.58749	1.67219	1.19093	1.07728	1.17595	-0.23041	-0.44657
0.37031	0.0216768	-0.366835	-0.636271	-0.664307	0.94722	1.2367
0.0869818	0.0514094	-0.178001	-0.212778	-0.193693	-0.22057	0.47279
1.9614	1.95982	0.725774	0.517016	0.554856	1.0829	1.2181
-1.29172	-0.855256	-1.9282	-1.66081	-1.5335	0.28279	0.16361
1.23859	0.348934	-0.1223	-0.190353	-0.324405	0.2312	0.029226
3.58989	4.18559	1.62828	1.95477	1.50993	1.7106	1.9386
1.2467	1.9371	0.75159	0.89439	0.90139	NaN	NaN
2.74058	3.76337	1.94939	2.30941	2.12504	1.6314	1.5516
0.56126	0.93191	0.57815	0.53613	0.60908	-0.12776	-0.65423
-0.0010741	0.0481751	-0.181203	-0.067286	-0.15199	0.033615	-0.23366
1.14823	0.85821	0.946975	0.980721	0.877515	0.65969	1.4518
-0.714479	-0.739316	-0.711039	-0.361064	-0.407498	1.0252	1.3179
0.34899	0.491383	-0.129118	-0.0722011	-0.0083668	-0.17075	-0.63174
1.6691	2.736	0.22008	0.044873	-0.068828	0.35401	0.14993
0.342875	0.202794	-0.135406	-0.0248251	0.0633283	0.39316	0.61421
0.9083	0.601747	0.742618	0.643456	0.803253	-0.59147	-0.75723
-1.72416	-1.07744	-0.514053	-0.51582	-0.587159	-0.092687	-1.0212
0.266049	0.427865	-0.0594157	0.118747	-0.225129	0.32939	-0.057381
1.77035	1.12574	0.954772	0.666126	0.547349	1.8355	1.6752
-0.152081	-0.0950088	0.114202	0.0289603	0.0736955	1.1701	0.66997
-0.208986	0.342965	0.440834	0.278576	0.36747	0.627	0.079381
-0.192917	0.0187047	0.0807226	0.0481688	0.141909	-0.0019535	-0.73613
0.182033	0.165392	0.20945	0.0927715	0.0758191	0.16541	0.17982
1.77288	2.06991	2.24903	2.08871	2.15883	0.97773	1.468
-0.547128	-0.71587	0.221941	0.254621	0.143176	-0.077892	-0.14015
3.73494	3.3432	1.58194	1.08789	1.03017	-1.0004	-0.11097

0.323814	-0.0051168	-0.211221	-0.206271	-0.31161	0.1106	-0.23277
-0.827632	-0.560192	0.631636	0.70181	0.565525	0.97573	1.1285
0.436119	0.445168	0.515287	0.619898	0.513569	0.41435	0.65692
-0.722178	-0.70973	-0.437721	-0.625372	-0.65819	0.49824	0.48136
-0.224544	-0.235251	-0.0283407	0.106431	0.0310477	-0.40203	-0.41831
-0.0375336	0.151964	0.0947516	0.202585	0.170719	0.0025663	-0.066171
-1.77398	-1.57527	0.605895	0.585109	0.721267	0.087607	0.48501
2.01799	2.06272	2.01684	2.03561	2.07884	1.3028	1.2847
-1.38356	-1.09859	-0.531512	-0.312847	-0.130246	-0.27756	-0.6747
-0.398046	-0.486356	0.463916	0.141009	0.223223	-0.020351	-0.052085
-0.358829	-0.428251	-0.808086	-0.703944	-0.776021	0.19016	0.41056
0.342547	0.266224	0.355153	0.376559	0.423349	0.39333	1.0764
-1.23592	-0.891056	0.498767	0.39991	0.137672	0.43593	-0.24807
-0.0385032	0.0703648	-0.0147881	0.0313577	-0.0290446	-0.056995	-0.050156
-0.345866	-0.427849	0.0593594	-0.0016736	-0.152037	0.19055	-0.3202
1.33319	0.781639	1.74749	1.47686	1.78653	NaN	NaN
-1.01665	-1.14513	-1.80574	-1.76879	-2.02025	-0.27747	-0.17224
1.49342	1.37756	1.55345	1.69328	1.46368	-0.67822	-0.597
-0.137118	-0.481062	-0.558902	0.0140695	-0.0110181	-0.16105	-0.066688
-0.00116	0.0261028	-0.275827	-0.49025	-0.295277	-0.53295	-0.26251
-0.874382	-0.981133	-0.735783	-0.834348	-0.697321	0.12407	-0.56893
-0.641441	-0.641406	1.44409	1.39762	1.29042	0.40074	0.64016
-1.38346	-0.676609	0.378189	0.177669	0.29978	-0.063004	-0.2379
0.183951	0.295928	0.0740391	0.150257	0.311348	-0.19398	0.2779
-0.631271	-0.450727	-0.307629	-0.145752	-0.462928	-0.32056	-0.61496
1.18639	1.52099	1.25708	1.46186	1.54135	0.31405	0.52864
-2.92719	-1.61104	0.597962	0.857015	0.808951	1.0472	1.2963
-0.517506	-0.223907	-0.0071412	0.0742472	0.131204	-0.27858	-0.38895
2.50528	2.13734	1.3985	1.51252	1.50266	0.15988	0.57571
1.58891	1.72837	1.36832	1.27666	1.18432	0.54958	0.56194
0.254904	0.437245	0.045978	-0.178218	-0.0432567	0.10314	0.021921
-0.228469	-0.244011	0.0476541	-0.0472972	0.0965793	0.14581	0.72506
-0.26713	-0.292117	0.34091	0.287896	0.168672	-0.67898	-0.6674
-0.0484454	-0.0642621	0.0783455	-0.037292	0.00187901	-0.53482	-0.088782
1.39063	1.31507	1.44365	1.24504	1.36261	0.037828	0.35314
0.292606	-0.0453191	0.734634	0.610035	0.731683	-0.97623	-1.1739
0.8083	0.70301	0.056757	0.087766	0.085	0.058262	0.55605
0.311593	0.402084	0.105477	0.159479	0.236254	-0.13698	-0.27394
1.32288	1.93944	0.469624	0.603832	0.615514	0.017709	0.59813
0.51952	0.39282	1.0058	0.97901	0.86081	-0.30224	-0.045338
-1.12165	-0.88282	2.42611	2.71512	2.83116	3.3509	3.8992
-2.17512	-1.73584	2.91795	3.58392	3.14553	3.3909	3.6405

-0.321142	-0.467071	-0.420301	-0.370202	-0.491668	NaN	NaN
0.644233	0.893777	0.112288	0.172519	0.0285321	0.062256	0.073834
0.185742	-0.35638	2.33678	1.74037	1.78715	0.39865	0.8557
0.531644	0.643511	0.470966	0.1476	0.107614	-0.006117	-0.056376
0.804241	1.69965	0.899666	0.783178	0.653392	2.9148	3.6788
2.80628	3.42116	2.82102	3.04941	2.97159	0.44579	0.71598
-0.509419	-0.488509	0.0886215	-0.218627	-0.159254	-0.16232	-0.073913
1.81652	0.817572	0.510521	0.481399	0.471008	-0.67162	-0.17984
0.37381	0.0772944	0.250377	-0.0036409	0.316619	-0.39763	0.0489
0.66602	0.3863	1.0448	0.88319	0.88871	0.28374	0.39924
0.662778	0.388632	0.133794	0.0864555	0.0365649	0.4092	0.61705
1.29929	1.22945	1.35452	1.31086	1.37831	-0.30599	-0.88416
-0.274414	-0.248989	0.22488	0.550397	0.438672	1.0567	0.62186
0.310928	0.0883515	0.0205321	0.0632394	0.0116739	NaN	NaN
-0.444276	-1.35726	-0.800811	-0.924269	-1.15702	-0.1091	-0.3726
0.333093	0.0943685	0.143029	0.0702699	0.2723	-0.71015	0.35424
2.61597	2.79817	1.66521	1.81994	1.73602	NaN	NaN
0.0447396	0.278624	0.580634	0.460308	0.567048	-0.2243	-0.58721
-1.7932	-1.60641	0.195363	0.106625	0.293574	0.41675	0.62214
1.13227	1.70106	1.24096	1.40056	1.26861	-0.18938	-0.45
-1.91237	-1.37079	-1.37686	-1.19911	-1.3162	-1.289	-0.92444
-0.394168	-0.393592	-0.409979	-0.224342	-0.315423	-0.063972	-0.18559
0.318033	-0.784382	-0.631954	-0.678633	-1.01654	0.75244	0.92409
3.24685	2.85263	2.16032	1.92946	2.06709	-0.43505	-0.29035
0.556843	0.603678	0.649902	0.666201	0.777824	-1.0387	-0.97982
-0.400961	-0.598721	-0.0881246	0.0134279	0.161788	-0.019002	0.26227
1.00316	0.824497	0.388923	0.272639	0.459096	-0.36214	0.12754
1.57487	1.07298	0.309215	0.14122	0.205646	-0.78068	-0.24735
-2.47734	-1.75381	-0.59374	-0.28647	-0.500827	-0.70652	-0.11095
-1.62307	-1.8337	0.928826	0.866217	0.183876	0.78419	-0.54888
0.710899	0.578651	0.176916	0.411201	0.329375	-0.25764	0.23291
0.125238	-0.0785367	0.313259	0.152649	0.216629	-0.5159	-0.036396
0.901178	1.00178	1.55912	1.80547	1.65621	-0.72926	-0.55602
3.38082	4.11012	3.10449	3.50839	4.00997	1.8598	1.7559
-0.731837	-0.595449	-0.240385	-0.179513	-0.201025	-0.72591	0.12014
1.72804	2.67018	0.684077	0.663195	0.737016	-0.54315	-0.23476
-0.761483	-0.473112	1.25359	1.21406	1.16	-0.33377	0.033649
0.61862	0.746841	0.0932413	0.243601	0.188119	0.32273	0.99707
-1.83669	-1.51475	0.40257	0.109708	0.206912	-1.0926	-0.67779
-0.173915	-0.427269	-0.664537	-0.56651	-0.562824	-0.087505	0.086712
0.212444	-0.389469	0.764172	0.324964	0.578346	0.27807	0.78017
0.405943	0.240224	0.0336105	0.0396361	-0.0674135	-0.27006	-0.025305

0.0390923	0.0798799	0.18414	0.0814695	0.140516	-0.67332	0.15142
0.82115	0.71241	-0.17966	-0.022202	-0.16248	-0.28277	-0.50926
0.30503	-0.32664	0.561775	0.30064	0.525061	0.58938	0.84205
2.02273	0.240613	1.23561	1.04447	1.1439	-0.60524	-0.62061
0.416572	0.320529	0.4481	0.671465	0.403344	0.0007302	0.042537
0.100668	0.492233	-0.0732134	0.003934	0.12813	-0.57691	-0.77278
0.733089	1.88298	0.616	0.375425	0.475771	0.27432	0.14812
0.73648	0.62679	0.8549	0.87082	0.96548	0.021695	-0.96411
1.62637	0.590945	-0.10052	-0.166426	-0.198971	0.38738	0.78111
-1.5946	-0.64228	-1.8546	-1.425	-1.7507	-0.79869	-0.34542
-1.3685	-0.99454	-0.942371	-0.788801	-0.818921	-1.3341	-0.36112
-0.44121	-0.0809897	-0.507829	-0.207727	-0.327447	-0.56065	-0.24765
-1.6179	-1.15826	0.0333765	-0.052211	0.0934493	0.27893	0.4997
-0.241328	0.147564	0.0741067	0.0294656	0.146063	-0.3909	-0.438
0.0666561	-0.0499133	-0.0309772	-0.198899	-0.010747	-0.69956	-0.35316
0.32052	0.245468	0.118402	0.0575184	0.403883	-0.70315	0.051543
0.173196	0.374989	0.0442245	-0.108167	0.0753425	-0.40984	-0.48757
0.358215	0.398462	0.204206	0.0204855	0.0468953	-1.1667	-0.65329
-0.475689	0.0485438	-0.735714	-0.321403	-0.358613	NaN	NaN
0.170066	0.0621432	0.482484	0.200507	0.389075	-0.66285	-0.69904
-0.228131	-0.385783	-0.0383484	-0.0212118	0.134163	-0.32025	-0.11332
0.579847	0.543384	0.562077	0.541086	0.57255	0.34898	0.0074697
-1.7679	-1.3997	-0.68912	-0.67787	-0.90602	0.15774	0.69831
-1.29709	-1.19384	-0.553816	-0.424769	-0.46456	0.23262	0.30324
-0.145498	-0.0599148	-0.30946	-0.287115	-0.191637	NaN	NaN
1.35834	0.839656	1.42124	1.25762	1.2437	0.32683	1.1463
1.18301	1.65413	1.66447	1.69988	1.83554	1.4655	1.2669
-0.61477	-0.66979	1.0692	0.88811	0.91611	NaN	NaN
-0.342642	-0.0577511	1.93467	1.74601	1.7921	0.55972	-0.5902
2.93092	2.5094	2.02577	1.74948	1.90673	0.90476	1.2303
-0.201527	-0.122078	-0.229687	-0.300245	-0.0143682	-0.60297	-0.90321
0.167081	0.0662933	0.166927	-0.0055851	0.0945941	-0.24218	-0.1554
-2.9362	-2.2576	-0.78691	-0.80039	-1.1252	-2.7258	-2.8544
1.25869	0.991389	2.00678	1.49755	1.6001	-0.74483	-0.11848
0.701	1.11934	0.0705945	0.279115	0.245615	-0.53631	-0.78221
-0.361186	-0.141259	-0.440835	-0.503574	-0.501894	-0.81966	-0.88743
1.83159	1.15883	0.447389	0.421309	0.395181	0.31778	0.35232
-0.073335	1.28387	0.56467	0.919999	0.673793	-1.2306	-1.5608
2.30926	2.34473	1.67546	1.73066	1.70465	1.8241	2.4612
0.367883	0.286775	0.00510413	-0.0199369	-0.0501395	0.018428	0.08958
2.72567	2.35283	3.00303	3.2824	3.12859	-0.070736	0.49316
4.0245	4.66823	2.75071	3.19489	3.09581	0.084309	-0.0005877

0.221483	0.118008	-0.0414219	0.141595	0.0474009	0.36529	0.61038
-0.127888	0.520897	-0.271375	-0.596417	-0.442109	-0.61625	-0.1951
-0.952823	-0.83919	0.468594	0.215704	0.402102	2.1249	2.4937
-0.581532	-0.690178	-0.764644	-0.966896	-0.77048	-0.19684	0.23574
-0.0093866	-0.0404936	-0.316776	-0.44449	-0.306059	-0.61123	-0.47037
-1.91915	-1.48218	0.864059	0.88529	0.908896	0.26411	0.66778
-0.39917	-0.408371	0.139133	0.347648	0.235382	-0.021918	0.99922
-0.398509	-0.353388	0.392583	0.299891	0.409628	-0.63236	-0.27279
1.3764	1.2469	1.45581	1.29698	1.39822	-0.13664	0.14386
0.57505	0.20802	1.3574	1.6871	1.3342	-0.15901	-0.10105
-1.08897	-0.722732	-0.449242	-0.385444	-0.349009	0.28678	0.064969
-0.191478	-0.201871	0.860973	0.763769	0.821681	-0.56439	0.24813
0.708437	0.34995	0.164323	0.123147	0.118563	0.078416	0.59976
1.1483	0.84694	0.23744	0.16762	0.26102	0.18347	0.49082
-0.0008607	0.0596983	-0.0625268	-0.106262	-0.0210237	0.40612	0.33195
-0.147167	-0.125343	-0.0584724	0.0617081	-0.104259	-0.3805	-0.53556
-0.384497	0.0186677	-0.333083	-0.238256	-0.310281	0.60036	0.37898
0.807498	1.15133	1.23049	1.37847	1.39469	-0.50303	-0.067633
-0.291816	-0.151078	-0.849147	-0.855562	-0.9178	0.44312	0.8034
1.91427	2.56442	1.28364	1.19582	1.29634	0.51098	0.33222

test_E-MEXP	test_E-MEXP	test_GSE7_tr	test_GSE7_1	test_GSE7_1	test_GSE7_1	test_GSE7_1
ref_E-MEXP-	ref_E-MEXP-	ref_GSE7_tn	ref_GSE7_0n	ref_GSE7_0n	ref_GSE7_0n	ref_GSE7_0n
E-MEXP-137(	E-MEXP-137(	GSE7	GSE7	GSE7	GSE7	GSE7
ArrayExpress	ArrayExpress	GEO	GEO	GEO	GEO	GEO
affymetrix	affymetrix	cDNA	cDNA	cDNA	cDNA	cDNA
relA_b2784:-	ISOLEUCINE:	trpR_b4393:-	INDOLE_ACR	INDOLE_ACR	INDOLE_ACR	INDOLE_ACR
140	142	165	173	177	178	179
0.37524	0.073708	0.062633	0.0080113	-0.23608	0.1222	0.12578
-2.1587	-1.0381	-0.47283	-0.72915	-0.45574	-0.6688	-1.0371
0.61643	0.26098	0.6373	0.48718	1.3065	1.3446	1.1014
-0.18268	-0.37418	0.054119	-0.11801	-0.055373	-0.161	0.047287
0.14384	0.18398	1.102	0.028747	0.58339	0.41606	0.81213
-0.093647	-0.075555	0.79096	0.38433	0.27884	0.37111	-0.093759
-1.4267	-1.4066	-0.18815	-0.070134	-0.51712	-0.74135	-0.40813
-0.54146	1.1271	-0.23878	0.57401	0.76935	0.94205	0.7735
0.15973	0.23313	1.0373	-0.12953	-0.13374	-0.098888	-0.096364
0.27007	0.48763	0.16054	0.38112	0.75939	0.68716	0.85925
0.9943	0.40234	-0.038879	0.024005	-0.27672	-0.19758	-0.28857
1.1068	-0.090738	-0.25305	0.96841	1.1671	0.833	1.0805
0.03308	0.18197	0.018031	-0.0027025	0.027187	0.06942	0.15584
1.0347	-0.42413	-0.21319	-0.36441	-0.23873	-0.030533	-0.28336
NaN	NaN	7.13E-05	-0.5809	-0.44738	-0.40302	-0.36911
-0.2401	-0.29601	-0.045916	0.14376	0.19043	-0.06105	-0.29069
1.7286	-0.11068	-0.048875	0.61443	0.7127	0.81171	1.3884
1.39	-1.0516	0.11903	0.37403	0.35237	0.81988	1.4354
0.27908	0.1961	0.50274	0.65067	0.70888	0.66162	1.1712
-0.37834	-0.16338	-0.15151	0.15045	0.12504	-0.10164	0.20175
0.37213	0.084101	0.40677	-0.28129	-0.18142	-0.21916	0.062
-0.040981	0.077909	-0.033248	-0.08181	0.059033	0.11556	0.2819
-0.1183	0.52602	-0.036914	0.86112	1.2894	0.9963	1.2907
NaN	NaN	-0.1857	0.17581	0.61043	0.45609	0.88596
0.74419	-0.2361	-0.030391	0.312	0.92623	0.43817	1.3818
-0.029085	0.21377	-0.3085	-0.24454	0.58674	-0.16935	-0.086794
0.87091	2.9313	-0.44233	-0.19061	0.097449	0.32669	0.34709
-0.44588	0.32847	0.16244	-0.095199	-0.11989	-0.030449	-0.11311
0.33543	-0.34021	0.042174	0.62105	0.67174	0.56121	0.72433
0.90108	1.1118	0.081975	0.7498	1.2917	1.3402	1.4078
1.1645	-0.2603	0.066078	0.2709	0.24699	0.41723	0.092824
-0.17633	0.98354	-0.37557	0.12791	0.36033	0.76772	0.47079

0.26568	1.2511	-0.10496	-0.4872	-0.2542	-0.27206	-0.27248
-0.13097	-0.13314	-0.018846	-0.04487	-0.087128	-0.12909	-0.062104
0.39924	0.67156	0.063499	0.79402	1.0627	1.2335	1.243
-0.11194	0.18904	-0.058807	-0.51686	-0.3382	-0.1974	-0.23959
0.28946	0.29599	0.44851	0.076207	0.12302	0.22092	0.65276
0.9943	1.6307	0.4272	0.53429	0.60841	0.86745	1.4124
-0.35344	0.70459	0.26214	0.16815	0.32096	0.58814	0.87189
-0.086587	-0.0019697	0.30757	0.17056	0.26937	0.23636	-0.050321
0.11691	-0.027254	0.13169	-0.027212	0.078612	0.053458	0.36858
-0.022533	0.094054	-0.12324	0.75753	0.74928	0.55455	0.99065
0.61232	0.58316	0.25587	-0.27856	-0.28663	-0.11135	0.020684
-0.73244	-0.35229	-0.017783	0.01817	-0.36089	-0.29202	-0.16542
0.25253	0.07966	-0.019833	0.16468	0.093872	0.23518	0.046856
-1.621	-0.55779	0.56676	-0.094267	-0.17055	-0.16362	0.31182
0.054523	0.55254	-0.29338	0.53149	0.46496	0.33342	-0.066405
0.88626	-0.69283	-0.17016	0.38298	0.65769	0.66589	0.39216
0.045389	-0.055965	-0.090663	0.34748	0.057806	-0.042628	0.16538
0.34668	-0.189	0.030527	0.24017	0.66301	0.76645	0.83859
0.38793	-0.22803	0.21407	-0.184	-0.055775	0.06091	0.22428
0.16408	-0.085927	0.20831	0.035607	0.31164	0.29905	0.46015
0.24175	0.33645	-0.10428	-0.53567	-0.40594	-0.17181	-0.23423
0.20093	0.89589	-0.20637	-0.20763	-0.16823	-0.11571	-0.3892
0.50111	0.0126	0.00093921	-0.20948	-0.14076	-0.13354	0.13193
0.028166	0.50686	-0.042611	-0.063911	0.24622	0.3467	0.59362
0.84816	0.25334	0.077687	0.29777	-0.025923	-0.13457	0.33118
0.84652	3.3599	0.5958	0.35288	0.25821	0.23863	0.016894
0.18542	0.47991	-0.14282	0.15164	0.0017989	0.077086	0.13588
0.66851	0.40512	0.38917	0.15088	0.30571	0.51732	1.1817
0.81828	2.168	-0.28805	-0.34878	-0.33535	-0.16703	-0.50923
1.9499	0.11881	-0.14577	0.46429	-0.063246	-0.18817	0.17268
2.7438	-0.035199	0.13614	0.41836	0.29245	0.35541	0.55909
0.7149	0.077279	0.068431	0.13027	0.33781	0.57774	0.8533
0.37439	0.59194	-0.23142	0.40838	0.5426	0.40895	0.5511
0.20055	1.2902	-0.14607	-0.47686	-0.34692	-0.60233	-0.61339
0.45404	1.1871	-0.128	0.90488	0.93221	1.189	2.008
0.29183	0.097663	0.16254	-0.44635	0.1596	0.20708	0.16123
0.28737	0.70175	0.42083	0.32178	0.3416	0.37421	0.64282
-0.57061	0.26714	0.4918	0.076207	0.14451	-0.020671	-0.074016
0.11024	0.75239	0.43878	0.17711	-0.1394	0.27821	0.73523
0.8432	0.28047	-0.26851	0.29147	0.12258	0.15912	-0.05016
0.79466	0.42488	0.19611	0.37795	0.53137	0.56469	1.2353
0.72379	1.4312	-0.048285	-0.2641	-0.69015	-0.61214	-0.40578

0.56287	1.3254	-0.33177	0.35912	0.81351	1.2887	1.369
1.0644	0.79621	0.46027	0.41667	0.23258	0.015653	-0.0056408
0.87975	0.38098	0.44508	0.73907	1.2546	1.2899	1.2658
1.2786	0.38994	0.41655	0.41286	0.31294	0.034736	-0.33901
0.6011	0.093362	0.2324	-0.61415	-0.13764	0.07791	0.35739
1.47	1.1065	-0.18679	-0.098002	0.1013	0.3635	0.47211
0.25596	-0.20093	0.14491	0.11962	-0.042319	-0.24572	-0.36534
0.4831	0.67138	0.80411	0.15131	0.23568	0.28833	0.57853
0.41635	0.2356	0.17331	0.18365	0.45396	0.62268	0.69499
0.53522	0.221	0.41077	0.54426	0.55459	0.42073	0.44128
0.13472	0.26485	0.50555	0.14691	0.029086	0.037951	-0.39959
0.26474	0.29679	0.23205	0.14468	0.20004	0.33905	0.48291
-0.075894	0.26381	0.80251	0.85264	1.0333	1.13	0.47737
0.74538	0.94917	0.071814	-0.30999	0.10962	0.13313	0.3174
NaN	NaN	0.98316	0.6795	0.66527	0.61695	0.036795
0.62407	0.22769	-0.043089	-0.32501	-0.27099	-0.15587	-0.14687
1.2074	0.87189	0.26334	0.069212	0.026345	0.21777	0.045781
0.36408	-0.10474	-0.40923	0.019437	0.14847	0.3136	0.41136
1.3208	0.54068	-0.27152	-0.58129	-1.0504	-0.90999	-0.85017
1.364	0.55867	-0.30567	-0.30555	0.14902	0.011564	-0.25085
0.008166	0.19715	0.52315	0.23334	0.33716	0.50227	1.1635
NaN	NaN	0.047311	0.51185	0.5904	0.50193	1.2181
0.8838	0.03191	0.049892	0.42176	0.40644	0.40103	1.284
0.91909	0.23075	0.32625	-0.12393	-0.14335	-0.046005	0.18185
0.51304	0.15514	-0.2547	-0.30096	-0.12846	0.0074523	0.014394
0.72727	-0.46975	0.21698	2.5673	3.0453	2.4274	1.9289
0.075996	-0.48927	-0.058765	0.7304	1.3491	1.1563	0.79358
0.88354	-0.085091	0.033524	0.57766	0.41039	0.59906	0.70548
1.2183	0.745	-0.01227	-0.15475	-0.16575	-0.23851	0.10873
2.7542	0.3684	-0.58305	-0.1346	-0.38652	0.0027744	-0.034188
2.7633	-0.24549	0.10641	1.1592	0.50859	0.46843	0.89274
2.9744	2.2278	0.22021	0.19274	0.29351	0.38685	0.265
2.9651	1.5846	0.081491	-0.09425	-0.050794	0.29557	-0.29438
0.94335	0.28332	-0.17792	-0.11913	-0.21411	-0.10805	-0.080046
0.29278	0.79751	0.039001	0.55055	0.6008	0.55978	0.52923
-0.050616	0.031677	0.91288	0.65983	0.53487	0.67824	-0.0053548
0.51885	0.12397	0.21816	-0.15008	-0.1518	-0.11992	-0.21455
-0.93288	-0.19688	-0.29011	0.50141	0.11038	0.47522	0.79097
0.89259	0.42665	0.40035	-0.17962	-0.13334	-0.11114	-0.2035
0.73733	0.34544	-0.0051829	-0.53907	-0.44454	-0.40935	-0.19704
1.4179	0.082747	0.032927	-0.058609	0.25558	0.25873	0.47916
1.1943	1.7094	0.22483	-0.057635	0.011515	0.30326	0.36818

-0.13805	-0.085557	-0.086085	1.0482	1.5367	1.6769	1.1094
1.6924	-1.22	-0.076594	0.24911	0.43634	0.59418	0.75988
0.12812	0.50506	0.34101	-0.062947	-0.067671	-0.22369	0.40032
-9.99E-05	-0.0001385	0.29815	-0.54994	-0.42844	-0.44206	-0.23178
-0.92287	0.84394	0.31871	1.115	1.5915	1.2536	1.8014
0.27642	0.81985	-0.29944	-0.0090726	-0.10581	-0.41237	-0.26672
-0.15756	0.10373	0.15822	-0.049487	-0.080619	0.092951	0.20448
1.1389	0.074143	0.1424	0.12077	0.60231	0.53726	0.51511
0.20041	0.34166	0.43401	0.4418	0.87545	0.76452	1.2256
0.18993	0.66224	0.44808	0.38759	0.2866	0.66449	-0.028452
NaN	NaN	0.0099472	-0.040671	-0.18181	-0.085132	-0.057002
0.31728	0.36794	-1.1679	0.16393	0.2473	0.45781	0.67339
0.2415	-0.13657	0.31609	-0.29026	-0.31295	-0.20324	-0.74411
-1.9981	-1.2145	0.45068	-0.531	-0.55209	-0.19002	-1.5899
-2.297	-1.7867	0.80794	-0.054615	-0.013853	0.23438	-0.99213
-0.5575	0.5914	0.074512	-0.30381	-0.28259	-0.14368	-0.39905
0.81822	1.0211	0.026278	-0.43166	-0.50549	-0.45616	-1.0204
-0.5842	-0.55066	0.19991	-0.032887	-0.020613	0.10674	0.13852
0.97328	0.53366	1.2121	0.088451	0.53059	0.91502	1.6067
0.75338	1.5214	-0.2668	0.42865	0.19616	-0.053199	-0.17699
0.11716	0.10202	-0.15411	0.31453	0.11139	-0.72711	-1.2752
1.5875	1.2767	0.73944	0.044818	-0.082972	-0.18147	0.2342
NaN	NaN	NaN	NaN	NaN	NaN	NaN
1.7114	1.6766	0.13046	-0.40205	-0.093712	0.010938	-0.18832
0.25268	0.74325	0.23238	-0.14394	0.37801	0.26898	0.45711
-1.86E-05	-0.43669	0.25704	-0.24488	-0.024522	0.099806	-0.076861
0.38199	0.20112	0.2749	-0.44273	-0.41876	-0.30259	-0.32269
0.66866	0.098588	-0.091678	0.045982	-0.064752	0.19418	-0.093357
-0.072465	-0.1196	0.65156	-0.064944	0.022148	0.053228	-0.0014187
0.27549	0.78415	-0.52161	0.43725	0.48759	0.47709	0.5209
0.37686	0.12061	0.59532	0.70173	0.51942	0.54766	0.15477
-0.4656	0.24596	0.029149	-0.25151	0.66621	0.56636	0.47198
0.036252	-0.49551	-0.095106	-0.49237	-0.85857	-0.70559	-0.84622
0.21347	0.36849	0.074419	-0.42314	-0.46262	-0.58554	-0.62246
1.8358	1.1045	-0.53659	-0.92945	-1.0667	-0.96907	-1.3139
1.0577	-0.23824	0.29805	0.051279	0.0057681	0.029804	-0.12157
0.505	0.64024	0.30017	-0.30663	-0.19652	-0.50594	-0.48423
-0.23435	-0.1135	0.41561	0.54365	0.3892	0.23732	0.48543
0.019	-0.037546	0.27805	0.22137	0.34602	0.4025	0.10333
0.95381	0.34402	0.2852	0.50947	0.62757	0.52117	0.54596
0.033313	0.32253	0.085068	0.016162	0.004311	0.018611	0.48065
-0.99677	-0.22527	0.80486	0.76041	0.77655	0.95636	0.8028

0.19308	0.14494	0.44318	0.065468	0.10098	0.047201	0.20685
1.091	0.42805	-0.11772	0.042778	0.24281	0.29019	0.24458
0.41375	0.14203	-0.16196	0.052109	0.13451	0.099187	0.47776
0.52537	-0.15066	0.2163	-0.27934	0.33657	-0.10392	0.081671
-0.12948	0.16818	0.33962	0.096081	0.064083	0.096099	0.68014
-0.23433	-0.025518	-0.015967	0.0068346	-0.060332	-0.13223	0.085235
0.35693	0.42039	0.088113	0.33342	0.96955	1.0857	0.92831
1.6071	0.5135	0.18966	1.4513	1.0737	0.9172	1.7734
0.14729	0.44664	1.2272	0.38633	0.48722	0.88385	-0.27426
0.23015	0.40297	-0.030832	-0.42125	-0.15937	-0.056843	0.030904
0.18609	-0.17284	-0.35879	-0.21622	-0.53349	-0.54625	-0.19036
0.13377	0.41758	0.1276	0.014443	0.17653	0.26547	0.28922
0.47016	0.17182	0.21524	-0.022819	-0.052814	0.078035	0.021488
-0.25028	-0.016472	0.71974	-0.070368	0.073221	0.026339	-0.21718
0.2856	0.20987	-0.13793	0.28282	0.46281	0.44135	0.60724
NaN	NaN	-0.13971	-0.069849	-0.20419	-0.17323	-0.37306
-0.5273	-1.1968	-1.5143	0.15222	0.31887	0.36792	0.61078
-0.75256	-0.83134	-0.18464	1.8143	1.74	2.0741	1.8876
-0.25355	-1.0459	0.1507	0.2693	0.11407	0.22313	0.080709
-0.48617	-0.38151	-0.24584	-0.30196	-0.33636	-0.34705	-0.4115
0.079725	-0.2445	0.056107	-0.68221	-0.52747	-0.26621	-0.8561
0.60316	0.065927	0.10279	0.28866	0.18063	0.078427	0.21662
-0.031794	0.40351	0.10001	0.082559	0.21897	0.3738	0.27056
-0.060546	-0.097818	-0.18354	-0.071693	-0.24711	-0.25658	-0.32242
-0.28031	0.33551	-0.27377	0.25891	0.43816	0.40121	0.774
0.24179	0.2019	0.11691	0.27296	0.74263	0.50131	0.64969
1.13	0.63716	0.22016	0.84879	0.18572	0.014537	0.24386
0.11417	0.86239	0.48844	0.25295	0.43151	0.63655	0.31838
0.086185	0.2003	-0.073754	0.30233	0.59266	0.429	0.48543
0.67332	0.3073	0.061208	0.29635	0.52151	0.64265	0.72986
0.14371	-0.12795	-0.029623	-0.16113	-0.0041018	0.10422	-0.022834
0.031619	-0.0070281	0.1217	-0.017038	0.029867	0.020987	0.0033458
-0.64429	-0.34271	0.10444	0.33097	0.18017	0.21946	0.26795
-0.54157	-0.44195	0.071356	0.012355	-0.32157	-0.1181	-0.16136
0.034249	-0.1444	0.97594	0.10043	0.27962	0.43349	0.22897
-0.51484	0.93561	-0.16981	0.42993	1.1667	1.0271	0.79754
-0.14056	-0.48258	0.18138	0.4701	0.28896	0.40137	0.66866
-0.28046	-0.37372	0.22194	0.28019	0.77556	0.66252	0.70502
-0.10731	0.28823	0.41955	0.83225	0.6836	0.35243	0.0091153
-0.053989	-0.011533	0.31691	-0.73112	-0.014769	0.15393	0.31899
3.3393	1.7822	0.59847	0.14916	0.2534	0.070744	0.19652
3.2905	1.2639	0.35036	-0.21884	0.012785	-0.080125	-0.12494

NaN	NaN	-0.090773	0.45311	0.66142	0.35214	0.62302
0.078279	0.22489	-0.27434	-0.13971	-0.078171	0.064147	-0.01952
0.41228	-0.50105	0.44345	0.21742	0.60696	0.070744	0.27629
0.041151	-0.089667	-0.080021	-0.30526	-0.15885	-0.32845	-0.26397
2.7986	-0.12793	0.27181	0.021348	0.39016	0.33974	0.11932
0.51158	0.81414	0.1694	0.62882	1.284	1.123	1.3607
0.13764	-0.24796	-0.10517	0.085847	0.41693	0.28763	0.021428
-0.56733	-0.3334	0.17778	-0.73003	-0.76997	-0.42112	-0.33701
-0.38409	-0.15694	0.081842	-0.0074342	-0.30745	-0.14137	-0.22748
0.47358	0.83471	0.37926	0.25264	0.28997	0.33572	0.2263
0.27659	0.10402	0.18901	0.4516	0.55683	0.54205	1.1115
-0.30276	-0.10012	-0.74873	0.40403	0.89512	1.1647	0.92771
0.82269	0.026463	0.036236	-0.17615	0.041583	0.10189	0.16675
NaN	NaN	0.3533	0.63122	0.94303	0.93172	1.4571
-0.65312	-2.4603	-0.6907	-0.75904	-1.3011	-0.80476	-2.3574
-0.34373	0.19034	0.11246	0.28498	-0.0062845	-0.097714	-0.31736
NaN	NaN	0.14845	0.28579	0.12747	0.54496	0.12239
0.091144	1.5517	-0.092497	-0.49413	-0.48341	-0.4736	-0.27995
0.36173	0.038416	-0.52016	0.012487	0.52877	0.42681	0.44153
-0.23328	0.62087	-0.10258	-0.26918	0.25127	0.158	0.0072969
-1.2207	-0.32325	1.7696	0.14579	0.054362	0.56778	-1.1195
0.024647	0.13129	-0.11359	0.07859	-0.12302	-0.48679	-0.059419
0.74246	-0.22576	-0.035714	0.31471	0.23702	0.21454	0.30614
-0.46303	0.10062	0.28361	-0.21162	-0.19617	-0.27401	-0.24195
-0.82517	-0.27221	-0.35596	-0.41554	-0.46933	-0.55352	-0.79499
-0.0007823	-0.40926	-0.2837	-0.083232	0.068149	0.1876	0.098725
-0.52917	-0.32317	-0.56141	-0.4002	-0.31091	-0.43731	-0.40019
-0.75692	1.5578	-0.76531	-1.1102	-0.51361	-0.44046	-0.90506
-1.0157	0.18673	-0.48095	0.21802	0.45224	0.53367	0.78772
0.53798	1.2073	-0.32558	-0.064944	-0.54743	-0.38784	-0.46812
-0.25443	-0.029318	-0.26044	-0.078198	1.0254	0.15315	0.10286
0.038555	-0.55596	0.29325	0.093058	0.34507	0.28392	0.25704
-0.60293	3.4282	-0.45944	0.052567	0.14072	0.10814	0.09331
2.7432	-0.74456	-1.1562	0.084439	0.10002	0.17371	-0.15913
-0.47854	-0.0001996	0.19953	0.36584	0.6228	0.36462	0.63473
-0.42674	0.04569	-0.17008	-0.28074	-0.19492	-0.14004	-0.1613
-0.3759	-0.4404	0.44261	0.26609	0.27518	0.255	0.11143
0.10351	-0.90178	0.09989	0.37174	0.26071	0.16989	0.13758
-0.96448	-0.062318	0.31922	-0.095318	-0.41714	-0.58918	-0.71726
-0.002014	-0.27989	-0.28148	0.32125	0.99665	0.64239	0.74472
0.43049	-0.2585	-0.24129	-0.27475	-0.065054	-0.31787	-0.33219
-0.24338	-0.0011767	0.19784	0.0475	0.035991	0.05611	0.038121

-0.51952	-0.21781	0.57557	0.28149	0.33033	0.27697	0.47798
-0.077952	-0.52475	0.1183	0.52963	0.54496	0.55265	0.026915
0.87534	-0.11067	0.13211	-0.44497	-0.83793	-0.59503	-0.7069
-0.62998	-0.24003	-0.34466	-0.087163	0.053766	0.05907	-0.15368
-0.10564	-0.22454	0.13832	-0.43049	-0.40707	-0.45951	-0.46577
-0.44935	-0.55033	0.43056	-0.48247	-0.49506	-0.47414	-0.35398
0.024393	-0.2241	-0.1299	-0.21378	-0.19262	-0.19865	-0.30155
0.24656	-0.047939	-0.22431	0.44499	0.56089	0.47443	0.53882
0.62336	-0.38493	-0.40452	-0.2966	-0.17015	-0.39562	-0.39894
-0.88591	-0.18868	-0.081568	0.16772	0.80968	0.45324	0.85498
-1.3452	-1.3831	-0.62494	-1.2573	-1.3533	-1.5061	-1.045
-0.59295	-0.52595	-0.25745	0.23439	-0.020716	-0.14098	-1.0535
0.22605	-0.031337	-0.37554	-0.15297	-0.13204	-0.2406	-0.42335
-0.2721	-0.012191	0.10933	0.15848	0.066667	0.11499	0.14068
-0.56985	-0.0094114	-0.10754	-0.22482	-0.15985	-0.18763	-0.10491
-0.74648	-0.44671	-0.10182	-0.020421	-0.015009	-0.1415	-0.10102
-0.2018	0.28851	-0.077659	0.50449	0.66876	0.52679	0.43343
-0.68351	-0.70517	0.7139	0.35268	0.52035	0.54313	-0.47919
NaN	NaN	-0.31296	-0.35047	-0.48238	-0.23088	-0.49091
-0.27157	0.16886	-0.0088704	-0.20682	-0.06972	-0.12897	-0.053363
-0.01938	0.29777	0.23144	-0.36907	-0.6007	-0.77788	-0.46474
0.39127	-0.16978	0.17151	0.031962	0.10367	0.24023	0.010685
-0.30539	-0.35685	-0.35597	-0.012117	-0.72075	-0.59379	-0.65105
0.71759	0.6259	-0.85506	-0.67703	-0.695	-1.0253	-1.2831
NaN	NaN	-0.20662	-0.3058	-0.26146	-0.34064	-0.25687
0.0022549	-0.72848	0.21033	1.2031	0.96345	1.1595	1.2547
1.3248	0.23615	-0.090679	-0.37263	-0.091303	0.11106	0.042823
NaN	NaN	0.41539	0.032285	0.15144	0.22997	0.049189
0.55383	-0.43736	0.52448	1.6106	1.0958	0.93493	1.1107
0.70805	-0.40921	-0.061968	-0.087782	0.2395	0.13614	0.24612
-0.51343	-0.28575	-0.064523	-0.24957	-0.11242	-0.35911	-0.24861
-0.29996	-0.34228	0.13847	0.1326	0.060477	-0.11789	-0.1232
-2.419	-0.87403	0.10228	0.002395	0.0051865	0.12067	0.081158
-0.55974	-0.31364	-0.27063	-0.37213	-0.53206	-0.28737	-0.41779
-0.027907	0.10594	0.63956	1.0825	1.1816	1.2606	0.88594
-0.70387	-0.77678	0.78425	0.44239	0.39634	0.66948	0.32296
-0.14138	-0.30545	-0.4296	-0.63128	-0.7776	-0.39483	-0.62164
-1.1084	1.1327	-0.18041	0.41787	0.21989	0.19503	0.050515
1.8977	0.49006	-0.11593	-0.08357	-0.079036	-0.023429	-0.10968
-0.096081	0.55052	0.16726	0.60036	0.26304	0.39549	0.26922
0.022553	0.29301	-0.17466	0.12994	0.50073	0.23114	0.40812
0.066519	-0.067425	0.27788	0.24869	0.17756	0.33857	0.54906

0.56336	1.1057	-0.086698	0.17642	0.023828	0.32079	0.56234
-0.76073	-0.28069	0.24896	0.051961	-0.044944	0.12484	-0.2321
0.23568	-0.60755	-0.15976	0.090919	0.029108	0.07715	-0.26982
0.18196	0.047083	0.16423	0.41798	1.5304	1.4637	1.4944
-0.4891	-0.40449	0.1968	0.2358	0.28359	0.40528	0.20799
0.095249	0.42674	0.021669	0.10069	0.73975	0.249	0.32657
0.17492	0.10185	0.57917	0.27845	0.24597	0.43777	0.40491
-0.5383	-0.092381	0.18901	0.25163	0.0018446	0.14329	0.43718
0.011203	-0.22522	0.49756	0.51728	1.2527	1.3711	1.1568
-0.10584	-0.49507	0.03754	-0.15265	-0.069539	-0.090549	-0.14032
0.60845	0.014313	-0.28473	-0.17755	0.15417	0.23258	0.5533
-0.56721	-0.17952	1.1951	-0.039801	0.25547	0.045001	-0.21301
-0.033552	-0.093979	0.32982	-0.35644	-0.45418	-0.48054	-0.48969
0.14573	-0.0073241	0.79801	0.36188	-0.014815	-0.11629	-0.17023
0.52361	-0.35488	0.37653	0.43943	0.6712	1.0431	1.3137
-0.29553	-0.36824	-0.35564	0.65504	0.34985	0.61799	0.52547
0.40202	-0.10413	0.095528	0.63912	0.68786	0.6842	1.0472
-0.58185	-0.56301	0.61753	0.32327	0.79751	0.75129	0.9263
0.5943	-0.0099229	0.18171	0.16523	0.79692	0.84046	1.1392
0.60333	1.1982	-0.11213	0.073886	0.15214	0.28744	0.25645

test_GSE7_1	test_GSE9_U	test_GSE9_U	test_GSE9_U	test_GSE9_le	test_GSE9_le	test_GSE9_le
ref_GSE7_0n	ref_GSE9_no	ref_GSE9_no	ref_GSE9_no	ref_GSE9_no	ref_GSE9_no	ref_GSE9_no
GSE7	GSE9	GSE9	GSE9	GSE9	GSE9	GSE9
GEO	GEO	GEO	GEO	GEO	GEO	GEO
cDNA	cDNA	cDNA	cDNA	cDNA	cDNA	cDNA
INDOLE_ACR	TIME:10,UV::	TIME:20,UV::	TIME:60,UV::	TIME:10,UV::	TIME:20,UV::	TIME:60,UV::
180	183	184	188	193	194	196
0.30537	0.44812	-0.02399	-0.067225	-0.35264	-0.45672	-0.47489
-0.73181	-0.13071	0.2436	-0.032283	0.13423	0.11428	0.21721
1.8233	-0.40599	-0.32426	-0.11652	-0.016873	-0.19618	-0.029976
-0.025	-0.057831	0.057108	0.088032	-0.083777	0.011105	0.0060942
0.66766	-0.07346	-0.12912	-0.1344	-0.070954	0.18348	0.15796
0.39144	0.057702	0.11594	0.3462	0.023596	-0.055438	-0.031805
-0.58764	0.58167	0.68574	0.68207	-0.75363	-0.70029	-0.51239
0.70003	-0.72485	-0.98457	-0.76813	-0.026408	0.011915	0.17122
-0.53464	0.27265	0.34284	0.54455	-0.21849	-0.26892	-0.17794
0.87495	-0.28457	-0.46722	-0.2392	0.19632	0.044259	0.047025
-0.3269	-1.0092	0.073441	-0.15189	-0.091332	-0.21436	-0.24528
1.7466	0.15416	0.39322	0.13698	-0.1057	-0.084891	-0.17999
-0.0464	0.0042935	-0.027536	-0.12022	-0.024175	0.10649	-0.092974
-0.15006	0.22469	0.036777	0.16694	0.020894	0.099598	0.22552
-0.20029	-0.099158	-0.065502	-0.10053	-0.086251	0.096083	0.016844
0.418	-0.02836	-0.15129	-0.10165	-0.021346	0.086052	0.051141
1.1601	0.228	0.014624	0.045364	-0.12515	-0.14609	0.052904
1.3345	-0.31873	-0.36714	-0.27765	-0.15074	0.0215	0.036704
0.95493	0.26598	0.099604	0.018837	-0.21851	-0.17399	-0.408
0.048713	0.24474	0.055701	0.14471	0.073923	-0.050611	-0.14558
-0.042555	0.23459	0.32718	0.18168	0.00893	0.12214	0.059314
0.21377	0.39856	0.18809	0.19529	0.026493	0.18023	0.16887
1.1763	0.041153	-0.074893	-0.10387	0.18036	-0.026658	0.042387
0.57444	0.05691	-0.4353	-0.34163	-0.3206	-0.089425	-0.27379
0.92321	-0.24512	-0.20409	-0.15937	0.38357	0.47	0.40798
-0.31194	-0.34802	-0.1652	-0.17328	0.24774	0.46937	0.42827
0.41557	-0.16327	-0.29791	-1.1258	0.013513	-0.10915	-0.010367
-0.091565	-0.041298	-0.18723	-0.1828	0.035815	-0.026012	-0.022478
0.79958	-0.25937	0.075484	0.1966	-0.073661	-0.20604	-0.063113
1.4465	-0.21384	-0.54348	-0.22171	-0.038789	-0.23062	-0.013263
0.21322	-0.16551	-0.11786	-0.041456	-0.1142	-0.053169	-0.12518
0.82952	0.01503	-0.16804	-0.31941	-0.0098395	0.05621	-0.0092047

-0.35521	0.17361	0.23451	0.20386	0.038917	-0.027371	-0.054408
-0.063772	0.045512	-0.074433	-0.0062144	0.13221	0.2081	-0.026605
1.7033	-0.070149	0.10421	0.016342	-0.020592	-0.12906	-0.35723
-0.12533	0.067093	0.13468	0.20112	-0.0009839	0.10325	0.028995
0.49495	-0.27949	-0.21719	-0.29476	-0.0041984	-0.17845	0.10332
1.2879	-0.46008	-0.52381	-0.70625	-0.04056	-0.036876	-0.14981
1.8045	-0.6241	-0.8338	-0.84812	0.025546	-0.10056	0.0634
0.58596	-0.37366	-0.42965	-0.40177	0.014259	0.044433	-0.14583
-0.04109	0.00022979	-0.19677	-0.028729	-0.018803	-0.075642	0.037437
0.76546	-0.05316	-0.33446	-0.23724	-0.045214	-0.061327	0.025781
0.091542	-0.12747	-0.097989	-0.29897	-0.026355	0.0033314	-0.13735
-0.13877	0.012894	0.13997	0.16829	-0.039263	-0.10211	0.0019541
0.15077	-0.2743	-0.17022	-0.18055	0.19743	0.22368	0.954
0.2162	0.011653	-0.095143	0.049547	-0.47168	-0.51065	-0.61432
0.14773	-0.80388	-0.85364	-0.75304	0.40443	0.71618	0.58464
0.33191	-0.74152	-0.14371	-0.23583	0.13398	0.47814	0.36525
0.094662	0.070986	-0.17611	0.1062	0.43378	0.088475	0.19618
0.69538	0.16709	-0.14329	-0.087458	-0.24185	-0.46614	-0.39034
0.19121	0.14795	0.13222	0.03121	0.097754	0.016625	0.0087255
0.16663	-0.027185	-0.14936	0.10728	-0.075962	-0.15015	0.036619
-0.13714	0.06539	-0.27812	-0.035062	-0.04694	0.044572	0.13305
-0.18424	0.055242	0.16339	0.060445	-0.12879	-0.1905	-0.11179
0.14252	0.19326	0.059537	-0.072081	0.029091	0.068673	0.050769
0.58308	-0.042424	0.011683	0.024737	0.020079	-0.042345	-0.20092
-0.34755	-0.25781	-0.25736	-0.28743	-0.046377	-0.032902	0.011457
-0.0001522	-0.62925	-0.55339	-0.58925	0.14963	0.32211	0.49015
0.26986	-0.20651	0.052587	-0.056917	-0.030429	-0.05567	0.055381
0.76854	-0.16578	-0.28124	-0.57425	-0.033761	0.0020656	-0.17981
-0.78092	-0.99229	-1.2071	-0.73666	0.037652	-0.015909	0.14902
-0.061308	0.3472	0.76803	0.52318	-0.32491	-0.41758	-0.31436
0.28605	0.68817	0.65436	0.49709	-0.42763	-0.35171	-0.29846
0.84349	-0.26905	-0.18986	-0.18875	0.16143	0.088916	0.11872
0.31246	-0.64304	-0.42896	-0.34618	0.27674	0.31901	0.49072
-0.50921	0.0022452	0.18721	0.24797	-0.27962	-0.29614	-0.23854
2.156	-0.1079	-0.083469	-0.14392	0.078349	0.14202	0.28049
0.34731	-0.90662	-0.44255	-0.42103	0.36239	0.38824	0.38854
0.88033	-0.51689	-0.1741	-0.17693	-0.49078	-0.76624	-0.85239
0.55301	-0.2526	0.0088621	-0.19423	-0.04116	0.083642	0.03373
0.27663	0.73252	0.7524	0.66932	-1.2024	-1.265	-1.4955
-0.033739	0.46847	0.037041	-0.071019	-0.31893	-0.57236	-0.58962
1.5526	-0.059483	-0.24701	-0.18489	-0.192	-0.34363	-0.25026
0.022675	-0.59147	-0.76618	-0.53606	0.41004	0.57373	0.52483

1.3744	0.072246	-0.43184	-0.41734	-0.14011	-0.42194	-0.48546
0.094455	-0.05962	-0.1432	-0.30962	-0.39888	-0.75106	-0.86454
1.5389	-0.55036	-0.60276	-0.74921	0.071721	-0.14036	-0.32461
-1.235	0.43363	0.035889	0.36591	0.10441	-0.058176	-0.015613
0.47643	-0.035818	-0.12992	-0.013842	-0.251	-0.19563	-0.19232
0.46912	-0.32008	-0.3622	-0.23535	0.0021089	0.022762	-0.027718
0.0027615	-0.3697	-0.37878	-0.65353	-0.050607	0.020964	-0.17105
1.0395	-0.18801	-0.34521	-0.056441	-0.34767	-0.23131	-0.50109
0.73309	-0.12508	-0.28402	-0.22646	-0.15276	-0.25995	-0.14712
0.36633	-0.17238	-0.1443	0.027779	-0.059586	-0.14936	-0.075014
-0.44309	0.032486	0.10056	0.12215	-0.045449	0.16699	0.0070764
0.38696	-0.10444	-0.30279	-0.13458	0.064169	0.069519	0.019935
0.47732	0.34465	0.12889	-0.014182	-0.085013	-0.176	-0.082781
0.26468	-0.26772	-0.40104	-0.42777	-0.10055	0.11935	-0.093812
-0.049169	0.10668	0.058802	0.13777	-0.49132	-0.58151	-0.64329
-0.1194	-0.32719	-0.2341	-0.38364	-0.0608	0.053489	0.23333
0.28359	-0.32072	-0.41377	-0.32112	0.09305	-0.0003829	-0.24614
0.63921	0.13235	-0.20817	-0.10255	-0.35149	-0.50663	-0.2905
-1.1258	-0.12998	-0.03236	0.038108	0.055206	0.032702	-0.093268
-0.33138	0.19947	0.1166	0.052882	-0.085796	0.054683	-0.25094
0.54534	-0.21597	-0.22634	-0.26039	0.15826	0.23306	0.28371
1.9778	-0.47719	-0.50888	-0.34481	0.00016212	0.090894	-0.01976
1.2864	-0.44541	-0.47366	-0.51737	-0.12107	0.082602	-0.032207
0.10363	-0.057539	-0.28923	-0.26807	-0.082591	-0.010937	-0.0078255
0.22255	-0.062043	-0.092067	-0.126	0.063664	0.11764	-0.14215
1.3952	0.027795	-0.075533	-0.064764	0.020757	-0.0052	-0.12132
0.64222	-0.18587	0.02776	-0.16079	-0.0069591	-0.035897	0.075482
0.92093	0.28763	0.067923	0.044279	-0.45825	-0.36926	-0.39733
0.075514	0.523	0.5356	0.51166	0.011014	-0.097832	0.21558
-0.13428	0.76517	0.49062	0.3973	-0.15912	-0.28199	0.041113
0.75283	0.20764	0.23805	0.40309	-0.51045	-0.52545	-0.45425
0.07773	-0.40385	-0.40346	-0.31978	0.23868	0.1317	0.0069286
0.10549	-0.61318	-0.42592	-0.40198	0.0056006	-0.1077	-0.11077
-0.31586	0.15092	0.099046	0.13896	0.14107	0.13062	0.020174
0.80788	-0.35861	-0.34393	-0.45458	0.1505	0.22967	0.25432
0.31984	0.097267	0.027632	-0.036741	-0.15061	-0.058052	-0.014125
-0.21585	-0.26126	-0.51044	-0.1571	-0.097141	0.047774	0.011383
1.3601	-0.55989	-0.84703	-0.83929	-0.28885	-0.4089	-0.49584
-0.29193	0.51983	0.28465	0.12323	-0.077688	0.030319	0.10762
-0.30374	0.35168	0.25717	0.19607	-0.036596	0.21686	0.16332
0.15759	-0.043707	-0.17021	-0.17126	0.011445	-0.068991	-0.048674
0.37836	-0.028608	0.11529	-0.0065369	-0.034487	-0.2354	-0.012708

0.97056	-0.20334	-0.35121	-0.31143	-0.12418	-0.16132	-0.13027
0.88522	0.21394	0.19456	0.020289	0.044354	0.047096	0.22387
0.82477	0.29775	0.57326	0.15061	-0.0084083	-0.059245	-0.24217
-0.20889	-0.24411	-0.29888	-0.39674	-0.28831	-0.20172	-0.13085
1.8821	-0.52064	-0.69326	-0.46781	0.074339	0.047484	-0.010258
-0.53621	-0.11092	-0.13446	-0.057998	0.028832	0.065002	0.30426
1.3034	-0.037452	-0.28794	-0.18078	-0.065318	-0.22592	-0.052291
0.77598	-0.30387	-0.23358	-0.25908	-0.087444	0.02687	0.062797
1.0769	-0.19587	-0.16272	-0.12138	-0.012651	0.12287	-0.066757
0.16264	0.095597	0.012163	-0.12719	-0.019894	-0.074805	0.10194
-0.25705	0.0060438	0.26299	0.42856	0.067469	-0.016881	-0.045268
0.52388	-0.73239	-0.77884	-0.92177	-0.061193	-0.21233	-0.26502
-0.4237	0.12963	0.22373	0.032432	-0.17918	-0.25715	-0.21728
-1.43	-0.097891	-0.17698	-0.36701	-0.15344	-0.2632	-0.29501
-1.4039	-0.14851	-0.1254	-0.35112	-0.065464	0.10232	0.076956
-0.22221	-0.26675	-0.28606	-0.43385	-0.14879	-0.27203	-0.17143
-1.2338	0.025393	-0.13195	-0.18248	-0.21648	-0.21962	-0.25447
0.20132	0.070704	-0.13367	0.15087	0.083519	0.037858	0.23138
1.0468	-0.39041	-0.65789	-0.76531	-0.45548	-0.32213	-0.52822
0.72596	0.36716	0.39598	0.0341	-0.0938	-0.051701	-0.17461
-1.3337	0.94474	0.25422	0.13557	-0.3389	-0.27576	-0.26576
0.16251	-0.082806	-0.045621	-0.0201	-0.25181	-0.2717	-0.37649
NaN	NaN	NaN	NaN	NaN	NaN	NaN
-0.29854	-0.34321	-0.25397	-0.40714	0.0037264	-0.0021034	0.21768
0.31643	-0.13439	-0.16618	-0.013961	-0.20743	-0.070736	0.057799
-0.13867	-0.77986	-0.29469	-0.46862	0.13223	0.84111	0.13022
-0.32725	0.2661	0.30001	-0.082921	0.0027594	-0.32359	-0.31503
0.018989	0.32313	0.22959	0.16352	-0.12062	-0.0025167	0.038211
0.32525	-0.18139	0.056665	-0.24876	-0.19533	-0.26379	-0.34651
0.35177	-0.096591	-0.47716	0.02573	-0.025118	-0.078434	-0.13175
0.23853	0.38818	0.11725	0.43986	-0.16341	0.026404	-0.19672
-0.086681	0.1356	0.011714	0.35431	-0.17964	-0.15843	-0.29712
-0.72194	0.050742	-0.059948	-0.15368	-0.0577	0.095192	-0.13924
-0.53563	-0.088063	0.037163	0.11252	0.020306	0.090336	-0.05546
-1.4035	-0.74377	-0.34512	0.027852	0.050667	0.19519	0.029051
0.052938	-0.0078441	-0.44422	-0.057163	-0.041474	0.10991	0.077637
-0.6158	-0.38106	-0.13603	-0.22448	-0.1568	-0.20982	-0.19706
0.52912	0.11713	-0.28119	-0.13104	-0.14515	-0.11862	-0.2176
0.11383	0.08126	0.013961	0.1984	-0.0055564	0.079966	-0.023432
0.898	-0.26338	-0.097109	-0.089002	0.010057	-0.16136	-0.34109
0.66226	-0.18599	-0.12244	-0.15763	0.002418	-0.046956	-0.026883
1.2037	-0.1261	-0.32295	-0.36404	-0.21422	-0.18364	-0.29599

0.19689	-0.39618	-0.0036758	-0.19045	-0.12683	-0.10184	0.064935
0.31724	-0.2056	-0.22019	-0.29362	0.071692	0.055843	0.10386
0.31814	0.186	0.29811	0.34153	0.077389	0.091314	0.05145
0.049493	-0.17613	0.15309	-0.025646	-0.030228	-0.15131	-0.08921
0.10021	0.38883	0.33901	0.11599	-0.29589	-0.35335	-0.2495
0.17823	0.19561	0.17765	0.018594	0.060778	0.12718	0.094672
1.018	0.29433	0.4366	0.12994	-0.016843	-0.066893	0.18717
2.6009	-0.13044	0.098546	0.1037	0.63612	0.35496	0.086134
-0.05482	-0.23521	-0.3381	-0.45844	-0.25954	-0.34946	-0.14391
0.32345	0.097078	0.1255	-0.090635	-0.089818	-0.19664	-0.27858
-0.32531	0.26514	0.33402	0.25422	-0.1431	-0.11365	0.011215
0.69188	-0.44232	-0.31542	-0.32437	-0.072308	0.0086971	-0.053481
-0.17426	0.069227	-0.093246	-0.014647	-0.077524	0.11084	-0.060755
-0.31814	-0.1032	-0.03186	0.047758	-0.20458	-0.10411	-0.18797
0.59023	-0.25438	-0.32803	0.012463	-0.11073	-0.21342	-0.16434
-0.4501	0.16844	0.098539	-0.0017322	-0.12737	-0.14572	-0.17131
0.89922	-1.2542	-1.3397	-0.58644	0.061743	-0.085783	0.1191
2.3464	-0.047907	-0.14199	-0.15229	0.35466	0.40867	0.40398
0.085349	0.19902	-0.27104	-0.17533	0.16809	0.023604	-0.2376
-0.3388	0.079642	0.40415	0.16214	-0.084919	0.03572	-0.22369
-0.81487	-0.36533	-0.29297	-0.4644	0.0065655	0.062458	0.015586
0.4127	-0.22764	0.064624	0.0034863	0.018319	0.03593	-0.047336
0.30496	-0.17596	-0.2961	-0.024139	-0.31565	-0.22785	-0.36921
-0.31087	0.05975	0.24136	0.22092	0.26264	-0.027083	0.19932
0.65417	-0.12007	-0.64026	-0.48007	0.13542	0.10783	0.13847
0.15335	-0.098381	-0.10975	-0.15	-0.046792	0.057003	-0.064153
0.58328	-0.1564	0.027889	-0.10627	-0.24244	-0.16243	0.02425
0.33628	-0.22134	-0.39202	-0.49378	-0.11183	0.017493	-0.10701
0.78124	-0.057854	0.30368	0.31859	-0.55802	-0.57618	-0.92446
0.63219	0.30164	-0.15915	-0.35549	-0.37109	-0.3738	-0.66522
0.16132	-0.088107	-0.043391	-0.14122	-0.054053	-0.31472	-0.071859
-0.42531	-0.10074	-0.039752	-0.032896	-0.074966	-0.082959	0.013055
0.16869	-0.62257	-0.83858	-0.87139	-0.016009	-0.015886	0.11931
-0.68518	-0.028686	-0.0050254	-0.20567	0.12184	-0.070546	0.058756
0.020172	-0.056085	-0.17821	0.011664	0.044783	-0.0304	-0.2794
0.60305	0.19576	-0.22399	-0.13358	0.0084191	-0.26509	0.010885
0.80501	-0.3131	-0.30027	-0.3401	0.17078	0.0322	0.035685
0.67845	-0.33728	-0.33215	-0.34386	-0.10123	-0.078556	-0.066649
-0.32509	0.33379	0.3373	0.15426	-0.042277	0.039139	0.14999
-0.085189	-0.27717	-0.10644	0.012185	-0.034588	0.057724	0.020499
0.17308	-0.41948	-0.41796	-0.65727	-0.16457	-0.22788	-0.018539
-0.51847	-0.54613	-0.54043	-0.60763	-0.084738	0.079137	0.31803

0.96139	-0.12201	-0.17263	-0.10602	-0.070236	-0.13267	-0.16527
0.10783	-0.061097	-0.19743	-0.23387	-0.052634	-0.18652	0.095197
-0.089269	-0.030146	0.074117	-0.071582	0.13765	0.36023	0.58483
-0.61413	0.22409	0.08528	-0.11497	-0.15637	-0.22969	-0.18099
0.15746	-0.049538	-0.14148	-0.033254	-0.1533	-0.22788	-0.10902
1.0798	-0.11282	-0.32012	-0.37836	-0.92932	-1.0375	-1.0234
-0.014672	0.1124	0.040209	0.48421	-0.062444	0.014779	-0.012948
-0.98405	-0.14521	-0.50826	-0.095632	-0.0048094	0.028115	-0.10202
-0.27405	-0.036091	-0.093489	0.12862	-0.0614	0.12692	0.024568
0.11446	-0.19731	-0.29337	-0.27663	-0.1051	-0.1382	-0.44824
0.86369	0.15669	0.27685	0.19261	0.046187	-0.10492	-0.032988
0.85084	-0.39083	-0.38308	-0.29369	-0.097106	-0.1131	-0.30633
-0.30945	-0.16293	-0.186	-0.076269	0.011312	-0.027421	-0.19081
0.93935	0.33411	-0.0087892	0.19786	-0.36107	-0.36786	-0.48827
-1.5535	-0.59964	0.071525	-0.059514	0.52867	0.41748	0.38773
-0.19541	-0.039012	0.14502	0.15297	0.088785	0.15669	0.073907
0.141	-0.52207	-0.28901	-0.46459	-0.035874	-0.0088435	0.096136
-0.46552	-0.73702	-0.70675	-0.38568	0.093871	0.19953	0.052448
0.46247	-0.30645	-0.61074	-0.45819	-0.21125	-0.074546	-0.24879
0.094431	-0.62591	-0.50311	-0.54358	0.25703	0.089298	0.050615
-0.88427	0.082301	0.00057089	0.05195	-0.13364	-0.15285	-0.4549
-0.066224	0.26627	0.66885	0.36077	-0.02212	-0.022241	-0.087797
0.076631	0.24898	0.17261	0.19692	-0.017638	0.037877	-0.059765
-0.54219	-0.34815	-0.036919	0.08134	-0.10579	0.16837	-0.075178
-0.61927	-0.10559	-0.22921	-0.19201	0.023104	0.12537	0.048825
-0.048622	-0.10085	-0.31535	-0.23221	0.19198	0.2738	0.27945
-0.23815	0.12241	0.091526	-0.43706	-0.036915	-0.024018	-0.11868
-0.82054	0.33744	0.1634	0.23464	0.32677	-0.072816	0.10415
0.70793	-0.17141	-0.77559	-0.7375	-0.1042	-0.1519	-0.13442
-0.13261	0.05391	0.05936	0.29751	-0.057707	-0.12711	-0.23864
0.030173	-0.12912	-0.32941	-0.43416	-0.16859	-0.20782	-0.14804
0.50544	-0.22041	-0.49934	0.15203	0.016936	-0.084521	-0.060224
-0.057789	0.054619	-0.30128	-0.28805	0.10425	0.074051	-0.057283
-0.192	0.54475	0.5931	0.39127	-0.21719	-0.27352	-0.35621
0.49391	0.014245	-0.18146	-0.16912	-0.26437	-0.13321	-0.40161
0.086397	-0.41891	-0.36697	-0.38435	-0.063641	0.084359	0.034309
0.26416	-0.69163	-0.44573	-0.59522	0.14281	0.15135	0.37101
-0.24968	-0.28199	-0.45588	-0.17975	0.096932	0.011285	-0.18403
-0.80244	0.18191	-0.1332	0.022521	-0.074314	-0.066063	0.056427
0.79645	-0.5739	-0.03878	0.17274	0.48489	0.54925	0.42684
-0.17768	0.085797	0.18092	0.20493	0.066082	0.063184	-0.012901
0.16542	-0.037801	-0.21054	-0.1116	-0.026685	0.00011786	0.28843

0.41061	-0.22671	-0.27742	-0.1896	0.25208	-0.083871	0.0048919
1.1705	-0.094754	0.17457	0.26254	-0.14318	-0.048578	-0.092823
-0.65948	-0.15736	0.407	0.12434	0.57346	0.30611	0.27306
-0.10595	0.19386	0.2203	-0.0035942	0.061605	0.0059405	0.1009
-0.29686	0.14983	0.41997	0.33038	0.00057802	-0.022571	-0.17353
-0.6926	0.2	-0.035838	0.01831	-0.12452	-0.2293	-0.36678
-0.38332	-0.024104	0.090878	-0.031561	-0.067516	0.16214	-0.12042
0.41986	-0.15325	-0.15364	0.018231	-0.10625	-0.20962	-0.30896
-0.54845	0.39003	0.28581	0.27709	-0.053821	-0.096009	0.32363
0.53098	-0.032668	-0.29148	-0.23458	0.16041	0.090983	0.026833
-1.6784	1.1798	1.4157	0.78494	-0.18978	-0.097746	-0.26657
-1.0193	0.3367	0.41066	0.04198	-0.30254	-0.38396	-0.48513
-0.43885	-0.4682	-0.24097	-0.38294	-0.12013	-0.20507	-0.032089
0.28792	0.072892	-0.050361	-0.080478	0.13152	0.018701	-0.01889
-0.3104	-0.061584	-0.14133	-0.266	0.13972	0.24573	0.38668
0.12087	0.13016	0.28172	0.20854	0.19463	0.2401	-0.027478
0.49987	-0.26713	-0.47754	-0.27116	-0.035175	0.076213	-0.0046814
-0.43977	-0.10028	-0.4729	-0.21787	-0.4112	-0.48623	-0.26771
-0.40535	-0.45925	-0.41864	-0.34465	-0.03439	0.33721	0.026597
-0.13194	-0.38679	-0.45106	-0.20479	-0.011578	-0.10876	-0.18466
-0.61914	0.65958	0.63881	0.33469	-0.13771	-0.12928	-0.28775
0.11116	0.05127	0.16444	0.30533	0.013553	-0.19421	-0.037703
-0.42888	0.14936	0.45278	0.21698	-0.28269	-0.076145	-0.088543
-1.0878	-0.47366	-0.40407	-0.42877	-0.048685	-0.042722	-0.24684
-0.23795	0.35784	0.78504	0.44355	-0.065117	-0.035504	0.020249
0.67482	0.87765	0.55097	0.44286	-0.57521	-0.60722	-0.44326
-0.42031	-0.8869	0.45165	0.57339	-0.7688	-0.82711	-1.0397
-0.02363	-0.031056	0.085141	0.15796	-0.12666	-0.12078	0.012199
1.0319	-0.23189	0.03811	0.27734	0.24843	0.46874	0.076332
-0.027127	0.16737	0.016167	0.02735	0.023691	-0.042481	-0.20126
-0.39869	-0.028069	0.07939	-0.093814	0.060684	-0.0095125	0.072712
-0.14517	-0.19039	-0.35837	0.040642	-0.10549	0.1308	0.065965
-0.037163	0.05838	0.053073	0.0078919	0.068384	0.034265	-0.0072267
-0.50779	-0.075605	-0.039503	0.086795	0.22038	0.15637	0.022461
1.2975	-0.19074	-0.3748	-0.4114	0.0089841	-0.048014	-0.15195
0.59875	0.054084	0.090308	-0.036979	-0.19472	0.010708	-0.0091705
-0.48282	0.039988	-0.034592	-0.12318	-0.10239	-0.0565	-0.13444
0.34436	-0.10202	-0.67463	-0.36105	-0.0003514	-0.065441	0.19883
0.043653	-0.10991	-0.29856	0.098712	-1.0117	-1.1246	-0.82877
0.41434	-0.11886	-0.0030512	0.0049223	-0.027913	-0.098251	0.12235
0.20517	-0.11922	-0.2827	-0.30483	0.11209	0.0097398	0.067269
0.1515	0.049757	-0.074963	0.13457	-0.013073	-0.024337	-0.00619

0.36349	-0.19673	-0.39601	-0.26878	-0.10952	0.048787	-0.074401
-0.33601	-0.58269	-0.54295	-0.28905	0.18473	0.19175	0.098571
-0.089032	0.005338	-0.25388	-0.3004	0.018932	0.054939	0.051795
1.3629	-0.2005	-0.25601	0.016772	0.35425	0.23043	0.17553
0.41161	-0.15023	-0.24459	-0.31871	-0.16302	-0.19157	-0.3319
0.43105	-0.16439	-0.14452	-0.17532	-0.10623	-0.17522	-0.04999
0.547	-0.032056	-0.066057	-0.0020739	-0.1092	0.0089299	0.030384
0.15267	0.0023131	-0.11012	-0.0065634	-0.066505	-0.061955	-0.1442
1.2007	-0.055969	0.23075	0.43882	0.21191	0.24902	0.31187
-0.10721	0.044845	-0.015125	0.045204	0.037013	0.031476	0.091128
0.83539	0.097388	0.30264	0.25883	-0.021965	-0.20075	-0.12869
-0.40189	-0.24823	-0.23649	-0.22188	0.047354	-0.011776	0.14513
-0.65625	0.12016	0.27818	0.15019	-0.020746	-0.14373	-0.015251
-0.38968	0.71967	0.13548	0.48132	-0.5533	-0.5418	-0.5773
1.6264	-0.12037	0.0037445	0.12817	-0.36234	-0.50226	-0.32369
0.84878	0.2965	0.23588	0.22065	-0.24461	-0.036407	-0.07029
0.66303	0.11009	-0.0044873	-0.077129	-0.028646	-0.053308	-0.041105
0.86952	-0.50014	-0.29083	-0.36059	0.011151	-0.13146	-0.081391
0.9075	0.02669	-0.076461	0.057948	0.15417	-0.13103	-0.11026
0.22407	-0.27421	-0.34997	-0.0023932	0.062376	-0.27038	-0.083098

test_GSE178	test_GSE209	test_GSE325	test_GSE325	test_GSE325	test_GSE325	test_GSE325
REF_GSE178	REF_GSE209	REF_GSE325	REF_GSE325	REF_GSE325	REF_GSE325	REF_GSE325
GSE1780	GSE2095	GSE3250	GSE3250	GSE3250	GSE3250	GSE3250
GEO	GEO	GEO	GEO	GEO	GEO	GEO
cDNA	cDNA	cDNA	cDNA	cDNA	cDNA	cDNA
CuSO4:2	GSNO:0.2	TIME:60,DIA	TIME:60,DIA	TIME:5,DIAU	TIME:60,DIA	TIME:60,DIA
220	243	268	269	302	311	312
-0.14843	-0.45395	0.92524	0.92783	-0.51808	0.42193	0.23672
-0.34202	-0.25773	0.46842	0.3711	0.28018	0.046558	-0.14734
0.056277	-0.15638	1.4776	1.6161	2.5626	1.376	1.2598
-0.18406	-0.097388	-0.33122	-0.19061	-0.08228	-0.28715	-0.14476
-0.13891	0.08498	0.34971	0.061108	-0.57394	-0.24527	-0.57104
-0.35734	0.058129	-0.15652	0.011943	-0.1793	0.10364	0.10777
0.58108	0.94375	-2.3604	-2.2162	-2.2287	-2.6636	-2.6048
0.42535	-0.065961	1.6343	1.7764	3.0461	1.0636	1.4212
-0.024686	0.056417	0.66422	1.2157	0.63223	0.7484	0.53417
0.083134	-0.4242	0.25576	1.3551	-0.29805	0.2894	0.70745
0.25759	-0.05288	1.042	1.0253	0.046876	0.61473	0.61419
0.30969	0.023202	-1.7887	-1.584	-1.2265	-0.4725	-0.44085
-0.0057918	0.35366	0.77782	0.46575	0.27633	0.024657	0.13135
-0.45893	0.10026	0.99805	0.52409	0.071563	0.63896	0.8028
-0.19308	-0.050801	0.15028	0.25294	-0.18941	0.27671	0.23405
-0.55816	0.26801	0.65179	1.0948	1.0026	1.0605	1.2372
0.43165	0.33991	-0.61312	-0.6313	-0.56587	-0.40357	-0.57019
-0.54591	-0.43471	1.6346	1.4591	0.47547	1.3923	1.4297
-0.1988	0.023745	0.29007	0.2738	0.095745	0.23801	0.14788
-0.095269	-0.0060912	1.5266	1.3899	1.4211	1.3566	1.0901
-0.50028	0.047504	-0.51698	-0.16587	1.6887	-0.16626	-0.4757
0.51203	0.08441	0.275	0.24678	0.14472	0.29077	0.15657
0.22107	-0.41116	1.6264	1.7714	2.4413	1.6593	1.6721
0.020573	-0.21609	0.98163	1.1588	0.92937	0.94527	1.0005
0.12599	0.25756	0.92202	-0.0094716	0.62462	0.51224	-0.13027
0.34134	-0.37316	-0.18397	-0.4593	-0.86507	-0.34595	-0.23628
0.28845	0.53766	1.5429	1.2109	1.1564	1.3205	1.195
0.40128	0.072914	-0.55225	-0.62938	0.071369	-0.91949	-0.92995
0.41033	0.29627	-0.24139	0.24838	-0.078024	0.057701	0.11072
1.4014	-0.20412	-0.18212	-0.2553	-0.55327	-0.13837	-0.056005
0.22737	0.40337	-0.24389	-0.39233	-0.44671	-0.18178	-0.25064
0.23479	0.44571	0.077785	0.22134	-0.48072	0.58051	0.45548

0.45946	-0.13709	0.053635	-0.42965	0.44076	0.16228	-0.073871
-0.1784	-0.0367	0.84721	0.98307	0.60597	1.5824	1.1954
0.38304	-0.24181	1.4033	1.6312	-0.064759	1.1666	1.5349
-0.13781	0.15732	-0.30195	-0.2474	0.10285	0.027611	-0.07294
0.077711	-0.37915	0.62244	0.45319	0.58214	0.14203	-0.54321
-0.0078007	0.12763	-0.21789	-0.21466	-0.36191	-0.25904	-0.40465
-0.65021	-0.12086	-0.77367	-0.84973	-0.21127	-0.89673	-1.0178
-0.11143	-0.04754	-0.1574	-0.2853	-0.17172	0.62979	0.29716
3.7286	0.10416	0.17123	0.318	0.0012212	-0.012072	-0.019912
-0.11576	-0.25365	0.045222	-0.24943	0.038795	-0.007939	0.066816
0.28148	0.1672	-0.1321	-0.18152	-0.47394	0.099239	0.22306
-0.63617	-0.015951	1.5008	1.2882	0.36255	0.23248	0.56321
0.10741	0.49999	0.060247	0.1848	0.06223	-0.03803	-0.13035
0.41073	0.4746	-0.26288	-0.28146	0.46267	-0.38828	-0.34604
1.274	-0.21056	0.14432	0.26162	0.421	0.14661	0.12255
0.29396	0.021815	-1.295	-1.1175	-0.61443	-1.2835	-1.2153
-0.080957	-0.15346	0.56728	0.6318	0.34342	0.7024	0.99757
-0.14709	-0.077706	0.069282	-0.023904	-1.1501	0.048763	0.33741
-0.24649	0.31071	0.28761	-0.10296	-0.22483	0.13378	0.18761
-0.1598	-0.011626	0.98189	1.096	0.26063	0.72523	0.83161
-0.1002	0.75984	0.19186	0.26062	0.37717	-0.21074	-0.080226
-0.075178	-0.064506	0.32718	0.35433	0.76192	0.019702	-0.39414
0.03347	-0.26863	0.32932	0.31222	-0.083947	0.12137	0.22954
-0.037831	0.20542	-0.67221	-0.45838	-0.23404	-0.12982	-0.090471
0.10939	-0.40753	-0.28847	-0.29963	0.27197	-0.40662	-0.28339
1.0468	0.52968	0.67033	0.88448	1.7245	-0.039753	0.29393
0.7184	-0.087282	-2.4518	-2.4276	-0.94962	-2.3335	-2.2765
0.041074	-0.073573	-0.016745	-0.27426	-0.18786	0.19959	0.1268
1.5658	0.11278	0.64288	0.77191	0.27269	1.4138	1.2951
-0.03809	0.086981	0.76986	0.58823	-0.37727	0.037205	0.04372
-0.22933	0.1454	-0.4997	-0.4532	-0.24264	-0.13828	-0.51895
0.41139	0.1476	-0.17676	-0.75113	-0.0092121	-0.076042	0.18118
-0.15582	-0.92361	0.067723	-0.14613	0.20194	0.34588	0.32273
-0.080143	0.27346	1.9592	1.9256	3.1473	0.30916	0.54097
-0.50613	-0.093075	0.38933	0.58259	-0.60413	-0.0037582	-0.0004831
0.99344	0.28947	-0.14314	-0.1976	-0.26943	-0.058599	0.039186
0.4351	-0.20638	-0.20586	0.046595	0.28913	0.69455	0.49248
-0.17276	-0.057834	0.38399	0.30022	0.58469	0.23452	-0.021443
0.34694	-0.091272	-0.23696	-0.029035	-1.1474	-0.065519	0.15577
0.82967	0.16436	0.42746	0.39374	0.22573	0.18548	0.22971
0.052961	0.090769	0.065671	-0.03846	-0.27406	-0.16385	0.063791
-0.038633	0.03149	0.20866	0.15743	-0.10741	0.58189	0.492

1.3533	-0.35872	0.66717	0.9534	0.096159	0.6727	0.26888
0.044893	0.03955	-0.53429	-0.67009	-0.62892	-0.42956	-0.37984
0.032088	-0.2523	-0.45169	-0.42826	-0.25043	0.063221	-0.15813
0.038399	0.29473	0.94712	1.1195	0.27215	0.47875	0.35212
0.32545	0.25237	-0.31552	0.022867	0.15016	0.023105	-0.18101
0.30521	0.034393	0.74981	0.55964	2.016	0.5003	0.55606
0.15511	0.19651	-0.53178	-0.48961	0.91021	0.044625	-0.059411
-0.24769	0.55365	0.67059	0.73614	0.12306	0.74394	0.91828
0.20894	0.43485	0.33189	0.29829	-0.27111	0.13865	0.3633
0.30706	-0.22957	0.086057	0.079893	0.57908	-0.026361	-0.027534
0.19796	-0.2016	0.75544	0.83182	0.21772	0.74613	0.91993
0.19917	0.18546	-0.084471	-0.0031013	0.54822	0.20081	-0.056205
0.13412	0.33199	1.9309	1.8976	1.5976	1.3642	1.4669
-0.20152	-0.25974	1.3292	1.3424	0.49155	0.82533	1.3431
0.82227	0.016753	1.26	1.3651	1.7045	0.4602	0.50842
0.61982	-0.45337	0.061814	0.18197	0.013091	0.11927	0.4264
0.19861	1.4107	0.20202	0.38451	-0.058739	-0.13257	-0.21198
0.22707	-0.016591	-0.056572	0.11458	-0.4938	-0.22308	-0.055121
-0.066542	0.24395	1.1942	1.3823	0.27169	0.88317	0.94782
0.11295	-0.2152	0.83459	0.97027	-0.057285	0.77634	0.76435
0.19516	0.042828	0.52893	0.55292	0.10818	0.37248	0.50032
0.3289	-0.0064809	0.0414	0.14996	0.25386	0.54658	0.72155
0.20511	-0.20216	-0.16722	0.19982	-0.012378	0.26382	0.53835
-0.071576	0.019151	2.8161	3.0264	1.7176	3.0588	2.9874
-0.07496	0.18679	0.47275	0.51999	0.62351	0.73942	0.56489
0.42915	-0.065615	-0.41435	-1.1274	-0.5559	0.12973	-0.051848
0.15822	-0.13541	-0.44419	-0.49412	-0.46233	-0.059896	-0.47157
-0.24927	0.74991	-0.53041	-0.45006	-0.53599	-0.52738	-0.52577
0.21391	-0.13441	-0.27093	-0.22393	-0.038632	-0.37073	-0.31737
-0.17581	-0.30462	0.18308	-0.13685	0.096	0.20038	0.35958
-0.16304	0.17815	0.12649	0.27248	-0.35955	-0.33687	-0.3831
0.39553	-0.10792	0.68839	0.64833	0.52001	0.45006	1.032
0.53138	0.15383	0.67709	0.47598	0.17982	0.56753	0.53598
0.37598	-0.40126	-0.12117	-0.1872	-0.18494	0.22364	0.27685
0.36283	-0.10051	-0.30782	-0.59286	-0.30436	0.098654	0.18068
-0.033189	-0.12531	0.52877	0.67366	0.81964	0.13983	0.2468
0.25722	-0.1257	0.17648	0.29864	0.60612	0.43103	0.37405
0.3714	0.17803	-1.8391	-1.8283	-0.79453	-0.37771	-0.39405
0.016265	0.010733	0.43366	0.58935	0.20227	0.15364	0.20045
0.054861	-0.30064	0.055835	0.098063	0.48814	0.039396	-0.40244
0.15524	0.21004	-0.23172	-0.17738	0.34158	0.041429	-0.090685
0.14947	0.094641	-0.50888	-0.45155	-0.29659	-0.38832	-0.48164

0.2095	0.22734	0.24428	0.10273	0.50757	0.18246	0.61627
0.15872	0.058279	-1.1374	-1.044	-0.12251	-0.53652	-0.46145
0.22185	-0.041575	-0.08387	-0.15022	-0.46829	-0.30851	-0.28539
1.0946	0.16793	-0.0006667	0.015941	0.36676	-0.10323	-0.28433
1.1713	-0.34564	0.27603	0.20775	-0.27541	0.12127	0.39405
-0.56189	0.15229	0.75663	0.96574	0.21204	0.44969	0.61572
0.082608	1.1423	0.50309	0.42687	0.53132	0.29822	0.52778
0.43038	0.054259	0.85602	0.79817	1.1889	0.38656	0.1804
0.17212	0.045993	0.14603	0.09972	-0.23144	0.31646	0.31789
-0.045739	-0.15226	1.216	1.367	1.2762	1.0501	0.97161
0.017152	0.22855	1.972	1.9308	-0.0011379	0.41351	0.67188
0.33271	0.1696	0.57902	0.74203	-0.59779	0.60729	0.79511
-0.055959	-0.163	1.1789	1.0819	0.75676	0.38442	0.69285
-0.77638	-0.30529	-0.20383	-0.42375	-0.42546	-0.23418	-0.41388
-0.96298	0.56888	-0.46577	-0.3714	-0.14065	-0.12336	-0.081554
0.25257	-0.15734	-0.21839	-0.085104	-0.78838	0.070027	0.18715
0.23145	0.24476	-0.64572	-0.66072	0.58071	0.014021	-0.24343
-0.15576	0.057257	-0.169	-0.22216	-0.0075578	-0.3543	0.38067
1.8907	-0.20626	-0.85675	-0.85313	-2.115	-0.87419	-0.80135
0.11808	0.2042	0.059021	-0.08202	0.26546	-0.55775	-0.61478
-0.23441	-0.64968	0.11768	0.0074509	0.028625	0.34012	0.27405
-0.35853	0.081232	1.0438	0.9899	0.73063	0.87993	0.86831
NaN	NaN	NaN	NaN	NaN	NaN	NaN
0.77063	0.38841	-0.098435	-0.043016	-0.26663	0.97848	1.0058
1.1228	-0.15842	-0.15743	-0.26514	0.24436	0.2947	0.17012
0.97907	-0.019705	-0.010699	-0.034336	0.68319	0.36888	0.22541
-0.18603	1.2763	-0.17108	-0.060607	-0.13214	-0.14412	-0.084762
0.15768	-0.3177	0.1028	-0.0007953	0.0049776	0.056932	-0.091539
0.1575	-0.18853	-0.18443	-0.068292	0.37808	-0.018805	0.094411
-0.38058	-0.12578	1.6865	1.8736	0.39248	1.6524	1.8636
-0.15479	0.24789	1.7691	1.2911	2.8245	0.52466	0.82015
0.33893	0.42157	-0.0788	0.0053324	0.091569	0.1608	0.085304
0.25751	0.016995	-0.076185	-0.22619	-0.14012	-0.13033	-0.16872
-0.35794	-0.52463	0.5891	0.50317	0.28825	0.50207	0.38619
-0.4769	0.20105	-0.34576	-0.13162	0.077291	-0.067747	-0.097956
0.19298	-0.04957	0.91132	0.71429	-0.62017	0.61677	0.78598
-0.027333	0.16598	-0.6794	-0.59076	0.67548	-0.25947	-0.45633
-0.1182	1.0412	0.87623	0.50398	1.431	0.23476	0.3591
-0.036271	1.9521	1.0122	1.1571	1.0851	0.97796	0.86563
0.38646	0.024451	0.88435	0.92184	0.966	0.62537	0.59164
0.18943	0.14224	0.59222	0.55351	0.87884	-0.02947	0.41775
-0.17556	0.49641	-0.22354	-0.28025	-0.27828	-0.47829	-0.47743

0.00048075	-0.041575	0.64586	0.74002	0.38775	0.65678	0.73891
0.17301	-0.1535	-0.099659	0.10281	0.0047983	0.015407	0.019243
0.12938	0.30295	-0.037817	0.0078654	0.21099	0.11211	0.23708
0.05897	0.049479	-0.029067	0.016708	0.58213	-0.046289	-0.21923
0.13478	-0.38629	0.14355	0.080917	0.22284	0.0064581	-0.045402
-0.017772	-0.20454	0.10068	0.037205	-0.14206	-0.56641	-0.39274
0.23187	2.1154	-0.30079	-0.24993	0.098437	0.060628	0.091658
3.2373	-0.23383	-1.2626	-1.1466	-2.1653	0.18515	0.2935
0.081688	-0.038374	1.8175	1.9212	1.1731	1.727	1.8274
-0.049603	0.27847	0.32559	0.16319	-0.55854	0.33983	0.64394
0.41128	0.17211	-0.10566	-0.19254	0.25746	0.086094	-0.091087
-0.22955	0.099918	0.014928	0.26411	-0.22435	0.20164	0.061981
0.3182	-0.13301	-0.25144	-0.12571	-0.21803	-0.047531	-0.22064
0.044876	1.1524	-0.076694	0.049997	-0.39046	-0.1966	0.015634
-0.43786	0.11025	1.4652	1.548	0.74183	0.88666	0.92763
0.74023	-0.075994	0.22778	0.14233	-0.072354	0.013012	-0.015426
-0.52783	-0.0080444	-1.3407	-1.4049	-0.646	-0.48077	-1.0075
0.21726	0.18226	-1.4936	-1.7015	-0.70658	-1.1011	-1.2771
0.036588	-0.3984	-1.7553	-1.425	-1.5108	-0.82013	-1.0751
-0.052611	0.18783	0.58514	0.74465	1.5161	0.8444	0.86349
0.072682	-0.10424	0.70129	0.76309	0.072307	0.4302	0.59229
-0.34192	-0.22623	-0.077123	-0.032144	0.48161	-0.073192	-0.1075
0.046001	0.060358	-0.01617	0.059983	-0.02613	0.26415	-0.082624
-0.023375	0.027354	0.26615	0.24584	-0.068044	0.14323	0.28117
0.075232	-0.0052365	0.79332	0.75722	0.89077	0.3548	0.51336
-0.043698	0.41145	0.43619	0.39024	0.36446	0.43405	0.33685
0.43026	0.11828	0.17477	0.20453	0.14642	0.22018	0.15404
0.22499	-0.18126	0.41679	0.53257	0.34193	0.61468	0.6366
-0.050282	1.1568	0.17753	0.24992	0.12511	-0.18502	0.015816
0.30341	-0.17569	-0.0003151	-0.13511	0.16142	-0.1902	-0.18754
0.090008	0.29209	-2.0876	-2.2397	0.67551	-1.1804	-1.1725
0.17802	0.08517	0.27936	0.030782	0.22522	-0.075991	-0.11978
-0.036984	1.2615	0.80086	0.64699	0.089469	0.66636	0.71728
0.18988	0.26959	-0.13481	-0.10345	0.27036	0.10871	0.3753
-0.14989	-0.15486	1.3216	1.449	3.2971	0.7978	1.1745
0.41591	-0.16025	-0.78606	-0.85621	0.32427	-0.18785	-0.24016
-0.066307	-0.1895	0.21868	0.19559	0.44419	0.032597	0.0045307
0.29085	0.41955	0.02005	-0.023867	-0.77062	0.15495	0.096169
0.084344	-0.1955	-0.40079	0.11321	1.6872	0.033983	-0.21861
0.30695	-0.032674	0.38356	0.40609	0.4447	0.3448	0.63025
0.41798	0.0042099	0.008443	0.11237	0.18937	0.25614	0.072884
1.021	-0.02108	-0.0064815	0.11325	0.0083303	0.42001	0.38183

0.13161	-0.13853	-0.33683	-0.44881	-0.10957	-0.071627	-0.051635
-0.04199	-0.59184	0.12273	0.5739	0.081208	0.19541	0.42304
0.52064	0.25975	-0.55692	-0.54973	0.32506	-0.43315	-0.45186
0.2542	-0.2906	0.32898	-0.4227	-0.45957	0.26826	-0.40016
-0.16682	-0.37647	1.2205	1.1406	0.22933	0.68676	0.58786
0.40859	0.371	-0.18845	0.1134	-0.5408	0.34959	0.44857
0.48814	1.2453	-1.0548	-1.0759	0.33655	-0.55211	-0.68431
0.26685	-0.53534	0.35596	0.18261	0.24194	0.40968	0.085844
-0.31832	0.0046357	-0.29121	-0.25343	0.27883	-0.264	-0.42669
0.22821	0.8078	-0.54832	-0.58773	-0.94706	-0.45664	-0.5297
0.044231	-0.16432	0.42883	0.14105	0.73604	-0.13024	0.0007853
0.24207	1.2857	-0.72049	-0.62269	0.0425	-0.06907	-0.0024443
0.76319	-0.14066	0.018284	0.06858	-0.94953	-0.031524	-0.016657
0.18846	-0.067248	-0.29302	-0.21409	0.086456	-0.226	-0.29628
-0.62064	0.019656	-1.6058	-1.6585	-1.6222	-1.9883	-2.3751
0.11759	-0.04158	-0.52223	-0.79791	0.049476	0.14223	-0.0039239
0.36749	0.19772	0.18101	0.20631	0.23009	0.23804	0.41595
0.82204	0.39515	-0.76192	-0.59096	-0.60396	-0.83855	-0.60766
0.56254	-0.02269	-0.24813	-0.18549	-0.068029	-0.06161	-0.12901
0.028024	0.5566	1.2905	1.3372	0.43678	1.4215	1.4545
0.35405	0.095013	1.7044	1.6922	1.8878	1.0807	1.236
0.019371	0.01595	0.10957	0.2756	0.28458	-0.013728	0.27997
-0.15307	0.088317	-0.25914	-0.19329	-0.63954	-0.52374	-0.97639
-0.20548	-0.018292	0.47953	0.56453	0.47438	0.4061	0.51154
0.39685	-0.16602	0.38831	0.31776	-0.038443	0.32949	0.29868
-0.40698	0.053014	-0.21858	-0.45035	0.89006	0.19502	0.066156
-0.81694	-0.46961	1.4351	1.4421	-0.80558	0.61864	0.54722
-0.65699	-0.39816	2.3764	2.5242	0.58296	1.8989	1.6156
-1.3969	-0.27478	1.6949	1.6879	0.4559	1.5585	1.8639
-1.1947	0.33391	1.6835	1.7136	0.91291	1.3429	1.0919
-0.2422	0.18269	-0.1094	0.048506	-0.14421	0.035449	0.1177
-0.25951	0.73477	-0.17793	0.16983	0.24852	0.098476	0.07826
-0.23145	-0.26573	1.2605	1.3043	0.37252	0.88843	0.80615
-0.067961	0.27183	-0.50561	-0.87123	-0.79872	-0.22176	-0.47072
0.52043	-0.10141	0.20446	-0.11947	-0.32979	-0.16649	-0.026223
-0.034704	-0.34267	-0.46631	-0.56247	-0.081151	0.10967	0.064904
0.12167	0.074171	0.060678	0.055646	0.48182	0.42905	0.12532
-0.0832	-0.36777	0.81894	1.0316	1.2849	1.0093	1.1809
0.097428	0.41525	1.4771	1.3697	0.94803	0.65951	0.88111
-0.22066	1.7347	-1.1409	-1.0302	-1.1994	-1.1695	-1.2841
0.1956	-0.28418	0.91338	1.0517	-0.024483	0.49745	0.17616
0.065095	0.15851	-0.37063	-0.24629	0.16391	0.14443	0.13866

-0.17552	0.32808	-0.19896	0.16855	0.18069	-0.063891	0.021687
0.12916	0.59816	-0.20527	-0.0046162	0.22325	0.11741	0.039086
0.07053	1.0233	0.47841	0.50006	-0.16745	0.13518	0.30251
0.11993	0.59876	-0.097104	0.26255	0.28145	-0.12014	0.31208
-0.0016278	0.27297	-0.24361	-0.038542	0.37678	-0.30399	-0.12689
0.041048	-0.34317	0.36332	0.23914	0.63903	0.19286	0.17217
-0.26521	0.13976	0.41286	0.61507	-0.55099	0.096306	0.12913
0.34333	0.032191	0.19713	0.26146	-0.51492	0.18227	0.47045
-0.40442	0.31939	0.39983	0.27126	-0.34577	0.23062	0.17407
0.6871	0.62206	-0.93648	-1.0336	0.16344	-1.0709	-1.1199
-0.21224	0.30603	-0.38628	-0.44512	-0.010452	-0.76483	-0.63588
-0.18983	0.39306	0.20287	0.21627	0.53405	-0.17512	-0.027896
0.34575	0.031613	0.44794	0.16827	1.0949	0.33401	0.30173
-0.64444	-0.046199	0.35232	0.29888	0.14411	0.53124	0.40155
0.005373	0.57737	0.78676	0.81473	0.12388	1.0536	0.71164
0.14861	0.49383	0.80695	0.72294	0.30962	0.74556	0.83702
0.67462	-0.2354	0.64042	0.56168	0.78385	0.6256	0.5775
0.20464	-0.21884	-0.19575	0.10685	-0.078445	-0.3575	-0.40096
-0.30922	0.49323	-1.5421	-1.5284	-0.065299	-1.4761	-1.5276
-0.0095012	2.0551	0.20879	0.063327	-0.080666	0.28453	0.17299
-0.079011	-0.27216	0.043168	0.33604	-0.68178	-0.12221	0.12575
-0.07746	-0.079888	-0.29903	-0.2879	-0.20254	0.18028	0.25273
-0.19756	-0.041575	-0.48597	-0.77378	-1.5072	-0.52024	-0.68775
0.95352	0.20005	-1.5543	-1.2244	-0.85354	-1.8394	-1.502
0.082066	-0.05842	0.16825	0.062306	0.52185	-0.063681	-0.066214
0.18344	0.23016	-0.095301	0.021358	-0.3119	0.28948	0.30695
0.44895	0.37292	0.24338	0.065057	-0.5402	0.15039	-0.34561
0.47575	0.0048523	0.036246	-0.039081	0.2728	0.034399	-0.037091
3.3979	0.6829	-0.015413	0.09943	0.1408	-0.42493	-0.52235
-0.24297	-0.14635	0.33188	0.14831	0.37176	0.28808	-0.063535
0.080557	-0.1446	0.27897	0.25223	0.50104	0.45191	0.60159
0.27492	-0.13165	-0.036321	0.29688	-0.033057	0.049805	-0.11813
0.38384	-0.16189	-0.14005	-0.75886	0.87791	-0.29267	-0.10269
-0.16705	1.0508	0.020461	0.049139	-0.3313	0.469	0.33212
0.056015	-0.16243	1.228	1.341	1.4644	1.2048	1.3918
0.18797	1.3804	0.18724	0.41644	0.34038	0.31299	0.18583
0.097691	0.56811	0.0057435	0.038496	-0.14078	0.052323	-0.28743
0.15433	-0.18491	1.0291	1.0598	0.049375	1.887	2.0626
0.41375	-0.21939	0.1467	0.20805	0.29409	-0.42177	-0.46262
-0.17448	-0.041575	0.48603	0.7711	0.34841	0.42237	0.58241
0.24285	-0.10351	0.10178	-0.15539	0.071216	0.092	-0.14461
0.23737	-0.15298	0.096524	0.17112	0.3233	0.15236	0.02655

0.11454	0.39949	0.49594	0.57197	0.51503	0.13217	0.30634
0.18387	-0.022817	-0.73834	-0.58911	0.32312	-0.25322	-0.3101
-0.21332	-0.042968	-0.50212	-0.64874	0.90415	-0.19426	-0.56109
0.13698	-0.30581	0.24057	0.1951	-1.0038	0.040241	0.043585
0.015304	-0.30689	-0.12307	-0.50742	-0.78452	0.15794	-0.26451
0.27625	0.0075179	-0.56398	-0.57503	0.013686	-0.28259	-0.15401
-0.46933	-0.043361	-0.12739	-0.025957	0.89177	0.17591	-0.15455
-0.36375	-0.14576	1.4373	1.5796	0.63927	0.77525	0.73436
0.0086381	-0.22565	0.48852	0.3619	0.091958	0.14517	-0.49436
-0.036457	0.0902	0.59084	0.59629	0.40609	0.15295	0.12215
0.24545	-0.041594	0.71859	0.71811	-0.11925	0.62991	0.5446
0.16278	-0.23385	0.37066	0.26457	2.8366	0.34341	0.78428
-0.73714	0.9572	0.013397	0.19217	0.080987	0.1192	0.17231
0.048294	0.53396	-0.53215	-0.62577	-1.8031	-1.0674	-0.97547
0.14016	-0.014987	-0.33911	-0.082628	0.39387	-0.26624	-0.18491
-0.2263	-0.065205	0.54891	0.45532	0.18708	0.060324	0.29509
0.16954	-0.156	-0.73018	-0.76095	-0.25081	-0.61156	-0.75152
0.20631	-0.21058	0.77064	0.83561	0.85139	1.5548	1.7033
-0.014574	-0.17317	-0.4308	-0.16056	-0.3034	0.49606	0.030019
-0.060735	0.06899	1.2597	1.6712	1.3875	0.94932	1.0449

test_GSE325	test_GSE325	test_GSE325	test_GSE325	test_GSE325	test_GSE325	test_GSE325
REF_GSE325	REF_GSE325	REF_GSE325	REF_GSE325	REF_GSE325	REF_GSE325	REF_GSE325
GSE3250	GSE3250	GSE3250	GSE3250	GSE3250	GSE3250	GSE3250
GEO	GEO	GEO	GEO	GEO	GEO	GEO
cDNA	cDNA	cDNA	cDNA	cDNA	cDNA	cDNA
TIME:60,DIA	TIME:60,DIA	TIME:60,DIA	TIME:180,DI	TIME:240,DI	TIME:240,DI	TIME:300,DI
314	315	316	323	327	329	346
0.19191	-0.55945	0.018567	0.94795	0.64501	0.032654	0.41856
1.5395	-0.019754	0.0899	-0.40134	-0.055473	1.8112	1.2455
1.4158	-2.4624	-1.7812	0.97413	0.93306	1.1862	1.1423
0.37592	0.10489	0.14341	-0.23277	-0.306	0.038245	0.738
-0.11174	-0.33387	-0.3818	-0.73949	-0.50549	-0.77376	-1.1727
0.4295	-0.34182	-0.046584	-0.14775	-0.3209	0.24831	0.11551
-1.7188	-0.5835	-0.41955	-1.9678	-1.9359	-1.3263	-1.6035
0.89473	0.51813	-0.65919	0.97069	1.3414	0.45461	0.57119
0.90963	-1.1402	-1.1409	0.9246	0.58947	0.77234	1.4158
0.71124	-0.096014	-0.024891	0.47703	-0.060002	0.26623	0.11695
0.10223	-1.1409	-0.95495	0.18757	0.051098	-0.0057075	-0.069784
0.51515	1.3073	1.3248	0.18034	0.1008	0.84366	0.86056
-0.20404	-0.24135	-0.1631	0.14091	0.24977	-0.24822	0.90443
-0.24214	-0.17729	0.041566	-0.13101	0.67664	-0.24642	-0.038499
0.33065	0.41722	0.45656	1.122	0.2355	0.29067	0.37201
0.46976	-1.342	-1.0356	0.75222	1.3377	1.395	1.0563
-0.14052	0.92115	0.66041	-0.45459	-0.4528	-0.53159	-0.29841
0.6943	-0.54452	-1.7724	1.0011	0.73475	-0.35977	0.089265
-0.15335	-0.59758	-0.56673	0.18547	0.081448	0.26813	-0.33188
0.52285	-1.9658	-1.8433	1.1362	1.44	1.0572	1.2365
0.34544	-0.77179	-0.81052	0.27143	0.16292	-0.81475	-0.19554
0.18229	0.41297	-0.13959	0.50094	0.1567	0.29962	0.15219
0.73426	-2.2653	-1.5596	1.1386	0.98819	0.67868	0.83195
0.48986	-1.6137	-1.445	0.25294	0.20618	0.020208	-0.27708
0.82428	-1.1001	-0.99701	0.65689	0.264	0.65556	0.73562
0.29558	0.45776	0.78343	0.10105	0.053802	0.90944	0.71358
3.4461	-2.5805	-2.7508	1.6164	1.0678	1.586	1.3038
-2.1024	0.38377	0.67747	-0.47365	-0.49969	-1.5561	-0.66104
0.048956	0.25131	0.1853	0.209	-0.052432	0.58401	0.15264
0.25973	0.0018937	0.049629	-0.31384	-0.30037	0.76643	-0.48344
-0.8784	-0.062714	0.12697	-0.14705	-0.34101	-1.1618	-1.1157
0.3372	0.076433	0.0061141	0.9577	0.71728	0.46891	0.54617

0.72484	0.282	0.33209	-0.028427	-0.085069	0.30526	0.2898
0.048786	-1.0613	-0.91444	0.60258	1.3154	0.71466	0.83591
1.2615	0.16954	-0.11053	1.0642	1.2643	1.2412	0.98143
-0.51336	-0.0484	0.12351	-0.014072	0.094537	0.25796	0.019004
0.35857	-0.55168	-0.51472	0.16546	-0.034367	-0.22989	-0.47217
-0.01475	-0.37105	-0.29483	-0.33538	-0.3396	-0.82594	-0.40588
0.99013	0.51629	-0.58287	-0.20011	-0.41812	0.44987	-0.11571
-0.59953	-0.22369	-0.24458	0.1441	0.25656	1.0387	0.49545
0.49256	0.25787	0.078743	0.14513	0.36961	1.2632	1.2275
0.33441	0.41244	0.30539	0.25265	0.26173	1.9231	1.4027
0.98831	0.55796	-0.57966	0.23643	0.22209	1.0204	1.1539
0.11554	-0.6474	-0.48792	0.96379	1.1257	0.29465	0.83977
0.29971	0.17183	0.49003	-0.02682	-0.2872	0.43154	0.47152
-0.25295	0.36057	0.44834	0.08327	-0.19264	-1.6776	-0.11736
0.60518	-0.14997	-0.25999	0.17724	0.33553	0.41914	0.081085
-1.1715	-0.22286	-0.048783	-0.33934	-1.0815	-1.2273	-1.6153
0.65405	-0.93715	-0.75693	0.63879	0.87869	0.35798	1.1583
0.43259	0.41819	0.24728	0.10319	0.11692	0.31837	0.69816
1.0234	0.18435	0.024441	0.20875	0.20331	0.89138	0.64894
0.55933	-0.22768	-0.34303	0.44952	0.66806	2.2516	1.3273
0.21254	-0.14837	-0.049412	-0.068633	0.23125	-0.019824	0.41712
-0.26832	-0.89141	-0.96873	-0.50703	-0.51622	-1.0092	-0.94501
-0.17207	-0.40144	-0.090605	0.55338	0.59587	0.33733	0.006757
0.27266	0.070733	0.36477	-0.026166	-0.0082266	0.068864	0.21307
-0.5222	-0.58008	-0.46579	-0.42407	-0.35272	-0.18058	-0.33432
2.0775	-2.4295	-1.2799	-0.10769	-0.052703	2.0049	2.3752
-1.318	2.7007	2.7863	-2.1166	-2.208	-1.97	-1.8485
0.51809	0.22018	0.11071	-0.05979	-0.16081	0.45649	0.64639
1.6798	-0.658	-1.2453	1.139	0.6633	-0.31853	-0.81573
0.069579	0.066227	-0.57801	0.067544	0.12028	-0.03316	-0.37768
-1.5615	0.23733	-0.49979	-0.19895	-0.11709	-2.8625	-2.567
-0.079782	-0.49392	0.29281	0.099514	0.22117	1.1849	1.3363
0.81692	-0.59307	-0.42533	0.039201	-0.12744	0.61433	0.59365
-0.51275	-1.8086	-1.8088	-0.90658	-0.68256	0.88373	0.36073
-0.37334	-0.81797	-0.78617	-0.93417	-0.99729	-0.81735	-1.8072
-0.020156	-0.11558	-0.021271	0.50406	0.47552	0.38213	0.56052
0.64278	-0.15471	-0.063589	0.12048	-0.1692	0.54407	0.052993
-0.12718	0.48679	0.55	0.2462	0.46325	0.12374	0.38882
0.026852	-0.5594	-0.34631	0.14664	0.080784	0.47064	0.30506
-0.32025	-0.4009	-0.40192	0.14532	0.18095	-0.55734	-0.1315
-0.47577	0.19663	-0.29121	0.13778	0.34283	0.92674	0.42784
-0.52311	-0.026311	-0.097206	-0.0088514	-0.10683	-0.89985	-1.5503

1.3441	0.14013	-0.046175	-0.35059	-1.0915	0.65151	-1.3256
-0.85389	0.28892	0.43047	-0.79357	-0.80386	-1.2658	-1.705
-0.14481	0.091672	0.22857	-0.24005	-0.12697	0.42315	-0.047878
0.19623	-2.3745	-2.0507	0.026122	-0.15979	-0.41332	-0.45611
0.25267	-0.34613	-0.45461	-0.16181	-0.062548	0.19684	0.30565
-0.32627	-1.1512	-0.26728	0.77884	0.67432	0.37446	0.4166
-0.16164	0.57858	0.45296	0.27753	0.2283	0.38726	0.19203
1.3801	-0.28201	-0.41579	0.42714	0.23383	0.3092	0.2162
0.29768	-0.056067	0.10855	-0.16325	0.019116	0.36579	0.40084
-0.20944	0.32664	0.031356	0.19762	0.20433	0.53582	0.090866
-0.25496	-0.50274	-0.27174	0.37036	0.73953	1.2684	0.6152
0.86389	-0.44804	-0.36325	0.19153	0.056449	-0.10001	0.8222
0.25317	-2.6655	-2.2385	0.73142	0.81967	1.2287	1.0913
1.8587	-0.74374	-0.46918	0.64828	0.66582	0.66067	0.92292
-0.43285	-1.7139	-1.5956	-0.096657	0.27344	0.15873	0.011025
-0.014729	-0.89243	-0.75235	0.36579	0.32022	0.084316	-0.017479
-0.18219	-1.028	-0.83813	0.55776	0.81115	1.1806	1.3559
-0.56706	0.64534	-0.10397	-0.027432	0.32629	1.0239	0.90032
1.9633	-1.5939	-1.6801	0.2469	0.12498	-0.39984	-0.067947
0.22001	0.78925	-1.6142	0.78631	0.81903	-0.51975	0.47342
-0.81198	1.088	1.0739	-0.18935	-0.074458	-0.91614	-0.85414
0.9813	-0.30794	-0.15546	0.33639	0.033484	1.1011	1.152
0.027236	0.03641	-0.087111	-0.010446	0.33617	0.48955	0.24347
1.2631	-2.0429	-2.217	0.88044	0.84861	1.5925	1.4212
0.5257	-0.69536	-0.40372	0.49564	0.68855	1.5816	1.4472
-0.64888	0.28961	-0.35011	-0.46654	-0.25944	1.3078	-0.84567
-0.43383	0.53915	0.44795	-0.289	-0.27816	-0.57347	-0.63693
-0.59859	1.0133	0.98107	-0.1656	-0.13462	-0.046128	0.2648
-0.29321	1.0093	0.76111	-0.18967	-0.32715	-1.1956	-1.0525
-0.56671	-0.22532	-0.012883	0.21941	0.3208	0.64314	-0.65741
-0.26852	0.072428	0.22221	-0.5451	-0.48822	-1.0976	-0.76295
1.2322	0.55089	-0.89675	0.72848	0.81399	-0.1716	0.6125
0.43262	-1.8019	-1.4516	0.74816	0.70447	0.92668	0.88272
1.1566	-0.19526	-0.23834	-0.019329	0.36746	0.92485	1.7431
-0.07824	0.37299	0.40496	0.13991	0.12481	1.1359	-1.3607
0.32627	-0.42687	-0.3745	0.15865	0.11614	-0.19859	0.25921
0.33765	-0.77543	-0.42484	0.29658	0.51439	1.6422	1.3679
0.27694	0.030825	0.96793	-0.56138	-0.74903	0.49721	0.74989
0.63898	-0.49423	-0.27533	0.07852	0.20372	0.76332	0.75134
0.6746	-0.091303	-0.23207	-0.18299	0.099136	0.2361	0.65881
-0.87256	-0.16812	0.069687	-0.67542	-0.17801	0.081635	-1.3562
0.016097	0.16962	0.1645	-0.18966	-0.12744	-0.6884	-0.38891

0.20133	-0.98667	-1.4299	-0.2635	-0.14532	-1.3354	-0.36067
-0.6536	0.23424	0.56964	0.013261	-0.23869	-0.2512	0.11262
0.08101	0.8107	1.1311	-0.25821	-0.21764	1.276	1.1549
-0.33571	-0.53315	-0.39857	0.13291	0.15542	0.10585	-0.57265
0.22847	-0.30804	-0.37295	0.21412	0.06895	0.0030917	-0.13178
0.35971	-0.070335	-0.66275	0.82777	0.62903	0.084152	0.64408
0.11026	-0.39174	-0.54941	0.48624	0.49821	0.19052	0.30159
0.84596	-1.6393	-1.558	0.058492	-0.084451	NaN	0.61901
0.37277	-0.23067	0.1032	0.16765	0.1296	0.15061	0.20396
0.45586	-1.7592	-1.3687	0.1472	0.45436	-0.075094	0.64679
-0.017262	-1.1676	-1.0573	-0.065524	0.032187	-1.1632	-0.65224
1.4725	-0.99045	-1.2395	0.49834	0.78961	1.3181	1.1114
0.24255	-1.3496	-1.4503	0.1553	0.088972	0.019999	0.28098
-0.34129	0.070313	0.57991	-0.010171	0.15745	0.044305	0.47792
-0.39657	-0.0038569	0.20784	-0.060756	0.15349	0.10517	0.43495
0.35662	0.48138	0.60331	-0.11349	-0.064415	-0.43241	0.061605
0.77377	-0.94377	-0.6263	0.047551	0.21579	-0.216	0.10931
0.82562	-0.5443	-0.52905	0.10123	0.53451	0.94704	1.183
-0.59934	0.99435	1.0868	-1.0128	-1.2541	-0.93527	-0.66758
-0.56802	0.075597	0.27953	-0.12081	-0.084865	0.61064	-0.28381
0.29328	-0.28621	-0.098492	-0.0026011	0.3013	0.76292	0.57466
1.2092	-1.0549	-0.95697	0.76499	0.68385	0.41826	0.95631
NaN	NaN	NaN	NaN	NaN	NaN	NaN
0.99214	-0.31115	-0.52427	0.87459	1.1086	0.95531	0.9881
0.48115	-0.36978	-0.27002	-0.029637	0.289	0.58353	0.60608
0.21896	-0.26409	0.090349	0.29683	-0.098161	1.5535	0.66677
-0.73301	-0.092836	-0.024316	0.0045651	0.073547	-1.2475	-0.74661
-0.07106	-0.23594	0.22667	-0.070746	-0.14917	-0.76491	-0.47382
0.14054	0.29601	0.23417	0.41086	0.50961	0.2658	1.0875
1.5287	-1.7312	-1.6386	2.2182	2.1848	1.2565	1.6738
0.24866	-1.9506	-1.8349	0.45286	0.29407	0.91348	0.67243
-0.36158	-0.018913	0.03717	-0.26769	-0.25489	0.58283	-0.20443
-0.18729	0.60837	1.0145	0.67752	0.37796	0.1844	0.41916
0.16503	-0.87863	-0.51372	0.14801	0.21774	1.1666	0.53213
0.47266	-0.070333	-0.37145	-0.023353	0.096452	0.19348	-0.34443
0.6918	-0.44786	-0.33394	0.98013	0.61002	0.2634	0.45724
-0.10939	-0.20078	0.45331	-0.51565	-0.45869	-0.25247	-0.22947
-0.12258	0.39609	0.44086	0.097284	0.2346	0.084376	-0.098169
0.057358	-1.8735	-1.6889	0.44934	0.63547	0.90063	0.89505
0.30943	-0.99398	-0.63252	0.66485	0.76246	0.3687	0.8019
0.85785	-1.0359	-0.78672	-0.11285	-0.21117	0.27193	0.12408
-0.26498	0.086714	0.074468	-0.64829	-0.6783	-1.6903	-0.36726

0.64399	0.28142	-0.12974	0.34257	0.67942	1.6933	1.5466
0.041028	0.22221	0.14965	-0.18432	-0.19784	0.074207	-0.0051544
0.70592	1.2211	1.1302	0.11808	0.2523	1.2223	1.253
0.13059	0.45294	-1.0044	-0.022925	0.08265	0.10843	0.25736
0.29537	1.2232	-1.5512	-0.010627	-0.080557	0.15886	0.56805
0.036902	-0.035639	0.20281	-0.36154	-0.064229	0.095114	0.31478
0.97783	-0.45089	-0.39664	-0.099345	-0.1225	0.43153	0.67137
0.57906	0.76882	0.7102	0.53475	0.32463	1.3078	1.1318
1.0003	-1.799	-2.0239	1.0699	1.2385	1.7494	2.0184
0.068258	0.44089	0.43555	0.21977	0.56769	1.0658	0.28786
0.0019742	0.10588	0.19923	-0.088186	-0.10134	-0.10079	-0.31997
0.36036	0.1335	0.24751	-0.0019275	0.11894	0.18888	0.69169
0.024313	0.40713	0.18219	0.074575	0.021776	0.042681	0.25181
-0.48564	-0.44355	-0.24756	-0.14876	-0.26976	0.17266	-0.61532
0.89377	0.18616	-0.31082	0.74302	0.70532	0.25134	0.694
0.71627	-0.46264	-0.52742	0.063302	0.06686	0.019054	0.49602
-0.39093	0.91009	0.72945	-0.57856	-1.1798	-0.90251	-0.37871
-1.556	-0.20847	-0.46506	-1.4268	-1.1983	-1.2641	-2.4
-0.54306	1.8418	1.7419	-0.83033	-0.60679	1.5061	-0.5778
0.60193	-0.64899	-0.65821	0.43105	0.15207	0.84362	1.3079
0.31265	-1.9267	-1.2378	0.57027	0.29233	0.52752	0.79025
-0.14277	0.16454	0.24748	0.059528	0.013206	0.045763	0.32978
0.29428	-0.23991	-0.25849	0.32183	0.51835	1.1607	0.89618
-0.19473	0.068262	0.10816	0.19903	0.59131	0.0080733	-0.061997
0.90546	-1.5372	-1.4697	0.068025	0.25741	2.0166	0.27328
0.45653	-0.78459	-0.53768	0.50445	0.78321	0.59478	1.2229
0.20825	0.41164	0.22212	0.080725	0.069441	0.042566	0.4895
0.68934	-0.62134	-0.44606	0.27439	0.16514	1.022	0.72774
-0.077197	-0.14044	0.081539	-0.050528	-0.13284	0.067234	-0.0094205
0.010099	0.19549	0.46818	-0.45667	-0.56752	0.41981	0.20533
-0.78728	0.81822	-0.39161	-1.1298	-1.3377	-1.0368	-0.92566
-0.0049977	1.1633	1.0832	-0.38802	-0.11948	0.19707	0.043651
-0.12691	-0.25892	0.29948	0.020685	0.42977	0.72981	0.32924
-0.25256	-0.082601	0.054246	-0.018511	0.038851	0.65935	0.68536
0.18062	-3.0546	-3.6166	0.27827	0.44393	-0.042034	-0.41387
0.025078	-0.07047	0.0040401	-0.35767	0.036446	-0.1892	-0.37959
-0.31939	-0.79958	-0.59254	0.097954	0.041115	0.59642	0.30287
-0.10382	0.1599	0.18249	0.2522	0.464	0.57887	0.3229
0.45866	-1.6919	-1.383	0.0014314	0.32501	1.4332	1.1818
0.17843	-0.49963	-0.61656	0.095676	0.12339	-0.066565	-0.95759
1.3708	-0.26818	0.092041	0.0087722	-0.09963	1.116	1.1039
1.1986	-1.3241	-1.6546	0.48253	0.42972	0.63504	1.1769

-0.054141	-0.77331	-0.52038	0.11367	0.096778	0.43297	0.1386
0.10944	0.72302	0.54771	0.49693	0.59459	0.49664	0.49081
-0.32031	0.020384	0.0058917	-0.10775	-0.04087	-1.0381	-1.334
0.050599	0.37097	0.42109	0.09486	-0.27723	1.3713	0.059772
0.46821	-0.34536	-0.42781	0.15922	-0.05424	0.41523	0.23014
0.5561	0.24448	0.36143	0.36473	0.27582	0.075389	0.35901
-0.19867	1.0797	1.0388	-0.43053	-0.032841	0.42723	0.41343
-0.66451	-0.86241	-0.60242	0.085369	0.044848	-0.65708	-0.48078
-0.16295	0.81042	0.84258	-0.61463	-0.74303	-0.41458	-0.32788
0.042593	0.2626	-0.16284	-0.1229	-0.39517	-0.62966	-0.93217
-0.23617	-0.063815	-0.61144	0.13962	-0.16734	0.67167	0.32157
0.66868	0.37935	0.87522	-0.079805	-0.095927	1.095	1.0839
0.21939	0.5188	-2.643	0.25878	0.096974	-0.11188	0.022297
0.13645	-0.55583	-0.3141	-0.30714	-0.14762	0.67695	0.66765
-1.7999	0.50393	-0.38127	-1.8209	-1.8603	-2.4545	-2.7677
-0.20458	0.11867	0.069491	0.1933	0.13456	0.47909	-0.55904
0.84504	-0.44031	-0.24724	0.49355	0.67495	1.4971	1.463
0.22379	0.36607	0.40961	-0.8233	-0.62245	-0.086738	-0.12305
-0.012457	0.025101	-0.095514	-0.16232	-0.11175	0.86586	1.0186
0.32876	0.27512	-0.076181	0.69581	1.0414	1.3474	0.24163
0.2361	-1.9138	-1.7158	0.27097	-0.20622	-0.10939	-0.067402
0.56116	0.21409	0.22719	0.41795	0.40146	1.6259	1.2493
-0.48799	0.96295	0.9342	-0.42138	-0.59816	-0.74094	-1.0415
0.7922	0.057982	0.51283	0.26138	0.038308	0.0076691	-0.088616
0.35721	0.34828	-0.0119	0.49134	0.4345	-0.12061	-0.23775
-0.43778	0.26162	0.34769	0.36715	0.48254	-1.0631	-1.2433
-0.6282	-1.6552	-1.635	0.471	0.56828	-0.9555	-1.5892
0.8719	-2.5897	-1.1849	1.5645	1.7803	-0.60933	-1.3552
1.6327	-1.7952	-1.81	1.7832	1.9225	0.36562	0.43075
1.9229	-2.0798	-1.7641	1.9108	1.8681	0.39379	0.45346
0.24031	0.058687	0.25058	0.017959	0.18523	0.18543	0.45147
0.081859	0.89088	0.75469	-0.21782	0.12836	0.18178	0.36322
0.86694	-0.70525	-0.92068	1.9263	1.9613	-0.38772	0.77034
-1.7455	1.196	1.337	0.025223	0.23964	-4.3836	-2.9597
0.55282	-0.047881	-0.04178	0.15353	0.054533	1.3218	1.3386
-0.43233	-1.4877	-0.76448	-0.12631	0.132	0.059529	0.1292
0.16578	-0.88027	-0.54443	0.39653	0.37595	1.0603	0.54247
0.80957	-0.14653	-0.076218	0.52406	0.59646	0.3561	-0.0033094
0.49216	0.091648	0.57845	0.57576	0.30684	0.2628	0.70085
-1.1718	-0.73035	-1.2774	-1.4479	-1.2994	-0.67211	-1.2525
-1.0972	-1.1135	-0.98182	0.55234	-0.19967	-0.99872	-0.25217
-0.10065	-0.3101	-0.14962	-0.021735	0.17275	0.43499	0.043361

0.27212	-0.01127	0.14653	-0.066804	-0.063598	0.27927	0.23148
0.26674	-0.13906	0.32886	0.069153	-0.17001	0.67068	0.33708
0.23036	-0.28446	-0.36333	0.32381	0.43732	0.077986	0.43442
0.2378	0.34552	0.17511	0.051882	0.15976	0.9507	0.75637
-0.15846	-0.28076	-0.074952	-0.68277	-0.3909	-0.51312	-0.011051
-0.18256	0.097838	0.64515	0.042038	-0.10041	0.19029	0.31066
-0.073991	-0.40714	-0.37294	-0.27523	-0.28571	-0.53161	-0.55821
-0.33731	-0.93459	-0.41381	-0.015663	0.051288	-0.25354	-0.17343
0.32312	0.57602	0.50163	0.28413	0.54271	0.13036	0.36061
-0.73194	0.24971	-0.16534	-0.1018	-0.248	-0.51764	-0.2704
-0.74679	0.9423	0.70824	-0.67731	-0.38653	-0.16733	-0.6129
0.23552	-0.25477	0.077203	-0.22802	-0.46446	0.01261	0.25349
0.7553	0.64872	0.54255	0.39245	0.12245	1.6809	1.3612
0.48569	0.12595	0.56032	0.42018	0.57517	1.6629	1.1338
-0.13212	-0.52462	-0.42159	0.064471	0.34616	1.3194	0.70951
0.16063	-0.38395	-0.057706	0.53978	0.16126	1.1585	1.2719
1.0113	-0.32669	-0.46617	0.40277	0.34561	0.58881	0.45057
-0.93743	0.69271	0.45513	-0.39763	0.063881	-0.059375	-0.33941
-1.6022	0.99911	1.0904	0.12613	0.31545	-0.92383	-0.58893
0.55412	0.24131	0.030882	0.33994	0.37867	-0.14013	0.82694
-0.10932	0.64356	0.83928	0.33174	0.13626	1.0016	0.53164
0.42548	1.313	0.22891	0.32516	0.063048	0.25246	0.60375
0.0909	-0.40281	-0.37088	-0.34273	-0.50028	-0.48831	-0.78458
-1.5881	1.9534	1.5544	-0.41706	-0.46472	-1.4828	-1.323
0.065388	0.025614	0.26132	-0.25258	-0.12999	-0.51855	-0.19593
0.63583	0.37082	0.37573	0.073283	-0.10029	-0.10778	-0.072751
0.49845	0.015576	0.010945	-0.252	-0.37103	0.27051	0.81313
-0.13137	-0.97942	-0.67923	-0.093106	0.19204	0.93355	0.15053
0.25541	-0.24092	-0.35936	-0.068259	-0.072981	0.90046	0.77972
0.97598	-0.12377	0.067444	0.8188	0.40923	1.0441	1.4277
0.37397	-0.72976	-0.51091	0.044822	0.17264	-0.21264	0.5789
-1.0707	-0.056733	0.27339	-0.093191	-0.18573	-0.034043	-1.2842
0.6712	-0.85214	-0.7991	-0.26705	-0.2433	0.43091	0.17015
0.61039	-0.14852	0.043525	0.64318	0.31246	0.16418	0.60548
0.025029	-0.11861	-1.4394	0.58411	1.0106	1.4	0.77121
0.034127	-0.51961	-0.59561	0.11129	-0.23423	0.78437	0.37803
-0.10822	-0.26894	-0.16924	-0.021024	0.074607	-0.20587	0.23958
3.1437	-1.2361	-1.3586	1.6988	1.4842	1.3015	1.8134
-0.16953	0.13203	0.02503	-0.45466	-0.38833	-0.49796	-0.76234
0.68961	0.17724	0.46493	0.36536	0.52923	0.74016	0.4839
-0.84919	0.6264	-0.17219	0.27656	0.068739	1.0941	0.4049
0.29582	-0.0059737	-0.092681	0.12609	0.16052	0.13177	0.10731

-0.26251	1.1634	-1.3455	0.34002	0.17294	-1.3219	-1.1991
-0.71949	-0.3936	-0.47337	-0.042387	-0.012547	-0.95932	-0.91384
0.53669	0.23098	0.14598	-0.39316	-0.24446	-1.1341	1.3342
0.21172	0.16909	-0.5559	-0.30972	-0.47134	-0.55243	-0.68552
-0.28963	0.66868	-0.14069	0.24916	-0.35332	0.51567	-0.49285
-0.46647	0.60564	0.65423	-0.036322	-0.013762	-0.83533	-0.21817
-0.010912	0.15845	0.23576	-0.128	-0.12879	0.69159	0.1488
0.045152	-0.30599	-0.043497	0.57101	0.44113	-0.88803	0.075571
0.80734	-0.53744	-0.25479	-0.0061955	0.081866	0.09403	0.18753
-0.32248	-0.4721	0.43343	0.30239	-0.047606	0.14233	-0.17006
-0.59262	-0.18474	-0.021867	0.085378	0.53485	0.44237	0.244
0.53009	-1.0631	-1.1243	0.15777	0.17989	0.50434	0.85594
-0.62997	0.67416	0.75085	-0.41	0.25533	0.070924	0.11093
-2.5738	3.739	3.7681	-0.82966	-1.0532	-0.88302	-0.90719
0.032436	-0.096604	-0.15262	-0.067204	-0.64684	-0.53726	-0.7679
0.3405	-0.18509	-0.14155	0.4305	0.25259	0.013054	-0.12322
-0.75968	-0.25392	0.1979	-0.40902	-0.60395	-2.5221	-1.1209
2.5173	-1.2711	-1.3177	1.5595	1.6365	1.9883	2.322
0.41235	0.63633	0.62347	-0.30085	-0.23666	1.0958	0.37525
1.2547	-0.83009	-1.2248	0.64062	0.48814	0.41081	0.40855

test_GSE325	test_GSE325	test_GSE390	test_GSE390	test_GSE390	test_GSE390	test_GSE393
REF_GSE325	REF_GSE325	test_GSE390	test_GSE390	test_GSE390	test_GSE390	ref_GSE3937
GSE3250	GSE3250	GSE3905	GSE3905	GSE3905	GSE3905	GSE3937
GEO	GEO	GEO	GEO	GEO	GEO	GEO
cDNA	cDNA	affymetrix	affymetrix	affymetrix	affymetrix	affymetrix
TIME:360,DI	TIME:360,DI	TIME:180	TIME:660	TIME:180,BI	TIME:660,BI	yceP_b1060:
347	348	429	430	433	434	437
0.41416	0.5839	0.3914061	1.16641	0.2947489	0.4638224	-0.011231
1.3111	1.6013	-0.3953662	-0.2416769	-0.4447226	-0.3347394	-0.4180244
1.6807	1.5894	-0.1025215	-1.431834	-0.2700524	-0.5526047	0.00053148
0.60356	0.46176	-0.5791792	-0.6437568	0.05297739	0.4163449	0.01850916
-0.39802	-0.83391	-0.9013032	-0.8292755	0.2050048	-0.1637414	-0.3510028
-0.01814	0.11877	-0.4386162	-1.268814	0.2522444	-1.083675	-0.4496286
-0.81404	-0.81336	-0.9397065	-0.6550135	-0.2612113	-0.1943915	-1.507501
1.0551	1.3106	-0.4504383	0.6477961	0.03649117	0.8337835	1.097764
0.82556	1.1614	-0.112132	-1.004023	-0.6741316	-0.8624307	0.04744342
0.30605	0.57443	-0.0826896	0.02263384	0.3582893	-0.1834638	0.5583797
-0.37265	0.09125	-0.7807268	-0.5885626	-0.3264614	0.07152022	-0.0691034
0.27064	0.82292	-0.2420116	-0.1529842	-0.42989	-0.6345683	-0.3205441
0.66237	0.36783	0.1078736	0.1157308	0.3324859	0.5070392	0.4688305
0.55001	0.57114	-0.9518893	-0.2705912	-1.106151	-0.7851373	-0.3961855
0.44905	0.47426	-0.4396218	-0.0157506	0.2579281	0.2568157	-0.7186585
0.71796	0.70859	-0.0507113	0.3260095	0.3267022	0.2059864	-0.1579682
-0.21194	-0.62735	0.0877117	0.8311054	-0.2676491	0.1854678	-0.5484903
0.079867	0.20861	-1.018164	-1.008302	-1.015376	0.1357452	0.5208369
-0.10741	-0.25213	-0.7571442	-0.0553042	-0.4352842	-0.7933946	0.4177777
1.3909	1.0698	-1.280243	0.1100964	-1.643654	1.07776	0.4215242
-0.076267	0.56296	0.4980475	0.4944812	0.4674576	-0.3451904	-1.474538
0.66943	0.71131	-0.6956797	0.8566049	0.03868091	-0.2738554	0.2892998
0.63829	0.86203	-0.3454853	1.841548	-0.0883851	1.977398	0.1160277
-0.37032	-0.24421	-1.224068	-0.2626272	-0.4383006	-0.0194113	0.5111892
0.74972	0.67828	0.7095787	1.367072	0.4614651	0.4846657	-0.6783536
0.31103	-0.31745	0.7412483	0.2220246	1.061873	0.5464791	-0.6480978
1.4212	1.4548	-0.2545559	0.2290013	-0.4066281	0.07844343	0.595159
-0.80809	-0.70933	-0.809887	-1.309827	-0.5675271	-2.42171	1.392661
0.79828	0.40737	-0.4437433	-0.0436897	0.3075931	0.1123727	3.04673
-0.066506	-0.22768	-1.022947	-0.499902	-1.002813	-0.7406062	2.748062
-0.4611	-0.66826	-1.597379	-0.5907464	-0.8245487	-2.339823	-0.3915615
-0.47791	0.38514	0.4017449	0.9768347	2.510786	2.351645	-0.6207731

0.3015	0.32989	-0.4033855	-1.071563	-0.4444192	-1.246854	-0.0894489
0.59569	0.32479	-0.2945077	0.7498725	-0.1268371	0.4428253	0.7710781
1.1404	1.1594	0.2815289	0.7680543	1.038254	0.8415671	-0.3548454
-0.025509	0.034962	-0.0964953	0.5153005	-0.5607278	-0.940242	0.6826072
-0.60084	-0.52109	-0.1926991	-0.2724554	-0.3508638	1.313622	-0.9741685
-0.21283	-0.27166	-1.187816	-0.6665283	-0.8976762	-0.2738632	0.1418856
0.43177	0.33336	0.1791086	0.6360079	-0.9544748	0.4781594	-0.2544185
0.50965	0.65754	-0.2796453	0.09151415	-0.3447366	0.4009718	-0.7435359
1.2933	0.9716	-1.013051	0.04574244	0.3203681	0.5851352	-0.5997039
1.2558	1.1333	-0.3172792	0.3638687	0.1800404	0.4351988	-0.9623254
0.68773	1.0653	-0.1982314	-0.2043998	-0.5202241	0.1236666	0.6628611
0.86836	1.3673	0.04290595	0.1299444	0.6962669	-0.2788354	-0.0953329
0.71165	-0.21712	0.4303174	0.5753174	0.1016454	0.657389	0.6492508
-0.19916	-0.19319	-0.5727784	-1.020977	0.04304153	-0.2391078	0.09361874
0.54972	0.63422	-0.0855082	0.2641201	-0.0333568	0.2816941	-0.2576302
-0.8103	-0.97382	-2.554589	-2.801826	-2.451489	-2.923497	-0.4081892
0.56238	0.64082	-0.1024798	-0.0066747	-0.1380329	0.6158999	1.402946
0.46471	1.4224	-0.7745944	-0.3962206	-0.3845629	-0.1741108	-0.0292943
1.1963	-0.36909	-1.219848	-2.954268	-1.694303	-2.47192	-0.3162455
1.0385	1.1016	-0.1207406	-0.122437	0.389126	0.38466	-0.1762877
0.42357	0.34894	-0.6352893	0.1686162	-0.2428519	0.3688188	0.07225396
-0.49057	-0.72629	0.6002322	2.014414	1.490751	2.925249	0.04236338
0.058508	0.33362	-0.3228516	-0.0244873	-0.3875691	0.1025256	-0.3972343
0.32149	0.39024	-0.5397574	-0.1865388	-0.5793513	-0.3239926	-0.1816413
-0.63828	-0.54474	-0.6932526	-1.183064	-0.1349473	-1.872626	-0.1277664
2.4907	3.1596	-0.0072714	0.04286193	0.79688	-0.63914	-0.6215551
-1.1941	-1.6037	-0.4523054	-0.4742967	0.7427957	-0.317716	-1.107358
0.60454	0.8544	0.8355632	-0.2789248	-0.3215796	0.1775108	-0.9472056
-0.0050377	0.22891	-1.65654	0.07583159	-1.087916	-0.3107292	-0.3008637
-0.45509	-0.42336	-0.8267163	-1.063261	-0.617518	-1.427508	0.9927466
-2.3141	-1.8267	-0.0010155	-3.615274	-0.8381831	-3.331642	-0.9437986
0.8615	0.66561	-0.2465814	-0.5415795	-1.441168	-1.287791	0.2210723
-0.015698	0.014518	0.2960101	0.2742089	0.7138424	-0.1879049	1.593957
0.13257	-0.47258	-0.3939608	-1.726447	-0.4605794	-1.85781	-1.51147
-1.3722	-1.5144	-1.696549	-1.525779	-1.211408	-0.6304884	-0.760778
-0.022074	0.013628	0.3314416	0.3967255	0.2385344	0.1718667	-0.1060783
-0.13607	-0.0079464	-1.156871	-0.3498604	-0.585179	-0.7721332	1.588143
-0.10119	0.19515	-0.4910661	0.292428	-0.6358559	-1.682024	0.0599807
0.47749	0.53555	-0.322836	-0.0819913	-1.181665	-0.5294884	1.292704
0.20425	0.20783	-0.1521977	-1.341375	0.07146307	-1.875447	0.03362705
0.11185	0.33211	-0.1186134	0.2168665	0.2521811	0.4879768	0.8046487
-0.73919	-0.34103	-0.5297175	-0.4067371	-0.5496171	-0.5063605	0.8547938

-1.0423	-1.0849	-0.9656768	-1.222571	-0.7727642	-1.409913	0.4692928
-0.58819	-0.7382	-0.1597387	-1.720038	-0.7037514	-0.4337036	2.436617
0.024158	-0.068546	0.633794	0.8900296	0.189973	-0.5518192	-0.8430856
0.21654	-0.18179	-0.4765616	1.054711	-0.1607053	2.330791	0.4673051
0.73258	0.80733	-0.445994	0.3113375	-0.0059722	-1.844081	0.2725049
0.23668	0.70304	-0.2411964	1.042117	-0.2142028	0.6575344	1.886569
0.31031	0.3311	-0.6575864	0.1056376	-0.5805102	-0.6198735	0.01574747
0.18111	0.46842	0.4245196	0.1508509	0.3483062	0.03281681	-0.0941809
0.40371	0.42631	-2.051518	-1.56715	-0.9731252	-1.535768	0.6290537
-0.15876	0.089675	0.406049	0.1567661	0.2957278	-0.2767885	0.08826892
1.0177	0.85212	-0.4619855	-0.2586315	-0.7159374	-0.7987315	-0.1568312
0.13366	0.10514	-0.3953961	0.08588042	-0.191297	0.5277273	-0.147036
0.98423	1.2575	-0.1571572	-0.4683704	-0.2027277	-0.106607	-0.0539245
0.56081	0.81985	-0.4948083	-1.38976	0.106457	0.2842697	0.4000124
0.32546	0.45106	-0.9918441	-1.310787	-1.987105	-1.601175	0.6376167
-0.87717	-0.47125	-2.221453	-2.491789	-2.659829	-2.182548	0.04912096
0.084599	0.59638	-0.5466355	0.00722749	-1.494838	-0.670519	0.1086773
0.77895	0.84592	0.09264093	-0.0052037	-0.8068537	-0.0263684	-0.8786851
0.33164	0.39026	-0.5422731	-0.5042451	-1.578268	-0.1601333	1.599734
0.075399	0.29999	0.477504	0.5950812	0.2999492	1.756461	0.3075851
-0.59792	-0.99232	0.7827229	1.305186	0.222189	0.7627432	0.4914088
0.68959	0.71598	0.2215437	0.4472795	1.011838	1.465098	0.9436126
0.33161	0.22837	-0.3458959	-1.414428	0.1675803	-0.3462444	0.5391009
1.3026	1.529	0.1604824	-0.8793182	0.07245722	-1.146557	-0.0203778
1.4546	1.6719	-0.0939306	0.23427	0.1562866	0.9347439	0.4711386
-0.85256	-0.49462	-0.9100492	-1.151369	-1.170552	-0.9480178	-0.4082216
-0.003436	-0.06432	-0.9125329	-0.8096396	-0.9124533	-1.074155	1.178962
-0.35578	0.11397	-0.642654	-1.620543	-0.7317834	-1.050934	0.6302247
-0.20937	-0.56481	-0.4620491	-3.25198	-1.58142	-3.559951	-0.2466992
-0.4212	-0.43153	0.1611644	-4.32807	-0.8697339	-5.006966	-1.806841
-0.75947	-0.68519	-0.9747156	-4.572092	-3.004309	-3.823307	0.8887664
0.01063	0.084482	0.2350126	1.371048	1.279728	1.470786	1.375586
0.91701	0.82758	0.9051838	1.305186	1.009673	1.576602	0.7444506
1.0216	1.7252	0.1779151	0.7980537	0.8004724	1.322421	-1.227014
-0.36351	0.092642	-0.4759797	-0.9784139	-0.0679472	-0.1831506	-0.0957947
0.70167	0.61653	-0.7397932	-1.577422	-0.2599859	-2.497294	-0.0554658
1.6812	1.6844	0.5601938	0.2001055	0.3912674	0.5076331	0.8330812
1.1733	1.5966	-0.5415315	0.5529166	-0.266325	-0.6723968	-0.1923333
0.83469	0.85547	0.4057976	-0.821168	0.0363381	-0.3714257	0.4051212
1.1022	0.82887	0.7441277	1.093679	0.04606664	0.1494701	0.5678517
-0.57178	-0.57481	-2.582948	-3.13977	-2.434935	-2.965532	1.196154
-0.23902	0.2794	0.2176985	-0.6523516	0.1680312	-0.7077192	1.478749

0.12355	0.26305	-0.6943116	0.2267569	0.4420688	-0.1776786	0.4275145
-0.23907	-0.14287	0.1261883	-0.0700638	0.4311751	-0.2919653	0.06353543
1.0927	1.1883	0.3806137	0.6853335	0.6880797	1.381491	-0.0386373
-0.36646	-0.081827	-0.0876349	0.484419	0.6248059	-2.008725	-0.2516604
-0.41699	-0.3394	-0.4943127	-0.3719797	-0.4911138	-0.751853	1.473478
0.17331	0.695	-0.5435672	-0.3132208	-0.2765825	-0.5926515	0.1580213
1.1292	1.2942	0.5526312	0.8090364	0.5118043	0.7824108	0.6805912
0.82336	0.63226	-0.0185553	-0.8400683	0.3488053	0.2376406	-1.249147
0.071703	-0.11834	-0.4485235	-0.1690745	-0.2983187	-0.4223264	0.7249455
0.47555	0.64568	-0.0963457	0.2905918	-0.1763305	0.3498377	0.3586264
-0.3252	0.17604	-0.9812351	-1.189445	-0.5467919	-1.248075	0.594431
1.2725	1.4218	-0.7581001	-0.2738983	-0.296751	-0.4282968	-0.3343935
0.013391	0.73338	0.00185856	-0.5816069	-0.4012118	-0.5920908	-0.8251712
0.2544	0.35152	-0.1694967	-0.8529432	-0.276426	-1.083897	0.4460507
0.035666	0.50948	-0.6691796	-1.018287	-0.6498968	-1.1867	0.9384828
0.24291	0.97712	-0.0663098	-0.4358735	-0.171814	-0.5919449	0.3735779
0.095719	0.22795	-0.478727	-0.4312466	0.0574441	-0.3919739	3.85183
0.59276	0.73685	0.2311687	0.3262581	0.1303623	0.5363649	0.9323108
-0.25884	-1.0832	-0.9419912	-1.738218	-1.006521	-1.491132	1.531906
-0.26674	-0.19874	0.5944965	1.67747	0.636813	1.844112	0.503545
0.72495	0.7391	-0.5932272	-0.8809422	-0.7016629	-0.6385054	0.2046014
0.80511	1.2003	0.5751389	1.240149	0.4171217	1.360989	1.34163
NaN	NaN	NaN	NaN	NaN	NaN	NaN
0.47355	1.4893	0.5511278	0.1222294	1.175452	0.2786723	2.00728
0.29677	0.012677	1.581309	0.4523246	0.4251581	0.7871068	0.00967426
0.74119	0.18307	-0.4969824	-0.9108495	-0.8535876	-1.349222	0.940648
-0.12654	-0.30632	-0.2232041	-0.9396406	-0.0312393	-0.5340327	0.7871596
0.022337	-0.54532	-1.411498	-2.168042	-1.477598	-2.162891	0.3891862
1.2368	0.98037	-0.3391805	-1.387266	-0.8003636	-2.510622	-0.6273584
0.84036	1.4398	-1.673793	-0.0593044	-0.6776	-0.3821347	1.112317
0.44093	0.54334	-0.1763661	-1.429705	-0.0430661	-0.6456184	0.5054138
0.11673	-0.084891	1.322065	0.5725294	0.7740056	0.2537838	-0.2953385
0.39571	0.22281	0.1325746	0.05991719	0.315012	-0.1261391	0.2878552
0.39872	0.41014	-0.0495097	0.5455332	0.1623306	0.5792651	-0.0687902
-0.13064	-0.61183	-0.1050415	-0.220016	-0.2351729	-0.3613597	0.1585831
0.048405	0.23456	0.5541023	1.161938	0.8674711	1.104002	-0.571445
-0.18725	-0.00514	-0.3658179	-0.6981074	-0.3412616	-0.7962146	0.6308991
-0.053671	0.12309	0.4563246	-0.6406996	0.6557461	0.4403715	-0.8105754
0.56094	0.73167	0.9941057	0.9727008	1.517906	0.5059787	-0.6003877
-0.098255	0.89687	-0.9298975	0.4861223	-0.6547509	-0.5868986	0.2055825
-0.20358	-0.2403	0.8033206	0.5292529	0.138648	0.3684414	0.2677037
-0.91628	-0.61263	-0.8173341	-1.864032	-0.7037574	-1.575008	0.3274639

1.9174	2.287	0.6556438	0.4689796	0.09761954	0.1061714	-0.7364827
-0.83634	-0.80751	0.1132611	0.1992025	0.05074579	0.5893463	0.4385577
1.0431	1.4658	0.6639144	0.4062396	0.2048168	0.7526108	0.5697854
-0.062812	0.26686	0.1353648	0.3247037	-0.0122294	-0.0346093	-0.2980622
0.12874	-0.44739	0.6300291	0.5470423	0.5965721	-0.5419573	0.9737401
1.0747	0.6332	0.6292307	0.5139103	0.6612492	1.155548	0.1245262
0.58389	0.82404	1.181009	0.8812266	1.072689	0.8448639	-0.4393067
0.55638	0.67074	0.2822451	1.07276	-0.0643985	0.9932668	-0.4803854
1.9267	2.2601	-0.3505928	0.3938029	-1.372921	-1.295718	0.3825168
0.36355	0.3315	-0.6947509	0.2428574	-0.4922994	-0.3636589	0.4002606
-0.21802	0.1655	0.9773755	0.1970508	0.4830278	0.2963843	0.220337
0.71922	1.9763	0.1738766	-0.005949	-0.6025517	0.01723646	0.418103
0.24478	0.19728	-0.5741778	-0.2681958	0.01257507	0.5370075	0.2497099
0.035397	0.43824	-1.474692	-1.065256	-0.8582395	-1.122017	0.4134971
0.68111	1.0003	0.3252351	0.367793	0.1965586	0.2543875	0.05459458
0.23284	-0.65725	0.07801945	0.2600127	0.2865062	0.1530098	2.131134
-0.065874	0.03642	-0.740679	0.03699916	-0.3076956	-0.7227542	0.02346255
-1.8582	-1.3648	-1.345591	-1.053773	-1.313498	-1.855019	0.3662128
-0.52995	-0.78603	-0.371064	-0.8385063	-0.3452672	-0.9196985	0.6297527
0.5491	0.69181	0.5837062	-1.552064	0.8897232	-0.7283077	-0.1213919
0.7897	1.1396	0.2595739	-0.3824739	0.5327314	-0.4071091	0.3870946
0.41143	0.55822	0.2835262	1.206423	0.5706535	0.6294656	-0.0367512
-0.35278	-0.050412	0.1760882	-0.80687	-0.9993982	-2.133244	-0.2236798
-0.40943	-0.119	0.03124471	0.3873023	-0.0892994	-0.8176882	-0.6663222
0.76	0.84437	0.588925	0.8786364	-0.0131409	1.2529	-0.2376086
1.3638	1.4565	0.2112417	0.3106948	0.5102282	0.6517513	0.3731276
0.21222	0.70573	0.7193139	0.5907054	0.1201543	-0.0711989	1.317189
0.88532	1.0355	-1.054312	-0.6577566	-0.2291271	-1.385701	0.2426121
-0.16519	-0.044479	0.06925897	0.6936867	0.4268726	1.115878	-0.1636418
0.87047	0.80136	0.8380965	0.5109503	0.3422707	0.03061185	0.7864723
-1.3377	-1.1294	-0.7840595	-0.6691492	-0.6443133	-1.042956	-0.3347282
-0.13043	-0.40524	-0.2818415	-0.0692842	-0.2463607	-0.6860703	-0.7525661
0.34242	0.3066	-0.0283615	-0.1427684	-0.2870929	0.03551834	-0.6216988
-0.32764	-0.46917	-0.4103624	-0.1239147	0.2639938	0.5577101	-0.4803539
-0.17639	-0.043263	-0.0128345	-0.1067076	-0.3815459	-1.166317	-1.424667
-0.049351	-0.1668	-0.3384685	-0.2404044	-0.1736956	-0.7581679	-0.5944504
0.47397	0.97192	-0.0025177	0.3996891	0.1642865	0.4540408	0.6794726
0.38635	0.32821	0.4428657	0.09868727	0.1645235	0.1701499	-0.2039737
1.2774	1.1728	0.5645367	-0.3043385	-0.057824	0.00359756	-0.5400162
0.072363	0.28582	0.3939129	1.230377	1.042308	1.367243	-0.1898669
1.7807	1.4864	-0.6586288	-0.1666078	-0.9200354	-0.4808901	2.825843
1.3154	1.4578	-0.2662835	-0.0543714	-0.2487222	0.00757923	2.013279

0.064901	-0.18411	1.021968	1.882562	0.8596313	1.057326	0.140841
0.0038674	0.49874	0.348459	0.4939691	-0.5404949	-0.9325114	-0.1464389
0.093695	-0.019513	-0.0146691	0.369413	0.3501969	0.1435105	-0.0113963
0.37772	0.28129	0.7935369	-0.2591694	0.1837486	-0.4309161	0.04282875
0.69512	0.25882	-0.0543824	0.150007	-0.8631012	-0.2973884	-0.1975757
0.53629	0.82126	-0.0915951	-0.9518645	-0.474868	-1.45526	0.2983943
0.49823	0.26828	-1.260413	-0.5863703	-0.7181082	-0.6925232	-0.2175321
-0.27534	-0.27528	0.5891189	-0.0305888	0.1033431	0.1642519	-0.1234759
-0.58417	-0.46062	0.4635335	0.8123527	-0.2008295	0.7046096	-0.2825157
-0.4776	-0.31392	-0.0775586	-1.09027	-0.2959424	-0.7153501	0.5183
0.34545	0.22974	0.5187286	0.3842403	0.3245353	0.6702933	1.035178
1.0889	1.3495	-0.1760085	-1.516128	-1.191523	-1.428768	0.3554222
-0.17155	0.020114	0.00725468	-0.8214173	0.04747499	-0.3863708	1.89352
0.99655	1.2323	-0.0861742	-0.041125	-0.2421841	-0.7715122	0.01801226
-1.2195	-1.1148	-0.0566486	-0.9637878	-0.1669137	-0.7083352	0.165527
-0.06616	-0.56178	0.7317494	1.165125	1.062759	1.07065	-1.062844
1.3462	1.6317	-0.2053132	0.3266096	-0.7986476	0.2568649	0.5710897
0.089424	0.046752	-0.3139411	-1.421261	-0.5429227	-1.822731	-0.1522872
1.171	1.3632	-0.0704941	0.5729312	-0.1843003	0.460291	0.3483217
0.48646	0.51631	0.3105127	0.8320668	0.4002836	0.3333008	-0.5735142
-0.12846	-0.30151	-0.1561335	-1.027388	-0.691689	-1.217178	-0.1825098
1.0072	1.3731	0.526983	0.3876765	0.1058193	-0.440751	-0.4123049
0.35808	0.065492	0.0114614	-0.0565913	0.07609368	-0.1730363	-0.4247935
0.070639	-0.15076	-0.6063018	0.6772817	0.2706936	0.555027	0.6262144
0.42938	0.56813	0.5612563	-0.3071342	0.1683482	0.1110215	0.01388949
-1.4201	-0.84895	-0.7177618	-0.0238837	-0.4106072	-1.057692	0.3257676
-1.5659	-1.3283	-2.132397	-1.507403	-1.701494	-0.8897217	0.7570141
-0.65358	-0.73732	-1.154262	-0.0028469	-0.8337012	-0.5251345	1.641481
0.55047	0.69419	-1.486279	-1.622761	-1.471793	-1.663377	1.702023
0.67771	0.87225	-0.8289576	-0.0089172	-0.7819661	-0.2880956	0.766155
0.29243	0.48647	0.2096538	-0.1127992	0.3663835	1.316576	0.796202
-0.040286	-0.08948	0.8245982	0.1492323	0.2480064	0.05697323	0.3366755
1.2585	1.4449	0.4151766	1.71041	0.5475869	1.776522	0.08075239
-2.6372	-3.091	-0.2575519	-2.710924	-1.081687	-2.509351	-0.9158473
1.0842	0.9228	-0.1814203	-0.342619	-0.3466416	-0.9757964	-0.0665874
-0.18402	-0.54869	0.1612076	-0.7736119	-0.0160125	-0.2048937	0.7462344
0.22443	0.20514	0.4112428	0.076894	0.516943	1.297467	1.268338
0.33975	0.61557	-0.5550289	0.3586641	-1.340175	-0.7061922	0.3766588
0.94389	0.79082	0.06706269	1.085054	0.9003782	1.111106	-0.3328999
-0.65553	-1.4306	-0.8204112	-1.07753	-0.6645981	-0.505445	0.5730541
-0.11419	-0.048512	0.4368813	0.3963027	-0.0978591	0.3589775	-0.9000094
-0.16651	-0.037405	-0.3508613	0.5058246	-0.1527615	-0.2813706	0.7098482

-0.11846	-0.21151	0.2043875	0.6706037	0.7593628	-0.1116104	0.5320643
0.11413	0.11117	0.7049777	-0.5454031	0.4961793	-0.2342665	-0.6719811
0.98615	0.9904	0.6920963	0.07340853	0.3299253	0.298627	-0.8307763
0.85753	0.53086	0.07370084	0.1134559	-0.5254598	-0.7816187	0.3892916
0.16867	0.37192	-0.3224961	-0.5744188	-0.7034085	-0.3839163	1.073529
0.43152	0.56646	-0.2515977	0.6544259	0.07069872	0.2884001	0.00745681
-0.14261	-0.25763	0.4760618	-0.2896717	-0.2108561	-0.7543632	0.1574203
-0.26348	-0.17427	-0.6255457	-0.8203852	-0.1916905	-0.4985684	-0.1229848
0.22613	0.11031	1.111968	0.09367339	1.001057	1.510698	0.8327835
-0.5409	-0.54349	-1.63408	-1.747557	-2.143109	-1.595333	0.1987068
-0.1544	-0.29516	0.2816138	-0.6122736	0.3200934	0.1849058	0.8223392
-0.4179	-0.27903	-0.1713642	-0.9424889	0.05239124	0.2662247	-0.5049272
1.4886	0.72401	-0.5365366	-0.4080858	-0.69254	-0.6905683	-0.1814176
0.42787	0.69397	0.6534426	1.333012	-0.7756025	0.4589524	0.2192167
0.33868	0.22569	-0.5457965	0.5051478	-0.0926333	0.4285436	0.3018768
0.57001	0.55045	0.06215554	0.6065922	-0.1546724	0.1728164	0.5318492
0.78453	0.81672	0.3275894	0.2017135	0.1284491	0.4850223	0.1827635
-0.16573	-0.16203	0.4715655	-0.8014004	0.4740433	-0.9721095	-0.515885
-0.71511	-0.46844	-0.9474655	-0.7363354	-0.8513444	-1.558164	-0.5837462
1.3601	1.2776	-0.0676477	0.03474576	-0.1516232	-0.6605533	0.7555622
-0.027147	0.04381	0.3634825	-0.4239196	0.1224217	-0.6238425	-0.6550203
0.22248	0.46217	0.2729511	-0.0145888	0.00405699	-0.2764384	0.06490923
-0.64975	-0.51775	0.435485	-0.3046174	-0.1016458	-1.131721	0.6890891
-0.87758	-0.8294	-1.818185	-1.506071	-1.857185	-1.686414	-0.3698015
0.25551	-0.19086	0.8794418	0.658754	0.2298049	0.6725998	-0.8237491
-0.063029	-0.021392	0.3081737	-2.51327	-0.9111607	-3.798634	0.8107452
1.7486	1.5755	-0.1439157	-1.174104	-0.8977728	-0.7656061	-0.2115725
0.2962	0.32424	0.3989153	-1.043351	-0.4654329	-0.9070327	0.4097171
0.99925	0.85854	-3.707777	-3.837173	-6.923947	-5.65893	0.3215027
1.5046	1.9854	0.5874806	0.2177372	-0.475735	0.9238198	0.1781105
0.87215	0.56512	-0.4349565	-1.579951	-1.554263	-2.597033	0.3043778
0.040081	0.28855	-0.9807105	-0.2725768	-1.055979	-1.292776	0.2492109
-0.23446	-0.44848	0.2096422	0.8298829	0.4479787	0.5394449	0.5116532
0.52575	0.57231	-0.7746856	-1.078719	-0.4641086	-1.064579	-0.5593426
0.60851	0.86317	1.100469	-0.9732615	0.3892071	-0.9604912	-0.6805342
0.41571	0.46172	-0.4415365	-0.5372343	-0.5936838	-0.8563101	-1.1396
0.0026691	0.10796	-0.8687741	-0.6476816	-2.18141	0.3895778	-0.2384688
1.1777	1.5459	-0.3330972	0.632535	-0.6033342	0.00945724	-0.0214342
-0.66424	-0.98687	-0.9815759	-0.6271399	-0.4046203	-0.9307803	0.9519113
-0.20689	-0.33846	1.067395	0.759942	1.093526	0.7941072	-0.0210671
0.40136	0.53778	0.4612122	0.4156988	0.193721	0.344428	0.234995
-0.01056	0.16957	0.7362163	0.371126	0.02237117	-0.5900025	-0.3047075

-0.22291	-0.18929	0.1371047	-0.4817291	0.6599454	0.4376509	-0.4452662
-1.2764	-0.8289	0.04524004	0.1891928	-0.1361186	-0.0609268	-0.4574437
0.41048	0.25355	-1.466517	-1.204632	-1.329372	-0.9918527	-0.082432
-0.79793	-0.81369	0.07169217	0.2271778	0.05549425	0.267773	0.1525768
-0.15532	0.67195	-0.0360283	-0.9090622	-0.4479308	-0.9120813	-0.4181315
-0.57232	-0.84003	0.1413298	-1.405489	0.02098919	-0.900784	0.4511864
0.39862	-0.024695	-0.1200546	-0.4789198	0.05895813	-0.3121842	0.2497597
0.41334	0.81321	-0.6009077	0.4596077	-1.071759	0.4313641	0.2816415
0.35088	0.77526	0.4222647	-0.5494944	0.2210232	0.1518133	0.4628032
0.90082	0.32366	-0.5658874	-1.628698	-0.6734767	-1.552363	0.00218913
0.059882	0.035413	-0.6236776	-0.0105779	-0.9314368	-0.2726007	-0.1471315
0.95203	0.97371	0.3077292	-0.4058684	-0.317123	-0.7297898	-0.2907811
-0.039308	-0.29631	0.09287992	0.578383	0.1540049	0.7941373	0.4267309
-0.95884	-1.1179	0.9770202	0.7286846	-0.1037856	0.6431244	0.6085218
0.029262	-0.55895	-0.7032195	0.2894525	0.6426251	-0.1373411	-0.4893334
0.10974	-0.14119	0.4896642	0.442383	0.4331807	0.4383747	-0.4843814
-0.49863	-0.75198	-1.020939	-0.4370304	-0.6185544	-0.5518749	-0.3155404
2.6534	2.7034	0.415228	0.06135135	0.2356814	-0.2365898	-0.1316446
0.80426	0.78582	0.1203153	-0.3602091	-0.0504596	0.2465594	-0.3955822
0.81789	1.0175	-0.5315797	-0.1603628	-0.1558542	-0.1483971	0.4771666

test_GSE432	test_GSE432	test_GSE434	test_GSE434	test_GSE434	test_GSE434	test_GSE434
REF_GSE432	REF_GSE432	ref_GSE4344	ref_GSE4344	ref_GSE4344	ref_GSE4344	ref_GSE4344
GSE4321	GSE4321	GSE4344	GSE4344	GSE4344	GSE4344	GSE4344
GEO	GEO	GEO	GEO	GEO	GEO	GEO
cDNA	cDNA	cDNA	cDNA	cDNA	cDNA	cDNA
rpoH_b3461	rpoH_b3461	RIFAMPICIN:	RIFAMPICIN:	RIFAMPICIN:	RIFAMPICIN:	RIFAMPICIN:
443	453	462	463	464	465	467
0.15605	0.11412	-0.013682	-0.35211	-0.12531	-0.553	-0.13437
0.033156	-0.17233	-0.01513	0.2572	-0.15436	0.0068596	0.054008
0.23273	0.18541	0.055473	-0.014761	0.030328	-0.11478	0.10081
-0.0074091	0.1479	0.2865	0.26348	0.13604	0.37179	0.34882
-0.02507	0.03715	-0.056383	-0.0013406	-0.088336	-0.18686	-0.027809
0.020479	-0.1392	-0.024975	0.14433	0.66818	1.0294	-0.051308
0.24947	-0.18443	-0.19121	0.13095	-1.7019	-1.7877	-0.032918
0.34857	0.32369	0.13884	-0.68916	-0.61427	-0.46497	-0.47625
0.2105	-0.22656	0.15084	0.25835	-0.03525	0.14987	0.29989
0.022744	0.17679	-0.20325	-0.26871	0.11471	0.031556	-0.016271
0.098624	0.27016	0.020078	-0.50752	-0.46479	-0.53979	-0.24187
0.29969	0.81049	-0.70865	-0.96749	-0.61696	-0.6819	-0.79739
0.2035	-0.065114	-0.31114	0.052213	-0.24989	0.016945	-0.10876
0.17038	0.11046	-0.25065	0.090068	0.12678	0.25407	0.21388
0.3707	0.24729	0.014931	0.072044	0.17253	0.015811	-0.052134
-0.075456	0.076605	0.010063	-0.085391	-0.10983	0.10275	-0.093921
-0.069174	-0.004224	-0.25924	-0.16869	-0.87553	-1.0821	-0.21774
0.063118	0.02903	0.10712	0.27598	-0.036952	-0.12649	0.18974
0.059677	0.18072	0.19852	0.11715	0.41604	0.50491	0.50351
0.053563	0.111	0.023174	0.28169	0.71945	0.89845	0.22117
0.18558	0.16808	-0.1097	0.035711	0.44365	0.37734	0.046339
0.5273	1.124	-0.36482	-0.35568	-0.30875	-0.088986	-0.07381
-0.050364	-0.28016	-0.1664	0.042135	0.14474	0.2774	-0.05102
-0.049215	0.082438	-0.13819	-0.065295	-0.52341	-0.3443	-0.30741
0.25086	0.2612	-0.066503	-0.17404	0.27442	0.0058217	0.026584
-0.029863	-0.045699	-0.38721	-0.51312	-0.51511	-0.60859	0.036384
0.46199	0.30272	0.48185	0.02167	-0.058976	-0.035106	-0.75745
-0.059352	0.34256	-0.055937	0.048136	-0.59547	-0.29449	-0.43681
-0.085372	0.0093814	-0.06463	0.18702	0.15921	0.15519	0.19645
0.54769	0.67505	-0.3633	-0.44091	-0.22983	-0.70393	-0.34495
-0.037046	-0.16578	-0.44157	-0.14954	-0.39165	-0.48378	-0.55275
-0.084422	0.16768	-0.74502	-1.2467	-0.44646	-0.71899	-0.96667

0.17106	0.11799	0.30878	-0.30055	0.14259	0.35434	0.00038096
0.08663	0.17251	-0.0088847	0.27749	0.79184	0.85621	0.30164
0.53064	-0.041905	-0.48642	-0.44651	-0.32487	-0.027869	-0.22604
0.14438	0.040235	0.052728	0.131	0.31972	0.14187	0.10023
-0.070442	-0.070101	0.036668	0.033754	-0.02227	0.22959	0.13533
0.21035	0.010489	0.23989	0.23836	0.38953	0.55965	0.25742
-0.1588	-0.0062143	-0.46502	-0.72713	0.12091	0.066843	-0.52194
0.076001	-0.0070569	-0.21572	0.15717	0.26424	0.55291	-0.082458
0.043707	-0.0055957	-0.012105	-0.032059	-0.19231	-0.020003	-0.10779
0.081295	0.064267	0.1381	0.10646	0.14654	0.054322	-0.032562
0.19312	0.1742	0.058353	0.19916	0.047735	0.0047165	-0.018668
-0.39987	0.054651	-0.052315	0.051244	0.089893	0.3108	-0.075497
0.18155	-0.28716	-0.034945	0.15598	-0.0007631	0.12218	0.088846
-0.0099022	0.095284	-0.098198	-0.67598	-1.0156	-1.4818	-0.075735
-0.15465	-0.021305	-0.23556	-0.99105	-0.91016	-1.1557	-0.72727
-0.064825	-0.18132	-0.22882	0.23437	0.18121	0.066913	0.19082
0.0078999	-0.10598	0.082075	0.0743	0.40093	0.22368	0.16406
0.02433	-0.019932	0.16024	0.14897	0.18377	0.57582	0.40292
0.070338	0.04412	-0.28835	-0.4955	-0.58229	-0.91342	-0.33963
-0.098288	-0.307	-0.0051892	-0.0033573	0.064908	0.19517	-0.28087
0.014726	0.21656	-0.21062	-0.098551	0.092061	0.074475	0.0003131
0.20153	0.39581	-0.40165	0.0053047	-0.079572	-0.32862	0.10371
-0.076218	-0.031205	-0.13101	-0.32194	-0.14112	-0.33099	-0.15109
0.50976	0.40825	-0.13184	-0.3482	-0.092863	0.054303	-0.045407
-0.25275	-0.064335	-0.13943	-0.14093	-0.22867	-0.29024	-0.10645
0.24525	0.36066	-0.091577	0.036749	-0.25954	-0.6854	-0.35513
0.043191	0.0022412	-0.16866	-0.088673	0.26063	0.15612	-0.090098
0.19533	0.17736	-0.32406	0.095571	0.065545	0.0028836	-0.15795
-0.046289	0.082695	0.01885	-0.23558	-0.19741	-0.078131	-0.15503
0.032058	-0.020301	0.16333	-0.081133	0.0023528	-0.27044	-0.1347
0.028921	-0.15122	0.15094	0.24395	0.28623	0.095803	-0.11065
-0.11744	0.093561	-0.32865	-0.043858	-0.43854	-0.3009	-0.11261
0.17424	0.13149	-0.084187	-0.01736	-0.046481	-0.57659	-0.049333
0.069685	-0.04798	-0.0183	-0.16435	-0.43254	-1.0988	-0.010087
0.081708	-0.0020075	0.14671	-0.3231	0.22357	0.14707	-0.20397
0.12797	0.07748	-0.19685	-0.37221	-0.27553	-0.31264	-0.27402
1.3318	1.4054	-0.34944	-0.4651	-0.14768	-0.20216	-0.42667
-0.089074	0.44572	0.017866	0.20735	0.29605	0.022265	0.044532
-0.024049	0.092365	-0.83026	-1.4748	-1.3887	-1.9419	-1.3813
0.070074	-0.062229	-0.53343	-0.92288	-0.66821	-1.0152	-0.46908
0.10173	0.13979	0.018318	-0.64498	-1.1541	-0.45907	-0.23973
0.20458	0.14722	0.47735	0.31548	0.081509	0.37697	0.15476

-0.26868	0.77434	-0.15528	-0.53976	0.088659	0.41794	-0.26206
-1.232	-0.90726	-0.37515	-0.94026	-0.84523	-1.2713	-0.4302
-0.036126	-0.011958	0.093028	-0.88992	-0.21498	-0.70699	-0.40369
0.034158	-0.059853	0.13709	-0.03691	-0.0017461	-0.1448	0.068429
-0.10198	-0.066718	0.0032052	0.29781	-0.029646	-0.05465	0.02064
0.36945	0.14838	0.16529	-0.041948	0.38307	0.7226	0.11733
0.29697	0.20111	0.076896	0.18908	0.4475	0.69856	0.33335
0.00064567	-0.033002	-0.41301	-0.54714	-0.45015	-0.76268	-0.14674
0.15102	0.15394	-0.31881	-0.26377	-0.18756	-0.037084	-0.36151
0.12627	0.15935	-0.13983	0.056911	0.23865	0.25291	0.16928
0.052237	0.14342	-0.09685	-0.093937	0.17276	0.27103	-0.0058555
0.014673	0.044626	-0.33076	-0.40008	0.14102	0.028519	-0.23245
-0.068835	0.075613	-0.2656	-0.4736	-0.58995	-0.47197	-0.29756
-0.042878	-0.001702	-0.28546	0.029366	0.065518	-0.0023808	-0.048336
0.044311	0.09957	-0.21368	-1.0276	-0.45584	-0.72882	-0.31274
-0.027128	-0.1353	-0.33032	-0.10298	-0.0094784	0.070366	0.0076933
-0.093384	0.037902	-0.08545	-0.087806	0.078719	-0.0073427	-0.065176
0.23982	-0.016428	-0.069813	-0.1331	-0.46125	-0.50624	-0.031491
0.26876	0.14249	-0.1118	-0.0054062	0.28601	-0.012358	-0.022213
0.0972	0.089703	0.058067	-0.024574	0.093762	-0.019484	0.053767
0.0094851	0.062519	-0.12689	-0.037598	-0.0040054	-0.29103	0.0032283
-0.07036	0.035553	-0.17187	-0.20105	-0.43183	-0.48046	0.038907
-0.020253	-0.084068	-0.056927	0.069398	0.22042	0.24886	0.079554
-0.089582	0.057431	-0.50505	0.15698	0.12393	-0.0044115	-0.054895
-0.097203	0.22039	-0.12343	-0.048934	-0.27105	-0.074447	-0.099466
0.21272	0.022109	-0.068487	0.060878	-0.27196	-0.12411	-0.033669
0.082704	0.028077	0.015737	-0.0094832	0.16914	0.21953	0.062782
-0.15704	0.30644	-0.70231	-0.46248	-0.20505	-0.39667	-0.57949
-0.009699	-0.1115	-0.11529	-0.085607	-0.0536	-0.037822	-0.013512
-0.34018	0.018136	-0.071563	-0.11086	-0.18327	-0.0081172	-0.0057583
0.057482	0.082187	0.15972	0.021929	-0.048432	0.17491	-0.024749
0.11985	0.26217	0.16039	0.15881	0.16761	0.56304	0.1261
0.074048	0.098231	0.17456	0.31516	0.6929	0.91565	0.31803
0.0047592	0.13659	-0.019242	0.22957	0.22028	0.17543	0.10509
0.33608	0.35053	-0.14674	-2.0381	-1.6711	-1.618	-1.3545
0.28162	0.30888	-0.41245	-0.26562	-0.24923	-0.2131	-0.41361
0.0064909	-0.33347	-0.056085	-0.15146	0.077814	0.086375	0.12349
1.0938	1.3431	-0.51161	-0.41124	-0.35389	-1.3139	-0.34138
0.34455	0.26406	-0.16211	-0.11318	-0.15947	-0.54129	-0.16137
0.083084	0.036631	0.2689	0.18073	-0.11706	0.095748	0.14676
0.15461	0.16254	-0.074987	0.096794	0.16616	0.34584	0.18077
-0.10368	0.40202	0.43927	-0.33723	-0.45237	-0.27247	-0.10027

-0.080426	0.090459	-0.26934	0.0022856	0.094658	-0.091176	-0.042226
-0.21224	-0.18981	-0.36445	-1.1476	-0.38932	-0.61159	-0.37702
-0.069578	-0.01554	0.0082252	-0.061866	0.20522	0.15926	0.039926
-0.0006925	0.22317	0.055581	-0.3332	-0.21824	-0.1277	-0.020197
0.017949	0.15224	-0.31623	-0.59814	-0.42929	-0.5071	-0.41912
0.01978	0.40586	0.57559	0.29132	0.092514	0.45716	0.21606
0.096684	0.3306	0.022707	-0.32198	0.31124	-0.0067572	-0.0944
-0.090742	0.17515	-0.026079	-0.040624	0.16859	0.050193	-0.089105
-0.019689	0.0043414	0.078043	-0.10876	-0.13314	-0.073763	-0.074991
0.066901	0.10317	-0.0226	-0.31421	-0.67897	-0.031605	-0.13338
0.35595	0.37449	-0.037024	0.091582	-0.43079	-0.44895	0.044714
0.046912	-0.38369	-0.16351	-1.538	-0.63372	-1.0286	-0.61877
-0.090928	0.041631	-0.26717	-0.24649	-0.69898	-0.52281	-0.24581
0.055333	0.18131	-0.19359	-0.11071	-0.48794	-0.42994	-0.25981
0.032021	0.02221	0.059562	-0.003783	0.32732	0.029527	0.059442
0.35409	0.45927	-0.50376	-1.7789	-1.2394	-1.132	-0.78281
0.2193	-0.0076622	-0.15858	-0.49477	0.013941	-0.092678	-0.13757
0.062535	0.19718	-0.11773	0.12689	-0.081037	-0.02685	-0.090364
0.14545	0.081347	-0.46336	-0.38554	-0.18466	-0.42412	-0.82783
-0.91088	-0.52347	0.011136	-0.075591	0.092139	0.059644	-0.06702
0.037955	0.019177	0.43984	0.3441	0.011084	0.23801	0.045063
-0.029354	0.051677	0.26133	-0.58451	-0.13702	-0.070103	-0.068597
NaN	NaN	NaN	NaN	NaN	NaN	NaN
0.089957	0.13199	0.082514	-0.0065609	-0.17854	-0.22074	-0.20544
-0.12468	0.10248	-0.42193	-0.54213	-0.35383	-0.52979	-0.38244
0.062125	-0.066533	0.19533	0.28224	0.10885	0.078431	-0.17496
0.15855	0.086855	0.15761	-0.28585	-0.60777	-0.52957	-0.14419
0.11763	0.035168	-0.040265	-0.084386	-0.020519	-0.15366	-0.50429
-0.039886	-0.036733	0.15157	-0.35659	-0.52179	-0.84403	-0.13736
0.29613	0.13183	-0.84519	-2.09	-0.24949	-0.1376	-1.0664
-0.26072	-0.10304	0.14467	0.38724	0.53387	0.12299	0.22767
0.75542	0.3856	-0.25502	-0.052081	-0.56153	-0.4752	0.074539
-0.082131	-0.095255	-0.17539	-0.029016	0.19957	0.11802	0.33111
-0.12942	0.16671	-0.18938	0.13791	0.11351	-0.073468	0.12095
-0.17409	0.04945	-0.10818	0.072116	-0.10627	-0.23354	0.052457
-0.16041	-0.02189	0.031585	-0.39342	-0.28053	-0.33776	-0.016256
0.17937	0.10157	-0.086497	-0.45922	-0.14439	0.058842	-0.42355
-0.2392	-0.29982	0.17743	0.42546	0.28506	0.57094	0.27272
-0.04028	-0.10293	-0.090304	-0.03755	0.090265	-0.012213	0.14158
-0.075467	0.03043	0.23948	0.63411	0.031126	-0.092401	-0.28633
-0.16896	-0.16534	-0.35511	-0.14881	-0.27211	-0.3654	-0.12246
-0.1094	-0.20129	-0.62465	0.041323	-0.53198	-0.46379	-0.30212

0.040682	-0.021285	0.29791	-0.025753	-0.091153	0.17207	0.21286
-0.058621	-0.053139	-0.088884	0.01863	0.14893	-0.1203	-0.048525
0.15313	0.099459	0.042133	-0.055797	-0.058626	0.067254	0.048429
0.097605	-0.024047	-0.25763	-0.12013	0.049813	0.13352	0.48569
0.01514	0.15047	0.35662	0.27167	-0.20466	-0.40109	0.62804
-0.035669	-0.076793	0.095318	0.35964	0.24264	0.44502	-0.038978
0.29612	0.11773	-0.52378	-0.8311	-0.67106	-0.95348	-0.48148
-0.0006822	0.33003	-1.4831	-2.006	-0.58151	-0.55831	-0.94265
-0.046301	0.12073	0.18153	-0.41309	-1.1082	-0.54118	-0.08417
0.53821	0.17512	-0.14984	-0.108	0.22471	-0.026075	0.26769
0.036823	-0.064073	0.073745	-0.18835	0.20221	-0.1199	-0.044506
-0.027917	-0.032212	0.055572	-0.31504	0.029732	-0.34877	0.0010799
0.035094	-0.25802	-0.20856	-0.008655	0.12274	0.085533	-0.048966
0.14907	0.17026	-0.12414	0.14848	0.0030654	0.068618	0.14227
0.38508	0.18781	-0.13202	0.036435	-0.10995	-0.037512	-0.081535
-0.0091241	0.047135	-0.041441	-0.29759	-0.47512	-0.59652	-0.19535
-0.060037	-0.035468	0.0068315	-0.11054	-0.0024556	-0.070321	0.087138
0.013768	-0.0019208	0.12442	-0.12208	0.084932	0.33041	0.04143
-0.20969	-0.52134	-0.56721	-1.3224	-0.85235	-1.4198	-0.32427
0.086339	-0.023944	0.031661	-0.037938	1.2953	1.9788	-0.11799
0.25721	0.13172	-0.089312	-0.027203	0.087928	0.27726	-0.028663
-0.13292	0.19484	-0.17242	0.23325	0.13031	0.19082	0.066568
-0.075068	-7.82E-05	-0.1628	0.039056	-0.60661	-0.47372	-0.073086
0.054272	0.13689	-0.022813	0.016175	0.14495	-0.12499	-0.011504
0.20017	-0.018898	0.0043246	-0.29919	0.16446	0.047821	-0.059865
-0.14497	-0.091731	-0.29433	-0.01032	-0.25391	-0.3132	-0.12601
0.0627	0.22002	0.2872	0.15319	-0.29439	-0.67568	-0.095738
0.02327	0.27597	0.0079949	-0.11349	-0.3128	-0.17571	-0.043714
-0.077467	0.025848	-1.0344	-0.66657	-1.5575	-1.5206	-0.60002
0.19597	-0.062562	-0.49886	-0.31552	-0.30693	-0.29169	-0.22013
-0.074185	0.035437	0.22919	-0.065672	-0.32081	-0.29235	-0.047449
0.05683	0.12162	0.00036037	0.017866	-0.0011824	0.4609	-0.11354
0.016527	0.07216	0.14266	-0.065854	-0.66558	-0.42484	-0.057337
-0.047855	0.076622	0.27732	0.20836	0.2376	0.2423	0.26958
0.057322	0.017682	-0.28016	-0.064234	0.18687	0.10959	-0.059474
-0.10488	0.19924	-0.72054	-1.4354	-0.83151	-0.63273	-0.72798
0.11739	0.052206	0.2094	0.056751	-0.024557	0.046259	0.14129
0.27892	0.21479	-0.16733	-0.74414	-0.46858	-0.2431	-0.12239
0.072066	0.043634	-0.083456	-0.31814	-0.73309	-0.62453	-0.18487
-0.031473	-0.071732	-0.18313	0.2105	-0.43654	-0.30925	-0.021454
-0.055981	0.2189	-0.65858	-0.28683	0.22738	0.050336	-0.75182
0.12873	0.43497	-0.62732	-1.2023	-0.76253	-0.5828	-1.0128

-0.058606	-0.16172	-0.31546	-0.38017	-0.11576	-0.21154	-0.28769
-0.018563	0.28284	0.21782	0.021446	0.15604	0.03826	0.099255
-0.023528	-0.098248	0.085492	-0.35875	-0.12821	-0.27046	-0.20616
0.17289	-0.046103	-0.0084439	-0.094346	-0.38966	-0.20002	-0.08852
-0.09479	0.0066167	0.036439	-0.26994	-0.2696	-0.35542	-0.15824
0.36184	0.20016	-0.72016	-1.9324	-1.2655	-1.0448	-0.79729
-0.2515	0.0041526	-0.50832	-0.41411	-0.3471	-0.42243	-0.27949
0.099668	0.053523	-0.32234	-0.096836	0.67114	0.37204	0.20211
0.037265	-0.052105	-0.022353	0.025838	0.24597	0.074075	0.12157
0.010092	0.21088	-0.35212	-0.42461	-0.4039	-0.38706	-0.6706
-0.04489	-0.064038	-0.17819	-0.13239	0.30144	0.501	0.12165
0.033716	0.2592	-1.0627	-0.38896	-0.71198	-0.93164	-0.45701
-0.19269	-0.15486	-0.27227	-0.11388	-0.49387	-0.72727	0.028793
0.050016	0.049861	-0.05137	-0.25672	0.27242	-0.03231	-0.14701
-0.47081	-0.65988	0.66522	0.15716	-0.097066	0.48685	0.1087
0.077288	0.38501	-0.05232	0.14799	0.46553	0.36707	0.11901
0.88555	0.48713	-0.37387	-0.25811	0.05704	0.79918	-0.58943
0.09095	0.15498	-0.3721	-0.33349	-0.49162	-0.57493	0.030863
0.046783	-0.048055	-0.72563	-0.23577	-0.7527	-0.5168	-0.40245
0.34502	0.10061	0.028891	0.1579	-0.32114	-0.91774	0.39562
0.11223	0.045764	-0.21385	-0.11856	-0.55823	-0.29587	0.16896
-0.11169	0.074175	0.28953	0.2443	-0.070463	1.4675	-0.39257
0.06734	0.066943	0.33851	-0.0037215	-0.667	-0.96817	0.12779
0.042088	-0.14921	-0.80073	-0.7904	-0.97414	-0.61159	-0.72274
0.13669	0.051314	0.10053	-0.11321	-0.04347	0.020717	-0.067603
-0.018296	0.032464	0.072208	-0.063763	0.21507	0.13848	0.014594
0.20633	0.16698	0.421	-0.35711	-0.080234	-0.13086	-0.12593
0.17842	-0.093646	-0.33298	-0.38013	-0.081177	-0.31588	-0.77784
1.1681	1.0323	-0.59926	-2.2005	-0.36432	-0.039414	-1.1354
0.31071	0.23319	-0.24199	-0.9392	-0.15573	-0.062885	-0.86821
0.020316	0.043049	0.20934	0.14591	-0.18997	0.35971	-0.14247
-0.12851	0.066783	0.13926	0.13515	0.38483	0.29143	0.23062
1.0164	0.89876	0.29196	0.27586	0.15108	0.15465	-0.41565
-0.33438	-0.27526	-0.39435	-0.13497	-0.58851	-0.9012	-0.1462
0.2116	0.18122	0.020416	0.075578	-0.17336	-0.0067853	-0.17536
-0.14056	-0.52998	-0.14934	0.014031	0.19018	0.17539	0.0071713
0.025798	0.14921	-0.4174	0.042358	-1.0025	-1.0587	-0.052541
0.13995	-0.07232	0.14287	0.2675	0.12569	0.17164	0.37078
-0.086198	0.014766	0.54433	0.27355	-0.26902	-0.54749	0.24077
-0.26147	-0.27811	0.091178	0.31682	0.13628	-0.20543	0.37926
0.28792	0.11155	-0.074295	0.23964	0.4315	0.37489	0.19874
0.0066845	0.017812	-0.16387	-0.034703	0.37422	0.67251	0.033919

-0.055908	0.16632	-0.030764	0.018348	0.33878	0.2895	0.078982
0.041983	0.1608	-0.070819	0.084864	0.21274	-0.037866	-0.013127
0.075093	0.0025548	0.05418	-1.474	-1.514	-1.9448	-0.15273
0.12449	0.26908	-0.21465	0.073797	-0.29951	-0.068582	-0.21531
-0.20907	0.084795	-0.25126	-0.14786	0.28239	-0.11835	0.018774
0.16631	0.16024	0.25281	0.33727	0.59796	0.85322	0.17884
0.04045	0.027691	-0.0714	-0.51286	-0.32265	-0.40878	-0.23879
-0.072455	-0.13311	0.00034898	0.038717	-0.20532	0.067877	-0.10228
0.034868	0.029083	0.023769	0.029705	-0.40681	-0.30582	-0.53866
0.26233	0.28043	-0.18201	-0.49726	-0.12505	0.10382	0.38918
-0.13503	-0.1682	0.38048	0.48076	0.70855	0.4539	0.066003
-0.15029	0.22936	-0.36212	0.0066635	-0.58098	-0.43173	-0.052526
-0.059797	-0.059267	0.00074535	0.22185	0.34391	0.25565	0.18975
-0.32786	0.095923	0.30283	0.59092	0.56408	1.2436	0.53844
0.015032	-0.044562	0.11718	0.45275	0.12397	0.4428	0.40587
0.10629	0.089397	0.035946	0.24487	0.050082	0.017591	0.1039
0.042599	0.27096	-0.28046	-0.24159	-0.11899	-0.15002	-0.020721
-0.022892	-0.033438	0.019979	-0.30439	-0.9457	-0.57782	-0.07719
-0.24145	0.066392	-0.40288	-0.91365	-0.4503	-0.72673	-0.56523
0.068479	0.042278	-0.41075	-0.025381	-0.33512	0.074752	-0.21558
-0.091867	-0.095834	-0.22204	-0.18395	-0.32682	-0.42891	0.091744
0.45881	0.56544	-0.46696	-0.49867	-1.1023	-1.0027	-0.38118
-0.12677	0.086092	0.3176	-0.15811	-0.42799	-0.4949	0.31733
0.0048671	0.46781	0.089017	0.35567	-0.035591	0.004821	-0.28654
-0.087482	-0.17604	0.10217	0.17823	-0.16003	0.048233	0.041477
-0.18928	0.32265	-0.59036	-0.14576	-0.56311	-0.87336	-0.0039227
-0.039587	0.016262	-0.34223	-0.12284	-0.44154	-0.67476	0.029433
0.25237	0.10857	-0.045198	-0.10558	-0.54158	-0.015357	-0.01163
0.25734	0.067158	-0.015386	-0.19111	-0.14783	0.062811	0.13465
0.11073	-0.028414	0.074641	0.21268	0.60178	0.67163	0.55299
-0.10504	0.01581	-0.17408	0.032458	0.082084	0.082877	-0.05714
0.1993	-0.20159	-0.2434	0.037485	0.22256	0.4307	-0.27391
0.0038765	0.054863	-0.043317	0.1857	0.23577	0.36554	0.37733
0.10096	-0.1422	0.04369	0.20237	0.49971	1.022	0.37525
0.060452	0.25052	-0.28332	0.0053448	-0.43302	-0.44604	-0.22144
0.060166	0.13624	0.080922	0.27255	0.12725	0.091136	0.23314
-0.069863	0.092837	-0.26859	-0.10723	0.25577	0.0094823	0.053097
-0.071345	0.2028	-0.12081	-0.48926	0.26541	0.2246	-0.46031
0.28013	-0.15507	-0.87684	-0.92382	-0.92401	-0.83442	-0.52698
0.073685	0.26327	0.29931	-0.31847	-0.17788	0.17347	0.14201
0.017639	-0.11462	-0.0022231	0.29418	0.15318	-0.0013143	-0.40134
0.020704	0.24033	-0.072016	0.044295	0.061733	0.1357	0.081933

-0.060928	-0.14645	-0.096127	0.038732	0.05293	-0.094052	-0.27213
-0.12332	-0.17158	-0.21539	0.035737	0.27197	0.018287	0.238
0.091287	0.064201	0.14598	0.2548	-0.13021	0.095551	0.0055727
-0.069207	0.23719	-0.51395	-0.9908	-0.96989	-1.0941	-0.17348
0.047053	0.15932	0.36009	-0.017633	0.47429	0.84394	0.12315
0.1094	0.25977	-0.20723	-0.34951	-0.8319	-0.28943	-0.039849
-0.10796	-0.01971	0.36	-0.010301	0.49935	0.57309	0.10939
-0.1082	-0.32298	0.26262	0.17926	0.24296	0.44863	0.20689
0.14607	0.1589	-0.61565	-0.066607	-0.18566	-0.2039	-0.072794
0.058424	0.0024756	0.31662	0.33723	0.36103	0.40886	-0.077532
-0.18949	-0.046981	-0.20094	-0.047978	-0.37914	0.10924	0.00073937
-0.22886	-0.31889	-0.12087	0.042996	-0.74502	-0.50292	-0.17999
-0.18611	-0.082172	-0.097662	0.33047	0.028826	-0.13449	0.36027
0.19552	0.51844	-0.31063	-1.0909	-0.018345	-0.033922	-0.44784
-0.063752	-0.2765	-0.049783	-0.059485	-0.59861	-0.13653	-0.23404
-0.091366	-0.062321	0.084607	0.14966	-0.025537	-0.0087745	0.058148
-0.24703	0.17257	-0.71779	-0.3254	-0.048343	-0.32459	-0.020615
0.13792	0.28936	-1.7855	-2.6208	-2.5037	-2.8651	-1.1894
-0.22603	0.012856	0.10309	0.23026	0.23196	0.36388	0.32906
0.4728	0.38996	0.18457	-0.58636	-0.50532	-0.23668	-0.20589

test_GSE434	test_GSE434	test_GSE434	test_GSE439	test_GSE439	test_GSE439	test_GSE439
ref_GSE4344	ref_GSE4344	ref_GSE4344	ref_GSE4394	ref_GSE4394	ref_GSE4394	ref_GSE4394
GSE4344	GSE4344	GSE4344	GSE4394	GSE4394	GSE4394	GSE4394
GEO	GEO	GEO	GEO	GEO	GEO	GEO
cDNA	cDNA	cDNA	cDNA	cDNA	cDNA	cDNA
RIFAMPICIN:	RIFAMPICIN:	RIFAMPICIN:	BW25113:1,S	pnp_b3164:-	RIFAMPICIN:	RIFAMPICIN:
468	469	470	495	497	498	499
-0.51261	0.1042	-0.093484	0.0075878	0.10693	-0.13863	-0.54979
0.12973	0.48707	0.18634	-0.16291	0.095152	0.059038	0.10739
-0.033159	0.22801	0.083951	-0.21323	-0.13769	-0.063073	-0.074756
0.24052	0.14922	0.40215	0.47071	-0.1023	-0.3937	-0.31358
-0.048294	0.17587	0.036065	0.38731	0.43544	-0.23547	-0.19729
0.3211	0.19142	0.32562	0.8414	0.38175	-0.020299	-0.012582
-0.097124	-0.23004	0.32435	0.19078	0.020728	-2.7911	-2.6089
-0.50766	-0.65061	-0.42799	0.1494	-0.55614	-0.20356	-0.10423
0.15704	0.36566	0.33221	-0.34608	0.27804	0.095534	0.023508
0.064517	0.62903	-0.057848	1.0777	0.52992	0.18947	0.25576
-0.56586	-0.13659	-0.27868	-0.11436	-0.20358	0.11829	-0.040881
-0.89666	-1.2726	-0.94372	1.263	0.93381	0.074033	0.10478
-0.12791	0.077507	-0.15428	-0.13375	-0.084875	-0.12287	-0.2464
0.050922	0.36099	0.098014	0.77331	1.2181	-0.23244	-0.17778
0.082382	0.17375	0.053077	-0.1023	-0.23153	-0.055716	-0.19351
0.18839	0.14591	0.028147	0.0016921	-0.099376	-0.16877	-0.10691
-0.48615	-0.48062	-0.12721	1.4351	-1.1505	-0.51399	-0.67756
0.042685	0.19519	0.17837	-0.22826	-0.11957	-0.32139	-0.25281
0.75974	1.2334	0.53133	0.30302	0.61187	-0.31961	-0.24187
0.2908	-0.10409	0.44209	0.093611	-0.40954	-0.11532	-0.18642
0.15936	0.53927	0.045335	0.40393	-0.16373	0.24511	0.24147
-0.46258	-0.38798	-0.2845	0.24846	0.49567	-0.59448	-0.76026
-0.23243	-0.36308	-0.044404	0.17242	0.21108	-0.17743	-0.23842
-0.43642	-0.32439	-0.20921	-0.208	-0.95369	-0.45122	-0.40161
-0.03767	-0.038015	-0.093045	0.52297	0.34687	0.083291	0.18279
-0.38484	-0.51924	-0.39367	0.33414	-0.010197	-0.28376	-0.29055
-0.33223	-0.57937	0.59037	0.024628	-1.1239	-0.63358	-0.30074
0.2755	0.34608	0.50226	-0.24456	-0.18104	0.36118	0.88994
0.060473	-0.30548	0.41982	-0.040746	-0.06874	-0.52427	-0.33348
-0.66323	-0.71178	-0.62954	-0.027913	0.16287	-0.60485	-0.37804
-0.56419	-0.63627	-0.029774	-0.059027	-0.25722	-0.51314	-0.7285
-1.2396	-1.5537	-1.1251	1.0426	-0.61373	-0.59368	-1.0616

0.099131	-0.83199	-0.090169	-0.10864	-0.27155	-0.015518	2.4906
0.049771	1.1025	0.45206	0.021185	0.19106	-0.05254	-0.045832
-0.3462	-0.6005	-0.45843	0.33147	-0.027224	-0.48663	-0.37856
0.10665	0.1246	0.24406	-0.20646	-0.1978	-0.12551	-0.045061
-0.037501	0.12966	0.013732	-0.38522	0.12813	0.54948	0.63526
0.21276	-0.063666	0.40068	0.38673	-0.23983	0.16991	0.22698
-0.63889	-0.388	-0.50491	-0.0086232	0.15259	-0.16038	-0.55931
0.25793	0.36849	-0.090256	0.45983	0.6415	0.056574	0.14841
-0.24124	0.28083	-0.0010011	0.032286	0.17573	-0.39072	-0.094725
0.055375	0.6025	0.075303	-0.31157	-0.34409	-0.037384	-0.58041
0.10178	-0.094036	0.10466	0.10529	0.11872	0.24273	0.26584
0.13969	0.11983	0.11645	-0.29385	-0.4622	0.47363	0.34208
0.046655	0.21875	0.19812	-0.3485	0.36309	-0.40093	-0.43383
-1.137	-1.2598	-0.84099	-0.14423	0.43235	-0.67118	-1.1132
-0.84996	-1.4174	-1.0099	0.76466	0.22328	-0.43011	-0.49668
-0.1562	0.060741	0.13715	-0.1661	0.21098	-0.50336	-0.45444
0.48382	0.45844	0.027216	0.54799	0.60492	0.09413	0.10039
-0.32013	-0.24526	0.36278	-0.33549	0.027556	-0.1339	-0.2184
-0.78671	-0.50909	-0.30694	0.21886	0.61164	-0.34622	-0.6683
-0.046521	-0.053819	0.031253	-0.071161	0.39348	-0.019913	0.026037
-0.1363	0.014084	-0.12731	-0.11411	-0.024641	-0.39272	-0.31761
0.050616	-0.55883	-0.25197	-0.092418	0.049173	0.499	1.2188
-0.25546	-0.11741	-0.14128	0.41443	0.35918	-0.48848	-0.99654
-0.32857	0.27844	-0.090136	0.68907	0.61112	-0.26334	-0.37448
-0.079301	-0.016224	-0.013161	0.0075878	0.16211	-0.18888	-0.62259
0.0075861	0.04244	0.21889	-0.08461	0.24263	0.035069	0.23845
0.008595	-0.1467	-0.10879	-0.082758	-0.16162	0.54077	0.42874
-0.1566	0.34942	0.048845	-0.018141	0.52296	-0.28881	-0.39797
-0.068264	0.21519	-0.13025	0.31395	-0.53949	0.28956	0.16352
0.14089	0.063428	-0.04078	-0.3317	-0.28738	0.28743	0.14792
0.15084	0.018485	0.28027	0.0011053	0.28013	-0.037917	-0.13452
-0.16426	-0.04961	-0.17606	-0.45048	-0.079787	-0.53346	-0.47046
-0.2394	-0.2962	0.055873	-0.33771	1.0109	-0.16888	-0.12156
0.27135	0.17474	0.21316	-0.53635	0.44138	-0.016302	0.2509
-0.16844	-0.17813	-0.20977	-0.067481	-0.79453	-0.040211	-0.090906
-0.55799	-0.4501	-0.47624	0.23538	0.24795	-0.44765	-0.343
-0.35162	-0.49143	-0.4395	-0.35383	-0.14248	-0.12996	-0.044383
0.12851	0.22339	0.19563	-0.12363	-0.16123	0.11667	0.070315
-2.3019	-2.4503	-1.2863	0.65566	0.69661	-2.063	-2.3669
-0.97075	-0.98597	-0.95248	-0.022236	0.23326	-0.62538	-0.28812
-0.76049	-0.39858	-0.68609	0.43114	0.33873	-0.41014	-0.32077
0.46079	0.3792	0.33463	0.70707	0.044659	0.52525	0.6861

-0.37294	-0.74633	-0.36734	0.0077047	-0.26337	0.70354	0.86497
-0.93125	-1.1936	-0.85786	1.6057	-0.23345	1.2083	1.2668
-1.0843	-0.77546	-0.67318	0.74097	0.84419	-1.0215	-1.2205
-0.15014	-0.18621	-0.036369	-0.29131	0.13833	-0.15139	-0.20781
0.19684	0.32216	0.38171	-0.21547	0.4082	-0.085636	-0.17293
0.23697	0.24092	0.061544	-0.23495	0.034849	0.34162	0.02633
0.69178	1.0423	0.46419	-0.21982	0.027041	-0.34236	0.023864
-0.82586	-1.2262	-0.70054	-0.91859	0.099361	-0.29837	-0.45229
-0.45378	0.2705	0.042959	-0.61459	-0.46044	-0.13364	-0.4136
0.12579	0.3382	-0.011067	0.058251	0.3852	-1.0077	-0.90969
-0.15487	-0.050976	0.022649	0.07556	0.38768	-0.47986	-0.0872
-0.11866	0.089935	-0.28451	0.11432	0.0094323	0.31332	0.09849
-0.39215	-0.43097	-0.134	0.086198	0.50097	-0.35376	-0.50798
0.10385	0.12351	0.11106	0.35849	-0.015701	-0.022475	0.062902
-1.2579	-1.404	-1.1843	0.96431	0.49491	-1.0056	-0.91121
0.015033	0.26427	-0.16967	-0.0072395	-0.055271	-0.069756	-0.19483
-0.19444	-0.028051	-0.038351	0.0010237	-0.044883	-0.18153	-0.10242
-0.209	0.29415	-0.031759	0.1273	0.12874	-0.40952	-0.63918
0.07834	0.25333	2.41E-05	-0.038125	-0.96725	-0.0098006	0.11844
0.016917	0.0086675	-0.054682	0.15113	-0.21896	-0.11801	-0.10865
0.053195	-0.11046	0.023672	-0.043466	-0.02142	0.12799	-0.080666
-0.14704	-0.26148	-0.27959	-0.27498	-0.14502	-0.28771	-0.34739
0.14242	0.30342	-0.033536	0.35745	-0.014479	0.23266	0.21062
0.14791	0.21694	0.046475	-0.14947	0.24438	-0.1329	-0.2682
-0.10347	0.029937	-0.0018436	-0.090849	0.043132	-0.33605	-0.34536
-0.13213	-0.24489	-0.073945	-0.02393	-0.13461	-0.19454	-0.33178
0.013706	0.20878	-0.17122	-0.38789	0.0059755	-0.0025643	-0.16443
-0.81898	-1.0065	-0.61097	0.92854	0.39294	-0.84194	-0.67597
0.12944	0.11982	-0.021974	0.19909	-0.12973	0.1887	0.31635
-0.031484	0.5726	-0.13822	0.073815	0.50406	0.087933	0.30751
-0.099049	-0.23419	-0.17172	0.31925	0.067422	-0.048015	-0.098854
0.36269	0.58476	0.50054	0.34229	0.18418	0.48615	0.36432
0.25358	0.17916	0.43548	0.53376	0.32639	0.54948	0.15282
0.30286	0.40166	0.39564	0.24246	0.39396	0.0027366	0.16805
-2.2038	-2.2789	-1.4739	0.43387	0.27598	-0.83015	-1.2816
-0.59954	-0.49746	-0.35988	-0.040939	0.25277	-0.95788	-1.0204
-0.1039	0.20977	0.047518	0.035304	0.099336	0.011906	0.12621
-0.48531	-0.68234	-0.62783	-0.66582	0.3462	-2.0594	-1.7195
-0.39613	-0.13946	0.070664	-0.018141	0.40487	-0.19545	-0.18082
0.29543	0.51525	0.33279	0.31836	0.32305	0.26161	0.080817
0.044446	0.086587	0.14058	0.12739	-0.10367	-0.40538	-0.21313
-0.28648	-0.36889	-0.065508	0.87061	0.33145	0.22491	0.31019

-0.088023	0.14698	0.0096951	-0.097059	-0.11795	-0.31499	-0.22114
-0.97647	-0.78275	-1.2408	-0.10179	0.22167	0.0050009	0.063121
-0.20165	0.088988	-0.0096404	0.16379	0.15496	-0.079338	0.0015534
-0.41863	-0.12212	-0.1383	0.085144	-0.1461	-0.16744	-0.17573
-0.62627	-0.90148	-0.68551	1.0567	-0.19605	-0.55694	-0.82856
0.76863	0.87135	0.50954	0.3425	-0.62628	0.77218	0.38069
-0.45334	-0.26498	0.032093	0.18942	0.023702	0.24125	0.28341
-0.10534	-0.03937	0.0099949	-0.21968	0.33196	0.011895	-0.043897
-0.31249	-0.13822	-0.124	0.18337	0.30365	-0.072441	-0.12928
-0.21014	-0.025988	-0.38539	-0.34289	-0.05312	-0.42983	-0.33432
0.090925	0.14749	0.19086	0.24012	-0.048689	0.045887	-0.035721
-1.097	-0.8627	-1.1014	-0.16174	-0.45733	-0.46172	-0.60735
-0.31931	-0.38628	-0.31877	-0.37222	-0.091962	-0.32845	-0.3865
-0.28177	-0.41607	-0.28767	0.62725	1.468	-0.26351	-0.58506
0.1909	0.29331	0.25486	0.3783	1.1277	-0.027121	0.1971
-1.4145	-1.3947	-1.5934	0.34037	0.30295	-0.87445	-0.68142
-0.1275	0.16842	-0.28432	0.76983	-0.093528	0.036232	-0.12981
0.20798	-0.048011	0.3732	0.16186	-0.40652	0.4409	0.33564
-0.73483	-0.83493	-0.46374	0.12178	0.30876	-1.3658	-2.0706
-0.36373	0.33088	0.099029	2.4166	1.4152	2.234	2.5463
0.13653	0.068993	0.12288	0.14379	0.3863	0.17339	0.14526
-0.25945	-0.48115	-0.43354	0.020367	0.09352	0.017176	-0.15552
NaN	NaN	NaN	NaN	NaN	NaN	NaN
-0.092668	0.075889	-0.036485	0.48755	0.58594	-0.37834	-0.26521
-0.60493	-0.86864	-0.33634	0.76864	-0.22749	-0.37538	-0.54175
0.041443	-0.25162	0.16902	0.24705	0.32986	-0.12911	0.082681
-0.32586	-0.10635	-0.14294	0.19855	0.12535	-0.6553	-0.73885
0.015611	-0.014549	-0.19241	0.24459	0.029621	0.040157	0.24898
-0.14988	-0.11999	-0.15718	-0.30286	0.54362	0.13307	0.16363
-1.9371	-2.1745	-1.9221	-0.016874	-0.057494	-0.58564	-0.5301
0.33002	0.23221	0.22759	-1.0425	0.20875	-0.14651	-0.21839
-0.04237	0.011694	-0.072504	-0.38015	-0.32322	-0.4205	-0.40206
-0.078046	0.61688	-0.20215	0.61545	0.65078	0.27277	0.19363
0.023889	0.13109	0.14303	-0.69176	0.086424	-0.29008	-0.2525
-0.0075717	0.22327	0.031241	0.28362	0.38775	0.24167	0.06192
-0.26675	-0.17368	-0.19068	0.17652	-0.092495	0.018863	-0.10421
-0.55259	-0.97843	-0.60395	0.42946	0.32371	0.48153	0.29369
0.15507	0.007823	0.132	-0.37402	0.13923	0.16687	0.059894
-0.080292	0.14114	0.19005	-0.11156	0.087873	-0.10392	-0.19223
0.39648	0.46389	0.93103	-0.07444	0.19702	-0.026927	0.0018985
-0.30073	-0.18544	-0.33111	-0.23144	0.28732	-0.19278	-0.3224
-0.17036	-0.44085	-0.27058	-0.12694	0.17637	-0.72455	-1.207

-0.083685	0.17074	-0.098083	-0.0079387	0.072321	-0.024587	0.16808
0.15785	0.15732	0.024736	0.32592	-0.029902	-0.065972	0.093187
-0.22364	0.21065	-0.15637	0.071998	0.18608	-0.14831	-0.32613
0.13531	0.013888	0.023835	-0.19068	0.51842	0.20011	0.10863
0.38449	0.36701	0.46723	0.050702	0.0042292	0.097484	0.18843
0.052051	0.31271	0.32105	0.14028	0.2951	-0.31882	0.0063782
-1.6019	-1.6968	-1.0654	-0.19643	-0.17429	-1.2935	-1.0904
-1.8644	-2.5219	-2.5141	1.4303	-1.014	-0.67606	-0.70715
-0.1713	0.0036754	-0.18614	0.4509	1.3422	-0.80302	-0.88249
0.23105	0.39204	-0.10087	0.11645	0.28083	0.3367	0.094106
-0.012967	-0.20127	-0.43792	0.42071	0.17038	0.38189	0.42798
0.00010103	0.11351	0.0071406	0.11099	0.12876	-0.31519	-0.27878
0.21351	0.74287	0.053778	-0.070831	0.037306	-0.2525	-0.36182
0.18161	0.85392	0.13997	-0.35094	-0.058625	0.26228	0.15248
0.19522	0.33734	0.14011	0.44132	-0.22201	-0.17356	-0.31662
-0.37822	-0.15814	-0.14333	0.024326	-0.21383	0.035946	0.023374
-0.12222	0.51094	-0.053699	-0.11008	-0.38462	0.048816	0.27709
0.41459	0.46825	0.099643	-0.48834	0.13247	0.44829	0.63564
-1.0761	-1.7096	-1.5803	-0.37869	0.31183	-0.16709	-0.14054
0.15964	0.4004	-0.02567	0.4862	0.26012	0.34401	0.23533
-0.11471	0.19457	-0.027426	-0.2119	0.046637	-0.02038	-0.093843
-0.10472	0.069309	0.28654	-0.099753	0.28428	-0.35376	-0.44814
-0.18923	-0.11567	-0.051063	0.090324	0.14527	-0.70277	-0.54368
-0.12833	0.043933	-0.0027753	-0.023865	-0.54277	0.3315	0.26297
-0.1503	-0.21643	-0.31921	0.14874	0.40306	0.085233	0.065667
-0.10426	0.074381	0.015311	-0.51209	-0.23947	-0.42087	-0.50571
-0.092219	-0.20795	-0.24881	0.50543	0.24163	0.12353	0.31486
-0.29895	-0.31272	-0.14131	-0.10653	0.41934	-0.34877	-0.39195
-1.2233	-1.0805	-0.7735	-0.0097358	0.17299	-1.0001	-1.7185
-0.25058	-0.22939	-0.35189	0.14566	0.80072	-0.62025	-0.46782
-0.048998	0.26797	0.20697	0.075499	0.18853	-0.12611	0.21535
0.017764	-0.15372	-0.054181	0.0068263	-0.29874	-0.036887	0.03013
0.021157	0.30604	-0.10815	-0.41879	-0.43476	-0.2485	-0.28359
0.48721	0.88008	0.55111	-0.39323	-0.20964	-0.079599	-0.16207
0.029678	0.33392	-0.18212	0.18121	0.11896	-0.14182	-0.039991
-1.6916	-1.5127	-1.2642	0.25263	0.2585	-1.1306	-1.2274
0.17202	0.39105	0.257	-0.02583	-0.076166	0.28975	-0.0091428
-0.75402	-0.58104	-0.55321	0.1217	0.37849	-0.6714	-0.31737
0.021196	-0.14596	-0.23859	0.83579	1.1615	-0.3777	-0.22961
-0.15875	-0.089321	0.10723	-0.15846	0.21603	-0.36868	-0.54976
-0.70752	-1.1505	-0.0791	0.084418	0.51287	-0.077536	-0.30449
-1.3225	-1.234	-0.85885	0.099335	1.0396	-0.27005	-0.31598

-0.35608	-0.29927	-0.13828	0.32654	0.23406	-0.44059	-0.50922
-0.063464	0.12196	0.1936	-0.33991	0.10471	0.028352	-0.055624
-0.0051294	-0.21669	-0.2674	0.046396	-0.43432	-0.26449	-0.07571
0.032453	-0.038537	-0.27593	0.4544	0.48905	-0.2184	-0.2229
-0.31107	-0.2493	-0.2982	0.16507	0.30171	-0.10352	-0.16876
-1.6509	-1.4205	-1.444	0.46165	0.57576	-1.1499	-1.0694
-0.7233	0.057047	-0.21709	0.52132	0.53069	-0.32388	-0.20841
0.072078	0.35913	-0.077706	0.17488	-0.015838	0.2988	0.25321
0.033308	0.34125	-0.1292	-0.34478	-0.19627	-0.088983	0.13244
-1.1281	-1.3426	-0.69287	0.73451	0.80297	-1.2409	-1.4283
0.11195	0.78833	-0.1092	-0.14879	0.036439	0.1699	0.1344
-0.93873	-0.78146	-0.14276	0.06405	0.47494	-1.1263	-1.6381
-0.23206	-0.32541	-0.25812	-0.056878	0.035866	-0.45788	-0.55504
-0.67726	-0.34437	-0.074228	0.6452	0.098351	-0.36948	-0.38268
0.53557	0.12434	0.17253	-0.46359	-0.17757	0.62504	0.6849
0.25868	0.3081	0.027641	-0.076789	0.6216	0.034934	0.060581
-0.5314	0.051933	0.13342	-0.12017	0.417	-0.3645	-0.58164
-0.2046	0.11462	-0.6237	-0.0070431	0.073055	0.17615	0.34046
-0.80193	-0.55261	-0.27186	-0.022016	0.025236	-0.30833	-0.58451
0.61379	0.93087	0.28445	0.31438	0.17216	-0.1343	0.04663
-0.078078	0.72467	0.095686	-0.21691	-0.11074	-0.73585	-0.40029
0.073188	-0.31576	-0.17832	0.020006	0.45876	0.083295	-0.056075
-0.10442	-0.20221	-0.28771	-0.033966	0.3242	-0.1707	-0.073119
-1.0169	-1.1796	-1.0598	-0.010481	0.52721	-1.1277	-1.6918
-0.1076	-0.026858	-0.18962	0.010494	-0.057579	-0.4632	-0.3677
-0.14358	0.23199	0.1356	0.31932	0.31545	0.15318	0.40004
-0.46434	-0.81946	-0.60957	-0.074009	-0.16197	-0.25768	-0.48498
-0.99596	-0.87838	-0.78427	-0.17935	-0.67123	-0.092449	-0.075117
-2.1657	-2.3092	-2.1198	-0.24889	-1.4827	-1.2618	-1.0125
-1.0585	-0.92014	-0.5269	0.10415	-1.0025	-1.5108	-1.7823
0.17873	0.32717	0.32334	0.25574	-0.33191	0.25193	0.13103
0.33514	0.20038	0.25919	-0.035047	0.059845	0.46484	0.0048245
0.26673	0.087382	0.79664	-0.39026	-1.2884	0.39639	0.48441
-0.13298	0.08264	-0.07977	0.15533	1.0465	0.023996	-0.066529
-0.20437	-0.23161	-0.076813	0.36527	0.57531	-0.17509	-0.19495
0.050877	0.36109	-0.16182	-0.12155	-0.1593	-0.0009735	0.10466
-0.14286	0.81816	0.16703	-0.2642	-0.029939	-0.42757	-0.6523
0.48712	0.83305	0.37732	0.51221	-0.021338	0.17145	0.058371
0.40738	-0.023162	0.58486	-0.071187	-0.24406	0.11564	0.059894
0.3533	-0.030447	-0.051885	0.071042	0.0012659	0.27662	0.25367
0.28768	0.086917	-0.041597	-0.39328	-0.15091	-0.48374	-0.34772
-0.42913	-0.11676	0.16909	-0.25394	-0.05806	-0.097587	-0.19894

-0.35919	-0.091094	-0.019167	0.11316	0.16557	-0.10135	0.044148
0.012411	0.13388	0.15328	-0.25037	-0.051084	0.13475	0.093397
-1.4009	-2.2004	-1.6449	0.82868	0.00092364	-0.89189	-0.8949
0.090507	-0.052023	0.14599	-0.1244	0.72737	-0.36223	-0.52245
0.10526	0.11878	-0.063667	0.085308	-0.055425	-0.34485	-0.18014
0.082275	-0.0009961	0.35083	-0.17444	0.16879	0.30362	0.1471
-0.57876	-0.47018	-0.24645	0.391	-0.20768	-0.11927	-0.28579
-0.21638	-0.43043	-0.23953	-0.19646	-0.10055	-0.046504	0.012786
0.022081	-0.020764	0.11162	0.0096001	0.038102	-0.36528	-0.42216
-0.33687	-0.16371	-0.3065	-0.040375	-0.18422	-0.26492	-0.12581
0.72275	0.51519	0.37008	0.14669	0.043586	0.34769	0.38911
-0.12339	-0.32934	-0.012521	-0.47987	-0.057529	-0.02852	-0.59746
0.12127	0.14253	0.078571	0.10123	0.16036	0.055374	0.2179
0.18797	0.2336	0.50035	-0.42513	-0.078404	-0.092594	0.0075594
0.80046	0.81128	0.65102	0.037193	0.62715	0.42557	0.31813
0.39111	-0.068715	0.16453	-0.17163	0.0367	-0.18668	0.037472
-0.26945	0.02828	-0.1182	-0.08454	0.21598	-0.12519	-0.18856
-0.32297	-0.37361	-0.27436	-0.78927	1.1818	-0.48601	-0.36718
-0.86072	-0.71426	-0.90167	-0.52736	-0.22657	-0.45741	-0.77515
-0.06282	-0.080684	0.072415	-0.26318	0.24866	-0.48916	-0.22417
-0.12843	-0.38669	-0.34233	0.52287	0.67729	-0.2795	-0.33148
-0.69121	-0.55289	-0.4368	-0.088022	0.29278	-0.97068	-0.88612
-0.37305	-0.36512	-0.18181	0.30597	0.21726	-0.12196	-0.28026
0.29875	0.25835	0.522	0.16962	0.021367	0.6002	0.89833
0.0059067	0.14203	0.22414	-0.0088392	0.17146	-0.052944	0.046466
-0.23697	-0.16343	-0.093901	0.22478	0.41633	-0.2909	-0.8804
-0.027959	0.44386	-0.18971	0.060942	0.24992	-0.88608	-0.47542
-0.0029077	0.15235	0.047219	-0.22047	0.075825	-0.5631	-0.39307
-0.17437	0.17954	-0.10601	-0.14982	-0.24192	-0.38486	-0.47964
0.53662	-0.079437	0.41742	0.12547	-0.021975	0.26705	0.039335
-0.055739	0.14216	0.15491	0.098019	0.46776	0.1849	0.1903
-0.10819	-0.02144	-0.24398	-0.02195	0.011188	0.026924	-0.021273
-0.072117	0.13522	0.22671	-0.55765	-0.17871	-0.28225	-0.22356
0.071545	0.14771	0.19308	-0.305	-0.27247	-0.039027	0.5265
-0.060626	-0.38312	-0.13586	-0.31037	-0.051044	-0.51014	-0.54275
0.25202	0.48855	0.3664	0.14535	0.053104	-0.060286	0.043469
-0.17838	0.2102	-0.16166	-0.22571	-0.28774	-0.099984	-0.30113
-0.35503	-0.60192	-0.67663	-0.046622	-0.85105	-0.047816	0.018878
-0.92526	-0.82204	-0.819	0.57992	0.16508	-0.55623	-0.66783
-0.10291	-0.25874	-0.31205	-0.065738	-0.71624	0.18629	0.12697
-0.18192	0.30412	0.36943	0.029757	0.19743	-0.53666	-0.54132
0.10762	0.38071	0.077737	0.0040387	-0.12023	0.080815	-0.017941

0.00091213	-0.064658	-0.032025	-0.076876	0.0081702	-0.049982	-0.26769
-0.044135	0.016178	0.061189	-0.22426	0.054286	-0.44223	-0.13346
0.28545	0.29858	0.28199	0.17888	0.11789	0.58922	0.63753
-1.0769	-1.0233	-1.2307	0.16428	-0.19003	-0.3139	-0.042223
0.0037472	-0.59997	0.18778	0.36053	0.0093447	-0.41196	-0.49011
0.13719	-0.13556	-0.32676	0.80125	0.29368	-0.065343	-0.21942
0.063034	-0.35168	0.32144	-0.39712	-0.15457	0.06991	-0.067775
0.13071	0.17839	0.31771	-0.40944	0.036459	0.089209	-0.080982
0.45456	0.43407	-0.55121	1.6218	1.5391	-0.59433	-0.41783
0.015208	-0.63366	0.53071	0.67295	0.79926	-0.46384	-0.25896
-0.32882	0.063722	-0.21438	0.18109	0.52238	-0.37604	-0.38755
0.034579	0.16879	-0.050583	-0.10554	0.35441	0.22678	-0.23913
0.48375	0.46067	0.43653	-0.191	0.064859	-0.30688	-0.20883
-0.93648	-0.87938	-0.55129	0.08536	-0.12925	-1.0956	-0.97863
-0.16606	0.14353	-0.0072631	0.45429	0.26911	-0.086814	-0.39416
0.12094	-0.060924	-0.185	-0.0083469	0.006607	0.5896	0.48237
-0.30668	0.02637	-0.42747	0.39366	-0.096795	-0.1375	-0.13697
-2.6461	-2.0639	-2.742	0.86974	0.36987	-2.2915	-1.5554
-0.16249	-0.35016	0.04091	-0.18089	0.038917	-0.070312	-0.17435
-0.41973	-0.69136	-0.79986	0.79427	-0.57786	-0.31713	-0.1732

test_GSE439	test_GSE439	test_GSE439	test_GSE439	test_GSE439	test_GSE439	test_GSE439
ref_GSE4394	ref_GSE4394	ref_GSE4394	ref_GSE4394	ref_GSE4394	ref_GSE4394	ref_GSE4394
GSE4394	GSE4394	GSE4394	GSE4394	GSE4394	GSE4394	GSE4394
GEO	GEO	GEO	GEO	GEO	GEO	GEO
cDNA	cDNA	cDNA	cDNA	cDNA	cDNA	cDNA
RIFAMPICIN:	RIFAMPICIN:	RIFAMPICIN:	RIFAMPICIN:	RIFAMPICIN:	RIFAMPICIN:	RIFAMPICIN:
500	502	503	504	505	506	507
-1.0301	-0.14677	-0.30163	-0.18638	-0.35994	-0.28891	-0.1395
0.17797	-0.022118	-0.076562	-0.13834	0.17594	-0.0029401	0.066212
-0.17351	0.099253	-0.045757	-0.021381	-0.037418	-0.057406	-0.015406
-0.35337	-0.10339	0.032815	-0.050404	0.069092	0.014578	-0.009194
-0.043387	0.047723	-0.50056	-0.096264	0.092716	0.10682	0.12779
-0.77392	-0.058373	0.05981	-0.062078	0.055788	0.073988	-0.2949
-1.8458	-0.26566	-0.91788	-0.85947	-0.70858	-0.54513	-0.49242
0.070859	-0.10846	0.01221	-0.050107	-0.16233	0.26843	-0.3836
-0.26938	-0.1493	-0.11724	-0.16741	-0.032802	-0.22339	0.10764
0.15123	0.087363	-0.17604	0.24339	0.21148	-0.045031	-0.27933
-0.0086574	-0.079146	-0.21921	-0.13819	-0.35222	0.22973	-0.60775
0.22104	-0.034545	-0.12656	-0.021695	-0.20361	0.052316	-0.0034026
0.090618	-0.16248	-0.17878	-0.15586	-0.12418	0.074335	0.26945
0.0068312	-0.22288	-0.45947	-0.18306	-0.083624	-0.32525	-0.28418
-0.092357	-0.16145	-0.10722	-0.0616	-0.13019	0.0059008	0.0082587
0.040046	-0.059442	-0.25512	-0.18994	0.012195	0.045645	-0.095325
-0.64798	0.044074	-0.27296	0.1368	-0.29274	0.048137	-0.078593
-0.0457	0.02503	-0.11212	-0.18319	-0.017024	0.13189	0.0091546
0.092471	-0.46338	-0.44594	-0.22448	-0.15397	-0.39942	-0.26198
0.21506	0.073669	-0.30658	-0.24211	-0.1562	-0.09224	-0.43001
1.0478	-0.10539	0.059831	-0.12309	0.36398	0.28092	0.23538
-0.64681	0.15122	-0.077356	-0.091778	-0.17345	0.15613	0.098259
-0.3647	-0.1608	-0.15939	-0.16928	-0.27433	0.14951	-0.31722
-0.1085	-0.015073	-0.066081	-0.075829	-0.031907	0.044347	-0.035293
-0.062928	-0.086128	-0.04074	-0.057762	0.11485	-0.14204	-0.01852
-0.67556	-0.26118	0.026651	-0.085784	-0.13447	-0.15952	-0.16968
-0.025759	0.15058	-0.058787	0.17228	-0.22123	0.090966	-0.21197
1.0005	0.14416	0.058525	-0.0012729	0.01517	0.1618	0.21551
-0.3272	-0.18671	0.18484	-0.32554	-0.20427	-0.21268	-0.29812
-0.46573	-0.20044	-0.18358	0.10761	-0.2844	-0.13579	-0.12851
-1.0335	-0.39044	-0.35511	-0.39728	-0.55908	-0.59765	-0.51528
-1.1793	-0.23557	-0.51548	-0.36537	-0.85651	-0.65379	-0.65855

-0.66317	-0.21692	-0.20463	-0.26828	-0.040999	-0.16719	-0.18325
0.47034	0.034419	-0.2153	-0.09922	0.12839	0.021925	-0.1106
-0.38742	-0.1055	-0.22759	-0.13532	-0.26307	0.0013447	-0.20162
-0.17961	0.247	0.0013756	-0.022151	-0.092516	0.12198	0.14638
0.46126	-0.018346	0.13828	-0.068145	-0.33737	0.021221	-0.21471
1.118	0.14284	-0.43485	-0.1018	-0.27941	0.0056896	-0.66916
-0.012596	0.034752	-0.18009	0.066075	-0.29273	-0.17802	-0.033626
0.62995	-0.0077241	-0.27692	-0.062615	-0.084467	0.13319	4.80E-05
0.0741	0.027992	-0.14041	-0.042561	0.0071601	-0.066647	-0.16022
-0.27361	-0.13501	-0.37092	-0.025563	-0.65596	-0.093414	-0.16427
0.15162	0.25719	0.30087	0.15048	0.22113	0.22994	0.024372
0.077151	-0.20071	-0.11537	-0.11438	-0.16399	-0.13478	-0.31443
-0.38749	-0.069452	-0.14119	-0.087681	-0.2407	-0.016986	-0.17524
-1.6158	-0.092369	-0.49756	-0.047102	-0.57134	0.020358	-0.42355
-1.0535	0.070792	-0.40399	0.044702	-0.55404	-0.44914	-0.8786
-0.4462	-0.19653	-0.22182	-0.22696	-0.18256	-0.14023	-0.28727
0.18038	0.2015	-0.0024766	0.12851	0.1799	-0.081917	-0.011603
-0.19707	-0.19541	-0.022812	-0.19848	-0.22335	-0.1201	-0.40223
-0.46549	-0.36866	-0.45367	-0.20784	-0.50731	-0.472	-0.14584
0.43769	0.12449	-0.06676	-0.092662	-0.24864	0.02169	0.059393
-0.53975	0.05728	-0.53655	-0.32945	-0.015105	-0.10707	-0.10918
-0.85791	-0.064494	0.02763	-0.027956	0.072263	0.068881	-0.0020621
-1.2297	-0.19146	-1.0223	-0.22623	-0.67025	-0.69463	-0.22768
-0.69663	-0.18508	-0.59027	-0.088302	-0.021649	-0.6329	-0.41638
-0.31588	-0.19453	-0.38937	-0.2391	-0.46452	-0.21926	-0.11513
0.51563	-0.013194	-0.1184	-0.10747	-0.081952	-0.056303	-0.027724
0.14403	-0.13188	0.18245	0.3555	0.21323	-0.0016789	0.1775
-0.51235	-0.034924	-0.16814	-0.11181	-0.40874	0.02962	-0.087861
0.3424	0.038522	0.31214	0.19728	0.079548	0.2531	0.25268
0.80519	0.099898	0.018525	-0.13093	-0.20503	-0.14326	-0.057191
-0.17586	-0.1369	0.038483	-0.047603	-0.16493	0.048711	-0.18993
-0.23264	-0.0015301	-0.073116	-0.23317	-0.11685	-0.16816	-0.043143
-0.18388	0.16489	0.21972	0.00097762	0.097385	0.041399	-0.012429
0.71608	-0.062705	0.88251	0.33948	0.73844	0.5521	0.56656
-0.83004	-0.083374	-0.23512	0.17246	-0.31307	-0.30728	-0.66443
-0.62368	-0.1748	-0.24282	-0.68891	-0.11415	-0.67049	-0.10745
-0.14444	-0.07643	-0.27443	-0.096395	-0.26609	-0.070515	-0.25786
0.004139	-0.11955	-0.077268	-0.041273	0.22676	-0.025255	0.32669
-2.2839	-0.70537	-1.4973	-1.0065	-1.4375	-0.8279	-1.1224
-1.0882	-0.78169	-1.1831	-0.35965	-0.54703	-1.0808	-1.092
0.06026	-0.11418	-0.15599	0.1572	-0.20367	-0.28886	-0.60423
0.93591	0.25958	0.32698	0.2511	0.14868	0.42986	0.44261

0.80917	0.01069	-0.17369	-0.027933	-0.22952	0.049426	-0.051195
0.48844	-0.22215	-0.82494	-0.08157	-0.78492	-0.45813	-1.2537
-1.8576	-0.58615	-0.66338	-0.53557	-1.2281	-1.5224	-1.8743
-0.44609	0.19292	-0.067873	0.010722	-0.090221	0.022066	-0.074098
0.28581	0.086726	0.011857	-0.098504	-0.31895	-0.049971	0.078412
-0.050738	-0.098472	0.15256	-0.0045951	0.12723	0.020049	0.14493
-0.40887	0.4893	-0.098502	-0.10846	0.033281	-0.18194	0.11784
-0.6257	-0.35135	-0.22947	0.034282	-0.44631	-0.20681	-0.048715
-0.40836	-0.43114	-0.30825	-0.30645	-0.50017	-0.4233	-0.424
-1.2833	-0.56343	-0.98137	-0.7003	-0.96642	-1.0919	-0.97403
-0.12999	-0.12385	-0.581	-0.2499	-0.19657	-0.21305	-0.54554
-0.098728	0.12888	-0.14087	0.20589	0.026959	0.032076	-0.073004
-0.34459	-0.28077	-0.78625	-0.078247	-0.83262	0.032613	-0.81773
-0.24123	-0.20691	0.21487	0.0063813	0.18233	-0.20188	-0.066095
-1.3666	0.089385	-0.969	0.12728	-0.82345	-0.2194	-1.5094
0.080842	0.25696	-0.14488	0.016019	-0.060701	0.10041	0.010813
-0.98787	-0.042835	-0.52273	-0.26099	-0.031182	-0.099628	-0.072104
-0.026326	-0.043679	-0.28154	0.022408	-0.41906	-0.31446	-0.3214
-0.16659	0.039814	0.19932	0.0070766	0.026813	-0.072683	-0.0052144
-0.30658	-0.069533	-0.17001	-0.19122	-0.15062	-0.071589	-0.056235
0.060779	0.019282	-0.050773	-0.28798	-0.1266	0.063474	-0.15349
-0.47542	-0.0248	-0.25384	-0.085739	-0.31531	-0.19968	-0.05265
0.019047	0.14641	-0.083181	0.068439	0.21326	0.19135	-0.042981
-0.35502	-0.14323	-0.136	-0.070672	-0.37252	-0.036001	-0.044968
-0.39821	-0.27844	-0.41731	-0.19768	0.021591	-0.1986	-0.0089527
-0.45478	-0.26939	-0.091192	-0.44032	-0.4542	-0.1763	-0.32454
-0.49938	-0.21896	-0.11329	0.11391	-0.073957	-0.21063	-0.26187
-0.82475	-0.36294	-0.36504	-0.26043	-0.59411	-0.50636	-0.44592
-0.089104	0.16528	-0.21495	-0.052054	0.26781	-0.096884	0.012535
0.10662	0.27644	-0.39138	-0.12106	-0.088347	0.09687	-0.11073
-0.33471	-0.097195	-0.21635	-0.3167	-0.11915	-0.21002	0.0091748
0.3943	0.085808	-0.11446	-0.1178	0.11821	0.040267	0.11523
0.77274	0.56237	-0.0040039	0.085632	-0.022355	0.26635	0.056466
0.043911	0.24931	0.0020453	0.044083	0.63649	-0.049697	0.35971
-0.93546	-0.2671	-0.9811	-0.234	-1.0289	-0.45165	-1.0609
-0.91475	-0.19685	-0.67214	-0.65848	-0.56803	-0.68478	-0.3076
0.23262	0.055113	0.017845	-0.19556	0.21526	-0.048538	0.21396
-2.3444	-1.0097	-1.6322	-0.92464	-1.206	-1.4069	-1.1678
-0.1062	-0.12683	-0.41819	-0.32286	-0.48136	-0.26322	0.0026744
0.77	-0.08065	0.01021	0.05465	0.016721	0.026705	-0.27092
-0.61773	-0.17286	-0.10756	-0.26609	-0.13049	-0.20018	-0.31885
0.085694	0.19257	0.23096	0.1123	0.078343	-0.058305	-0.52819

-0.30832	-0.060524	-0.15627	-0.20109	0.042359	0.053155	-0.027581
-1.1119	0.087548	0.10118	0.17688	-0.17024	-0.27708	-1.0193
-0.31436	-0.048959	-0.26461	-0.13333	0.023678	-0.050532	0.0030262
-0.12306	-0.0041516	-0.078358	-0.031158	0.094029	-0.052989	-0.039223
-0.92643	-0.17114	-0.42073	0.34187	-0.39852	-0.10255	-0.14768
1.1684	0.21235	0.52709	0.22203	0.51899	0.27957	0.49929
0.049566	0.06035	-0.023018	0.084029	-0.19985	-0.098923	-0.026768
0.58257	0.15466	-0.25655	-0.33215	-0.2899	-0.16823	0.12291
0.1306	-0.083131	-0.18462	-0.03005	0.073288	-0.092545	-0.073969
0.0060536	-0.24615	-0.3596	-0.21612	-0.28892	-0.49975	-0.49411
0.36935	0.12298	0.32	-0.049158	0.12466	0.08624	-0.019262
-0.87679	-0.24054	-0.36003	0.037191	-0.56258	-0.34188	-0.9082
-1.0214	-0.19881	-0.20033	-0.19192	-0.15242	-0.71519	-0.47732
-0.12077	-0.028604	-0.34477	-0.017823	-0.39545	-0.5972	-1.1473
-0.30635	-0.39439	-0.99185	-0.045112	-0.8002	-0.75321	-1.9868
-1.3935	-0.56612	-0.66855	-0.43404	-0.56488	-0.90209	-1.4116
-0.25173	-0.24828	0.056417	-0.037811	-0.4641	0.1189	0.10872
0.35864	0.15548	0.030945	-0.059951	0.1675	0.15301	0.20418
-2.0319	-0.56304	-0.6645	-0.74928	-0.96844	-0.86979	-0.54833
2.0031	-0.12514	-0.022962	0.047519	-0.30367	-0.038492	-0.26141
0.45034	-0.11918	-0.084527	-0.12839	0.085532	-0.058186	0.13557
0.19258	-0.08457	0.074684	-0.063923	-0.071207	0.010976	0.15541
NaN	NaN	NaN	NaN	NaN	NaN	NaN
-0.9778	-0.12486	-0.37456	-0.027816	-0.30366	-0.18613	-0.90423
-0.19341	-0.2138	-0.21142	-0.26586	-0.15778	-0.2315	-0.072941
-0.055839	-0.19796	-0.033234	-0.24726	-0.42551	0.047845	-0.20525
-0.20116	-0.089615	-0.10306	-0.056703	-0.19124	-0.15908	-0.036432
-0.18054	0.13536	-0.35672	-0.014077	0.12099	-0.10792	-0.08596
-0.65874	0.19885	0.17688	0.16616	0.27608	-0.31433	-0.40076
-0.68203	-0.092622	-0.24676	-0.13621	0.020718	-0.24238	-0.40453
-0.22986	-0.21125	-0.12509	-0.33074	-0.1511	-0.72408	0.039096
-0.18047	-0.18388	-0.36274	-0.13889	-0.060056	-0.12161	0.07332
0.085803	0.21652	0.07137	0.059323	0.10953	-0.055322	0.017513
-0.10053	0.34993	-0.06663	0.17371	-0.35635	0.54661	0.036006
-0.26293	-0.12161	0.17329	0.28999	0.18314	0.19816	0.12954
0.13726	0.0019691	0.1417	0.78123	0.05053	-0.046742	0.12461
-0.29906	0.050681	0.030947	-0.0083719	-0.063013	0.026042	-0.12431
0.023817	0.0048825	0.088732	0.18524	0.14623	0.17102	0.19656
-0.01349	-0.096517	-0.17332	-0.21391	0.00067841	-0.029099	0.17742
-0.037348	0.36524	-0.10398	-0.044702	-0.017261	-0.073902	-0.13795
-0.15998	-0.24879	-0.22291	-0.11719	-0.19346	-0.1673	-0.089313
-1.0709	-0.43231	-0.80137	-0.41861	-0.73256	-0.46079	-0.61344

0.38131	0.083825	0.059709	-0.10566	0.10979	-0.056394	0.10655
0.22943	0.13077	-0.12463	-0.13882	-0.09086	0.039725	-0.089118
0.32205	-0.20347	-0.13454	0.058041	-0.22704	-0.28897	-0.12395
-0.043033	0.27616	-0.13541	-0.12828	0.071439	0.20017	0.067334
0.24249	0.071641	0.19946	-0.0087371	-0.0092249	0.068913	0.07434
0.17888	-0.099259	-0.16842	-0.16241	0.00027501	-0.029309	0.18452
-1.312	-0.36339	-0.65553	-0.42013	-0.48438	-0.91039	-0.62868
-0.87585	-0.26396	-0.24203	-0.22508	-0.21937	-0.18129	-0.17432
-0.056405	-0.16779	-0.77719	-0.063923	-0.85071	-0.37823	-1.8174
0.6286	-0.11027	0.20032	0.088984	0.28555	0.012953	0.065717
0.1197	0.11067	0.48031	0.12852	0.34645	0.088921	0.085651
0.30963	0.065923	-0.12965	-0.076726	-0.087527	-0.31114	-0.22965
-0.19069	-0.044026	-0.36958	-0.093215	-0.063679	0.023214	-0.0039566
-0.0092735	-0.08241	0.16606	0.10323	0.18886	-0.15613	0.29685
0.53629	-0.20315	0.03428	-0.16863	-0.0091251	-0.012439	0.19348
-0.42209	0.13101	-0.1326	0.1972	-0.21632	-0.014306	-0.32087
0.072328	-0.054667	-0.0008004	0.23987	0.028317	0.30594	0.13989
0.81291	-0.028494	0.17604	0.14795	0.14797	0.095955	0.20827
-0.977	0.04887	-0.22136	0.11772	-0.39605	-0.41551	-0.32827
0.53851	0.073472	0.033698	-0.078495	0.1696	0.22412	-0.016728
-0.39762	0.054536	-0.069075	0.0016173	-0.17377	-0.042405	-0.019771
0.15522	-0.15122	0.055651	-0.1664	-0.30219	-0.043813	0.22874
-0.5244	-0.055538	-0.59077	-0.28122	-0.34577	-0.33181	-0.33827
0.75408	-0.029996	-0.2752	-0.093042	-0.30999	-0.27716	-0.071464
0.060855	0.17692	0.14925	0.13556	0.054365	0.072932	0.084155
-0.25346	-0.27724	-0.59979	-0.23163	-0.10729	-0.1421	0.19774
0.19618	-0.053219	0.25257	0.11242	0.064913	-0.1148	-0.061029
-0.50189	-0.16961	-0.28384	-0.27692	-0.30446	-0.29451	-0.262
-1.2928	-0.018723	-0.85914	-0.087418	-0.9543	-0.024159	-0.51189
-0.35593	-0.47747	-0.65732	-0.35668	-0.26041	-0.81679	-0.42421
0.46197	0.083141	0.10446	0.26157	0.111	0.10532	-0.13191
-0.032363	0.048714	-0.14018	-0.0473	0.20598	0.083182	0.055289
-0.24974	-0.1827	-0.4853	-0.042886	-0.14307	-0.26686	-0.32282
-0.01194	-0.12906	-0.22406	-0.1435	-0.40479	-0.18422	-0.12002
-0.15238	-0.016795	-0.23462	0.085441	0.017083	-0.14009	0.088143
-0.74444	-0.092738	-0.43249	0.39508	-0.37815	-0.45025	-0.49138
0.60155	0.0318	0.092441	0.20801	-0.29606	0.05639	0.0054322
-0.83626	0.067701	-0.37221	0.13454	-0.20008	-0.14633	-0.41931
-0.47344	-0.04195	-0.72355	0.020838	-0.45429	-0.15719	-1.6055
-0.18092	-0.079879	-0.079724	-0.030751	-0.33223	-0.063005	-0.028037
-0.83111	-0.22655	-0.32653	-0.10851	-0.4848	-0.1965	-0.42072
-0.49815	-0.055897	-0.34441	-0.034734	-0.36318	-0.18204	-0.24395

-0.32833	-0.029107	-0.22808	-0.20616	-0.4604	-0.056294	-0.2552
0.47008	-0.44186	-0.063976	-0.55245	-0.19341	-0.72948	-0.093134
-0.3094	-0.0031736	0.13719	0.27509	0.14238	0.14704	-0.033055
-0.35797	0.013535	-0.12531	0.054302	-0.19305	-0.27675	-0.71706
0.19519	-0.037143	0.0061216	0.091755	-0.096191	0.12577	-0.038582
-0.58429	-0.42909	-0.58844	-0.22608	-0.88205	-0.51614	-1.2219
-0.34122	-0.087236	-0.4326	-0.098192	0.0096965	-0.20415	-0.12234
0.32759	-0.081527	0.056177	-0.016564	0.05016	-0.010819	0.093216
0.010215	0.44362	-0.069351	0.084469	-0.19003	0.04328	-0.19446
-1.5789	-0.36346	-0.47822	-0.49021	-0.82485	-1.0073	-0.5613
0.0079556	-0.059758	0.18915	0.13008	0.29381	0.26881	-0.047464
-1.3121	-0.34731	-0.63921	-0.54723	-0.61808	-0.60984	-0.26654
-0.3026	0.034421	-0.28375	-0.020567	-0.27965	-0.15608	-0.11893
-0.52675	-0.22979	-0.44642	-0.17732	-0.094581	-0.13892	0.032111
-0.10135	0.59696	0.39183	0.41707	0.05904	0.46447	-0.53705
0.051311	-0.38938	-0.33998	-0.08187	0.00641	0.13925	-0.10101
-0.016402	-0.26839	-0.5356	-0.37791	-0.66618	-0.0236	-0.12172
-0.34997	0.14134	-0.079386	0.10635	-0.16954	-0.14152	-0.38624
-0.51198	-0.22959	-0.93416	-0.23704	-0.18966	-0.1677	-0.04704
-0.2744	-0.11218	0.11852	0.24242	-0.032444	-0.12655	-0.13336
-1.3589	-0.56335	-0.71824	-0.38422	-0.55174	-2.2295	-1.2776
0.022465	-0.056224	0.15793	-0.12136	-0.16532	-0.078902	0.090826
-0.706	-0.027459	-0.10739	0.20002	0.066388	-0.1779	-0.30239
-1.345	-0.57575	-0.6114	-0.64465	-0.67121	-1.2018	-0.74547
-0.73216	0.066299	-0.27479	-0.065631	0.25721	0.065599	-0.22707
0.76429	0.16515	-0.071438	0.09483	0.089985	0.085082	0.076942
-0.28313	-0.067056	0.030772	-0.023052	0.032082	-0.098542	0.015993
-0.34927	-0.23529	-0.013186	-0.0864	-0.18764	-0.10607	-0.086074
-1.9753	-0.1418	-0.71013	-0.5146	-0.22893	-0.71008	-0.64921
-2.4164	-0.14423	-0.96074	-0.21432	-1.113	-0.48608	-0.66548
0.65807	-0.040633	0.20399	-0.00068	0.018329	-0.098593	0.26244
0.0012897	-0.1606	-0.19059	0.030145	0.20084	0.092172	0.008276
1.1342	0.23866	-0.10576	0.10628	0.23385	0.1381	-0.052107
-0.16441	-0.19977	-0.97609	-0.13229	-0.60247	0.013979	-0.52759
-0.18165	-0.059966	0.0042057	0.019086	-0.17749	-0.021057	-0.10753
-0.06337	0.062744	-0.017457	0.13085	0.16471	0.15741	0.079787
-0.26285	-0.078573	-0.31799	-0.29401	-0.3703	-0.23039	0.36758
-0.15307	0.16158	0.27192	0.15643	0.21558	0.024517	-0.29502
-0.23915	0.090848	0.2703	-0.17411	0.18259	0.066904	0.22737
-0.20565	0.001525	0.24567	0.2207	0.23766	0.23185	0.14093
-0.39483	-0.1124	-0.16345	-0.13637	-0.17425	-0.24896	-0.16413
-0.13107	-0.087964	-0.3558	-0.22639	-0.0061734	0.17932	0.096967

0.15889	-0.21867	0.14354	-0.16014	-0.12304	-0.15214	-0.062432
0.14848	0.032813	-0.037175	0.041876	0.06795	0.058779	0.1344
-1.214	-0.29614	-0.26618	-0.034388	-0.24095	-0.81672	-1.427
0.023606	-0.0022265	-0.30694	-0.074762	-0.50567	-0.11431	-0.0732
-0.15032	-0.14523	-0.30661	-0.027234	-0.021253	-0.11969	-0.08595
0.46207	-0.0029043	0.07888	-0.14072	0.16401	-0.46923	0.26614
-0.34934	-0.1445	-0.027363	0.085003	-0.30498	-0.10301	-0.70313
-0.32471	-0.058005	0.074471	-0.14825	-0.090475	-0.029903	-0.054994
0.13422	0.068243	0.19177	-0.080311	0.040237	0.060642	-0.13399
0.38005	-0.10944	-0.23451	0.041235	-0.22483	-0.25706	-0.33891
1.1067	0.13949	0.2027	0.12381	0.23659	0.64095	0.72827
-0.31287	-0.1813	-0.30598	-0.076084	-0.29735	-0.48424	-0.099473
0.3955	0.10362	0.19487	0.061977	-0.090424	0.19125	0.4002
0.0614	-0.094429	0.055351	-0.23613	-0.12384	-0.20681	-0.1002
1.1271	-0.051542	0.07892	-0.043747	0.21181	-0.066259	0.26941
0.12189	-0.045172	0.018547	-0.15107	-0.13854	-0.020357	0.13745
-0.69013	-0.28136	-0.31016	-0.097658	0.12425	-0.33555	-0.037001
-0.84695	-0.24755	-0.39312	-0.0042218	-0.36475	-0.81417	-1.2619
-0.90757	0.14572	-0.34979	-0.32885	-0.36559	-0.40585	-0.2509
-0.19382	-0.61627	-0.78096	-0.45818	-0.092428	-0.47431	-0.041298
-0.0050879	0.032389	-0.065399	0.059512	-0.14366	0.18812	-0.1044
-0.92654	-0.38132	-0.23565	-0.271	-0.21004	-0.47496	-0.4357
-0.37597	0.029818	-0.002832	0.069384	-0.17377	-0.066495	0.0076394
1.0173	0.064975	-0.062886	0.089954	0.037078	0.19838	0.22483
0.15531	0.069831	-0.026483	-0.046381	0.12035	0.010306	0.24548
-0.53242	-0.34596	-0.70377	-0.32685	-0.70062	-0.37888	-0.26437
-1.2703	-0.32479	-0.71009	-0.30989	-0.45792	-0.99404	-1.0278
-0.1409	-0.14974	-0.78446	0.030137	0.13026	-0.36547	0.049706
-0.38064	-0.25166	-0.012009	-0.21966	-0.14917	0.073145	0.077875
0.17687	0.24692	-0.06047	0.094412	0.065069	0.0117	-0.081242
0.12221	-0.0091945	-0.3192	0.12075	-0.14022	0.053402	-0.28327
-0.097316	0.0056994	0.040533	-0.022115	0.029167	-0.040969	-0.044907
-0.34092	-0.18164	-0.17268	-0.12282	-0.12945	-0.16066	-0.38323
-0.065569	-0.066624	-0.31305	-0.154	-0.017988	0.16361	-0.072093
-0.19573	-0.12546	-0.30507	-0.41207	-0.24682	-0.18681	0.21653
-0.22735	0.23799	-0.2076	0.090872	0.056153	-0.096028	-0.20192
-0.26284	0.18185	-0.05769	-0.087337	-0.20741	0.14583	0.13297
-0.0011428	0.13073	0.15356	0.28001	0.12102	0.41531	0.22153
-0.72696	-0.12368	-0.31092	-0.24726	-0.27107	-0.2885	-0.12012
0.79205	0.032292	0.23431	0.071596	0.27242	0.12212	0.22749
-0.35747	-0.30154	-0.30638	-0.33626	-0.41102	-0.46021	-0.21173
0.18564	0.065385	-0.041723	-0.086064	0.15767	-0.16913	0.437

-0.25531	0.092695	-0.000812	-0.011299	-0.30697	-0.11228	-0.54056
-0.33701	-0.16533	-0.16457	-0.19097	-0.12562	-0.1012	0.16555
0.36047	0.38566	0.22274	-0.0028578	0.27463	-0.03708	0.21077
-0.57832	-0.20285	-0.038386	0.036419	0.11375	-0.09675	-0.16763
-0.69438	-0.09531	-0.3012	-0.26657	-0.22074	-0.14301	-0.33646
-0.28848	0.041214	-0.18958	-0.018624	-0.13706	-0.46436	-0.64963
-0.21162	-0.086453	-0.11447	-0.19316	-0.37043	0.019773	-0.14222
0.2512	-0.09216	-0.08043	-0.1166	0.083228	0.25928	-0.26239
-0.73021	-0.5022	-0.5157	-0.19791	-0.13774	-0.45439	-0.74496
-0.44181	-0.30842	-0.19375	-0.26904	-0.31346	-0.33832	-0.268
0.00014751	-0.087724	-0.23951	-0.20799	0.069426	-0.036189	0.19015
0.067754	0.072547	-0.26159	-0.038911	0.067296	-0.22704	-0.44582
-0.058681	-0.040507	-0.15221	-0.068607	-0.24971	0.12573	0.033255
-1.3958	-0.41838	-1.0738	-0.29042	-0.18247	-0.23732	-0.23839
-0.29067	-0.15335	-0.034092	0.057491	0.015232	-0.27803	-0.1852
0.15314	0.086072	0.2969	0.21335	0.12757	0.47477	0.34285
-0.53708	-0.056404	-0.4337	0.02767	-0.20271	-0.32229	-0.32828
-2.7214	-1.391	-1.3762	-1.0487	-1.1852	-2.2827	-1.7861
-0.19823	-0.12183	0.0082091	-0.17013	-0.12249	-0.098386	-0.064296
-0.18305	-0.3498	-0.24144	-0.26126	-0.13881	-0.35189	-0.20148

test_GSE439	test_GSE439	test_GSE439	test_GSE439	test_GSE439	test_GSE439	test_GSE439
ref_GSE4394	ref_GSE4394	ref_GSE4394	ref_GSE4394	ref_GSE4394	ref_GSE4394	ref_GSE4394
GSE4394	GSE4394	GSE4394	GSE4394	GSE4394	GSE4394	GSE4394
GEO	GEO	GEO	GEO	GEO	GEO	GEO
cDNA	cDNA	cDNA	cDNA	cDNA	cDNA	cDNA
RIFAMPICIN:	RIFAMPICIN:	RIFAMPICIN:	RIFAMPICIN:	RIFAMPICIN:	RIFAMPICIN:	RIFAMPICIN:
508	509	511	514	515	516	520
-0.23728	-0.23787	0.1387	-0.23853	-0.25792	-1.0504	-0.057412
0.1042	0.0058021	0.066236	-0.12196	0.060688	0.15993	0.061519
0.070013	0.61034	-0.057618	0.031617	-0.11502	-0.11637	0.1851
0.29973	-0.28174	0.04431	-0.062142	0.02198	-0.11273	0.0089126
0.133	-0.24885	0.028392	-0.021109	-0.11621	-0.031856	-0.075229
0.15025	-0.63323	-0.37488	-0.12912	-0.046954	-0.77017	0.0082268
-0.47907	-0.58027	-0.525	-0.73885	-0.80594	-1.2165	-0.40478
0.32866	-0.35664	0.041251	0.050125	-0.0056127	0.46926	-0.10276
-0.084742	0.14207	0.66102	-0.037107	-0.070774	0.0905	-0.17371
-0.23159	-0.027851	-0.050249	0.034531	0.05987	-0.23102	0.12853
-0.030105	-0.75151	-0.54429	-0.19469	-0.065515	0.17314	-0.022602
0.40225	0.056925	0.031068	0.0081557	-0.15547	-0.086945	-0.15104
0.089761	0.2109	0.16384	-0.16013	-0.029426	-0.0002972	-0.010152
-0.0097148	-0.059736	0.09791	-0.3173	-0.34907	-0.30023	-0.049219
0.22866	0.11324	0.093853	-0.01595	0.025662	-0.06852	0.031176
0.077116	0.00073647	-0.11424	-0.10752	-0.02324	-0.33841	-0.018497
0.046784	0.021081	-0.062901	0.055223	-0.048472	-0.30491	-0.075997
0.097812	0.019465	0.088812	-0.024531	0.060062	-0.073583	0.065595
-0.457	0.085552	0.15605	-0.39948	-0.081822	-0.27414	-0.074955
0.19566	0.038702	-0.060378	-0.23473	-0.18434	-0.33647	-0.21949
0.17465	0.2511	0.29676	-0.33184	-0.70485	0.10399	0.025672
0.38796	-0.34146	-0.094569	-0.095348	-0.22571	-0.63971	-0.037122
0.20104	-0.3311	-0.010723	-0.1566	-0.11678	-0.21751	-0.40749
0.058572	-0.27751	-0.092389	-0.051666	-0.059203	-0.31659	0.086549
0.18818	0.25624	0.11932	0.014222	0.019665	-0.24506	0.10593
-0.12819	-0.15497	-0.43197	-0.19709	-0.21803	-0.78257	0.093892
0.0013817	-0.053228	-0.17802	-0.15926	0.059692	0.25882	-0.15363
0.35531	0.54111	0.83279	0.34281	0.29779	0.75167	0.1577
-0.012638	-0.1442	1.111	-0.16834	-0.0004931	-0.26646	-0.28702
-0.06327	-0.38169	-0.34393	0.055164	-0.037441	-0.3099	0.062578
-0.51421	-0.25464	-0.14397	-0.27285	-0.12388	-0.47784	-0.5216
-0.49616	-0.70889	-0.65388	-0.36954	-0.21786	-1.0999	-0.07278

-0.19355	-0.5862	0.064848	-0.19418	-0.22582	-0.0031263	-0.076288
0.30395	0.18057	0.34913	0.029286	0.0041832	-0.06393	-0.02618
-0.33987	-0.28588	0.054033	-0.26512	-0.36186	-0.31685	-0.22564
0.041319	0.13738	0.037132	0.015822	0.01536	0.014195	-0.047612
-0.015457	0.11102	0.17163	-0.090406	0.045708	0.082089	-0.28823
0.53821	1.1295	-0.19831	-0.18346	-0.0097476	0.083638	-0.45288
-0.078225	-0.041168	0.026104	0.26786	-0.05559	-0.055119	-0.11615
0.29226	0.067811	0.31773	-0.090691	0.015661	0.20301	-0.080402
0.05181	-0.38968	0.074643	-0.20104	-0.17267	0.16105	-0.11695
-0.047815	-0.29454	0.043778	-0.33319	-0.15074	0.051631	-0.22657
0.004504	-0.11723	-0.14915	0.11621	0.051275	0.083526	-0.0062521
-0.1415	-0.30636	0.16121	-0.11904	-0.070968	0.17894	-0.34747
0.0049382	-0.021041	0.074759	-0.11197	0.014145	0.1647	-0.32476
0.10526	-0.22759	-0.72654	-0.57129	-0.22162	-1.9473	-0.022571
-0.50091	-0.89626	-1.164	-0.55382	-0.55565	-1.1807	-0.24738
-0.012444	-0.11856	0.073012	-0.0479	-0.17156	-0.016891	-0.18448
-0.38001	0.13871	-0.11091	0.13194	-0.044073	-0.20323	0.11538
-0.17128	-0.0018958	0.33125	-0.12659	-0.013517	0.056093	-0.2803
-0.2947	-0.035196	-0.30764	-0.47072	-0.23612	-0.78865	-0.10884
-0.23394	-0.019726	0.16079	-0.079416	-0.066676	0.08548	-0.051325
-0.10809	-0.31592	-0.1259	-0.040484	-0.072981	-0.44993	0.0043751
0.040083	0.07783	0.14947	0.22384	0.30239	-0.68714	0.0057592
-0.27939	-0.78939	-0.6392	-0.64082	-0.061869	-0.49446	-0.14079
-0.72265	-0.56052	-0.32441	-0.097268	-0.080808	-0.26799	-0.044137
-0.033352	-0.20415	-0.15783	0.19632	-0.18157	-0.12469	-0.029748
-0.0003671	0.3104	0.46316	0.47872	0.27345	0.94034	-0.15682
0.33631	0.45481	0.49932	0.080816	0.11817	0.32357	0.050434
0.044543	0.087852	0.15356	-0.11212	-0.088824	-0.39016	-0.10003
0.67199	0.54819	0.3736	0.27796	0.21539	0.42413	0.078263
-0.0056219	0.38709	0.71432	-0.12603	0.025605	0.084171	-0.30654
0.0012651	0.69876	0.41858	-0.27037	0.061029	0.012505	-0.38441
-0.070054	0.096896	0.27745	-0.17671	-0.029096	-0.26024	-0.18801
0.23907	0.27493	0.13949	0.22384	-0.056753	-0.16032	-0.010651
0.60723	1.0477	0.35981	0.44829	0.44877	0.50915	0.26032
-0.40603	-0.70239	-0.80635	-0.29318	-0.12874	-0.33387	-0.36315
-0.33881	-0.43946	-0.10579	0.47895	-0.035226	0.057324	-0.020779
-0.10897	-0.37195	-0.030875	-0.25922	-0.21781	0.0074235	-0.26102
-0.080765	-0.082547	0.20351	0.09084	0.058318	0.12694	0.0057964
-0.64136	-1.3341	-1.242	-1.8618	-1.2836	-2.795	-0.59254
-1.0392	-0.74065	-0.65447	-1.1944	-0.95276	-1.4228	-0.42926
-0.2981	-0.56766	-0.48823	-0.44934	-0.12422	0.15272	-0.21733
0.56518	0.36966	0.70764	0.29118	0.15744	0.64848	0.23664

0.15305	-0.25688	-0.056214	-0.20675	-0.1943	0.08876	-0.24312
-0.6452	-1.6558	-1.2201	-0.39877	-0.64464	-1.4033	-0.46745
-1.0984	-1.638	-1.4513	-1.4548	-0.78184	-0.78822	-0.56668
0.13166	-0.19978	-0.24418	0.082015	0.017112	-0.11424	0.0026681
-0.050189	0.11063	0.10405	-0.10219	-0.025078	-0.13092	0.0011781
0.39763	0.44673	0.64427	-0.093116	0.0076836	0.26102	-0.23203
-0.25069	0.64851	0.69895	-0.19775	0.067434	0.46705	-0.060475
-0.27167	-0.23808	-0.42687	-0.31635	-0.2752	-1.0094	-0.081791
-0.18636	-0.40043	-0.19263	-0.54753	-0.15473	-0.073051	-0.38564
-1.4582	-0.87755	-0.72604	-0.83249	-0.8282	-0.77196	-0.79722
-0.10879	-0.95223	-0.29175	-0.17492	-0.27009	0.088747	-0.16675
-0.058252	-0.10235	-0.16703	0.0037237	0.22381	0.16287	-0.053787
-0.032023	-0.86715	-0.71798	-0.37953	-0.18132	-0.52432	-0.25087
-0.18775	-0.067569	0.097649	0.19211	0.035247	-0.16192	0.15447
-0.35987	-2.168	-1.6204	-0.44	-0.59087	-1.2007	-0.54204
-0.024722	0.25361	0.11051	0.044597	0.017411	0.2524	0.063148
0.0050618	-0.12649	-0.09014	-0.019019	-0.087515	-0.047558	-0.041853
-0.26159	-0.21607	-0.18245	-0.094112	-0.24983	0.16069	-0.13828
-0.15781	0.21074	0.13887	0.19987	-0.0034765	-0.02365	-0.030159
-0.058226	0.1556	0.018201	-0.15396	-0.045829	-0.27122	-0.23793
0.067683	0.038551	-0.23281	0.00093942	-0.005828	-0.26615	0.0017557
-0.015165	-0.10269	0.15083	-0.17905	-0.13906	-0.3145	-0.16595
-0.089497	0.096604	0.04107	-0.051041	-0.19582	-0.05739	0.0036299
0.10111	-0.19878	-0.13029	-0.18124	-0.10994	-0.35694	-0.068447
-0.0068943	-0.31821	0.080125	-0.10413	-0.12297	0.14136	-0.10353
0.18753	0.23754	0.38838	-0.1766	-0.2928	-0.76557	-0.23793
-0.23614	-0.18118	-0.037415	-0.0090106	-0.13409	0.10409	-0.42308
-0.44386	-0.52423	-0.6674	-0.39937	-0.35006	-0.4863	-0.22485
-0.12175	-0.5854	-0.027737	-0.016867	0.10232	0.23226	0.18605
-0.15604	0.022359	0.0022599	-0.073372	0.058932	0.58849	-0.035143
0.041276	-0.25873	0.18612	-0.19951	-0.061065	0.4981	-0.28431
0.2516	0.57867	0.54679	0.092152	0.21034	0.76206	-0.079468
0.59632	0.010647	0.24553	0.0027993	0.14774	0.31818	-0.32231
-0.10906	0.62939	0.076128	0.065187	0.11902	0.42524	0.2978
-0.4367	-1.1665	-1.3589	-0.1559	-0.33876	-0.28883	-0.24148
-0.50414	-0.59347	-0.16242	-0.30402	-0.27741	-0.72898	-0.2752
0.15831	-0.14449	0.3005	-0.017374	0.11095	-0.01478	0.16197
-1.7148	-2.1109	-1.1054	-0.62837	-0.75191	-1.1418	-0.55373
-0.13297	-0.12702	0.026246	-0.3327	-0.12135	-0.68029	-0.040176
-0.31199	-0.25523	-0.19155	-0.26438	-0.21174	-0.41717	-0.068551
-0.14002	0.55998	-0.047787	-0.37165	0.01438	-0.079218	-0.17218
-0.10429	-0.31306	-0.32444	-0.063673	0.010864	0.21606	-0.010704

-0.12175	-0.24587	0.11338	0.16534	0.037924	0.18193	-0.0053897
-1.0789	-1.0202	-1.1223	-0.63334	-0.41395	-1.007	-0.20136
0.14154	-0.50821	0.30797	-0.099668	-0.22244	0.010955	-0.17935
-0.21481	-0.24483	-0.13935	0.0062608	0.011298	0.4452	0.013269
-0.13818	-0.36336	-0.67177	-0.27617	-0.18144	-0.84629	0.24411
0.39796	0.64671	0.29026	0.36834	0.50277	1.1065	0.24684
0.0015485	-0.3116	-0.12303	-0.14544	-0.018843	0.15918	-0.048443
0.065593	0.048483	0.19426	-0.082241	-0.30404	-0.68959	0.0019392
-0.21378	-0.34899	-0.29786	-0.012548	0.12146	0.19894	0.10286
-0.26826	-0.80672	-0.51571	-0.4204	-0.29445	0.083829	-0.20177
0.085466	-0.000564	0.12503	0.48239	0.26506	0.10387	0.30011
-0.37365	-1.0424	-0.53888	-1.1378	-0.55273	-0.32878	-0.067938
-0.94861	-0.4989	-0.10027	-0.40647	-0.44231	-0.30781	-0.22826
-0.71686	-1.6998	-1.5121	-0.32833	-0.40625	-0.3117	-0.16589
-1.0323	-1.6041	-1.34	-1.3472	-0.7628	-1.7529	-0.83737
-1.2257	-2.1685	-1.8507	-0.39981	-0.50014	-0.73116	-0.45779
-0.19706	0.16956	-0.21907	-0.35596	-0.015776	-0.83997	-0.22543
0.34185	0.21809	0.24055	0.17934	-0.0030648	0.061768	0.38506
-0.56207	-0.37887	-0.46816	-0.83548	-0.17796	-1.0398	-0.28538
-0.10878	-0.1535	-0.14099	0.097349	0.0090741	0.15107	-0.14455
0.00050002	-0.27103	-0.021191	-0.082159	-0.12606	0.13349	-0.064711
-0.037121	0.011962	0.049464	0.054377	-0.14915	-0.027133	-0.034636
NaN	NaN	NaN	NaN	NaN	NaN	NaN
-0.26798	-1.0934	-0.93632	-0.22502	-0.30005	-0.365	-0.18926
-0.046389	-0.0002614	-0.12223	-0.078365	-0.021842	0.00048074	-0.0058606
0.24066	0.43808	0.40783	0.098522	-0.14994	0.69668	-0.27068
-0.18987	-0.34511	-0.33078	0.096254	0.019725	-0.11705	0.097052
0.011904	-0.60167	-0.32273	-0.051557	-0.076111	0.20459	0.044506
-0.5291	-1.2295	-0.57978	0.12397	-0.038938	-0.62426	0.18666
-0.36215	-1.0505	-0.85952	-0.15901	-0.268	-0.095431	-0.11286
-0.88941	-0.24408	-0.10305	-0.094268	-0.13268	-0.28995	-0.040018
0.065468	-0.40863	-0.0813	-0.16939	-0.18171	-0.30413	-0.19988
-0.19753	0.14822	-0.073906	-0.12783	0.0035286	-0.33327	0.044526
-0.080185	-0.1086	0.36992	-0.11996	-0.12728	-0.25584	-0.028306
0.20831	0.18568	0.11403	0.1561	-0.073263	-0.15033	-0.077271
-0.0064631	-0.0071521	0.42662	-0.015924	0.076693	0.098754	0.08525
-0.068404	-0.10853	-0.16007	-0.15219	-0.060405	-0.62799	-0.012993
0.23583	-0.02298	0.11191	0.14171	0.097849	0.0057757	0.030058
0.0022522	0.097011	0.11394	-0.069136	-0.10208	0.15404	-0.062199
-0.19261	-0.093092	0.073346	-0.075429	0.016801	0.12212	0.092392
-0.01351	0.20123	0.045499	-0.065103	-0.047191	-0.56012	-0.0063648
-0.25802	-0.40345	-0.4851	-0.26327	-0.27918	-0.63347	-0.18073

-0.12399	-0.071364	0.14763	0.10098	-0.06372	0.52221	0.016341
0.026267	-0.32729	-0.23253	0.10529	0.17632	0.59281	0.23216
-0.049369	-0.3691	-0.19537	-0.35819	-0.12013	-0.058786	-0.19177
-0.12149	-0.34336	-0.28624	-0.03081	-0.10414	0.1283	0.025839
0.036324	0.22128	0.15419	0.14099	0.16949	0.31877	0.093409
0.11547	-0.059975	0.11804	-0.027208	0.029099	0.23604	0.073249
-0.98914	-0.93069	-0.56451	-0.63048	-0.63989	-0.98337	-0.39842
-0.29851	-0.52361	-1.0086	-0.23766	-0.26533	-1.0493	-0.24685
-0.42909	-2.0956	-1.796	-0.75585	-0.58632	-0.48285	-0.66286
-0.14958	0.53399	0.1172	0.39875	0.010456	0.61036	0.23994
-0.27335	-0.49774	-0.55057	0.1678	0.10711	0.16746	0.052005
-0.17534	-0.42684	-0.44893	-0.13157	-0.075673	0.28473	0.020807
0.20597	-0.3305	0.25163	-0.053647	-0.24322	-0.1392	-0.17994
0.133	0.288	0.27779	-0.083511	-0.061884	0.0553	-0.075646
0.37325	0.53389	0.70935	0.26313	-0.019179	1.2237	0.0017739
-0.18269	-0.11092	-0.13769	-0.2155	0.10545	-0.54202	0.074902
0.39338	0.2639	0.056058	-0.072035	0.17421	0.27099	0.23169
0.55078	0.19888	0.33942	0.0092883	0.023605	0.33775	-0.0012394
-0.659	-0.66543	-1.0014	-0.28697	-0.42983	-1.4033	-0.16024
0.041766	0.33507	0.28818	-0.11763	-0.085303	-0.15892	0.023079
0.080203	-0.036539	0.071545	-0.17613	-0.15182	0.055747	-0.11345
0.084753	0.2109	0.4273	-0.059756	-0.20656	-0.18713	-0.11754
-0.36233	-0.27601	-0.53817	-0.022964	-0.18458	-0.33693	-0.024715
0.12851	-0.083735	0.20256	-0.20884	-0.10496	0.22656	0.04881
0.28383	0.27848	0.48118	0.33836	0.28968	-0.18308	0.053474
0.18645	-0.23586	0.77732	-0.3083	-0.29716	-0.30542	-0.3409
-0.10337	0.055387	-0.2927	0.10946	0.20077	0.27381	-0.036482
-0.3459	-0.48669	-0.40714	-0.21664	-0.25932	-0.76035	-0.089933
0.0061706	-0.19963	-0.62487	-1.1289	-0.52852	-1.6508	-0.077983
-0.9639	0.10593	-0.091652	-0.46954	-0.79471	-0.74975	-0.14107
0.086159	0.23452	0.25375	-0.16123	0.03133	-0.058335	0.061035
0.13734	-0.23155	-0.31531	-0.19224	-0.25334	-0.31345	-0.057257
-0.22827	-0.67511	-0.075765	-0.45738	-0.12466	0.18009	-0.12416
-0.11102	-0.32089	0.27115	-0.48517	-0.11586	0.082891	-0.15757
-0.21813	-0.51541	-0.015841	-0.1652	-0.15923	-0.14544	-0.015424
-0.63953	-0.69498	-0.44492	-0.311	-0.23495	-0.37978	-0.072339
-0.007812	0.26598	0.32141	-0.060375	-0.10247	0.3148	-0.082221
-0.2126	-1.009	-0.59946	-0.2712	-0.27146	-0.37289	-0.18736
-0.25069	-1.0477	-1.2404	-0.67879	-0.59462	-0.97081	-0.71413
-0.042829	0.069722	0.015215	-0.22087	-0.10744	-0.20488	-0.017466
-0.080663	-0.10057	-0.013625	-0.47483	-0.062544	-0.37682	-0.4346
-0.24462	-0.34816	-0.48673	-0.073228	-0.10675	0.20154	0.23922

0.13808	-0.29961	-0.46936	-0.13712	-0.16414	-0.92697	-0.1151
-0.21646	-0.23251	0.059139	-0.31152	-0.15364	0.090666	-0.10969
-0.10402	-0.36071	-0.35495	0.12978	-0.13477	-0.098461	0.29332
-0.38407	0.027006	-0.43913	-0.34642	-0.47386	-0.77058	-0.2106
0.1072	0.004082	-0.031847	0.034695	-0.08966	-0.13625	-0.0011915
-0.66403	-1.3587	-1.3084	-0.80136	-0.56152	-0.11594	-0.29462
-0.26287	-0.52238	-0.26705	-0.13117	-0.25154	-0.30694	-0.020941
-0.25472	-0.0079156	-0.27931	-0.012012	-0.061031	0.1572	0.082412
0.14578	-0.13166	0.25706	-0.083131	0.015409	-0.24755	0.025806
-0.40011	-0.47249	-0.37509	-1.1647	-0.036765	-1.3117	-0.34807
0.40135	-0.018543	0.8815	-0.11222	0.047931	0.2167	0.26627
-0.30977	-0.32801	-0.21299	-0.67091	-0.34843	-1.716	-0.10951
0.11834	-0.29527	0.18187	-0.25343	-0.29645	-0.78974	-0.12979
-0.094345	-0.43176	-0.0038769	-0.21383	-0.19234	0.080595	-0.11772
-0.22148	-0.75847	-0.88206	0.39741	0.35314	-0.20941	0.39671
-0.035198	-0.30751	0.15645	0.14434	0.018053	0.22161	-0.14828
0.23378	-0.0097179	0.3911	-0.50901	-0.45763	-0.2293	-0.16254
-0.37899	-0.1303	-0.276	-0.28837	-0.17791	-0.44787	-0.29573
-0.075333	-0.53087	-0.065343	-0.20296	-0.1897	-0.21125	-0.16642
-0.028875	0.21952	0.017123	-0.09062	-0.048374	-0.26306	0.0036264
-2.0467	-1.5902	-0.96277	-1.1062	-1.0616	-2.1078	-0.56351
0.067781	0.19047	0.2164	0.085983	-0.061826	-0.28809	-0.032756
-0.32116	-0.71409	-0.75123	0.061781	-0.14772	-0.50613	-0.0052801
-0.9839	-0.34103	-0.61908	-1.4118	-0.44584	-1.6337	-0.34857
-0.29652	-0.91225	-0.53073	0.19905	0.0021704	-0.066512	0.16423
0.078483	0.25238	-0.32487	-0.24765	-0.30596	0.054552	0.043657
0.043767	0.12312	0.0081185	-0.10685	0.21078	0.018534	0.10014
0.047124	-0.05515	-0.29233	-0.0089	0.042843	-0.069317	0.022415
-0.73531	-1.3878	-0.56904	-0.22792	-0.24645	-0.43544	-0.39613
-0.35259	-0.70768	-0.64717	-0.36392	-0.29951	-0.90266	-0.43094
-0.019263	0.25668	0.4822	-0.14712	-0.10479	0.46499	-0.067783
-0.088371	0.56304	0.04663	0.17706	0.020296	0.15366	0.17115
0.27262	0.19453	-0.0015159	0.11551	0.046695	0.36556	0.11521
-0.09562	-0.37412	-0.2712	-0.85178	-0.33906	-0.79381	-0.34569
-0.044553	-0.064449	-0.12369	-0.12383	-0.17355	-0.11327	-0.082173
0.085209	-0.24725	-0.025788	-0.01311	0.018776	0.16707	-0.022868
-0.016842	0.31314	0.23664	-0.25483	-0.24001	-0.32752	-0.088492
-0.71901	-0.24575	-0.54458	0.35136	0.41111	-0.52155	0.28243
0.45268	-0.0010151	0.29328	0.062789	0.11902	0.30698	-0.20153
0.22974	-0.26546	-0.1938	0.27898	0.39857	0.46903	0.16039
-0.16591	-0.091045	-0.13986	-0.14303	-0.097631	-0.15303	-0.18627
0.13469	-0.23192	0.23962	-0.12	-0.21699	0.10328	-0.18667

-0.017738	0.098866	0.49718	0.081237	-0.054064	-0.13373	-0.12176
0.04873	0.069706	0.047338	0.1564	0.057697	0.39027	0.049877
-1.2917	-2.0927	-1.9724	-0.3881	-0.19111	-0.33461	-0.14336
-0.079813	0.03917	-0.036168	-0.23046	-0.22894	-0.46275	-0.13552
-0.17303	-0.14492	0.05957	-0.12001	-0.1685	-0.33562	0.032621
-0.54099	0.58106	0.073676	-0.025665	0.0083982	0.076562	-0.16245
-0.12005	-0.46359	-0.86238	-0.77513	-0.45054	-0.21006	-0.035373
0.085289	0.32047	0.18544	-0.00875	0.063515	-0.061759	-0.1102
-0.071912	-0.52808	-0.34733	-0.19462	-0.13542	0.022972	0.056235
-0.24906	0.27777	-0.54916	-0.37431	-0.27725	-0.27418	-0.04243
1.0358	0.58078	0.98951	0.41455	0.2511	0.49274	0.15301
-0.37063	-0.17237	-0.16745	-0.44908	-0.19857	-0.90879	0.08914
0.12902	0.24999	0.17822	0.22639	0.19644	0.42718	0.78563
0.026314	0.076608	0.47284	-0.12376	-0.035438	0.075563	-0.34671
0.076421	0.2717	0.34612	0.12977	0.094542	0.09232	0.23503
0.016688	0.30408	0.1657	-0.10499	-0.13088	0.062365	-0.071979
-0.3326	-0.39839	-0.24825	-0.20317	-0.2245	-0.59429	-0.1722
-1.0841	-0.50292	-0.56336	-0.72923	-0.7098	-1.2978	-0.27038
-0.41944	-0.47683	-0.3264	-0.094276	-0.25094	-0.88796	-0.035138
-0.38299	-0.51544	0.0064546	-0.21225	-0.18258	-0.067495	-0.23065
0.3743	-0.19776	-0.060737	-0.22096	-0.37148	-0.41666	-0.1101
-0.45398	-0.54947	-0.51286	-0.22164	-0.31102	-0.46405	0.013617
-0.21044	-0.14753	-0.033415	-0.0074095	0.0045365	-0.52311	0.16781
0.32552	0.41629	0.80005	0.088944	-0.14829	0.59238	-0.006995
-0.129	-0.021473	-0.095696	0.0066361	0.049239	-0.0068955	-0.053186
-0.23666	-0.22996	-0.27041	-0.41013	-0.15757	-0.65591	0.0063533
-1.1867	-0.78052	-0.75843	-0.85364	-0.63122	-0.92815	-0.55632
-0.17868	-0.25326	0.084376	-0.27577	-0.33305	-0.21392	-0.27057
-0.099318	-0.13999	-0.11499	-0.018463	0.012418	0.20867	-0.24439
-0.15681	0.64242	0.3372	0.045943	-0.07603	-0.2001	0.049969
0.037301	-0.41827	-0.50738	-0.2885	-0.4387	-0.6284	-0.369
0.32448	-0.042937	0.39243	-0.071002	0.036887	0.02889	-0.17996
0.15791	-0.10293	0.090342	-0.1571	-0.029294	-0.31782	-0.382
0.25725	-0.030975	0.21093	-0.061981	-0.17233	0.16574	-0.23479
-0.16691	0.0038578	0.034624	-0.23958	-0.21082	-0.67687	-0.10444
0.071842	-0.42278	-0.16255	0.21398	0.057093	-0.17569	-0.044995
0.028892	0.039429	0.16987	-0.048713	-0.06465	-0.068662	2.19E-05
0.30732	0.24239	-0.21405	0.24382	0.29196	0.46568	0.085203
-0.30553	-0.11062	-0.39965	-0.045526	-0.13498	-0.29093	-0.060055
0.12847	0.31008	0.69457	-0.028085	0.098321	0.51672	0.081674
-0.15212	-0.015296	0.0087528	-0.36756	-0.20843	-0.46667	-0.27683
0.091077	0.19874	0.060856	0.16721	0.18444	0.41946	0.13534

-0.11229	0.26782	0.19723	-0.44481	-0.23623	-0.55577	-0.25623
-0.13989	-0.025395	0.11787	0.023198	-0.047823	-0.25879	0.019867
0.035585	0.037927	0.039744	0.18852	0.084599	0.42493	0.1374
-0.21295	-0.7394	-0.63146	-0.014833	-0.054332	-0.62163	-0.030346
-0.059892	-0.74731	-0.029026	-0.18947	-0.20262	-0.11881	-0.49605
-0.38595	-0.7917	-0.83829	-0.50762	-0.34062	-0.092076	-0.17917
0.004667	-0.038855	0.34366	-0.046497	-0.063301	0.002449	-0.30453
0.020278	0.11661	0.78308	-0.019765	0.014133	0.2611	-0.30946
-0.53059	-0.35085	-0.48562	-0.69963	-0.81914	-1.1363	-0.19813
-0.4179	-0.11859	0.047251	-0.33477	-0.21835	-0.50284	-0.5449
0.032116	-0.37764	-0.18302	-0.17332	-0.22584	0.3751	-0.17863
-0.16624	-0.74131	-0.17135	-0.68217	-0.073961	-0.41872	0.078545
-0.020204	0.092761	0.098925	-0.053932	-0.12788	-0.27854	-0.11648
-0.072038	-0.58543	-0.28534	-0.32906	-0.44966	-0.67793	-0.44442
-0.23039	-0.32133	-0.31335	-0.3251	0.011816	0.042635	0.053046
0.10529	0.011237	0.0081869	0.34113	0.098982	0.078279	0.071475
-0.37717	-0.31952	-0.1277	-0.51474	-0.35108	-0.65135	-0.12837
-2.8603	-2.044	-1.6123	-2.6817	-1.9462	-2.8439	-0.82529
0.047178	0.21938	0.48646	-0.19897	-0.095574	-0.094443	-0.27728
-0.12368	-0.3913	-0.49286	-0.13323	-0.13373	-0.023065	-0.33589

test_GSE439	test_GSE439	test_GSE439	test_GSE439	test_GSE439	test_GSE439	test_GSE439
ref_GSE4394	ref_GSE4394	ref_GSE4394	ref_GSE4394	ref_GSE4394	ref_GSE4394	ref_GSE4394
GSE4394	GSE4394	GSE4394	GSE4394	GSE4394	GSE4394	GSE4394
GEO	GEO	GEO	GEO	GEO	GEO	GEO
cDNA	cDNA	cDNA	cDNA	cDNA	cDNA	cDNA
RIFAMPICIN:	RIFAMPICIN:	RIFAMPICIN:	RIFAMPICIN:	RIFAMPICIN:	RIFAMPICIN:	RIFAMPICIN:
521	522	523	524	525	526	527
-0.1559	-0.11646	-0.26312	-0.14253	-0.33551	-0.53371	-0.69694
0.064492	0.040947	-0.053936	0.20738	0.23122	0.11106	0.13535
-0.097718	0.14425	-0.023746	-0.39551	0.12356	-0.2871	0.054542
-0.097554	-0.15694	-0.023016	-0.28796	-0.1682	-0.18507	0.26653
-0.075038	0.098332	0.095307	-0.17341	-0.0047865	-0.089995	0.17384
-0.11453	-0.11395	0.076565	-0.47828	-0.38113	-0.48028	-0.92156
-0.77735	-0.87172	-0.65212	-0.9699	-1.0247	-1.147	-0.031828
0.017717	-0.11596	-0.12811	-0.2276	-0.062108	0.032723	0.079403
-0.12425	-0.079423	-0.0086222	-0.18788	-0.12403	-0.091527	0.49187
0.094185	0.0423	0.11479	0.028801	0.17373	0.0040449	-0.47372
-0.10787	0.064366	-0.10745	-0.10487	-0.31231	0.096307	-0.033496
0.011035	-0.10187	-0.13621	-0.069309	-0.021773	-0.016114	0.31817
-0.13799	-0.066459	-0.10969	-0.16831	-0.02686	0.064597	0.29477
0.10852	-0.23524	-0.085418	-0.10641	0.18356	0.17956	-0.26889
0.10262	-0.0064546	-0.2185	0.034129	0.15744	0.18885	0.52893
-0.12848	-0.045334	-0.17428	-0.20635	-0.0024713	-0.25037	-0.04544
-0.12076	-0.019577	-0.15831	0.014724	-0.19711	-0.19229	-0.46029
-0.10302	0.06203	0.11562	0.046987	-0.084973	-0.023227	0.48793
-0.18352	-0.23968	0.012752	0.25521	-0.14829	-0.035238	-0.41941
-0.0005451	-0.34315	-0.029621	-0.16362	-0.020684	-0.064878	-0.32109
0.38889	0.23125	0.16655	0.1777	0.37718	0.12315	-0.64814
-0.057392	-0.12443	-0.010728	-0.37637	-0.4273	-0.56835	-0.08689
-0.21535	-0.34065	-0.18632	-0.61799	-0.41444	-0.53449	0.18956
-0.019955	-0.056512	0.047555	-0.074273	-0.10024	-0.053779	0.17761
0.094716	0.10639	-0.11247	0.1097	0.088046	-0.069246	-0.29124
-0.26011	0.04624	-0.31151	-0.30097	-0.45316	-0.56059	0.090897
-0.11406	-0.078129	-0.23349	-0.062403	-0.27709	-0.17774	0.044116
0.29998	0.11077	0.23323	0.45859	0.42645	0.50263	0.73692
-0.3501	-0.40359	-0.30004	-0.056374	-0.37598	-0.32844	0.3457
-0.20866	0.032518	-0.020834	-0.37606	-0.5251	-0.31384	-0.026647
-0.49007	-0.44769	-0.38992	-0.23848	-0.6202	-0.88535	-0.48217
-0.1559	-0.18582	-0.13149	-0.69822	-0.39021	-0.58589	-0.46738

-0.19469	-0.33413	-0.0030186	-0.19688	-0.074501	-0.26081	0.0014935
0.24237	0.026405	0.13351	0.19247	0.11718	0.28031	0.013044
-0.19593	-0.24516	-0.15392	-0.3207	-0.34385	-0.38022	0.082172
0.10458	0.087053	0.094009	0.25618	0.035197	0.1083	0.46581
-0.30885	-0.34113	-0.13995	-0.012825	-0.40285	-0.42481	0.37001
0.12821	-0.30171	-0.03674	-0.14265	-0.20056	-0.019454	-0.18115
0.13874	-0.093311	-0.0073535	0.13902	0.21251	0.22735	0.16979
0.18907	0.017916	0.011449	0.23905	0.13004	0.33252	-0.067874
-0.19935	-0.233	-0.26623	-0.063474	0.0074993	-0.015047	0.062851
-0.16623	-0.19997	-0.30659	-0.1537	0.01124	-0.10149	-0.16187
0.15563	-0.065039	-0.065069	0.095437	0.14569	0.065863	-0.062109
-0.28852	-0.26098	-0.12008	-0.44434	-0.47017	-0.39734	-0.33093
-0.13377	-0.24908	0.058098	-0.026526	-0.26522	-0.073186	0.79469
-0.4208	-0.2349	-0.27801	-0.20962	-0.68642	-1.2065	-1.2126
0.037911	-0.29469	0.13991	-0.76803	-0.22887	-0.12579	0.060633
-0.3627	-0.16689	-0.41872	-0.21671	-0.59042	-0.63144	0.022381
0.11696	0.054361	-0.16681	0.30821	0.098914	-0.079676	-0.25262
-0.16156	-0.27057	0.02574	-0.046934	0.05065	0.58718	1.2692
-0.085257	-0.17366	-0.089098	-0.058246	-0.26288	-0.083377	-0.14028
0.00065519	-0.091388	-0.013855	0.048468	0.22594	0.25925	0.33431
-0.33352	-0.13945	-0.23046	-0.1869	-0.20746	-0.20145	-0.10923
0.15472	-0.018146	-0.082323	0.1134	-0.092901	0.095543	-0.088153
-0.13893	-0.22882	-0.20374	-0.16649	-0.46079	-0.37484	-0.77302
-0.19393	-0.31325	-0.12425	-0.51471	-0.38692	-0.38799	-0.75506
-0.1199	-0.024165	-0.2471	0.037262	-0.18788	-0.13432	-0.34566
-0.0047377	0.014881	0.098599	0.32406	0.30899	0.13335	0.54972
0.30207	0.2502	0.11835	0.2246	-0.06265	-0.1629	-0.026204
-0.075801	-0.05892	-0.084168	0.0026887	0.053598	-0.08138	0.015824
0.025961	0.015849	-0.09402	0.14762	-0.076628	-0.04408	0.24829
-0.26468	-0.13912	-0.12868	0.19716	-0.39956	-0.16004	0.43987
-0.019573	-0.25379	-0.020704	-0.078653	-0.11147	0.083247	0.024159
-0.23903	-0.27781	-0.12168	-0.29068	-0.38423	-0.40224	0.12502
0.20073	0.012656	0.038065	0.081081	0.049529	0.18941	-0.21866
-0.088126	0.44102	-0.16339	0.76194	0.060338	-0.094222	0.12207
-0.21946	-0.34556	-0.35501	-0.72202	0.076156	-0.15735	-0.27423
-0.21272	-0.29565	-0.016564	-0.5201	-0.32013	-0.37872	0.092459
-0.20583	-0.29717	-0.12391	-0.15311	-0.096092	-0.0392	0.20899
0.22619	0.032919	-0.0028486	0.01178	0.19166	0.2907	0.14222
-0.87809	-0.87885	-0.77106	-1.1386	-1.4628	-1.7757	-1.8849
-0.42499	-0.76032	-0.26119	-1.2277	-0.58888	-0.66636	-1.2113
-0.24972	-0.42923	-0.14469	-0.35051	-0.35944	0.1386	0.0093913
0.27571	0.44177	0.044479	0.55213	0.48925	0.47911	0.41551

-0.073837	-0.091582	-0.011212	-0.11417	0.047755	0.26229	-0.0413
-0.38272	-0.60291	-0.30106	-1.2422	-0.81917	-0.957	-0.74975
-0.50985	-0.71901	-0.43413	-0.87707	-1.4186	-0.79936	-0.88353
-0.028198	0.066448	0.059261	-0.038791	-0.019637	-0.011101	0.19942
0.060167	-0.12592	0.065831	0.080979	0.18404	0.090363	0.339
-0.20065	0.19357	-0.044694	0.19993	-0.0031437	0.058125	-0.14973
0.11212	-0.099008	-0.054055	-0.0090769	0.024182	0.15977	0.24352
-0.18272	-0.14912	-0.10516	-0.25479	-0.2556	-0.44238	-0.43525
-0.35135	-0.27739	-0.53423	-0.20867	-0.3307	0.16614	-0.14882
-0.98667	-1.1609	-0.77949	-1.323	-1.2193	-1.1649	-0.17601
-0.19225	-0.33828	-0.23663	-0.61027	-0.16216	-0.33093	-0.17804
-0.16	0.14784	-0.061601	0.17997	0.012837	0.069684	-0.31297
-0.15279	-0.40763	-0.36987	-0.50813	-0.30799	-0.20539	-0.43979
0.1503	0.036995	-0.024713	0.067433	0.040177	-0.092175	-0.34485
-0.51408	-0.91215	-0.57236	-1.6639	-1.1246	-1.2253	-0.84224
0.076678	-0.010477	0.19058	0.098956	0.13771	0.41475	0.70547
0.0083187	-0.063568	0.05196	-0.0025431	-0.13789	-0.18805	-0.31811
0.079032	-0.1886	0.0063447	-0.20921	-0.012495	0.38436	0.43293
0.37279	-0.0006495	0.069197	0.020868	-0.1536	-0.12139	-0.33726
-0.12073	0.0053961	0.12999	-0.17474	-0.094927	-0.23994	0.29385
-0.0002853	0.077657	-0.097184	-0.043856	-0.18386	-0.33012	0.31223
-0.026835	-0.1763	-0.02291	-0.26693	-0.20153	0.0039814	0.50742
-0.26196	-0.036051	-0.081811	-0.24125	-0.23423	-0.39452	-0.63627
0.028309	-0.13419	0.0043554	0.020701	-0.034795	0.15911	0.29936
-0.041323	-0.20899	0.047604	-0.26529	-0.0049016	0.21563	0.38256
-0.42347	-0.31391	-0.075149	-0.26889	-0.024009	-0.34139	-0.2976
0.035489	-0.2266	-0.24027	-0.11137	-0.16219	-0.30674	-0.31342
-0.114	-0.33421	-0.0040195	-0.86331	-0.78651	-0.96414	-0.98997
-0.060362	0.12369	-0.026917	-0.10674	-0.12748	-0.14118	-0.32604
0.22657	0.093832	-0.11713	-0.19597	0.19924	0.0015876	0.13006
-0.36541	-0.28906	-0.51881	-0.558	-0.58525	-0.65539	-0.073071
0.024007	0.11617	0.093767	0.33928	0.16227	0.39718	0.15536
0.11951	0.064235	0.1839	0.0047487	0.15001	0.0051779	-0.0399
0.024626	0.033002	0.19051	0.41424	0.082952	-0.11655	-0.6028
-0.54582	-0.44997	-0.51658	-0.82614	-1.1695	-0.99094	-0.63254
-0.47147	-0.31537	-0.30726	-0.94721	-0.46529	-0.8203	-0.29622
0.21038	0.077285	0.14117	-0.18404	0.25966	0.16599	0.20069
-0.71211	-1.2082	-0.60744	-1.6459	-0.93138	-1.0514	-0.84055
-0.039206	-0.10164	0.0331	0.007376	-0.079115	-0.15765	-0.18629
-0.23006	-0.1444	-0.24316	-0.23476	0.098553	0.12733	-0.22437
-0.36989	-0.27599	0.029077	-0.26488	-0.28512	-0.39548	-0.51309
-0.12341	-0.189	-0.095889	-0.54919	-0.067947	0.017958	-0.087477

-0.013706	-0.089012	0.12611	-0.12243	0.053828	0.0043213	0.14307
-0.22492	-0.37986	-0.23794	-0.97119	-0.60859	-0.50872	-0.9164
-0.094487	-0.27052	0.051101	-0.14264	0.066542	-0.20787	0.039646
0.084571	0.055037	-0.023316	-0.21338	0.037602	0.058217	0.26933
-0.33693	-0.08846	-0.081764	-0.05621	-0.16813	-0.49917	-0.76755
0.16012	0.16479	0.037078	0.44436	0.60641	0.32962	0.20051
0.048538	-0.14083	-0.090908	-0.075355	0.24162	0.097782	-0.03127
-0.18779	0.11495	-0.13604	-0.15586	0.045834	-0.35314	0.21745
0.093104	-0.0253	-0.045069	-0.14597	-0.056767	-0.081303	0.098421
-0.35206	-0.53263	-0.2698	-0.55342	-0.31565	0.07637	-0.094837
0.17363	0.20881	0.094727	0.20963	0.060389	-0.05261	0.17699
-0.20733	-0.45369	-0.30345	-0.40786	-0.62169	-0.21613	-0.16297
-0.23228	-0.3926	-0.31447	-0.59547	-0.94875	-1.2045	-0.54886
-0.16426	-0.38228	-0.3174	-1.1562	-0.75638	-0.78769	-0.74231
-0.67976	-1.0479	-0.71673	-1.5849	-0.87339	-0.92357	-1.5068
-0.49044	-0.75093	-0.52731	-1.2647	-1.0352	-1.0308	-0.59472
-0.21867	-0.13673	0.010368	-0.37486	-0.23028	-0.56669	-0.55012
0.18587	0.064389	-0.10014	0.21977	0.03474	0.33074	0.15731
-0.89364	-0.31846	-0.69151	-0.2793	-0.50347	-0.49817	-0.79052
-0.44989	-0.22182	-0.49275	-0.084926	-0.67849	-0.41465	-0.50138
-0.10863	-0.089269	-0.12212	-0.019606	0.19567	0.20032	-0.030666
0.011129	-0.082791	-0.037243	0.045377	0.0031025	0.37864	0.40537
NaN	NaN	NaN	NaN	NaN	NaN	NaN
-0.03467	-0.1467	-0.13027	-0.44198	0.17489	0.13018	-0.019445
-0.23859	0.10562	-0.087619	-0.12238	-0.22844	-0.038311	0.20078
-0.064201	-0.1794	-0.022611	0.15013	-0.20959	0.17429	-0.17291
-0.02317	-0.024885	0.024931	-0.018779	-0.21067	0.061164	-0.18214
-0.062999	0.096981	-0.26465	-0.17321	-0.010909	-0.10335	-0.24046
0.088737	0.24495	-0.059589	-0.61485	-0.14434	-0.42713	-0.40681
-0.30206	-0.37192	-0.15992	-0.71087	-0.54412	-0.77778	-0.73702
-0.16258	-0.11633	0.0845	-0.37065	0.053399	-0.29986	0.16099
-0.09795	-0.19745	-0.012025	-0.20126	0.021144	0.018758	0.12715
0.16171	0.17105	0.016428	0.080197	0.03634	0.050466	-0.42977
-0.035704	0.07476	-0.1989	-0.022635	0.018074	0.031636	0.17749
0.092098	0.059243	0.12743	-0.19157	0.002313	-0.19189	-0.029276
0.15215	0.13882	0.078252	0.089442	0.10477	0.16669	0.30331
0.2775	0.1695	0.34651	-0.14194	0.02784	-0.31876	-0.47298
-0.025915	0.10748	0.19096	0.034057	-0.027152	-0.23356	0.32931
-0.0009085	-0.069884	0.065664	-0.051661	0.15237	0.22044	0.29805
0.21081	-0.15403	0.082923	-0.13475	-0.022855	-0.072357	-0.072254
0.053212	0.004449	-0.0019711	0.15426	0.032446	0.23136	-0.015939
-0.44059	-0.36214	-0.31083	-0.41369	-0.76472	-0.7829	0.22864

0.009111	0.045892	-0.048491	0.13773	0.29202	0.29941	0.29806
-0.20214	-0.19627	-0.099267	-0.073778	0.1409	0.22269	-0.0031011
-0.071367	-0.22432	-0.26519	-0.021466	0.022547	0.047356	-0.10954
-0.037714	-0.021218	-0.16355	0.16928	0.030728	-0.1444	-0.32225
0.12497	0.067837	0.12316	0.23456	0.23013	0.42814	0.45638
0.085387	0.073621	0.18724	0.066743	0.084432	0.23164	0.32358
-0.62577	-0.67614	-0.45323	-1.1701	-1.0869	-1.271	-0.61994
0.015459	-0.27072	0.070371	-0.36442	-0.1299	-0.033398	-0.39356
-0.40023	-0.97454	-0.252	-1.315	-0.98809	-0.91938	-0.46352
0.16745	0.15978	0.14817	0.17996	0.29213	0.50514	0.43976
0.10667	0.066656	0.05362	-0.011169	0.011138	-0.15107	0.031571
-0.30344	-0.19137	-0.097537	-0.24223	-0.080199	-0.77262	0.030498
-0.20082	-0.12939	-0.18708	-0.14438	-0.10761	-0.16697	-0.10373
0.034604	-0.0030443	0.11073	-0.22656	0.2141	0.19131	0.097479
0.079491	-0.0054037	0.053215	0.34868	0.27432	0.72394	1.0003
0.024991	0.080578	-0.020756	0.065107	0.058009	0.087061	-0.30434
0.32939	0.018584	-0.056886	0.22228	0.21593	0.028862	-0.31264
0.090692	0.054564	0.16398	0.14692	0.11346	0.11726	0.023805
-0.21545	-0.11514	-0.21776	-0.46248	-0.76384	-1.0163	-0.86472
0.21989	0.058625	0.062435	-0.055839	0.010031	-0.044728	-0.35893
0.029552	-0.08769	0.03025	-0.10936	0.13329	0.083444	0.32351
0.084699	-0.01245	-0.047704	0.074804	0.18942	0.24839	0.27809
-0.25378	-0.25668	-0.28097	-0.34225	-0.43204	-0.38994	0.11413
-0.11698	-0.16317	-0.11095	-0.10871	0.0485	0.37089	0.034537
0.21037	-0.094881	0.17304	0.066021	-0.10688	0.04104	0.087202
-0.30263	-0.30923	-0.30918	-0.025625	0.043781	0.25169	0.30262
-0.014112	0.099068	0.085642	0.096311	0.12291	-0.041205	-0.314
-0.17292	-0.26647	-0.17609	-0.43158	-0.3987	-0.60764	-0.3064
-0.48701	-0.2145	-0.35576	-0.19353	-0.36388	-0.5765	-0.84103
-0.17742	-0.40417	-0.17215	-0.3207	-0.12637	0.14616	-0.47373
0.26224	-0.063089	0.23796	0.014129	0.11922	0.070547	-0.0089103
0.050792	-0.14552	-0.1444	-0.2283	-0.20241	0.097524	-0.34075
-0.18915	-0.26214	-0.35718	-0.21739	0.067908	0.014404	0.23838
-0.17301	-0.39778	-0.30653	-0.071906	0.010589	0.015238	0.0059376
0.033485	0.05633	-0.032437	-0.16716	-0.21913	-0.14879	-0.11306
-0.20007	-0.28132	-0.35343	-0.64578	-0.58215	-0.77209	-0.22224
-0.025858	-0.20051	-0.053473	0.28675	0.14516	0.33626	0.22172
-0.078913	-0.32768	-0.10718	-0.75406	-0.35545	-0.44649	-0.23209
-0.35179	-0.92744	-0.44069	-1.0559	-0.40563	-0.55961	-1.1359
-0.20465	-0.055317	-0.0011639	0.085397	0.054331	0.11404	-0.029569
-0.11112	-0.29413	-0.14439	-0.12343	-0.54678	-0.22712	0.38815
0.072166	-0.14311	-0.063401	-0.15769	0.38373	0.37937	0.46979

0.053387	-0.16865	-0.13588	-0.16602	-0.19974	-0.044433	-0.23786
-0.027962	-0.17756	-0.17607	0.067635	-0.12169	0.35949	0.19458
0.044758	0.18718	-0.096074	-0.42748	-0.21797	-0.31493	-0.41881
-0.20258	-0.27198	-0.27801	-0.3272	-0.053466	-0.020682	-0.46036
0.0054432	-0.032152	-0.086289	0.14379	-0.045588	-0.11151	0.04917
-0.35538	-0.65946	-0.44411	-1.1049	-0.52893	-0.12448	-0.072638
-0.30754	-0.23926	-0.27458	-0.23579	-0.24622	-0.23603	-0.30734
-0.053388	-0.052884	-0.098766	0.0057811	-0.19077	-0.066698	-0.51364
0.078949	0.077928	-0.062434	0.11047	-0.1017	-0.28117	0.35223
-0.33697	-0.42979	-0.35399	-0.36991	-0.6709	-0.73502	-0.65806
-0.0061388	-0.0063191	0.18848	0.12289	0.035499	0.20318	-0.16975
-0.52399	-0.34379	-0.40454	-0.4289	-0.18913	-0.72349	-0.69833
-0.011676	-0.080415	0.045758	-0.12866	0.085284	-0.091075	0.18515
-0.11347	-0.31424	-0.020602	-0.32965	0.025754	-0.0059396	0.18747
0.21367	0.12943	0.063386	-0.081639	0.12913	-0.40236	-1.0895
0.038336	0.032589	-0.06504	0.12644	0.019864	-0.010204	-0.17142
-0.48383	-0.25031	-0.34632	-0.1664	-0.27774	-0.081212	0.28622
-0.10756	-0.16655	-0.21395	-0.27935	-0.29504	-0.31654	-0.91872
-0.19257	-0.31568	-0.18048	-0.32512	0.1291	-0.050098	0.2087
0.07937	0.068669	-0.078344	-0.069473	0.12306	0.23025	-0.27764
-0.14802	-0.66185	-0.25136	-1.7535	-0.65392	-0.72097	-1.2084
0.12906	0.013695	0.069346	0.22358	0.10945	0.24133	0.12669
-0.03826	-0.11802	0.11436	-0.44808	-0.30994	-0.65561	-0.47097
-0.45202	-0.42303	-0.37732	-0.67188	-0.6307	-0.48212	-0.72486
-0.070517	0.11986	-0.11249	-0.31134	-0.12394	-0.3737	-0.24948
-0.067131	-0.38198	-0.25725	0.01779	0.20989	0.19441	-0.25972
0.14012	0.12976	-0.0006736	-0.00121	0.024299	-0.49916	0.073483
-0.049413	0.093887	-0.23124	-0.12293	-0.074433	-0.080419	1.2931
-0.18392	-0.40131	-0.14513	-0.99032	-0.2383	-0.33932	-0.022421
-0.30703	-0.49499	-0.31852	-0.75708	-0.52757	-0.4206	-0.77101
-0.18326	-0.24224	-0.17268	0.067233	0.16169	0.21618	0.043394
-0.1801	0.081209	-0.13471	-0.22901	-0.13103	-0.2141	-0.039675
0.18507	0.1602	-0.010115	-0.040395	0.15679	0.12631	0.06381
-0.34818	-0.35134	-0.52454	-0.36041	-0.30815	-0.63508	-1.0664
0.0020566	-0.067763	0.018776	-0.13319	-0.038261	-0.22623	0.2146
0.07231	-0.036493	0.05533	-0.09575	0.0087395	-0.011368	-0.11692
-0.1608	-0.19808	-0.26471	0.047335	-0.04314	0.024244	0.20871
0.19466	0.15266	0.087928	-0.10922	-0.30216	-0.53797	-0.80437
-0.015641	0.13753	0.15253	0.15067	0.083717	-0.1601	-0.019241
0.073467	0.20185	0.16038	-0.040375	0.081764	-0.36905	-0.0075935
0.048537	-0.11555	-0.0091361	-0.080797	0.043522	-0.073711	-0.21531
-0.090689	-0.20604	0.035096	-0.18134	0.094316	0.28131	0.22993

0.065929	0.090942	-0.10595	-0.16098	-0.13182	0.061739	-0.093351
0.15621	0.039205	0.10432	0.090173	0.13906	0.23674	0.39464
-0.24575	-0.42569	-0.35632	-1.3097	-1.1368	-1.2248	-0.78626
-0.029828	-0.11802	-0.18602	-0.21851	-0.18902	-0.29985	-0.12128
0.16421	0.059023	-0.014051	-0.099232	0.073362	0.065477	-0.29333
0.16174	0.07028	0.22266	0.3857	-0.10132	-0.095059	-0.33933
-0.12777	-0.30787	-0.16736	-0.24458	-0.36588	-0.022191	-0.5848
-0.030197	-0.0088165	-0.040303	0.11212	-0.12827	-0.081396	0.38944
-0.091208	-0.12928	-0.07931	-0.29244	-0.044689	-0.1459	-0.18156
-0.20848	-0.24227	-0.1036	-0.52927	-0.049634	0.054372	-0.24402
0.24773	0.38748	0.23453	0.8371	0.50966	0.31165	-0.20948
-0.20896	-0.050214	-0.054194	0.045294	-0.23414	-0.3074	-0.61542
-0.027848	0.059466	0.010808	-0.17631	0.40831	-0.27363	-0.11312
-0.077176	-0.17409	0.006427	-0.086453	-0.088721	-0.099276	0.63201
0.26079	0.271	-0.062218	0.43251	0.34877	0.46317	0.036933
0.025716	0.049345	0.038065	-0.074614	0.13505	-0.039727	0.097224
-0.24006	-0.15297	-0.11227	-0.34944	0.020106	0.011781	0.01895
-0.067023	-0.34553	-0.1705	-0.94245	-0.22471	-0.37746	-0.75496
-0.2975	-0.076061	-0.2148	-0.41796	-0.21045	-0.51383	-0.23831
-0.36757	-0.011403	-0.23093	-0.16178	-0.18568	0.11564	0.10235
0.043312	-0.20062	-0.01714	-0.31282	-0.22669	-0.38617	-0.24968
-0.23002	-0.32853	-0.11376	-0.61626	-0.35934	-0.44042	-0.01278
0.16875	0.12214	0.10302	-0.044985	-0.010056	-0.19589	0.074627
-0.055216	0.19151	-0.016892	0.17983	0.25534	0.32069	0.46859
-0.022906	0.029734	0.0098379	-0.020993	0.060273	-0.15394	0.12423
-0.010956	-0.12049	-0.1552	-0.32575	-0.13012	0.13799	-0.044843
-0.41719	-0.62829	-0.19297	-0.66522	-0.4177	-0.40198	-1.1728
-0.2709	-0.2836	-0.34029	-0.42279	-0.15035	-0.29054	0.065424
-0.07632	-0.19112	0.058107	-0.31364	-0.15065	0.073357	0.50463
0.20308	0.15902	0.13315	0.16211	0.030234	-0.11022	-0.43911
-0.050352	-0.22825	0.0074557	-0.55039	-0.20253	-0.33025	-0.53018
-0.18591	0.0071812	-0.010472	0.078257	-0.16132	-0.0092692	0.036653
-0.13017	-0.18505	-0.16273	0.28928	-0.37933	-0.51129	0.11572
-0.12092	-0.11453	-0.0084321	-0.094345	-0.012974	-0.070961	0.16996
-0.21416	-0.18856	-0.15969	-0.27597	-0.37137	-0.094998	0.10011
0.0113	0.021815	-0.020091	-0.36028	-0.42282	-0.37783	-0.41257
0.14332	0.033651	-0.075753	-0.0040648	0.15781	0.23469	0.64131
0.13173	0.10143	0.14511	0.19577	-0.036714	-0.12216	-0.05009
-0.16576	-0.33097	-0.21816	-0.22895	-0.65249	-0.31404	-0.23954
0.027825	-0.065569	-0.01426	0.16607	0.3028	0.55045	0.39067
-0.27505	-0.27312	-0.08385	-0.09088	-0.32064	-0.40293	-0.0051472
0.18057	0.13705	0.057226	0.083417	0.20577	0.40004	0.99786

-0.15702	-0.20675	0.19672	-0.16286	-0.13811	-0.070763	0.35003
-0.21042	-0.047445	0.013753	0.0063002	-0.013884	-0.27851	0.36339
-0.041055	0.27346	0.12663	0.30627	0.34081	0.1721	-0.16804
-0.060984	-0.10617	0.043396	-0.35624	-0.21907	-0.23362	-0.13334
-0.46768	-0.47305	-0.18926	-0.37159	-0.52984	-0.43043	-0.064297
-0.22542	-0.26387	-0.30331	-0.78396	-0.31949	-0.22282	-0.26159
-0.10302	-0.23097	-0.02023	0.02998	-0.14898	-0.0019765	0.30731
-0.12842	0.56538	-0.099056	0.24418	0.083131	-0.179	-0.19387
-0.085323	-0.47923	-0.21798	-0.62454	0.0031025	-0.1962	-0.75186
-0.19042	-0.34659	-0.17279	-0.46361	-0.5241	-0.41409	-0.20678
-0.043507	-0.04472	-0.15575	0.0527	0.22264	0.29142	0.27595
0.037735	-0.011215	-0.077547	-0.39974	-0.063571	-0.24497	-0.13623
0.18428	-0.070588	-0.10658	0.030164	0.043015	0.033063	0.14147
-0.12569	-0.64661	-0.29249	-0.36973	-0.52079	-0.52657	-0.058517
-0.20649	-0.17511	-0.2386	-0.20826	-0.32192	-0.13198	-0.23514
0.13739	0.12709	0.13449	0.29979	0.28625	-0.064319	0.13815
-0.23514	-0.33057	-0.15742	-0.29394	-0.43528	-0.50432	-0.72047
-0.65836	-1.151	-0.56614	-1.692	-1.0497	-1.359	-1.7743
-0.16439	-0.24992	-0.10143	-0.18435	-0.1843	-0.13312	0.30196
-0.21963	-0.17524	-0.17989	-0.25313	-0.43741	-0.66056	-0.28274

test_GSE439	test_GSE439	test_GSE439	test_GSE439	test_GSE439	test_GSE439	test_GSE439
ref_GSE4394	ref_GSE4394	ref_GSE4394	ref_GSE4394	ref_GSE4394	ref_GSE4394	ref_GSE4394
GSE4394	GSE4394	GSE4394	GSE4394	GSE4394	GSE4394	GSE4394
GEO	GEO	GEO	GEO	GEO	GEO	GEO
cDNA	cDNA	cDNA	cDNA	cDNA	cDNA	cDNA
RIFAMPICIN:	RIFAMPICIN:	RIFAMPICIN:	RIFAMPICIN:	RIFAMPICIN:	RIFAMPICIN:	RIFAMPICIN:
528	529	530	533	534	535	536
-0.22088	-0.031415	-0.32382	-0.61973	-0.36812	-0.7387	-0.78781
0.061978	0.093959	0.24719	0.30762	0.19214	0.37939	0.26808
0.076641	0.17443	-0.26616	-0.24709	-0.080312	-0.10187	0.15099
-0.15884	-0.043754	0.07899	0.016849	-0.16092	-0.31953	-0.20125
0.033743	0.032097	-0.033447	0.019107	0.031887	-0.21022	-0.13651
-0.39686	-0.18535	-0.44888	-0.23165	-0.31056	-0.66372	-0.56541
-1.05	-0.61686	-0.21478	-1.9189	-2.141	-1.515	-1.7158
0.22882	0.033999	0.09923	0.037797	-0.11623	-0.11765	0.020057
0.26662	0.2762	0.16932	-0.49915	-0.31878	-0.071498	0.13623
-0.10482	-0.28744	-0.24605	0.095468	0.12602	0.33968	0.19243
0.086533	-0.18447	-0.041682	-0.051778	-0.49667	-0.0014016	0.068144
-0.12534	-0.14535	0.28981	0.0067292	0.025659	0.11477	0.023433
0.073239	0.1427	0.29848	-0.32406	-0.079689	0.16695	0.020236
0.082	-0.094353	0.25809	-1.0665	-0.37042	-0.078192	0.027496
0.12944	0.25215	0.42631	0.057745	0.090047	0.10424	-0.012548
-0.071475	0.13114	0.019506	-0.14716	-0.045192	-0.17806	0.034082
-0.47101	-0.32201	-0.10729	-0.045856	0.084774	0.23596	0.1803
0.35213	0.30258	0.13417	0.012932	-0.12589	-0.16919	-0.052944
0.025282	-0.062672	0.097231	-0.67251	-0.28876	0.066546	0.2329
-0.020213	0.10552	0.22298	-0.20281	0.10962	0.43007	0.27495
0.21678	0.083634	0.57001	0.012282	0.13961	0.62927	0.54743
-0.17611	-0.13185	-0.13206	-0.73304	-0.45055	-0.63874	-0.81963
0.021228	0.037308	0.12291	0.12646	0.11548	-0.10285	-0.19039
0.015138	0.088691	-0.0081311	-0.54811	-0.12184	-0.043967	-0.14047
-0.0024997	-0.043198	0.12614	0.37201	0.39233	0.37621	0.47951
-0.21003	-0.18144	-0.35904	-0.54673	-0.36887	-0.85763	-0.80356
-0.025002	0.0041764	-0.14389	0.21703	0.018723	0.19665	0.35652
0.3263	0.38486	0.53162	0.12446	0.43418	1.0508	0.92172
-0.18097	-0.078961	-0.054922	0.089016	-0.38758	-0.5063	-0.48043
-0.50658	-0.15518	0.074452	-0.36942	-0.28682	-0.43655	-0.78034
-0.85016	-0.46444	-0.2417	-0.5216	-0.56209	-0.48516	-0.5889
-0.21643	-0.0035303	-0.44144	-1.4228	-0.84332	-1.3328	-1.5438

-0.070354	-0.044785	0.025603	-0.56458	-0.53047	-0.35135	-0.28853
0.28252	0.17197	0.53332	-0.083725	0.076666	0.030017	0.27241
0.11113	0.20299	-0.080657	-0.12058	-0.33659	-0.55684	-0.45415
0.19116	0.26077	0.1275	-0.09396	-0.069009	-0.17099	-0.048659
-0.080719	-0.19974	-0.01124	0.37287	0.45036	1.2907	1.274
-0.2266	-0.41378	0.069712	0.65096	0.74425	1.6703	1.7549
-0.14278	-0.026012	0.22383	-0.65528	-0.31335	-0.25344	-0.17964
0.27633	0.17249	0.25777	-0.13649	-0.0079458	0.23294	0.28672
-0.0020369	-0.14658	0.10199	-0.13882	-0.31816	0.33731	0.19596
-0.020583	-0.11425	0.032753	-0.25105	-0.20536	-0.39277	-0.33853
0.27081	0.032808	-0.068003	0.61785	0.22022	0.07057	0.025971
-0.028314	-0.15483	-0.093695	0.34853	0.22995	0.29071	0.3129
-0.057166	-0.10472	0.31759	-0.21058	-0.21461	-0.40209	-0.18626
-0.15262	0.080372	-0.80756	-1.2225	-0.7201	-1.2995	-1.3439
-0.0065954	-0.15669	0.18008	-0.34395	-0.39931	-1.0547	-0.93059
-0.16373	0.053098	-0.014118	-0.45073	-0.41392	-0.32543	-0.30974
-0.03619	-0.3729	0.0033367	-0.057989	-0.035449	-0.022998	0.26571
-0.13091	0.0032074	0.44952	-0.2782	-0.45841	-0.34383	-0.44297
-0.64065	-0.53317	0.025487	-1.0607	-0.41755	-0.43613	-0.46494
-0.17486	0.071999	0.20036	-0.036118	-0.15047	0.0030535	0.61529
0.10108	0.083197	-0.37638	-0.59018	-0.60756	-0.48478	-0.49167
0.038389	0.39792	-0.087514	1.0828	0.44815	-0.21323	-0.26149
-0.92424	-0.7177	-0.71503	-1.0761	-1.0126	-0.91504	-1.1147
0.11299	0.10425	-0.58309	-0.82291	-0.53349	-0.58942	-0.686
-0.21835	-0.079476	-0.20967	-1.2242	-0.27992	-0.3304	-0.5206
-0.20329	0.11277	0.25823	-0.10649	0.11691	0.55025	0.54717
0.11222	-0.29733	0.0278	0.3312	0.024808	0.87002	0.93438
-0.22304	-0.0315	0.22143	-0.85151	-0.3545	-0.27239	-0.22403
0.20191	0.15154	-0.013869	0.09134	0.13715	0.71639	0.85067
-0.086827	0.033652	-0.02177	0.27069	-0.35532	0.033736	1.1217
-0.20348	0.13681	0.18435	-0.14281	-0.16688	-0.1287	0.11699
0.17075	0.19245	0.039052	-0.49855	-0.64227	-0.35341	0.48741
0.159	0.071904	-0.096578	0.2486	0.060545	0.018431	0.022425
-0.41137	0.017089	-0.12094	1.4508	1.3128	1.2981	1.5994
-0.19611	-0.47796	-0.29792	-0.29645	-0.15665	-0.1632	0.01583
-0.10252	0.0043186	-0.0057083	-0.51844	-0.55118	-0.58214	-0.51914
-0.31748	-0.10701	0.083005	0.033509	-0.31215	-0.0038783	0.11222
0.23199	0.21971	0.22385	0.1868	0.13347	-0.096998	0.1237
-1.3829	-1.0427	-0.84094	-1.7773	-1.439	-1.8802	-1.8193
-0.38918	-0.50754	-0.69825	-1.3277	-0.82244	-1.1164	-1.2486
-0.45202	-0.51867	-0.035046	-0.047705	-0.33631	-0.28718	-0.12525
0.31677	0.17774	0.74794	0.078985	0.16671	0.63243	0.46935

-0.22918	-0.43111	0.098842	0.38919	0.061883	0.3661	0.45991
-2.0477	-1.9703	-0.72229	-0.93892	-1.0872	-1.9082	-1.8275
-1.3961	-1.3173	-0.85215	-1.4306	-1.5396	-2.1456	-2.1628
0.34926	0.21458	-0.059968	0.0069656	0.0071408	-0.16657	-0.13706
-0.066323	0.15902	0.1321	-0.45977	-0.19839	-0.029445	-0.1835
-0.025165	-0.22143	0.11518	0.10062	0.19334	0.62797	0.11696
0.20334	0.44333	0.6234	-0.65513	-0.43192	0.051088	-0.027775
0.15581	0.19787	-0.2309	-0.67784	-0.3652	-0.32885	-0.53864
-0.20018	-0.31044	-0.11787	-0.58724	-0.44941	-0.38314	-0.22649
-1.3801	-1.1607	-0.45333	-0.97449	-1.2108	-1.9109	-0.84319
-0.29378	-0.14308	-0.060997	-0.38887	-0.53615	-0.58064	-0.5654
-0.005157	0.10824	-0.079791	0.19169	0.18592	-0.016915	0.096378
-0.77866	-0.71015	-0.28493	-0.85706	-0.45547	-0.55648	-0.67309
0.22335	-0.098614	-0.13095	-0.06183	-0.11433	-0.11201	-0.36919
-0.96735	-1.1622	-0.91234	0.06381	-0.12581	-0.76652	-0.69162
0.05116	0.11102	0.25267	-0.18797	-0.032186	-0.16773	-0.094312
0.085247	-0.076174	-0.2725	-0.49622	-0.17586	-0.53564	-0.55927
-0.46637	-0.23847	0.23332	-0.70108	-0.38587	-0.17272	-0.28687
-0.1349	-0.1143	-0.34457	0.01528	-0.077987	-0.16605	0.042272
-0.075624	0.14459	0.038883	-0.31068	-0.30091	-0.10629	-0.26472
0.036957	0.23191	-0.026923	0.1813	0.1847	0.010491	0.018017
0.074744	-0.11767	0.2436	-0.48535	-0.4	-0.45345	-0.62242
0.0097584	-0.43109	-0.39959	-0.075007	0.14171	-0.098136	0.11929
-0.19499	0.033796	0.14155	-0.43297	-0.14278	-0.19977	-0.27147
0.22858	0.15889	0.11574	-0.31991	-0.30016	-0.29251	-0.14348
-0.23889	-0.046332	-0.030769	-0.39055	-0.26511	-0.036441	0.58907
-0.095172	0.0092133	-0.28473	-0.092726	-0.26622	-0.40619	-0.033806
-0.27167	-0.052626	-0.44117	-0.70208	-0.92439	-0.92307	-1.2013
-0.058666	-0.28274	-0.12651	-0.24361	0.17088	-0.032715	-0.27445
-0.11653	-0.27168	0.22931	0.070921	0.27376	-0.1998	0.093649
0.1427	0.067984	-0.33055	-0.44054	0.011494	-0.1535	0.1534
0.071072	0.10775	0.41923	-0.025357	0.0096534	0.2805	0.21306
0.01349	0.10131	0.47139	0.10033	0.4216	0.22998	0.41939
0.089424	0.056801	0.47776	0.0062732	0.45108	0.25677	0.42801
-1.2753	-1.2365	-0.5606	-1.4176	-1.2847	-1.4616	-1.6453
-0.35244	-0.33119	-0.35912	-1.5393	-0.91513	-1.1379	-1.0265
0.019235	0.1291	0.31401	0.1035	0.11792	0.03204	0.36848
-0.61207	-0.61178	-1.019	-1.6662	-1.2953	-1.6651	-1.4753
0.04658	0.18721	-0.080175	-0.71484	-0.40063	-0.28553	-0.11487
0.1344	-0.53836	-0.011831	-0.066242	0.098031	0.65431	0.2863
-0.39024	-0.22631	-0.0037391	-0.062584	-0.053991	-0.49578	-0.32878
0.2587	-0.044655	-0.16616	0.27863	0.0059182	-0.27716	-0.077181

0.13891	0.1608	0.062715	-0.059828	-0.21197	0.089944	-0.28352
0.0030458	-0.39324	-0.67101	-0.10878	-0.24625	-1.1727	-1.1242
0.14775	0.11203	-0.11261	-0.21434	-0.27963	-0.22243	-0.089721
0.013833	-0.094832	0.1682	-0.058689	-0.036756	-0.054003	-0.15942
-0.55111	-0.26314	-0.69506	-0.45384	-0.24174	-0.13075	-0.056622
0.69182	0.24627	0.49461	0.48144	0.62912	0.96382	1.0188
0.046922	-0.12007	0.11161	0.11789	-0.12069	0.11519	0.15784
-0.19822	-0.052487	-0.18873	-0.61428	-0.27735	-0.30685	-0.21593
-0.30583	-0.21379	0.018598	-0.070768	-0.32907	-0.36061	-0.40589
-0.10091	-0.10701	-0.18366	-0.48486	-0.97741	-0.50608	-0.38461
-0.025736	0.062744	-0.27926	0.19041	0.15151	0.10316	0.18039
-0.94295	-0.75707	-0.30194	-0.73509	-0.88881	-1.1775	-0.91913
-0.28632	-0.076472	-0.75874	-0.84371	-0.79159	-1.433	-1.2718
-0.34508	-0.2496	-0.80404	-0.49544	-0.46333	-0.76908	-0.68846
-0.59245	-0.90072	-1.2421	-0.63196	-0.45172	-0.33736	-0.25415
-1.3851	-1.5561	-1.0898	-0.71862	-0.96299	-1.8786	-1.7761
0.03111	-0.13323	-0.33445	-0.39833	-0.40544	-0.31121	-0.42831
0.14338	-0.076816	0.16836	0.35107	0.22494	0.33647	0.20435
-0.92272	-0.53424	-0.46641	-1.5199	-0.88903	-0.78681	-0.67026
-2.5562	-2.3646	-0.51174	-0.23196	-0.34434	-0.062987	-0.092705
-0.23399	-0.22544	0.13373	0.14977	-0.0054095	0.38023	0.26108
0.24667	0.10128	0.067203	0.090507	-0.09803	0.10045	0.114
NaN	NaN	NaN	NaN	NaN	NaN	NaN
-0.10787	-0.38896	0.14868	-0.5003	-0.37757	-0.60775	-0.43923
-0.51103	-0.44236	0.035864	-0.47686	-0.10147	0.0137	-0.061043
-0.19257	-0.04953	0.026357	0.04883	-0.059828	0.37574	0.4279
-0.55973	-0.40087	0.28844	-0.94748	-0.47252	-0.57544	-0.59585
-0.058906	-0.12848	0.18673	-0.10637	-0.033387	-0.050644	-0.17767
-0.11506	-0.12983	-0.43413	0.058396	0.099427	-1.1087	-0.92429
-0.34283	-0.35761	-1.0419	-0.22362	-0.080113	-0.15938	-0.029012
0.41151	0.22952	0.18423	-0.71902	-0.47803	-0.47675	-0.51477
0.24816	0.18896	0.127	-0.30096	-0.32767	-0.18982	-0.20861
0.20333	0.21203	-0.24876	0.19863	0.20721	-0.15884	-0.17196
0.30232	0.25499	0.021914	-0.4312	-0.027372	-0.15722	-0.28632
0.39139	0.22077	-0.2911	0.062929	-0.019352	-0.022725	-0.10163
-0.10127	-0.28271	0.21019	0.19053	-0.051131	0.189	-0.13249
0.19077	0.18754	-0.50874	0.14735	0.059942	-0.10839	-0.20586
0.16592	0.23162	-0.017669	-0.19676	-0.19529	-0.13078	-0.071187
0.27339	0.16372	0.23494	-0.14659	-0.076128	0.044506	-0.13842
0.1044	0.14843	-0.06296	-0.044817	-0.12061	-0.016707	-0.011652
-0.24531	0.00074025	0.20088	-0.75141	-0.19748	-0.13324	-0.43573
-0.74643	-0.61793	-0.20003	-1.6542	-1.0481	-1.3537	-1.5402

0.13847	-0.0029014	0.30885	0.18045	0.059182	0.54723	0.26211
0.17343	-0.041171	-0.11188	0.13612	-0.086368	0.25016	-0.018332
-0.38903	-0.30347	-0.24224	-0.070861	-0.19285	-0.014131	-0.084582
-0.091912	-0.26913	-0.090133	-0.063442	-0.064791	-0.30953	0.15754
0.11725	0.27948	0.11448	0.15434	0.18347	0.37624	0.28037
0.39561	0.17509	0.30138	0.0097735	-0.0041629	0.21079	-0.070584
-0.84897	-0.64374	-0.52694	-0.66883	-0.90931	-1.2051	-1.0521
-1.3614	-0.95593	-0.28159	0.14251	0.16582	-0.27837	-0.40816
0.13017	-0.1335	-0.81713	-0.57142	-0.81862	-0.91378	-0.82888
0.094577	-0.10326	0.56069	-0.13282	0.10596	0.18154	0.18097
0.074514	-0.25527	0.018345	0.42755	0.23984	-0.11694	-0.16104
-0.42382	-0.34912	-0.13623	0.014989	-0.037189	-0.10492	-0.075443
0.25364	0.15655	-0.23562	-0.30993	-0.15274	-0.3582	-0.28099
0.032703	0.047337	-0.085004	0.22421	0.094301	0.28461	0.097369
-0.11156	0.011624	0.58524	-0.44331	-0.1727	-0.12681	-0.1306
-0.43355	-0.30332	-0.18619	-0.15567	-0.0011358	-0.19077	-0.15796
0.034852	0.1583	-0.27761	0.18649	0.18813	0.70351	0.88241
-0.07196	0.024378	0.057633	0.49973	0.23233	0.39058	0.5209
-0.84595	-0.50454	-0.65021	-0.28446	-0.3533	-1.0211	-0.75258
0.029625	0.051863	0.089419	-0.045922	0.047276	0.35605	0.32135
0.23692	0.11514	0.20257	-0.32961	-0.23294	-0.2567	-0.26274
0.034182	0.088559	0.23269	-0.30471	-0.063996	-0.087954	0.1966
-0.41955	-0.17423	-0.1214	-0.99415	-0.66801	-0.78881	-0.8607
0.1067	0.16413	-0.30985	0.1013	0.0024455	-0.01068	0.41201
0.22259	-0.10553	0.19698	-0.16653	0.088275	-0.089159	0.15428
0.27058	0.08569	0.24895	-0.56443	-0.28949	-0.28148	-0.15348
0.099354	-0.0012865	-0.20938	0.15566	0.31023	0.36493	-0.025833
-0.20227	-0.26085	-0.20038	-1.0169	-0.7446	-0.95785	-1.0832
-0.021363	0.23326	-0.21372	-1.7946	-0.63554	-0.71382	-0.77421
-0.2781	-0.38195	-0.37462	-0.7261	-0.24775	-0.30489	-0.2343
0.00080778	0.068686	0.010656	0.080498	0.20148	0.37178	0.38688
-0.020094	-0.12784	-0.45781	0.066578	0.00055071	0.071894	-0.045522
0.014648	-0.1389	0.12216	-0.13332	-0.30505	-0.024374	-0.33681
0.10661	-0.045437	0.047469	0.02501	-0.1775	-0.26319	-0.029028
0.39701	0.21823	-0.10123	-0.48558	-0.42215	-0.61004	-0.61209
-1.1338	-0.98823	-0.0783	-0.82713	-0.72145	-1.0969	-0.96611
0.10073	-0.021626	-0.036392	0.16204	-0.034754	0.37012	0.38775
-0.43181	-0.29853	-0.39815	-0.18535	-0.31014	-0.61615	-0.85525
-0.36492	-0.60341	-0.93124	-0.40222	-0.3562	-0.2402	-0.096906
-0.076733	0.10237	0.1101	-0.51313	-0.0091165	-0.17875	0.11521
-0.5832	-0.42947	0.24712	-0.66919	-0.38898	-0.55212	-0.58945
-0.29337	-0.20492	0.55689	-0.26634	-0.38388	-0.2365	-0.33581

-0.92145	-0.83175	-0.17429	-0.57084	-0.33057	-0.70389	-0.7632
0.065566	0.038854	-0.21849	-0.22317	-0.12376	0.082558	-0.083723
-0.26206	-0.33668	-0.24872	-0.085476	0.075379	0.36294	0.10036
-0.17296	-0.40893	-0.12272	-0.38241	-0.20573	-0.16767	-0.23978
0.022495	0.31479	0.20284	-0.019193	0.049519	-0.10583	-0.16693
-1.1401	-1.0584	-0.38358	-1.1198	-1.2743	-1.6296	-1.3575
-0.098774	-0.13027	-0.30068	-0.29966	-0.41299	-0.30874	-0.28915
-0.054363	-0.14887	-0.13311	0.13123	0.08028	0.28053	0.23113
0.076149	0.13777	0.32155	-0.037274	-0.0019339	0.25799	-0.14649
-1.0466	-0.79873	-0.61257	-1.4689	-0.80441	-0.90676	0.23349
-0.19485	-0.16473	-0.23797	-0.11877	0.085493	0.096432	0.049248
-0.79717	-0.50659	-0.15473	-2.0417	-0.96498	-1.0317	-0.95793
0.32692	0.29847	0.17009	-0.47966	-0.26835	-0.23822	-0.19197
-0.065371	0.00015351	0.13377	0.015399	-0.41322	0.27959	-0.31317
0.34686	-0.044961	-1.0956	0.36929	0.38982	0.15787	0.059333
0.15679	0.082987	0.12234	-0.24464	-0.034321	-0.10838	-0.075063
-0.28138	-0.051284	-0.03713	-1.0674	-0.56362	-0.072196	-0.19548
-0.63405	-0.39891	-0.31	-0.080605	-0.15979	-0.032775	0.065736
-0.042809	-0.081663	0.17676	-0.76414	-0.44556	-0.3947	-0.37289
-0.014326	-0.088172	0.13023	-0.37793	-0.25908	-0.096378	-0.25679
-0.84959	-1.1104	-0.87338	-1.4989	-1.234	-1.9449	-1.9138
-0.052204	0.10571	0.1533	0.0065417	-0.014597	-0.060886	0.0098053
-0.12958	-0.26431	-0.43853	0.39795	0.20337	-0.52878	-0.57154
-0.87182	-0.63568	-0.63705	-1.098	-0.83854	-1.1099	-0.98915
-0.41565	-0.31541	-0.36681	-0.46895	-0.38463	-0.60726	-0.54174
0.19794	-0.30414	0.011505	0.080344	0.20465	0.11556	0.59545
0.006188	0.0077205	0.063772	0.043586	-0.10005	-0.092936	0.010148
-0.26506	0.038736	-0.071427	-0.38693	0.044461	-0.17562	0.026542
-0.77842	-0.60016	-0.20242	-0.41925	-0.54891	-1.1923	-1.1405
-1.3557	-1.1642	-0.5952	-2.3513	-1.3396	-1.5818	-0.5134
0.21521	-0.21298	0.18689	0.31651	0.1904	0.8017	0.89833
-0.14733	0.22084	0.017938	-0.063027	-0.022473	0.13659	0.12006
0.13212	-0.014064	0.061911	-0.047049	0.1279	0.01832	-0.16407
0.10084	0.16129	-0.28878	-0.67314	-0.063086	-0.0846	-0.25645
-0.19843	-0.13523	0.057861	-0.099746	-0.12487	-0.12889	-0.34362
-0.14386	-0.23001	-0.19035	-0.1243	-0.21182	-0.14575	0.055379
-0.14326	-0.11579	0.15095	-0.70666	-0.4966	-0.20752	-0.30761
-0.032845	0.21888	-0.44184	-0.24982	-0.028821	-0.4597	-0.46099
0.13026	0.15307	0.089032	0.72773	0.60259	0.39322	0.3143
0.24024	0.12384	-0.16761	0.26104	0.25323	0.098027	0.010594
0.10731	0.15869	-0.11403	-0.64267	-0.27926	-0.42588	-0.33979
0.31533	0.22476	0.2682	-0.25434	-0.31107	0.16051	-0.083721

-0.25801	0.0108	0.17961	-0.101	0.048062	0.10277	0.082176
0.15438	0.12298	0.18861	0.15086	0.10215	0.044317	0.086203
-0.79065	-0.72175	-0.85293	-0.36063	-0.59728	-2.0758	-2.0699
-0.50845	-0.28367	-0.14399	-0.42599	-0.2441	-0.11522	-0.29461
0.075151	0.0081376	-0.0067463	-0.2149	0.014349	0.058379	0.044941
-0.066794	-0.050974	0.11458	0.10414	0.12619	0.10952	0.091904
-0.31283	-0.31733	-0.40124	-0.18175	-0.12621	-0.298	-0.38237
-0.016666	0.098565	0.097304	-0.042209	-0.064715	0.033723	-0.0013845
0.08946	-0.21379	-0.083238	-0.46869	-0.44515	-0.39152	-0.4735
-0.081626	-0.24329	0.03786	-0.053347	-0.10328	0.15606	0.12555
0.27604	0.24309	0.2099	0.71865	0.53307	1.1419	1.2336
-0.070105	-0.03813	-0.29371	-0.94959	-0.30232	-0.50275	-0.47397
0.0088324	0.29682	-0.45375	-0.60515	0.53158	0.14992	-0.40288
0.052963	0.1188	0.24256	-0.0035418	-0.16489	-0.020459	-0.13488
0.21007	0.16237	0.60936	-0.16299	0.089388	0.48883	0.67633
-0.0067889	0.026917	0.15675	-0.17227	-0.07767	-0.18359	-0.11006
-0.48799	-0.39731	-0.19012	-0.23064	-0.27797	-0.5733	-0.45612
-0.0033222	-0.13714	-0.39785	-1.1076	-0.52554	-0.84259	-1.049
-0.11969	-0.012147	-0.32045	-0.67595	-0.57732	-0.7595	-1.0255
-0.054517	-0.055614	-0.0045354	-0.61157	-0.3995	-0.1234	-0.1934
-0.11627	0.1246	-0.11647	-0.6104	-0.54807	-0.72063	-0.75806
-0.35759	-0.35933	-0.27602	-0.7988	-0.53794	-0.81792	-0.86392
-0.13296	0.0070003	0.16844	-0.45328	-0.03938	-0.43894	-0.63324
0.67813	0.7028	0.58729	0.20749	0.23754	0.75639	0.75431
0.11598	0.094739	-0.16299	-0.13733	-0.19727	-0.22275	-0.27276
-0.58086	-0.66351	-0.2002	-1.0015	-0.42841	-0.2675	-0.26746
-0.45443	-0.23514	-0.78784	-0.69523	-0.61599	-0.55838	-0.35011
-0.21551	-0.34542	0.030908	-0.5182	-0.55956	-0.3762	-0.60292
-0.10552	0.025542	0.013414	0.053766	-0.012676	0.009949	0.13874
0.0038405	0.02413	0.10646	-0.136	0.094147	-0.1525	0.28601
-0.15354	0.12238	-0.35007	-0.12437	-0.097595	-0.02239	0.18293
-0.07248	0.014559	0.14128	0.13632	0.035438	0.095332	-0.049342
-0.31078	-0.18421	-0.11294	-0.17924	-0.37329	-0.3618	0.17149
0.21344	0.33923	0.174	3.6459	-0.25055	-0.050796	0.021097
0.011732	0.036528	0.20013	-0.85843	-0.43809	-0.38553	-0.15745
-0.14051	-0.17001	-0.67578	-0.25316	-0.16477	-0.47495	-0.71942
0.050774	0.26124	0.46739	-0.34507	-0.12595	-0.018742	-0.062614
-0.1918	-0.1024	-0.042576	0.68544	0.52	0.68223	0.68247
-0.33172	-0.23893	-0.31295	-0.69433	-0.73759	-0.77613	-0.73191
0.18655	-0.010783	0.20978	0.41481	0.12754	0.83115	0.62861
-0.55324	-0.2915	0.23469	-0.98012	-0.61624	-0.57878	-0.39138
0.18087	0.19308	0.30571	-0.010574	0.049611	0.33106	0.31515

-0.24471	-0.18537	0.10408	-0.38393	0.38419	-0.38928	-0.32749
0.35996	0.2498	0.28671	-0.56361	-0.46575	-0.44489	-0.34512
0.32303	-0.15786	0.34478	0.22366	0.14873	0.25339	0.23925
-0.26808	-0.15834	-0.24447	-0.07953	-0.37956	-0.82171	-0.65314
-0.56611	-0.33685	-0.054317	-0.054205	-0.63561	-0.75385	-0.75474
0.086996	-0.32287	-0.3442	-0.4802	-0.47441	-0.76949	-0.63879
-0.088691	0.01624	0.21046	-0.061629	-0.16165	-0.15861	-0.0071291
-0.14575	-0.15949	-0.18769	-0.022864	-0.24445	0.040539	0.088121
-0.29147	-0.27472	-0.54661	-1.2598	-0.52332	-0.64607	-0.67466
-0.71118	-0.52661	-0.1653	-0.42318	-0.37897	-0.59983	-0.60034
-0.13214	-0.1797	0.23875	-0.092878	-0.048078	0.0058869	-0.016721
0.35251	-0.19895	-0.27814	-0.25137	-0.40191	-0.29519	-0.22428
-0.075917	0.14895	0.056332	-0.14542	0.016509	-0.008114	-0.15083
-0.31916	-0.25452	0.026731	-1.106	-0.96116	-1.4299	-1.2351
-0.73184	-0.67221	-0.19552	-0.39715	-0.2892	-0.26162	-0.067603
0.4666	0.32722	0.29659	0.63208	0.55809	0.50866	0.43159
-0.041358	-0.16478	-0.70287	-0.3767	-0.17775	-0.21632	-0.3481
-0.35877	-0.32041	-1.2106	-2.2723	-1.9414	-1.9747	-2.0759
0.02531	-0.027093	0.11773	-0.15909	-0.1673	-0.06942	0.18685
-0.2226	-0.12612	-0.38159	0.01498	-0.30073	-0.39861	-0.40284

test_GSE439	test_GSE439	test_GSE439	test_GSE439	test_GSE439	test_GSE439	test_GSE439
ref_GSE4394	ref_GSE4394	ref_GSE4394	ref_GSE4394	ref_GSE4394	ref_GSE4394	ref_GSE4394
GSE4394	GSE4394	GSE4394	GSE4394	GSE4394	GSE4394	GSE4394
GEO	GEO	GEO	GEO	GEO	GEO	GEO
cDNA	cDNA	cDNA	cDNA	cDNA	cDNA	cDNA
RIFAMPICIN:	RIFAMPICIN:	RIFAMPICIN:	RIFAMPICIN:	RIFAMPICIN:	RIFAMPICIN:	RIFAMPICIN:
537	538	539	541	543	545	547
-0.14539	-0.70521	-0.1142	-0.18193	-0.38317	-0.32945	-0.32455
-0.033676	0.20127	0.0072767	0.05697	0.078136	0.076517	0.095517
-0.030127	-0.047548	-0.0014224	-0.16026	0.054425	-0.066444	0.30038
0.0024922	-0.018055	-0.0015201	-0.12577	-0.043594	-0.29365	-0.15502
-0.074815	-0.16719	0.076271	0.08455	-0.046205	0.082643	0.040241
0.011769	-0.69222	0.20197	0.014601	0.054528	0.096082	-0.24262
-0.8048	-1.0943	-0.77934	-1.2032	-1.4709	-1.0995	-1.0395
0.019552	0.26775	0.093958	0.15155	0.13019	0.19206	0.28435
0.060293	0.25637	0.025905	0.057702	0.19411	0.21725	0.17006
0.21485	-0.030355	0.048427	-0.047145	-0.16869	-0.08305	-0.10416
0.18636	0.22854	0.33661	-0.25242	-0.075455	-0.12004	-0.079788
0.099038	0.43449	0.012222	-0.068582	-0.12126	-0.055128	-0.17681
-0.073375	0.29838	-0.021426	-0.095454	0.14292	0.16282	0.083357
-0.067639	-0.0071637	-0.02312	-0.038371	-0.10146	-0.10865	-0.27731
-0.17849	0.14814	-0.054538	0.044075	0.0054971	0.35808	0.1799
-0.0089467	-0.1654	0.028525	-0.016272	0.19473	0.12063	0.18983
0.20762	0.23287	0.15034	-0.083844	-0.13807	-0.23079	-0.44938
-0.06816	0.013877	-0.011664	-0.027426	0.016009	0.2319	0.035174
-0.05037	-0.25185	-0.026791	-0.10451	-0.13773	-0.25735	-0.34577
-0.02655	0.045484	-0.033192	-0.075205	0.079816	0.10171	-0.15021
0.058254	0.34595	-0.16434	0.096331	0.12121	0.068019	-0.20299
0.060792	-0.21732	0.0062765	-0.19893	-0.23702	-0.01285	-0.24125
-0.01724	0.072573	-0.0127	-0.07206	0.0037888	-0.012175	0.033783
0.12639	-0.23919	0.12583	0.011592	0.058062	0.032587	0.09669
0.12058	0.3262	0.030019	-0.012796	0.024603	-0.043413	-0.0091397
0.14267	-0.70802	0.012647	0.18433	-0.33436	-0.076565	-0.1805
0.048847	0.55195	0.088255	0.15812	-0.0053011	0.35693	0.14764
0.17867	1.1279	0.065487	0.12991	0.13218	0.22617	0.16492
-0.12548	-0.35826	-0.16949	-0.11496	-0.09205	0.038272	0.17206
-0.14768	-0.41911	-0.13269	-0.045988	0.073976	-0.049693	0.1375
-0.19225	-0.54747	-0.22049	-0.48885	-0.52164	-0.74847	-0.53326
-0.11355	-0.87871	-0.14995	-0.10007	-0.13074	-0.1477	0.037493

-0.13443	0.063297	-0.10814	-0.20301	-0.25574	-0.31037	-0.091563
-0.054655	0.13217	-0.042312	-0.016294	0.10565	-0.067628	-0.076305
0.12232	-0.50069	0.1594	0.11605	0.11625	0.013994	0.12022
-0.20533	0.56786	0.28442	0.0062932	0.25183	0.23643	0.11387
-0.18664	1.1952	-0.21073	-0.094695	-0.075791	-0.12809	0.44164
-0.042771	1.9449	-0.14188	-0.30745	-0.44583	-0.15474	-0.34188
-0.28628	-0.079713	0.02159	-0.0029819	0.043753	-0.13981	-0.050166
-0.0068639	0.10055	-0.073129	-0.052763	-0.016487	-0.005812	-0.30285
0.050755	0.25755	-0.071204	-0.041398	0.00068698	-0.079548	0.20966
-0.086084	0.15207	-0.18222	-0.23851	0.036273	-0.0035587	-0.02214
0.19501	0.29736	0.15798	0.3714	0.2559	0.20685	0.29349
-0.066052	0.30766	0.062658	-0.14299	0.35486	-0.20671	0.33081
-0.12245	-0.030884	-0.27765	-0.12174	-0.2475	-0.27048	0.052801
-0.0012578	-1.1429	-0.087375	-0.10906	-0.15577	0.0063954	0.0071468
0.12638	-0.9679	0.066511	0.0062702	-0.087957	0.13797	0.15176
-0.11897	-0.54804	-0.18693	-0.12621	0.18042	-0.16761	0.11705
0.14189	0.006729	0.13345	0.038437	0.19365	0.16208	0.13803
-0.1565	0.042337	-0.09408	-0.18121	-0.35	-0.11293	-0.085989
-0.15083	-0.40279	-0.15107	-0.22831	-0.42887	-0.57846	-0.43319
-0.13963	0.045484	-0.059204	-0.017019	-0.017653	-0.018634	0.17014
-0.12886	0.00089485	0.01353	-0.20872	-0.1282	-0.191	-0.031484
-0.063311	-0.36851	-0.11816	-0.030535	-0.21341	-0.020155	-0.18714
-0.1554	-1.0341	-0.20571	-0.5188	-0.50889	-0.91949	-0.982
0.050761	-0.44645	-0.12019	0.021174	-0.043111	-0.1031	-0.0016206
-0.17881	-0.37459	-0.015542	-0.12596	-0.033909	-0.36164	-0.22705
0.050741	1.0425	-0.027688	0.058876	-0.048967	-0.1417	0.21641
0.14058	0.44375	0.22329	0.23886	0.088712	0.07508	0.31259
-0.070427	-0.26408	-0.07166	-0.10904	-0.33586	-0.28492	-0.35019
0.043193	0.36463	0.0831	-0.0043648	0.11027	0.20509	0.15605
-0.067334	0.065811	0.1506	0.079011	0.063565	-0.14185	0.29632
-0.05767	-0.16501	-0.039916	0.18103	0.26248	-0.13295	0.32855
-0.048889	-0.1565	0.014378	-0.048605	0.12574	0.08793	0.067724
-0.11638	-0.014449	-0.16881	0.1223	0.054089	-0.037806	0.11688
-0.16295	1.2808	-0.032646	-0.044579	-0.18635	-0.18681	-0.15511
-0.084364	-0.20568	-0.12355	-0.35679	-0.14313	0.081556	-0.06485
-0.095633	-0.2718	-0.19069	0.025642	-0.1299	-0.11231	0.021747
-0.095374	0.17616	-0.072132	-0.030738	-0.30364	-0.28614	-0.30439
0.0034705	0.077923	-0.040162	-0.051356	0.21271	0.37837	0.063462
-0.42755	-0.97591	-0.43651	-0.9116	-1.628	-1.8237	-1.4579
-0.13249	-0.75827	-0.0198	-0.16988	-0.33208	-0.30745	-0.3272
0.11945	-0.058488	-0.0046769	0.040092	-0.24454	-0.36183	-0.18008
0.055196	0.62255	-0.0050385	0.1545	0.20392	0.19365	0.13588

-0.15121	0.61831	-0.026538	-0.23394	-0.33365	-0.26465	-0.020697
-0.77977	-1.4589	-0.65277	-1.1258	-1.3115	-1.5297	-1.733
-0.28446	-1.5853	-0.090594	-0.80549	-0.84711	-1.2892	-1.3299
-0.03452	-0.12322	-1.41E-05	-0.14708	0.20171	0.19324	0.15472
-0.11255	0.068803	-0.09286	0.042861	0.13944	0.049021	0.029862
-0.11989	0.099054	-0.10655	0.0085499	0.16901	0.08268	0.13189
0.07787	-0.1046	0.069845	0.075617	0.30051	0.19873	0.31305
-0.084845	-0.55399	-0.069017	-0.16358	-0.0071457	0.12672	0.036699
-0.34573	0.1742	-0.32589	-0.44712	-0.28418	-0.068898	-0.093288
-0.36771	-1.57	-0.33076	-0.855	-1.1657	-1.2468	-0.94642
-0.052674	0.045065	-0.0093021	-0.10131	-0.08092	-0.17432	-0.0044532
0.028973	-0.10984	0.04956	-0.091043	-0.072975	-0.14418	-0.21294
-0.14087	0.17808	-0.24353	-0.48241	-0.33818	-0.76216	-0.49047
0.039853	-0.18623	-0.070351	0.079958	0.04315	-0.11769	0.025297
0.073926	-0.44964	-0.01494	-0.44653	-0.46034	-0.5724	-0.50784
0.038574	-0.075742	-0.029268	-0.01931	-0.25535	-0.24949	0.095214
-0.12783	-0.30284	0.029152	-0.041827	-0.1378	-0.16904	-0.2378
0.17951	-0.16161	-0.051527	-0.18541	-0.18169	-0.20778	-0.10226
0.089436	-0.25471	-0.049966	0.15986	-0.20011	-0.13063	-0.072319
-0.0082897	-0.099675	-0.0748	0.043891	0.12371	-0.026517	0.11008
-0.015846	0.68796	-0.074872	0.081954	-0.054279	0.25674	0.013906
-0.0043036	-0.17589	-0.08125	-0.17486	-0.26159	0.0053071	-0.20904
0.052135	-0.13606	-0.048453	0.12436	0.054229	-0.017127	-0.20744
0.021108	-0.26247	-0.043129	0.081737	-0.095946	-0.16645	-0.083709
-0.080846	0.14884	-0.054691	-0.093439	0.019222	0.094084	0.025216
-0.094265	-0.10338	-0.049141	0.075794	0.12185	-0.014169	0.0031142
-0.16492	-0.31645	-0.22941	0.038534	0.030024	0.025257	-0.16281
-0.040697	-0.91095	0.071316	-0.12394	-0.12717	-0.18743	-0.14041
-0.014814	0.12419	-0.044149	-0.015591	-0.072573	-0.21585	-0.30277
-0.11335	-0.081129	-0.011289	-0.0021501	0.028019	-0.10797	0.11148
-0.068478	-0.026974	-0.072363	-0.037828	-0.022893	-0.0008469	0.048543
0.017197	0.29958	-0.05833	0.034474	0.21265	0.015942	-0.14368
-0.10014	0.24666	0.16049	0.19696	0.28201	0.059037	-0.014583
0.26776	-0.064391	-0.13207	-0.12518	0.15092	0.10621	-0.30783
-0.41436	-0.35391	-0.35904	-0.43086	-0.87352	-0.47527	-0.61879
-0.085993	-0.45732	-0.11838	-0.41165	-0.49156	-0.29575	-0.60547
0.047741	0.027429	-0.081094	-0.037208	0.093128	0.031883	0.20653
-0.45117	-0.78705	-0.32735	-0.62858	-0.96055	-0.86957	-0.8785
0.0039771	-0.058457	-0.15479	-0.05402	-0.068472	-0.017	-0.075113
0.0045674	0.36831	-0.085235	-0.10145	-0.010925	0.16162	0.065449
0.0028304	-0.19947	-0.021778	0.045134	-0.16562	0.053158	-0.25
0.33006	-0.2494	0.34633	0.35578	0.2092	0.31217	0.29507

-0.079945	0.035746	-0.0077244	0.045915	0.084052	-0.025054	-0.015206
0.030605	-1.4455	-0.033465	-0.23808	-0.26389	-0.22657	-0.25523
-0.072301	0.16789	0.067829	0.0051458	0.14862	0.069744	-0.050867
-0.066906	-0.037989	0.16701	0.0096252	0.050201	0.0084975	0.24039
-0.086659	-0.31549	-0.080058	-0.18071	-0.3829	-0.34565	-0.4423
0.11519	0.67894	0.063355	0.13925	0.013531	0.19732	0.1693
-0.061083	-0.097288	0.0032162	0.21804	0.21851	0.062396	0.30732
-0.063539	-0.18258	-0.061762	0.027093	0.013616	-0.15669	-0.22894
-0.035578	-0.15146	-0.059233	-0.037684	-0.20372	-0.048915	0.041596
-0.042432	-0.18572	-0.063249	-0.25963	-0.024373	-0.14547	-0.069551
-0.038229	0.23279	-0.13842	-0.32186	0.12565	0.062757	0.081771
-0.15541	-1.206	-0.12616	-0.47913	-0.57696	-0.81102	-0.8215
0.046266	-1.1758	0.079433	-0.15237	0.029126	-0.24531	-0.035437
0.14502	-0.13593	0.16809	0.16607	0.089905	-0.0036165	-0.10944
-0.01573	-0.32565	0.0075465	-0.049955	-0.26504	-0.098612	-0.14842
-0.088002	-1.2165	-0.16193	-0.6494	-0.62467	-0.92011	-0.66889
-0.024992	-0.24702	0.042857	-0.26287	-0.17962	-0.32927	-0.25
0.040526	0.23071	-0.0025708	0.20712	0.25248	0.33843	0.30714
-0.44267	-0.63049	-0.5438	-1.0144	-1.1342	-1.1303	-0.87956
-0.65019	0.49374	-0.51015	-1.4637	-1.5127	-2.0507	-2.1211
-0.21088	0.16689	-0.18876	-0.17135	0.11594	-0.050067	0.091351
-0.015446	0.33767	-0.0095866	0.17757	0.10611	0.12351	0.29691
NaN	NaN	NaN	NaN	NaN	NaN	NaN
0.10622	-0.53133	0.02538	-0.11368	0.033431	-0.039574	-0.030432
-0.12002	0.026511	-0.1554	-0.10525	-0.48127	-0.36674	-0.40955
-0.099302	0.063947	-0.022584	0.023755	-0.020579	-0.15744	0.052558
-0.028955	-0.029222	-0.17101	-0.26999	-0.11626	-0.42362	-0.27521
0.13712	-0.063462	-0.029116	-0.053649	-0.052608	-0.098776	-0.17967
-0.062924	-0.81183	0.011835	0.051018	-0.10759	0.023085	-0.047597
-0.016333	-0.025062	0.012447	-0.16521	-0.19235	-0.23314	-0.096181
0.080682	-0.15157	0.074989	0.016144	0.36444	0.35396	0.10661
0.03046	-0.14214	0.012108	-0.045922	0.11996	0.12464	0.052676
0.15344	-0.18969	0.1181	0.14337	0.13118	0.12322	0.0064862
-0.12551	-0.071236	-0.091896	-0.074711	-0.031568	-0.12573	0.0056967
0.24181	0.0010003	0.18723	0.40483	0.34684	0.069019	0.39191
0.11122	0.1482	-0.031427	0.077759	0.13899	0.19039	0.24052
0.027453	-0.5458	0.11242	0.15511	0.032756	0.14913	0.23044
-0.053251	-0.36661	-0.076035	0.022149	0.043344	-0.14322	-0.020506
-0.12008	0.078487	-0.023504	-0.010316	0.15407	0.093088	0.17395
-0.060249	-0.17007	0.032518	0.077232	-0.024381	-0.10072	-0.17203
-0.12359	-0.35732	-0.10736	-0.052275	-0.26143	-0.42442	-0.10154
-0.35574	-0.74686	-0.28545	-0.41511	-0.15462	-0.39491	-0.3679

0.095977	0.47014	-0.0027683	0.014503	0.1929	0.092756	0.22464
-0.080138	0.031933	-0.028381	0.043728	0.089143	-0.11538	0.041517
0.04716	0.2283	-0.092948	-0.15125	-0.17535	-0.021465	-0.18927
-0.080789	0.08076	-0.14169	-0.015144	-0.26299	0.068555	0.0030374
0.073343	0.33384	0.085043	0.13709	0.13764	0.0834	0.24374
0.035403	0.095995	-0.12727	0.053513	-0.018993	0.15004	0.25985
-0.3691	-0.80305	-0.18468	-0.57826	-0.76323	-0.68635	-0.7041
-0.38967	-0.49074	-0.37416	-0.80248	-0.91925	-1.0968	-0.65747
0.26427	-0.54626	0.29363	0.15044	0.15639	0.20751	0.2126
0.010813	0.19393	-0.032096	-0.020928	0.021495	-0.16311	-0.40415
0.011111	-0.17576	0.144	0.13241	0.28823	0.28788	-0.077138
-0.12591	-0.016391	0.06449	-0.20322	-0.22825	-0.065365	-0.027724
-0.1328	0.011775	-0.068804	-0.048137	0.13031	0.025648	0.087933
-0.12044	0.46967	0.031455	0.19877	0.33111	-0.076026	0.048827
0.029175	0.12446	-0.0030414	0.030446	0.0060197	0.20153	0.13622
-0.21545	-0.32626	-0.14521	-0.091704	-0.27296	-0.41521	-0.6003
-0.092937	0.68116	0.091453	-0.08139	-0.055708	0.15098	0.28969
-0.15156	0.69358	-0.20839	-0.051412	0.1017	-0.016	-0.014081
-0.077427	-1.3706	-0.082468	-0.21165	-0.53627	-0.76922	-0.60238
0.10937	0.32322	-0.043018	0.015468	0.010876	0.083545	-0.2288
-0.0068806	-0.37805	-0.041602	0.0098837	0.03489	0.098732	-0.079217
0.065164	0.07691	-0.10799	-0.072563	-0.18976	-0.06362	-0.035871
-0.074024	-0.3899	0.017827	-0.11504	-0.055354	0.097185	-0.37606
-0.088181	0.051591	0.050946	0.025177	-0.25808	-0.071311	-0.12339
0.1165	0.039774	0.12564	0.099092	0.27056	0.067281	-0.22218
-0.12402	0.10198	-0.13852	-0.029628	0.065402	0.3579	0.12124
0.10722	0.046817	0.069821	0.21901	-0.044293	0.064662	-0.017284
-0.039242	-0.51141	0.097377	-0.063758	-0.18903	-0.14521	-0.14141
-0.049096	-0.49047	-0.089506	0.078177	0.047483	-0.023021	0.12004
-0.12122	-0.0001197	-0.1409	-0.26945	-0.52938	-0.44783	-0.79946
0.021314	0.3555	0.079114	-0.072056	-0.049042	-0.10989	-0.067725
-0.028066	-0.046814	0.07865	0.012843	0.034457	0.16563	-0.13424
-0.20406	0.11353	-0.19643	-0.15191	-0.13632	-0.10689	0.24512
-0.12519	-0.0039105	-0.14987	-0.24583	0.034586	0.10423	0.077462
0.053232	-0.53752	0.2429	0.25528	0.2368	0.084251	0.18729
-0.048883	-0.25273	-0.12622	-0.51559	-0.60095	-0.6051	-0.69982
-0.080518	0.54663	-0.071055	-0.033184	-0.055597	0.11746	0.1395
0.13737	-0.0414	0.15748	0.14715	0.30607	-0.032693	-0.0212
0.16936	-0.10611	0.026683	-0.19454	-0.3692	-0.18619	-0.23704
-0.045721	0.11357	-0.1115	-0.072335	-0.062142	-0.1074	-0.20556
-0.17245	-0.14292	-0.14598	-0.13011	-0.44212	-0.53137	-0.63459
-0.22983	-0.059775	-0.027479	-0.081033	-0.23942	-0.097106	-0.11658

0.10441	-0.36314	-0.020052	-0.22469	-0.31808	-0.62858	-0.43563
-0.10619	0.34194	-0.15007	-0.19606	-0.11213	-0.081735	-0.0010546
0.21594	-0.32702	0.08518	0.03598	-0.0045825	-0.22239	0.12925
0.15654	-0.10062	0.065089	0.0092516	0.012851	-0.010544	-0.15787
0.049533	-0.14205	0.096368	-0.010198	0.1984	0.29839	0.3665
-0.073419	-0.72521	-0.20897	-0.74301	-0.67464	-0.84068	-0.664
-0.10248	-0.39769	0.020085	-0.017972	-0.18692	-0.058519	-0.085326
0.00070951	0.16705	0.077891	0.14806	-0.27125	-0.11198	-0.028917
-0.060633	-0.039243	-0.10561	0.12056	-0.078561	0.017381	0.19079
-0.15092	-0.85793	-0.1093	-0.58243	-0.89244	-1.0304	-0.92959
0.05459	-0.081384	0.083958	0.0016322	0.071228	-0.041831	-0.13142
-0.29058	-0.87714	-0.30745	-0.72767	-0.93231	-1.1663	-0.19221
-0.17086	-0.18066	-0.1507	-0.050103	-0.018377	0.27475	0.29112
-0.14252	-0.11211	-0.14405	-0.10268	-0.092435	-0.12292	0.070162
0.14679	-1.1025	-0.08088	0.080125	0.085618	0.15298	0.12651
-0.039022	-0.072424	-0.0027755	-0.016776	-0.1389	-0.022788	-0.0096067
-0.28123	0.3964	-0.32178	-0.50523	-0.61773	-0.41546	-0.33844
0.37807	0.0025871	0.049363	-0.18146	-0.37669	-0.55997	-0.62389
-0.19812	-0.17131	-0.089593	-0.15014	-0.23387	-0.19577	-0.22404
0.3575	-0.13554	0.28394	0.21855	0.28911	0.12888	0.015651
0.1884	-1.2731	0.17873	-0.094436	-0.83685	-0.90217	-1.1596
-0.012726	0.14076	-0.1253	0.0044434	-0.0057334	0.057174	-0.034769
0.034213	-0.32135	0.11054	-0.071785	0.069129	0.023041	-0.010129
-0.12946	-0.88401	-0.2042	-0.75549	-1.1064	-1.3217	-0.98599
-0.066178	-0.51837	0.0037264	-0.088987	-0.12012	-0.092882	-0.12711
0.027113	-0.0099352	0.095431	0.093253	-0.051775	0.15681	0.078383
-0.17051	-0.055027	-0.12936	0.010962	0.122	0.0049951	0.2009
-0.13841	0.25151	-0.094863	-0.056213	-0.070356	0.12478	-0.021117
-0.32507	-0.38375	-0.13042	-0.41081	-0.27437	-0.38177	-0.42032
-0.29886	-1.2278	-0.25609	-0.46287	-0.62508	-1.0146	-0.72149
-0.12301	0.59758	0.022832	-0.037157	0.010762	0.048386	0.30744
0.011188	0.56205	0.0072303	-0.20805	0.057213	-0.03074	0.059111
0.0075832	0.031241	-0.051952	0.085137	-0.06325	0.15558	-0.11715
-0.27248	-0.21186	-0.15648	0.12521	-0.098461	-0.066642	-0.44387
-0.0049219	0.036235	-0.052758	0.037652	-0.12036	-0.008558	0.076858
0.13995	0.21997	0.26267	0.11122	0.0022909	0.0039817	-0.1119
-0.20987	-0.15757	-0.22416	-0.23543	-0.64563	-0.48025	-0.34436
0.39174	-0.80161	0.11271	0.39418	0.33978	-0.12798	-0.26126
-0.025209	0.082672	0.071053	0.12844	0.42674	0.16896	0.14666
-0.0003056	0.30651	0.078812	0.22986	0.23071	0.061393	0.11886
-0.081537	-0.091309	-0.089881	0.014263	0.01989	0.10285	-0.16944
-0.22629	0.14378	0.0056533	-0.12294	0.14063	0.055375	0.096151

-0.040441	-0.010716	-0.15532	-0.091253	-0.048994	-0.18672	-0.43195
0.043703	-0.012296	-0.088864	0.039171	-0.0042585	0.078174	0.087758
0.22074	-1.5017	0.1398	-0.14359	-0.052737	-0.62677	-0.49353
-0.11254	-0.16168	-0.12442	-0.21559	-0.1934	-0.23475	-0.33571
-0.052226	0.22575	-0.066387	0.021436	-0.069698	-0.040793	-0.093419
-0.029193	-0.2665	-0.034015	-0.033983	-0.14967	-0.085673	-0.30333
-0.17352	-0.086584	-0.072504	-0.29308	-0.23784	-0.47508	-0.52349
-0.0028617	-0.14078	-0.034987	0.095391	-0.087003	-0.08108	0.11399
0.015391	-0.153	0.13092	0.0014736	0.029923	-0.055405	0.026022
0.041166	0.10382	0.038275	-0.099225	0.00083867	0.01905	-0.0964
-0.13499	0.68693	-0.24202	-0.1042	-0.1322	0.21584	0.14126
0.029531	0.45602	0.005263	-0.068725	-0.29367	-0.40737	-0.078101
0.13163	0.12456	-0.0045706	0.13505	0.17716	0.06547	0.59227
0.021581	0.066009	-0.14748	-0.035798	0.032436	0.18174	0.059839
0.018842	0.30234	-0.039567	0.084311	0.19176	0.16219	0.019579
-0.03768	0.50642	-0.0013502	0.059712	0.1604	0.12699	0.20395
-0.0098306	-0.38307	0.086716	-0.18356	-0.10792	-0.33616	-0.22529
0.14248	-0.68057	0.053856	0.14012	0.097072	0.059375	-0.032046
-0.089853	-0.5107	-0.10926	-0.13666	-0.17409	-0.18301	-0.10828
-0.20142	0.1274	-0.040566	-0.34189	-0.28163	-0.087218	0.10613
0.096707	-0.29718	0.065261	-0.063937	0.00031945	0.34492	0.32864
-0.12345	-0.53182	-0.011203	-0.060176	-0.16062	0.0098795	-0.088039
0.022733	-0.048396	0.063137	-0.018901	-0.20412	-0.24537	-0.20367
-0.0004426	0.91244	0.037968	0.062801	0.24879	0.45252	0.12865
0.021416	0.048269	0.13727	0.1713	0.08223	0.2389	0.15641
-0.08205	-0.19628	-0.0068218	-0.50274	-0.63581	-0.81753	-0.83327
-0.067989	-0.21263	-0.03352	-0.29204	-0.54686	-0.65989	-0.68118
-0.14777	0.23546	0.15041	-0.17419	0.082314	0.25659	0.28695
0.0026475	0.14681	-0.077681	0.080418	0.041198	-0.064087	0.23745
0.036353	0.082003	-0.18063	0.033483	0.14052	-0.063312	-0.26396
0.11603	0.062389	0.074674	-0.058143	0.33122	-0.043275	-0.08041
-0.16642	0.089903	-0.1374	-0.0030807	0.21863	0.13398	0.090273
-0.089832	-0.32229	-0.025558	-0.079059	-0.19833	-0.32303	-0.12662
-0.12386	0.28879	-0.051228	-0.090293	0.29867	0.081553	0.036425
-0.074369	-0.18203	-0.089639	-0.12693	-0.011838	0.29589	0.18434
-0.032203	-0.45894	0.0083566	-0.02081	-0.10369	-0.0072238	-0.23829
-0.031675	0.048527	-0.062473	-0.087797	0.055398	0.030872	0.04104
0.089769	0.56618	0.02841	0.15687	0.037188	-0.083819	0.11013
-0.016305	-0.067138	-0.065064	-0.082632	-0.093814	-0.07002	-0.0022943
0.03125	0.36982	0.1117	0.019931	0.24026	0.060414	0.3756
-0.18725	-0.26222	-0.23955	-0.46083	-0.18494	-0.61775	-0.26426
0.021154	0.46682	-0.046715	-0.016249	-0.15799	0.32116	0.089348

-0.14447	-0.34264	-0.060694	-0.083455	0.076303	-0.18207	0.19674
0.084893	0.044883	-0.026254	-0.1984	-0.21346	-0.13475	-0.041812
0.057852	0.3351	0.20635	0.010855	-0.0065594	-0.23362	0.0013171
0.09134	-0.70696	0.066676	-0.031337	-0.17774	-0.028006	-0.11983
-0.16931	-0.39903	-0.20701	-0.22805	-0.22575	-0.13131	-0.26877
0.15819	-0.66587	0.10774	-0.083073	-0.20382	-0.047987	-0.0001647
-0.080752	-0.1359	-0.12469	-0.16283	0.072285	-0.098524	0.12382
0.0098167	-0.011857	0.15254	-0.026147	0.137	0.18441	-0.1823
0.12516	-0.43647	0.083919	-0.12269	-0.10879	-0.20366	-0.30878
-0.20784	-0.19207	-0.32566	-0.38535	-0.67591	-0.60764	-0.47774
0.035882	0.40547	-0.010666	-0.16433	-0.099901	-0.027615	-0.089477
0.1079	-0.25282	-0.12033	-0.098302	-0.11779	-0.07533	0.15297
-0.15105	0.093955	-0.12631	0.13413	0.29549	0.2653	0.027365
-0.15915	-0.9788	-0.23313	-0.43397	-0.11316	-0.080739	-0.24453
0.17231	0.0011067	0.045383	-0.062809	-0.32623	-0.3905	-0.4399
-0.048659	-0.094602	0.026983	0.24038	0.19638	0.24828	0.21588
0.026142	-0.23691	-0.046534	-0.014975	-0.101	-0.14055	-0.36874
0.074562	-1.542	0.025028	-0.19026	-0.090264	-0.2415	-0.3528
0.040767	-0.079727	0.010175	-0.019236	-0.016302	0.084688	0.13017
0.082958	0.050436	0.058489	-0.017026	-0.034896	0.14496	0.067957

test_GSE439	test_GSE439	test_GSE439	test_GSE439	test_GSE439	test_GSE439	test_GSE439
ref_GSE4394	ref_GSE4394	ref_GSE4394	ref_GSE4394	ref_GSE4394	ref_GSE4394	ref_GSE4394
GSE4394	GSE4394	GSE4394	GSE4394	GSE4394	GSE4394	GSE4394
GEO	GEO	GEO	GEO	GEO	GEO	GEO
cDNA	cDNA	cDNA	cDNA	cDNA	cDNA	cDNA
RIFAMPICIN:	RIFAMPICIN:	RIFAMPICIN:	RIFAMPICIN:	RIFAMPICIN:	RIFAMPICIN:	RIFAMPICIN:
548	549	550	551	553	554	555
-0.20243	-0.028563	-0.60861	-0.069357	-0.42189	-0.41724	-0.57465
0.083196	0.16675	0.63315	0.32789	0.18513	0.18269	0.32628
-0.066652	-0.0015633	0.063591	-0.081182	-0.075168	0.25953	0.01553
-0.0006105	-0.025803	-0.18479	-0.26224	0.20021	0.049675	-0.15917
-0.018265	0.052149	0.0038331	0.26428	-0.089775	0.111	0.11775
-0.015525	0.026999	-0.96772	-0.63921	-0.29794	-0.22163	-0.64856
-0.88404	-0.71929	-1.0316	-0.95606	-1.5699	-1.3287	-0.98033
-0.040425	-0.048339	0.19866	0.1763	-0.058888	-0.18775	-0.27192
0.17426	0.17515	-0.16684	0.13368	0.013223	-0.23556	-0.03916
-0.11834	-0.04516	0.010369	-0.023812	-0.19063	0.14603	0.16188
-0.18024	-0.12439	0.18432	-0.43055	-0.29271	0.055837	-0.18548
0.087193	-0.12429	0.070484	0.16728	-0.24064	-0.16614	0.14633
0.031478	0.11688	0.13476	0.44053	-0.049566	0.073831	0.0062297
-0.035839	-0.0062991	-0.23196	0.11193	-0.34826	0.069775	0.28401
0.11099	0.20812	0.10144	0.36804	-0.014921	0.14043	0.15134
-0.021759	0.12024	0.07746	-0.16973	-0.36481	0.16275	0.090247
-0.15568	-0.10567	-0.079692	0.10179	-0.45811	-0.38374	-0.8473
0.10087	0.13789	0.011903	0.19688	-0.21481	-0.22433	0.014448
-0.024493	-0.069259	0.023777	0.20333	-0.39241	-0.45128	-0.075021
0.1886	0.068889	-0.53247	0.27108	-0.40229	-0.67662	-0.43238
0.21989	0.18777	0.15241	-0.14304	-0.22546	-0.13999	0.19194
-0.11912	-0.13889	-0.45106	0.16135	-0.14889	-0.163	-0.12689
0.11671	0.081413	-0.53993	-0.66105	-0.29234	-0.5887	-0.58936
-0.020637	-0.053874	0.025162	0.34803	-0.15277	-0.2043	0.0094485
0.026566	-0.12556	0.24661	0.15517	-0.054006	0.10461	0.029807
-0.16542	-0.31817	-0.36437	-0.12766	-0.1369	0.011745	-0.32103
0.075681	-0.08039	0.25735	0.30298	-0.26559	-0.027133	0.099231
-0.080595	0.14393	0.55726	0.85142	-0.015024	0.39402	0.1403
-0.017221	-0.008696	-0.11014	-0.3412	0.01169	-0.56285	-0.27868
-0.16324	-0.45069	0.0042993	-0.26718	-0.36231	-0.24093	-0.40937
-0.54937	-0.35163	-0.85034	-0.83443	-0.54361	-0.69879	-0.81199
0.13776	0.030797	-0.81381	-0.71396	-1.0184	-0.48163	-0.7773

-0.12619	0.0032306	-0.14799	-0.041304	-0.15871	0.054597	-0.14057
0.27645	0.057271	0.21117	0.28945	0.036159	-0.0033738	0.044073
0.10067	0.17988	-0.24532	-0.097738	0.017886	-0.3222	-0.16405
0.089568	0.24579	0.30056	0.14242	0.13109	0.035212	0.11775
-0.045502	-0.10132	-0.21974	-0.14108	-0.24562	-0.35233	-0.31398
-0.46957	0.0062139	-0.20622	-0.11304	0.15506	-0.41932	-0.45766
-0.12071	0.23405	-0.066035	0.024853	-0.15263	-0.26177	-0.31675
0.25446	0.1979	0.41979	0.3509	-0.097188	0.13438	0.17755
0.0032584	-0.11392	0.12134	0.30261	-0.15193	-0.083545	-0.018775
-0.10835	-0.1653	-0.052558	0.018013	-0.20834	-0.17533	-0.2519
-0.012689	0.036205	0.012507	-0.12211	0.19396	0.30485	0.098891
0.10029	-0.18327	-0.33552	-0.48241	0.090406	-0.14001	-0.17312
-0.071412	-0.1858	-0.22507	0.067141	-0.091615	-0.32001	-0.062863
0.026676	-0.057653	-1.0528	-0.44102	-0.3753	-0.29198	-0.47951
0.16501	-0.060797	-0.79093	-0.4874	-0.5504	-0.31093	-0.7204
-0.037432	-0.014878	-0.41521	-0.35565	-0.35386	-0.21595	-0.28557
0.31315	0.095264	0.088258	0.30292	0.03965	0.078995	-0.038005
-0.03652	-0.074196	-0.0006747	-0.038485	-0.11526	-0.4072	-0.23277
-0.41438	-0.1606	-0.45171	0.043005	-0.21912	-0.26047	-0.66148
-0.070072	-0.077062	0.087651	0.11052	-0.031668	-0.036027	-0.068134
-0.058541	-0.023472	-0.055021	-0.094148	-0.61926	-0.29745	-0.38128
0.050043	0.28271	-0.38835	-0.21163	0.28985	-0.1131	0.31545
-0.85799	-0.42685	-0.27735	-0.52446	-0.31868	-0.56804	-0.70273
0.33384	0.1189	-0.2798	-0.35887	-0.29162	-0.06227	-0.0066414
-0.19039	-0.072633	-0.31616	-0.088055	-0.2692	-0.19489	-0.15737
-0.15772	0.10136	0.061311	0.14836	-0.36107	-0.08257	-0.41668
-0.030888	-0.0092432	0.32045	0.24095	-0.10494	0.019444	0.16289
-0.25847	-0.0723	-0.13988	0.46832	-0.078497	-0.027303	-0.035534
0.27404	0.10738	0.31474	-0.080584	-0.37344	-0.085564	-0.29185
-0.17563	-0.081338	0.12519	0.0036364	0.12337	-0.41849	0.17748
-0.053884	-0.19825	-0.20228	0.043518	0.048701	-0.44536	0.25374
0.26676	0.15018	-0.46813	-0.19868	-0.26593	-0.25307	-0.16643
0.12808	0.24816	-0.1249	0.21876	-0.14808	-0.017544	-0.24905
-0.033277	-0.067721	0.53464	0.46535	0.53503	0.1774	0.40767
-0.1682	-0.16521	-0.07488	-0.35279	-0.42924	-0.23549	-0.70005
0.10311	0.080708	-0.39639	-0.19397	-0.28157	-0.27993	-0.211
-0.063088	-0.18534	-0.22688	-0.26136	0.044153	0.14529	-0.10078
0.10749	0.16996	0.036163	0.35776	-0.033917	0.15611	0.24777
-1.1141	-1.0629	-1.9082	-1.5757	-1.7951	-1.5865	-1.2978
-0.16752	-0.32745	-1.1557	-0.42124	-1.1964	-0.35917	-0.41729
-0.15427	-0.37585	-0.048385	-0.23587	-0.28409	-0.13518	-0.5932
0.43765	0.26021	0.72384	0.68204	0.28937	0.34702	0.64104

-0.091765	-0.17368	-0.045502	0.0036663	0.018246	0.065751	0.18664
-1.3109	-1.5326	-0.89651	-0.64146	0.67106	0.79939	-0.11244
-0.44832	-1.0157	-0.95844	-1.3835	-0.47755	-0.77275	-1.3887
0.0073507	0.08108	-0.12324	0.18493	-0.13369	-0.11336	-0.1006
-0.073358	0.10572	-0.075211	-0.019852	-0.097608	0.010555	0.13054
-0.0020628	-0.09068	0.67976	0.0067775	0.16789	-0.10912	0.030879
0.38413	0.17846	0.60506	0.64815	-0.14253	-0.29369	-0.039083
-0.032057	0.10031	-0.47242	0.1556	-0.20551	-0.2558	-0.46113
-0.35163	-0.4082	-0.040952	-0.094365	-0.7176	-0.62064	-0.4797
-0.72557	-1.2225	-1.197	-1.1055	-1.4397	-1.5897	-1.2505
-0.13478	-0.15454	-0.12297	-0.051896	-0.58912	-0.33026	-0.4441
-0.12543	0.0092512	0.3428	-0.28774	0.010006	0.17107	-0.012161
-0.35592	-0.3478	-0.60547	-0.52596	-0.83125	-0.7109	-1.0674
0.12014	0.28411	-0.14462	-0.21993	-0.36603	-0.13473	-0.26825
-0.49413	-0.73179	-1.4653	-1.0345	-1.0431	-0.55727	-0.99329
0.13386	-1.54E-05	0.238	0.42006	-0.09376	-0.024684	0.19105
0.092605	0.10755	-0.1583	-0.10852	-0.27741	-0.21131	-0.14671
-0.34055	-0.29388	-0.28779	-0.12609	-0.14055	-0.15862	-0.22264
-0.011633	-0.11561	-0.23447	-0.34319	-0.45975	-0.13987	-0.56797
0.042704	0.13138	-0.29266	-0.36251	-0.10907	-0.28449	-0.20868
-0.085946	0.21793	-0.041028	-0.10705	-0.0009457	0.16889	-0.023724
-0.23789	-0.092635	-0.15084	0.30696	-0.1111	-0.015106	-0.082118
-0.16723	0.016257	0.15794	-0.175	-0.34797	-0.12586	-0.2546
-0.12201	0.069068	-0.14738	0.30096	-0.055505	-0.135	-0.019913
0.13487	0.065633	-0.14305	0.27423	-0.45112	-0.14094	0.1544
-0.12907	0.038476	-0.5901	-0.53238	-0.3877	-0.3446	-0.49103
-0.012002	0.12302	-0.27456	-0.30668	-0.054968	-0.27121	-0.37791
-0.23206	-0.16686	-0.96623	-0.63309	-0.7126	-0.62945	-0.75881
0.15856	-0.30467	0.13293	-0.022918	-0.21756	-0.076223	-0.088684
0.29722	-0.27285	0.29752	0.31387	-0.24652	-0.1178	-0.077992
0.092362	-0.068844	-0.42652	-0.13886	-0.30263	-0.51976	-0.31896
0.094673	0.21628	0.55486	0.38163	0.20037	-0.019146	0.011361
0.26828	0.18167	0.090409	0.094756	0.15858	-0.03355	-0.01418
0.25299	0.1167	0.30879	0.062662	0.12098	0.11807	-0.4336
-0.74574	-0.92012	-0.6771	-0.85919	-0.81748	-0.63606	-0.76215
-0.40914	-0.34077	-0.88745	0.0051018	-0.76928	-0.69615	-0.60428
-0.16324	-0.072801	0.2122	0.34296	-0.03814	-0.19886	0.31653
-0.65449	-0.50001	-1.6961	-0.6041	-1.7248	-0.97105	-0.82909
-0.12026	-0.075891	-0.37736	0.11743	-0.29557	-0.21088	-0.099997
0.050646	-0.086435	-0.033106	-0.1197	-0.070464	-0.12057	-0.38064
-0.094656	-0.031994	-0.42782	-0.40663	-0.20745	-0.4736	-0.58878
0.20322	0.086051	-0.38805	-0.094859	0.16288	0.19498	-0.15446

0.1557	0.10031	-0.2515	-0.070282	-0.32637	-0.23006	0.054349
-0.10045	-0.38146	-0.81349	-1.1319	-0.28151	-0.35062	-1.0848
0.0050528	0.16124	-0.13366	0.08726	-0.19272	-0.074073	0.26125
0.12678	-0.14179	0.10824	0.094202	-0.043429	0.0049766	0.09029
-0.39418	-0.15752	-0.70747	-0.52484	-0.29286	-0.38736	-0.50903
0.48661	0.28315	0.8658	0.46143	0.13001	0.079914	0.43498
-0.10889	-0.074292	0.23403	0.080679	0.18032	0.025276	0.15588
-0.29639	-0.25569	-0.33324	-0.036983	-0.16591	-0.39663	-0.19772
-0.028822	-0.23946	0.014415	-0.093454	-0.22274	0.058665	0.12433
-0.11634	-0.22754	-0.045221	-0.25756	-0.73252	-0.46823	-0.36589
-0.32711	-0.028896	0.457	0.10331	0.086518	0.075969	0.13862
-0.4441	-0.59165	-0.40373	-0.60234	-0.30341	-0.40198	-0.93153
-0.05873	-0.1689	-0.46559	-0.76872	-0.61044	-0.73157	-0.95425
-0.064844	-0.21459	-0.69855	-0.45993	-0.58698	-0.3623	-0.85337
0.05939	-0.21824	-0.88672	-1.1751	-1.1309	-1.2541	-1.6013
-0.69557	-1.0678	-1.0313	-0.95408	-0.8097	-0.7346	-0.87734
-0.18946	-0.18312	-0.40795	-0.25101	-0.39199	0.019543	-0.10533
-0.18372	0.21915	0.10533	0.27432	0.011383	-0.037885	-0.092289
-0.58103	-0.56216	-0.94178	-0.47494	-1.0324	-1.1132	-0.88162
-1.5926	-1.9011	-0.014712	-0.12729	2.1344	2.081	1.6347
-0.24531	-0.21621	0.089694	0.10169	0.021049	0.13907	-0.014605
-0.14297	-0.055879	0.054897	0.14256	0.069901	0.18054	0.19796
NaN	NaN	NaN	NaN	NaN	NaN	NaN
-0.25995	-0.27418	-0.32398	-0.24507	-0.48003	-0.11656	-0.61229
-0.22924	-0.35914	0.14005	0.11799	-0.16569	-0.21827	0.01768
-0.21171	-0.0021337	0.3206	0.52648	0.10802	-0.18183	0.097545
-0.29017	-0.32735	-0.15924	-0.26081	-0.18907	-0.081023	-0.39694
-0.13786	-0.15767	-0.14601	-0.30926	-0.071018	-0.064614	-0.26397
0.039347	-0.22761	-0.58925	-0.27895	0.010254	0.261	-0.3285
-0.14665	-0.32203	-0.50783	-0.63826	-0.97764	-0.80973	-0.77439
0.13831	0.17774	-0.36052	0.15926	-0.19142	0.44544	-0.11574
0.14649	0.1987	-0.18227	0.30708	-0.74608	-0.11283	0.12551
0.31083	0.19018	0.036283	0.13109	0.09404	0.23057	0.015378
-0.07836	0.050517	-0.13422	0.34525	0.048472	-0.20134	0.15162
0.072962	0.13307	0.066218	0.18903	-0.0039342	0.10332	0.16055
0.066876	0.14086	0.31832	0.22709	0.082135	0.1422	-0.19974
0.06455	0.12917	-0.083904	-0.51279	0.22211	-0.044808	-0.28745
0.18571	0.023687	0.04392	-0.25959	0.11858	-0.16166	0.10329
0.13669	0.081006	0.17921	0.51978	0.045291	0.087259	0.30114
-0.16068	0.13821	-0.012907	-0.13536	-0.067859	0.15288	0.05142
-0.15714	0.0053093	-0.11928	0.1654	-0.084528	-0.030032	-0.13223
-0.45023	-0.65209	-0.50401	-0.239	-1.03	-0.45455	-0.71436

0.13541	-0.087031	0.44814	0.50427	0.12619	-0.020866	-0.029667
-0.0032839	-0.075599	0.36256	-0.1135	0.12876	0.068772	0.021273
-0.28641	-0.45355	0.10876	-0.054652	0.023804	0.037164	0.0085671
-0.47963	-0.25315	0.0090651	-0.23731	-0.4606	-0.14329	-0.40548
0.065681	0.049267	0.006976	0.089848	0.18113	0.23255	0.28866
0.00285	0.18797	0.25924	0.71019	-0.018413	0.10163	0.31262
-0.6126	-0.55884	-1.4352	-1.046	-0.47567	-0.82076	-0.98715
-0.60668	-1.1977	-0.64406	-1.1319	-0.12891	-0.42601	-0.14174
0.078761	0.027344	-0.81649	-1.2354	-0.9583	-0.73392	-0.96272
0.1791	-0.11875	0.18101	0.11422	0.079386	0.022291	0.2598
-0.27325	0.0055471	0.11381	-0.12753	0.36102	0.23409	0.26008
-0.15755	-0.031004	0.074939	-0.041975	-0.13688	-0.12809	-0.045956
0.061255	0.2206	0.069609	0.24655	0.090997	0.10383	0.39215
-0.015392	0.16446	0.40042	0.10962	0.16655	0.12958	0.027153
-0.31189	0.052312	1.1108	1.0848	0.14337	0.18606	0.57949
-0.50709	-0.23337	-0.18137	-0.24657	0.076683	0.061698	-0.18849
0.15717	-0.13684	0.18036	-0.079	0.12587	0.36556	0.3146
-0.19113	-0.15493	0.17525	0.19534	0.17641	0.23544	0.30104
-0.4272	-0.42274	-1.288	-1.1951	-0.16297	-0.22838	-0.56628
0.19429	0.17066	-0.21775	-0.15322	0.18871	0.29555	0.11283
0.0050861	0.027245	-0.078741	0.096685	0.039637	-0.14582	-0.032633
-0.072081	0.031505	-0.018742	0.52344	0.11873	-0.12665	0.014204
-0.168	-0.33921	-0.025723	0.056846	-0.36744	-0.18366	-0.26981
-0.10835	-0.17065	0.11809	-0.10783	0.063645	0.18594	-0.069039
-0.5508	0.21285	-0.31946	0.10783	0.41557	0.263	0.23571
-0.053016	-0.081817	0.04425	0.44933	-0.38575	-0.08414	0.35921
0.34199	0.082261	0.11603	-0.11309	-0.047087	0.097863	0.12906
-0.10102	-0.19972	-0.30568	0.15983	-0.24563	-0.19841	-0.25642
0.029379	0.060319	-0.87086	-0.41145	-0.88622	-0.27105	-0.58271
-0.39682	-0.33099	-0.47642	0.1324	-0.49903	-0.12577	-0.042121
0.014635	-0.0077459	0.052765	-0.091881	-0.049467	-0.080701	-0.14038
-0.2044	-0.031886	-0.34421	-0.65142	-0.17883	0.017497	-0.22756
-0.13197	-0.43091	-0.10369	0.10093	-0.36976	-0.22898	-0.21802
-0.1779	-0.26536	0.042024	-0.021237	-0.053175	0.082897	-0.025815
0.41703	0.3206	-0.26397	-0.0075883	-0.038654	0.021287	-0.041364
-0.65154	-0.68774	-0.47593	-0.3428	-0.38985	-0.26394	-0.45275
-0.1087	0.080664	0.27324	0.15251	0.20772	0.14014	0.32173
0.028994	-0.061187	-0.37357	-0.41047	-0.58747	-0.26683	-0.58233
-0.14776	-0.30388	-0.89311	-0.48368	-0.62285	-0.41966	-0.65862
0.014696	0.071836	-0.14149	0.14704	-0.27755	-0.41352	0.18789
-0.48296	-0.11581	-0.26705	-0.209	-0.32827	-1.0799	-0.62668
-0.21043	-0.26402	0.14532	-0.17203	-0.39579	-0.0627	-0.17185

-0.47184	-0.35138	-0.4012	-0.36684	-0.36808	-0.26146	-0.72719
-0.0782	-0.19035	0.19068	0.05172	-0.0073743	0.14174	0.008329
0.027918	-0.33332	-0.20734	-0.26931	0.022962	0.16831	-0.10305
-0.024782	-0.18525	-0.054737	-0.075495	-0.36455	-0.19507	-0.31464
0.21803	0.055822	-0.14922	0.020813	0.09258	0.53575	0.075535
-0.5074	-0.93088	-0.50278	-0.54193	-1.0146	-0.86338	-1.0134
-0.19244	-0.093236	-0.1617	0.17095	-0.46196	-0.12076	0.087944
-0.009482	-0.18922	0.58224	-0.090085	-0.022074	-0.037315	-0.13597
0.04184	-0.13768	0.086731	0.15782	-0.13612	-0.076202	0.10417
-0.62877	-0.61333	-1.2608	-0.61276	-0.78455	-0.80133	-0.78616
0.10135	-0.092062	0.01745	-0.085203	0.11243	-0.024712	0.056128
-0.86611	-0.44085	-0.64852	0.071872	-1.1076	-0.70618	-0.68382
0.024823	0.18882	-0.039532	0.11609	-0.12447	-0.1431	-0.0092999
-0.17702	-0.024172	-0.16109	0.31307	-0.30081	-0.019146	0.14781
0.2776	0.089942	-0.43522	-0.45676	0.10552	0.33447	-0.013602
0.019365	0.023695	0.27596	0.29381	-0.0041405	0.22541	0.25879
-0.56859	-0.12139	0.13154	0.36189	-0.76119	-0.6564	-0.10125
-0.18428	-0.37591	-0.65155	-0.27479	-0.22664	-0.2303	-0.18822
-0.30167	-0.040169	-0.12642	0.33559	-0.63983	-0.14095	0.17758
0.17642	0.26535	-0.073192	0.22775	-0.058637	0.20598	0.18159
0.032697	-0.5365	-2.2591	-1.4009	-0.74573	-0.60344	-1.43
-0.085462	0.13116	0.0034475	0.60186	0.097787	-0.0005652	0.1235
-0.067441	-0.078081	-0.57582	-0.14649	-0.13475	0.25245	-0.093729
-0.69315	-0.73508	-0.3046	-1.0207	-0.77818	-0.90631	-1.2643
-0.17149	-0.25349	-0.3024	-0.12748	-0.24601	0.063699	-0.16843
0.12573	-0.12171	0.41522	-0.13152	0.25494	0.13679	-0.10432
-0.10145	-0.07927	-0.23436	-0.12728	0.0083799	-0.05684	-0.22105
-0.098082	-0.070082	-0.013319	0.097981	-0.37439	-0.26024	-0.64084
-0.33771	-0.31906	-0.56588	-0.21309	-0.78843	-0.39191	-0.94947
-0.61006	-0.65116	-0.92662	-0.80835	-1.7615	-1.6162	-2.8838
0.046044	-0.12636	0.32679	0.065549	0.093571	0.23207	0.16557
0.010561	-0.035637	-0.065063	0.13611	-0.26812	0.0028661	0.15686
0.079246	-0.008517	0.27611	0.050318	-0.33452	0.091233	-0.16603
-0.35886	-0.16306	-0.64541	-0.45928	-0.71006	-0.028803	-0.46813
-0.14822	-0.072802	-0.0026952	0.25185	0.0021399	0.017598	0.084128
-0.050279	-0.087379	0.15287	0.012074	-0.15701	-0.07862	-0.042753
-0.38318	-0.046702	-0.17608	0.43733	-0.29314	-0.15492	-0.12067
0.58362	0.27935	-0.70141	-0.48071	0.20042	0.22965	-0.34613
0.20581	0.21115	0.078416	-0.11656	0.27767	0.22453	0.031698
0.25116	0.32475	0.032905	-0.19611	0.3404	0.31453	-0.032737
0.22656	0.16513	-0.38514	0.18945	0.11482	0.085842	-0.0071154
-0.090029	0.25742	-0.19476	0.27229	-0.32289	0.064762	0.20194

-0.12058	-0.025409	-0.069484	-0.099364	0.23652	-0.1348	-0.24232
-0.074257	0.035501	0.19526	0.35057	0.016232	0.19048	0.10944
-0.059753	-0.59134	-1.2441	-1.6317	-0.4509	-0.56289	-1.9279
-0.47602	-0.17003	-0.24437	0.1398	-0.36008	-0.1461	-0.20045
0.11722	-0.020196	-0.051872	0.10167	-0.14409	0.064869	0.091331
0.19353	0.13483	-0.038441	-0.10939	0.34596	0.024267	0.18063
-0.21117	-0.37026	-0.059405	-0.66771	-0.12519	-0.41285	-0.86037
0.072965	-0.090677	0.0055732	0.11775	-0.074461	-0.19302	-0.037435
-0.0063695	0.030767	-0.046733	-0.10635	-0.12648	-0.042892	-0.28352
0.029609	-0.08172	0.12498	-0.23109	-0.23794	0.040138	-0.27892
-0.016166	-0.04545	1.1191	1.0776	0.46419	0.41795	0.73924
-0.14176	0.13159	-0.68744	-0.26216	-0.36822	-0.29899	-0.48154
-0.043872	0.16214	0.38457	0.18712	-0.33666	0.19062	0.10336
0.04408	0.047572	-0.04382	-0.093367	0.050851	-0.34581	-0.095856
0.17526	0.15349	0.52856	0.48491	0.13248	0.18784	0.11051
0.051013	-0.043743	0.066693	0.28302	-0.17397	0.18233	0.93302
-0.10898	-0.18087	-0.27736	0.087876	-0.40575	-0.0493	-0.14251
0.29459	0.050379	-0.96918	-0.33941	-0.60096	-0.28295	-0.43098
-0.091793	-0.33055	-0.27936	-0.1061	-0.54703	-0.27354	-0.37382
-0.35959	-0.025836	-0.17971	0.34853	-0.64428	-0.03911	0.27734
0.1104	0.054107	-0.10473	-0.046244	-0.059243	-0.013215	-0.13146
-0.22334	-0.14607	-0.45047	-0.46838	-0.55698	-0.29274	-0.39707
-0.02819	-0.088874	-0.17287	0.17918	0.0013803	0.046628	0.073338
0.47516	0.29974	0.14795	0.55914	-0.080824	0.013628	0.24702
0.10063	0.022683	0.17081	0.16328	-0.0061144	0.099734	0.21617
-0.57203	-0.30233	-0.59792	-0.14294	-0.39722	-0.37629	-0.29943
-0.24555	-0.34681	-1.3196	-0.49064	-0.50717	-0.53053	-0.32774
-0.29382	-0.30335	-0.036852	-0.013757	-0.74401	-0.14615	-0.1277
0.089973	-0.036413	-0.082508	-0.42829	0.14497	-0.17002	-0.07335
0.33	0.18311	-0.23045	0.50325	0.17575	-0.1627	0.1654
-0.13121	0.064516	-0.65885	-0.6675	-0.50651	-0.41763	-0.80219
0.015947	0.08902	-0.12236	-0.281	0.06641	-0.24185	-0.17676
-0.083097	-0.1322	0.39978	-0.28001	0.11333	-0.61788	-0.30963
0.14742	0.18271	-0.12182	0.01414	-0.23514	-0.012531	0.083976
0.084037	-0.043411	-0.13776	0.48362	-0.53648	-0.046632	0.1007
-0.12355	-0.27645	-0.30239	0.058273	-0.12968	-0.099557	0.019907
-0.028803	0.060518	0.12347	0.48384	-0.015918	-0.063958	0.21476
0.2081	-0.063291	0.29139	-0.11098	-0.27135	-0.079095	-0.034903
-0.14599	-0.24061	0.074	-0.035036	-0.52788	-0.17363	-0.29711
0.1213	0.14884	0.46618	0.4281	0.16354	-0.030417	0.2067
-0.41196	-0.19053	-0.67831	-0.26079	-0.44916	-0.51411	-0.23765
0.12291	0.042203	0.21303	0.20874	-0.086332	0.14515	0.30226

-0.23571	-0.13467	-0.65124	-0.20728	-0.084481	-0.34257	-0.23589
0.0083789	0.21415	-0.26634	0.068489	-0.25421	-0.19791	-0.047244
0.03515	-0.053547	0.27522	-0.08406	0.20724	0.0064559	-0.10644
-0.10639	-0.079493	-0.47068	-0.34635	-0.2013	-0.07471	-0.24278
-0.08719	-0.037745	-0.61546	-0.54909	-0.40049	-0.34545	-0.52229
0.038934	-0.31763	-0.48317	-0.59745	-0.24324	-0.36094	-0.54454
-0.011151	0.1353	0.22161	-0.12266	0.017705	-0.35953	-0.0041026
-0.088969	-0.04254	0.25699	0.034195	0.0063736	-0.25315	-0.077659
0.014713	-0.07445	-0.68643	-0.2289	-0.94651	-0.32585	-0.14526
-0.31919	-0.4243	-0.38579	-0.36404	0.08083	-0.35819	-0.36709
-0.15079	-0.072253	0.34393	0.40605	-0.27341	-0.10044	0.14028
0.21409	-0.19736	-0.15755	0.044208	-0.23322	0.0341	-0.062082
-0.093309	0.10376	-0.020965	0.16127	-0.089613	0.037337	0.16017
-0.43328	-0.28859	-0.21302	-0.064349	-0.9152	-0.30941	-0.24692
-0.28037	-0.50274	0.15004	-0.31492	-0.2781	-0.23864	-0.51789
0.15588	0.29016	0.0844	0.22049	0.28991	0.074727	0.38672
0.034732	-0.0022092	-0.63001	-0.30444	-0.63537	-0.25732	-0.26048
0.056021	-0.077875	-2.138	-1.2594	-2.0409	-1.4665	-1.9527
0.10912	0.13246	-0.25117	-0.30889	0.074988	-0.32816	-0.12112
0.11911	0.0037245	-0.19092	-0.48606	0.1467	-0.59368	-0.33705

test_GSE439	test_GSE598	test_GSE312	test_GSE671	test_GSE671	test_GSE920	test_GSE920
ref_GSE4394	ref_GSE5981	ref_GSE3123	ref_GSE6712	ref_GSE6712	ref_GSE9203	ref_GSE9203
GSE4394	GSE5981	GSE3123	GSE6712	GSE6712	GSE9203	GSE9203
GEO	GEO	GEO	GEO	GEO	GEO	GEO
cDNA	cDNA	cDNA	p33	p33	affymetrix	affymetrix
RIFAMPICIN:	rsd_b3995:1	pM30:-1,pRN	EVO_BENZAL	EVO_BENZALKONIUM_CH		lsrR_b1512:-
556	627	659	721	726	959	961
-0.62297	0.2116081	0.21263	-1.118329	-0.239033	0.5279144	1.253149
0.6581	0.8435462	0.027525	1.493606	-0.4261993	2.714668	2.992377
0.0029761	0.59116	0.48738	-0.8309046	0.06492689	0.1477353	0.6885945
-0.17557	0.3097922	0.08558	0.422784	0.09329149	-0.0247039	-1.103812
0.3266	0.1457679	0.17648	-0.5099867	0.14925	-1.359731	-0.9852861
-0.55231	0.1378507	-0.0048601	-0.6362809	0.1264235	0.5780292	0.5190854
-0.56702	-0.0221506	0.3598	-0.1448108	0.01486317	1.78908	0.5415429
-0.041716	-0.0227111	0.28768	-0.517643	0.3352911	1.260195	1.023712
0.011938	0.5335641	0.028739	0.472821	-0.6675488	0.3074053	0.08519033
-0.14938	-0.2324409	-0.17944	-1.008292	-0.185267	-0.0183853	-0.2806374
-0.061437	0.01913998	0.18563	-0.1071142	1.544986	2.974378	0.6808127
0.28375	1.002758	-0.17713	0.8855317	1.357666	-0.1312066	-0.3991083
0.25222	-0.106314	0.42453	-0.434034	-1.637179	-0.1044509	0.1971423
0.051092	0.6102277	-0.2426	-0.6432289	-3.881306	-0.6831375	-0.3719952
0.36763	0.0674858	-0.13247	-0.1773515	0.4944439	-0.100918	0.3666757
-0.066047	0.7247476	0.22326	-0.6979327	-1.806126	-0.5052879	-0.1804162
-0.976	0.6940264	-0.48255	0.8079183	-0.0249832	-0.4867907	-0.4750728
-0.0049252	0.04104031	0.3166	0.4591397	2.032895	-0.5515019	-1.020146
-0.21204	-0.0456136	0.050031	-1.639587	-3.475288	0.6246575	0.3137406
-0.60053	0.2106658	-0.070146	-1.411487	-3.878125	1.878365	0.3709332
0.32721	0.2006331	0.19917	0.1512835	0.3377062	-0.5677531	-0.6088459
0.10615	0.04561028	4.3124	-0.2130819	0.1815987	-0.1924774	0.1569206
-0.0026319	0.02715583	0.86176	-0.2280865	-2.732183	0.1209801	-1.237052
0.10934	-0.1581447	0.31021	0.4620754	1.682383	0.5067542	0.2418501
-0.17897	-0.032078	-0.24315	0.27967	0.531649	0.4016073	0.4934429
-0.23669	0.3748665	-0.0056916	0.3707774	-0.6847044	-0.0450605	0.03064127
0.46601	0.5846011	0.29028	1.786509	-0.9141478	1.440761	0.6804647
0.43422	0.2413434	-0.22551	0.3183344	-4.699037	0.9142448	0.8182082
-0.057136	0.3033921	-0.21682	-0.9160352	-1.857815	1.04232	0.2364008
-0.16795	-0.1502073	-0.42854	-1.772408	-1.503892	1.12249	0.5382334
-0.57962	0.6430107	0.15084	0.3250258	-1.823786	1.956833	1.850706
-0.98334	0.09636552	-0.093099	1.192794	1.142755	0.04467536	0.02197286

-0.031169	0.0139379	-0.24548	-0.6120187	-2.475707	0.3063171	0.08680406
0.20234	-0.0058929	-0.27088	-0.000775	-0.8445728	0.4129008	-0.5151907
0.036613	-0.1693676	-0.22437	0.02493219	-0.7715345	-0.7274675	-0.0817713
0.52487	-0.10178	0.052468	-1.084872	-3.505053	-1.304441	0.70365
-0.02977	-0.0828274	0.18813	-0.8285153	1.024517	-0.2061625	-0.3336723
-0.12433	-0.0463567	0.021409	-0.4125211	-1.752362	0.3632872	0.6334066
-0.034269	-0.1401331	0.10664	0.3538924	0.3218727	0.3765475	-0.5036529
0.098259	0.06888274	-0.044394	-0.456927	-0.0576006	0.0060056	-0.3952568
-0.10428	-0.0402368	0.20849	-0.5393887	0.2013822	-0.4781346	0.2273434
-0.16103	-0.0806936	0.32517	-0.0627714	0.4780686	1.276513	0.1343771
-0.060416	-0.0229115	0.25281	-0.7214375	-0.2865736	-0.8918773	0.07851479
-0.046797	1.224701	-0.22923	-1.640048	-4.754666	0.09469976	-0.1881227
0.06337	0.9501505	0.18511	-0.5380406	-0.282666	-0.0955651	-0.3198758
-0.93605	0.03277197	-0.037957	-0.5204329	-0.3542099	0.134652	0.03275261
0.07889	-0.0684503	0.031078	0.1877364	-2.024615	0.2118509	-0.0261391
-0.33276	NaN	0.25046	-0.6245652	-0.2686429	-0.5896047	0.2941961
0.17649	0.01612141	0.2573	-0.5550173	-0.7952292	0.6157175	0.2414175
-0.051443	-0.136461	-0.37467	-0.716871	-4.500566	-0.3430496	-0.057383
-0.19427	-0.1840112	0.34708	-0.8823161	0.3324905	0.1442824	0.07168071
-0.11153	-0.053633	-0.092633	-0.5320607	-0.0182367	-0.0977264	-0.110209
-0.289	-0.1564496	-0.2057	0.6790113	-1.571097	-0.0994731	-0.2357454
0.13901	0.02286994	0.24302	-0.000775	0.08447246	0.08368418	0.05566814
-0.33979	0.9867598	0.27245	-0.4979007	-2.365374	1.22588	0.4105986
-0.6075	-0.1715928	-0.21388	0.4044216	-0.6414114	-0.3352162	-0.4010392
-0.47321	1.242054	0.33617	-0.8498671	0.4343389	1.985902	1.228689
0.0096935	-0.4022959	0.16507	-1.065117	-2.252096	0.4237265	0.5210046
0.2571	-0.1456478	-0.058927	0.03330899	0.4393307	0.07319349	-0.2175523
-0.077878	0.5699699	0.074283	-0.9100323	0.4667927	-0.3981106	-0.2895802
-0.12679	0.7025711	0.11203	-0.1214116	-2.916291	-0.6559516	0.0738023
0.11212	0.1785355	-0.40509	0.00456633	1.106168	1.643674	0.9890916
0.053055	-0.0062735	-0.3766	-1.401272	-0.0180808	3.121063	3.130699
-0.035062	-0.1184	-0.267	-0.487436	-1.100065	1.416834	0.9254726
-0.25233	0.9025302	-0.49913	-0.2063194	1.269017	1.901413	0.9356903
0.81185	0.4363799	-0.74343	0.4876385	0.5835804	-2.187974	-0.1525484
-0.53386	0.05385954	0.13551	-1.960382	-4.411422	-2.144173	-0.9082197
-0.10844	0.1464411	0.23375	-1.038324	-1.316109	0.7557821	0.3469143
-0.063317	-0.3130985	-0.26266	-0.1002512	-0.6233856	-0.1377659	0.2872189
0.3767	0.03849441	-0.14442	1.509242	-0.0152613	-0.1839071	-0.40915
-1.8547	0.03477262	-0.054756	-0.000775	-0.0708362	0.5064929	-0.1066414
-0.95593	-0.103025	-0.045312	0.2610715	0.4284828	0.7300391	0.3645012
-0.28052	0.1622916	0.02861	-0.0209942	0.03513766	-0.4927694	-0.3548155
0.78417	-0.6073118	0.39162	-0.4803149	-2.759056	-0.4308013	0.4662198

0.23279	0.802173	-0.2295	0.6047619	-3.391781	0.1112152	0.5176829
-0.48739	0.08489686	0.45617	-0.5098831	-2.309758	-0.1911957	-0.6930346
-1.228	-0.1133068	0.13594	-0.046954	-0.700057	0.00338366	-0.679596
-0.22191	0.9309702	0.44196	1.498636	1.407379	0.1818978	0.308356
0.33237	-0.0813388	0.17543	-1.043345	-1.293193	0.6757486	-0.196947
0.6061	-0.1127797	0.11337	-0.1071142	-0.282666	0.4410714	-0.3990083
-0.01348	-0.2490834	-0.18969	0.09744624	-1.418074	-0.6515614	0.3306883
-0.51407	-0.0502172	-0.13552	0.1099987	-0.9665234	0.1023682	0.05288675
-0.15174	-0.0399471	0.090572	0.2043825	0.3624755	0.01701199	0.2828478
-0.75485	-0.4318042	-0.06902	-1.039588	-2.034114	-0.3746041	0.4365453
-0.13247	-0.021879	0.22995	-0.0035141	0.5165915	0.8737951	0.2101674
0.16663	0.07495733	-0.0098853	0.06108604	1.600332	-0.0130339	-0.3247611
-0.67559	-0.179549	0.41662	-0.6801842	0.1581374	0.0859761	0.3573302
-0.094789	-0.1964205	-0.11983	-0.4566679	1.124901	1.767337	0.8928574
-1.0735	-0.2125382	0.43166	-0.4283142	0.3880045	0.8594952	0.3943725
0.46719	-0.167772	0.071828	1.006979	1.289655	-0.3169678	-0.1635447
0.028992	0.2501065	0.27449	0.07193615	-1.037978	0.392298	0.323267
0.39159	-0.2014069	0.091239	0.1662452	0.2426088	-0.3214836	-0.3751911
-0.57029	0.01696114	-0.083899	-0.0463878	-0.317245	-0.1121986	0.9488568
-0.10836	0.4629353	-0.12127	0.4454732	0.03178064	0.2203838	-0.0575751
-0.18206	-0.0719701	-0.2281	1.306029	0.304506	0.6921812	0.4760637
0.12658	-0.031663	0.094068	0.02357474	-0.3088828	0.1941772	0.6241619
-0.11522	0.02367361	0.22684	-0.2731681	-2.695311	0.0024676	0.2059317
-0.063728	0.2315402	-0.034605	-0.5405842	-1.550582	0.2190985	-3.169571
0.12465	0.075532	-0.10584	0.3119763	-2.059163	-1.236819	-0.4949906
-0.51055	-0.2395783	-0.086812	-0.041404	-0.4692952	-0.1350405	0.484499
-0.46362	-0.2349077	0.18731	0.648365	0.5669626	-0.3156741	0.8690392
-0.26734	-0.3932851	-0.026037	-0.0434759	0.00832991	0.6423597	0.6916812
-0.32905	0.67719	0.053331	0.00426325	0.8892921	4.162391	2.303075
0.063152	0.025598	0.20905	0.2441305	0.3983664	-0.0235706	1.565628
-0.24545	0.19594	-0.49809	0.05330808	0.7202244	-0.1913994	1.313147
0.46537	0.15799	0.045442	0.53162	0.7652441	0.8591293	0.1798686
-0.17886	-0.24832	0.044879	-0.0460619	-3.369355	0.3394007	0.2041307
0.41426	-0.020177	0.18652	-0.1535428	0.1102844	-0.1198061	-0.7667622
-0.7048	-0.13119	0.50239	-0.8298721	0.613032	1.755026	1.121358
-0.41	-0.0630375	0.5103	-0.2048021	-0.0415124	-0.0053662	0.2053791
0.40698	-0.197308	0.089943	-1.069048	-0.5403408	0.05426433	-0.0803739
-0.99441	-0.047924	-0.041438	0.2118209	-0.065688	-0.3413208	0.4285577
0.26727	-0.1140884	0.21153	-0.2918693	-0.0324966	2.516587	1.067038
-0.10705	0.1761425	0.26905	-0.0044887	-1.557172	0.8592089	0.6082067
-0.45472	-0.2466484	0.15653	-0.3004044	-0.5696254	-0.1576485	0.8613949
-0.12842	0.1740478	0.26117	0.09211321	-0.0102316	0.3712991	0.4395894

0.046802	-0.0940264	-0.095593	0.3725058	0.6503567	-0.5345505	-0.0324128
-1.2471	-0.104018	-0.078925	-0.2355069	0.5288825	-0.3959518	-0.5401306
-0.02014	-0.1484169	-0.20884	-0.0686236	-0.0803834	-0.0213986	-0.2176052
0.20995	0.1400319	0.24841	0.3170135	0.1041516	0.2296852	0.1317852
-0.88617	-0.1704637	0.45492	-0.1034391	-0.0427771	0.02262584	0.2457867
0.85432	0.8498647	0.14479	-0.213757	-0.8460571	0.2926674	-0.220449
0.34739	0.08662981	0.022043	-0.4011762	-0.282666	0.2822337	0.7967561
-0.16302	0.04375558	0.079462	-0.2721502	0.01600935	1.835452	1.65724
0.025769	-0.076946	-0.049718	0.5014754	0.5364727	1.471177	0.2428818
-0.2474	0.04502999	0.33986	-0.4616337	-0.9127109	0.2816625	0.4702994
0.36714	0.4521953	-0.21543	-6.522051	-4.379118	-0.3271242	-0.1369991
-0.72634	0.00666793	0.96859	0.2588022	-0.0276176	0.7164625	0.7421892
-0.79012	-0.3006491	0.3088	0.4917168	0.5179079	-0.0577827	0.05972055
-0.55839	-0.1626206	0.91138	-0.5104543	-0.4785279	-0.677501	0.08563808
-1.2368	0.06528416	0.85464	-1.714788	-2.784269	-1.167667	-0.0482072
-1.0337	-0.5309811	0.13583	0.495424	-0.0776187	-0.0894192	0.4160751
0.047714	-0.1995736	0.015505	-0.7038241	-2.567906	0.8360586	0.7700217
0.37541	0.3431533	0.00095105	-0.0427399	1.645906	-0.4354125	-0.0172829
-0.9869	-0.2313548	-0.004941	0.6988271	1.06425	0.6451337	0.542908
0.71293	-0.008099	0.21188	0.2836941	-0.1561682	-0.4274739	0.3219405
0.0065685	0.2078977	0.066986	-0.0286608	-1.024846	-1.983554	0.7084382
0.33497	-0.02647	0.15405	0.07161019	-0.5393066	0.2192674	-0.0895526
NaN	NaN	NaN	NaN	NaN	NaN	NaN
-0.71522	NaN	0.33548	0.3617656	0.202812	0.7915363	0.5879735
0.27003	0.02262879	-0.054173	-0.9347236	0.340812	0.3997246	0.07019878
0.36993	0.4413615	0.29268	-0.7874208	-1.680158	0.6431026	0.502389
0.13369	-0.1020729	0.27775	0.61601	-0.6650212	0.9199544	0.9126796
-0.29501	0.1049033	0.14847	0.9427767	0.8815152	0.1886919	1.391645
-0.42314	-0.2133876	0.66601	0.6508111	0.6737529	0.3575778	0.688659
-1.2589	-0.0752188	0.18036	0.4002777	0.173221	-0.1904512	-0.5602148
-0.078829	-0.0131303	0.28085	-0.1071142	-0.282666	0.4815375	1.600112
-0.13342	0.04797308	-0.25402	-0.0876154	-0.1117975	0.8259026	0.08212828
0.010076	-0.1997929	0.013798	0.5095717	-0.3533567	-0.4040387	-0.203295
-0.030792	-0.0871371	0.090233	-0.566593	-0.5076261	0.4290537	0.2551928
0.23085	-0.2896753	0.11642	-0.5686519	0.2970342	0.2758839	0.3444209
0.179	-0.1088847	0.065013	-0.3896867	1.058535	0.6054777	0.5997033
-0.3372	-0.1809823	0.31776	-0.2623682	-0.1354996	0.5987111	0.3732238
-0.19569	0.0825312	-0.0032676	-0.8294555	-0.5549854	0.09079279	0.6587357
0.21699	0.0059767	-0.15452	-0.2852405	-0.282666	-0.3938374	0.1127654
0.26616	-0.2046441	-0.12983	0.6747443	-0.7823928	0.4394365	0.2068589
-0.04604	0.5352456	0.20854	0.8314818	0.7410607	1.572966	0.8211789
-0.43058	-0.4033329	-0.54236	-0.2861175	0.5174336	0.2123066	0.6989136

0.22257	-0.0767825	-0.068141	-0.1071142	-0.282666	0.2891888	-0.1655899
0.11087	0.4057586	0.24636	-0.7224511	-1.941046	0.9526123	0.09699922
0.12592	0.3242153	0.23604	0.1785264	-1.578954	0.2854317	-0.4217078
-0.32314	0.1336291	0.25591	0.09757059	1.235148	0.4152895	0.6073589
0.013565	-0.3019811	0.069661	-0.170149	0.08781801	-0.7137634	-0.4568454
0.3681	-0.0216566	-0.13529	-0.0182571	-1.073677	0.7092665	0.706834
-1.0521	1.215667	-0.054883	1.637251	1.082761	0.3233707	0.2065494
-0.44018	0.09084458	0.58451	-0.1477343	2.311915	0.6129021	-0.086857
-0.76382	0.3348488	0.2798	-1.59771	-1.646791	-0.8849189	-0.4135769
-0.32977	0.6991284	-0.08928	0.09161256	-1.245756	-0.296351	0.1251153
0.015267	-0.2267128	-0.075655	0.02067797	-0.6476532	-0.111631	0.4601054
-0.18271	-0.1842957	0.092794	0.17934	0.6844374	-0.2573136	-0.079625
0.65444	0.1219021	-0.051746	0.4313503	-1.3048	0.4093474	0.3033678
0.48649	-0.0974839	0.10577	-1.646694	-1.403814	0.1679532	-0.0093854
1.1951	NaN	0.14857	0.0495526	-2.18238	-0.5681783	-0.165993
-0.46273	-0.2016694	0.3133	0.2818405	0.8574792	-0.9199814	-0.3969866
0.53714	0.3116191	0.041766	-1.107321	-0.7156778	-0.630643	-0.5096747
0.40514	0.5555113	-0.22706	0.1448007	-0.2318033	-0.4002723	-0.0446368
-1.4043	0.3827645	-0.36834	0.2340382	0.5202671	-0.2146835	-0.2426339
-0.087009	0.00211231	-0.036748	-0.8055178	-0.5141281	-0.4811906	-0.00633
0.024318	-0.1846785	0.42294	-0.8513924	-1.654983	0.1034675	0.4027181
0.063232	0.1557906	-0.11883	-0.3522308	-1.105353	0.1362026	0.1711172
0.19746	-0.2821637	0.099313	0.5932353	0.02545754	0.4565593	0.6664701
-0.18123	-0.0604988	0.078728	0.1631992	-2.101735	-0.0216031	-1.13501
0.0036387	0.3735428	0.12537	0.04087682	-0.0173386	-0.4925294	-0.9267916
0.30643	-0.1498058	0.02454	0.03101601	-0.639727	0.6148066	0.4588109
0.42087	-0.1306341	0.013296	1.364304	0.7995748	-0.0114597	0.6455523
0.20141	-0.1945606	0.22032	0.1574012	-0.282666	0.2615929	0.6958786
-0.63118	-0.0453003	0.28826	0.4293665	-0.0592093	0.7162307	-0.4090493
-0.25097	-0.0304639	-0.42457	0.2791135	-2.024409	1.045231	0.080413
-0.12702	0.7458543	-0.15582	-0.000775	1.612431	0.5246509	-0.148377
-0.063678	-0.0639144	-0.18512	0.06823288	0.5995653	0.4956372	0.2847769
-0.021535	0.09204038	0.10201	-0.8084026	-0.0959348	-0.347128	-0.314982
-0.076532	0.5224535	0.18049	-0.1071142	-0.282666	-0.1902664	0.2367314
0.093746	-0.0481618	0.11225	-0.1071142	-0.282666	0.4263837	0.721873
-0.20603	0.1392411	-0.012858	-0.4168701	-1.627393	0.4083417	-0.1272338
0.56096	-0.007751	0.16241	-0.6971592	-0.2562896	-0.0862632	0.02691654
-0.30054	0.261048	0.010565	-0.9489096	-3.092877	-0.2571697	0.3695609
-0.71928	-0.10283	0.10703	-1.816805	-1.022106	0.653136	1.141793
-0.20413	NaN	-0.014096	-0.0305542	0.4531744	-0.5500522	-0.5906496
-0.45928	-0.0067926	-0.12479	0.4874438	0.8061545	0.7583076	0.2736461
-0.14822	-0.3657989	0.3103	1.918158	0.4770839	0.5354987	0.3948077

-0.39592	-0.0889133	0.12972	-0.1071142	-0.282666	-0.0970871	0.0380395
0.097958	-0.3371849	0.26062	0.7002225	-1.58441	-0.2843784	0.2040504
-0.10604	0.03216436	0.069404	0.1067114	1.229422	0.2271086	0.1987853
-0.21115	0.7256298	0.22801	-1.348539	-6.459389	0.7166726	0.1564684
0.080923	-0.150579	0.4203	0.312988	0.9060341	0.4038099	-0.0134002
-0.51469	-0.097481	0.096831	-1.574613	-1.097666	1.159919	0.9399376
0.00086327	-0.0512182	-0.2568	-0.5645952	-0.5511319	-0.7159389	-0.5797793
0.080043	-0.2257659	-0.27387	-0.6354831	1.302572	-0.1662937	0.5703823
0.15557	NaN	-0.2389	-0.1071142	1.659957	-0.2671524	0.5121614
-0.90643	-0.1689178	-0.080669	-0.5617727	-0.0567034	0.7482492	0.5902729
0.11381	0.1408398	-0.074344	-0.1071142	-0.282666	-0.1197463	0.1487446
-0.56759	-0.2414307	-0.21012	0.2313605	-0.0362117	0.7530934	0.0302443
-0.15748	0.5608226	0.015061	0.2464328	0.1542358	-0.0195314	0.2432954
-0.0087495	-0.1009934	-0.052571	0.3589662	-4.180617	-0.2431031	0.1007041
-0.87126	-0.4546132	1.0266	-3.082901	-2.692017	-0.8410497	-0.8683741
0.46739	-0.0564599	-0.092992	-3.431853	-0.0994079	-0.1533529	0.3716918
0.37943	-0.0116815	-0.027043	-0.1911881	-3.54903	-0.5709236	0.04610315
-0.76984	0.1373707	0.11061	-2.206934	-2.806277	1.010501	0.4998349
-0.11749	NaN	0.10648	0.5532693	-0.0281226	0.6863178	0.4792265
-0.19565	-0.1873004	0.012864	-0.964432	-2.603309	0.6407627	0.2506404
-2.3064	-0.0045938	0.54473	-0.8732829	-0.6104751	0.7743932	0.7381375
0.18167	0.1554899	-0.16156	0.09475561	-0.087354	-0.072198	-0.3948327
-0.17883	-0.0826939	0.018298	-0.4625005	0.812581	-0.4837876	-0.2598543
-1.1858	-0.1165519	0.3783	-0.0361492	-0.9082139	0.2158351	0.00434886
-0.25788	0.00901725	0.2635	-0.7610257	0.08926083	0.3589064	0.02061813
-0.21461	0.4915129	0.1012	-3.702778	-0.0163092	0.1938938	0.5387121
-0.55206	0.00613854	0.22226	-0.1626956	-0.0235002	0.07761381	0.4044907
-0.2496	0.3754853	0.19755	0.2590581	0.6222459	0.00027632	0.4343334
-0.91854	0.06177612	-0.010505	-0.3539443	-0.5213453	-0.300617	0.3449964
-2.0235	-0.0433808	0.18051	-0.8807128	-0.1935072	-1.470737	-0.0043567
0.38481	-0.0124662	0.14166	-0.6389879	0.2468791	-0.4344146	-0.5186355
0.042571	0.1752271	0.070324	-1.06973	-1.715285	-0.4706409	-0.4967239
-0.13728	0.2116219	0.13423	0.2871631	1.600522	1.280778	0.5769922
-0.8686	0.7222279	-0.95546	-1.633906	-3.468516	-0.0051937	3.015063
-0.0002849	-0.0170294	0.0050534	-0.0392413	0.2834221	-0.5585626	0.5565879
0.28267	-0.0714958	-0.13876	-1.759955	-4.34415	-0.9470446	-0.8077081
0.052473	0.1079667	0.10609	-0.3745608	-3.526407	0.7896021	0.4139562
-0.84498	0.1904421	0.23217	-0.1450132	0.694665	0.427122	1.165111
0.14511	0.3931864	-0.12137	-0.403572	0.2677549	1.084602	1.055589
-0.055422	0.7665788	0.22094	-0.0223669	0.4410121	0.13603	-0.1641916
-0.093433	-0.5560642	-0.19742	0.2344602	-2.249724	-1.552013	-0.4332234
0.14107	0.1739088	-0.21488	-0.5484007	-2.619182	-0.1397947	0.1031735

0.17358	0.8756837	-0.1527	-1.323539	-2.305682	0.3575845	0.1967817
0.37949	-0.1817985	-0.033013	0.8232906	0.7997217	0.3030638	-0.1905278
-1.6744	-0.1816831	0.10782	0.8711795	0.09414491	-0.4852286	-0.0182751
0.016002	1.398873	0.035228	-0.6603697	-0.0302451	-1.791654	0.2803093
0.074272	0.2428533	0.038853	-0.326093	-0.4864075	1.132989	0.6951449
-0.009978	-0.1119657	0.21158	0.04014495	-0.2831029	0.8065385	0.3173268
-0.82314	0.8224192	0.27442	0.0882205	0.4690728	0.4306211	0.1343337
0.24972	0.0635328	-0.024179	1.234163	0.7616545	0.4082425	0.4079669
-0.3089	0.07177026	0.007553	-0.2433056	0.2286379	1.054389	-1.058212
-0.30968	-0.1499577	0.11852	-0.1355462	0.3228703	0.7290057	0.6581746
0.98817	-0.2981235	0.08542	-0.82765	-1.652107	-0.2688219	1.269339
-0.76488	0.4679501	0.41544	-0.1071142	-0.282666	1.445051	0.05266325
0.47057	-0.1564122	0.067525	-0.5839348	-0.0967865	-0.3585209	0.4103401
0.17591	0.02223176	-0.26543	0.2279435	0.3827421	-0.200459	-0.3181156
0.47127	0.211358	-0.17965	-0.6800608	-1.700738	0.3860335	-0.266296
0.16287	0.5745216	0.099922	-0.2451695	-0.109674	-0.0231521	-0.0921879
-0.046679	-0.0454125	-0.24128	0.1683598	0.6671059	0.07822366	-0.3303398
-0.77753	-0.0838169	0.52574	-0.6144388	-0.8961571	0.3051122	0.8010544
-0.10776	-0.172847	0.23659	0.1645226	-0.903062	0.6686339	-0.0386955
0.52551	-0.0690119	0.1667	-0.8453277	0.2539277	-0.2654806	-0.6375958
0.081055	-0.2413624	0.52238	0.04293045	-0.5393472	0.6338766	0.3489045
-0.39716	0.01103093	-0.077813	0.484994	1.458993	0.5195907	0.5162364
0.111	-0.261086	0.092808	0.6343528	-0.0426503	0.2457037	0.2874443
0.33717	0.9525403	0.10069	-1.035	-2.735725	0.8712221	1.037782
0.4056	-0.0577413	0.079312	-0.1879337	-0.6326616	-0.0958869	-0.4170106
0.058616	0.5102494	0.40604	-0.8305451	-3.577038	0.09395374	-0.3228847
-0.68895	0.7577552	0.24534	-1.393798	-2.633824	1.904739	0.3608805
0.14138	NaN	0.10564	-0.5548621	-3.285887	2.314837	0.5136773
0.15769	-0.4299466	0.59874	4.992754	0.8465438	0.2513468	0.854498
-0.016491	0.00892624	-0.052472	-0.6383889	-3.318169	-0.2561214	0.4297323
-0.6268	NaN	-0.0085756	0.254493	-4.1352	0.1962586	0.1197069
0.10624	-0.1878629	-0.42671	0.1963929	0.7262414	-0.0678858	-0.1217394
-0.26309	0.6188404	-0.24982	0.866164	1.233152	0.06433794	-0.6011401
0.080436	0.2202962	-0.032703	-0.7220328	0.1851836	-0.0916653	0.2766295
0.11293	0.04038179	0.070981	-0.2140017	-0.6714387	1.93139	1.785581
0.076964	-0.0439208	0.13668	-2.022552	1.126408	-0.1316979	-0.186723
0.21194	0.4690114	-0.057161	0.02304258	0.1030518	0.06689135	-0.1036346
-0.12939	-0.0559578	0.18863	0.5324218	1.503446	-0.1850945	-0.2315578
-0.17916	-0.2266895	-0.03519	1.133275	0.5078545	0.6509795	0.8113185
0.34696	0.1066433	-0.070395	-0.1071142	-0.282666	1.356029	0.2948957
-0.16722	0.5385062	-0.19107	-0.4249434	0.2873273	0.4168968	0.6309181
0.43112	-0.105393	0.18562	0.4627702	1.021921	-0.0031167	1.179372

-0.047991	-0.165039	0.056608	0.6988403	0.8369599	1.305538	0.4054081
-0.044205	0.7983336	0.12195	-1.237514	-2.192813	-0.1324987	0.4342265
0.16537	0.1636322	0.17783	0.2347489	-0.0734142	0.3504311	-0.9941921
-0.57976	-0.1209363	-0.098532	0.08313658	0.6241601	-0.0830883	-0.2765684
-0.21352	0.4193996	-0.098007	-0.4239826	0.3464056	0.00315346	0.6520692
-0.58259	-0.3051265	0.25175	0.5152733	0.2873589	0.9264732	0.107708
-0.034693	-0.0690953	-0.14382	0.166084	1.180941	0.3564982	0.5285714
0.04685	0.0879305	-0.021484	-0.1562232	0.7533511	-0.335244	-0.0134113
-0.50177	-0.1272638	0.07493	-0.2281037	-2.782106	1.569169	0.2465087
-0.18048	-0.2028345	-0.090232	0.5855654	0.1626452	0.394489	0.1399857
0.32995	0.03378948	-0.24332	-0.2050851	0.2022934	-0.2728729	0.0558777
-0.19715	-0.174673	0.28851	0.2761784	1.541327	2.473479	1.106889
0.48852	-0.0966538	0.043302	-0.1071142	-0.282666	1.066373	0.3640392
0.21275	0.4072417	0.14261	-1.871925	-0.5139301	-0.7451017	-1.099904
-0.57637	-0.1085641	0.34002	0.7519404	0.3317824	-0.5041044	-0.59519
0.14393	-0.218444	-0.22572	-1.695449	-2.627765	0.02919788	-0.2579997
-0.38148	-0.1004552	-0.2774	1.275145	1.092117	0.2990357	0.2565654
-2.786	-0.0886535	0.19875	-0.7115201	-1.246275	0.6156589	0.1350628
0.12458	-0.1650699	-0.21108	0.3845059	-0.282666	-0.0264257	-0.3207782
-0.24303	0.2633753	0.001888	-1.157057	-3.75279	0.4561651	-0.048867

test_GSE920	test_GSE920	test_GSE110	test_GSE110	test_GSE110	test_GSE110	test_GSE134
ref_GSE9203	ref_GSE9203	ref_GSE1105	ref_GSE1105	ref_GSE1105	ref_GSE1105	ref_GSE1344
GSE9203	GSE9203	GSE11052	GSE11052	GSE11052	GSE11052	GSE13444
GEO	GEO	GEO	GEO	GEO	GEO	GEO
affymetrix	affymetrix	cDNA	cDNA	cDNA	cDNA	cDNA
lsrK_b1511:-	lsrK_b1511:-	MOPS:-1,MA	MOPS:-1,MA	MOPS:-1,MA	MOPS:-1,MA	ISOBUTANOL
962	963	969	971	972	976	1050
-0.1057531	0.8991168	-0.75901	-1.154	-0.62551	-1.075	0.049195
0.346561	1.967026	-0.017609	-0.16687	-0.10929	-0.018661	-0.10922
-0.3868925	0.7642792	0.23206	0.20848	0.17277	-0.11964	0.48039
-0.3687091	-0.4504738	0.32049	0.73964	0.1009	0.70923	1.0168
-0.8655148	-0.6039358	2.6658	1.5853	1.1699	0.62823	-0.11694
0.1426103	0.6216094	-0.011936	-0.44069	0.30533	-0.070247	-0.36006
0.731677	0.3479339	2.3748	2.0944	2.2677	3.7649	0.27038
0.1403045	1.166655	-0.92447	-0.64703	-0.41878	-0.75091	0.43447
-0.2640708	0.01864837	-0.75096	-0.8476	-0.26933	-0.13599	-0.058282
0.02077178	-0.2212929	0.86239	1.4323	0.8731	-0.65207	1.0154
0.01160551	1.216148	0.79301	0.89433	0.45389	1.5064	0.31565
-0.1754141	-0.9672698	0.93688	1.2378	0.55446	0.61502	-1.2616
-0.2085525	-0.2023072	0.20932	1.4764	0.34948	0.11438	0.32836
-1.237617	-0.5845251	-0.89883	-0.69626	-1.1204	-0.35256	-0.8339
-1.126168	0.050209	1.6916	0.67718	0.9046	0.0086135	0.56092
-0.6236085	-0.2051161	-0.46662	-0.37539	0.3142	-0.58656	0.30864
-0.5954939	-0.7297814	2.6978	3.0376	2.4671	0.37215	-0.22029
-1.146643	-0.0457949	-0.42222	-0.70847	-0.037446	-0.20377	0.13491
0.07722026	0.9794549	-0.49896	-0.63997	-0.093515	-0.57105	0.86235
-0.4949272	0.4785112	-0.56284	-0.35955	-0.77635	-0.65172	0.44726
-0.4097077	-0.3649755	0.29768	0.37674	0.29969	0.288	0.30006
-0.6086174	0.5653101	-0.62751	-0.53597	0.028732	0.090999	-0.28371
-0.8617812	-1.32099	0.71894	0.80333	0.22574	-0.39328	-0.28728
-0.4186302	-0.1266524	0.26674	0.42228	0.30695	-0.12432	0.30932
0.1981666	0.394417	-0.15442	-0.14619	-0.36086	0.8675	2.8124
-0.3402768	0.2625784	0.74773	1.0761	0.75107	1.0775	0.72424
0.3036948	-0.1312496	-0.18272	0.034937	-0.45986	-0.056865	1.1437
-0.2219083	0.3032709	0.37512	-0.45547	0.33864	0.38276	-0.049495
0.03172708	0.5173041	-0.40145	-0.70779	-0.59055	0.35994	0.24308
1.4353	0.414042	2.4711	2.6109	1.414	0.87184	2.0283
1.433323	1.504381	0.28316	0.89399	0.6492	0.2101	1.6863
-0.0515261	-0.0501128	0.62453	1.1029	0.81793	0.96564	1.0563

0.5368597	0.2156294	1.8618	2.5397	1.2449	0.52666	0.57963
-0.3330843	0.01589034	-0.084419	-0.093787	0.64551	-0.013837	0.094528
0.3692381	-0.0703023	0.28172	0.028646	0.60023	0.2834	0.12081
0.6389991	-0.1088844	-1.6545	-1.0481	-1.4305	-0.27982	-1.0673
-0.4924636	-0.2230697	-0.33345	-0.74968	-0.13147	-0.16526	-0.17436
-0.1514578	-0.0094711	0.21895	0.058577	-0.0050909	-0.34916	-0.44455
0.4437083	0.3595244	-1.3007	-0.94416	-1.3499	0.22106	-0.083198
-0.0007348	0.107914	-0.24492	-0.13451	-0.28371	-0.10256	0.44328
-0.5884401	0.1405451	-0.36107	-0.81334	0.4323	0.25976	0.31946
0.5429576	0.4831533	0.27779	0.75011	-0.0050421	0.0035308	-1.8459
-0.0298679	-0.2681437	-0.5972	-0.54939	-0.13526	0.011976	0.72797
0.881761	-0.5257332	-0.52677	-1.4972	-0.38404	-0.056865	0.36015
0.4768025	0.00266066	-0.0272	0.30989	0.049921	-0.017794	-0.5689
0.3201126	0.2732338	-0.075639	0.23846	-0.2093	0.08412	0.28995
0.5425661	-0.0662388	0.61945	0.87143	0.52345	-0.34595	0.19472
1.087698	0.4786503	-1.1114	-0.85456	-0.72823	0.11982	-0.44361
0.3335519	0.4855454	0.0044168	0.26254	0.13344	-0.011098	-0.060644
-0.1841683	-0.2499415	-1.9411	-1.7279	-1.7666	0.088434	0.55748
-0.0109544	0.315151	0.49214	0.24329	0.42352	-0.16498	-0.035569
0.2908445	-0.3177579	0.78803	-0.10027	0.014576	-0.041562	-0.71273
1.413782	0.2112357	0.51821	0.70234	0.1265	0.093958	1.3196
0.4853065	0.2987574	-0.10497	0.020693	-0.177	0.97916	0.43223
-0.3192679	0.4424222	0.48299	0.25501	0.38837	0.28852	-0.58146
-0.6886575	0.04710335	-0.23548	0.28319	-0.091277	-0.052789	0.34502
0.7273178	1.470645	0.078308	-0.013464	0.037151	-0.1508	0.85292
0.00703885	0.2483632	0.47826	0.12063	0.66879	-0.056865	1.3886
-0.6813536	-0.3867963	-0.79552	-1.1949	-0.6719	0.24332	0.062785
-0.175013	-0.1458796	-0.56169	-0.35924	-0.95824	-0.38722	-0.28292
0.1985706	-0.756976	0.029224	0.57706	0.15152	-0.058614	0.67501
0.2887455	0.5946214	0.35831	0.19658	0.27393	0.16193	-2.3922
1.456999	3.391807	0.10078	0.86837	0.6689	-0.21814	-0.3638
1.022714	0.8783785	0.37732	0.75803	-0.055488	1.1569	0.083736
1.742517	1.088736	-0.061872	0.11853	-0.15235	0.20217	-0.090009
0.9471644	0.102693	0.8286	0.25055	0.4677	0.067737	0.66029
-0.5013994	-0.1949893	-0.0487	-0.01804	-0.049599	-0.97744	-0.5166
0.4617452	0.5069064	1.0964	1.8589	1.0671	1.6277	0.81984
0.1893013	0.1714815	1.5673	1.6323	1.346	0.46758	0.9797
0.3507984	0.3279882	-0.069558	-0.15903	-0.6431	0.4548	-0.50234
0.00085181	0.1135044	-0.034018	-0.20979	-0.32083	0.047641	0.7391
0.2945938	0.4729728	2.3393	1.8912	3.1384	0.51545	0.15305
0.1510161	0.16681	0.88373	0.97418	1.0957	0.05548	0.099478
-0.2947748	1.278428	-0.14747	0.44279	0.35473	0.40632	0.36615

0.4577246	0.02552211	0.30776	0.22653	1.0622	-0.056865	0.85471
0.7841782	0.05130361	-0.6962	-0.72768	-0.62556	0.031565	0.625
0.5584156	-0.0440033	1.0935	0.70177	1.1527	0.085194	0.14118
-0.6576856	0.3298791	2.3913	2.7564	1.7714	-0.44514	-0.29031
0.09519143	-0.2999887	1.3206	1.8463	1.4977	0.55067	1.2621
-0.8709338	0.06699422	2.7704	2.5455	2.2541	0.84467	1.1655
-1.246174	0.2526707	-0.66895	-0.69696	-0.29452	-0.027915	-0.025238
0.6002483	0.3613398	-0.60231	-0.49717	-0.39097	-0.57781	0.66073
0.2768029	0.03824992	0.16171	0.075325	0.09685	-0.3301	-0.73553
0.3367192	-0.113108	-1.6229	-1.8537	-1.4687	-0.12451	0.62787
-0.0272841	0.429269	-0.80054	-0.90766	-0.52882	-0.32327	0.19055
0.2716163	0.367722	0.39	0.56048	0.10548	0.58829	0.4347
-0.7229391	0.08711414	0.78965	1.2411	0.283	0.55686	0.12284
-0.0754359	1.324705	0.84416	0.44984	0.57429	0.32379	0.5074
-0.6506447	-0.1215281	-0.42983	0.20257	-0.38387	-1.1316	-0.80909
0.00012285	0.2721561	4.57E-05	0.53141	-0.2527	-0.78784	-0.85956
0.2835288	0.1998855	0.17916	-0.046712	0.059832	0.10632	0.51859
-0.7144493	-0.4839509	-0.90393	-0.65891	0.0070318	-0.37854	-0.57451
0.5042288	0.3385459	0.2377	0.3325	0.24307	0.14516	0.3865
0.5014397	-0.4352871	0.1593	0.035841	0.016028	0.43155	0.89266
0.8068527	0.5486104	0.06221	-0.056543	-0.21536	-0.031307	-0.47457
0.1180951	0.9513106	0.20656	0.24148	-0.07235	-0.21942	0.64331
0.05339802	0.4283652	0.98403	0.44602	0.25879	0.05616	0.52578
-0.8365895	-1.105874	2.248	-0.94661	2.437	-0.0003069	0.13376
-0.0526008	-0.3452167	-0.34454	-0.24107	-0.29507	-0.30411	0.2819
-0.3332403	0.4390022	0.46446	1.0748	-0.056676	0.53752	0.96001
0.4636768	1.2068	2.052	1.3851	0.47968	0.83343	1.7547
1.014634	0.7341933	0.33043	-1.0067	-0.15747	0.10932	0.30647
1.162239	1.787884	0.073568	0.27376	0.18443	-0.27289	0.5865
1.722241	1.362573	-0.36466	-0.21808	-0.47404	-0.036491	1.0892
2.259109	1.928189	-0.19282	-0.13035	-0.50827	-0.024016	-2.2837
0.8137855	0.5478706	-0.57264	-0.14671	-0.31802	-0.16623	0.1145
-0.1601088	0.3089951	0.13693	0.40915	0.38047	0.18431	0.473
-1.149969	-0.3118616	0.31876	0.64399	0.20983	0.43182	-0.086151
1.801844	1.302231	0.87758	0.78432	0.84318	0.15135	0.34405
-0.2226079	0.2579001	-0.63392	-0.76196	-0.49948	-0.23209	1.6994
0.2912745	0.04104276	0.11187	0.58602	0.070007	0.80954	0.30277
-0.2116928	0.3072981	-0.45116	-0.02161	-0.29882	0.077162	-0.74345
1.311813	1.17099	0.4796	0.25249	0.51724	0.34017	0.43702
1.064411	0.3104516	-0.14086	-0.16487	-0.010901	-0.20437	0.36583
1.763224	1.248848	-0.67121	-0.38725	-0.6867	-1.7059	0.25717
0.4350957	0.1019483	0.98194	0.84914	1.4262	0.695	0.37766

0.1956897	-0.8397741	0.80036	0.90194	0.51722	0.68506	0.065067
-0.0606234	-0.4508117	-0.9357	-0.37322	-0.063723	0.93539	-1.0705
0.2870773	-0.0432964	0.33953	0.87516	0.031468	0.2809	0.24631
-0.3055019	0.6832328	0.93288	0.40959	0.50552	-0.13582	-0.022669
-0.1835246	0.5506385	0.46929	0.85157	0.33972	-0.55741	0.16299
0.1836597	-0.5227485	1.683	1.1377	1.9547	-0.056865	-0.14169
1.100474	0.6708046	0.3409	0.3598	0.40482	0.60254	-0.23436
1.217957	0.6963881	0.45938	0.72656	0.29561	0.67582	0.068282
0.7376862	0.7937356	0.94716	0.9162	0.86334	-0.38703	0.43865
0.3870321	0.5027388	0.076697	-0.13273	0.2671	-0.082603	-0.94879
0.0796963	-0.4583318	-0.14125	-0.35551	-0.23171	-0.056865	-0.35274
1.015656	0.3005475	-0.039026	0.053175	-0.11576	0.010099	0.72449
0.01976701	0.1878848	-0.47349	-1.2966	-0.84098	-0.43459	0.31903
-0.4345343	-0.0272161	-0.96669	-0.99781	-0.58873	-0.24596	-0.50728
-0.1641499	-0.1494151	-2.9488	-2.9656	-1.099	-0.81799	-0.87299
0.3624671	0.1166456	-1.4422	-1.469	-0.58625	-0.34426	0.62531
0.7129294	0.5716709	-0.3001	-0.27114	-0.32343	-0.13372	0.41837
0.2476356	-0.185349	4.82	4.2064	3.7313	0.8864	-0.96004
0.9713437	0.4945222	-0.38741	-0.49842	-0.30507	0.39584	1.1799
0.1175495	0.5221708	3.3951	3.3067	2.578	3.4046	-1.1144
-0.7349775	1.144797	5.4264	5.9129	3.7299	5.4567	-0.13782
0.6500548	0.0778526	0.1854	0.084604	-0.003019	-0.10678	0.070904
NaN	NaN	NaN	NaN	NaN	NaN	NaN
-0.1405123	0.3155081	0.10995	-0.052623	0.24419	0.01119	1.2347
0.8719165	0.2688512	-0.079284	0.44894	-0.13403	-0.11232	0.6516
0.4623439	0.1166202	0.37554	0.50044	0.17789	0.075813	1.6983
-0.1745366	0.9931682	0.074761	0.12028	-0.19096	0.36395	0.0025494
1.223316	0.3005613	0.18264	0.4706	0.0060506	0.74654	0.71244
1.012333	0.5827394	1.17	1.1261	1.6948	-0.16072	0.60737
0.1803488	-0.0066257	2.019	2.2639	1.2642	1.4447	0.16819
0.04331146	1.81728	-0.062908	-0.65767	-0.23523	-1.0049	0.87923
0.6054911	0.0916741	0.69941	0.98487	0.17713	0.50794	0.38233
-0.5787875	-0.1986628	-0.58432	-1.298	-0.69615	-0.56002	-0.3361
0.06159571	0.3863065	-0.48345	-0.14729	0.0020737	-0.6855	-0.63359
0.2996671	0.1817855	0.34489	0.17468	0.41391	-0.21378	0.68954
0.2060828	0.6787933	0.47555	0.64439	-0.27818	0.13713	0.18519
0.7833294	0.2223197	1.6439	1.3312	1.3784	0.59879	0.94932
0.1281509	0.6214434	0.40741	-0.011774	0.3587	-0.0002259	-0.44554
0.1972346	-0.1849227	2.0286	2.3698	1.7881	3.4794	2.8447
0.762781	0.1532437	0.19232	0.19387	0.11079	-0.11174	0.91563
-0.6955568	1.318674	1.0576	1.584	0.59262	0.037231	0.012288
1.393539	0.6846608	2.608	1.7596	2.2377	0.21886	-0.1538

0.2228575	-0.1499945	0.41039	0.54197	0.13614	0.20998	0.30388
0.3696867	0.2490291	-1.9653	-1.7065	-1.6905	0.21618	-0.021346
0.3300169	0.06417443	0.36786	0.19578	0.47261	-0.009096	0.14163
0.03604784	0.5183196	-0.071125	0.14869	-0.23684	0.10619	0.027159
-0.6548112	-1.0298	0.26099	0.67729	0.3057	-0.34354	0.84502
0.3169448	0.2073573	-0.798	-0.61479	-0.61834	-0.15317	0.048544
0.6614507	-0.0140717	-0.38598	-0.13419	-0.10105	-0.1175	0.47212
-0.2169958	0.1358334	0.98073	1.4364	0.88203	0.19321	0.21818
-1.487426	-1.240481	-0.10622	-0.17859	-0.1603	-0.43874	0.16022
0.1287591	-0.4034489	0.57345	0.11705	0.64523	0.062563	-0.37603
-0.3188709	0.5931601	-0.3515	0.35469	0.79876	-0.31448	0.1336
-0.0751921	-0.0897513	1.352	1.8285	1.2407	1.2836	0.018657
0.1080104	0.00034906	0.65966	0.49885	0.33151	0.3319	0.71923
-0.0798154	0.05559811	-0.27138	-0.059826	-0.44635	-0.17281	-0.22198
-0.2909438	-0.7189107	-0.10481	0.14924	-0.15019	0.066108	-0.26525
-0.0280426	-0.7699847	-0.46062	-0.46896	-1.1636	0.89357	-0.20876
-0.3315178	-0.6224574	1.1783	1.3522	1.0229	1.1363	-0.083245
0.3104555	0.01939079	-0.42056	-0.33009	-0.95753	1.3571	0.36894
0.4921514	-0.5940715	-0.003685	-0.17097	-0.091903	1.226	-0.31394
0.2433071	0.5039101	0.38489	0.19456	0.53765	0.012045	0.23463
0.04025291	1.143554	-1.0334	-0.99861	-0.24884	-0.42236	1.2541
-0.3380436	0.02343323	-0.096963	-0.020517	-0.23191	0.44368	-0.10202
0.1634764	0.5340938	-0.12904	-0.26612	-0.26649	0.31771	-0.087436
-0.5261764	-0.1249143	-0.4131	-0.31523	-0.51848	1.0424	0.10591
-0.9482746	-0.9356913	-0.029968	-0.036289	-0.15059	-0.86802	0.30121
0.6522524	0.6368699	0.18264	-0.41937	0.090052	-0.87092	-0.11925
0.6104646	0.5451332	0.74162	0.87358	0.81603	1.607	0.41902
-0.201155	0.8248095	-1.3384	-1.0546	-1.3679	-0.36658	0.50168
0.4081475	-0.481649	1.0818	1.0501	0.88013	2.3493	0.020881
0.9171197	0.7117924	0.45897	0.11823	0.48881	-0.41663	0.39135
0.1510107	-0.0107115	1.364	1.332	1.2264	0.33992	0.12847
0.6252494	0.2011427	0.39698	0.30023	0.44772	0.86272	-0.042866
-0.3643036	-0.3694182	0.47152	0.079919	0.080596	0.30636	0.2055
0.2885848	-0.553151	-0.30232	-0.16382	-0.15788	-0.38668	0.86441
0.2467071	0.784297	-0.37887	-0.61651	-0.31565	-0.57244	0.33971
-0.6136637	-0.2316681	0.11448	0.22544	0.48953	0.50558	-0.65091
0.358805	0.2750398	0.2302	0.22441	0.1451	-0.019229	0.43225
0.2043964	0.500146	0.41573	0.38549	0.35469	0.60877	0.87003
-1.06684	0.8910509	-0.048032	-0.65807	-0.16691	-0.0063217	-0.18519
-0.2209461	-0.3746124	0.3835	0.65884	0.25976	0.37076	0.68931
0.6467388	0.2378974	1.4076	1.47	1.3989	0.40826	0.44768
-0.0036389	-0.4386735	-0.21862	-0.071247	-0.28965	-0.16834	0.9973

-0.2977789	-0.2422963	0.8205	1.4478	0.44315	0.59746	0.12417
0.3606729	0.05140514	0.37081	0.86917	0.11552	0.018426	-0.043206
-0.0463526	-0.1780686	2.5476	3.0423	1.9156	2.6753	0.095876
0.01646055	0.6498395	-0.90049	-0.50321	-0.8937	0.024545	0.114
0.7637062	-0.0095984	0.28916	0.6099	0.61207	0.087846	0.44472
1.273237	1.579977	1.1256	1.3203	0.62821	0.87568	0.99579
-0.2072483	-0.2676194	-0.47497	0.40534	-0.29039	-0.08098	0.72333
1.088134	1.125041	0.031103	-0.11079	-0.45364	-0.095772	-0.30555
-0.1714284	-0.5668968	0.034098	-0.080658	-0.37755	0.51254	-0.91528
0.5535216	0.1298483	0.092366	-0.014695	-0.029916	0.45733	0.6654
0.4283986	0.5062496	0.75515	0.85031	0.22152	0.3883	-0.70575
0.2598909	0.03357388	0.074493	0.084557	0.16533	1.4298	0.92218
-0.1490823	0.01689998	0.1172	0.70955	-0.52363	0.38426	-0.0047311
-0.1529605	0.2717434	0.87654	1.531	0.58427	1.1262	0.13339
0.737623	-1.292178	-2.012	-2.1853	-0.71063	-0.2732	-1.5764
0.5040726	0.4512472	0.74585	1.0141	0.72852	-0.059542	-0.27357
0.5071858	0.1379484	0.029057	0.57838	-0.78924	0.25825	2.0513
0.5785184	0.5003383	0.029728	0.062248	0.34762	0.014195	0.74781
0.6585444	-0.0109471	-0.28461	0.67056	-0.46497	-0.17258	0.64143
-0.0198021	-0.0061732	0.81788	0.15695	0.32516	0.14188	0.89668
0.1017228	1.111778	-1.8997	-2.1245	-1.8991	-0.99255	1.4036
0.1851138	-0.8846145	-0.13511	0.070251	0.095814	-0.18832	-0.45684
-0.5891633	-0.3522325	-0.54671	-0.69655	-0.2919	0.19695	-0.92776
0.1940016	0.0314943	0.13783	-0.16935	0.31565	-0.23128	2.1667
0.5848795	0.2947584	-1.677	-0.94445	0.078883	-0.52995	-0.64141
-0.8700525	0.00468693	-0.81234	-0.46144	-0.92026	0.28632	0.28685
0.763772	0.7668546	0.5736	0.61763	0.44393	-1.1722	-0.768
0.9379478	0.8824646	-0.40212	0.082678	-0.23399	0.94037	0.19386
0.2466619	0.2823988	1.0359	1.737	0.91806	-0.26649	0.30327
-0.1006322	-0.8441976	0.79685	1.5775	0.69785	-0.14445	0.22631
-0.2003986	-0.2359265	0.3551	0.34599	0.14675	0.068838	0.019301
0.1942624	-0.3188703	1.0833	1.1575	0.68777	0.32413	0.73536
0.5671353	0.3649955	-0.21137	0.12736	-0.28181	0.64109	0.50713
1.327524	1.063763	-1.2214	-0.71333	-0.75829	-0.56744	-0.019571
-0.092618	0.2662605	-0.3803	-0.21553	0.11673	0.00067913	0.23815
0.03144702	-0.4652637	0.19261	0.17861	-0.17048	-0.12833	-0.12779
0.4594019	0.2795058	-0.71077	-0.6115	-0.93025	-0.48343	-0.010137
0.9969523	1.094142	-0.3426	-0.78941	-0.35073	-0.15537	0.11797
-0.2455002	1.510038	-0.044916	0.19306	-0.22646	-0.50466	-0.47845
0.2519686	-0.1341917	-0.37897	-0.99696	-0.70901	-0.042565	-0.047677
0.2733805	-0.7950161	-0.67147	-1.4782	-1.11	-0.21023	-1.2009
0.01997327	0.02926881	-0.38892	-0.12596	-0.66872	0.0068537	0.57085

-0.1350932	0.4451275	-0.90848	-1.5957	-1.0469	-0.36944	0.12866
0.8531216	0.9135302	-0.099901	-0.10437	0.027838	-0.24794	1.6453
-0.1956801	-0.1835089	-1.648	-1.5692	-1.4812	-0.58703	0.0019442
-0.0699567	-0.2941775	-0.068376	-0.72118	-0.35995	-0.073359	0.36119
0.4317074	0.6020113	2.0056	2.2813	1.7457	0.46964	-0.65683
-0.2559847	0.06861951	-0.51443	-0.51887	-0.44919	-0.58514	0.15018
1.062201	0.02975211	1.0586	1.7178	1.1033	0.51924	0.802
0.248149	0.4338245	2.1448	2.3796	1.7058	0.83322	0.60813
0.3452569	-0.1365255	0.50732	0.81655	0.20399	0.0099446	0.12467
0.9480224	0.4210601	-0.88664	-1.0028	-0.70153	0.22655	0.46815
0.09025416	1.479599	0.14258	0.59091	0.38473	0.62627	0.077385
0.1171157	0.4068121	-1.0198	-1.3987	-1.0009	0.29234	-0.1939
-0.8472797	0.8239303	-0.50045	-0.31853	-0.53911	-0.1209	0.15565
-0.36364	0.2350506	-0.042485	0.49498	-0.063711	-0.059763	0.58884
-0.369508	-0.0352564	0.36013	0.53772	0.28803	-0.37573	-0.0036064
0.7204077	0.5664565	-1.6304	-1.0615	-1.0182	-0.17377	-0.56206
-0.2314454	-0.1896849	0.62105	0.48215	0.47111	0.85146	0.48588
0.07304792	0.7104621	-0.023395	0.27295	-0.21867	-0.57647	1.6673
0.8075717	-0.1501369	3.2119	2.7522	3.057	0.34623	-0.82013
-0.2781676	0.07329019	0.58678	1.1443	0.95496	0.24419	0.013585
0.9949379	-0.0226308	1.5461	2.2753	1.6271	0.78891	0.36005
0.492196	0.5921587	0.050496	-0.29773	0.33178	0.14629	0.27456
0.4467287	0.1655244	-0.43345	-0.38267	-0.31668	-0.11611	-0.48637
0.3960473	1.084928	0.17163	0.31846	0.22984	0.13822	1.038
-0.3524236	-0.5893568	0.87009	0.97034	-0.46707	0.27217	0.63759
0.6021285	0.01152874	0.21323	0.43695	0.22966	0.18787	-0.35182
0.5880319	0.6829029	0.09157	0.4812	0.11697	0.63	0.025992
0.958312	1.121359	1.2312	1.2959	0.74313	1.2795	0.99576
0.3089234	0.251779	3.0634	3.8414	2.4394	2.0526	1.1819
-0.2319037	1.048394	-1.0015	-0.93104	-0.96914	0.17113	0.21469
-0.5814774	1.179359	0.48129	0.76106	0.29046	-0.56919	0.79073
-0.2083873	0.117585	0.67252	0.5772	0.60679	0.067924	-0.13839
0.01757883	-0.0173714	0.85825	0.82688	1.1009	0.10552	0.37136
0.6876669	0.6103078	2.6278	2.9819	3.0388	0.64881	1.1527
1.384469	1.553355	0.036182	-0.29505	0.24966	-0.71257	2.7023
0.3700251	-0.0506722	0.27692	0.59911	0.0060284	0.48891	0.6613
-0.0339981	0.3035281	0.27767	-0.32757	-0.049823	-0.32878	-0.24791
-0.3176071	-0.5182807	-0.12715	0.18044	-0.18889	-0.13699	0.61217
0.6711311	0.5217089	0.48936	0.68241	0.35529	-0.25544	-0.14236
0.290334	-0.105614	-0.24966	0.019544	-0.48802	-0.15981	1.6121
-0.2427421	0.1091243	-0.95326	-0.478	-1.2214	0.59997	2.1016
0.9127499	-2.088338	2.8542	2.3483	2.1766	0.5477	0.24164

0.3906005	0.9707583	0.78624	0.73458	0.59575	0.84016	0.30895
0.3801407	0.7398744	0.51591	0.75107	0.35822	0.5691	0.34137
0.7544771	0.5260033	-0.48658	-0.28581	-0.54354	-0.25443	-0.14753
-0.1811467	0.357877	0.2037	1.0945	-0.34826	0.31788	-0.21646
0.5033201	0.2533994	-0.25078	-0.34881	-0.39918	0.012874	0.43572
0.8039602	0.2411628	0.33377	0.21506	0.15617	0.23865	0.13179
0.1878302	1.07482	0.54043	1.1755	0.509	-0.58674	-0.14032
0.5695176	-0.3094224	0.9253	1.0014	1.1721	0.059609	-0.49632
0.8002851	0.1794237	0.55595	0.37687	0.91273	-0.12315	0.11209
-0.3171047	0.08656999	-0.11103	-0.080154	0.4698	0.073839	-0.49134
-0.0546038	-0.2038089	0.034418	0.40039	0.35262	-0.27954	-0.65942
0.1266305	1.248545	1.4447	1.3373	1.7646	0.21151	-0.83225
0.3183546	-0.0354995	1.012	1.4265	1.3375	0.54445	-0.48637
0.4398222	-1.082582	1.1842	0.64841	1.3529	0.54414	0.35266
0.8028943	0.2316954	0.33414	0.64894	1.0077	-0.083626	0.77231
-0.1026165	-0.0564867	-0.28298	-0.30008	-0.14775	0.35463	-1.0153
0.3317064	-0.3714259	0.7516	0.82256	0.51419	-0.84916	0.46929
-0.0039526	0.3662236	1.0417	1.6017	0.7005	-0.40991	0.72411
-0.6002786	-0.1219457	0.20897	0.39094	0.13163	0.076639	0.63847
0.3575512	-0.677859	1.3021	0.072693	1.2088	-0.26665	0.18961

test_GSE134	test_GSE134	test_GSE134	test_GSE134	test_GSE134	test_GSE134	test_GSE134
ref_GSE1344	ref_GSE1344	ref_GSE1344	ref_GSE1344	ref_GSE1344	ref_GSE1344	ref_GSE1344
GSE13444	GSE13444	GSE13444	GSE13444	GSE13444	GSE13444	GSE13444
GEO	GEO	GEO	GEO	GEO	GEO	GEO
cDNA	cDNA	cDNA	cDNA	cDNA	cDNA	cDNA
ISOBUTANOL	ISOBUTANOL	ISOBUTANOL	ISOBUTANOL	ISOBUTANOL	ISOBUTANOL	ISOBUTANOL
1056	1057	1083	1092	1097	1099	1100
-0.38725	-0.43999	0.8887	-0.086042	-0.15362	-1.1313	-0.50808
-0.69326	-0.76894	-0.50964	-0.27339	0.06525	0.36838	0.26383
0.045112	0.45606	0.38878	-0.24263	0.38772	0.2188	-0.022181
1.4725	0.34744	0.86687	1.4967	1.0263	-0.29011	0.45592
0.76488	0.71467	0.42747	-0.39298	0.072278	0.18661	-0.18998
-0.2845	-0.62923	0.027117	-0.97481	-0.78831	-0.54214	-0.67655
0.11221	0.22756	0.19843	1.4177	0.60606	0.18049	0.65353
0.052088	0.2051	1.0465	0.38031	0.5652	0.95229	0.52253
0.32708	0.39767	-0.17725	1.0105	0.1296	-0.22419	-0.37116
0.75914	0.82659	0.89118	2.2495	1.1082	0.22615	0.44013
1.7809	2.1276	1.3131	0.26598	1.5664	0.15888	0.75961
-0.98591	-1.1819	-0.73522	-0.70153	-1.5307	-0.93826	-0.93763
0.35654	0.23815	-0.43296	-0.22095	0.69167	1.368	1.1564
-0.7565	-0.85973	-0.4152	-0.27047	-1.075	-0.3857	-1.0064
1.1936	1.7565	0.30916	-1.3525	0.34475	-0.054012	-0.056838
0.65599	0.95125	-0.061885	-1.2523	-0.31487	-1.949	-1.266
-0.29326	-0.34961	0.39215	0.14174	-0.91553	-0.35405	-0.63083
-0.32524	-0.21685	-1.4373	-1.5208	0.88751	0.92718	-0.23514
0.1238	0.071069	0.46097	0.89536	1.2299	0.31624	0.58739
-0.0064583	0.36525	0.13747	0.5061	1.0301	0.55496	0.26056
0.03269	-0.14701	0.72769	0.63136	-0.021797	-0.10789	-0.35623
-0.7791	-0.92636	-0.47036	-1.1037	-0.56057	-0.15823	-0.3985
-1.4636	-1.1024	-0.23461	-3.1581	-0.39742	-1.1994	-0.12369
0.015342	0.23345	0.71829	-0.88335	-0.0038624	0.0029315	-0.13969
4.5297	4.1289	0.06557	4.7101	4.2826	2.1017	4.3897
0.84637	0.77	1.0297	1.1643	0.43651	0.72936	0.70634
1.086	1.0577	2.6209	2.1648	1.405	0.14209	0.81303
-0.2027	-0.30998	-0.62249	-0.97391	-0.36237	-0.33067	-0.5994
0.82841	1.0454	1.0222	1.4968	0.424	0.55782	0.28297
2.8156	2.957	1.733	2.2888	1.3747	1.1807	1.6205
1.9218	1.1302	2.0767	1.9369	1.8367	1.0354	2.7235
-0.019734	0.14118	-0.33959	0.50094	1.0408	1.3232	1.4843

0.59616	0.15536	1.2665	1.4235	0.8508	0.50868	0.070123
0.053877	0.65764	0.52251	0.2624	0.34473	0.40731	0.21416
0.32977	0.29068	-0.20798	-0.0087464	-0.14631	-0.4897	-0.2438
-0.75922	-0.9439	-1.7526	-2.4034	-0.88172	-1.7159	-2.6574
-0.058132	-0.30195	0.2738	-0.5092	-0.60245	-0.56487	-0.74517
1.1098	1.1144	0.011208	0.36934	-1.338	-1.1654	-0.73655
0.21328	-1.4869	-0.73049	0.059669	-0.15099	-1.5642	-2.7727
-0.08149	-0.60003	0.54008	0.49288	0.17995	-0.60955	0.17979
0.86041	0.87173	-0.74267	0.89504	0.38415	-0.46529	-0.17198
-0.58367	0.29874	-0.37262	0.48898	-0.61333	-1.4177	-1.0847
0.13749	0.29658	0.24819	0.96337	0.53381	0.99138	0.54402
0.65864	0.61531	-0.10525	1.3038	0.14907	0.21662	0.42517
-1.2494	-2.4832	-0.24474	-1.2243	-0.57746	-1.1793	-0.68805
0.066405	0.34571	0.23786	0.98345	0.31147	-0.055676	0.059692
-0.071089	0.08191	0.20495	0.82619	0.0075014	0.80796	0.0481
0.041092	0.01628	-0.88509	0.048238	-0.80413	0.53006	0.067307
-0.44199	0.17251	0.018428	-0.61853	0.97514	-0.84195	-0.25852
-0.0042911	0.084582	-0.079666	0.096771	0.228	0.094516	0.23111
0.33345	0.34808	0.43879	0.095425	0.60544	-0.038004	0.22857
0.76562	0.69393	0.3749	-0.56962	0.46928	-1.0367	0.12002
0.7971	1.2815	2.4533	1.3146	2.6853	1.3024	1.2709
-0.1902	-0.31564	0.84821	0.43789	0.40916	0.54835	0.30889
0.10153	0.012733	-0.44024	-0.32626	-0.71804	-0.32327	-0.33598
-0.052978	-0.62609	-0.19711	0.78944	0.0073002	0.11372	0.42021
-0.22772	0.015972	0.13268	-0.15839	0.88091	-0.15746	0.15838
1.266	1.2979	1.4902	1.621	1.3527	0.88312	1.0017
-0.053033	-0.071327	-0.12003	0.065886	-0.025585	0.70481	0.73635
0.52844	0.52652	1.1956	-0.96825	0.12965	-0.14773	0.1645
0.56822	0.6369	1.198	0.22672	0.9318	0.8732	2.0012
-1.1343	-1.0938	-3.2962	-1.452	-1.6826	-0.75448	-0.53412
-0.20346	-0.56954	-1.296	0.049306	-0.69446	0.50815	0.50089
1.0467	0.37066	0.94011	1.0462	0.78907	-0.94639	-0.73303
0.24311	0.17996	0.41576	0.77125	0.65968	-0.73829	-0.16494
0.10797	0.31516	0.6307	-0.18874	0.58286	0.28837	0.49325
0.35928	0.64961	0.29161	-0.23947	0.92182	0.66886	0.52081
0.68046	1.0003	1.0893	1.8922	0.74549	0.0045162	0.3071
1.3329	1.665	1.4692	0.55112	0.81172	0.38918	0.87451
0.22291	0.0068793	0.13418	0.27079	-0.096826	-0.082413	0.019367
0.75114	0.83361	0.59018	1.4281	1.2643	0.96316	1.1799
-0.42608	-0.41001	-0.70388	-0.25284	0.14126	0.059225	0.21339
0.56833	0.31474	0.11774	-0.70794	0.51286	0.15664	0.30184
0.089086	-0.225	1.0408	1.0866	0.13307	0.54119	0.50147

1.3856	1.7384	0.79464	0.82365	0.78281	0.91532	0.77257
0.83744	1.3789	0.80328	0.77121	0.99582	0.64654	1.0739
-0.45332	-0.37025	-0.25139	0.13049	0.38202	0.83515	0.85978
-0.57701	-0.63525	-0.81644	-2.1208	-0.81102	0.012299	-0.26752
0.81252	0.47607	0.62466	0.75747	0.64726	0.73634	0.52626
0.96478	0.95264	1.0521	0.79838	1.2112	0.38715	1.0557
-0.61155	-0.9576	-0.50004	0.33469	-0.17896	-0.3228	-0.70218
0.93172	0.89914	1.3416	0.55268	0.79893	0.614	0.62982
0.31382	0.36172	0.12504	0.47423	-0.57134	-0.67694	-0.017266
1.2361	0.95504	0.71469	1.0084	0.6155	0.61589	0.8728
0.45603	0.47703	0.2309	-0.020576	-0.31696	0.031151	0.0021812
0.51981	0.22388	0.29939	-0.0074808	0.38613	-0.40593	-0.24571
-0.79445	-0.76466	-0.32089	-0.27854	0.021155	0.24181	0.33376
0.30167	0.57844	0.22105	0.71397	-0.28725	-1.3293	0.082145
0.15486	-0.17136	-0.093103	-0.64304	-0.8784	-0.90802	-0.83097
-0.12231	-0.29938	-0.55263	-0.92118	-1.1794	-0.85714	-1.4113
1.2393	1.4681	1.1945	0.1981	0.52929	0.32763	1.1081
-0.16785	-0.44791	-0.16218	-0.49609	-0.54295	-0.055406	0.0045147
2.3095	1.9445	0.4859	0.79592	0.40476	0.11661	0.025952
0.06791	0.10252	0.093181	1.1891	0.71315	0.32662	0.41143
0.059737	-0.17347	0.74042	-0.47791	-0.19052	-1.28	-0.41365
0.94146	0.64157	1.4665	0.84667	0.41322	0.13792	0.4546
0.26109	-0.20563	1.597	-0.74407	0.49956	0.8268	0.76705
-0.54522	-0.65098	-0.3244	-0.52603	-0.14803	0.085569	0.021318
-0.60897	-0.72722	0.097794	-0.44542	-0.39796	-0.61728	-0.4884
0.81538	0.72539	0.64495	1.0192	0.93757	4.75E-05	0.37497
2.3161	1.8949	2.2351	1.9705	2.3452	0.014125	1.4131
-0.34829	0.092587	-0.80243	-0.22544	0.27245	0.42746	0.44695
2.078	1.6676	0.19749	0.10909	0.11377	0.41401	0.55397
1.547	1.3623	-0.67458	-0.3071	0.74675	1.7795	2.586
-1.6428	-1.7021	-3.5367	-2.0551	-3.2544	-0.61359	-2.2378
0.12436	0.39303	0.18518	0.58525	0.50565	0.52319	1.2714
0.65731	0.71068	0.5992	-0.1101	0.77359	1.5906	1.5703
-0.58015	-0.26565	-0.34003	-0.93517	-0.011955	0.045538	0.30664
1.3035	1.1434	0.65997	0.9362	0.57077	1.1471	1.1545
0.48029	1.3311	1.4217	1.6906	1.7087	0.58689	1.0897
-0.026071	0.027195	0.27915	0.75545	0.42699	0.43414	0.65859
-0.67129	-0.25986	-1.4035	-0.26089	-1.1548	-0.77884	-0.67781
0.24381	0.58971	0.32794	0.24586	0.67456	0.54096	0.52596
0.038861	0.23758	0.079659	0.30811	0.40105	-0.031639	0.65048
0.67605	0.78342	0.38184	0.4518	0.31608	0.020332	0.49221
0.29863	0.50227	-0.22805	-0.13698	0.44515	1.1201	1.5668

-0.40923	-0.27182	0.15259	-0.10964	-0.061053	0.22401	0.20374
-1.4747	-1.2749	-1.3959	-0.18882	-1.1754	-1.3484	-1.5446
-0.55822	-0.74359	-0.071063	-0.40754	0.40323	0.1077	0.26976
-0.25186	-1.0417	0.60205	-0.30438	-0.55168	-0.21464	-1.1345
0.20342	0.2097	0.50494	1.1002	-0.017618	0.84735	0.74957
0.47837	0.42293	1.081	0.040879	0.18076	0.39925	0.56144
-0.037524	0.078351	0.75147	0.093219	0.15774	-0.10792	-0.25693
0.26633	0.31529	0.17763	0.013539	0.42485	0.37676	0.65558
0.13022	0.019441	0.46013	1.1566	0.82419	1.0319	0.9899
-0.12083	-0.14732	-0.34361	-0.44909	-1.0071	-0.19415	-0.44546
0.10103	0.12652	-1.3166	-0.29889	-0.2881	0.52042	0.27963
0.91682	0.78846	0.68097	0.79929	0.4799	0.9543	0.65765
-0.35008	-0.41983	0.18939	1.3847	0.98614	0.35221	0.465
0.2326	0.36902	0.18994	-0.80887	-0.76644	-0.33296	-0.66913
-0.051526	-0.219	-1.3333	-0.67193	-1.0761	0.21881	-0.0022696
0.76715	0.56022	0.015096	0.37243	0.9232	2.0883	2.2378
0.031074	0.050771	-0.74903	-0.98187	0.36635	-0.17045	0.041151
-0.31889	-0.80663	-0.94568	-1.1846	-0.49335	-0.85348	-0.1794
1.9542	1.589	1.3299	1.257	1.5098	3.2256	2.7319
-0.34229	-0.18187	-0.79883	-0.59482	-0.82328	0.68977	-0.26138
-0.43447	-0.47639	-0.66687	-1.3251	-0.26031	0.25983	-0.12289
-0.51839	-0.62968	-0.16022	-0.2702	-0.21479	-0.40668	-0.3123
NaN	NaN	NaN	NaN	NaN	NaN	NaN
1.5145	1.4913	1.1253	0.56652	1.3143	2.6344	1.8558
0.55275	0.56255	0.4545	0.65418	0.40452	0.77845	0.98981
0.21609	0.12981	0.46123	1.2961	1.5678	1.1905	1.3083
0.7513	0.62324	0.43951	0.72717	0.23951	0.68119	0.28668
0.2645	0.30199	1.0572	0.97913	0.70413	0.39844	0.7543
-0.12726	0.083154	-0.28871	0.5666	-0.4018	0.261	0.14705
-0.44346	-0.64203	-0.18478	1.1012	0.42859	0.38753	0.62006
0.06737	-0.014236	0.35778	0.41098	1.0113	-0.93163	0.56973
-0.31602	-0.29843	0.79705	0.41632	0.87659	0.14174	0.47517
0.66892	0.64069	0.43681	0.2171	0.13944	-0.023814	-0.11226
-0.28992	-0.41824	-0.45632	0.54897	-0.45705	-0.67561	-0.6813
0.075939	0.41601	0.78795	1.1288	0.70706	0.40086	0.3208
0.25308	-0.16717	0.36456	0.31206	0.30749	0.38589	0.29154
0.98567	0.75729	0.77119	1.2523	0.71308	1.1953	0.99293
0.59293	0.29609	1.0211	-0.86422	1.1995	-0.51689	1.2388
-0.13879	-0.23866	0.32175	1.13	0.37498	0.9183	1.0161
1.1063	1.0312	0.57067	0.63299	0.61782	0.96917	0.6481
0.89008	0.4842	-0.20878	0.37561	-0.17063	-0.42673	0.12615
1.0831	1.8697	1.0868	0.63974	-0.62613	-0.091834	-0.27977

-0.2608	-0.14549	-0.19067	-0.09541	0.14789	0.26632	0.37826
-0.017254	-0.11142	-0.4121	-0.16913	-0.1803	-0.37504	-0.16656
-0.60356	-1.312	-0.13703	-1.1912	-0.32754	-0.64815	-0.90624
-0.24741	-0.32651	-1.0423	-0.57895	-0.093025	0.19595	-0.40639
0.55591	0.5894	0.72641	0.35707	0.70462	0.31795	0.42763
0.3384	0.42223	0.071145	0.26533	-0.20927	0.53983	0.2725
0.57611	0.44236	0.52079	0.19613	0.1359	0.081055	0.027358
0.77925	0.80207	0.39684	0.34787	0.091977	0.24957	0.0082728
-0.085187	-0.042837	0.3315	-0.56594	0.07006	-0.1715	-0.16506
0.80156	0.45961	0.14704	0.7107	0.050015	0.29332	0.47394
0.38223	0.23928	-0.10135	0.093468	-0.088891	0.84971	0.08965
-0.32966	-0.39969	-0.847	0.040754	0.74057	-0.5952	-0.063111
0.32323	0.23856	0.096948	0.95757	0.40636	-0.15156	1.0123
0.25191	0.21302	-0.29747	0.11761	-0.089217	0.02805	-0.031302
-0.22479	-0.20513	-0.092705	-0.6122	0.2441	0.5956	0.67009
1.6475	1.3401	1.08	2.4023	0.51111	0.2751	0.47871
0.92956	0.87925	-0.98241	-0.23347	0.096837	-0.63767	-0.094377
-0.26879	-0.11905	-0.87051	-0.00069	0.024986	0.14267	0.26693
-0.69571	-0.80975	-1.1638	-1.0456	-0.8996	-0.65004	-0.96907
0.15007	0.075048	-0.11188	0.052158	-0.052545	0.26739	0.12273
1.1264	1.0817	0.89502	0.2662	1.6372	1.6839	1.6903
0.23719	0.10171	0.41444	0.33915	0.68681	0.75537	0.30617
-0.082229	0.053713	0.39549	0.5653	0.013478	-0.79793	0.037667
-0.32885	-1.4289	0.096256	-1.0635	-0.35421	0.16586	-0.49639
0.68773	0.87317	1.3548	0.57358	0.27785	0.42023	0.1971
-0.46972	-0.66334	-0.29456	-0.18568	0.083982	-0.079501	0.10927
0.68281	0.56781	0.077468	0.15095	0.4888	0.64057	0.87986
0.96155	1.6991	-0.36547	0.033834	0.55282	0.37724	0.5271
-0.0993	-0.19935	0.11505	0.59701	-0.46913	0.34886	0.37319
0.97124	0.98614	0.8363	0.46525	0.27721	0.26878	0.37932
0.39022	0.71159	0.72028	0.47517	0.11069	-0.015812	0.16934
-0.41795	0.02209	-0.12855	-0.76489	-0.60856	-0.53178	-1.3081
0.50648	1.7996	0.49694	1.128	-0.024191	0.29663	-1.4056
0.054278	-0.44841	-0.013324	0.74184	-0.45067	0.61257	0.65171
0.92734	0.56361	4.6935	0.61456	0.097139	0.81136	1.0023
-0.080084	-0.10358	-0.78256	-0.29677	-0.46554	-0.11363	-0.073578
0.10835	-0.18018	-0.04463	-0.42192	0.31962	-0.39611	0.35501
0.60536	0.46589	0.4967	0.59684	0.77266	0.72288	0.81482
-0.9296	-0.87192	-0.11919	-0.41112	-0.26975	-1.8775	-0.81251
0.50609	-0.30177	-0.54269	-0.63952	1.2672	0.10611	0.30228
1.0456	0.92746	0.8273	1.2642	0.97984	1.241	1.6349
1.6571	1.7669	1.0808	1.1317	1.344	-0.16675	0.871

0.15369	0.37422	0.028685	-0.30522	0.27067	0.99035	0.60548
0.071612	0.12705	0.12913	0.099442	0.19758	-0.0068472	0.4869
-0.16667	-0.40872	0.090046	-0.10474	0.49021	0.9498	0.7876
-0.79051	-0.81256	0.70733	-0.64831	0.17935	0.24769	0.21431
-0.4618	-0.80028	-0.38896	-0.72587	-0.1542	-1.7645	0.1078
0.62317	0.76378	0.8146	0.86042	1.2304	1.1403	1.8978
0.31634	0.42009	0.77391	0.53918	0.76982	0.063043	0.21589
0.30899	0.29065	0.066355	0.16419	0.001627	0.49808	-0.51335
-1.2874	-0.64216	-1.303	-1.9303	-0.64137	-0.57653	-0.42959
0.47889	0.73483	0.43504	0.48071	0.4693	0.65275	1.2572
-0.64863	-0.58308	-0.78629	-0.51848	-0.88545	0.14568	0.14457
0.80015	0.84757	0.81883	1.3712	0.94243	0.59465	1.6073
0.43261	0.36016	0.77964	1.2983	0.23653	0.56227	0.80726
0.38274	0.58643	0.33608	0.44314	0.34696	0.54884	0.33404
-1.8634	-1.967	-3.1704	-2.1385	-2.5386	0.2843	-1.2985
0.33822	0.014851	-0.52976	-0.78623	-0.71168	-0.23755	-0.93565
2.1841	1.9669	2.893	2.9976	2.05	2.5857	3.6653
0.6273	0.63367	0.33851	0.61244	0.84913	1.1329	1.1132
0.48249	0.51952	0.29704	1.0146	0.83163	0.18931	1.0428
0.7295	0.86473	1.2752	0.77695	0.88737	0.54847	0.71894
1.0778	1.1877	0.76054	0.93024	1.4239	0.66175	0.98197
0.014884	0.94757	0.38649	-0.018665	-0.40017	0.37301	-0.85291
-0.822	-0.87419	-0.83106	-0.098663	-0.54008	-0.53827	-0.32801
0.44201	0.80198	0.47122	0.74722	1.1526	1.0753	0.52234
-0.019866	-0.15783	-0.34587	0.14047	-0.54878	-0.15662	-0.30931
-0.29526	0.061341	0.16287	-0.058132	0.36613	0.39807	0.33827
0.22259	-0.16889	-0.18671	-0.51901	-0.85883	-0.28171	-0.34253
3.3565	2.5272	2.0482	1.1994	0.87224	0.13085	0.80169
0.99927	1.5493	1.5403	0.82336	-0.172	0.21133	0.35804
2.6002	2.2693	2.4772	1.489	0.35171	0.55685	0.81074
-0.16023	-0.52723	0.038957	-0.4387	0.3453	-0.092776	0.12532
0.46346	-0.12546	1.0133	0.66901	1.2889	1.0267	1.1235
2.1971	1.5069	2.2244	0.82732	1.0448	1.1104	1.6995
-0.29715	-0.63941	-0.62818	0.10705	-0.091952	-0.056189	-0.041576
0.46416	0.84376	0.67577	1.5337	0.62438	0.15115	0.40683
-0.38881	-1.0458	-0.78619	-0.68342	-0.41284	-0.13345	-0.25792
0.5904	0.24939	0.56783	1.0028	0.16378	-0.17657	0.079243
0.26121	0.61072	1.8233	0.63513	-0.54316	0.84677	-0.41218
-0.11004	-0.16514	-0.50443	0.79421	-0.74051	-1.4327	-1.2584
0.28382	0.22297	-1.0503	-0.019891	-0.50094	-0.4555	-0.38751
-1.177	-1.6263	-1.3526	-0.73846	-1.3651	-1.2317	-1.8335
-0.32851	-0.068682	-0.070569	0.74694	0.41638	0.30585	0.28267

-0.31299	-0.42905	-0.52125	0.02345	0.4431	0.34803	0.54714
1.1232	1.3246	1.6589	1.7455	1.5392	1.2052	1.255
0.028624	-0.084442	-0.30147	0.034078	-0.010795	-0.36009	0.039413
-0.025453	0.012753	0.056715	0.0036189	0.094757	-0.039881	-0.23876
-0.30496	-0.24721	-0.088359	0.024087	-0.44112	0.19487	-0.0854
0.42434	1.2174	0.60679	1.6056	0.29322	0.2764	0.34857
0.86454	0.59062	0.40085	1.0387	0.59595	0.047922	0.19962
0.20374	0.26555	-0.22175	0.41638	0.72676	0.31342	0.6279
0.46477	0.55577	0.47785	0.21028	-0.011851	0.26027	0.28962
-0.087829	-0.20189	0.077477	1.2589	1.1579	0.17587	1.2774
-0.28952	-0.18989	-0.086522	-0.50034	0.074399	-0.049319	0.028882
-1.825	-2.0045	-2.3865	-1.9335	-0.062618	-0.11961	0.26102
-0.03283	-0.17382	-0.14053	-0.39546	0.19424	0.17405	0.23566
0.24494	0.55323	0.27208	1.4518	0.54127	0.95412	0.85893
-0.4493	-0.15631	-0.091947	-0.080006	0.3193	0.55604	0.27929
0.21085	0.010568	-0.59791	-0.91955	-0.54801	-0.25293	0.014352
-0.2941	-0.19894	0.53444	-0.32527	0.60014	0.12153	0.34056
0.32207	0.77551	0.67453	0.94039	1.4957	0.38364	0.93163
-0.43343	-0.40659	-0.71856	-0.12177	-0.98506	-0.41938	-0.32537
-0.35961	-0.68655	-0.39733	-0.28458	0.2225	-0.025484	0.0077306
0.78053	0.64066	0.53213	0.41145	0.76536	0.59417	0.87495
0.095521	0.13606	-0.18787	0.48976	0.46725	0.08484	0.0038746
0.34941	0.68982	-0.73177	-0.87344	-0.83734	0.15428	0.099335
0.89142	0.61064	0.18167	0.16244	0.99154	1.7761	2.0266
0.40267	0.14093	-0.024796	0.28708	0.82043	0.53463	0.6111
0.27516	0.40524	-0.46825	0.31015	-0.40373	0.016744	-0.015458
-0.30844	-0.36056	-0.56972	0.036307	-0.10107	-0.089759	-0.13041
0.81678	1.1479	0.63471	1.1751	0.96763	1.0659	1.7025
1.2598	1.3377	1.6472	2.8282	1.2893	1.4718	2.0966
-0.30595	-0.19744	-0.06377	0.15937	0.24999	0.14663	0.40185
0.13063	0.22359	0.24123	0.078946	0.58646	0.57101	0.4459
0.011078	-0.053799	0.61723	-0.23203	0.096187	0.66609	-0.40398
0.29591	0.20825	0.67886	0.26429	0.1817	0.071781	0.27115
3.3556	3.1481	1.8261	0.49434	1.7876	1.8267	1.1856
1.7053	1.6584	3.1815	2.0096	2.5365	1.6794	1.7145
0.81321	0.77982	0.23353	0.70101	0.71602	0.86828	1.4937
0.024674	-0.18908	-0.48903	-0.31661	-0.21744	-0.37973	-0.27556
-0.17486	-0.18391	0.90387	1.066	0.55953	0.25241	0.46398
-0.5126	-0.45645	-0.60021	-0.43632	-0.34731	0.15529	-0.49278
0.38348	0.53057	1.5609	1.1923	1.475	0.25346	0.57306
1.9129	0.67657	1.3792	0.92618	2.0784	0.73735	1.4104
-0.097735	-0.21027	0.24987	0.50404	0.28251	0.24494	0.070934

0.19988	-0.11244	0.49158	0.20667	0.50786	-0.063698	0.14727
0.60998	0.32784	-0.042959	-0.8296	0.22984	0.9408	-0.033768
-0.62342	-0.60476	-0.70801	-0.69747	-0.030044	0.19618	0.13339
-0.38017	-0.25041	-0.83489	-0.78591	-0.26516	-0.1615	-0.049295
0.32701	0.63908	-0.18582	0.34697	0.17722	0.14197	1.0551
-0.27684	-0.27134	0.19808	1.5581	0.2072	0.43065	0.69699
-0.3155	0.0028442	-0.14457	-0.9392	-0.40099	-0.08357	0.12915
0.17454	0.45539	-0.94892	-0.70012	-0.76817	-0.43103	-0.54693
0.19188	-0.20566	0.052757	0.098268	0.40428	0.50897	0.29135
-0.69656	-0.75086	-0.29842	-0.91266	-0.64969	-0.35011	-0.41983
-0.016352	0.083457	-0.69185	-0.93098	-0.80068	-0.38237	-1.1907
0.25252	0.33609	0.68914	0.18132	-0.89189	-0.40376	-0.60134
-0.78535	-0.70968	-0.31181	-0.46796	-0.67065	-0.031073	-0.26318
0.40519	1.0829	0.70371	-0.22555	0.48776	0.28388	-0.030366
-0.21331	-0.22697	0.91564	0.79255	0.6279	0.62692	0.60758
-0.89246	-1.0787	-0.51573	-1.3125	-1.0409	-0.85292	-0.2346
0.68689	0.55824	0.41641	0.91436	0.39189	0.18556	0.28461
0.73505	0.86876	0.95683	0.7132	0.93218	0.69545	0.46252
-0.044316	-0.38293	0.76798	0.52375	0.68114	0.50045	0.50265
-0.41837	-0.26883	1.0135	0.76913	0.14976	0.42408	0.17995

test_GSE134	test_GSE112	test_GSE112	test_GSE112	test_GSE366	test_GSE451	test_GSE451
ref_GSE1344	ref_GSE1121	ref_GSE1121	ref_GSE1121	ref_GSE3665	ref_GSE4511	ref_GSE4511
GSE13444	GSE1121	GSE1121	GSE1121	GSE3665	GSE4511	GSE4511
GEO	GEO	GEO	GEO	GEO	GEO	GEO
cDNA	affymetrix	affymetrix	affymetrix	affymetrix	affymetrix	affymetrix
ISOBUTANOL	arcA_b4401:	soxS_b4062:-1				
1110	1173	1187	1189	1224	1242	1243
0.55853	0.01443061	-0.038132	0.1800611	0.01016922	-0.2772281	-0.2230447
-0.6053	-0.192307	-0.5438881	0.2168583	-0.0290785	0.03716115	-0.023742
0.41511	-0.3176204	-0.2121726	0.2591158	-0.0402547	-0.0743019	-0.1713672
0.80738	0.07859337	0.2368288	0.2296502	-0.0459981	-0.1431205	-0.2726271
0.10824	0.1098069	0.1415556	0.03821826	0.04119369	-0.368403	-0.534246
-0.39414	0.116277	0.04212472	0.06583082	-0.1489876	-0.4626964	-0.1594475
-0.21198	0.1108671	-0.0050052	0.1345497	0.3956857	-0.3321623	-0.1451764
0.67263	-0.146048	-0.3183113	0.2586306	-0.1699096	-0.4169275	-0.2653871
-0.074932	-0.2171874	-0.1954195	0.1951578	-0.3313271	0.29131282	0.33198645
0.96327	-0.1909972	-0.1985157	0.1883138	-0.0177953	-0.0784855	-0.1554647
0.84371	-0.3740714	-0.5815318	-0.0084175	-0.1652297	-0.1359225	0.05910673
-0.83915	0.04471012	-0.3565949	0.07724564	-0.2875831	0.24707652	0.19500591
-0.28427	-0.0510165	-0.1576202	0.08508749	-0.2861391	-0.092201	-0.0752649
2.6144	-0.0559929	0.1102249	0.1981749	-0.0522566	0.26581686	0.24612278
0.85323	0.1411984	0.1497011	0.1867868	-0.0264076	-0.0866855	-0.1394889
-0.15556	0.07550398	0.04509214	-0.0565744	0.00077036	0.06409174	0.09363686
-0.32937	0.1163761	0.1091197	0.2520054	0.26574	-0.1392502	-0.0654705
0.070923	0.258573	-0.2580512	0.1892888	0.01196319	0.27461973	0.25891225
0.50503	-0.1489192	-0.1714853	0.107268	0.08223532	0.1411433	0.27493676
0.35759	-0.1446153	0.04313189	0.2128044	0.00632784	-0.1096713	-0.2212621
-0.33939	-0.0424489	0.04891129	0.08134757	0.2078499	0.01488517	-0.0951663
-0.61748	0.08034425	-0.0573018	0.109833	0.078184	-0.2735303	-0.0489324
-0.65884	0.553408	0.02569023	0.2046812	0.1086188	0.11741069	0.10931524
0.3404	-0.2442221	-0.2272684	0.04391404	-0.0012048	0.23602833	0.37010768
2.9186	-0.2097969	-0.1707504	0.1870973	0.1735578	0.10507873	0.10443684
0.48253	-0.0450784	-0.0836579	0.0268621	0.1849375	-0.1205	-0.2094971
1.7976	-0.1351008	-0.2950486	0.4368978	-0.23731	-0.1378247	-0.0386153
-0.48761	-0.0520017	0.00348598	0.3096741	-0.2442255	-0.1756147	-0.0925478
0.71323	-0.149927	-0.3744754	-0.0865484	-0.10182	-0.4015486	-0.4502982
1.557	-0.0721818	-0.2580493	-0.0128403	-0.1472421	0.22504415	0.2681583
1.1732	-0.270155	-0.5434635	0.03956085	-0.0592733	0.10535273	0.17802072
-0.38637	-0.0785157	-0.1768573	0.06073567	-0.130227	-0.291241	-0.2394845

0.974	-0.0102972	0.1103356	0.1698493	0.00200903	-0.3318911	-0.1345764
0.51005	-0.1427584	-0.3902984	0.02769292	-0.2425044	0.27500028	0.32934847
-0.019978	-0.3557959	-0.3739163	0.02687144	0.07434871	0.42517929	0.35536937
-0.84681	0.1415829	0.1302386	0.2454125	-0.5745415	0.06099817	0.05305474
0.43777	-0.3057995	-0.3454291	0.0641511	0.00898476	0.17127937	0.24597039
1.1937	-0.4487488	-0.5540744	0.02697916	-0.0306242	-0.0606003	-0.1223995
-0.37805	-0.4568397	-0.3674678	-0.1992195	-0.0413525	-0.1176208	-0.0137801
0.10663	-0.1165191	-0.0952642	0.1558659	0.00511055	-0.2661909	-0.1064087
-1.1799	0.05660787	-0.0712634	-0.2382661	-0.0068544	-0.2860055	-0.2337298
0.08258	-0.1061306	-0.1476036	-0.0171436	-0.1551022	-0.3250264	-0.2025971
-0.21095	-0.1337676	-0.3603082	0.02726841	0.05647531	-0.042516	0.06064047
-0.27965	-0.0721083	-0.5232455	0.07696197	-0.2138513	-0.1186957	-0.113741
-0.98864	-0.1209518	-0.0990517	0.026715	0.09129789	-0.2589792	-0.0674895
0.0059698	0.00349815	0.08490964	0.1898663	0.2011603	0.032298	-0.0293068
-0.26292	0.06641373	-0.1866288	0.1002627	-0.1337747	-0.1635477	-0.1155674
-0.76045	0.06146836	-0.0561572	0.1118192	-0.0880448	-0.6971688	-0.483211
0.18077	0.06304196	0.04701297	-0.0487015	-0.0261424	-0.0014472	0.12420497
0.4262	0.1391628	-0.0141035	0.00575926	0.02247045	-0.2221646	-0.2929934
-0.31649	-0.0324586	0.078819	0.2700526	-0.1047657	-0.4779082	-0.2362592
0.36369	-0.0055285	-0.0505539	-0.131076	-0.1045378	0.01493557	0.04582001
1.1065	-0.5985798	-0.0332852	0.03263898	-0.0207977	-0.3985572	-0.2576307
1.2628	-0.0145003	-0.0504173	0.2168965	-0.0883905	-0.4068205	-0.4403996
-0.36847	-0.1268661	-0.3484531	0.1004094	0.02142874	0.20737736	-0.0299253
-0.25807	-0.0923252	-0.1989049	-0.1638512	0.0787537	-0.3602235	-0.1974255
-0.22059	-0.0063412	-0.1287805	0.07358633	-0.0179643	-0.2013483	-0.2431643
1.4333	-0.00606	-0.2498928	-0.1431584	-0.1793138	-0.0993514	-0.047271
-1.4225	-0.3262793	-0.3218444	-0.032798	-0.098651	0.11903874	0.21554979
0.64478	-0.190613	-0.0665895	0.09532054	0.1380577	-0.091281	-0.1582132
0.032855	0.08243692	0.0010743	0.1922409	0.06173963	-0.129456	-0.0193945
-1.404	0.1253075	-0.07772	0.1716679	-0.0761998	-1.0926182	-0.6973221
-0.1523	0.03792755	-0.1226598	0.1087434	-0.2433428	-1.5155893	-0.8893667
0.23326	-0.120706	-0.0618066	-0.0473343	0.07221883	0.09446789	0.10975996
0.28577	-0.0165545	-0.0825449	0.01408659	-0.2514244	-0.1657969	-0.0126484
0.60317	0.06422012	0.2055758	0.1870273	-0.2868441	-0.0176913	0.07896555
-0.79933	-0.1211697	-0.1247907	0.06592636	0.09203662	-0.0425896	-0.0908477
0.46512	-0.0013187	-0.0099898	0.1432052	0.00540284	0.01697289	0.09013363
1.4095	-0.0537612	-0.0109084	0.201787	0.0393871	-0.2945493	-0.3239824
-0.037976	-0.0572901	-0.143754	0.1090678	-0.1112745	0.32720187	0.21398317
0.72663	-0.163845	-0.100562	0.1382931	0.05068316	-0.5933893	-0.2218435
-0.92481	-0.1356849	-0.0995615	0.0746564	-0.0760564	0.15589572	0.14584293
0.73648	-0.1635678	-0.1109984	-0.0398026	0.05087908	-0.1470472	-0.1349641
0.38029	-0.3046336	-0.2048853	0.00569734	-0.0238614	0.38792939	0.28130473

0.11599	0.09421592	-0.0687703	0.2863376	-0.3216467	-0.3298099	-0.4141373
0.11042	-0.0826901	-0.1233725	0.09726895	-0.0372039	-0.4459401	-0.2421045
0.21697	-0.1864524	-0.1154294	0.03788554	-0.0318341	-0.3910105	-0.2274321
-0.24501	0.447069	-0.2923514	0.1097518	0.1430004	0.02115387	-0.1488217
0.56152	-0.2648629	-0.2297659	-0.0015848	0.03507503	0.1914601	0.10642784
0.10072	-0.2721521	-0.4098918	-0.0952245	0.1668892	0.06708675	0.17156028
-0.59202	-0.0504703	-0.1268101	0.1334269	-0.0552478	-0.1901495	-0.0927721
0.87297	-0.1047324	-0.0241358	-0.1548699	-0.1517104	-0.3625781	-0.1760595
0.20866	-0.2034811	-0.4447777	-0.1414731	-0.1407583	-0.1171543	0.03940423
0.90399	-0.0550059	-0.125498	-0.0038475	-0.0864888	-0.083994	-0.107678
-0.095608	-0.1328244	-0.1776946	0.1029576	-0.3089689	0.15782787	0.12786653
0.22824	0.03854487	-0.1526779	0.0677244	0.03231576	-0.0740625	-0.0941481
0.21069	-0.1481404	0.00093874	0.00633911	-0.0740834	-0.1433728	-0.1834079
0.72468	-0.1638755	-0.1239952	-0.3281791	0.1156839	-0.2308726	-0.1170449
-0.78311	0.1575735	-0.3396511	-0.0154379	-0.27876	0.07562226	0.17547289
-0.81895	-0.1162427	-0.1194798	-0.0770982	0.1516592	-0.4722711	-0.3669032
0.3992	-1.347843	-0.2909826	-0.0105257	-0.0365827	0.14012784	0.21846318
-0.72561	-0.2043044	-0.2486626	0.02101566	-0.1705928	0.22863061	0.21325299
0.8585	-0.1374547	-0.1224897	-0.1012115	-0.1861	-0.4335061	-0.4783164
-0.65907	0.1160076	-0.0200993	0.2129609	0.08684516	-0.3134531	-0.3817305
-0.10575	0.01810451	-0.0124966	0.02106475	-0.0384992	-0.0375343	-0.0623057
0.45406	-0.0163274	-0.1511046	-0.1843996	-0.078989	-0.058097	-0.1563092
-0.64924	0.0591372	-0.1008848	-0.0405655	-0.1244101	-0.2193479	-0.1037021
-0.34141	0.02052068	-0.1260712	0.08782185	-0.0235419	0.00517617	-0.0577412
-1.3788	-0.1188421	-0.1416841	0.01667655	0.05161912	0.13162103	0.0895247
-0.68591	0.1381462	-0.0675631	-0.1348204	0.1409231	-0.2271289	-0.2108326
0.69307	-0.1691784	-0.3118193	-0.1767106	-0.0501163	-0.2922672	-0.3232799
-0.2566	0.01164357	-0.0534405	0.09850886	0.01483833	-0.2428175	-0.2983768
-0.18881	0.028423	0.030465	-0.0015387	-0.053029	0.064063	0.050534
-0.1168	-0.365	-0.25326	0.094412	0.081165	-1.0293	-0.50439
-2.5323	-0.097638	-0.18207	0.16924	0.022511	-1.1215	-0.88225
0.24663	-0.42056	-0.6593	-0.29252	-0.10641	-0.042117	-0.038006
0.67946	-0.15789	-0.43517	7.27E-05	-0.073548	0.044516	-0.077915
-0.22654	-0.029895	0.088013	-0.078086	-0.052444	-0.095499	-0.32552
0.56273	0.28138	-0.16733	0.019668	-0.19478	-0.097747	-0.34342
1.3998	-0.1287031	-0.2928662	0.01923906	0.203405	0.06233368	0.05729108
0.21157	-0.3286426	0.00798336	0.1265018	0.1131857	0.5076475	0.246088
-1.0238	-0.153878	-0.0325233	-0.1930984	-0.1732985	-0.3927259	-0.2499293
0.50426	-0.0852089	-0.1436122	0.04094843	-0.0127591	-0.281191	-0.1370138
-0.1355	-0.0293238	-0.0742844	0.1280613	0.1282721	-0.2351715	-0.3528229
0.28461	-0.0291856	-0.1331098	-0.0884731	-0.0275549	-0.0766025	0.03258308
0.055253	-0.0617917	-0.1577694	-0.0026087	0.0454814	0.006169	0.05807288

-0.26578	0.09864284	0.1099264	0.09313104	0.2454561	-0.6667316	-0.657932
-1.6409	0.05950478	0.2085307	-0.081652	0.03053796	0.05620435	-0.0202667
-0.37861	-0.1489671	-0.132854	-0.1704168	0.01681391	0.01801425	0.08108945
0.22698	-0.2813707	-0.2632067	-0.1972513	-0.1275826	0.08308077	0.26120425
-0.30062	-0.087827	-0.1194622	-0.0744464	0.02519107	-0.0422061	-0.00406
0.89761	0.00130014	-0.1415472	0.03558937	0.02978797	-0.1316257	-0.1420122
0.18426	-0.1587776	-0.2282812	-0.1242121	-0.0931866	-0.0937791	-0.0852632
-0.46244	-0.2104958	-0.189757	-0.1993586	-0.0910619	-0.0466921	0.05496309
-0.60949	-0.0908387	0.00250927	-0.1553417	-0.0963488	-0.1719482	-0.0872845
-0.18049	-0.2370251	-0.1859631	-0.3084333	0.03549985	-0.0468986	-0.0251038
-0.70072	-0.0341411	-0.3272869	-0.0741498	0.09976936	-0.3293915	-0.1575468
0.75456	-0.2197174	-0.0300582	-0.2906398	0.08318857	-0.0957788	-0.0507682
-0.37914	-0.0089098	-0.2323805	0.1195664	-0.044271	0.182484	0.10038343
-0.53491	-0.2793458	-0.4301116	-0.0540829	0.02075638	0.15630791	0.15081761
-0.06188	-0.3800814	-0.4016099	-0.0829606	0.105203	0.08351658	0.04597294
-0.061373	0.01200831	-0.1039684	0.0226586	-0.0848605	-0.2295228	-0.1789833
-0.22755	-0.2364826	-0.1759861	-0.120102	-0.0966102	-0.3109865	-0.1669276
0.052663	-0.0172857	-0.2620426	-0.1036562	-0.1263699	-0.5670132	-0.3910954
0.49068	-0.0614178	-0.5250488	0.0161174	-0.0270377	-0.0143866	-0.0308986
-2.054	-0.0243786	-0.077722	-0.350026	0.03088906	-0.2143033	-0.1714409
-1.3129	-0.2073496	0.07792128	-0.4238503	-0.0619462	-0.0969704	-0.1785106
0.19925	-0.1153047	-0.1930279	-0.0345408	0.0226234	0.11832399	0.18884463
NaN	-0.073547	-0.16443	-0.39026	-0.11315	0.128	0.23767
0.15944	0.03379999	-0.4943516	0.04837507	-0.1026685	-0.1104395	-0.1522792
0.040941	0.05174521	0.2144812	0.121286	-0.1053	0.13115099	0.07946083
1.1623	0.1565967	-0.2771166	0.3147429	0.1298959	0.03538212	0.02520638
0.59294	-0.1591354	-0.0835522	-0.2607522	-0.0538098	-0.3542557	-0.1761315
0.77408	0.2172266	0.09785189	0.03243709	0.151489	-0.1788633	-0.2744629
-0.31466	-0.0242329	-0.3492733	-0.0901356	0.09060838	0.17390422	0.08929294
0.42242	-0.1695439	-0.221445	0.07226571	0.077465	0.35098391	0.35844129
-0.49559	0.1040391	0.06170426	0.133456	0.1860647	0.14884578	0.18384982
0.99575	-0.1195785	-0.0290573	0.07412414	0.00565363	0.12274895	0.19473852
0.22534	-0.1820024	-0.3483845	-0.0004655	-0.0692219	0.05375264	0.04252346
-0.39426	-0.0982061	-0.3346156	-0.010861	0.1246898	0.00932198	0.0154721
0.74169	-0.0493716	-0.3250378	0.02500548	0.1379912	0.04708272	0.11910214
0.59076	0.2191562	0.3175121	-0.0915202	-0.0053366	-0.0131205	0.01106065
0.16749	-0.0842869	-0.0601889	-0.1880217	-0.3158662	0.07592236	-0.0322197
-0.069093	0.06421442	-0.00738	0.130435	-0.000211	-0.1356717	0.00120709
0.23253	0.1859926	0.29904	-0.2109529	-0.029515	-0.1010111	-0.106997
0.81235	-0.0237283	-0.0997009	-0.2852611	0.1154017	0.08172509	0.10870447
0.25685	-0.1056191	-0.1473111	-0.0249051	-0.0870971	0.0344925	-0.0386326
-0.24048	-0.2363983	-0.3330686	-0.3628698	0.1044207	-0.3681677	-0.0675228

-0.12169	0.07665857	-0.1411602	-0.0042483	-0.0473795	0.28726432	0.21671896
0.19661	-0.0912992	0.00616586	-0.181422	0.03244878	-0.1287899	-0.1709558
0.088434	0.1349696	0.1161538	0.08325542	-0.228553	0.01365758	-0.0323273
-0.41565	0.01178463	0.00041796	0.07904318	-0.1895059	0.21662036	0.14364785
0.63342	0.1416107	0.2210406	-0.2320917	0.192238	0.08339652	0.03110316
0.18146	-0.1473433	0.09424095	0.1124676	-0.1135022	0.11513035	0.06575425
0.21603	0.02511167	0.0762421	-0.0549875	0.08817861	-0.4674397	-0.2350772
-0.5751	0.2386516	0.025554	0.05754375	-0.0937732	0.00321268	0.0532348
0.52267	0.1035531	-0.2247842	-0.0621453	-0.2991013	0.05012677	0.11102998
0.12503	-0.02677	0.00111648	-0.0483375	0.03991252	-0.2040454	-0.1842466
0.25959	0.07021355	0.08674802	0.127531	0.1899691	-0.0007956	0.11530301
-0.46182	-0.0360239	-0.0952743	0.11917	-0.0200031	-0.0524121	0.04351822
0.087285	-0.0422014	-0.051442	-0.6057496	-0.0160342	-0.2619918	-0.2870173
0.4617	0.1222866	0.1024861	-0.3889378	-0.1012741	-0.0458362	-0.1048368
0.082885	0.0270712	0.1380825	0.01108052	-0.1351	0.15193296	0.03579443
2.1782	0.05483899	0.06323873	0.1117881	-0.2871884	-0.2727509	-0.3519265
-0.40568	-0.1723162	-0.2086374	-0.0066995	-0.2278545	0.01769041	-0.1279643
0.26031	-0.0471189	-0.2570142	-0.1518587	0.02820599	0.26639645	0.1531156
-1.1039	-0.0589543	-0.1275217	-0.4281715	-0.1488289	-0.2035708	-0.0889619
0.45244	0.01324552	0.05009398	-0.0634345	-0.0336946	-0.3518133	-0.1693364
0.20442	0.1291476	-0.0154424	-0.019571	-0.1131141	-0.3864744	-0.2288365
0.20886	-0.0348708	0.1631622	0.1653223	0.0757057	-0.1171314	0.00578814
-0.039478	0.04204522	-0.0085115	-0.0160262	-0.1308147	-0.0073397	-0.1526382
-0.37895	-0.1825903	-0.003579	0.08041888	-0.0386188	0.15719965	0.12014066
0.46689	0.194688	0.03476609	0.1848035	-0.0110305	0.4455387	0.34867675
0.17005	0.02436912	0.1288792	-0.2832474	-0.1223525	0.04577415	0.0342527
0.095146	-0.0393201	-0.1201287	0.02450397	-0.0464739	-0.3268168	-0.1134329
0.3067	0.03921696	0.01746799	-0.5021527	-0.0921172	-0.1511405	-0.052814
0.71204	-0.0755715	-0.0459177	-0.581009	-0.0970642	-0.3436342	-0.170921
1.1173	0.00604184	-0.158989	-0.0143257	-0.1420055	0.50945109	0.36207172
0.36509	-0.0132273	-0.1716021	-0.0701985	0.2327985	0.3339775	0.17932923
-0.83737	-0.0363604	-0.0646601	-0.2708346	-0.1793926	-0.1649494	-0.1490739
0.66187	-0.0616344	0.0402753	-0.359374	-0.0782518	-0.1655249	-0.1080008
-0.57666	0.03728532	-0.0323789	-0.040493	-0.2884837	-0.173729	-0.2128336
0.51792	0.02689331	-0.0650293	0.1072115	0.1324656	0.0689989	0.02324584
-1.2406	-0.2952609	-0.4265827	-0.203035	0.0430641	0.06397779	-0.0016482
-0.43717	-0.0332074	-0.0576033	-0.4850838	-0.034197	-0.3427512	-0.3083195
0.67485	-0.1031286	-0.1362143	-0.5011128	-0.0425569	-0.1642713	0.05739156
-0.50501	0.531504	-0.2299561	-0.1321503	-0.0149755	-0.368785	-0.1630771
-0.38839	-0.0389287	-0.0373205	0.02621276	-0.16863	-0.0101518	-0.0950415
0.1479	-0.2351078	-0.523986	-0.3412439	-0.1632755	-0.0615826	-0.1310178
1.1823	-0.1102535	-0.6498099	-0.3618522	-0.2787153	-0.1028305	-0.0240989

0.19499	0.0770344	-0.0783516	-0.0309128	0.05508951	0.39283921	0.31419806
0.13715	-0.0390397	-0.1476944	-0.0680082	0.05014393	0.22850411	0.09623239
-0.29946	-0.0422916	0.06135546	-0.852723	0.2218611	-0.1680459	-0.2875991
-0.030707	-0.0195673	0.08969952	-0.4263042	0.1224972	-0.2521991	-0.2331169
0.095573	-0.1895487	-0.1173754	-0.2569099	-0.1740772	0.08986261	-0.0870146
1.101	0.06694751	-0.1186442	-0.0869153	-0.1871447	0.00681506	-0.02458
1.3418	-0.0707434	0.1196221	-0.8890827	0.1110903	-0.1215755	-0.1616969
0.14924	0.08185967	0.01644543	-0.0505335	0.1664112	-0.1379129	-0.2315771
-0.3881	0.01468166	0.05915026	-0.0189764	-0.1888641	-0.2127195	-0.1997735
0.18173	-0.070044	-0.1203443	-0.1205621	0.068441	0.09737574	0.00822645
-0.38048	0.00127821	-0.0100875	-0.1886313	-0.2864331	-0.3491351	-0.2214276
0.72842	0.06265983	0.2229457	-0.2593468	0.1325314	-0.3320034	-0.206939
0.51057	0.04497867	-0.2010071	0.00039109	-0.0991004	-0.0254157	-0.0452507
0.42334	-0.0954073	-0.1197876	-0.116522	-0.059843	-0.0948314	-0.0638632
-2.1571	0.1517881	0.02367662	-0.097411	-0.2168307	-0.4710255	-0.4691647
0.19542	0.1617533	0.03711935	0.06192139	-0.3283126	-0.5713987	-0.5806625
1.821	-0.106289	-0.2513034	-0.2278787	-0.0387278	-0.2901493	-0.1407002
0.27851	-0.1099378	-0.0892828	-0.1733517	-0.0098071	0.42819045	0.21190103
0.14236	-0.063157	-0.069029	-0.2604704	-0.1226001	-0.2653089	-0.2591546
0.70722	0.2089801	0.5723169	-0.5217922	-0.0018057	0.10491673	0.1497349
0.49925	-0.1677113	-0.0906742	-0.1204062	-0.0653621	-0.1934569	-0.0957157
0.48096	0.1580758	0.2267051	0.1389997	-0.0216518	0.33250139	0.1821689
-1.0115	-0.0949603	0.05098871	-0.081467	0.01736567	-0.1671354	-0.1672904
0.56837	0.2265918	0.2722326	-0.4533906	-0.1277171	0.17482441	-0.0585885
-0.24038	0.04394591	0.2202185	-0.0051494	-0.1014893	0.03387321	-0.1033645
-0.37865	0.2205253	0.1331498	-0.0609806	-0.2835306	0.54715	0.24892478
0.14265	-0.3409584	-0.0136927	-0.6596169	-0.1697644	-0.0409559	-0.0724926
1.9636	-1.058109	-0.3685375	-0.4560312	0.08050424	-0.2171324	-0.2333752
1.9965	-0.3945683	-0.1816956	-0.3724761	-0.175647	-0.2481451	-0.1687556
1.9088	-0.427553	-0.2353884	-0.2284334	-0.074545	-0.1240205	-0.0924287
0.40079	-0.1149579	-0.174037	0.04702277	-0.1204406	0.0227929	-0.0152324
-0.054495	0.04589298	0.02877211	-0.0321982	0.0239826	0.26834863	0.20693732
1.9818	0.03306334	-0.1388257	0.1554363	0.04949819	0.01926936	0.10539269
-0.65005	-0.0857259	-0.336521	0.3231565	-0.145011	0.08234664	0.00145457
0.53978	-0.09311	-0.0100685	-0.6495941	0.07620551	-0.2880012	-0.26607
-0.66312	0.00418222	0.08343652	-0.2456829	-0.0764588	-0.2149561	-0.1334405
1.1162	-0.1132582	-0.0250309	-0.0584775	0.02696019	-0.0121911	-0.0842555
0.53614	-0.061639	-0.2383121	-0.443943	-0.1539502	-0.079479	-0.1980044
-0.31219	0.2815417	0.3296478	-0.2240804	0.1352345	-0.2738875	-0.1618149
-0.52689	0.1649893	0.1859353	-0.588062	0.07176084	0.08082423	0.12968308
-0.44366	0.1244152	0.1320344	-0.2764568	0.01625504	0.2239284	0.2712389
0.60971	0.05680217	0.1038117	-0.3252644	0.00130135	-0.0119839	-0.0848995

0.11002	-0.0642187	0.06671116	-0.0448097	0.09530571	0.10783835	0.10164795
0.868	0.14039	0.14459	0.089403	-0.017114	-0.0054632	-0.17316
0.092538	0.07942363	0.03501684	-0.0288594	-0.0673291	0.21037981	0.05230821
0.41356	0.0802589	0.03399856	-0.2741798	0.07357217	0.04598873	0.0492712
-0.12278	-0.0321242	-0.1703403	-0.4323816	0.03504156	-0.1246824	-0.059862
-0.46777	0.02917158	0.07710419	-0.3720678	0.06261245	-0.1472051	-0.1309035
0.62051	-0.0301841	-0.1144927	-0.1952583	0.1419495	-0.3168091	-0.4262606
0.29591	-0.13884	-0.11827	-0.18398	0.080783	-0.28022	-0.19241
0.82155	0.09149478	0.2333316	0.1521988	-0.2060042	0.27413305	-0.0011433
-0.6994	0.09537464	-0.0516093	-0.4070115	-0.069235	0.28854872	0.2491211
0.2468	0.1573703	0.1826006	-0.399516	0.09477545	0.47483596	0.30527793
-0.3143	0.4347781	0.3757674	0.1919233	-0.030644	-0.0170855	-0.1619032
-0.10372	-0.0967339	-0.0241134	0.06071934	-0.1125564	0.0673192	-0.0839438
0.42298	0.3722688	0.353694	0.2351962	0.05124444	0.2447945	0.07007948
0.45739	0.00195431	-0.0582855	-0.2692101	0.2811616	0.29604896	0.20960705
-0.025091	-0.0689091	-0.0946924	-0.2540232	0.04772583	0.23967045	0.11400715
0.053815	0.01888997	0.1957948	-0.1905458	0.1896403	-0.5134763	-0.3305628
0.99851	0.1201821	-0.0013242	-0.1896331	0.05229754	0.13821755	0.05057917
1.5047	0.2091125	-0.0962485	-0.1050836	-0.0867063	0.38967332	0.18297251
0.079348	-0.144499	-0.00525	0.00669264	0.0989589	0.03017066	0.17189899
0.42812	0.00303624	0.2388286	-0.0923396	0.1067595	-0.0904126	-0.0298975
-0.32925	-0.0020501	-0.0284283	-0.1634211	-0.0549969	-0.0737706	-0.2673207
-0.35631	0.04860158	0.00426124	-0.0040853	0.20738	-0.0210215	-0.1368522
-0.73209	0.02118365	-0.1016295	0.1255133	-0.1773344	-0.1793821	-0.3027473
0.38312	0.08344913	0.2496201	0.1802078	0.2174968	0.2210324	0.13635326
-0.049802	0.1377923	0.04433694	8.72E-06	-0.0516417	-0.3010457	-0.2588326
-0.066223	-0.1800892	-0.1300405	0.09741491	-0.1243311	-0.1414399	-0.1064297
0.80965	-0.2220079	-0.1436531	-0.1807111	0.010154	-0.4443055	-0.1583788
0.5398	0.0005633	-1.425427	-0.7594031	-0.3128116	0.05092591	0.19597767
0.29504	-0.1562576	-0.238045	-0.5066116	0.01510036	0.09753788	0.03941167
0.76907	0.04481115	0.07637208	-0.141453	0.02931073	0.12834216	0.06943304
-0.2426	0.3193405	0.4503501	-0.0011898	0.1298567	0.1564948	0.11465075
0.82744	-0.1742177	-0.049826	0.09253724	-0.11016	-0.0230999	0.01389035
3.4824	0.07499917	0.2186816	0.2081645	-0.0278518	-0.0568758	0.06667272
1.9934	-0.0394273	-0.0314361	0.04431145	-0.0293657	-0.1109367	0.00796383
0.01519	-0.0026919	0.00641697	-0.0477807	-0.0365007	-0.0902203	0.00599067
0.51929	0.02886687	-0.1378613	-0.1926169	-0.2117584	0.04642332	0.11829829
0.35553	-0.004423	0.01790308	-0.5379686	0.04113827	0.3959971	0.2489661
-0.070413	0.1205807	0.02897552	-0.4632573	0.02426316	-0.0459909	-0.116278
1.5117	-0.3849802	0.1774263	0.1647365	-0.2500845	0.09538884	-0.1403901
1.0294	-0.1992368	-0.1023572	0.2364904	-0.0815839	0.01351611	0.03080883
0.45786	0.08480305	0.04125251	-0.5662066	-0.1392226	0.299253	0.07234667

0.79959	-0.1225697	-0.0067918	0.06169171	0.04132391	-0.3247722	-0.4318528
0.45262	0.1599775	0.1612997	0.0001738	-0.0805708	0.05471778	0.23799669
-0.18198	0.0774386	0.09572443	-0.2589319	-0.0662568	0.12875914	0.16735818
-0.17162	0.1851809	0.09863624	0.1439877	0.1740486	-0.312782	-0.0559601
0.42086	-0.145192	-0.0129241	0.2011409	-0.1496916	-0.1132251	-0.0306698
-0.53977	-0.1107028	-0.1378391	-0.102261	-0.0194583	-0.3648419	-0.3833268
0.16612	-0.0780215	-0.011675	0.1089377	-0.1412351	-0.0315119	-0.0754518
0.22992	-0.1931803	-0.0820875	0.1423189	-0.272238	0.01825841	-0.0211651
-0.015376	0.00770377	-0.056867	-0.7342606	0.07764781	0.11261237	-0.03819
-0.41737	0.2760651	0.2758754	0.07565068	-0.061045	-0.1857245	-0.212401
0.56134	-0.0443618	-0.0180919	0.1050786	-0.108282	0.0319729	-0.0235872
0.58608	0.04814888	-0.0801461	-0.1034661	-0.1766447	0.10176702	0.10221961
0.33946	0.1038359	0.2471979	0.03122315	-0.0350888	0.03532244	0.1075245
0.16619	-0.1297705	0.0993398	0.00499199	-0.0001552	-0.1455526	-0.1329038
0.53623	0.07998937	0.1490251	-0.0526443	-0.2032478	0.08096712	0.24771362
0.15433	-0.0365638	-0.0604382	-0.2695017	-0.1952738	0.17396038	-0.0181592
0.087187	0.0848381	-0.0435863	-0.0882188	0.07185181	0.27509683	0.23588728
0.72717	0.06784205	0.02905007	-0.2501704	0.01993765	0.30749311	0.3157227
0.018708	0.03076765	-0.0472663	-0.7026714	0.07403402	0.30806276	0.09396926
0.14039	-1.440167	0.5269701	0.1685309	0.00539406	0.41500552	0.23264252

test_GSE451	test_GSE451	test_GSE456	test_GSE477	test_GSE477	test_GSE477	test_GSE477
ref_GSE4511	ref_GSE4511	ref_GSE4562	ref_GSE4778	ref_GSE4778	ref_GSE4778	ref_GSE4778
GSE4511	GSE4511	GSE4562	GSE4778	GSE4778	GSE4778	GSE4778
GEO	GEO	GEO	GEO	GEO	GEO	GEO
affymetrix	affymetrix	affymetrix	affymetrix	affymetrix	affymetrix	affymetrix
	pH:2	yceB_b1063:	TIME:1	TIME:1	TIME:1	TIME:1
1245	1249	1275	1288	1289	1290	1292
-0.4105235	-0.1117979	0.38969808	-0.1361591	-0.2453051	-0.0116957	0.01958887
-0.0975171	-0.0697808	-0.4118119	-0.1059582	-0.227956	0.0185829	0.04624519
-0.2459582	-0.2507351	-0.4472193	0.06649328	-0.1681618	-0.0360787	0.07498524
-0.124297	-0.2506696	0.00430769	0.19940625	0.15118064	0.04420724	0.21820575
-0.3207308	-0.3367578	-0.2067639	-0.1544694	0.10327924	-0.1321365	-0.0818059
-0.1644823	-0.3635749	-0.2852043	0.06349837	0.02358252	-0.1216194	0.0835916
-0.2766838	-0.6364809	-0.4095675	-0.3307782	-0.2942823	-0.4998578	-0.2634837
-0.2185445	0.13452887	0.62179882	0.12255864	0.06194224	-0.1337143	0.2555383
0.0737008	0.20198449	0.07810531	-0.0269038	0.10380029	0.03798941	0.07874707
-0.2369457	-0.2791935	0.24492224	0.38157178	0.2339475	0.27609631	0.22028032
-0.2297018	-0.208651	0.45923157	0.3594537	-0.037641	0.00933146	0.16694485
0.1627353	0.28992611	2.3477162	0.86020028	0.85655698	0.97430175	0.64361945
-0.2118931	-0.1022352	0.81144013	-0.0085614	0.11708746	-0.0983653	0.05314753
0.46329737	0.38928989	-0.551718	-0.1335489	0.08370088	-0.0751518	-0.0339728
-0.0251851	0.13835092	0.52872531	-0.0526437	0.00522337	-0.0474276	0.14787508
-0.0401933	0.06797406	-0.3657825	0.20996764	0.16073745	0.35102429	0.1416002
-0.0466745	-0.051707	-1.3660979	0.08082102	0.25119187	0.20436524	0.03723577
0.21579306	1.0162277	0.17350259	-0.4070151	-0.3603158	-0.3588694	-0.3718807
0.15345085	-0.0480502	-0.0490194	0.07352489	-0.0389401	0.00561269	0.14776192
-0.1466167	-0.0287436	-0.8005086	0.04882477	0.18356293	0.03561588	0.12611684
-0.1485281	-0.0026524	0.12688968	0.11431053	0.01412872	-0.0353291	-0.0319509
-0.1287864	-0.1761932	-0.5597171	-0.0829993	-0.1308363	-0.2963132	-0.059026
0.16913759	-0.1337365	-0.9048647	-0.3549855	-0.0533275	-0.2485874	-0.2019101
0.08968732	0.35054089	-0.1487765	0.00546084	-0.0128969	0.04213063	-0.0120877
0.02358498	1.1318947	0.03385427	-0.1630301	-0.1441705	-0.2987719	-0.0710008
-0.0662099	0.06765672	0.30022063	-0.0875975	-0.0651835	-0.2446792	-0.0764054
-0.4109712	0.0264724	1.1536619	0.26674664	0.03620127	0.08198402	0.36429328
-0.7647954	-0.4487494	-0.5950001	0.27816558	0.28675355	0.13008681	0.29053616
-0.5046695	-0.3585911	0.8903631	-0.0691117	-0.1124251	-0.4917991	-0.0956484
0.07348025	-0.0776111	2.5163487	0.31313457	0.30565435	0.12761011	0.203516
0.23388127	0.47806596	-0.0935995	-0.3850096	-0.5281534	-0.3005573	-0.1554034
-0.5645546	-0.4838848	0.51263612	0.61072267	0.45782193	0.16310853	0.40069366

-0.0491445	-0.1482488	-0.9255911	0.22407731	0.05126527	0.03111661	0.05198692
0.28311554	0.33825408	-0.0998168	0.02500596	-0.0049532	0.20631895	0.16746159
-0.0230293	-0.3738596	0.17661592	0.16371078	0.10578962	0.3106006	0.30909649
-0.0159649	0.03964059	0.01080168	-0.1748492	-0.0326935	-0.1103694	-0.0112622
0.19053119	0.19953878	-0.1827305	-0.0562897	-0.0370771	-0.0980572	0.04108237
-0.1117659	-0.1453517	0.60392216	0.26922996	0.09290665	0.11002229	0.25795282
-0.1069568	-0.1143975	-0.0502593	0.11961376	0.13419116	-0.1590788	-0.0197886
-0.0873088	-0.1160283	0.01670399	0.02389937	0.03337741	-0.0086004	0.08520094
-0.1963406	-0.1320322	-0.1354371	-0.2312278	-0.2796526	-0.2259039	-0.3783962
-0.212685	-0.2891748	0.49227692	0.01048412	0.00433244	-0.0401677	-0.0835392
-0.2372143	-0.107885	0.2529429	-0.0949001	-0.228871	-0.1580909	-0.1520657
-0.8804654	-0.654032	-0.1877318	0.15497549	0.19157675	0.36592864	0.41911153
-0.201666	-0.0875098	0.2476827	0.03140199	0.01943644	-0.0108039	0.06305687
-0.0229973	-0.0262986	-0.078822	0.0027511	-0.003599	0.00649152	0.13813408
-0.1315019	-0.2695152	0.4267114	0.12845027	-0.0225032	-0.401931	0.234996
-0.4178843	0.04700058	-0.1475441	-0.3869088	-0.3735261	-0.3077134	-0.283697
0.31968123	0.23593046	0.00262338	-0.1968913	-0.2002559	-0.1447725	-0.1624479
-0.209572	-0.1283123	0.48812743	-0.0930724	-0.0922513	0.00436959	-0.0948072
-0.2432543	-0.4345601	-0.4807508	0.19664063	0.16199813	-0.129273	0.19736308
-0.0163127	0.03868577	0.59652469	-0.0555077	-0.0683825	-0.0690164	-0.0029583
-0.2289965	-0.2933037	-0.926717	0.54595639	0.50461594	0.28462485	0.32416821
-0.3357007	0.23156538	-0.881963	0.26944789	0.06769569	0.19513025	0.19806048
0.09653083	-0.0080496	0.05928414	-0.1153242	-0.0148572	0.09917291	0.00314755
-0.3226488	0.04986961	0.53341445	0.03636648	-0.0348388	-0.017978	-0.0980968
-0.0986923	0.01901599	-0.2551466	-0.0557494	-0.1107596	-0.1136432	-0.0706721
-0.1399686	-0.0836751	-0.6642044	-0.0471683	-0.0645223	0.04032489	0.1602481
-0.2008575	-0.2197725	-0.7607367	0.39895106	0.10388752	0.1673462	0.27877826
-0.1205854	-0.0879304	0.16222055	0.2198382	0.23787071	0.12300718	0.13095092
-0.1341999	-0.1565063	0.31182986	0.15481067	0.13537229	0.0272105	0.11898187
-0.7587827	-0.7801381	0.314695	-0.1555024	-0.1530325	-0.1760797	-0.181611
-0.6347014	-1.3772783	0.14872976	-0.0974933	0.15171132	-0.06454	-0.0484345
0.10951539	0.00643879	0.3469021	-0.0115162	0.01602723	-0.0677256	0.01531317
-0.1500371	-0.1971074	0.13705537	0.07813579	-0.0499284	-0.0547912	0.06676555
0.14085108	0.53701663	-0.6467885	-0.0442708	0.1203131	0.07690092	0.06051556
-0.0665512	-0.1671042	-0.1168632	-0.0459234	-0.1784314	-0.1444549	-0.0094153
0.09218842	0.17323284	-0.0952656	0.11198561	0.04067752	-0.0071032	0.02956149
-0.1389607	0.04931827	-0.2427259	0.45789581	0.37383729	0.37423478	0.20322734
0.26608396	0.23286868	0.46185148	-0.1011615	-0.0382034	-0.3698157	0.02959462
-0.2802981	-0.7925217	-0.5403405	1.0026043	0.78802999	0.82602391	0.86029914
0.0957148	-0.2962745	-0.0637053	-0.1818576	-0.1038117	-0.0828244	-0.1322786
-0.0422872	-0.0581841	0.37460495	0.2527395	0.18487971	0.00940234	0.0738121
-0.0078517	-0.2699749	0.06084028	0.38712144	0.04936331	0.01994634	0.25313367

-0.8606902	-0.3027865	-0.2573931	0.18188167	0.27384034	0.38237876	0.19689977
-0.4005302	-0.4081703	0.34652472	0.42156591	0.03635124	-0.0922173	0.22686656
-0.1260001	-0.3094569	0.37343705	0.26081606	0.00373628	-0.0532532	-0.0419352
-0.1387012	-0.0093174	0.12271356	-0.0332183	-0.0391286	-0.1283862	-0.0808252
0.01405773	0.10819833	0.01854743	0.19960164	0.13576057	0.09823064	0.12043655
0.00384048	-0.0110458	0.24553992	-0.2185602	-0.2510794	-0.3032686	-0.2459503
-0.1052966	-0.0295099	0.39469595	0.15731554	-0.0972425	-0.1576714	0.03702629
-0.0660027	-0.3730236	-0.3655181	-0.1120191	-0.304978	-0.4228268	-0.1320624
0.03883438	0.04616902	-0.0210709	0.16252023	-0.147224	-0.1211962	-0.1330155
-0.1278197	-0.2040032	-0.2103686	0.05071737	-0.0896653	-0.050065	0.10191598
0.123883	0.02220669	-0.0636715	-0.045115	-0.0433472	-0.0593963	-0.0705962
-0.0494031	-0.0703801	-0.114988	0.08326077	-0.0289408	-0.2799635	-0.0148057
-0.2708868	-0.4259292	0.48177628	0.02895743	0.09011513	-0.004427	0.09552971
-0.3101378	-0.4542211	-0.3671771	-0.0919118	-0.2420541	-0.2848303	-0.2303586
-0.2278568	-1.1206668	0.32237486	0.02865647	-0.0524001	-0.0479398	0.25431891
-0.3690224	-0.6228755	0.33960968	0.01645734	-0.2279877	-0.6772714	-0.2099777
0.21723914	0.05417644	-0.138199	0.12337733	-0.0614747	-0.2092508	0.02296428
0.21835274	0.33810128	-0.1177982	-0.1246095	-0.2428226	-0.2498737	-0.1248854
-0.6145569	-0.4430464	0.11743863	0.23360986	0.11091337	0.18454333	0.13503618
-0.0507359	-0.2706768	-0.1400707	0.0506646	-0.0257061	-0.1154535	0.00373168
0.04726609	-0.0405194	0.7338858	-0.0245183	-0.0469124	0.02500358	-0.0631018
-0.1305612	-0.0727581	0.59422988	0.28033045	0.18897574	0.26068156	0.08989787
-0.1802982	-0.0076101	0.04944008	0.56422375	0.57373658	0.48021372	0.62458106
0.01786909	-0.1155922	-0.1821915	0.15465131	-0.0798627	-0.0795394	0.01206023
0.08855484	-0.0032444	0.3620244	0.05761581	0.15931043	-0.0425016	0.06607008
-0.1067903	-0.6732275	0.35848324	0.75694851	0.65117427	0.7186909	0.7753229
-0.3295348	-1.4220846	0.8008017	0.81150582	0.73019487	0.64603559	0.61216932
-0.1296206	-0.5019178	0.37951577	-0.0849767	-0.2659582	-0.2921959	-0.266851
0.05813	0.032844	1.0272	-0.16715	-0.077645	0.079752	-0.11478
-0.39594	-0.8796	0.29683	-0.11481	-0.15009	-0.3249	-0.20648
-0.54146	-1.1668	0.0087456	-0.68364	-0.76423	-0.68308	-0.66149
-0.18011	-0.37475	1.4105	0.16928	-0.24409	-0.10209	0.14926
-0.25015	-0.19332	1.22	-0.043103	-0.12171	-0.16639	-0.014878
-0.22955	-0.14031	0.43578	-0.032635	-0.047374	-0.31757	-0.010705
-0.47705	-0.42718	0.86015	0.14862	0.13559	0.09985	0.283
-0.0137563	-0.213103	-0.1890352	-0.0060075	-0.0901379	-0.0524421	0.07655106
0.20277898	0.43277097	-0.2180288	0.10073752	0.13408655	0.08624568	0.05920025
-0.2237598	-0.2293749	-0.0882735	0.89313407	0.85022947	0.7160449	0.71906631
-0.1280828	-0.2815582	-0.0078104	-0.1132512	-0.1735515	-0.2806058	-0.1462497
-0.1556433	-0.2571172	-0.0050271	-0.0004055	-0.055296	-0.0083875	-0.0432298
0.01809211	0.13749198	-0.1935436	-0.4202329	-0.5840103	-0.5525839	-0.3445233
-0.3762086	-0.2162922	0.28489782	0.27033681	0.32223222	0.28044501	0.47658197

-0.3568799	-1.7087696	-0.0797121	-0.1937179	-0.0548955	-0.2795137	0.12324634
0.15491173	-0.1182862	0.01206464	-0.0182703	-0.0545121	-0.3459972	-0.1883245
0.14455309	0.03825379	0.20411493	-0.0604025	-0.0081387	-0.0594975	-0.138711
0.1967044	0.10754827	0.17621568	0.1468526	0.03280693	-0.1196876	0.07931171
0.12277206	-0.516269	-0.22155	0.0663352	0.02495464	0.00278316	0.23962736
-0.2171103	-0.5314885	-0.0043487	0.1567147	0.07988371	0.07305233	0.11390812
-0.0756897	0.0953889	0.83760259	0.16763458	0.13134154	0.06797174	0.20223097
0.08367807	-0.0284863	0.34796337	0.09914614	-0.116023	-0.0652568	0.04557809
-0.2493299	-0.2297725	0.11684678	0.49693998	0.38590606	0.25851522	0.33382952
-0.1451742	-0.3784976	0.05118609	0.1361587	0.10115197	0.13083087	0.07682934
-0.0212322	0.4260326	-1.5117937	0.30621833	0.40311592	0.43937788	0.53867859
-0.3406333	-0.5306484	-0.1942683	0.10585097	0.08579606	0.16570158	0.0598678
0.02711356	-0.3771532	0.34372081	0.05181763	0.15425364	0.29804647	0.38335358
0.09197811	-0.3253245	0.10125182	-0.0157814	-0.0276275	0.13570047	0.17555899
0.18968838	0.01226601	0.29372283	0.04452531	0.13783809	0.23384903	0.39563292
-0.0580973	-0.1809076	-0.3038464	0.01323897	-0.0104378	-0.1421902	-0.0468137
-0.1814358	-0.0360904	0.91266514	0.18263514	0.00038788	-0.0361834	0.04365261
-0.5100784	-1.3440776	0.68143974	0.12907463	0.09758474	0.17005847	0.09731975
-0.1252825	-0.0471904	0.8233872	0.09521747	0.01816419	0.05729606	0.08295047
-0.1903133	-0.1775572	0.71986707	0.02951642	0.07452295	0.09147622	0.01349118
-0.1352496	-0.3109242	-0.1540006	0.23917891	0.01129707	0.12931937	0.096266
0.1253334	0.00199592	0.05497187	-0.1339745	-0.0014472	0.03147954	-0.0725638
-0.038771	-0.0082691	0.65583	0.34991	0.15256	0.062103	0.39406
-0.2044346	0.49498917	2.7525445	0.27199915	0.37165423	0.23523176	0.33095294
0.12769899	-0.0974494	-0.2521514	0.07336479	0.01747903	0.12852665	0.07211297
-0.0457723	0.27221508	-0.2377085	-0.0876027	-0.2527783	-0.1005504	0.002326
-0.2290849	-0.3849451	0.18598368	0.09232761	0.04272869	-0.0817493	0.01749425
-0.0945084	0.13998677	0.58553734	0.54673629	0.53633209	0.75505982	0.36990689
0.1921875	-0.4264317	-0.085652	0.08591081	-0.0432933	0.07663579	0.15129841
0.43770201	0.25502449	-0.0306917	0.1609265	0.1076766	0.12334993	-0.0137399
0.17138705	-0.0952947	-0.0261379	-0.1922466	-0.1346637	-0.0611059	-0.0732094
0.30810144	-0.0837968	-0.1928947	0.26775331	0.20894084	0.19834167	0.16155619
0.05482354	-0.259507	-0.2128944	0.04281757	0.01213319	0.17375674	0.04920367
0.04995952	0.15318628	0.51894175	0.09172626	0.04909164	-0.0218877	0.04165223
0.04363237	0.2104926	-0.0993884	-0.0893436	-0.1139473	-0.2162363	-0.220205
0.12752711	0.00653049	-0.1896135	0.09673279	0.00714711	-0.0455885	0.05062501
-0.0429023	-0.4386552	0.64279911	0.19589407	0.0850669	0.15290628	0.13778335
-0.0993182	-0.0555276	-0.0927548	0.1302614	0.04239994	0.31588727	0.06024644
-0.0447686	-0.1160303	-0.2033889	-0.0995512	0.04023743	-0.0214756	-0.1277404
-0.0164432	-0.1970149	-0.5303471	0.05587519	-0.049429	-0.0124059	-0.093755
-0.0717464	0.14996774	0.30747286	0.02138779	-0.1138247	-0.035066	-0.0577311
0.0060448	-0.5656933	-0.0220976	1.3387389	0.90863393	1.0081177	0.92315794

0.18118693	0.16096508	0.18753845	0.23598253	0.16881688	0.27376632	0.35337807
-0.2162214	-0.1462855	0.11889845	0.0070365	-0.075262	0.09962931	-0.0893914
-0.039686	0.23767057	0.3199419	-0.3628152	-0.1627054	-0.2112283	-0.1936239
0.26122754	0.08554413	-0.1339679	0.49231666	0.30914954	0.26667134	0.30412835
0.04983854	-0.1065602	-0.0977919	-0.1637112	-0.0866389	-0.1810003	-0.02778
0.08900619	0.06139804	0.27908679	0.10229787	0.10474989	-0.025247	0.12757424
-0.0407039	-0.0951348	-0.2421565	0.02195649	-0.0101665	0.01372864	-0.0948858
0.15710111	0.11032741	0.92493025	0.00655225	-0.0561498	-0.0164954	-0.1552171
0.13266235	-0.0841783	0.08911366	0.41079515	0.40083752	0.48776563	0.19674352
-0.0971431	-0.0546175	0.49920761	0.31077096	0.22519489	0.27644221	0.13012144
0.11159413	0.31803758	-0.2122667	-0.0638218	0.09303713	0.0758732	-0.195312
0.01776373	0.2066699	0.04312417	-0.175967	-0.2249987	-0.305553	-0.3137706
-0.2921206	-0.4632376	0.24461295	0.1805968	0.05882347	0.16900157	0.07943461
-0.0756856	-0.0175061	0.81978148	-0.0009584	-0.0146696	0.24157545	0.00215083
0.3343036	0.56193199	-0.4089582	0.24498241	0.32951946	0.57421527	0.16193487
-0.3052314	-0.2282785	0.92003955	0.07776928	0.03117449	-0.109986	0.06146162
-0.0563806	0.478334	0.43776848	-0.0719478	-0.2764955	-0.2499533	-0.3300898
0.2486686	0.09185832	0.39584	0.07618157	-0.0655194	-0.0329124	-0.0682254
0.04908983	-0.0855543	-0.0108928	0.32717484	0.16312178	0.25533346	0.0849607
-0.1083244	-0.2169646	-0.3101298	0.20614936	0.05782755	0.04298111	0.08419919
-0.3674476	-0.1697977	0.10892481	0.11326226	-0.0780585	-0.0811375	-0.0876254
-0.083179	0.08814456	0.09838639	0.11386377	0.026003	0.03087458	-0.0793189
-0.1088659	-0.30512	0.31900456	0.01738233	-0.0569647	-0.0486805	-0.0433746
0.1424168	0.10522522	-0.1667116	0.08140813	0.08582038	0.06834432	0.067224
0.35636641	0.33307404	-0.0064179	0.04767321	0.196942	0.24029131	0.12895075
-0.1169727	0.08733014	0.15158866	0.04405948	-0.0088084	0.11999145	-0.0652504
-0.1392417	-0.2398894	0.31076633	0.08618203	0.07349606	0.03668747	-0.0596663
-0.2555912	-0.2622479	-0.3718952	0.36320078	0.23065137	0.24562867	0.29701732
-0.1267335	-0.5363422	-0.6041593	0.16121833	0.04808923	0.05958247	0.14590381
0.32697472	0.04732057	0.19451642	0.37404888	0.35644794	0.569677	0.33357608
0.29280887	0.21360768	0.45129807	0.06681313	0.02138753	0.05532968	0.03700197
-0.2228707	-0.2230015	0.34917971	0.35323803	0.27291794	0.3476554	0.33307574
-0.1512569	-0.1909529	0.12503275	0.18313836	0.12932565	0.16726342	0.04570259
-0.1854576	-0.0933181	0.38904963	0.02568597	0.0726348	0.0301223	0.0137616
-0.046741	0.15069581	-0.1181939	0.35404944	0.13178759	0.34344371	0.3224202
0.02158376	-0.1660358	0.11015883	0.39943939	0.15898687	0.10561843	0.19590462
-0.2412714	-0.4622901	0.23646796	0.02025938	0.0634878	0.10285092	0.08581789
-0.0031803	0.02657742	-0.4535302	0.0534572	-0.0319342	-0.2645006	0.12605901
-0.2325441	-0.8879477	-0.5814316	0.00237895	-0.0333293	0.08068984	0.01060821
-0.0831902	0.21819388	-0.0342989	0.07322348	-0.1167044	0.04392444	-0.0108969
-0.1453885	-0.1559256	2.3919083	0.40858166	-0.0035866	0.07272748	0.15762537
-0.1212515	-0.1184474	2.6530918	0.50836598	0.181727	0.26588938	0.31281109

0.32516551	0.22168307	0.23806821	0.0308182	-0.0056186	0.03016894	-0.062704
0.06776358	0.09391847	0.60567823	0.03679094	0.01500799	0.25366699	0.00823929
-0.2935675	-0.2040944	0.0768591	0.09544296	0.05968791	-0.0007001	0.05012949
-0.3255326	-0.3365501	-0.0336034	0.08494995	-0.0323494	-0.1244679	0.02874622
-0.1024827	0.07404252	0.69011665	0.10155516	0.04841506	0.14017332	-0.0288972
-0.0327141	0.1496989	0.33737184	-0.076314	0.05014443	-0.0209356	-0.0407706
-0.1527287	-0.2161861	-0.0325701	0.3468888	0.23448491	0.27767133	0.29191633
-0.0588561	-0.0060017	0.54285321	-0.0815221	-0.0046575	0.25377741	0.02566368
-0.1118163	-0.0775707	0.03228115	-0.0189856	-0.0178265	0.38837312	-0.0024374
-0.1169017	-0.0634636	0.91109985	0.50197603	0.5584174	0.52692433	0.50271865
-0.3853311	-0.7413588	0.24670507	-0.0406076	-0.0100696	0.17497679	-0.0052272
-0.202802	-0.3990076	-0.3802038	0.022664	0.06153255	0.06096564	0.12133104
-0.2547964	-0.3266068	-0.0630759	-0.0290346	-0.0956471	-0.0042633	-0.0640331
-0.2804016	-0.2597295	0.06803608	0.27142146	0.17140691	0.37526157	0.19139309
-0.4626191	-0.1453725	-0.0465531	-0.0487696	-0.1474226	-0.1242974	-0.182902
-0.4106953	-0.2807198	0.12870027	-0.1260319	-0.130961	0.15145055	-0.1011871
0.19414196	-0.3474846	-0.3324801	0.23280381	0.17876285	0.35792572	0.22480214
0.10436099	-0.1642516	0.85736603	0.36138471	0.36030442	0.37194488	0.40427873
-0.2078377	-0.1197607	-0.0829716	0.21168424	0.14892202	0.10668948	0.26212885
-0.1103655	-0.3699833	-0.6231526	0.04116696	-0.0130209	-0.0175762	0.15544684
-0.1968194	-0.1678656	-0.0525533	0.02636901	0.19745195	0.06933453	0.14574609
0.15746448	0.22498699	-0.2951006	-0.1608231	0.03030764	0.00435669	-0.0656793
-0.1097066	-0.0748099	-0.1917821	-0.2275647	-0.1004636	-0.0549307	0.00744194
-0.1263501	0.08141667	0.15375922	0.21594117	0.1593295	0.1074434	0.22990753
0.03970145	-0.0234089	-0.1196275	0.15715326	0.00307923	-0.0766004	0.26581628
0.56412037	0.57657909	-0.1702529	0.56512148	0.67889345	0.70672457	0.53210183
-0.1403485	-0.1935431	0.0960474	0.05695986	0.11734392	0.07047107	-0.0867787
-0.126251	-0.2194975	-1.6103564	0.08977407	0.09322544	0.15046313	0.05490893
-0.2104339	-0.2645147	-0.1758334	0.40918497	0.26624996	0.4317436	0.35517007
-0.107	-0.2170872	-0.3618579	0.69112498	0.37729783	0.4137047	0.40777309
-0.0302884	-0.0304025	0.53573711	0.17142897	0.1876028	0.56333097	0.00933538
0.31858077	0.3162746	0.12150057	0.31099444	0.2758943	0.39072851	0.22448685
-0.0380049	-0.1988657	0.8003645	0.23838296	0.12573243	0.49768296	0.20712055
-0.2421366	-0.2729601	3.3100187	-0.1621293	-0.0536354	0.05576776	-0.1391565
-0.2790849	-0.3727704	0.02528682	0.19982228	0.02574315	0.24429186	0.12814213
-0.25533	-0.4970665	-0.1783664	0.14311068	-0.0131826	0.0425883	0.18412426
-0.1288874	-0.1342198	0.14479578	0.0690209	0.11685618	0.08486817	-0.03823
-0.2751387	-0.2156992	0.28110127	0.11031454	-0.0469991	0.07812703	0.00939499
-0.0495653	-1.4462418	-0.4847736	0.24268271	0.6776668	0.82201758	0.96241231
-0.0625398	0.22006051	0.34570048	-0.4061299	-0.3592055	-0.3296926	-0.3342918
0.21790831	0.16557878	0.11178215	-0.1497979	-0.1120056	0.02974961	-0.0598918
-0.0972048	-0.1182777	0.43246924	0.11166105	-0.015889	0.19521169	0.1707305

0.21155055	0.06450435	0.14785554	0.04346057	0.04862189	0.33444548	0.08705564
-0.11748	0.01125	-0.12301	-0.16237	-0.046223	0.043301	-0.044366
0.3510987	0.91316657	-0.3237424	-0.443115	-0.2858119	-0.3206076	-0.2708197
0.00988281	-0.0560685	0.4707258	0.09932019	0.22304628	0.23676609	0.23403249
-0.2384119	-0.1108887	0.35242185	0.27410837	0.06449057	0.0917609	0.03474196
-0.215838	-0.1057087	0.83415115	0.26523509	0.3969917	0.49311995	0.42521097
-0.3799993	-0.8669249	0.12891612	0.13799065	0.08798961	0.15429692	0.17785297
-0.26611	-0.72999	0.25268	0.82254	0.65946	0.67246	0.69287
0.00916469	0.14958453	0.24961524	-0.0494993	0.06852043	0.14694346	0.0074522
0.39484404	0.60010221	0.03257818	0.37949705	0.25613061	0.28780471	0.2113972
0.50483769	0.54079733	0.14023777	-0.0279393	-0.0624351	0.00736773	-0.0143322
-0.0865372	-0.2691066	-0.0525905	0.1393282	0.0320145	0.17033636	0.31837588
-0.1198138	-0.1400355	-0.313205	-0.2256595	-0.2147928	-0.196856	-0.3215248
0.18829951	0.20304954	0.00222137	-0.199014	-0.0965031	0.19904275	-0.0297888
0.30932121	0.25825737	0.1169873	0.07143487	-0.0612811	0.02473937	-0.0117937
0.17891954	0.13592944	0.29460222	0.17424678	0.05265396	0.34279469	0.14929513
-0.3695132	0.40918791	-0.0509548	0.12221194	0.12345377	-0.0260601	0.068625
0.05968921	0.14636715	0.02363633	-0.2977738	-0.291525	-0.1390267	-0.199239
0.17170629	0.41462138	0.62596711	-0.2792043	-0.1394072	-0.218064	-0.0916356
0.1049179	-0.1636262	-0.4863853	0.45155506	0.25301149	0.3314413	0.33066967
-0.1167593	-0.1814676	0.44236813	-0.2462126	-0.1534988	-0.1931626	-0.2218006
-0.4098806	0.06328441	0.43560471	0.31653394	0.49771524	0.54693597	0.48266815
-0.0566134	-0.0797014	0.64341595	0.15343752	0.15411998	0.48227353	0.17107135
-0.473234	0.49991391	-0.7329703	0.11346125	0.11512368	0.18648682	0.04440191
0.11256294	0.32195118	0.04669729	-0.0948187	0.12142356	0.27803366	-0.0566863
-0.1666667	-0.422104	0.47970007	-0.1275047	-0.0741959	-0.0059334	-0.0329863
-0.2682874	-0.1759762	0.5041441	0.03851846	0.1556139	0.13840613	0.18036246
-0.41934	-0.3003745	0.30136322	0.20637201	0.17638883	0.19460416	-0.0349974
0.02364629	-0.0732163	0.37319734	0.51471989	0.42778184	0.40060022	0.34958142
-0.0252676	0.21605467	0.79965341	0.17638819	0.16209317	0.40831647	0.16319171
0.06588769	0.02398931	-0.0463678	0.32011129	0.31408458	0.40888357	0.33309071
0.19741066	-0.0158228	-0.363643	0.13471889	0.18104047	0.08961268	0.31119523
-0.0265914	-0.0605843	0.31641854	0.06289551	0.00225679	0.04768061	-0.0180911
0.0289723	-0.1494797	-0.1865385	0.66656482	0.43959724	0.60060896	0.45152004
-0.0428655	-0.402501	-0.6688916	0.09531178	0.10549422	0.30929445	0.16416767
0.03143904	0.10037169	-0.3097023	0.19422705	-0.0644059	-0.0225753	0.09633629
0.05950088	-0.0259873	0.06573352	0.36454329	0.0130166	0.22752645	0.18652277
0.42237681	-0.0272619	-0.2626968	-0.031813	-0.0853539	0.00489074	-0.1164477
0.17655339	0.37739952	0.09460541	0.34991027	0.44989305	0.41577447	0.45034025
-0.0196732	0.09703924	-0.8192328	-0.0583504	0.03168928	0.00912769	-0.0201739
-0.0900844	0.13671819	0.22783123	0.20934454	0.22144406	0.16329929	0.1789948
0.32144076	0.36296618	0.41902534	0.15501977	0.14994797	0.26035686	0.12521579

-0.3072617	-0.244271	-0.3820471	0.20384448	0.07838107	0.0150893	0.09887653
0.2155855	0.22720404	-0.2540398	-0.0352184	-0.0995989	-0.2200156	-0.0029519
0.15587554	0.06689263	-0.0501083	0.01217938	-0.0932034	-0.1186968	0.00522426
0.3045013	0.41338937	-0.5341207	-0.3368671	-0.3542789	-0.516419	-0.1856391
0.01241146	-0.0863555	-0.3726435	0.28355541	0.19250143	0.16998443	-0.0240178
-0.3890533	-0.1345566	0.16141797	0.41888706	0.1605308	0.14904743	0.22857367
-0.0957987	-0.0984658	0.11104067	0.03109373	-0.0472833	0.00833438	-0.0626456
-0.0350171	-0.1855768	0.10042897	0.10643104	0.04420401	0.2172025	0.0103056
-0.0296641	0.13713481	0.31514834	0.13791095	-0.1288059	-0.044932	0.01677385
-0.2163206	-0.2136824	-0.1579988	0.17488169	0.11524013	0.18425321	0.092052
-0.0404246	0.01741299	0.23999162	0.01477114	-0.007354	0.00347448	-0.0072058
0.07986726	-0.0576653	0.43225387	0.24214671	0.17727426	0.47756383	0.38474372
0.00665871	-0.114737	0.54623008	0.08567703	-0.030679	0.02623247	-0.062566
-0.4900556	-0.3981074	0.68111537	0.3181502	0.05426917	0.27770611	0.07928615
0.12195008	0.24523787	-0.122717	0.08239633	-0.0880152	-0.1164622	-0.005331
0.20485892	-0.0716314	0.06415286	0.30102173	0.00126392	0.09056463	-0.0023234
0.2154442	-0.0314051	0.4301025	0.03335761	-0.0062387	-0.0186432	-0.0345643
-0.0053717	0.07248675	-0.1717718	0.14035717	-0.0599284	-0.0558039	0.12982724
0.16320673	0.31170958	0.08640755	0.01145459	-0.2108415	-0.2654547	-0.0633637
0.07118119	-0.1574589	-0.0144061	0.19483178	0.3572436	0.26518221	0.18765977

test_GSE477	test_GSE477	test_GSE477	test_GSE477	test_GSE477	test_GSE555	test_GSE642
ref_GSE4778	ref_GSE4778	ref_GSE4778	ref_GSE4778	ref_GSE4778	ref_GSE5552	ref_GSE6425
GSE4778	GSE4778	GSE4778	GSE4778	GSE4778	GSE5552	GSE6425
GEO	GEO	GEO	GEO	GEO	GEO	GEO
affymetrix	affymetrix	affymetrix	affymetrix	affymetrix	affymetrix	affymetrix
TIME:5	TIME:5	TIME:5	TIME:5	TIME:10	INDOLE:0.5,	INDOLE_TIME
1294	1295	1296	1297	1298	1305	1309
-0.3594636	-0.0050605	-0.1237101	-0.1458077	-0.1488388	0.48937151	-0.1703905
0.01535498	0.32397412	0.16914608	0.00405223	0.0565441	-0.0597519	0.03813275
0.02682885	0.16149238	0.07905187	0.19529202	0.1662822	-0.0939831	-0.0610374
0.00087192	0.04371012	-0.0494068	0.06811723	-0.1560405	-0.108561	-0.1380753
-0.0348733	-0.0060993	-0.2690003	-0.0698413	0.01702072	0.0507847	-0.073968
-0.1952095	-0.1070277	-0.1631394	-0.1238045	0.13479297	-0.1221223	0.03824042
-0.6790314	-0.3950626	-0.5747561	-0.4958306	-0.1676758	-0.1260589	-0.0242019
0.60664667	0.61224109	0.47282958	0.73488497	0.63314014	0.0947993	-0.0715956
-0.0678238	0.094895	-0.0290747	-0.1483671	0.02107952	0.29321595	-0.0010525
0.67430019	0.72151317	0.61791593	0.80498259	0.05629856	0.10441203	-0.1802267
0.05190935	0.31578529	0.33881984	0.34804462	-0.0285579	-0.0864574	-0.277765
2.1087889	2.1195497	2.074885	1.8546324	0.91636543	-0.3789454	0.42154291
0.08280903	0.18406251	-0.038795	0.05352831	0.09432403	-0.0253115	0.0019894
-0.2533494	-0.2720969	-0.4498165	-0.4248348	-0.2666901	-0.073757	-0.0662255
-0.0288299	0.04415712	-0.0136888	-0.017349	-0.0979749	-0.7622709	-0.1068308
0.19469803	0.27757123	0.26334416	0.15506851	0.17276076	0.04704809	0.08716429
0.90923517	0.72927617	0.65716556	0.543644	-0.3281279	0.11275	-0.0888584
-0.8762389	-0.8469033	-0.863886	-1.0026861	-0.9424658	-0.0150433	0.09410935
0.12516027	0.09970913	0.09582397	-0.1011244	0.00882819	-0.4682734	-0.0359411
0.06724236	-0.001165	0.11754655	0.06582496	0.02875644	-0.0832406	0.23125547
0.15321151	0.03659564	-0.0707598	0.01633425	-0.1256226	0.22021779	-0.0022603
-0.1670546	-0.0620758	-0.1689025	-0.1520023	-0.0772106	-0.25589	0.03064314
0.4899887	0.55089977	0.36987246	0.28975295	0.39599652	0.21435069	0.28793839
0.18956011	0.2134289	0.18074669	-0.0316769	0.24930522	0.033295	0.02366477
-0.3128232	-0.1862326	-0.3030609	-0.2927892	-0.0132164	-1.1809134	0.1850189
-0.0909623	-0.1128623	-0.3053817	-0.0956784	-0.2620127	-0.0975086	-0.0088711
1.000675	1.0192227	0.88351312	0.88974913	0.63970929	0.39194	0.03912673
-0.0967506	0.03732798	0.11829046	0.10467418	-0.0557707	0.2071851	0.11236697
-0.0571802	-0.0206628	-0.1062926	-0.022707	-0.0702273	0.014131	-0.1659399
0.4137606	0.14416317	0.05048145	0.20733933	0.25711357	-0.7367354	0.14423396
-0.4753735	-0.2365308	-0.3160224	-0.2287119	-0.4889937	-0.03951	-0.2401774
0.71292308	0.54393855	0.56127724	0.82689012	0.10513469	-0.2258595	0.0501233

0.17027874	0.04803428	0.0122207	0.01503999	0.10572461	-0.4378909	0.12961785
0.09034461	0.11505777	-0.0420885	-0.0472012	0.08941934	0.05649857	0.16477855
0.4535731	0.36260325	0.50630589	0.33569142	0.13654581	0.10126659	0.31083336
-0.0589652	-0.0368647	-0.1444118	-0.0662077	-0.1483387	0.31085131	0.12754754
-0.0666662	0.04148081	-0.0393477	-0.0607955	-0.0214054	0.04252493	0.03594487
0.43924018	0.49384061	0.3757667	0.45992148	0.29262446	-0.0198241	0.01038179
0.10282823	0.22167892	0.12190532	0.12820389	0.11588622	-0.0257775	0.06408625
0.18138195	0.12655226	0.08815175	0.01328038	0.04003939	-0.1578304	0.08311064
-0.2259198	-0.231218	-0.2218043	-0.1782691	-0.112267	0.12529607	-0.2071135
0.04193327	0.0112828	0.03159389	0.03604347	-0.000781	0.03710646	0.10560421
-0.1469487	0.01023766	-0.1025216	-0.167988	-0.0757345	-0.0215679	-0.1147552
-0.4295173	-0.0315377	-0.0944788	0.02850339	-0.4234517	0.04947015	0.31716201
0.03386959	0.07432801	0.04674897	0.07961418	-0.0453143	-0.109232	-0.0348336
0.08329003	-0.0267699	0.06571246	0.12085929	0.03948687	0.12908233	0.02469209
0.05707412	0.21610478	0.07974633	0.21536618	0.24122595	0.1047043	0.01189468
-0.4943145	-0.6110028	-0.6636687	-0.6408361	-0.4415319	-0.2745532	-0.1043847
-0.1195819	-0.0576694	-0.1783524	-0.1479133	0.02319078	-0.1493282	0.17023362
-0.1420199	-0.1770939	-0.2158776	-0.0597675	-0.4220858	-0.0772186	-0.0981518
0.15087883	0.18453312	0.16901699	0.28960893	-0.0500493	-0.0725129	-0.2229133
0.03499658	0.01477207	-0.0087344	-0.068002	0.0767171	0.05085264	0.08705006
0.4244788	0.36131533	0.39341692	0.30993598	0.13339013	0.28208528	-0.2355875
0.31789357	0.18780139	0.19358123	0.48788941	0.54806336	0.15786389	0.02974914
-0.2182043	-0.1435766	-0.2353259	-0.2282201	-0.1742089	-0.0855602	0.18506242
0.0691892	0.26931762	0.13586496	0.11705493	0.1090186	-0.0222678	0.01414529
-0.1451575	-0.0768445	-0.0422415	-0.0946731	-0.1013336	0.05313148	-0.0769211
0.0825759	0.21812018	0.2607068	0.36850204	0.2288674	0.5238356	0.15838627
1.1633654	1.1809205	1.0066368	1.3807742	0.86315186	0.01330597	-0.0590771
0.16789776	0.20839656	0.24997302	0.18233265	0.08109081	0.057487	0.07371846
0.48299034	0.33609626	0.33708105	0.40125242	0.36445756	0.2596128	-0.0539689
-0.1978761	-0.1024292	-0.110915	-0.2938874	-0.1089749	-0.906649	-0.1314806
0.10297491	0.04540335	0.07428571	-0.1123563	-0.0038695	-1.4954592	-0.039956
-0.0738612	-0.0912633	-0.1019988	-0.1771217	-0.1644129	0.05474367	0.0225326
0.18121314	0.31375034	0.25193124	0.28864064	0.06028512	0.10435999	-0.0616726
-0.7011679	-0.7123071	-0.7960548	-0.7340228	-0.7223912	0.18448649	0.1886568
-0.1365766	0.02635814	-0.2494649	-0.1090111	-0.2923346	-0.5628485	0.10517963
0.2074459	0.12494905	0.06662941	0.1085924	-0.084621	-0.1360223	-0.1693413
1.158643	1.0677409	1.1365182	1.1564844	0.81029425	0.09331526	0.10626725
-0.116672	0.00765259	-0.1127766	-0.1141911	0.01454889	-0.0242163	0.10906923
1.0210672	1.1973515	1.0160239	1.1010593	0.8266687	-0.0598151	-0.2890112
-0.4431943	-0.25027	-0.4134752	-0.3869673	-0.1523711	0.24035753	0.07233361
0.59585348	0.4575302	0.44456204	0.56088895	-0.0397966	0.08286299	-0.0427524
0.4969384	0.45605154	0.27458003	0.41259706	0.13719666	-0.2498185	-0.0710692

0.81270336	0.76815338	0.73485716	0.88240604	-0.0039292	0.11732645	0.17884545
0.47115555	0.54728751	0.43737765	0.67085918	-0.069817	-0.2748141	-0.1827615
0.01616936	-0.0129529	-0.0407498	-0.0374413	-0.1962358	-0.2588673	0.10403418
0.4408746	0.51762695	0.43363616	0.55691766	0.08265305	0.18876345	0.01198202
0.26002445	0.36824843	0.37165078	0.38910856	-0.0216852	0.03694372	-0.0195225
-0.2102584	-0.2154246	-0.1113086	-0.150407	0.11690373	-0.0121711	-0.081442
0.03815939	0.01444966	0.04966574	0.05312984	0.12351554	0.26747521	-0.0892276
-0.2858957	-0.2410324	-0.1629177	-0.2490155	-0.1282291	-0.0626913	-0.2502019
-0.0477532	-0.0661917	-0.0349877	0.05097189	-0.0345959	-0.1878213	-0.2399165
-0.2105858	-0.2306999	-0.319899	-0.2672554	-0.0449513	0.18082592	0.06620664
0.06193888	0.08377644	0.03708875	-0.0008254	0.07187944	-0.1612982	0.07767265
0.11666259	0.25477429	0.17724977	0.13057103	0.18135922	0.00527924	0.20747857
0.10038766	0.26900479	0.16799598	0.19188491	0.17850823	0.08792694	-0.1376122
0.08315583	0.25983077	0.21768052	0.1544445	0.29803119	-0.091533	-0.1218585
0.62123438	0.82389289	0.77563211	0.87695286	0.5777987	-0.17917	-0.0076315
-0.2984638	-0.1323721	-0.302956	-0.3554032	-0.2350372	-0.0747588	-0.0766724
0.05826031	0.07508377	0.05286737	0.10314856	0.24207683	-0.1289984	-0.0925319
-0.2522045	-0.266994	-0.2914836	-0.0886201	-0.1343396	-0.218391	0.02504589
0.82079023	0.94475119	0.79738435	0.97657258	1.2980893	-0.2237	0.12072547
-0.05186	-0.1498524	-0.2346739	-0.16516	-0.2253313	0.10578392	0.07010676
-0.0311801	0.0521006	-0.008737	-0.1172298	-0.0188052	-0.0498023	0.13776708
0.39996471	0.40486433	0.45584067	0.28830017	-0.0293389	-0.25003	-0.0467873
0.56340932	0.43293602	0.45937618	0.49461925	-0.1098043	-0.0536243	-0.1805889
0.19461669	0.20632234	0.12705314	0.28462136	0.32825407	-0.1269612	-0.0100364
0.01433663	0.17185333	0.06429314	0.11259428	0.01256732	-0.0462325	0.13321993
0.93199465	0.64660463	0.71348495	0.897092	0.64724267	-0.1341214	-0.2194429
1.6161939	1.3806296	1.340586	1.3297143	1.0822698	-0.1269343	-0.1540785
-0.2778065	-0.2245049	-0.2243875	-0.2599967	-0.2665893	-0.2444454	-0.1955385
-0.075681	-0.11876	-0.12676	-0.059815	-0.062962	-0.15561	0.11908
-0.24771	-0.19462	-0.26757	-0.29961	-0.24311	0.033953	0.10829
-0.72414	-0.7111	-0.61702	-0.62245	-0.33443	0.019005	-0.065501
0.04782	0.41603	0.32406	0.2989	0.040397	0.085527	-0.16912
0.083736	0.20606	0.19447	0.18435	0.032795	0.072428	-0.18294
-0.26383	-0.098606	-0.35212	-0.22526	-0.27204	-0.0023057	-0.01756
0.031327	0.086162	-0.052135	0.14292	0.044644	-0.037407	0.18398
0.08577959	0.35167231	0.34256016	0.29880374	0.34517116	0.07664553	-0.1872239
0.18564969	0.19173876	0.12216392	0.1052445	0.08403854	0.11268733	-0.033841
0.77547734	0.76536361	0.78144428	0.84676743	0.8596626	-0.3849873	-0.1837185
-0.2282135	-0.1654491	-0.2596804	-0.2758119	-0.1452319	-0.5372648	-0.2324456
0.04610274	-0.0433565	-0.1882024	-0.0893965	-0.0014229	-0.0687505	0.08715369
-0.4874261	-0.549549	-0.5478452	-0.4323194	-0.550311	-0.7168726	-0.1079139
0.0421174	0.24900433	0.24880451	0.35206438	-0.4726915	-0.3911018	-0.0157963

0.33935862	0.17220581	0.13959768	0.41096893	0.44148625	0.03020549	-0.0204594
-0.6639901	-0.7396091	-0.7303342	-0.7131523	-0.4044438	0.16119492	-0.0121171
0.07949784	0.0690572	0.00488227	0.03677689	-0.112628	-0.0107451	0.12514078
0.14679314	0.14698029	0.2236544	0.15650882	0.18200135	-0.0073029	-0.1265521
0.85467473	0.74943994	0.79315091	0.83972225	0.44665075	-0.1138036	-0.0900226
0.02013977	0.06439853	0.07524497	0.00454289	-0.2088216	0.29410464	0.09978075
0.22393659	0.14557524	0.17059276	0.22131325	-0.1471428	-0.1876349	0.11829714
0.12449115	-0.0688934	0.03766624	0.19025513	0.00837592	0.01035958	-0.0289515
0.33618765	0.5227501	0.43415339	0.40465106	0.19180255	-0.0044946	-0.1055636
0.26308893	0.39428818	0.28652615	0.34890978	0.47835746	0.19969333	-0.0792295
1.0396554	1.2054928	1.1537762	1.3041261	0.99221494	0.13613316	-0.0603432
-0.0375413	-0.0085224	-0.0677441	-0.0414071	-0.004105	0.0127082	-0.1007895
-0.1913292	0.13795899	0.09064167	0.09563606	0.36692339	-0.2085473	-0.1137938
-0.4499199	-0.2415031	-0.2126725	-0.2833926	0.06873239	0.12798634	-0.1900184
-0.5816698	-0.3903428	-0.2286941	-0.159621	0.02828672	-0.3460938	0.00556411
0.33375975	0.28489342	0.24197946	0.047877	0.25734395	-0.360446	0.11981186
-0.0465004	0.1867857	0.06666575	0.01766416	-0.3685558	-0.0079483	-0.2181558
1.343405	1.6573566	1.6301157	1.2516173	1.6957724	-0.2904113	-0.3390595
0.15552414	0.30343412	0.26493991	0.29829056	-0.0051754	-0.5663414	-0.2139793
0.17406614	0.14555969	0.06838867	0.18598178	-0.002295	-0.3129463	0.04408858
0.26171385	0.16794776	0.24520325	0.19713932	0.16911904	-0.5336421	0.06178988
-0.0066435	-0.0138445	0.03060073	0.0672194	-0.0882381	-0.2835755	-0.0926014
0.15191	0.12383	0.054985	0.19606	0.036544	0.0027753	-0.0005716
0.02433052	-0.0122812	0.10157366	0.09050831	-0.0144396	0.02674511	0.23149972
0.47130586	0.30313401	0.35400944	0.31659346	0.15481725	-0.3973	-0.2507687
0.22845901	-0.077789	-0.0352434	0.19385551	-0.3265345	-0.0789146	-0.1983158
0.11236385	0.06007603	0.07701077	0.18204151	0.10408587	-0.3012216	-0.1542924
0.49319823	0.17749102	0.12412513	0.21671584	-0.1391151	-0.3354192	0.27264655
-0.2826215	-0.2013493	-0.0993035	-0.112269	0.08798011	-0.2003789	-0.0931297
0.25419908	0.2618731	0.36145661	0.08430888	0.31452584	-0.12048	0.23881883
-0.0837109	-0.2095578	-0.0878529	-0.1366935	0.0666055	0.06385289	-0.0004842
0.5897694	0.6625718	0.66070819	0.57266597	0.17144442	-0.0193875	-0.0045203
0.12109663	0.00336543	0.05150358	0.0477775	0.05550735	0.10481057	0.11429323
0.08097253	0.16173092	0.17494763	0.2467324	0.12778145	-0.1662679	-0.0255607
-0.5647067	-0.585596	-0.6368346	-0.516301	-0.4963769	-0.1243937	-0.1667882
-0.0256037	-0.0875704	-0.2265059	-0.1213972	-0.0485809	0.13177415	0.25388909
0.61321297	0.41411699	0.46997411	0.48743374	0.29552123	-0.4555755	0.1060389
0.17456998	0.1215993	0.0447759	0.04973974	0.00529577	-0.0385774	-0.2369478
-0.14288	-0.2049532	-0.0939643	-0.1343461	-0.063264	-0.0323785	0.05527295
-0.0546698	-0.121924	-0.1258323	-0.0794321	-0.1276383	-0.5051106	0.27148659
-0.0482546	-0.0261252	0.03578161	-0.0856254	-0.1046655	0.03519023	-0.2616544
1.3891095	1.3796591	1.3439144	1.4720361	0.88307743	-0.1808636	-0.2344834

0.27457286	0.27533725	0.16464112	0.19626753	0.15220489	-0.0851761	0.16349601
0.06825827	-0.0379772	0.00571153	0.02695757	-0.0768983	-0.0674775	-0.0556937
-0.1542266	-0.2238397	-0.3721739	-0.2770595	-0.2717958	0.04265472	0.00903292
0.1193976	0.09421601	0.13996667	0.1890946	-0.268966	-0.0586699	0.1809398
-0.0985039	-0.2817972	-0.1352921	-0.1760484	-0.2665348	-0.0486426	0.11214539
0.10267765	-0.0280729	-0.0831616	-0.0326056	-0.1114335	0.03023356	-0.0812502
-0.1038788	-0.2035167	-0.1589191	-0.2999181	-0.0308765	-0.2772119	-0.0675143
0.04751544	-0.0810224	-0.1708343	0.13622666	-0.1036074	-0.1506211	-0.0253379
0.49093719	0.72843915	0.6794722	0.76300206	0.54197885	0.06435813	0.22273059
0.34268429	0.4625471	0.39979547	0.49088133	0.22737243	0.08792017	-0.1844255
0.07671352	0.04142687	0.04211635	0.0362921	0.09687483	-0.1114563	-0.0047093
-0.4001078	-0.3101522	-0.4381687	-0.3196556	-0.2374976	-0.2423723	-0.1071764
0.05405088	0.12701549	0.21429078	0.0725122	-0.0542558	-0.0186676	-0.0534797
-0.0132064	-0.0162017	-0.0258551	0.07637705	-0.0975486	0.01770388	0.11869166
0.26403738	0.03772805	0.14310618	-0.0122034	-0.0281048	0.00049322	-0.0488526
0.0530209	0.06878068	0.31313592	0.12025184	0.19233989	-0.2506809	-0.0542231
-0.7219015	-0.8632103	-0.8475993	-0.7877828	-0.9005608	-0.1655958	0.06534878
0.07244869	0.17946861	0.22837681	0.19228333	-0.037356	0.23508279	0.12888278
0.63720475	0.55239312	0.55404219	0.41868148	0.25848987	0.09134048	0.06509826
0.3619359	0.47483131	0.51818355	0.49596873	0.6039719	-0.0159729	-0.3831693
-0.0203852	-0.0113174	0.06879914	0.09222357	0.06985065	0.01956076	-0.2995361
0.27610194	0.33684962	0.23577977	0.26241073	0.11973834	0.13650229	0.01821973
-0.0300438	-5.60E-05	-0.0323217	0.04470696	-0.0306315	0.18624436	-0.0581863
0.01600517	-0.0145779	0.00775903	0.03846103	0.00239732	0.04517763	0.17749793
0.00250937	0.05273739	0.05871184	0.1024976	0.2046842	0.09749739	0.11460414
0.12653152	0.06353104	-0.0086984	-0.0143399	-0.175216	0.0370821	0.17943512
0.768727	0.4045339	0.54474718	0.54148367	0.28686189	-0.106994	-0.0676606
0.50285191	0.59270574	0.67726863	0.72222079	0.2366578	-0.0062294	-0.1762933
0.25378813	0.21234419	0.23261265	0.28315653	0.16750722	-0.6676649	-0.044008
0.5338707	0.61381706	0.45298298	0.44024193	0.14176169	0.16850712	0.0505179
0.03881528	0.14966678	0.13770374	0.12627677	0.13064315	-0.0927846	-0.0193365
0.24776777	0.26397142	0.2121395	0.43815412	0.00936417	-0.0489701	-0.0834071
0.27184928	0.19268433	0.26675509	0.26317041	0.34503742	-0.0838591	-0.2670615
0.08211278	0.06968452	0.04805196	-0.0120713	0.05204022	-0.0312699	-0.1198861
0.3360191	0.42350361	0.35113226	0.35884782	0.49155323	0.02422847	0.01613652
0.28774863	0.55772274	0.56566166	0.63906293	0.28911939	-0.1667198	-0.0618484
-0.0323425	0.05666461	0.00270958	0.08842525	0.25851701	-0.83996	-0.1478154
-0.1522671	-0.0418651	-0.0310901	-0.0309926	0.03344362	0.03699617	-0.4052207
0.99196529	0.98569954	0.98211648	1.012644	0.712432	-0.0655337	-0.0462721
0.05809435	0.09978597	-0.0237024	0.13493421	-0.0360233	-0.056544	0.08342636
0.32756902	0.42584669	0.39101669	0.47959498	-0.0191439	-0.0352067	0.01920027
0.58925143	0.45030919	0.4689266	0.62193992	-0.1039958	-0.0054175	-0.2344707

0.10066468	0.19670482	0.13134665	0.12348734	-0.0164288	0.21888396	0.24704583
0.05968519	0.02224588	0.12014948	0.01039314	0.02992699	0.08188465	-0.0025999
0.39393144	0.25925008	0.21021364	0.2510481	0.15151188	-0.0552562	-0.2710184
0.33834771	0.33826638	0.41728056	0.55772903	-0.0011008	-0.2059285	-0.107288
0.19771551	0.02564356	0.21902784	0.1271496	-0.1622216	-0.0567772	-0.079958
-0.0830034	-0.2037462	-0.1161322	-0.1932471	-0.0987481	-0.259577	-0.2607152
0.5670629	0.46056336	0.55243561	0.65074994	0.17929192	0.06003506	-0.2871536
-0.0919575	-0.11701	-0.0731672	-0.1768065	-0.1881144	0.0360312	0.38318436
0.00620327	-0.1033835	-0.0174666	0.01476364	-0.0258343	0.25657984	0.01531234
0.38929191	0.44431885	0.32160417	0.34484365	-0.0447768	-0.20791	-0.0349922
-0.0364843	-0.1649296	-0.1175897	-0.0937121	-0.1659035	-0.0098222	0.05679433
0.12411892	0.08547075	0.02955031	0.22168931	-0.1096212	-0.1929682	-0.429988
-0.6413695	-0.5079772	-0.4850478	-0.4421284	-0.2183804	-0.192001	-0.1559354
0.25572997	0.33498726	0.24444145	0.3853472	-0.0079894	-0.0809515	-0.0771701
-1.6101638	-1.5917022	-1.421715	-1.4585975	-1.9271212	-0.0476491	-0.0976139
-0.1197305	-0.1226092	-0.1314166	-0.0931308	-0.106492	0.22328407	0.04343847
0.54146494	0.61379305	0.73283202	0.87584703	0.83651593	-0.2432836	0.08448594
0.68656873	0.63020033	0.71013456	0.73785633	0.32510211	-0.305624	-0.1338291
-0.0173685	-0.0596198	0.05657275	0.05740244	-0.0541583	-0.0961845	-0.2078013
0.26056122	0.55611473	0.42279607	0.53101988	0.64147165	-0.0945717	-0.1550118
-0.2096977	-0.0393174	0.05793328	-0.0571546	0.25921907	-5.56E-05	-0.4162016
-0.1045752	-0.1396207	-0.1110939	-0.0604717	-0.0329094	-0.002532	-0.0302581
-0.0306333	0.09544881	0.04090317	0.0569664	0.05513665	-0.0459964	0.03748164
0.03788178	0.00092472	-0.1092753	-0.0754182	0.08649332	-0.1005016	0.18738744
0.25027812	0.17963397	0.1346822	0.34891892	0.08950327	0.18324857	0.05349746
0.24732165	0.14553626	0.14218034	0.25319915	0.15338429	-0.0489424	0.16142309
0.10131307	-0.0390839	0.04415972	-0.0717013	0.04904897	0.64205664	-0.0884107
0.75366817	0.69449306	0.60203237	0.69151463	1.0061772	-0.2907664	-0.2366183
1.0360789	0.88088991	1.0097644	1.0586158	0.94526834	-0.5673891	-0.0324436
1.1977169	1.0641581	1.0631337	1.2622022	1.1061902	-0.0375858	-0.001903
0.11161977	0.16645935	0.28539434	0.04625612	0.20875105	0.20126051	-0.0044221
0.49427927	0.39036579	0.42501319	0.41110222	-0.4569609	-0.0674451	-0.1419779
0.73028105	0.85126181	0.93640205	0.77540243	1.9891161	0.46207775	-0.2382986
-0.9296459	-0.9760512	-0.8291578	-0.7341554	-0.4496877	-0.1575734	0.02772939
0.13610538	0.35923123	0.43740728	0.56748803	0.1971704	-0.4409864	-0.1436027
0.10072293	0.10105626	0.16048775	0.16556937	-0.0242108	0.05307042	-0.1555184
0.19671424	0.07134096	0.11371081	0.16943191	0.02036486	-0.2284185	-0.0001841
0.08566962	0.22088078	0.30077866	0.31156569	0.36920487	0.24948032	-0.0233559
2.0145822	2.4118428	2.4082119	2.4152126	3.0365116	-0.4364101	-0.2222333
-0.4416867	-0.3731004	-0.3371487	-0.3483681	-0.4213919	-0.08763	0.12034578
-0.4622013	-0.4041591	-0.3867615	-0.3818858	-0.09691	-0.0143145	0.21249199
0.05458692	0.03278226	0.19319554	0.09720586	-0.0824439	0.02057229	0.10664866

0.09716245	-0.0162153	0.03922232	0.09883419	-0.0994655	0.13271163	-0.1038793
0.003909	-0.054718	-0.0006525	-0.0007372	-0.18761	0.055531	-0.11358
-0.9291858	-0.7437534	-0.746148	-0.7449452	0.02248522	0.04936204	0.10703774
0.06626131	0.04443917	0.12773462	0.15677436	0.19895576	-0.0120779	-0.0156559
-0.1584519	-0.1343434	-0.1312424	-0.1026984	-0.3677955	0.10137803	0.05214056
0.24209788	0.17808323	0.33964246	0.15583153	0.74260964	-0.0588274	-0.0513616
0.36949409	0.34858738	0.38186464	0.41228552	-0.0216338	0.11022992	-0.2673694
1.507	1.2961	1.2683	1.3726	0.32051	-0.086604	0.03675
0.04912541	-0.1081123	-0.0281412	-0.0333025	-0.2355123	0.03597826	0.0483423
0.10547112	0.13850222	0.13781283	0.10927282	0.16893758	0.030408	0.24512571
0.13554391	0.11709118	0.13011311	0.07527945	0.18030559	-0.6258846	0.08774617
-0.1303178	-0.0722106	0.03536963	0.16184473	0.03104482	-0.0969867	-0.0999292
-0.2752533	-0.2254696	-0.2048599	-0.3476243	-0.087338	0.16624533	-0.2074057
-0.1555819	-0.0915389	-0.0564161	0.03846283	-0.0348719	0.05290626	-0.0101794
-0.1113813	-0.1003647	-0.0570116	-0.0446031	0.12880227	-0.0310205	0.11220483
0.09818706	0.02198797	0.24430265	0.30189729	0.23642449	-0.1733324	-0.062502
0.04027869	-0.0230864	0.06986099	-0.0757015	-0.2171877	0.04389968	-0.1941669
-0.1484557	-0.0153975	0.1045131	0.08849548	0.20925263	-0.2215643	-0.2056225
-0.7046225	-0.5309092	-0.5113045	-0.5331378	-0.3176102	0.20575619	0.16821266
0.44794722	0.30498419	0.43652465	0.40743312	0.17361202	-0.1906561	-0.179296
-0.1702871	-0.1694576	-0.028498	-0.044257	-0.2678635	0.0783909	-0.1837905
0.48135815	0.55720074	0.55226729	0.51999073	0.28474172	0.12498802	0.0376025
0.12675022	0.07532731	0.0994452	0.08406402	-0.1035133	0.071337	-0.0178988
0.92753133	0.85715227	0.88463767	0.96764793	0.42738133	-0.1190041	0.08367509
-0.0833569	-0.1203336	-0.0366645	-0.2173626	-0.1291693	-0.2657202	0.1928918
0.14278351	-0.1318783	0.02965941	0.06118475	0.06494319	0.13370575	0.00423051
0.0881706	-0.0190167	0.04754433	0.19414361	-0.2197389	0.05448213	0.0650802
0.19723194	0.22837504	0.18614589	0.04761705	-0.035283	0.78074	-0.188956
1.0264505	0.90429467	0.73669079	0.79780099	0.19202855	0.25982479	-0.2873398
0.16838701	0.14950971	0.24844123	0.14681203	0.27440007	0.1487327	-0.0366222
0.28563142	0.28849479	0.33055521	0.43853753	0.20887909	0.13639603	-0.2234803
0.07988543	0.15183539	0.17992558	0.15584485	0.32861991	-0.0239807	-0.0180686
0.21293486	0.19918562	0.20326767	0.04946112	0.21665	-0.27351	-0.0160448
0.79821107	0.60694399	0.71111709	0.80301015	0.57165685	-0.2780423	-0.3422459
0.18647472	0.25435682	0.26111692	0.30264507	0.2904062	-0.2191096	-0.1997622
0.19844167	0.09404608	0.19450416	0.21906233	0.12801753	-0.099386	-0.3257203
0.27171708	0.33697148	0.46735848	0.34143254	0.4058492	-0.2703352	-0.2056908
0.98944429	0.99777569	1.0010142	0.9303926	0.88347465	0.05734665	0.03684147
0.22493502	0.14739113	0.20162374	0.21423746	0.15829477	0.17667669	-0.0346408
0.19405697	0.23791595	0.18140106	0.13720825	0.22301917	-0.1398895	-0.0415359
0.16147882	0.19579353	0.17488693	0.26164706	-0.2716136	0.23026976	-0.0994696
-8.34E-05	0.04361647	0.08733501	0.17279947	0.17599668	0.34145729	0.23863845

0.06030241	-0.0149362	0.10277634	0.18151518	-0.0035267	-0.03142	-0.013621
-0.0574514	-0.1170613	-0.1648856	0.00968991	-0.1194081	-0.6956885	-0.1637989
0.15631514	0.15116614	0.16955893	0.30274284	0.10726155	-0.3000633	-0.133241
0.07389843	0.1364863	0.22662568	0.28112336	0.14180914	0.00982133	-0.2330078
0.50124293	0.4642632	0.58320638	0.30845776	0.26853943	-0.073025	0.0783767
0.19069686	0.25556921	0.28539508	0.17664648	0.13772946	-0.0373129	-0.2043377
-0.0132568	0.0895643	0.22693119	0.08773543	0.0640752	-0.1391449	-0.1363098
0.13705644	0.03858867	0.12388215	0.17016	0.06589673	-0.0878979	-0.1025284
-0.0846751	-0.0657476	-0.0020579	0.0893386	0.18266199	0.25921045	0.02237477
-0.0858587	-0.0378511	0.00968578	-0.1227386	0.09493544	-0.014149	-0.103698
0.31692472	0.25483115	0.23565729	0.37226159	0.04458246	-0.1300718	-0.0769432
0.37237875	0.41876045	0.52234577	0.67300337	0.45421579	-0.3456761	-0.3377349
-0.0728245	-0.0679108	-0.0096434	-0.3798932	-0.1009977	0.0077024	0.00209827
0.14462609	-0.0765257	0.04850118	-0.1153418	0.03508724	0.082199	-0.005589
0.04749965	-0.0075696	-0.0678446	0.05880089	-0.276166	0.00805081	-0.0646849
0.19259011	0.27239166	0.39359287	0.30043395	0.21979383	0.2901122	0.0484404
-0.0766725	0.0048221	-0.0227414	-0.0094757	-0.0155428	0.11513227	-0.1688095
0.13011074	0.38253803	0.31248872	0.53394576	0.25690322	-0.1022392	-0.1731685
-0.1816365	-0.0067291	0.14884679	0.31619837	0.09393939	-0.0883078	-0.2874508
0.84583147	0.86079315	0.77402241	0.81953851	0.50264413	0.23621381	-0.0576495

test_GSE642	test_GSE642	test_GSE683	test_GSE683	test_GSE683	ref_GSE6836	test_GSE683
ref_GSE6425	ref_GSE6425	ref_GSE6836	ref_GSE6836	ref_GSE6836	ref_GSE6836	ref_GSE6836
GSE6425	GSE6425	GSE6836	GSE6836	GSE6836	GSE6836	GSE6836
GEO	GEO	GEO	GEO	GEO	GEO	GEO
affymetrix	affymetrix	affymetrix	affymetrix	affymetrix	affymetrix	affymetrix
MG1655:-1,l	EVO_OXYGEI	dinP_b0231:	dinP_b0231:	lon_b0439:+	NORFLOXACI	recA_b2699:
1320	1332	1364	1365	1370	1373	1380
-0.4149445	0.04543537	0.14245607	-0.1087654	0.07381642	0.4222156	-0.2262669
-0.7574098	3.2677273	-0.0111084	0.05376608	0.06251921	0.33158877	0.01744064
-0.907744	0.22607109	-0.3099134	-0.0544217	0.22423762	1.0924925	0.37462045
-0.4070194	0.01467498	-0.105761	0.01867323	0.01086183	0.17119031	0.02258371
0.06868355	-0.0948457	-0.2142912	-0.154707	-0.0971752	0.20379966	-0.0946101
-0.4508763	1.0275318	-0.2579444	-0.3228145	-0.1176117	0.38308106	-0.2047217
-0.7354701	-0.2802504	-0.027389	-0.0144646	-0.1031214	0.71798922	0.01284916
-0.4199181	0.76271224	0.21655214	0.11040729	0.14687737	0.61135613	-0.08883
-0.345195	0.17640936	-0.0864735	0.01097671	0.08235899	0.32211902	0.09914282
-0.0623745	0.3601466	-0.0315707	0.05424185	0.14239395	0.13404433	-0.0320001
-0.5473194	0.05824681	-0.0447677	-0.0180267	0.07595753	1.3050422	-1.0960193
0.37919871	0.3783454	-0.0783293	0.05644915	0.31705329	0.42837614	-0.0150789
0.27963797	-0.274368	0.04398409	-0.0726343	0.05107627	0.22459183	0.0408575
-0.2849713	-0.2237999	0.11996429	0.07414181	0.02202735	0.03767659	-0.1095374
0.13865275	0.00663727	0.03541697	-0.0338237	0.03342563	-0.0445242	0.07353616
0.17720322	0.12693938	-0.136596	-0.0506772	0.01707337	0.17216256	-0.0110642
-0.1527716	-0.3031905	-0.1945183	0.0623053	0.06067597	-0.0389468	-0.1890245
1.3152375	-0.4986105	-0.2321905	-0.2202659	0.02900211	-0.0296089	-0.1147998
-0.2795207	0.07932183	-0.1713966	-0.0470626	-0.0675028	0.23150787	-0.2233375
0.40725084	0.10603185	0.0817387	0.10498458	-0.0006173	-0.1997453	0.1809197
-0.0322877	0.0275161	-0.2122831	-0.2350731	-0.2611648	0.08182765	-0.0797825
0.17048145	-0.1318236	0.07769112	0.02144703	-0.0067528	0.10218434	-0.0636381
0.53980231	-0.0324892	-0.2325675	-0.1514634	-5.36E-05	0.31992328	-0.0378615
0.05888109	0.05490296	-0.0494847	-0.0296027	-0.0701637	0.33536759	-0.0713044
0.47961309	0.15326192	-0.0844833	-0.0368361	0.00242972	-0.0780477	-0.2158593
0.20747808	-0.0866319	0.18400914	-0.0684274	-0.0039796	0.6157982	-0.1315677
0.49568304	0.60610624	0.14821004	0.1829409	0.18939206	0.17044108	-0.0541487
-0.0099538	0.16973613	0.00069762	0.25150463	0.30780643	-0.1076635	0.08692285
-0.3297523	-0.2776695	0.05905186	-0.1014216	-0.0353582	-0.0675849	-0.1963917
-0.122043	0.49245838	-0.4107936	-0.4470758	0.16193308	0.26917484	-0.2008601
-0.6642831	-0.0220633	-0.2412027	-0.1252652	0.12087211	0.16202989	-0.2288123
0.20935332	0.29480907	-0.0974202	-0.0350457	0.25424347	0.2589915	-0.1128077

-0.3322069	-0.0502503	-0.2171397	-0.2857845	-0.1469073	-0.0490868	-0.1154071
0.12535091	0.09231016	-0.1453202	-0.0295806	-0.0790459	0.21801871	-0.1125021
0.03203627	0.52109401	-0.0879072	-0.1538339	0.14675099	0.28497349	-0.2977146
0.02721089	0.00529744	-0.0502026	-0.086326	-0.0967848	0.10040275	-0.0905148
-0.0489071	-0.1889251	-0.2780934	-0.0566183	-0.1227648	0.17256927	-0.0947303
-0.2847358	-0.0806719	-0.0211234	-0.0080037	-0.0047037	0.1911931	-0.2080496
-0.0043039	0.02444985	-0.031991	-0.0771595	-0.0871021	0.16698195	-0.011954
0.46729205	0.02332919	-0.0246355	0.07341641	0.00613939	0.10567356	0.11279425
0.02560141	-0.4361341	-0.11116	-0.0797174	-0.0814827	-0.0062954	-0.0508383
-0.0882263	-0.0502195	-0.0598772	0.01854936	-0.0694971	0.08206862	-0.0612174
0.31878863	0.04508388	0.04168847	0.0265231	-0.0335789	-0.1652038	-0.0485477
0.09575051	0.30850609	-0.1350006	0.09728774	0.35239286	0.03706379	0.2060665
-0.0551829	-0.1224694	0.03685766	0.06789722	-0.0333996	-0.2788466	0.04578594
-0.1278864	0.33347624	-0.1389035	0.01985309	-0.0234739	0.11291193	-0.121933
0.12870334	-0.1627125	0.0090135	-0.0688885	0.03417723	-0.286314	0.15412241
0.30306918	-0.527039	0.31668375	0.04047438	0.06252752	-0.8853858	0.36549787
0.34581655	0.00341009	-0.0326676	-0.063265	0.02883057	-0.2156059	0.04192044
-0.4166112	-0.0968306	-0.0709284	-0.2029306	0.03480263	-0.021118	-0.0254679
-0.4808812	-0.4399376	-0.062965	-0.1167125	-0.0762973	-0.1134082	-0.2067504
0.01489319	-0.0435372	0.00040912	0.0829186	-0.1189772	-0.01888	0.12667753
-0.816354	0.11210512	-0.0850117	0.07039688	-0.0406915	0.17103954	-0.2725629
-0.1580129	0.11262816	0.03800475	0.05229944	0.026302	0.05657199	0.09746191
0.26268448	0.23157899	-0.0845967	0.14011204	0.02773311	-0.0034272	0.05893341
0.06719701	0.04538181	-0.1516497	-0.1130672	-0.1158337	-0.0171906	-0.2001678
0.01301615	0.06335795	-0.1012731	-0.0933804	-0.0066526	0.26862237	-0.0584977
-1.0103347	0.08573328	-0.1017872	0.04165816	0.36111107	0.44401667	0.06514785
-0.2244019	0.87290963	-0.0757047	-0.0859728	0.18517253	0.01635495	-0.0264882
0.0674971	0.27928932	-0.0064185	0.07134264	-0.0388756	-0.0200074	-0.0403012
0.01843004	-0.0285011	-0.0833081	0.11163176	0.1096943	-0.5975565	0.03684752
-0.2248954	-0.2095058	-0.1363457	-0.2133512	0.05469178	0.17205531	-0.4520247
-0.2816817	-0.000822	-0.3247187	-0.2874919	-0.1565791	0.14231974	-0.4028202
-0.1307985	0.20988267	0.11160357	0.08567039	0.17683806	-0.0095086	0.03812076
-0.3380533	-0.1599376	-0.0198946	-0.0214562	0.05242994	-0.0527537	-0.0978084
0.32651479	0.02814819	-0.1199589	0.06418854	0.0518801	0.03082963	0.00827504
0.14338196	0.10953069	-0.0146339	-0.0055433	-0.0043562	0.01019395	-0.0208777
-0.3430028	-0.2472868	0.15527768	0.11165749	0.05301916	0.03110368	-0.0589145
-0.5293344	0.40804718	-0.143976	-0.2611071	0.02465798	-0.1342374	-0.0999345
-0.2464912	-0.0746626	0.03729559	0.00126646	-0.1048325	0.27890736	0.15007658
-0.6506016	0.29920318	-0.4403336	-0.0399479	0.05725063	-0.2353063	-0.4364454
0.28559754	0.04042955	-0.1282308	-0.0897393	-0.1435218	-0.1022583	-0.0784171
-0.2432669	0.06687385	-0.1245582	-0.0881687	0.14360936	0.12014505	-0.2068734
0.89564848	0.08634028	-0.0296959	0.00992995	-0.163006	0.1512856	-0.3210253

0.12230526	0.0977649	-0.3874701	-0.2461101	0.27265481	-0.2414182	0.02990779
-0.2466043	-0.1463153	-0.2055971	-0.1238992	0.3248229	-0.4772971	-0.2807981
-0.1332812	-0.0155893	-0.1815344	-0.0525065	0.19484543	-0.2394205	-0.007961
-0.0452229	-0.0767791	0.04529057	0.1286222	0.19340657	-0.1056773	0.11642684
-0.0846859	0.07401847	-0.0023787	-0.0853369	-0.1299855	0.38141098	-0.1128282
-0.1470315	0.01230035	0.00519823	-0.009421	0.14647603	0.25689416	-0.0252657
-0.3331089	-0.2693694	-0.0510639	-0.0111991	0.03557654	0.08762906	0.0438524
-0.3569415	0.1044467	-0.2621489	-0.2798683	0.0566532	-0.3623219	-0.0830152
-0.4142588	0.32678264	-0.0755918	-0.0156308	0.0321013	-0.3079295	-0.0311596
-0.3208419	-0.1314337	0.2703057	0.11411448	0.07836718	0.0962051	0.18422251
0.30212519	0.09721542	0.07356653	0.06667756	-0.0577762	0.10369603	-0.0227581
0.10357705	0.17725023	0.00406236	-0.0764524	-0.0722112	0.00642309	-0.0249889
0.2802516	-0.1611283	-0.0449042	0.02382239	0.09294841	0.20716847	-0.0227245
-0.3744826	-0.0081621	-0.3569629	-0.0664614	0.14736828	0.25494266	0.03497776
0.09138459	-0.1477246	-0.4073928	-0.1921738	0.27676708	0.52797966	-0.0754581
-0.4608922	-0.3422724	-0.1534829	-0.0621206	-0.0447496	-0.139422	-0.1845833
-1.1599111	0.16935684	-0.0429823	-0.0214844	0.01999501	0.0015975	-0.0012229
0.06012418	-0.0251244	-0.0113492	-0.0401341	-0.0166906	-0.0240071	-0.1004698
-0.0080962	0.09619085	0.14963598	0.01974645	-0.011479	-0.0938134	-0.041842
-0.0883662	0.16507819	4.97E-06	-0.0790028	0.0611127	-0.2294499	-0.1691851
0.1045567	-0.0082193	0.04207055	0.01078518	0.13756591	0.06665099	0.07531775
-0.1046626	0.62797927	-0.017221	-0.0201938	0.17163896	0.16441567	0.02756772
-0.9387912	0.35520551	-0.0972234	0.07122099	0.18885743	0.20672452	-0.0001102
-0.2229426	0.27712537	-0.0307177	0.0468722	0.06170291	0.28368483	0.14857931
0.55532762	-0.085459	-0.0209474	-0.1675004	-0.0410846	0.21985703	-0.017605
-0.8795913	-1.0049863	-0.2184683	-0.0176974	-0.0560078	-0.0809965	-0.0688182
-0.7870372	-1.3561921	-0.1011513	0.11070298	0.06436846	-0.7190132	-0.018557
-0.512256	-0.3607666	-0.1218357	-0.1985821	-0.0742484	-0.3650589	-0.3406872
0.1746	0.061088	-0.042482	-0.090996	0.12258	0.64371	-0.15153
0.22396	0.1339	-0.25567	-0.085177	-0.025973	-0.089974	-0.37666
-0.090398	-0.21762	-0.052794	-0.045847	0.036909	-0.51822	-0.39499
-1.063	-0.10493	-0.12237	-0.14806	0.12544	0.27419	-0.42544
-1.3851	-0.2328	-0.045122	-0.08839	0.030482	0.36633	-0.26725
-0.0012209	0.059079	-0.076621	0.088382	0.067567	0.29395	0.015348
-1.5707	-0.35971	0.15955	0.2342	0.28647	0.026349	-0.082613
-0.3612001	0.14603104	-0.2072251	-0.0678892	-0.021436	0.63630806	-0.0976295
-0.1647531	0.13888881	-0.101352	-0.0444806	0.02752893	0.03901245	-0.0226762
-0.4100636	-0.249016	-0.1719225	-0.0321518	0.12643081	-0.1931402	-0.1757708
-0.4197212	0.24547418	-0.0817021	-0.2355626	-0.0966391	0.26596186	-0.1866902
-0.2253557	0.46009856	-0.1627893	-0.1356689	-0.0495142	0.08693673	-0.098602
0.67093041	0.09692221	-0.3331881	-0.2274902	-0.0707875	-0.4779025	-0.0544361
0.54462672	0.11832577	-0.0861424	0.14312145	0.12560032	0.00045234	-0.1282815

0.12498161	0.01971886	-0.1684785	-0.1143506	0.07398789	-0.3125312	0.01079209
0.44791937	-0.4776634	0.18958086	0.0712185	0.03064572	-0.8222644	0.29306169
-0.0154941	0.14608575	-0.2313301	-0.0575852	-0.0906995	0.22694074	-0.1250779
-0.3624153	0.09976655	0.056189	-0.0422667	-0.014101	-0.0757214	0.09029452
0.03440276	-0.2024507	-0.0955357	-0.0973741	-0.1163451	-0.0125957	0.11234871
0.11612368	0.37210525	0.0125577	0.03532473	0.07896193	-0.3594654	-0.1662461
0.22396432	0.18484707	-0.0514853	0.00217289	0.11119849	0.05150682	0.01597304
-0.1405823	0.06263187	-0.1330105	0.00404942	0.05844805	0.3129416	0.00473162
-0.0636963	0.02571345	0.00253659	0.18144708	0.05984745	0.15781189	-0.1872102
-0.458888	0.6382536	-0.3708655	-0.2778603	0.04634574	0.73118985	-0.252373
0.19867258	0.26675544	-0.0595021	-0.1770203	0.15145583	-0.0286962	-0.1474835
-0.3970376	0.24646801	-0.2554035	-0.0837428	0.07460726	-0.2961725	-0.2222191
-0.6306213	0.21920371	-0.7727558	-0.3510211	-0.1177746	1.6943156	-0.3480796
-1.3010566	-0.4759281	-0.1600328	-0.0183616	0.05539551	0.41273747	-0.0088414
-1.4430163	-0.6301382	-0.2870928	-0.0663097	0.12470652	0.38858923	0.01819909
0.41321738	0.31019303	0.01374841	-0.1759416	0.04409955	-0.194112	-0.0509953
-0.3049212	0.85979922	-0.2447973	-0.1979523	0.15113573	0.21811803	-0.1728343
-1.023379	-0.955777	-0.1848311	-0.1228725	-0.0055393	-0.053987	-0.1167631
-0.4161856	0.01431874	-0.2397137	-0.2034108	0.14412348	0.25663257	-0.3133451
-0.0636906	-0.1064033	0.09088619	-0.0370769	0.06345718	-0.0107157	-0.0855033
0.33860935	-0.030071	-0.0652099	-0.0456337	0.08089935	0.03145719	-0.0200527
-0.0306992	0.07152591	0.05487685	0.12067383	0.01709449	-0.08617	-0.2648667
-0.22366	-0.0090443	-0.12986	-0.18054	-0.033026	0.13159	-0.143
0.48571241	0.20998168	0.00416014	-0.3240278	0.17165175	0.13730075	-0.2808337
0.01411103	0.28663439	0.00323168	0.04688706	-0.2047781	-0.4559526	-0.1929176
-0.9450805	-0.4942728	0.11297938	0.07373054	0.05142804	-0.0931664	-0.0344028
-0.4742197	-0.0037421	-0.1652154	-0.0689926	0.03483527	-0.0038876	-0.2326812
0.26354881	0.06756394	-0.0002448	-0.0438567	0.09092625	0.20595095	0.1376825
0.08633632	0.26825164	-0.453864	-0.3470298	-0.1758581	0.70377327	-0.3577797
-0.0090102	0.16559469	0.06729209	-0.0187466	0.08463006	-0.1939372	0.03095365
0.30077327	0.22485416	-0.2300733	-0.166732	-0.0682718	0.75906087	-0.1506239
-0.0824716	0.51611389	0.02941749	-0.032624	-0.0268508	0.06662936	-0.0820755
-0.0459695	0.16035114	-0.0753438	0.0620187	-0.0263257	-0.3135096	-0.0699244
0.08421301	0.15114813	0.01707535	-0.0647571	0.08341655	0.10158133	-0.0136631
-1.308471	-0.6288481	0.10635875	0.07023655	0.06881451	-0.2373446	0.16989993
0.09415679	-0.0017296	-0.1251485	0.04751846	0.04148267	-0.149442	0.02377971
-0.1246589	0.40016519	-0.1120788	0.22122034	0.19448374	-0.1263774	-0.0133113
0.13074781	0.21308723	-0.1010033	-0.0541293	-0.0429702	0.03131858	-0.109553
0.12279522	0.19824834	0.06082682	0.06654635	0.04167693	-0.0612223	0.09846806
-0.2102163	0.18272258	-0.3991495	-0.3228727	-0.2014123	-0.0497532	-0.2654145
-0.2645005	0.32170504	-0.0807886	0.07317222	0.0074382	0.08274214	-0.0571315
-1.5090518	0.07910887	-0.3098063	-0.23633	-0.1590911	0.25466395	-0.2687044

0.41818029	0.26561206	0.04622182	-0.0036445	0.09829902	0.01579311	0.00205775
-0.0559105	0.18476508	0.02028645	-0.014739	-0.0135239	-0.242646	-0.0437902
0.40261605	0.17434093	-0.0015307	0.05401982	-0.0147523	0.01961467	0.09887277
0.3117538	0.39301966	-0.32525	-0.1874517	-0.0642819	0.32920593	0.08955951
0.59629587	0.02991638	0.2017869	0.15319093	0.10159325	-0.1880503	0.07654121
0.22410334	0.08151424	0.10422937	0.05732884	-0.0190651	0.06753325	0.13177993
-0.1259406	0.49579675	0.06120713	-0.08489	-0.1110897	-0.0475186	-0.044742
0.05384808	0.12169828	0.17883318	0.38766704	0.39086494	-0.2297811	0.40266028
0.20245233	0.29592278	-0.3376316	-0.2453049	-0.1327577	0.55030848	-0.1604504
-0.1475339	0.16146468	0.09129835	-0.0306288	0.05296224	0.04007919	0.0346221
0.2456355	0.11461699	0.03249636	0.11480386	-0.0039759	-0.3505063	0.04604814
-0.2850953	0.13003001	-0.1026087	-0.0708572	0.05216306	0.15019334	0.0273985
-0.2520236	0.04154874	0.06547434	0.03482488	0.09833676	-0.0213427	0.10272325
0.04202387	-0.1110363	0.19282775	0.1796343	0.04014302	0.08638542	0.15725424
0.1842844	0.02448868	0.20109622	0.09357474	-0.0439739	-0.2058871	0.01197088
-0.0422331	0.21675238	-0.1485946	-0.0653273	-0.0719943	-0.0709174	-0.220613
1.2664131	0.4260201	-0.2957108	-0.1841459	-0.284559	-0.1737978	-0.1914215
0.50828235	0.19672721	0.20304906	-0.0532251	0.13487239	-0.2248318	-0.1356624
0.23424144	0.20035165	-0.1530555	0.10474089	0.12583187	-0.0215526	-0.0972514
-0.5908508	2.6253575	-0.4349509	-0.2588892	0.1371939	-0.6182151	-0.4144322
-0.3647969	1.3084743	-0.2982192	-0.132501	-0.0010334	0.40108276	-0.217163
0.22326569	0.34899029	0.23111728	0.13991718	-0.0060212	-0.0195972	0.06742235
-0.1332096	0.11653621	-0.0770017	-0.0266304	-0.120924	0.19655109	0.03131229
0.1385405	0.78336855	-0.045328	-0.0396644	-0.0244101	0.08675393	-0.0702886
0.4794273	0.02060721	-0.0076292	-0.0544786	-0.0536961	0.27851683	0.11372947
0.12112063	0.0147694	-0.0620819	-0.0171507	-0.0857054	0.21281052	-0.1933493
0.11504468	0.35130527	0.01893742	0.06368612	0.10845464	-0.0396614	-0.1848263
-0.7770764	0.35449204	-0.2769213	-0.0842485	0.17923073	0.79080708	-0.1199741
0.00224317	0.22352766	-0.0100642	0.15322288	0.04889626	-0.1118837	0.10437223
-0.0228717	1.3654342	0.08145443	0.01344946	0.20967407	-0.0747394	0.09941966
0.24418997	0.36306856	-0.1631246	-0.1305269	-0.0039949	0.51110088	-0.1116521
0.08012137	-0.0698987	0.20981788	0.04049699	-0.0012371	-0.0128514	0.16027037
-0.7280858	-0.0947826	0.00548297	0.04046101	0.0442489	0.17973986	0.04182158
0.13875431	-0.0892703	-0.0344953	0.13153522	-0.0108149	-0.0575453	0.06441726
-0.0333311	1.2987982	-0.3070904	-0.083441	-0.1084752	0.82378525	-0.1943617
0.16642774	0.48946727	-0.0052881	0.00552378	0.05164953	0.1826002	-0.0856213
-0.0655059	0.72033843	-0.0392183	-0.0972559	0.07403444	0.07087433	-0.1032978
-0.9175125	0.06162955	-0.0538232	0.14104986	-0.0203382	-0.0769522	-0.2789395
-0.3572586	0.05022637	-0.4262803	-0.1741569	0.14107987	0.1831741	-0.0557469
-0.1949957	0.28822412	0.0138768	0.02469503	0.0554897	0.12933441	0.03457445
-0.5515079	0.39734421	-0.0511622	0.00076602	0.30427561	0.180531	-0.0999638
-0.6665961	0.26837425	-0.1606211	-0.1026344	0.20139327	-0.0861681	-0.3710142

0.51096749	0.36286518	0.01825401	0.00743683	0.11911891	0.29902744	0.1377947
0.48913497	0.08926281	0.14931196	0.06932315	0.0980302	-0.1595747	0.05385474
-0.2299286	-0.2960205	0.0914295	0.10454624	0.17623114	-0.3194377	0.08797544
-4.97E-05	0.09896758	-0.170925	-0.0189696	-0.0461659	0.07893384	0.00136924
-0.229199	-0.0998068	0.06988393	0.1602836	-0.1291697	0.08032462	0.11765967
-0.7521322	0.30149604	0.00928617	0.06685097	0.01718443	0.61800752	0.04403508
-0.0473143	0.11349622	0.1609471	-0.0215223	0.27156753	0.05612212	0.11968219
-1.0641002	1.9528797	-0.0935828	-0.0258228	-0.2734347	0.85742836	-0.1805653
0.06475409	0.09573884	-0.0360486	-0.0278478	0.01906982	0.09031763	0.08262108
0.2430082	0.62270601	0.10935275	0.10502692	0.17132261	0.55095102	0.09640757
-0.2696299	0.0589338	-0.0159225	0.00126118	-0.1237278	-0.1931722	-0.3255736
-1.0600436	0.12269346	0.03917256	0.03526346	-0.0534081	-0.3349626	-0.208072
-0.2403062	0.05195586	0.17010819	0.03163957	-0.030489	-0.6420274	0.24219798
-0.2521725	0.30006623	0.1172996	0.15103026	0.02792546	-0.1533069	0.02903587
0.56413367	-2.0170369	0.17877169	0.16793874	-0.1622916	-0.8235041	-0.0526666
-0.1687575	-0.0781217	0.1502907	0.21985189	0.13937338	-0.145875	0.27598722
-0.4310982	-0.0260763	-0.1423917	-0.0810687	-0.0641864	0.05674188	-0.1989317
-0.6062323	0.15541452	0.2237577	0.04305629	0.13608161	1.0048453	0.08428233
-0.9228247	-0.1252404	-0.0150531	0.03762293	-0.0892213	0.0574151	-0.2860512
-0.0510042	-0.7312018	-0.1631788	-0.1363989	0.00515064	0.45330774	-0.1079433
-1.0561595	-0.677217	-0.2588594	0.11241071	0.2051929	0.32232653	0.16761683
-0.0550577	-0.000402	0.02703077	0.0142857	0.00687256	-0.0001606	2.92E-05
0.02716057	-0.1345877	-0.0618495	-0.0270792	-0.1045417	0.03071448	0.09345751
0.21719432	0.16628478	0.01442305	-0.0344133	0.0298876	0.15863689	-0.073905
0.80275019	-0.0081036	0.02330963	0.06978081	-0.0445637	-0.0678996	-0.001622
-0.0828323	0.27568953	0.06019074	0.03269559	0.16161804	0.15792681	0.23702228
0.06771049	0.06778562	-0.1404048	0.06074782	0.07050536	0.00523542	-0.1265436
-1.0294879	0.40491044	0.317907	0.30227258	0.18159567	-0.0068607	0.0423256
-0.0727704	0.85964281	0.03152212	-0.0259183	0.10701994	-0.5994	-0.3798581
0.00134693	-0.0042519	-0.0631769	-0.1179706	0.01443493	0.05439347	-0.3273278
0.0701603	-0.0327869	0.04750446	-0.099905	0.08346711	-0.030241	0.06197907
0.25698672	0.42682416	-0.09401	0.01286911	-0.0452956	0.1446321	-0.0175686
-0.0951387	-0.4279108	0.60066998	0.48681887	0.24946359	-0.4248401	0.04111055
-0.0115262	0.12899165	-0.0467543	-0.1061201	-0.0183066	-0.4491797	-0.1240459
-0.2393971	-0.2169822	-0.0274079	-0.0847444	-0.1908792	0.12821482	-0.0187633
-0.2413907	-0.1241081	0.12608356	0.06089137	0.03034349	-0.1162029	-0.1716067
0.11270207	-0.1510084	0.10117762	-0.0616199	-0.1054368	-0.0215439	0.05542219
-0.3433205	0.12645913	-0.0011724	-0.2111091	0.22302884	-0.0938784	-0.0243791
-0.3923502	-0.5103803	-0.3276257	0.15813289	0.72819846	0.19560215	0.25685647
-0.1333749	-0.9898811	0.20679946	0.14901588	0.09828985	-0.1748644	0.21005205
0.31162185	-0.302557	0.17334207	0.01835364	0.05936086	0.00500793	0.37190029
0.22419093	0.02745401	-0.0485772	-0.0451414	0.05389756	0.18226883	-0.0885227

-0.009451	-0.0788131	0.05486501	0.0597803	-0.0056063	-0.0027308	-0.0216331
-0.23709	-0.024979	-0.0047843	-0.065237	0.02857	0.078019	0.090072
0.21880976	-0.2479756	0.29487116	0.15224357	-0.0365872	-0.0894124	0.45974518
-0.3487012	-0.22139	0.06192931	-0.1154396	0.0292634	0.10188345	0.00957125
-0.2139594	-0.0306767	-0.1221408	-0.0511077	-0.023948	-0.1364099	-0.1548279
0.24951833	-0.1919998	-0.4240707	-0.0171049	0.22007439	0.6994856	0.08523044
-0.7370452	0.54799627	-0.0513064	-0.1605562	-0.0174701	0.08541289	-0.2122608
-0.48396	0.6718	-0.024501	-0.11234	-0.024324	-0.012214	-0.15308
0.63297301	-0.0826439	-0.0772848	-0.2682314	-0.1027754	0.35507756	-0.0433293
-0.135628	0.1896689	-0.1090455	-0.0839795	0.01232663	0.06036734	0.09675161
-0.0692193	-0.0375583	0.13861695	0.03756506	0.01212787	0.52564729	0.13629929
-0.1931751	0.04255313	-0.1567017	-0.0720735	0.08907871	0.73074221	0.07500522
-0.7182533	-0.0589321	0.0750253	-0.0537492	0.12535338	0.43439066	0.02687518
0.06862417	-0.0667203	0.09556812	0.10673731	0.00199847	0.15212614	0.12205769
0.59338102	0.04530883	-0.0988981	-0.0609447	0.01039977	0.218454	-0.0180531
0.10233206	0.19730417	0.02510228	0.07913448	0.17923508	0.06301416	0.10990016
-0.6562645	-0.056379	-0.1740117	-0.0273859	-0.0454648	0.21480967	-0.0353395
0.08170782	0.96349576	-0.218059	-0.2122932	-0.0641333	1.2129721	0.44284559
-0.2208772	0.14313391	-0.019033	-0.0233473	-0.1408406	0.36280584	0.07330151
-0.8329746	0.01365544	-0.2139154	-0.0723497	-0.0797748	-0.0937147	-0.1406807
0.26440644	0.0038711	-0.0549725	-0.0705131	-0.0067249	-0.0859315	0.01249756
-0.4865775	0.18837981	0.11348748	0.03693666	0.15793286	0.61788428	-0.0183793
-0.1711301	0.14899412	0.1542253	0.04228845	0.04892561	-0.1332403	0.08281654
0.45404827	0.4814705	0.02238071	0.16057591	0.17512126	-0.4833297	0.13134858
0.044157	0.25103601	0.03038665	-0.0054527	-0.014329	0.10871911	0.07579496
0.11353986	0.0558337	-0.0639414	-0.1703758	0.06198603	-0.4238749	-0.0043545
-0.3115709	0.11590716	0.49544467	0.19414358	0.14477292	1.5602122	-0.0763464
-0.5745685	-0.0904241	0.13026408	0.05813579	-0.0311486	0.24711903	-0.2653846
-0.4889093	-0.8703327	-0.1068383	-0.0523479	0.07824852	-0.2723567	-0.0265597
0.27407411	0.11644386	-0.1541606	-0.0635889	-0.0207493	0.15644123	-0.0454738
-0.3054366	0.1733355	0.01116248	-0.0072789	0.18720194	0.41688503	0.04432803
0.05586986	-0.04929	-0.0173384	-0.0508566	0.00660367	0.12252035	-0.0150853
0.08171107	0.01881066	-0.0671636	-0.1997145	-0.0215588	0.25144514	-0.2022528
-1.0972638	0.15304115	-0.0087382	-0.2182003	-0.1480243	0.06334541	-0.2789697
-0.6932555	0.27269558	-0.112086	-0.2875986	-0.1195877	0.80842329	-0.2425117
-1.1234422	-0.0494595	-0.0427755	0.00645237	0.10260975	-0.1344014	0.11141047
-0.1755349	0.30274791	-0.1487996	-0.2015444	-0.0996398	0.08448024	-0.1566294
0.1057233	0.37058412	0.25812764	0.11845939	0.12034942	-0.1380734	0.01715785
0.11474824	0.31591398	-0.16024	-0.2028975	-0.0031524	0.55244081	-0.0902246
0.37807627	0.37587372	-0.0142522	-0.0983796	0.01081739	0.1833728	0.05386536
-0.3776095	0.04599648	-0.0037038	-0.1114589	0.17140343	0.02141138	0.11709529
0.48543452	0.05472958	0.04344055	-0.0087543	0.10212717	0.0738536	0.20012083

-0.2817195	-0.0425851	-0.0413635	-0.0267537	0.09358973	0.29223631	-0.1485828
0.10622366	0.07906841	0.04421666	0.16796068	0.10397218	-0.3474027	0.1750512
-0.1630901	-0.0443314	-0.0715459	-0.0694913	0.03509166	-0.0609334	0.03475008
-0.3621994	-0.1506789	-0.002197	-0.1524494	-0.0387207	-0.7308907	-0.0109453
-0.0754411	0.25278751	0.09333216	-0.0509502	-0.0836901	-0.047565	-0.0040481
-0.8686127	0.26174787	-0.081412	0.01106466	0.23598697	0.06807435	-0.0756367
-0.4790101	0.38971244	-0.1701085	-0.0948202	-0.0420209	0.29066379	-0.1764968
0.29935365	0.02684441	0.15518996	0.08659799	0.16234774	-0.2179297	-0.0055067
-0.1715283	0.02178597	-0.0070584	-0.0168531	0.03855081	0.24712789	0.00227154
-0.0031527	-0.3711533	-0.1101554	-0.0364909	0.08373814	-0.1139699	0.0254748
-0.0248055	0.13571043	0.03735901	-0.1621699	0.02700605	0.06141494	-0.0400552
-0.6144322	-0.0994165	-0.4732494	-0.2226311	0.0229802	1.0832667	-0.1693186
0.17557277	-0.116926	0.16604054	-0.0304171	-0.0795666	-0.1446958	0.14878448
-0.0452119	0.03314182	0.12506567	-0.027997	0.07870098	-0.0117858	0.14406497
-0.1741716	0.073458	0.00882997	-0.1719283	-0.0543368	0.0598294	-0.0122458
0.05195609	0.17146512	0.2811405	0.13926319	0.25344171	0.09764485	0.18720992
-0.292627	0.06847395	0.18001746	0.06746422	-0.0236666	0.13233871	0.18296895
-0.7067948	0.30548789	-0.097866	-0.2324234	-0.2335375	0.38407702	-0.3410833
0.06909978	0.24805256	-0.1477955	-0.2802875	-0.0319607	0.23833028	-0.038736
1.8380868	0.28457163	-0.1602229	-0.1599914	0.23088984	0.50264862	0.22734493

test_GSE683	test_GSE683	test_GSE683	ref_GSE6836	test_GSE683	test_GSE683	test_GSE683
ref_GSE6836	ref_GSE6836	ref_GSE6836	ref_GSE6836	ref_GSE6836	ref_GSE6836	ref_GSE6836
GSE6836	GSE6836	GSE6836	GSE6836	GSE6836	GSE6836	GSE6836
GEO	GEO	GEO	GEO	GEO	GEO	GEO
affymetrix	affymetrix	affymetrix	affymetrix	affymetrix	affymetrix	affymetrix
relE_b1563:+	relE_b1563:+	relE_b1563:+	NORFLOXACI	NORFLOXACI	zipA_b2412:+1	
1382	1383	1384	1400	1401	1403	1406
0.1711126	-0.2084208	0.06127939	0.55477437	1.3116371	-0.274243	-0.0913459
-0.0441989	-0.1362287	-0.0309755	0.1425599	0.62419872	0.42103509	-0.0467106
0.23144673	0.44532718	1.2612194	1.1998047	1.4882427	0.15076848	1.3660549
-0.1733984	-0.2179864	-0.3303044	0.26341005	0.27096832	-0.0719764	0.19059587
-0.1213534	-0.1439127	-0.2088276	0.16916563	0.26603199	-0.1968936	0.0538935
-0.4026261	-0.3686538	-0.1663154	0.25385392	0.45354674	-0.6482007	0.38043791
0.22298521	-0.3413808	-0.6334027	0.19408913	0.5286511	-0.5871757	0.38335168
0.00361981	-0.0761279	-0.2567057	1.0180811	1.5266615	-0.3222453	0.27022324
-0.0751269	0.01262671	0.28961345	-0.1571506	0.0697389	0.34567453	-0.1022243
-0.0808363	-0.0733356	-0.0995327	0.08133824	0.17688489	0.06891477	0.11518699
0.03831372	-0.1646734	-0.1334676	1.4128892	1.6891077	-0.6510552	0.36741674
0.41524281	0.56458807	1.0345188	0.85012014	0.52657649	0.26236862	0.35473201
0.48557433	0.53157867	0.82048623	0.56225591	0.49543896	-0.5400201	0.08588893
0.36078877	0.31977669	0.49441764	0.00032047	0.07320918	-0.3072664	0.05060306
0.05320651	0.10667884	0.21449423	0.52578333	0.31987055	-0.3106495	-0.0579519
0.02899296	-0.089859	0.044587	0.22242239	0.25377913	-0.4355528	0.04271027
0.51350636	0.60337467	0.96206716	0.70894537	0.50484526	-0.0075171	0.03651778
1.73401	1.8650145	2.4375425	0.31509591	0.38400049	0.0851075	-0.3493818
0.14141269	-0.0456437	0.20121281	0.15481952	0.20445193	-0.101514	0.08843607
0.10364495	0.05649759	0.08746157	0.01784865	-0.0339335	0.09096513	0.06253626
-0.1559201	-0.288858	-0.2355836	0.10431388	-0.0440067	-0.5533682	0.06470759
-0.0470239	-0.1109631	0.01318043	-0.1411409	-0.0471985	-0.257059	0.09850975
0.02346976	0.22092129	0.66974314	0.52535942	0.10134224	-0.3959611	-0.0259223
-0.1103729	-0.1982225	-0.1997396	0.24880055	0.30083832	0.06103631	0.07985989
0.11581707	0.42837991	1.0977001	-0.6214875	-0.3778286	0.39956439	-0.6054263
0.14367987	0.20430217	0.44267692	0.63412637	0.61433642	-0.6622347	0.0630488
0.28560741	0.22430749	0.18284563	0.3449636	0.7703489	0.25179836	0.36444761
-0.0620897	-0.2032228	-0.2288134	-0.8339261	-0.8272005	0.43123415	0.24202693
0.25412751	0.28641259	0.37521505	0.62600403	0.89694178	-0.9074084	0.16567724
0.35527364	0.49195454	0.82743769	0.5813992	0.77004614	-1.0200601	0.22380476
0.18070415	0.04593003	0.19204286	0.00562398	0.19542649	-0.2769286	0.02468369
0.1708786	0.17159673	0.29231299	0.37814503	0.34224617	-0.7729133	0.28875305

-0.0460593	-0.1307678	0.08873418	-0.2771538	-0.1571554	-0.4904556	0.22316164
-0.0509679	-0.0682557	-0.1424469	0.05003077	0.25070074	0.43639704	0.00486459
-0.158016	-0.0946322	0.07420401	-0.0842491	0.14119974	0.28314663	0.50009117
-0.1347301	-0.0858818	-0.0898999	0.07463838	0.07988498	-0.2918177	-0.1400741
-0.0120478	-0.1327515	-0.1026324	-0.0473939	-0.0248219	0.1799553	-0.0039614
0.41484885	0.28930232	0.40086752	0.15508355	0.3602982	0.0424583	0.01500045
-0.0531137	-0.0817097	-0.1798168	-0.1395372	0.16373215	-0.1458019	0.08502649
-0.0217483	-0.1203638	-0.0234251	0.00650246	0.02996897	-0.2734003	0.12499339
0.06200614	0.13618739	0.32321943	-0.2720889	-0.1604981	-0.3668418	-0.1058242
0.00107633	-0.1047327	0.10521383	0.04487263	0.09766894	-0.1968584	0.07680846
0.23370071	0.28088872	0.28685055	-0.275391	-0.1377433	0.21296969	-0.2173512
0.16431966	-0.0515041	-0.3818053	-0.1774895	-0.1871046	0.1933199	0.16249895
0.25548078	0.18346416	0.22645328	-0.1014977	-0.0362233	0.12814895	-0.1768106
-0.3559425	-0.4244571	-0.7501438	-0.2076376	-0.2098719	-0.1918812	0.02063626
0.46192627	0.5583125	0.59493805	-0.482624	-0.4718884	-0.5827233	-0.1436408
0.97417506	0.94131215	1.1214211	-0.917462	-0.7752323	-0.3031284	-0.4986429
0.13178444	0.14550573	0.07726227	-0.1479395	-0.0390104	-0.3383987	-0.0775652
0.03566901	-0.1245951	0.07166216	-0.2696695	-0.1332023	-0.3753335	-0.056904
-0.1235062	-0.1982838	-0.2567373	-0.5669601	-0.4804687	-0.0233053	0.19534116
0.00694146	0.06562791	0.17121369	0.07111101	-0.0031213	-0.5762567	-0.0407883
-0.1754594	-0.3003814	-0.3262943	0.42673554	0.44333306	-0.1943266	0.64274657
-0.0069732	-0.0162818	-0.1840526	0.11973055	0.25146722	-0.3741882	0.15958748
0.38977882	0.56579255	0.56558695	-0.1941204	-0.1667707	-0.1681867	-0.1487586
0.02672931	0.11078639	0.28045523	-0.3640059	-0.2856067	-0.6609028	-0.0493884
-0.1018104	-0.1792269	0.24672963	0.01879524	0.0197911	-0.3531228	0.05621317
0.27688862	0.3667332	0.7119219	0.08978453	0.02378668	-0.400724	0.59933091
0.18801683	0.06093831	0.22616428	0.18953158	0.0808592	-0.3909821	0.42401294
-0.009054	-0.0284199	-0.058235	-0.0713318	-0.0833055	0.14170516	-0.0074556
0.15997493	0.09470895	0.33552646	-0.8473594	-0.8951614	-0.4341117	-0.092479
1.0622788	1.4283358	2.1213656	0.26698491	0.63264905	-0.4641965	-0.3458102
0.50387023	0.72149321	1.162081	-0.3038722	-0.1241905	-0.5683233	-0.3363897
0.05457775	0.04423654	0.37167004	-0.0973355	-0.0556086	-0.1439855	0.05091209
0.31942828	0.42779458	0.69309522	-0.3391451	-0.3654055	0.23194024	0.00934161
-0.3570642	-0.2377932	-0.0005531	-0.2600353	-0.5777089	0.05644228	-0.0990917
0.13764031	0.01305664	0.0564917	-0.1939043	-0.2643389	-0.1181942	-0.00409
0.11029525	0.06441594	0.10918512	-0.0087098	0.22842306	-0.6370558	0.15734811
0.05304934	0.20670523	0.29153626	-0.1097808	-0.4253876	-0.2945797	-0.1046402
-0.1520525	-0.1893841	-0.0159174	3.506869	3.8787375	-1.0774063	0.70244833
0.16484648	0.2997868	0.43771961	-0.3844998	-0.2244314	0.14826083	-0.0130593
-0.0191197	-0.1079569	-0.0045333	-0.7503026	-0.7196626	-0.2138465	0.09806759
-0.0307537	-0.0165992	-0.0458056	-0.1512662	-0.0608102	0.15574551	0.12073548
-0.4593318	-0.2702241	-0.2297837	-0.9855882	-1.1072841	-0.0510111	-0.0904642

0.34465738	0.31236663	0.380252	-0.4160086	-0.4183218	0.28644917	0.05789502
-0.0295435	-0.0561153	0.03495777	-0.7159398	-0.4256077	0.08159497	0.27265056
0.01513073	-0.1124479	0.13608363	-0.8896933	-0.9185333	-0.1998202	0.02049968
0.53978715	0.61875098	1.3667594	-0.2765467	-0.2569454	-0.0282359	-0.1864826
-0.0926976	-0.0786063	-0.2088775	-0.0102388	0.15632943	0.02770171	0.30056743
-0.0110036	0.13255508	0.45424035	-0.0112555	0.05747017	0.08554254	0.16450173
0.22621901	0.02037584	0.07088103	-0.328201	-0.3620931	-0.0823897	-0.1345818
0.09118618	-0.0395753	0.03441487	-0.7395902	-0.7705555	-0.6362489	0.18329772
0.33480549	0.22804313	0.49470666	-0.3541112	-0.4222781	-0.2625822	-0.0122291
0.16378576	-0.0020731	0.10577078	-0.2472757	-0.060159	-0.2588198	-0.0188499
-0.0574408	-0.1201585	-0.1299513	-0.3016927	-0.1718823	-0.0806547	-0.163594
-0.1432355	-0.1632791	-0.1768819	-0.3071525	-0.2422773	-0.1202076	-0.0354511
-0.0066184	0.1934169	0.15611015	-0.1023539	-0.2229722	0.12561171	-0.1017917
0.0268363	-0.0720425	0.20038513	-0.1165419	-0.1588778	-0.4816498	0.05406005
0.2343058	0.60910331	1.0731568	-0.1995125	-0.3754344	0.0717255	-0.0067553
-0.0529764	-0.0898217	0.16369077	-0.7621393	-0.7038147	-0.3168071	-0.1723922
-0.0349993	-0.1113607	-0.1452479	-0.434127	-0.2435434	-0.1392281	-0.1099574
0.03817556	-0.049968	-0.0082347	0.08507345	-0.2245459	-0.5533707	-0.2222524
0.38613681	0.50134173	0.54080828	0.1851414	0.26100796	0.29196	0.08217093
0.40002191	0.31255019	0.56760835	-0.2063009	-0.2342729	-0.1535911	0.07873611
-0.0918528	-0.0823899	-0.0903031	-0.0285403	-0.1347413	-0.1268335	-0.0922913
0.02604928	-0.0126287	0.06910523	0.08042452	0.10555344	-0.1440719	0.22278677
0.06675196	-0.0163114	-0.0039403	0.15558861	0.23086218	-0.2582312	0.39274992
-0.0817532	-0.1332941	-0.0086245	0.29605432	0.01107075	-0.4581981	0.1259822
-0.1871023	-0.1278367	-0.1657463	-0.2197339	-0.1263051	-0.0550414	0.10054761
0.39973292	0.25040722	0.27604904	-0.5448559	-0.6477219	0.32364	-0.6426328
0.30569405	0.16543925	0.1447092	-0.9166319	-1.0596731	0.30062831	-0.1993232
-0.1121772	-0.1344421	0.06171938	-0.9451163	-0.8056538	-0.2183515	-0.1709969
0.14268	0.10751	0.5478	0.81	0.9324	-0.5255	0.19607
0.25864	0.21839	0.63171	-0.90811	-0.61113	-0.26732	-0.42524
1.1569	1.3269	1.9367	-1.0842	-0.81668	-0.17946	-0.60589
4.3061	4.7952	5.1788	-0.25168	-0.31917	-0.35575	-0.18498
2.4069	2.6982	2.9057	-0.2362	-0.12585	-0.34981	-0.025489
-0.097541	-0.065292	-0.17345	-0.10814	0.039482	0.21013	-0.13865
0.22879	0.18877	0.16861	-0.82104	-0.73907	0.15669	-0.1496
-0.2160373	-0.1776254	-0.0994063	0.02073662	0.01356873	-0.3050525	0.72495344
-0.1431112	-0.1143136	-0.0672076	-0.1567043	-0.1786379	-0.1675262	-0.0737084
0.00705621	0.18757298	0.25073808	-0.7152651	-0.9180455	0.03999032	-0.0928979
-0.2391408	-0.2689994	-0.1756726	-0.5177108	-0.5129612	-0.1774138	-0.0218844
-0.0137701	-0.1488551	-0.0598422	-0.2293486	-0.1236824	-0.2002655	0.13911082
1.0254582	1.0679291	1.2486577	-0.9704922	-1.03525	-0.0185825	-0.4578492
0.23183186	0.39558518	0.21351619	-0.7187286	-0.6581038	-0.0410737	0.13270791

0.18411299	0.15712254	0.3788321	-0.5174715	-0.4806116	0.16568699	-0.2882099
0.2166676	0.13175485	0.30616051	-1.2485969	-1.1211049	-0.1271452	-0.3965428
-0.1527432	-0.1371805	-0.0146574	-0.3203026	-0.2562609	-0.0744328	-0.0215465
0.02499987	0.08266927	0.05157056	-0.3493355	-0.3308288	-0.133586	-0.0560505
0.00911838	-0.1211111	-0.0054244	-0.6368239	-0.7551194	-0.1440853	0.18165629
0.07390952	0.24253921	0.31016955	-0.7150657	-0.7330374	-0.0049616	0.1760625
0.04565505	-0.030266	0.02490471	-0.0620479	-0.1455952	-0.1351576	-0.0868295
0.03761686	0.03299759	0.39290654	-0.0247275	-0.0902869	-0.1091069	0.24829695
0.00673526	0.19285834	0.21346341	-0.1341606	-0.1248734	0.07656984	0.0799421
-0.37284	-0.2875037	-0.1123815	0.04723829	0.00898676	0.01992534	0.53246496
-1.0842076	-1.2448301	-1.9167827	-0.7034925	-0.9904469	-0.09708	0.08299085
0.04971789	0.01797844	0.03838611	-0.6328567	-0.5216537	0.04581299	0.00995563
-0.5774395	-0.5691382	-0.6341814	0.14610183	0.01021416	-0.2191192	0.66567109
0.48577343	0.41967779	0.46504824	-0.2169337	-0.2075689	-0.0524418	0.09034929
0.80793186	0.81272504	1.0123387	-0.3341158	-0.32109	0.04465276	0.17142898
-0.0112618	-0.066043	-0.1459511	-0.6578582	-0.7271678	-0.1619571	-0.1535266
0.15327948	0.10222613	0.38609843	-0.0412354	-0.0418897	-0.4146691	0.23508123
0.14804263	0.1229288	0.52113563	0.0082553	-0.1847315	-0.0086634	-0.0258239
0.50602325	0.65234219	1.0445971	0.60793361	0.66902723	-0.2391453	0.24580254
0.08424221	0.09292964	0.34698605	-0.1014757	-0.1662465	0.26348203	-0.0580267
-0.1518114	-0.0678518	-0.1933211	-0.3244696	-0.2584632	0.21600538	-0.0054664
-0.0650369	0.03674696	-0.0994174	-0.2021929	-0.1693514	-0.0457278	0.07599852
-0.045658	-0.068281	0.11532	-0.038204	0.016111	-0.1227	-0.043354
-0.3194422	-0.0627299	-0.1539004	0.18935808	0.21080367	-0.3335198	-0.0223777
-0.2167007	-0.2149085	-0.1402075	-0.5710328	-0.7124681	-0.1066431	0.14198703
0.20984393	0.0770618	0.0609472	-0.1358096	0.00663282	-0.0116299	0.13995056
0.08925418	0.28564307	0.49384875	-0.263264	-0.3257608	0.00066298	0.06940053
0.20383199	0.11399596	0.27121027	0.02605984	0.0010972	0.05138576	0.05215044
-0.3534071	-0.2996145	-0.3589046	-0.4078591	-0.5273664	-0.2619437	0.59431548
0.04099805	-0.0391197	0.06994354	-0.1093041	-0.0574401	0.08734265	0.08629174
-0.1912546	-0.1312231	0.30636006	0.00701207	-0.2721275	-0.3207142	0.26368816
-0.1899981	-0.2098005	-0.0927083	-0.0827836	-0.0199661	-0.1348527	0.00867069
-0.1456721	-0.2373544	-0.366786	-0.1642638	0.08604963	-0.0530477	-0.018634
-0.0104313	0.10496398	0.08168249	-0.1874379	0.00148312	0.05504337	-0.0995646
0.36132857	0.3231467	0.55228166	-0.2753995	-0.1256286	-0.2803347	-0.0754921
0.0299808	0.1530858	0.04592731	-0.3579461	-0.3335799	0.10864602	-0.0799402
-0.0513314	-0.0044752	-0.1201094	-0.2827886	-0.2912686	0.08835684	0.15220459
-0.0656256	-0.2555073	-0.1986894	-0.2663572	-0.2345368	0.07900869	0.00743563
-0.0932457	-0.0242596	-0.0666616	-0.151048	-0.2074513	0.11192859	-0.0538289
0.34446101	0.38692082	0.8694457	-0.369295	-0.4615649	-0.1517837	0.08425623
-0.0729567	-0.1241456	-0.1864701	-0.0199371	-0.0990647	-0.0630284	-0.0596856
-0.2998066	-0.2051511	-0.187864	0.34962319	0.04933308	-0.5938301	0.23512555

-0.0877805	0.01748827	-0.129719	-0.2378788	-0.0673021	-0.0231751	-0.003259
0.1808816	0.10302986	0.22164879	-0.0275176	0.02412067	-0.15221	0.0622587
0.07232393	0.12967515	0.13597284	-0.0603359	-0.0615412	0.04946292	-0.0660708
0.01030845	-0.0376876	-0.1079042	0.20691585	0.35281445	-0.097614	0.04915006
-0.0073696	0.17378383	0.23769531	0.01182932	-0.0833781	0.17741458	0.04953034
0.06867879	-0.0654208	0.03039493	0.09376945	0.10545887	-0.0128887	-0.0796133
0.41997207	0.16563282	0.59692986	-0.0444376	0.00448467	-0.1127807	0.25670147
0.14783029	0.08236219	0.31249104	-0.4346499	-0.5910949	0.29493311	-0.3281649
-0.1394752	0.07443596	0.53815239	0.30272143	0.11142387	-0.1609371	0.08512522
0.07081242	-0.0177336	0.1047507	-0.0092213	-0.1031395	0.23806034	0.04190969
0.19152709	0.13659854	-0.0304255	-0.1994245	-0.1075829	0.14511239	-0.187389
0.27171862	0.35776948	0.59468325	0.08226444	0.1904894	0.0570201	0.16160244
0.08609311	0.12353991	0.48804949	0.22295477	0.16865844	0.27632563	0.10974894
-0.0045238	0.0660917	0.03884795	0.3075665	0.21121089	0.33526406	0.0832035
0.26046009	0.38622879	0.32635229	0.1950843	0.10941463	0.20802215	-0.0257505
0.04903543	0.02908738	0.37461913	-0.1652777	-0.0656249	0.0262963	-0.0211984
0.07172794	-0.0136971	0.25573676	0.21728021	0.4457214	0.34112922	-0.3832775
-0.4713635	-0.2994934	-0.351104	-0.16563	0.08015066	0.12283932	-0.0693777
0.12496031	0.03587306	0.06071178	0.25254995	0.12813899	-0.0880487	-0.0984094
-0.6155433	-0.7261258	-0.8009671	0.91548893	1.207326	-1.0059296	0.07808205
-0.3295148	-0.389244	-0.4460796	0.71885785	0.77250228	-0.3300972	0.10648264
0.38941843	0.33492349	0.70641032	0.25756662	0.23322844	0.07428875	0.09558074
-0.0419754	-0.0020715	-0.0368157	0.05706659	0.00857892	-0.036274	0.17523246
-0.173406	-0.0336794	-0.2474328	-0.0275997	0.11463415	0.20871134	-0.0895857
0.03589716	0.04618037	-0.0284612	0.17119803	0.2938552	-0.0215012	0.16380488
0.03911075	-0.0124875	0.1955709	0.32617598	0.26132447	0.20427998	0.16871173
0.26823435	0.17722724	0.25757609	0.38059064	0.51519803	0.13371406	-0.0774547
-0.4434312	-0.2437486	-0.3202275	0.52844555	0.4078825	-0.1181163	0.70158061
0.66216917	0.78456851	1.1304541	0.56628628	0.61804458	-0.084945	-0.1492539
0.0504908	-0.0589677	-0.0186046	-0.028996	0.01398121	-0.3596787	0.55521309
-0.1402047	-0.0876279	-0.1911725	0.1195579	0.37860248	-0.2151035	0.2385607
0.02822491	0.08480851	0.04782599	0.42446793	0.40568512	-0.2034811	-0.0271757
-0.1535677	-0.112704	-0.2214307	0.49899288	0.59856765	-0.0858055	0.05711069
-0.014878	0.04702277	-0.0100454	0.07380296	0.11311361	0.19686006	-0.0023836
-0.1919896	-0.0962402	0.06146884	0.5789359	0.48258648	-0.2039776	0.39355834
-0.2202845	-0.116695	-0.2121059	0.21968922	0.22833312	-0.5043333	0.47449945
-0.1839329	-0.1040826	0.05410386	0.28041511	0.20889349	-0.0732362	0.01426083
0.38644807	0.36553436	0.33058854	0.26387744	0.38126681	-0.0431334	0.05836214
0.64904862	1.1168071	2.2277743	0.59335137	0.26082983	0.11252336	0.16655504
-0.0868546	-0.1655447	-0.1055554	0.31914658	0.25520872	0.03936819	0.12697544
0.71263842	0.90717942	1.1798781	0.50533338	0.54204471	-0.0515068	0.14606141
0.4589744	0.58121631	0.9443224	0.11800673	0.36733641	-0.0754082	0.06357396

-0.2119318	-0.1810508	-0.162436	0.29576843	0.41838119	-0.1951229	0.22057118
0.19163373	0.37110612	0.59281898	0.33494778	0.25747452	0.22456012	-0.0837507
0.00439173	-0.0874204	0.05342003	0.17570651	0.02179761	-0.1910271	0.11134017
0.03736198	-0.0127811	-0.022883	0.30270348	0.19360845	-0.0552826	0.21613412
0.48361513	0.56281701	0.7736364	0.37822402	0.22472658	0.2044271	-0.0517766
-0.5034243	-0.2780893	-0.2134563	0.73222811	1.0161039	0.05535871	0.52152897
0.27199859	0.18519659	0.2221836	0.16710656	0.06465955	-0.3495607	0.67456572
-0.1386873	-0.3501213	-0.4435862	0.42269983	0.1211154	0.03049122	0.02172477
-0.1526572	-0.0805684	-0.0793135	0.50269339	0.19844315	0.24772508	0.11337713
0.16581558	0.36866037	0.76342513	0.82989925	0.76974605	-0.0522204	0.32587222
0.03385362	0.13560646	0.16280885	-0.0436486	-0.1018039	0.3402075	0.06070573
0.02593374	-0.0900746	-0.0653742	0.15354945	0.33460344	-0.0441191	0.58116891
0.22518987	0.2847578	0.51365231	-0.2710827	-0.2704069	0.2516766	-0.0534708
0.22419176	0.17721939	0.2995608	0.13877358	0.26400176	0.0758458	0.25777276
0.3906805	0.35102449	0.37793876	-1.0229245	-1.3005863	0.39112842	-0.6180712
0.162122	0.23018786	0.09363691	0.21751168	0.02443652	0.29774567	0.0101313
1.1976212	1.138586	1.2320396	0.56876206	0.72360874	-0.5324154	0.21722489
-0.0748832	-0.05966	-0.0164885	1.1569861	1.2369934	-0.0846725	0.80170626
0.25206253	0.27023959	0.33841102	0.49669904	0.36093652	0.04582709	0.13935621
-0.2177302	-0.1135473	-0.0714655	0.56344125	0.51956644	0.11434155	0.2383627
-0.0791611	-0.0114453	0.04133461	0.81859597	0.6663138	0.21298923	0.90254674
-0.0179249	0.02416393	0.07142572	0.23929776	0.2019151	0.26480851	0.13054757
0.0823639	0.19832401	0.30084119	0.11630467	0.07575483	0.20533989	0.00299032
-0.2177054	-0.1671284	-0.25745	0.2873167	0.21122159	-0.3904349	0.12450689
0.04806931	-0.0081918	0.02447143	-0.1782485	-0.1852587	0.07896673	0.02608091
0.35948887	0.40154227	0.48565992	0.46121292	0.474667	-0.1214152	0.04983761
-0.0546788	0.21595323	-0.1618637	0.51376692	0.47547353	0.08133475	0.18444577
-0.2694772	-0.1489148	-0.3271259	1.2476789	1.8672031	0.03143775	0.81101177
0.08382906	0.04919908	0.1745737	0.27191811	0.66547472	-0.1185437	0.26332337
0.03865617	-0.0853543	0.08817056	1.0789996	1.7239421	-0.1271844	0.56131029
0.28229288	0.37521887	0.61379391	0.9309631	0.64065209	0.39356437	0.12902231
-0.1348648	-0.0479131	-0.2638175	0.50124477	0.48090139	0.1642705	0.45607538
-0.077883	0.07264348	0.08846478	1.3319967	1.5028093	0.2386807	0.39415553
0.38486954	0.42507103	0.64171452	-0.2651582	-0.1148054	0.04311801	-0.1925175
-0.2489726	-0.1352296	-0.0726566	0.36362006	0.16579629	0.02188609	0.18879018
0.24413896	0.26089689	0.37599423	0.37014991	0.15226198	0.21620405	0.29212589
-0.0829615	0.01121334	-0.0655721	0.38563842	0.32911129	0.30852785	0.08617859
0.86564859	0.94882595	1.0428395	0.47565863	0.57275759	-0.3847867	0.23995244
0.15384981	0.30321946	0.49584855	0.59882973	-0.1816934	-0.3902226	-0.0214612
0.31279521	0.23358289	0.31785922	0.22544112	-0.0097797	0.14419488	-0.2312216
0.46310446	0.44209134	0.66060865	0.39035715	0.34186111	0.33231141	-0.1363518
-0.0991885	-0.0428045	0.31631348	0.47572793	0.36446481	0.06784561	0.03185496

-0.0634456	0.08659276	0.01209617	0.64786308	0.41522569	0.3622492	0.01254957
-0.20998	-0.10264	-0.10175	0.2671	0.18845	0.12825	0.14709
0.85393983	0.89650664	1.0292989	0.6637882	0.97556344	0.1853592	-0.1875061
0.05273362	0.11518465	0.03345023	0.52221744	0.40998674	-0.5108606	0.44820526
-0.0688685	-0.0746848	-0.083752	0.0851056	0.23251718	-0.0794963	0.03698712
0.39314686	0.28873228	0.13131687	0.65382662	0.32794264	0.26283929	0.43582339
-0.1796482	-0.2374222	-0.2163471	0.4370911	0.53645348	0.06635484	0.24141807
-0.020786	0.0054113	0.20892	0.14013	0.037162	0.14342	0.21033
-0.0783734	0.19084124	0.28723093	0.78036597	0.76827215	0.22999154	0.27881494
-0.0131668	-0.0134977	-0.0106265	0.02003252	0.04918904	-0.0170134	0.06463122
-0.2663624	-0.2502799	-0.2571276	0.38780917	0.52498439	-0.0631986	0.00800756
-0.0731905	0.20817129	0.18811153	0.52794997	0.45741134	-0.4071345	0.45588575
0.07906489	0.12195354	0.25417922	0.49084723	0.40082514	0.22096244	0.16791352
-0.0130357	0.08138006	0.01477012	0.46350242	0.43438081	0.44701138	-0.0182792
-0.0307196	0.01365723	-0.0908682	0.3781383	0.25576017	0.04940742	0.0272219
0.14437151	0.15920569	0.0759444	0.68975665	0.48701021	-0.0669687	0.10893463
-0.3667436	-0.3188091	-0.4896086	0.75273884	0.65084676	-0.3217006	0.21443634
-0.3660327	-0.1408682	-0.0779114	1.0360629	0.78061965	-0.0674441	0.94324667
0.13674288	0.37150347	0.28974138	0.22121498	0.48045357	0.42959927	-0.1886039
0.00420741	-0.2206782	-0.2270847	0.03272107	0.15337784	0.08331727	0.44459445
0.00615251	-0.1055843	0.11895389	0.23120372	0.33308018	0.24258416	0.34505648
0.18694978	0.20847935	0.47726227	0.75150175	0.73211712	-0.0499882	0.3413309
0.40909669	0.42669838	0.62876195	0.11740261	0.21447727	0.14709894	-0.1938088
-0.1989779	-0.5475312	-0.5074736	0.00667491	-0.1077498	0.09495169	0.25649764
0.11177769	0.17963172	0.26834804	0.4101075	0.3788357	0.23368	0.09330556
0.38105242	0.47040088	0.9475439	0.08424557	0.13523245	0.13265499	-0.0061996
0.48968859	0.42711083	0.79786917	1.9995841	2.1662388	-0.0788001	0.59066777
-0.1537993	-0.1193956	-0.0930365	0.31684086	0.73304914	0.00453923	0.22241652
0.3757363	0.34573732	0.6740322	2.16768	1.8528428	-0.3901247	0.68930004
0.16160836	0.12824223	0.21471797	0.36173621	0.27439374	0.0472188	0.04524455
-0.0556151	-0.0044043	0.10607358	0.46507487	0.33733049	0.11431407	0.19748423
-0.0030595	-0.0939241	-0.0644365	0.47296917	0.27656073	-0.2537539	0.26772929
-0.1946733	-0.1530761	-0.2498732	0.25179923	0.19441526	0.07125046	0.28579955
-0.3689546	-0.4786287	-0.5190604	0.17336176	0.84970632	0.19153617	0.44993317
-0.3370037	-0.3694747	-0.4394991	0.90661737	0.87883958	-0.0625202	0.56074232
-0.0057349	-0.0114161	0.02131125	-0.077128	0.11348242	0.08463117	0.30755069
-0.096161	-0.1810152	-0.1949198	0.07117235	0.31483123	0.13117472	0.01280805
-0.2919239	-0.2517554	-0.5327967	0.10898974	0.25940323	0.09876139	0.22091816
0.49693998	0.67859973	1.046947	0.89064317	1.0613644	-0.8077706	0.19701757
0.03589402	0.05258068	0.00246371	0.31842743	0.45169313	0.29856927	0.10268853
0.01815497	-0.0807285	-0.0532431	0.36834024	0.32127137	0.26033416	0.30002627
0.22111176	0.25168606	0.2031746	0.27840408	0.26455466	-0.2215476	-0.002622

0.14833902	0.30415054	0.3265434	0.08411668	0.06850522	-0.0936316	0.14864621
0.26231141	0.30865241	0.31062778	-0.0637694	0.04606833	0.26124377	0.0276387
-0.0076105	-0.0210588	-0.0414064	-0.0432017	-0.0188683	-0.1362447	-0.031322
0.4949499	0.39561117	0.71632588	0.12927669	-0.0376041	-0.0579264	0.02734009
0.10664987	0.10123818	0.03961699	0.27328507	0.1930656	0.11865182	0.25413585
0.35280263	0.31439103	0.63568267	0.46332741	0.49820767	-0.1813661	0.37589064
-0.1605035	-0.0405503	0.05960243	0.77938589	0.51409346	0.14919494	0.19346785
0.2221875	0.32017924	0.20931259	0.40287044	0.15609838	0.31672271	0.06059381
-0.0350607	-0.0096345	0.00202018	0.26822251	0.33117417	-0.3313248	0.2027371
0.14426006	0.16220879	0.22516961	0.32203199	0.22080549	-0.2979002	0.15167486
0.10603173	0.14271067	0.4116536	0.27995837	0.25235304	0.27892744	0.1505379
-0.4591366	-0.6515507	-0.4241007	1.3172286	1.0932067	-0.4160956	0.76719849
0.26485028	0.25193523	0.28239138	0.40996655	0.27574601	-0.0399175	0.05746948
0.2789592	0.21112011	0.23934431	1.0087629	0.64351119	-0.2737819	0.24098533
-0.0006818	0.04627574	0.19323183	0.32668258	0.36549492	0.10248329	0.15125707
-0.0745778	-0.1641333	-0.2120778	0.60772509	0.49987089	0.13903285	0.00952917
-0.0005582	0.13687258	0.03939478	0.13752487	0.20205101	0.13886553	-0.1366344
0.07616924	0.29118146	0.34184697	0.53687103	0.65872705	-0.5217191	0.51783003
-0.0403182	0.09094374	-0.0104805	0.34360054	0.28195587	-0.57133	0.26616786
0.35069731	0.50915795	0.74032543	0.40177187	0.31565802	0.0107838	0.44324381

test_GSE683	test_GSE683	test_GSE683	test_GSE683	test_GSE683	test_GSE683	test_GSE683
ref_GSE6836	ref_GSE6836	ref_GSE6836	ref_GSE6836	ref_GSE6836	ref_GSE6836	ref_GSE6836
GSE6836	GSE6836	GSE6836	GSE6836	GSE6836	GSE6836	GSE6836
GEO	GEO	GEO	GEO	GEO	GEO	GEO
affymetrix	affymetrix	affymetrix	affymetrix	affymetrix	affymetrix	affymetrix
minE_b1174:	yoeB_b4539:	yoeB_b4539:	yoeB_b4539:+1		NORFLOXACI	NORFLOXACI
1494	1499	1500	1501	1547	1549	1550
0.04806109	0.62590498	0.59697676	0.54200044	-0.0704683	0.64140803	0.40708775
-0.0322869	-0.0829117	0.04060907	-0.1482256	0.13699782	-0.095849	-0.095398
-0.2422709	0.32770549	0.77523999	1.061249	0.2629782	-0.4698961	-0.595076
0.04194965	-0.3241279	-0.0415905	-0.0954264	-0.1461967	0.3167614	0.33967399
0.03925111	0.26326885	0.09673683	0.38874847	0.08130398	0.06309633	-0.113737
0.04076476	0.05314901	0.12186306	0.11390106	-0.2306353	-1.3004933	-1.3863256
0.66022785	0.89152353	1.3339895	0.65610515	-0.20517	-1.1415106	-1.2218249
-0.1119325	-0.9128353	-0.8720136	-1.1370776	-0.0575567	-0.4730096	-0.5165202
0.15233169	0.19675782	0.15066156	0.24114766	0.06362056	0.01924427	-0.013468
0.03513557	0.10415059	0.00832775	-0.0332885	0.00578752	-0.5507033	-0.5023215
-0.4813745	0.18899794	-0.053938	-0.0453215	-0.0357718	0.55689089	0.22480756
-0.4478326	0.05192054	-0.4946404	0.15840449	0.06691562	-0.1217159	-0.0826086
-0.3798253	0.24286482	0.08668074	0.38391556	-0.0330216	-0.0833926	-0.1346903
0.24532893	0.86287688	0.64681106	0.80710002	-0.0547333	0.13432478	-0.3670707
-0.3485572	0.41593591	-0.0418466	0.4150759	0.13210925	0.50651345	0.52278091
0.08018725	0.61321005	0.36344399	0.76244693	0.00136644	-0.1577781	-0.3615086
-0.2058762	1.1282082	0.49681694	1.1197128	0.14873369	0.63313567	0.22445804
-0.211771	3.2999559	2.3745114	3.0239901	0.00753999	0.70410488	0.40552719
-0.0204316	0.93642429	0.40533264	0.8646891	-0.0787226	-0.2993539	-0.3391163
-0.0184372	-0.2590399	-0.0816495	0.08325873	-0.0056712	-0.0508266	0.16659552
-0.2098498	0.00330955	-0.2348853	-0.0524316	-0.0259115	-0.2727397	-0.3659635
0.03003763	0.07849418	0.14632454	0.0873396	0.06885636	-0.0281012	-0.1644227
-0.0920908	-0.0095091	-0.2911914	0.08932416	0.04184408	0.33130069	0.1378594
0.01051487	-0.1830059	-0.2326395	-0.0330365	0.06121359	-0.3215881	-0.3167025
0.17736219	0.77322407	0.43458165	0.46791522	0.0807548	0.70527198	0.77376229
0.04752622	0.02338531	0.30574309	0.27370574	-0.0329744	0.505451	0.17875756
0.22529777	-0.2882955	-0.3190947	-0.4938454	0.11874933	0.65440288	0.7227937
0.44503086	-0.1723314	-0.2937164	-0.475566	-0.1967782	-0.5090069	-0.4241625
-0.0038939	0.22708624	0.53417211	0.3614447	-0.0074952	0.0688667	0.06414855
-0.352376	1.0229593	0.75456295	0.73899307	0.02925903	0.40268421	0.12005704
-0.1108005	0.89473205	0.62408099	0.6544628	0.11904297	-0.4121067	-0.465565
0.14850278	0.17853276	-0.0684905	-0.1009575	-0.0540962	-1.1224309	-0.8745591

0.34383112	0.53709225	0.48258173	0.36070389	0.04325726	-0.3864234	-0.5799347
0.03286027	-0.0161667	-0.0157186	0.20837076	-0.0354341	0.0565895	0.00743164
0.15603158	0.05551402	0.01852425	0.02508386	0.02873395	-0.2349837	-0.2329913
-0.0264737	-0.059211	-0.1341911	0.27191906	0.14179105	0.06761749	-0.1869446
0.00841	0.38093083	0.14789334	-0.0200857	-0.0850176	0.18440288	0.4136219
-0.0667279	1.4030215	0.88619199	1.0935913	-0.1280724	0.20781063	0.12423305
0.22894677	0.22855331	0.16301858	-0.0268878	-0.151672	-0.0693707	-0.1355897
0.05195204	-0.07496	-0.0528938	-0.148919	0.13289346	-0.0545644	-0.1482753
0.14820837	0.53399429	0.45259678	0.55614566	-0.1122572	-0.2359881	-0.388453
-0.0604274	0.26018462	0.09042665	0.38011402	-0.0044955	0.09682781	0.01917123
-0.1174326	0.54378025	0.36507542	0.64735766	0.09733143	0.39997256	0.62120856
0.23462858	-0.8514955	-0.93756	-0.9460588	-0.5177827	-0.71396	-0.925092
-0.0727124	0.68245925	0.41228867	0.43243817	-0.1277396	0.43505005	0.49832161
0.05529188	-0.3520729	-0.2724455	-0.4457898	-0.1409218	0.44897374	0.47321276
-0.114555	0.4758933	0.24216498	0.38230313	-0.2550717	1.1686158	1.1815664
0.32656722	1.4605719	1.3190395	1.3937841	-0.0051263	2.734029	2.7365594
0.19006256	0.36084315	0.28746193	0.36154249	-0.0988734	0.71453049	0.24265424
0.05269253	0.71560292	0.40600068	0.39516076	-0.0421194	-0.3048572	-0.5719312
0.10841965	0.50687005	0.29555827	0.27414832	-0.0438283	-0.5232956	-0.3673339
0.02919137	0.56403286	0.24962307	0.6868687	-0.0790948	-0.1085358	-0.1352253
0.20610577	0.27598447	0.12222007	0.24402669	-0.0051022	-1.0657214	-1.0324169
0.04808549	-0.1945089	-0.12141	-0.165374	0.086599	-0.0786878	-0.3027588
0.12441561	1.1505498	0.65137262	0.89248466	-0.0257835	0.39241459	0.2864766
-0.2562844	0.82698274	0.50017828	0.73878924	-0.2378583	-0.0650106	-0.0951557
-0.0082444	0.19740525	0.07854765	0.06052196	0.14455392	-0.5114269	-0.7568259
-0.047447	-0.130728	-0.2154039	0.06229915	0.01754144	-1.0137006	-1.2051042
0.05950448	-0.0938707	-0.2433986	-0.2857539	-0.2876158	-0.5723692	-1.437995
-0.0105747	-0.07779	0.1048504	9.19E-05	-0.0873207	-0.210647	-0.3784991
0.23148744	0.6709239	0.34573228	0.49163231	-0.0280393	0.64008467	0.19682695
-0.3212408	2.051234	1.9691274	1.8683093	0.02331483	0.07716371	0.22267438
0.06440036	1.4298918	1.6600687	1.335479	-0.2782767	-0.2735615	-0.6814881
-0.0607417	0.58614083	0.28934049	0.55133665	0.11331284	-0.019122	-0.1781526
-0.0639622	0.77597278	0.28729193	0.58102528	-0.0474828	0.22774575	0.32585581
-0.2161305	-0.1904803	-0.1600151	0.01283079	0.02562137	0.20121445	0.15699914
0.06722891	0.83459386	0.37549409	0.55345212	-0.2398156	-0.1074683	-0.3441283
-0.1032482	0.48147674	0.38156342	0.34115214	0.28385822	1.1393117	0.99103279
-0.3545088	0.07315902	-0.2620683	-0.1626547	0.00825841	-0.6237167	-0.5251624
-0.415294	-0.9711251	-0.3835548	-0.5341566	-0.1042708	0.01628328	-0.0864185
-0.2165475	1.2024441	0.74248263	0.79559123	-0.4332075	-0.8428304	-0.7763903
0.09020501	0.06889227	0.07459227	-0.097686	-0.0498945	-0.4490302	-0.5315528
0.06198664	0.58760046	0.17225567	0.34102337	-0.004438	-0.1344664	-0.0828167
-0.1489372	0.27222444	-0.0449869	0.16477625	-0.0306764	0.46901714	0.06991422

-0.1018846	0.13937359	0.0210458	0.16368401	0.13315025	-1.6498672	-1.5800823
-0.0452017	0.67662344	0.45743045	0.23840213	0.06923058	-0.4715665	-0.4259576
-0.0608837	0.18618367	0.14657244	0.03093565	-0.2125295	-0.762504	-0.755071
-0.0606288	1.9877141	0.7424968	1.1780767	-0.0019262	0.292708	0.51426307
0.04748208	0.07652565	0.14801775	0.05990611	0.0148744	-0.0740283	-0.0983604
0.01534486	0.79648	0.25384677	0.34073999	-0.0021599	0.2929298	0.22708792
-0.0687472	0.89979413	0.35115127	0.58987231	-0.0520632	0.08724184	0.01022675
0.25066975	0.43022297	0.37380457	0.28028001	0.11087689	-0.2873876	-0.249232
-0.065344	0.51247719	0.16863172	0.30849894	-0.2994428	-0.5643336	-0.7251118
0.23617779	-0.170743	0.06278719	-0.5391067	-0.0834425	0.15310077	0.10971943
0.14210823	-0.2063177	0.02607102	-0.5194178	0.00868806	0.08964902	0.09216648
0.10296666	0.24461716	0.12781646	0.16396324	-0.1318959	-0.2521463	-0.1769484
-0.0928357	-0.2206128	-0.1641447	-0.1691281	-0.0948187	-0.2480532	-0.2037965
-0.0640583	-0.1365277	-0.1847956	-0.3302117	-0.0545068	-0.2546958	-0.4342716
-0.0652317	0.99274904	0.24377051	0.90407338	-0.0495714	0.69450388	0.93780911
-0.073085	1.1267822	0.51576188	0.73503805	-0.0972929	-0.4622953	-0.2777455
-0.1422289	0.23961953	0.0038752	-0.1706541	-0.0777092	-0.1826262	-0.2011531
-0.3550865	0.12090185	-0.0832351	-0.2852037	-0.0307582	0.07770417	0.22776237
0.13825528	1.0560962	0.31778319	0.76506696	-0.1485128	0.66122325	0.68062611
-0.0042591	0.44241042	0.21094056	0.19633151	-0.1877641	-0.5824969	-0.6672232
-0.1066853	0.1730691	-0.0653345	0.00864114	0.06003641	-0.0437561	0.12311557
-0.0480611	0.36767785	0.17547688	0.28273936	-0.1300345	-0.2321173	-0.2898879
-0.0452078	0.92191463	0.47002045	0.6738538	-0.0348474	-0.7259667	-0.9887523
-0.4558744	-0.539656	-0.5492441	-0.4681575	-0.0724165	-0.3863664	-0.5297582
0.06270491	-0.013501	0.02558174	-0.1940689	0.04968915	0.07156358	0.02979714
0.20360593	1.7781785	1.1832663	1.2418002	-0.1155569	-0.492793	-0.3746009
0.16199977	1.3708202	0.66379689	0.94306904	0.13336464	-0.93254	-0.7428069
-0.0568461	0.70350774	0.40770344	0.42042805	-0.2436733	0.06112103	0.03389057
-0.33954	1.0746	0.77726	0.59237	-0.054256	-0.0041061	-0.21486
-0.20686	1.6169	1.5437	0.8011	-0.093359	-0.17491	-0.11314
-0.06476	2.475	1.9603	1.9377	-0.16458	0.12025	-0.078404
-0.21637	1.0531	0.31776	0.60705	0.061476	0.61244	0.62609
0.027072	0.87185	0.2107	0.67686	0.15073	0.65981	0.82272
0.019523	-0.042954	-0.045837	-0.24403	-0.03496	-0.083286	-0.11143
0.047234	-0.24619	-0.16776	-0.38573	-0.18269	-0.17934	-0.010166
-0.275709	0.50646957	0.59355388	0.4520369	-0.158026	-1.2830259	-1.2249694
0.12686858	0.14270509	0.06363414	0.22893078	-0.0354612	0.17883878	-0.1297176
-0.1814201	0.4177025	0.09494427	-0.0118824	-0.1894124	-1.2727949	-1.0727858
0.00368985	0.23877503	0.16806409	0.02864027	0.03477975	-0.4073579	-0.215489
0.15595368	0.49718491	0.37658296	0.20398694	0.00302033	0.12611186	0.10396714
0.11527115	1.4860286	0.87335786	1.0231789	-0.4271522	-0.3203363	-0.7226648
-0.0622999	0.4381224	0.4119912	0.3159518	-0.049932	-0.7202707	-0.6046163

0.28588124	0.45581491	0.28836356	0.23798921	0.03382324	-0.210935	-0.0970649
0.13896809	0.92266152	0.79651222	0.79690497	0.18124029	-0.0579991	-0.0694099
0.0665614	0.48924151	0.2059581	0.24466922	-0.0428096	-0.0740548	-0.2008587
0.08687156	0.12100553	0.14602187	-0.0899118	-0.0077807	-0.0126315	0.04768762
0.01162167	0.00500519	-0.0479196	-0.1404146	-0.1038168	-0.4372206	-0.675412
0.28056532	0.10018721	0.04389533	0.04699374	0.05691888	-0.2097208	-0.2218392
-0.1117279	0.00377582	-0.0374853	-0.240269	-0.037197	0.12846234	-0.1482114
-0.104105	1.2035525	0.4716386	0.62799341	-0.2153549	-0.2556474	-0.3244821
-0.0401953	0.61467307	0.37085848	0.36901315	0.03058373	-0.3056569	-0.3210147
-0.0110066	-0.1419184	-0.2505894	-0.1840309	-0.0944547	-1.3170004	-1.2175261
-0.2175358	-1.4561165	-1.9654726	-1.8136333	-0.1763873	-0.4899302	-0.4057959
0.16905515	0.55056502	0.45273327	0.49171089	0.058396	-1.0781039	-0.8725918
-0.2441245	-0.770782	-0.4809022	-0.5462207	-0.0989686	-1.9399105	-1.8924728
-0.0799226	0.66416975	0.68922167	0.79556427	-0.0625829	-0.2191887	-0.2426866
-0.1772126	1.2175534	1.2182445	1.4212615	-0.2912688	-0.817217	-0.7073449
-0.1091516	-0.0441718	-0.256384	-0.1110585	-0.3063701	-0.0751368	0.05007309
-0.4196152	1.0618109	0.42944127	0.63339567	-0.0712314	-0.8907153	-0.8803104
-0.1678077	1.5326013	0.33993809	0.97456664	0.02187731	0.15881596	0.03872203
-0.5323428	1.0712371	0.62236361	0.90576902	-0.2355986	-0.2530677	-0.175891
-0.0283943	0.39080993	0.06865117	0.11369052	0.12089696	0.07419104	0.13336372
0.25510163	0.18934804	0.19451107	0.06335825	0.09753066	0.15354941	0.26038842
0.1139994	1.2056326	0.8976586	0.99407156	-0.0680421	-0.0614644	-0.0318583
-0.06725	2.304	2.2854	2.3591	-0.041932	-0.30758	-0.24498
-0.3928414	0.98430799	1.0920433	0.90479948	0.11268391	-0.3697494	-0.3461133
0.04122316	-0.022868	-0.058322	-0.0192642	-0.0440327	-0.2513884	-0.3602428
0.32876502	0.44441639	0.2515343	0.35397061	0.0496676	1.673495	1.6706427
-0.1253466	0.97756626	0.65363414	0.64371115	0.0573556	-0.2819966	-0.1775635
0.13550809	0.93722357	0.68673433	0.82781249	0.1849458	-0.1285972	0.27642305
-0.1728258	0.04222896	0.09225784	0.08340395	-0.1609065	-0.667191	-0.4598559
0.13741329	0.16567959	0.09636912	0.07831173	-0.0100142	0.05991909	0.16803077
-0.1436947	-0.0269803	-0.010592	0.36359753	-0.041115	-0.4444014	-0.4467582
0.06412498	0.11134243	0.0220324	-0.0068948	-0.0364152	-0.1712512	-0.1777188
0.18612766	-0.256316	-0.2216748	-0.2960671	-0.0065725	-0.0673589	-0.0426245
0.18891789	0.29081174	0.1587213	0.22568402	0.04684004	0.14152173	-0.0354637
-0.1847645	0.41357073	0.32774917	0.32866327	0.08699211	-0.9004786	-1.002508
0.20104775	0.53484637	0.47855944	0.20828828	0.13817946	-0.0814039	0.09616185
0.04463788	-0.3279641	-0.3391045	-0.4394946	-0.0065397	-0.4262673	-0.5354225
0.08256963	-0.0626832	-0.0102298	-0.1530498	0.06156949	0.02391319	-0.1554326
-0.011968	-0.3212061	-0.1315893	-0.320786	0.06136117	0.01510536	0.04061308
-0.0152627	1.1100989	1.1185552	1.1334352	0.13944148	0.29024339	0.39599866
-0.1154108	-0.1449157	-0.0556725	-0.0773772	-0.0736941	-0.181714	-0.0875294
-0.5840449	0.25506123	-0.1262489	-0.0988141	-0.7136689	-0.4060047	-0.6911792

0.12657534	-0.0101863	0.04434084	-0.0306878	-0.0845769	0.14413593	0.25643644
0.06651301	1.2225763	0.83059553	0.92547752	0.09734168	0.18150815	0.30598533
0.03226578	0.10339243	0.09752962	0.06156288	0.01725296	0.24707188	0.03321177
0.07204336	0.12498686	0.29074564	0.2087585	-0.0042117	0.22943879	0.15520648
0.06376754	0.08084635	0.0905492	-0.0048644	0.18253399	0.12103959	0.41337996
-0.0008596	-0.2008847	-0.2593064	-0.1103209	-0.0003884	-0.0369002	0.01761341
0.07280877	-0.0244728	0.13502514	-0.1294972	-0.2132156	-0.7469536	-0.7569404
0.20363932	1.0631148	0.50934964	0.60176055	-0.0431838	1.1775849	1.2096426
-0.1493034	-0.1626378	-0.1107018	0.16106024	-0.1035687	-0.355514	-0.0318111
-0.1056431	0.23046436	0.10702843	0.25920159	0.0334871	-0.0365721	-0.1019981
0.16797513	-0.0298575	-0.0022645	-0.0855716	0.09736189	-0.0265364	-0.1361727
0.03226044	1.2110782	0.97985069	1.0445245	-0.0849856	-0.8057856	-0.719438
-0.1913716	0.09324229	-0.0187316	0.03811797	0.11916626	-0.1160687	-0.0734754
-0.131443	-0.4207982	-0.322669	-0.1131032	-0.0186806	0.14537938	0.41240631
0.05765717	0.18749127	0.12426323	0.34067349	0.08164481	0.06354661	0.33342463
0.07844183	0.32172091	0.22494605	0.17911973	-0.0006735	-0.2209169	-0.2221283
0.25036898	0.79740519	0.44882178	0.85083874	-0.1397099	0.31199958	0.30314016
0.09449727	-0.3934742	-0.4754051	-0.4997714	-0.015229	0.12119315	0.02430994
-0.2938436	0.02873209	-0.0834973	-0.0346541	-0.0028236	-1.1005039	-0.8386945
-0.2939144	-1.5432001	-0.7854898	-1.1840342	0.02543266	-0.268546	-0.2182661
-0.3308285	-1.0662552	-0.5990112	-0.7354157	0.09692095	-0.0724112	0.05727186
-0.1664335	0.40809057	0.34106522	0.45240912	-0.0051145	0.05555692	0.01313716
-0.1149238	-0.090522	-0.0670458	-0.1374621	-0.1283036	-0.026523	-0.0412262
0.08056036	-0.211128	-0.2827354	-0.0920258	0.00794864	0.0764512	0.07349519
0.13143859	-0.239634	-0.1143852	-0.0012791	0.01486285	0.43884023	0.38806434
-0.1522851	0.14939879	0.06366069	0.3660427	0.20113336	-0.0872433	0.06233319
0.12034074	0.14084077	0.14726906	0.11903254	-0.0133954	0.60866511	0.58002915
-0.089653	-0.3604501	-0.2882743	-0.2356939	-0.1497611	-1.4764802	-1.3378392
-0.2048857	0.84599095	0.58553515	0.73108224	-0.0929859	-0.3963817	-0.2152615
-0.0381968	0.51540144	0.17022845	0.60880946	-0.3725215	-0.6791221	-0.6566916
0.16189551	0.16449317	0.23154928	0.02217975	0.03343553	0.06140042	-0.0045708
-0.1971549	0.35506624	-0.0183499	0.36893474	-0.219912	-0.0914069	-0.0296859
-0.038409	-0.6518893	-0.4207176	-0.6489046	-0.0608704	-0.0607368	-0.05606
-0.0026494	-0.0747739	-0.1199791	0.02899336	0.06254306	-0.1003674	-0.0830149
-0.3963861	0.29327828	0.16104202	0.18394392	-0.2412314	-0.3990923	-0.3069622
-0.2197572	-0.380872	-0.392294	-0.3641057	-0.0745732	-0.5440694	-0.5737603
-0.208128	0.1649617	-0.0404183	0.3271597	-0.0113996	-0.2767304	-0.3333625
0.05643389	0.42130889	0.46738347	0.26504045	-0.0417224	-0.0413206	0.19248132
-0.3690417	1.2893581	0.3647454	1.2759266	0.13160051	0.86422264	1.0574132
-0.1494342	-0.2529114	-0.2565459	-0.1143655	0.02057574	-0.0455103	-0.0237486
-0.2201108	1.7003499	0.82263856	1.3320246	-0.0013986	-0.4015383	-0.3127039
-0.1017391	2.0247699	1.0920379	1.8019317	0.02140875	-0.6238072	-0.5167648

-0.0370406	-0.1674633	-0.2852841	-0.0707532	-0.0448078	-0.0996173	-0.0133296
-0.1251104	0.74046413	0.17749098	0.67538101	-0.1515546	-0.2346957	-0.244182
-0.093179	-0.0840589	-0.3252094	-0.176337	0.20090617	0.03436416	0.29342843
-0.1660843	0.02158396	0.19910388	0.12163835	-0.0690691	-0.4332526	-0.1920241
-0.3006151	0.47721728	0.16552329	0.58711687	-0.015377	-0.1261291	0.11334409
-0.001477	0.54691722	0.47958304	0.75554594	-0.2088554	-1.0956819	-0.9827084
0.01297945	0.20479733	0.23853387	0.23681788	-0.1199235	-0.60971	-0.5882108
-0.4061791	-0.5618981	-0.1974063	-0.2451753	-0.1500566	-0.4646829	-0.3821553
-0.2539927	-0.562374	-0.5315914	-0.0940917	-0.0094022	-0.0244885	-0.2003555
-0.3605195	0.41008805	0.06256586	0.5095532	-0.0885783	-0.8643935	-1.1560639
0.04273102	0.74268022	0.61592549	0.68609691	-0.0325815	0.36028443	0.49818639
-0.0071418	-0.2721629	-0.1130804	-0.1706739	0.06167695	-1.3316596	-1.2349386
-0.0245601	0.26200753	0.24292316	0.20895774	-0.0335646	-0.1640727	-0.2175619
-0.079457	0.5453924	0.20846144	0.34358226	-0.0310088	-0.4343985	-0.4308138
-0.0938203	0.09445482	-0.112953	-0.1902276	-0.1759546	-1.2587913	-1.1015555
-0.1211871	-0.0881836	-0.2688034	-0.1389944	0.05957233	-0.0897109	-0.0205343
-0.096147	1.3701154	1.3087744	1.2879311	0.02383516	-0.553842	-0.4868147
-0.2876681	-0.6284158	-0.4844623	-0.3611085	-0.2369689	-0.140373	-0.1926214
-0.316622	0.11783273	0.14981776	0.32298458	0.05989081	-0.9164874	-0.8814294
-0.1205831	-0.410718	-0.3410078	-0.2742498	-0.0574485	-0.2598607	-0.2681714
-0.2692746	-0.4944987	0.02722506	0.06405953	-0.0780998	-0.5267521	-0.3856723
0.05100589	-0.0744869	-0.1096618	0.0277132	0.06263275	0.16547907	0.35985952
-0.0366934	0.55519229	0.34425821	0.41145664	0.22369073	0.59820069	0.64707593
-0.063097	-0.4403734	-0.1946552	-0.3231329	-0.1614601	0.21417379	0.38804639
0.05420654	-0.0027582	0.07959218	0.11951768	-0.0946744	-0.0462159	-0.0097817
-0.0887662	-0.0047799	-0.0623453	-0.1093074	-0.0861319	0.24204609	0.40340925
0.11757544	-0.3593928	-0.5663737	-0.165394	-0.1097522	-0.0426642	0.10779665
0.34930539	-1.2490172	-1.2499393	-1.1967196	0.13345211	0.24978702	0.47722822
0.26137025	0.11246535	-0.2985765	-0.1196505	-0.2891796	0.311234	0.27241852
0.31671752	-0.5754665	-0.6070567	-0.711435	-0.2693433	0.04701434	-0.0282183
-0.543233	0.02238067	-0.167625	0.19154035	-0.0155439	-0.0197222	0.05631648
-0.0421988	-0.1310758	-0.1009678	0.15329766	-0.1778581	-0.4957432	-0.6552825
0.38490018	-1.4002265	-1.5067798	-1.0039038	-0.041821	0.25199213	0.29428877
-0.0767734	0.32767535	0.49282453	0.38496542	-0.7231439	-2.4775164	-2.2071315
-0.1867582	-0.2052905	-0.199652	-0.1520086	-0.0222179	-0.1421341	-0.1180513
-0.1722845	-0.0765043	-0.0131191	0.05489021	-0.0363193	-0.0363315	0.31326309
-0.2442469	0.4300134	0.09275062	0.23696376	-0.0293123	0.50192409	0.59180059
-0.0342736	0.45974301	0.40654721	0.49164092	-0.0954265	-0.2556018	-0.205727
-0.7019793	-0.3874786	-0.4005023	1.122042	-0.034104	-0.0196552	0.06888398
-0.2976942	-0.2934027	-0.3471615	-0.0781999	-0.1750535	1.2595069	1.3217149
0.10634307	0.51932857	0.27893306	0.59314957	0.0613544	0.14857529	0.10929848
-0.2736796	0.20581981	-0.0824346	0.3751127	0.11430629	-0.1155101	-0.1453463

-0.3179618	-0.3967794	-0.4827252	-0.1493395	-0.0186238	0.02122153	0.1157866
-0.034797	-0.35198	-0.29644	-0.25927	0.039462	-0.24381	-0.18931
0.09672105	0.32551816	0.40784194	0.24306327	0.06072224	1.6691517	1.6124721
-0.2127843	-0.5403393	-0.4493662	-0.5126312	-0.2395791	-0.4583373	-0.5453234
0.11113448	-0.0791741	-0.2086112	-0.1827851	-0.119584	0.22507169	0.1888412
-0.3950339	-0.5123438	-0.463038	-0.3470907	-0.0882506	-0.1023354	0.12973207
-0.0285076	-0.5731776	-0.2980228	-0.7133967	0.04918816	0.65865192	0.76250945
-0.17756	-0.24828	-0.18927	-0.43165	0.088556	-0.66041	-0.45759
-0.239827	-0.0882577	-0.01681	0.20490955	0.09564238	-0.3969108	-0.2969265
0.11458088	0.28868584	0.10472836	0.08419489	-0.1237767	-0.0400828	0.07935337
0.03907773	-0.4634696	-0.2858569	-0.2619768	-0.0183535	0.27106672	0.17166159
0.02099566	-0.216217	0.00557383	-0.1000707	-0.1750126	-0.6121019	-0.5884273
-0.14354	-0.0588653	0.01183537	0.18645038	-0.189657	-0.6927574	-0.3295419
-0.2123835	-0.3367592	-0.3880404	0.04179455	0.08184996	0.12382913	0.36874511
-0.0899126	-0.3073537	-0.2925834	-0.2945124	0.02605467	-0.0134371	-0.0775135
-0.0693839	-0.4571287	-0.5948694	-0.0439507	-0.0767819	-0.0612388	-0.1875909
-0.0238292	-0.8520489	-0.7923955	-0.8427911	-0.1026464	-0.8148168	-0.9476041
-0.537378	-0.8379004	-0.5981098	-0.2512505	-0.204551	-1.4858891	-1.4632954
0.19022995	0.39951976	0.19132526	0.37887063	-0.2040069	0.24176536	0.17128298
0.12370513	0.03557766	0.17161628	-0.0167458	-0.0987921	-0.4063583	-0.3171516
0.09647325	0.27556984	0.18092926	0.26394677	-0.0358314	1.0880117	1.1483048
-0.0813401	-0.2513478	-0.0600173	-0.2276161	-0.0860685	-0.7767436	-0.6704172
-0.1121228	0.06869065	0.14819478	0.12810126	0.13641002	-0.2862855	-0.4498289
0.28964463	-0.3749197	-0.3808713	-0.2963844	-0.1296698	-0.9878727	-0.9765808
-0.07886	-0.0325309	-0.026879	0.18968318	0.06202171	0.0381445	0.16144296
0.00744655	1.0761311	0.72451217	0.96744634	-0.0527187	-0.6159515	-0.4517136
-0.1065842	-0.0666949	0.02629112	0.18476454	0.15185831	0.99540111	1.0860445
-0.0272361	0.01082536	0.0798337	0.02816758	0.23634564	-1.0227826	-1.0318854
-0.1354825	0.22903071	0.16553201	0.3712175	-0.0444612	1.2443353	1.1805872
-0.1589931	-0.084742	-0.0923486	0.02455816	-0.029266	-0.0214474	0.08800091
-0.205232	-0.5818221	-0.2660569	-0.2350566	-0.1354274	-0.315455	-0.2549644
-0.0643242	-0.4391267	-0.5847063	-0.0252249	-0.0384551	0.09323128	-0.0441684
0.03516214	-0.3856374	-0.2907014	-0.1592734	0.03171443	-0.397961	-0.2638571
0.46932386	-0.3240509	0.30391827	0.09850564	-0.1970462	0.14990168	0.12026856
-0.3041273	-0.8333705	-0.5791637	-0.4018345	0.33968165	-1.3751423	-1.2815992
0.14300294	-0.0094958	0.19352146	-0.0281604	-0.2414404	0.03724024	0.00552364
0.27033001	0.4243111	0.29617517	0.43010245	-0.0652488	-0.2592345	-0.1928347
0.51765081	-0.468934	-0.2375124	-0.3500976	-0.1586493	0.95561196	0.92825451
-0.1214651	0.9290921	1.0326752	0.5478852	-0.1076918	-0.2159578	-0.1791095
-0.0125137	-0.2567197	-0.2614176	-0.1294491	-0.0495121	0.00520969	0.24064587
-0.2585395	0.12195083	0.02938247	0.1395481	0.20493794	-0.8254921	-0.7561467
0.07014531	-0.0894528	-0.0731743	-0.1014839	-0.0116082	0.23988288	0.09649369

-0.0655646	0.39244397	0.22389434	0.13671326	0.17963003	-0.3360687	-0.3259286
0.17347453	0.50849212	0.37721179	0.59517671	0.06285408	0.41649875	0.48326455
0.03003611	-0.0429402	-0.0858538	-0.1750033	0.01907209	-0.0455029	-0.0335807
0.03085261	0.0198224	0.07619938	-0.0494113	0.00158735	-0.2978945	-0.2615805
-0.0300707	0.1506443	0.12786538	0.23327334	0.04696035	-0.085841	-0.1366282
-0.2045987	0.70338121	0.71699422	0.65394598	0.00038238	-0.3238931	-0.1216783
-0.5350283	0.76157573	0.2457091	0.77466317	-0.1020348	-0.0726386	0.00530389
-0.2170898	-0.1332075	-0.3317013	0.13153479	0.01252119	0.08756504	0.04980453
0.05101935	0.04975745	0.06314726	-0.1118309	-0.0422638	0.06644809	-0.0017353
-0.3316122	0.22862214	0.13960868	0.18789107	0.00022241	-0.2652223	-0.1686285
-0.167842	0.20685072	-0.0412454	0.24644751	0.07083701	-0.0532191	0.11191607
-0.6241812	-0.7519677	-0.6599058	-0.2413272	0.21114859	-1.5278117	-1.5165919
-0.0961717	1.2151062	0.44347207	0.99706892	0.03394501	0.08558985	0.10853896
-0.4472573	0.71142424	0.08278424	0.66218915	0.11205116	-0.0333199	0.02209198
-0.2495111	0.17410297	0.15975324	0.1272195	-0.1672269	-0.0247803	-0.0805007
-0.329579	-0.6467737	-0.6542565	-0.5794441	0.00863537	-0.1245489	-0.0522939
0.09003853	0.46274926	0.29569435	0.46473807	-0.0855088	0.45674703	0.50260303
-0.1293211	0.14476362	0.19044376	0.15622114	0.244042	-0.3455945	-0.4243707
-0.1654507	0.20103858	0.09351117	-0.1495149	-0.241866	-0.2261627	-0.3751619
-0.0320065	0.18198408	0.13693234	0.42498627	0.05580723	0.7275688	0.63627731

test_GSE683	test_GSE683	test_GSE683	test_GSE683	test_GSE683	test_GSE683	test_GSE683
ref_GSE6836	ref_GSE6836	ref_GSE6836	ref_GSE6836	ref_GSE6836	ref_GSE6836	ref_GSE6836
GSE6836	GSE6836	GSE6836	GSE6836	GSE6836	GSE6836	GSE6836
GEO	GEO	GEO	GEO	GEO	GEO	GEO
affymetrix	affymetrix	affymetrix	affymetrix	affymetrix	affymetrix	affymetrix
NORFLOXACI	ccdB:+1,recA	ccdB:+1,recA	ccdB:+1,recA	TIME:180	GLUCOSE:12,	GLUCOSE:12,
1551	1593	1594	1604	1610	1617	1618
0.56285209	0.32526034	0.15334791	0.35710326	0.07523884	-0.0522333	0.29076985
0.00418922	0.00469688	-0.3097657	-0.1740447	0.99166468	1.2350283	0.41662845
-0.8330644	0.06851025	-0.0235954	0.55336955	0.85075936	0.83241389	0.62474044
0.24467196	0.01382865	0.09522267	-0.1339433	1.1446191	0.96440088	1.0825378
0.07162913	-0.1406041	0.04086158	-0.066784	0.25037221	0.15815582	0.18357446
-1.1714798	-0.3142317	-0.058707	0.0340802	0.58250005	0.3578534	0.77759008
-0.8165112	0.71770991	0.42561133	0.27875162	0.17132569	0.1874159	0.05164921
0.1769787	0.0780798	-0.0510868	0.00532709	1.5291454	1.1446585	1.384681
0.07435001	-0.0665126	-0.1197973	-0.1025793	0.03882107	0.09649354	0.0413446
-0.6313931	-0.2838214	-0.0799585	-0.0471944	0.65063187	0.60695676	0.86547209
0.59891511	-1.1550615	-1.2748252	-0.9499541	0.10779576	0.19000784	0.15347073
-0.1348294	0.37305399	0.20051131	-0.3895491	1.5939763	1.5644137	1.6142399
-0.047816	-0.1849045	-0.1575225	-0.2315708	0.34085187	0.28681778	0.39732295
0.07694339	0.29788342	0.28120368	0.66124563	-0.0128623	0.0152634	0.00772826
0.50239503	0.07597486	0.10184166	0.18892036	0.09389442	0.03655322	0.06149897
0.11760224	0.01124252	-0.0570079	-0.2380684	0.11428413	-0.0170963	0.25968151
0.62032043	0.95198341	0.96083552	0.55662177	-0.361205	-0.3060806	-0.4284251
0.74261043	0.43121241	0.42082726	0.2792807	-0.5178831	-0.4246964	-0.4972229
-0.3705036	-0.1094314	-0.1513346	0.07967627	-0.1036566	-0.1636281	-0.0732108
0.13938808	-0.179126	-0.1491926	-0.2121181	0.08280394	0.12123111	0.08584303
-0.0228607	0.08167185	0.15917327	-0.0047186	-0.0050156	0.10037596	0.15652102
0.08737237	-0.0733588	-0.091006	-0.1394997	0.62605197	0.8012294	0.98902023
0.37111341	-0.1248552	-0.1580654	0.03670009	0.33377456	0.21039055	0.37226961
-0.3227186	-0.3165541	-0.2350334	-0.075762	0.39495011	0.27252647	0.3140323
0.37104803	0.16065947	0.20134393	-0.1132597	-0.0860178	-0.1970431	-0.1186828
0.6797313	0.25825845	0.18801812	0.17845184	0.55500759	0.62936192	0.34150963
0.51245657	0.48614309	0.20739396	-0.0075129	0.09128895	-0.0734617	0.52512283
-0.3531974	0.0293959	-0.5706202	-1.0558688	0.03910329	0.49233907	0.02908313
-0.0306717	-0.4533281	-0.5925694	-0.7032534	-0.0700568	-0.057034	-0.062768
0.36099464	-1.4502947	-1.5247374	-2.2029691	0.20636117	0.59211025	0.52782617
-0.4074357	-0.1814114	-0.4147233	-0.1161413	-0.7557701	-0.7793574	-0.73206
-0.9938936	-0.0667978	-0.15886	-0.0889603	1.1339202	0.73509821	0.99447206

-0.242198	0.15478508	-0.0104752	-0.2607113	0.98061607	0.8305791	1.5426053
0.03235393	-0.0973707	-0.1480526	-0.1274426	0.14679828	0.0188699	0.13800836
-0.3503209	-0.1967953	-0.3034994	-0.2532234	0.65501706	0.5518128	1.116119
0.11258147	-0.1160021	0.01046205	0.08356098	-0.0938614	-0.0664897	0.05501184
0.19929764	-0.0037561	0.03721979	-0.2751112	0.01144793	0.06484166	0.05833373
0.2533141	-0.431951	-0.2701957	-0.4131979	0.39480385	0.23338174	0.523312
0.03068001	-0.9831442	-0.9168371	-1.3606622	0.38783645	0.55306897	0.08388072
0.02357955	0.01162376	0.11129181	-0.1037618	0.19904078	0.13843639	0.17262665
-0.0826119	-0.2367054	-0.1602001	-0.1596041	-0.2579556	-0.0437049	-0.2033393
0.14556632	-0.0803666	-0.1118955	-0.2755652	0.10223386	0.0577261	0.15065816
0.41200257	-0.6827183	-0.4886975	-0.4981818	0.41171299	0.25958257	0.39705818
-1.0931794	-1.6778335	-2.4071427	-2.315615	-1.2693012	-0.6520967	-2.0156414
0.47113605	-0.0817222	-0.0240339	-0.298204	-0.1857818	-0.1728125	-0.2094597
0.32884706	-0.5648638	-0.6359158	-0.7374352	0.28419656	0.48643775	-0.0575069
1.3832794	-0.9149245	-0.8729744	-1.1965496	0.2183096	0.53916223	0.12302885
2.9106769	0.59001941	0.60520367	0.44138609	-1.3510032	-0.8885704	-1.2522543
0.74513654	-0.0144366	0.01105432	-0.1284789	-0.0460405	-0.0551198	0.01724283
-0.2524953	-0.6093363	-0.6440421	-0.4853433	-0.2145164	-0.2437508	-0.0066098
-0.4656628	-2.3998349	-2.093596	-2.5643447	-0.0589922	-0.1187397	0.17550748
-0.0103369	-4.0208037	-3.7460114	-3.9059974	0.05794715	0.07208041	0.16249858
-1.1283639	-0.9929925	-0.9028554	-1.1898116	1.0177833	0.48806546	0.81229928
-0.0357688	-0.1763455	-0.2850185	-0.477535	0.18194831	0.18054445	0.17806226
0.50554254	-0.9555603	-0.8077351	-0.7798075	0.94660588	0.94812634	0.71404513
-0.103474	-0.8883768	-1.0609017	-1.158542	0.05901611	0.19129381	0.21801284
-0.3724235	-0.5259695	-0.30967	-0.2298847	-0.0291043	-0.1528726	-0.1033015
-0.8252731	-1.0881465	-1.1164565	-0.6327811	1.2498409	0.97838155	2.2957748
-0.531047	-1.8600453	-1.6939829	-1.4787388	0.30010048	0.16902041	1.3132386
-0.2388116	-0.1134126	-0.1508824	0.11643489	0.25322303	0.24589537	0.25373032
0.56655006	-0.3695026	-0.4241554	-0.6639091	0.1273657	0.1642959	0.92576595
0.22829511	-0.5147231	-0.0683166	-0.0132032	-0.4309869	-0.5033204	-0.2165465
-0.5499247	-0.31466	-0.5281594	-0.6392268	-0.7119041	-0.6728798	-0.6188497
-0.0233765	-0.1521766	-0.1018096	-0.1797413	0.42100534	0.51686801	0.5914733
0.11073152	0.19611959	0.18640326	-0.0690508	0.08946505	0.18330857	-0.0137173
0.27471353	0.09098846	0.04135574	0.01875845	0.21521131	0.21470656	0.14807932
-0.1565071	-0.1308459	-0.0003947	0.04094704	-0.10235	0.01614684	-0.1312939
1.1274506	0.37222018	0.35514505	0.21347073	0.88745043	0.58551649	1.056597
-0.4891356	-0.4658559	-0.5738004	-0.6603607	1.9615337	1.2244358	2.2150034
0.08658193	-4.7661618	-4.4653017	-3.3832637	-0.1986251	-0.0596316	-0.0394104
-0.7240204	-0.6332695	-0.643565	-0.7622425	1.4988928	1.5406442	1.708982
-0.3576379	-0.1502928	-0.1516149	-0.1041443	-0.5107047	-0.3627183	-0.614666
-0.2893628	0.06395508	0.11641785	0.02876339	0.18073896	-0.0282253	0.33547441
0.47612916	0.134649	0.08231023	0.02841674	0.75852097	0.66340646	0.26175079

-1.3177278	-2.0588162	-2.4243519	-3.2468257	0.71833587	0.69725467	1.0778964
-0.5550724	-0.1257416	0.02817819	-0.020693	0.68678331	0.17410252	0.61235482
-0.7368485	-0.1490824	-0.1841073	-0.3740908	-0.1372749	-0.1919081	0.0917109
0.30680882	-0.0315779	0.06516481	0.1630727	-0.0369387	0.03517431	-0.0987499
-0.1690394	-0.1334357	-0.1127039	0.15690023	0.43378315	0.08838997	0.46579152
0.21832734	0.0822149	0.06102037	0.01739587	0.24366348	-0.0798186	0.44705785
0.06434804	-0.0208667	-0.0837285	-0.0286716	-0.1566791	0.13337643	-0.1403265
-0.1706341	-0.1269529	-0.2901057	-0.1428929	-0.2929287	-0.3180769	-0.1630359
-0.5824871	-0.0494947	-0.0963089	0.05891366	-0.1658872	-0.2605036	0.11804246
0.32159618	0.05088792	0.03092505	0.03175429	-0.2716194	-0.1216795	-0.639241
0.01470534	0.02905894	0.07170969	0.07544344	-0.1604127	-0.2139682	-0.2183794
-0.2087182	-0.025646	0.02158632	0.0781233	0.08891928	0.07108676	0.14984292
-0.316397	0.01072684	-0.0377311	0.07932362	0.16049154	0.12916219	0.03941022
-0.2169905	0.48829215	0.34358429	0.2542374	0.13109076	0.01132464	0.15744674
0.55534374	0.03839779	0.02709563	-0.1595456	1.0139211	0.75595409	1.0464706
-0.4221559	0.3122976	0.0470489	0.04359581	0.30344727	0.48824198	0.42610257
-0.2232193	-0.2293259	0.03847421	-0.0714137	0.69719835	0.650096	0.53395152
0.08173756	-0.0525916	0.12971694	0.23391798	-0.2527771	-0.159093	-0.2922102
0.75942984	-0.0480027	-0.0706152	-0.1269022	-0.0352275	0.1453958	-0.0648874
-0.6253874	-0.1257685	-0.1818117	-0.1741409	0.5022965	0.36220536	0.23880578
0.115602	-0.0919688	-0.1073912	-0.1695646	-0.0673169	0.03756299	-0.1066108
-0.0942542	-0.2217095	-0.1258408	-0.1506797	0.93963546	0.80065361	1.0593588
-0.6101358	-0.0310851	-0.1255071	-0.4255099	0.80113311	0.50874359	0.85153645
-0.3942739	-0.4999296	-0.3713737	-0.0730844	0.39039341	0.17255269	0.41979747
0.13428261	-0.0775467	-0.183575	-0.1468093	0.07274209	0.11610035	0.20006319
-0.2243359	0.1267642	0.10805395	-0.3083385	0.03017403	0.34191213	-0.3476545
-0.6601157	-0.2620454	-0.5043118	-1.5714067	1.7598582	1.6056138	1.6164387
0.06205256	0.38752041	0.26428893	-0.0018008	-0.2939944	-0.1748017	-0.4057513
0.042879	-0.26434	-0.22668	-0.26567	-0.12125	-0.029789	-0.054663
-0.21781	-0.081024	0.014203	-0.018054	-0.1551	-0.22086	-0.22204
-0.016357	-0.11316	-0.03712	-0.034537	-0.9187	-0.83558	-0.98268
0.40539	0.18686	0.046492	0.045382	1.9422	1.3025	2.3511
0.59559	0.43355	0.23577	-0.11059	1.8456	1.3367	2.2862
-0.053864	-0.038006	-0.0019865	0.15321	0.087324	0.027784	0.02648
0.10115	-0.61919	-0.9417	-0.76768	0.8391	0.8464	0.80622
-1.3152123	0.07850099	0.04365548	0.19335697	-0.0176769	-0.1406083	-0.0087104
-0.0470563	-0.1784402	-0.2401259	0.06747139	0.08080688	-0.0600309	0.19645379
-1.2603218	-0.3536077	-0.2348394	-0.4396091	0.85416949	0.5453723	1.6770731
-0.395509	0.62018987	0.27859633	0.17920786	1.0503698	0.84723141	1.1351465
0.16098904	0.0225337	0.04279355	0.05168727	-0.1297911	-0.2161958	0.00278143
-0.4596122	-0.6412811	-0.5176302	-0.6689064	-0.4982418	-0.4772984	-0.3268487
-0.5371859	-0.2070183	-0.2845384	-0.3844818	0.18865598	0.28705517	0.19255469

-0.0999681	0.21327591	0.18180637	-0.0395412	-0.5681117	-0.4864382	-0.5018998
-0.1487309	0.20454942	-0.0555954	-0.1131032	-0.4606383	-0.2396951	0.36514328
-0.1080675	-0.0660906	-0.1789804	0.01897633	-0.0350594	-0.0864567	-0.0574715
-0.0090428	-0.1690627	-0.2033272	-0.0590257	0.27568941	0.2488335	0.38492516
-0.4309965	-0.3992277	-0.4347836	-0.4620189	0.80761346	0.98805485	1.0145212
-0.2700908	0.83221159	0.85265919	0.70399451	0.73491883	0.5212494	0.94837447
0.22474184	0.05488118	-0.0097013	0.10625032	0.2190209	0.03509981	0.40953534
-0.3202671	0.10585981	0.07565229	-0.1550711	0.04736249	0.02897963	-0.0060234
-0.1518803	-0.0384646	-0.003199	-0.2138909	0.36908804	0.505862	0.25462115
-1.1568539	-0.2411132	-0.037737	-0.029482	0.60262453	0.32860491	0.86317447
-0.3876263	-1.4404676	-1.5175	-1.707311	0.42619249	0.7978606	1.128654
-0.9191819	-0.5738668	-0.2702492	-0.4271408	0.82753843	0.83997891	0.94309631
-1.5806956	-0.5743109	-0.3476951	-0.1159284	0.30165054	0.07922629	0.38388184
-0.3504157	0.39132681	0.42772638	0.2224549	-0.3416745	-0.3298007	-0.2902206
-0.7435602	0.5751898	0.46179003	0.23268126	-0.6965574	-0.6412363	-0.7642341
-0.0229825	-1.1094226	-1.1747232	-1.1037479	-0.3329084	-0.0174527	-0.1349785
-0.9555895	0.00330118	-0.1178734	-0.1761959	-0.0431333	-0.220778	0.12370242
0.04895791	0.49517001	0.28243513	0.06054715	0.68459527	0.60886636	0.54215779
-0.4369304	-0.3674099	-0.548179	-0.8209411	-0.5288489	-0.3366686	-0.5475006
0.04139333	-0.0100401	-0.0710705	-0.0513664	1.6816004	0.94858927	2.321062
0.16305631	0.00582882	-0.0450585	0.22370824	0.22032669	0.64996935	0.36373088
-0.0703815	0.2209662	0.18967861	0.15996381	0.07637708	0.0318955	0.09915476
-0.52104	0.10182	-0.0050844	-0.19379	0.33086	0.20525	0.41761
-0.4238204	-0.8390509	-0.7603314	-1.1895573	1.6510888	1.3360433	1.6219895
-0.3286685	-0.0947401	0.07391443	0.06743587	-0.172211	0.0851348	0.22173165
1.6336215	-0.3361101	-0.5176317	-0.8763892	0.25348887	0.34706386	0.28521088
-0.2625319	-0.1346986	0.00104593	0.06123416	0.43634596	0.34268133	0.48307158
0.2360622	0.05046125	-0.0299434	0.09649405	0.00224598	0.02552113	0.00089318
-0.6380131	-0.0718029	-0.014743	0.10921529	-0.4226733	-0.324387	-0.4510484
-0.0406287	0.03334887	0.03027924	-0.0376936	-0.0611971	-0.0730317	-0.127004
-0.5605469	0.20505859	0.25525642	0.18144971	-0.2098319	-0.1911296	-0.3157146
-0.2387237	-0.0995926	-0.1726908	-0.0723902	-0.3985554	-0.3215096	-0.5349437
-0.1511418	0.45732693	0.37425727	0.08523993	-0.2142461	-0.3344523	-0.1326969
0.00367335	-0.1638072	-0.0880113	0.10482921	0.02232393	0.12191083	-0.005559
-0.7942548	0.53094529	0.37660562	0.37311798	0.08743951	0.05006125	-0.3556844
0.05453437	0.27965047	0.21800418	0.30379252	0.06951412	0.13272542	0.06556893
-0.6340288	0.34436611	0.12503302	0.00921164	1.0298887	0.92984187	0.83390192
-0.0572304	-0.0003612	-0.1494539	0.02030291	-0.0239866	0.03624404	0.0794595
0.09584411	0.03423886	-0.1036476	0.041035	-0.0325021	-0.0199642	-0.1519385
0.45650553	-0.6479599	-0.7643489	-0.6929839	-0.2970574	-0.5095806	-0.3220518
-0.0501242	0.08836366	0.16290011	0.05785165	0.0165295	0.03806012	-0.0271935
-0.4062032	-0.7603125	-0.736897	-0.700607	0.53526931	0.5882532	-0.105072

0.1571553	-0.113383	-0.1499354	0.18647305	-0.1454899	0.02210185	0.01481772
0.22060167	0.05399998	-0.2174542	-0.2202982	-0.016892	-0.1529388	0.05629492
0.279203	-0.3474271	-0.1641162	-0.0352726	-0.0065818	0.06776162	-0.0422129
0.14661975	0.00873641	0.13106262	0.30527968	0.24821488	0.13432228	0.05491428
0.26608885	0.03612515	-0.0813133	-0.0345581	-0.0409771	0.02346572	0.00668552
-0.0414503	0.03470505	0.09823787	-0.061452	-0.1473774	-0.1303135	-0.0935922
-0.7779837	0.21367927	0.1422312	0.20762557	-0.3217638	-0.4557232	-0.3248049
1.1663128	0.3012527	0.47228772	-0.166557	1.2762091	0.58500976	2.1426863
-0.1294273	-0.2063402	-0.2872792	-0.1429912	-0.0826369	-0.0024569	0.12949407
-0.0521459	-0.0380718	0.08655401	0.22389195	0.15054255	-0.0420455	0.18460858
0.00713947	0.14297201	0.07094201	-0.0288204	0.10207337	0.22298689	0.04104749
-0.7642506	-0.1435146	-0.1486625	-0.2314211	0.21572441	0.30480414	0.06634865
-0.115915	-0.0288208	0.05958474	-0.026666	0.35235172	0.20630958	0.53919735
0.34477859	-0.1123425	-0.0758166	0.05230663	-0.0402923	-0.0597047	0.10566629
0.01996572	0.01843241	-0.0087902	0.06923851	0.16001818	-0.0472366	0.14493535
-0.051065	-0.2076175	-0.3147852	0.06212641	-0.1104779	-0.2159441	-0.2181197
0.41405931	-0.8796883	-1.1020576	-0.5390042	-0.8664781	-0.6314792	-1.0816421
0.23011944	0.71155096	0.59331716	0.10943802	-0.285741	-0.0302123	-0.3435157
-0.735588	0.31404453	0.19612229	0.04226562	-0.0123896	0.21080446	0.09092093
-0.3078032	0.07719288	-0.1411794	0.10907465	0.06963044	-0.0602254	0.18583393
-0.1617166	-0.5742093	-0.6072081	0.23321736	0.16733742	0.24937855	-0.0285222
0.0147801	0.22492913	0.28554751	0.10951891	0.45784719	0.41978267	0.4821969
0.07448842	-0.150996	0.0573211	0.11491169	0.12500285	0.14345217	0.05141961
0.10952527	-0.7282589	-0.6274099	0.16433053	-0.1971771	-0.1467739	-0.2103529
0.40032986	-0.0399823	-0.1856132	-0.0336951	-0.1806698	-0.2442199	-0.1833254
0.00310184	0.06148544	0.07409658	-0.2003329	0.08451673	0.21429119	0.08979088
0.57473212	0.14424342	0.1164187	-0.0346222	1.2136094	0.76641827	1.3472181
-1.4271671	-0.1955557	-0.1544463	-0.1078175	0.27845368	0.24536076	0.36755121
-0.387034	0.42373186	0.1142902	0.10116995	-0.1758043	-0.2653989	-0.0649638
-0.8397224	0.19266937	-0.0745467	-0.0005629	0.40043216	0.03664567	0.24533616
0.01625849	0.14959455	0.00866788	-0.0315987	-0.3046965	-0.1537822	-0.3370294
-0.3251579	0.18971699	0.2365529	-0.0950391	0.15508398	0.08342268	-0.0224562
-0.0472578	-0.4750434	-0.6474983	0.04380262	-0.3462016	-0.1983741	-0.2019528
0.05393446	0.13328384	-0.0348186	-0.0945178	-0.1862302	-0.2094483	-0.1679337
-0.3840651	-0.5255216	-0.4233141	-0.2570582	-0.1622246	-0.295199	-0.1180987
-0.8072384	-0.1446191	-0.0524129	-0.0688603	0.13854061	-0.1709873	0.23679246
-0.4712104	0.059059	0.09237078	0.06284708	0.19175417	0.32443375	0.43979532
0.04977194	-0.1508367	-0.2654092	-0.0873506	0.46926493	0.40658707	0.66755434
0.64513178	0.89554192	0.89290005	0.74324446	0.81331706	0.65628902	0.74256936
-0.09069	0.09573741	0.11809979	-0.0311238	-0.2898525	-0.4809452	-0.3076644
-0.5446787	0.56098361	0.55470629	0.5214817	0.11226094	-0.0052062	0.26614003
-0.7060454	0.43042239	0.22204324	-0.195487	0.16690137	-0.1204605	0.24761294

0.02943471	-0.1013388	0.02801373	0.02575522	0.51185987	0.38545954	0.77537307
-0.2018276	-0.5671649	-0.4052215	-0.07318	0.05257729	0.00305244	0.19259731
0.26553997	-0.2731134	0.04479222	-0.2668211	0.23515622	0.08252437	1.0041959
-0.3914368	-0.3747866	-0.1908621	-0.1166303	0.02449089	0.0195543	-0.0941751
-0.0387548	0.38785781	0.35666188	0.47028998	0.14469467	-0.0919847	0.12778273
-1.0532103	-0.0276482	0.25068798	0.25027962	0.9051191	0.54514226	1.0735701
-0.594588	0.26597248	0.24019011	0.38231685	-0.0687437	0.07832058	-0.0459596
-0.3497525	-0.6716982	-0.4966715	0.19445861	-0.0057666	0.16844104	-0.0815213
-0.1486125	0.00417105	0.05914571	0.08295747	-0.1133526	-0.0174667	0.11611726
-0.8752063	0.10883319	0.25992558	0.17041194	0.88608665	0.52861897	0.68433083
0.29106853	0.10098321	-0.0071559	-0.0126502	0.93265418	0.70969957	1.2137621
-1.2472931	-0.416696	-0.4042754	-0.1323112	0.0859028	-0.2739431	-0.013284
-0.2501241	0.01147893	0.10000525	-0.067074	-0.9233483	-0.7707516	-1.2931876
-0.4196196	0.25474138	0.16836957	0.15300267	0.48529722	0.20977866	0.66595167
-1.491201	0.66497366	0.50944508	0.47997306	-2.2685527	-2.0417457	-2.5112486
-0.0431195	0.11050417	0.06432255	0.06005064	0.11807094	0.0978497	0.04185043
-0.6039086	1.1019107	0.92597197	0.80317155	0.67134297	0.49525771	1.0549435
-0.2393021	0.10792388	0.246816	0.91926411	1.065184	0.81536361	1.1060981
-1.0744558	0.39712857	0.42816017	0.42303537	0.20409593	-0.0964741	0.25088336
-0.4517789	-0.1603188	-0.158296	-0.1242746	0.03782773	0.20196654	0.35713569
-0.5217115	0.25484682	0.1515217	0.19163927	-0.1619955	-0.4864653	0.06933794
0.25839744	0.04060862	0.10529715	0.13801228	-0.0677509	-0.1388216	-0.2765244
0.40880185	0.4728333	0.66116998	0.68927329	-0.2551438	-0.1986618	-0.6208082
0.11683285	0.08777627	0.22715174	0.28714299	-0.2296878	-0.0067994	-0.1118224
-0.2656882	0.1315289	-0.0010196	0.13046577	-0.2624634	-0.4003522	-0.2651387
0.24205278	-0.1129704	-0.0420828	0.18302064	-0.2816096	-0.2817572	-0.4301783
-0.0688182	-0.2235603	-0.2539485	-0.0820326	0.51997952	0.43708919	0.556752
0.20423238	-2.5544154	-2.592162	-2.6120679	2.7511041	2.3426802	2.9750461
0.18904499	-0.2496131	-0.1225082	-0.0325774	2.3441208	2.0948209	2.4339694
0.13767564	-1.0377298	-0.9387251	-0.5099571	1.9971927	1.5594902	1.7753688
0.01465549	0.08439217	0.05394649	-0.0956739	0.08608617	0.00330026	0.26425508
-0.6846826	0.01204198	0.1641602	0.37179961	-0.3393534	-0.5002901	-0.1345209
0.33720141	0.67780127	0.39153232	0.17928434	0.2147295	-0.0017658	0.35892039
-2.359196	-0.9625091	-0.982683	-0.5673381	-1.9439735	-1.6346876	-1.6432697
-0.3592495	-0.0888902	-0.0666412	0.12657515	-0.0518274	-0.0501798	0.18825294
0.12105047	0.33120983	0.11894882	0.050122	1.1693683	0.73883541	1.2056405
0.52993892	-0.2390013	-0.110052	-0.2321352	0.19252267	0.19659129	-0.0132572
-0.3793587	0.52770184	0.4667411	0.89679568	-0.5743429	-0.7384461	-0.4440916
-0.2632945	0.73032932	0.68742511	0.57188262	0.19352109	0.2573879	0.32838825
1.0437869	0.76615707	0.89469907	0.64086578	-1.8100799	-1.2479929	-1.8385847
0.21575936	-0.0033936	0.19770148	0.59907397	0.68685446	0.61357484	0.6410091
-0.1005657	0.02480656	0.10678941	0.0286111	0.04184317	0.07633665	-0.0060974

0.05235412	0.11470184	0.10435707	0.26016742	-0.086687	-0.0902574	-0.0319841
-0.22399	-0.12856	-0.027088	0.19024	0.21947	0.10044	0.32597
1.671597	-0.5139477	-0.3773075	-0.231362	-1.1033042	-0.7063104	-1.3687197
-0.4699562	0.02961337	-0.0675715	0.0934086	-0.5284722	-0.3638814	-0.3529556
0.12698412	-0.149353	-0.3441996	-0.3553217	1.3007069	0.46315419	1.3226408
-0.10443	0.30838177	0.29515229	0.17739763	-0.2699237	-0.4313712	-0.181081
0.60091742	-1.7530091	-1.8644393	-0.7222114	1.9068106	1.7146842	1.7204453
-0.52366	-0.26652	-0.35312	-0.11906	0.56827	0.45841	0.92176
-0.1761626	0.26575759	0.46831839	0.64746274	-0.0427352	-0.0345185	-0.2244934
0.10705781	0.09124241	0.03090699	0.35730557	0.06715222	0.1232287	0.13622378
0.35860057	-0.137319	0.03191615	0.4006543	-0.0810716	0.62134531	-0.5534429
-0.7964917	-0.7443265	-0.6757376	0.1433237	-0.3430992	-0.3137955	-0.3073269
-0.5839931	0.02700501	0.15217445	0.1500317	-0.5890309	-0.4991221	-0.5810247
0.218855	-0.0776009	0.11839236	0.26555767	-0.1018486	-0.0751814	-0.2279393
-0.0585119	-0.0900896	-0.0646982	0.26138047	-0.1591913	-0.1953803	-0.0474921
-0.1758772	-0.3083504	-0.2149571	-0.0059276	-0.1250026	-0.0884335	0.05133997
-1.1792294	-0.4572172	-0.3620096	-0.4670417	0.37316439	0.16594761	0.83752326
-1.3600639	-0.6853881	-0.5729241	-0.3223242	-0.2128657	-0.4655134	-0.4094511
0.23833041	-0.1856063	-0.1674958	-0.1967676	-0.0036148	0.16217755	-0.6413608
-0.5287678	0.17177172	0.15901858	0.19680628	0.56851681	0.3568615	0.58302775
0.94447162	0.29271431	0.2427914	0.26465533	-0.0845459	-0.2734393	-0.3832328
-0.6604013	-0.1224932	-0.326065	-0.1347432	0.49011251	0.17611362	0.6244476
-0.6038993	0.13203716	-0.0694405	-0.0690902	-0.4114773	-0.4670087	-0.741203
-0.8684195	-0.9215024	-1.1437691	-0.7962849	-0.0676693	0.04730309	0.08478632
0.10050165	-0.0797593	0.02637912	0.32513897	0.00950677	-0.0675277	-0.0968133
-0.5453666	0.00339601	0.022225	-0.1747831	0.36737563	0.34221597	0.46733806
0.9903611	-3.0233598	-2.9569436	-2.6265331	-0.5989236	-0.6701168	-0.9004846
-0.9506337	-0.4706243	-0.5113066	0.01675703	0.34138366	0.09731056	0.198653
0.71168007	-0.2904786	-0.309538	-0.3565641	0.63554726	0.3868111	0.45438009
0.00878195	-0.2708275	-0.6388823	-0.0884564	0.50774963	0.47335495	0.57917308
-0.5025355	0.02941196	0.0741972	-0.0069983	-0.0697262	-0.1687923	0.19059885
-0.1093518	0.27694359	0.16892145	0.08222936	0.06797116	-0.001444	-0.0852632
-0.384336	-0.1281851	-0.1031839	0.01191568	-0.2207605	-0.39469	-0.0759175
-0.0184951	-1.3838452	-1.3030053	-0.6082336	1.0458286	0.8845109	0.4024672
-1.4984329	-0.5471467	-0.4201857	0.04179012	-0.051352	-0.0974916	-0.2407175
-0.0591079	-0.1834096	-0.2097101	-0.214057	0.06404405	0.01154129	-0.0686162
-0.2135266	-0.4821139	-0.4336159	-0.2430661	-0.1366233	-0.1246995	-0.2435645
1.1146085	-0.2577545	-0.3023314	-0.3119024	0.75056131	0.53241259	1.1071302
-0.601377	0.22006183	-0.0412957	0.3153993	-0.0417084	-0.5667716	-0.1162481
0.07171576	0.01265208	-0.0591965	0.04089703	0.60485006	0.2587667	0.72784073
-0.7136682	0.05768552	-0.0721562	0.05841626	-0.2113209	-0.3067	-0.4118813
0.00494763	-0.1327527	-0.1692822	0.19308256	-0.1217888	-0.24283	-0.1633717

-0.3755143	-0.2188564	-0.2233921	0.00230055	1.0380742	0.5575861	1.599399
0.33161508	0.04787911	0.12784394	0.44092117	0.12458666	-0.0984213	0.23485651
-0.1014688	-0.1527101	-0.0780769	-0.0166634	-0.3706414	-0.3917205	-0.2831573
-0.2619732	0.34401563	0.33540628	0.28060097	-0.8476056	-0.9951927	-0.6654762
-0.0799748	-0.0403715	0.06523622	-0.0177215	0.16630083	0.06101315	0.43471758
-0.4913287	0.07485561	-0.0310667	-0.0189884	0.09835749	0.12182534	0.34851332
0.02592741	-0.0383616	-0.1859668	0.19104264	0.13484323	0.04048337	0.01303746
-0.0356518	-0.0568698	-0.0919559	-0.1855606	0.08267826	-0.0256518	0.01290223
-0.017851	0.17182617	0.10163066	-0.0292098	-0.0206916	-0.0988059	-0.0523952
-0.5605991	0.05145906	0.036473	-0.18632	0.19714174	0.15063598	0.30527938
-0.0194202	0.05692923	0.02060257	0.16228637	0.17808127	-0.0067488	0.2212878
-1.5561272	-0.7901376	-0.7600552	-0.0076968	0.02269583	-0.1246031	0.17140937
-0.0960707	0.12966398	0.11711766	0.15478017	-0.0353881	-0.0707358	0.0763276
-0.1497279	0.40639222	0.19478358	-0.0512809	-0.2846108	0.0449839	0.04135424
-0.0839409	0.00774993	0.13379353	0.08731837	-0.1855031	-0.6091418	-0.1473119
-0.1739709	0.03411843	0.1680946	0.01816513	-0.2316382	-0.2441003	-0.4099819
0.43352994	0.17166814	0.1959612	-0.1346654	-0.3886328	-0.341753	-0.6156304
-0.7006143	-0.9033876	-0.6897269	-0.4057898	0.42238421	0.15968005	0.21434684
-0.3796703	0.18650485	0.04455467	0.04536968	-0.0017667	-0.2656641	-0.0847942
0.24642552	-0.1862868	-0.2064149	-0.6343203	0.41618869	0.5817046	0.6148031

test_GSE683	test_GSE683	test_GSE692	test_GSE699	test_GSE732	test_GSE732	test_GSE732
ref_GSE6836	ref_GSE6836	test_GSE692	ref_GSE6992	ref_GSE7326	ref_GSE7326	ref_GSE7326
GSE6836	GSE6836	GSE6923	GSE6992	GSE7326	GSE7326	GSE7326
GEO	GEO	GEO	GEO	GEO	GEO	GEO
affymetrix	affymetrix	affymetrix	affymetrix	affymetrix	affymetrix	affymetrix
GLUCOSE:12,	GLUCOSE:12,	ATCC25404:1	PARAQUAT:0	PaeR7I M:+1		PaeR7I M:+1,
1619	1620	1622	1638	1657	1658	1659
0.1244828	0.30094231	0.08873946	0.14114367	-0.1726521	-0.0713483	0.21954719
0.39462305	0.38911564	-0.1754923	-0.1439119	0.01411873	0.02516767	0.03311979
0.67332084	0.50569647	-0.1035543	0.44175531	0.23028306	0.23817766	-0.2383361
1.134376	1.0457679	-0.0386726	0.16696989	-0.0501082	0.01484147	0.23762708
0.15733894	0.15769341	-0.2527235	-0.0072151	0.15868785	0.0578995	0.07300368
0.56034817	0.60424944	-0.03772	-0.0845278	0.10955098	-0.0320726	-0.0580203
-0.0713128	0.08104003	0.10919944	0.01433711	0.24984563	0.61001929	-0.1006012
1.3634321	1.1171783	0.73574798	-0.1713889	-0.3049197	-0.4878588	0.06621996
0.00735901	0.02082192	-0.1605803	-0.0797124	0.03430986	-0.0473869	0.06217128
0.72449165	0.59943677	0.39335648	-0.4147751	-0.0221032	-0.0775284	-0.1174881
0.26197526	0.19884768	0.60858319	-0.0344088	-0.5498425	-0.2319676	-0.1539469
1.3435296	1.0295884	-0.3436693	-0.8903126	-0.0385656	-0.4857397	0.04770662
0.55715023	0.53354279	-0.0429736	-0.0540008	-0.2584512	-0.1618144	0.17383566
0.09358313	0.14859026	-0.0836023	-0.4406999	0.20515619	0.08482698	0.0231145
0.02830733	0.12037916	0.35538181	-0.2053987	-0.0203945	-0.0334757	0.04000463
0.08762849	0.36473429	-0.2606559	0.12532705	0.040395	-0.0177658	0.17534087
-0.3183674	-0.0562153	0.08769268	-0.20362	0.20483234	-0.2168642	-0.0616911
-0.5690435	-0.3844761	0.16332646	0.11805543	-0.1227401	-0.0998324	-0.090927
-0.2083711	-0.1966192	-0.0891826	0.00945883	0.03075289	0.06499177	-0.1130312
0.15989651	0.11792328	-0.0162936	0.0564319	-0.0859915	-0.0933831	0.20848486
-0.0060071	-0.1773632	-0.1352109	0.17128368	-0.1322338	0.07427838	0.25207248
0.74186148	0.91370965	-0.0232028	0.13585	0.00282537	0.03025359	0.00671832
0.5635213	0.59912572	-0.0874185	0.35373677	0.26750441	-0.3109032	0.19229565
0.23273005	0.05372437	0.00419688	-0.10929	-0.1339955	-0.1549905	-0.0289253
-0.0078601	0.08205991	0.1802153	0.217069	-0.1106351	-0.0235599	0.1949246
0.58896921	0.51457191	0.128542	0.00842026	-0.0122576	0.01652688	0.33502335
0.16426078	0.18800245	0.77872691	-0.74337	-0.2515976	-0.121664	-0.0816709
0.07882577	0.11252575	-0.153127	-1.1014777	0.15342805	0.19456624	-0.1785328
-0.2415778	-0.0524229	0.03316643	0.42668	-0.3173515	-0.0329548	-0.0226478
0.57237836	0.83960086	1.5067811	-0.4991128	-0.1813828	-0.1247492	-0.0237112
-0.9600933	-0.6211738	0.26866227	-0.4886264	-0.3501631	-0.2174906	-0.1961534
1.0914595	0.91813623	0.55635431	-0.6824526	-0.109334	-0.2001419	-0.0585071

0.79164014	1.3653648	-0.5398222	-0.3563328	0.18194125	0.169422	0.03974454
0.08134899	0.11802496	-0.115809	-0.0691286	-0.1312688	-0.0558554	-0.1078327
0.57740427	0.63144984	0.49895417	0.0381098	0.12803045	0.21580118	-0.1697079
0.04380956	0.12140761	0.31404791	0.03573957	-0.0727947	0.00786346	-0.1060437
0.06774214	0.03334013	-0.0254698	-0.1284344	-0.0101806	-0.0152813	0.01542618
0.39456432	0.45521518	0.11105835	-0.0770002	-0.0995839	-0.1245085	-0.0712607
0.15594107	0.13021253	-0.0337767	-0.2086142	-0.0458973	-0.1370082	-0.1223502
0.21393448	0.18704894	-0.0530361	-0.2117561	0.07923843	0.09108554	0.20421923
-0.1590242	-0.1015512	0.02165236	0.12563354	-0.0605006	-0.0732715	0.22719934
0.08964064	-0.022159	0.17204339	0.03378562	0.01449607	-0.0453782	-0.0911386
0.48566314	0.31423644	0.29970095	0.02142146	-0.1033471	0.07463781	-0.0696849
-2.4715448	-2.5317152	0.99754929	-1.2943799	0.08826197	0.27593916	-0.3807799
-0.1641448	-0.1914915	0.13763477	-0.1681589	-0.16547	0.01369258	-0.0948049
0.02913555	-0.3002066	0.20381385	0.00631944	0.01844398	0.15453884	0.01451838
0.26830937	0.1677223	0.56043662	0.14482392	-0.2452501	-0.0417732	0.20581984
-1.2830814	-1.1898166	0.25377883	-0.3406415	-0.0004488	0.0813915	0.24074805
-0.2656918	-0.0780958	-0.2332508	-0.1000997	0.01082896	-0.0907929	0.10839771
-0.2355253	-0.040981	0.05856128	-0.4660827	-0.1279738	-0.0249771	0.03434185
0.27888991	0.09821162	0.08703144	0.00588434	0.12093787	0.12725542	-0.0671643
0.2247049	0.2060471	0.11481081	-0.0138085	-0.0023624	0.00453908	0.12961653
0.6093225	0.49635777	-0.0948356	-0.0007539	0.24105852	0.06206868	-0.0837976
0.04021582	0.12645106	0.33532835	-0.0292593	0.0939693	0.07042495	0.16069689
0.96575211	0.87555956	0.38653278	-0.184977	-0.0145538	0.04934974	0.19067349
0.13664374	0.02740224	0.15759892	0.11981042	-0.1144253	-0.1318831	0.24088165
-0.0737045	-0.0097359	0.0015171	-0.2206281	-0.1052379	-0.1962237	-0.1399416
2.3258008	2.4326765	0.01949029	-0.2550841	0.19352653	-0.10991	0.00270467
0.92408979	0.54477951	-0.0143149	-0.3297474	0.09090681	-0.1110373	0.03584774
0.40538254	0.31013095	0.60277794	-0.1385301	0.06472361	0.00578687	0.25863606
0.59557621	0.67326338	0.02401783	0.07487762	0.26319378	0.28358839	0.17606213
-0.5225452	-0.1368847	-0.330673	-0.3764627	-0.0546343	-0.0385599	-0.064797
-0.8086504	-0.5627313	-0.9295579	-0.6481649	0.02849653	0.19915743	-0.1364014
0.48941344	0.46780158	-0.0071039	-0.1987842	-0.1245409	-0.0884054	0.11292006
0.14300651	0.02045751	0.0191992	-0.2068659	0.09315383	-0.0176594	-0.2422817
0.13535549	0.1729249	0.04280349	0.19109426	0.14425173	-0.4243317	0.02255431
-0.0476191	-0.0364784	0.37457705	-0.1054624	0.0542802	-0.0062776	-0.0265425
0.90395279	1.4165723	0.16776585	0.1087385	-0.1659696	-0.0562718	0.05357592
2.4972462	2.6064212	0.02386088	-0.0996362	0.02713068	-0.079787	0.20884538
-0.0182804	0.07140241	-1.7808791	0.10610516	0.0716362	-0.020775	0.0057974
1.7319268	1.936392	0.51795891	-0.338841	-0.0460838	0.05385402	-0.2481605
-0.589357	-0.613019	0.03564437	-0.2382447	0.07541913	0.29284783	-0.1117649
0.23549311	0.18313483	0.37860224	0.02648982	0.046305	-0.1040691	-0.0811574
0.32140413	0.24521986	0.36144301	-0.2475058	-0.1713578	-0.0849078	-0.1140826

1.2168318	1.0326004	-0.3359101	-1.1843881	0.06843094	-0.1045987	-0.0896078
0.48800952	0.19989566	0.45148651	-0.1923241	-0.0377154	0.04053124	-0.1326629
-0.0789806	-0.0111256	0.23307624	0.17895101	0.13192191	0.13547024	0.05652717
0.03628824	-0.0932259	-0.0529902	0.26595574	-0.0120642	-0.0270378	-0.2209685
0.48557385	0.36802071	0.05697957	-0.0031658	-0.0221923	-0.0452399	-0.0030258
0.57456862	0.32905836	0.57596502	0.22252454	0.04292327	0.14843445	0.07625009
-0.0006039	-0.0183605	-0.0418561	0.02715231	-0.255671	-0.1018496	-0.0272921
-0.3973386	-0.316006	-0.040247	0.10294468	0.01561127	0.11707483	0.13140814
-0.1252202	-0.0262491	0.10060713	0.04310543	-0.1453275	-0.0717774	-0.0670686
-0.5792417	-0.799168	0.28676135	-0.1595422	0.20158817	0.50964931	0.01354579
-0.1675785	-0.1683944	0.05620424	-0.0918225	0.02324484	0.07753823	0.20331577
0.22978192	0.2822778	0.34116295	-0.1087407	-0.0972261	-0.0439788	0.21804567
0.20086988	0.1768879	0.81802877	0.04197604	-0.0928726	-0.0718405	0.09288309
0.10017502	-0.058958	0.18757876	0.14559066	-0.2038703	-0.0808831	-0.1404095
1.1292719	0.95125501	0.10303675	0.35699	0.07415811	0.08029419	0.02973678
0.47635126	0.357643	0.19836907	-0.0434419	-0.1780088	0.02300555	-0.0926183
0.65012228	0.70742778	0.65160785	0.00761508	-0.217706	-0.0455323	-0.1234757
-0.2737419	-0.248774	0.32996775	0.14093188	-0.1218404	0.00822777	-0.1377692
0.14510407	0.09253343	0.57696405	0.0080727	-0.1016207	-0.1434576	0.12118194
0.27250266	0.44067951	0.48732242	0.18438428	-0.0494588	-0.1004942	0.00997014
-0.0111708	0.0052084	0.87866083	-0.027839	-0.0688664	-0.089704	0.00299161
0.95387849	0.95380769	0.42940938	0.17497	-0.1316062	0.02292953	0.03796481
0.64214874	0.76350641	0.2152421	-0.2540678	-0.1676178	-0.2137051	0.23563084
0.34519333	0.09699353	-0.064747	0.02034275	-0.1053612	-0.2436267	0.07346529
0.09248726	0.16454298	0.22701586	-0.1019316	0.14170758	0.06458115	0.33142909
-0.0904388	-0.0322786	0.51588752	-0.3785742	-0.3249445	-0.2666632	-0.1178084
1.8510417	2.2972113	0.48511346	-0.3091805	-0.4046243	-0.0608349	-0.1154968
-0.503009	-0.2887846	-0.0858063	-0.2282172	-0.4012274	-0.085896	-0.397194
0.027177	-0.095593	-0.12102	0.031422	-0.010769	0.05419	-0.0060877
-0.25662	-0.19306	-0.94263	-0.51841	-0.2	0.03213	-0.20154
-1.0159	-0.92785	0.14766	-0.34544	-0.32821	0.022076	-0.44878
2.9668	2.733	0.93545	-0.42839	-0.42852	-0.23671	-0.29445
3.0008	2.9102	1.0728	-0.55634	-0.2851	-0.15931	-0.12624
0.38202	0.1002	0.49681	-0.17856	-0.10601	-0.039284	-0.12271
1.0888	1.1247	0.55524	-1.1651	-0.24902	-0.084813	-0.28569
-0.012219	-0.1135539	0.04699679	-0.2103593	-0.0571946	-0.1193467	-0.2101854
0.04488676	0.01842707	0.04319202	0.15719083	0.02272472	-0.0116459	-0.1400012
2.1425	2.0253021	0.43118618	-0.1935348	-0.2118617	-0.0561352	-0.2054843
1.1955814	1.4034847	0.28211634	-0.3169377	-0.1140361	-0.0669271	-0.0847651
0.04208607	0.13255859	0.03661949	0.04009826	-0.1284688	-0.0320779	-0.1224399
-0.3598031	-0.5568251	0.02029557	0.00096398	-0.4799208	-0.353599	0.0765267
0.71404456	0.78889921	0.64291135	-1.0543522	-0.0850705	-0.0523797	0.07982489

-0.6240339	-0.433095	-0.0170979	0.00099581	-0.0955694	-0.2389705	-0.1378938
0.48501617	0.8159541	-0.0392613	-0.0236618	0.00098299	0.29212378	0.00185303
-0.2071096	-0.0616508	-0.0904723	0.07638441	0.05520836	0.03241038	0.01424413
0.48506809	0.44898053	0.30610165	-0.2262983	-0.0603132	-0.0718692	-0.1262768
0.46735409	0.39963292	0.13693368	-0.1178908	0.06048109	-0.0447218	0.00672503
1.1394392	1.2963839	0.2497434	-0.5020003	-0.0233886	0.15375543	-0.0811066
0.43342579	0.37290862	0.36473545	-0.009998	-0.1560486	-0.117606	-0.0138969
0.02970047	-0.0465537	0.28547255	-0.326749	-0.1927474	0.01341563	-0.3206116
0.65629708	0.37803726	0.20266717	-0.219205	-0.0487326	-0.0124057	-0.0641784
0.59458549	0.47157135	0.15674414	-0.0603216	-0.1940763	-0.1558874	-0.0274473
0.38954002	-0.0674895	-0.7662851	-0.2922636	-0.260298	-0.2519432	0.11972555
1.0946872	1.2195484	0.61248628	-0.4319588	0.03087551	0.10423778	-0.0828687
0.45390966	0.41986147	0.32504333	-0.1529408	-0.05934	-0.2030847	-0.0743854
-0.3482246	-0.2382665	-0.0076413	-0.0450527	0.02970521	0.21759891	-0.0020808
-0.8325851	-0.786298	0.18689164	-0.2300953	-0.2165765	-0.2422704	0.16258197
-0.2699525	-0.3837846	0.00943393	-0.2540273	-0.0273456	-0.1119324	-0.0917086
0.12918795	-0.2014884	-0.0283813	-0.1028607	-0.3861614	-0.3890414	-0.2442111
0.58033823	0.33817662	0.16887613	-0.1789654	-0.2723453	0.00697334	-0.1296923
-0.7436532	-0.7938072	0.31791068	-0.3987924	-0.3428591	-0.3453276	-0.2198923
1.7302033	1.4754305	0.17163131	-0.1964482	-0.0830085	0.02598935	-0.022468
0.31906753	0.36072061	0.09194301	-0.1545058	0.03630533	0.02582725	-0.0001208
0.10061347	0.09395048	0.4427783	0.01451079	-0.0530397	-0.1099395	-0.1180246
0.3387	0.36756	0.35526	0.098843	0.1667	0.29986	0.0098862
2.0921171	2.205762	1.4065927	-0.7532676	-0.3012418	-0.3739523	-0.0571865
-0.1842354	-0.1118078	0.05748802	0.19982	-0.1205475	-0.1226998	-0.2114299
0.20241883	0.32779354	0.09392606	-0.3239661	0.05913413	-0.0468859	0.04024604
0.40928123	0.49832064	-0.1664903	-0.1035381	-0.0975889	-0.0013799	-0.2210271
0.11120444	0.44399793	0.23288526	-0.5629597	-0.0416844	-0.1060224	0.13494599
-0.4963557	-0.418529	-0.1189356	-0.2139311	-0.0765484	0.050009	-0.0378519
-0.2075148	-0.05443	0.03849543	-0.05238	0.05351996	0.02804403	-0.0727224
-0.3659648	-0.2382011	0.03418244	0.30230452	0.0808324	-0.0028122	-0.2426627
-0.5215094	-0.4245776	0.01550853	0.12108733	0.093362	-0.2088088	-0.0556981
-0.2341112	-0.2759051	-0.2321871	0.60958575	-0.0072724	0.07538733	-0.1034169
0.00243883	0.05180479	0.35718299	0.13173526	-0.0434486	-0.0058642	-0.0414687
-0.2857016	-0.3031522	-0.0241877	-0.3009909	-0.1143044	0.01912938	-0.1208246
0.0695993	0.11057032	-0.153668	0.1568036	0.05307591	0.07445949	0.01187472
0.82719426	0.84149564	0.38483804	-0.3109493	0.03542895	0.00579277	0.05253739
-0.0819422	0.04980691	0.13902475	0.03221791	0.0622783	0.04059583	0.11046696
-0.0864586	-0.0579005	0.10387542	0.05697229	0.13678603	0.01075276	0.11537414
-0.4702017	-0.3603145	-0.0835899	-0.0217013	0.03872412	-0.1053098	0.13467914
-0.0101394	0.04747148	0.34156154	0.02330124	0.03527152	0.01082349	-0.1177824
-0.4837585	-0.8532202	0.0961725	-0.3160065	-0.3422749	-0.2470754	-0.2375309

-0.2317935	-0.0858658	0.05002919	0.08175169	-0.0245685	-0.174339	0.03985767
-0.0639753	0.01733141	0.05008246	-0.4514508	0.03867612	-0.055658	-0.248902
-0.0087052	0.07697488	0.03828729	-0.0456809	0.02647004	0.02476287	0.14047505
-0.0509161	-0.0997177	0.08210196	-0.3046299	-0.084475	-0.0997079	0.04820629
0.14366214	0.24999928	-0.0740465	0.21680808	0.20533783	0.10148848	0.1101422
-0.1842959	-0.1023373	-0.0440371	-0.0124025	0.04091363	0.16227746	0.12922682
-0.4309466	-0.341129	-0.3472532	-0.1637264	-0.0581547	0.06074419	-0.1196669
1.743547	1.4997773	0.7928186	0.82435621	-0.1141776	-0.1933031	0.11285532
0.01342886	0.11739733	0.03232761	-0.0478155	0.13716238	-0.0463916	0.14851225
0.08926703	0.22489376	0.0011185	0.04129832	-0.0838869	-0.0194347	-0.0848311
-0.1812145	0.05295689	-0.2676781	-0.004035	0.19618656	0.06264198	0.09542684
0.25510985	0.4746188	0.13906186	-0.329096	-0.0568624	-0.0532244	0.03348196
1.0070791	1.0837433	0.14888241	-0.1012457	-0.1257693	-0.2271177	0.00103252
-0.01399	0.15865269	0.0757421	0.09048252	-0.0940885	-0.1734094	0.027751
0.20226262	0.31595198	-0.0793024	-0.1128677	0.1357176	0.11816583	0.02078445
-0.2678573	-0.3336271	-0.0437383	0.05965025	-0.0096982	-0.0013667	-0.0037019
-1.0895445	-1.2896584	0.0747901	-1.4810175	-0.2065926	-0.0836825	-0.0049513
-0.0957316	-0.0711666	-0.1253767	-0.2451408	-0.3645152	-0.2601041	-0.1138915
0.01681298	-0.2279507	0.0583096	0.0249259	0.09137607	0.08741896	0.05666669
0.16066405	0.20262457	-0.3134644	0.0922644	-0.2395534	-0.629539	-0.2929638
0.15358308	0.19267903	-0.1772864	0.1509142	-0.1052596	-0.3897069	-0.0532895
0.48983654	0.39249091	-0.2226673	0.24615545	0.07527596	0.15482105	-0.0032979
0.1369612	0.11467647	-0.0292422	-0.056882	-0.105919	-0.0835273	0.00439578
-0.1410529	-0.2452687	-0.1795902	-0.0920214	0.01576842	0.02193875	-0.0533521
-0.2484114	-0.1229233	-0.028045	-0.0582653	0.06876551	0.10183457	0.04540668
0.21721198	0.20479285	0.17208909	-0.2428222	0.10207227	-0.0083666	0.02048042
1.4469503	1.4736593	0.29936377	-0.0091754	0.01156143	-0.1012227	0.02907256
0.48097416	0.2416482	-0.0483451	-0.2024173	-0.1442539	-0.2642726	-0.1582984
0.06027692	0.08472922	-0.0693405	-0.0627938	-0.1574412	-0.0571972	-0.0279935
0.0198948	-0.1722834	0.05947157	0.0284193	0.05470491	-0.012991	-0.1051842
-0.4080656	-0.4079243	0.32384776	0.27094661	0.02350678	0.1029036	-0.1149613
0.25115608	-0.0337037	0.21176847	-0.0084725	0.00381475	0.00093113	-0.1012562
-0.2889632	-0.1198658	0.26182082	-0.1513219	-0.1315892	-0.0824271	-0.1497526
-0.0582723	-0.2088529	0.19704084	-0.1259721	0.02246918	-0.0776621	-0.0958538
-0.0711146	-0.0785751	0.26262222	-0.1025321	0.04262814	-0.1184692	0.07052885
0.22736634	-0.03538	-0.3372069	-0.1720566	-0.0761687	-0.0235759	-0.2782542
0.40694709	0.26474196	0.096294	0.076447	0.05269368	0.05622778	0.18936302
0.8171955	0.66780816	0.02547516	0.09212054	-0.3147257	-0.1339122	-0.2520314
0.61897927	0.68492183	-0.2181038	0.48006987	-0.0846766	-0.1465712	-0.2865715
-0.2219471	-0.2264867	-0.0446001	0.0093016	0.0464483	-0.0432218	0.11545009
0.56574737	0.59583509	0.96666862	-0.2285494	-0.1535097	-0.0492731	-0.0961462
0.42377863	0.648138	1.7688894	-0.3118016	-0.2938437	-0.1930992	-0.1271026

0.68066615	0.59311857	-0.2591774	-0.2146105	0.08908388	0.07565843	-0.0665172
0.11579813	-0.0165395	-0.140974	0.06958409	0.06059654	0.06187146	0.02612851
1.1421852	2.0112373	0.01369385	-0.2678333	-0.2266015	-0.215031	0.16055575
0.01154367	-0.1811389	-0.2203303	0.00622319	-0.0503307	0.00523232	0.01541251
0.01046963	-0.1472561	-0.0405151	-0.2494582	-0.0982973	0.05207971	0.0138562
1.1523602	1.2657214	0.16167258	-0.4693981	-0.0837061	0.04731959	0.01368782
-0.0469895	-0.2662068	-0.1598094	-0.0507404	0.10286466	-0.0236873	-0.1261704
0.04061103	-0.0461798	0.10107834	0.08800213	0.10753624	0.04200141	0.0144926
-0.0660364	-0.0753571	0.04548962	0.12067307	0.0596273	-0.0235468	-0.1505548
0.80780592	0.66769061	-0.0471568	-0.46351	-0.0135877	-0.0315766	-0.0398923
0.88591902	0.89835343	0.08746819	-0.1746113	0.04011594	0.27982295	-0.4084701
-0.3529472	-0.3079675	-0.429267	-0.0881717	-0.0225899	-0.1097375	0.00295285
-1.3235494	-1.3134512	0.37986676	-0.7384589	-0.0895037	-0.0478725	-0.3581421
0.56460319	0.41718023	-0.163567	-0.0993547	-0.008486	0.04075376	-0.0629312
-2.4048723	-2.301306	0.27273955	-0.085715	-0.2739104	0.00323779	0.1618199
0.04889323	0.04208314	-0.0022452	-0.1940883	-0.1225197	-0.0227679	0.01573051
0.66590178	0.73500811	0.27246824	0.12523344	-0.2113582	0.01646453	0.00303604
1.1372568	1.0398359	0.0411358	-0.3582895	0.08293051	-0.0675856	-0.0350059
0.28505361	0.16894026	-0.2176533	-0.289944	-0.2358475	-0.086977	-0.1497433
0.23812587	0.20216768	-0.2783872	-0.021529	0.189855	-0.0019751	-0.1640894
-0.0196478	-0.0787795	-0.4790521	0.09857854	0.12657977	-0.0744932	-0.2846234
-0.2332984	-0.2293295	-0.232107	0.07935086	0.16717509	0.13216437	0.09191741
-0.4832462	-0.627919	-0.0661222	0.04050266	0.03834014	0.00271525	0.02999882
-0.1832401	-0.1983127	0.14042184	-0.0675178	0.10867464	-0.0709452	-0.0736137
-0.3622688	-0.3650129	-0.3762628	-0.4434014	-0.0344592	-0.0626401	-0.157102
-0.6351073	-0.3473229	-0.263176	-0.0668733	0.0463535	0.07508987	-0.0356662
0.41147994	0.22745394	0.16026231	0.07135997	0.04287937	-0.1648553	-0.0493163
2.617273	2.2762721	-1.0940193	0.15506571	0.06059127	-0.021728	-0.3108734
2.0615204	1.3981042	-0.1449537	-0.2913618	-6.13E-05	0.05286427	-0.1744255
1.591337	0.86399947	-0.4747474	-0.1732495	0.06263441	0.00857182	-0.3392216
0.14313649	0.15172601	0.0111377	-0.0274357	-0.0313758	-0.0949704	-0.1771594
-0.4462213	-0.3379087	-0.0452873	0.0294731	-0.059859	-0.1804893	0.01833189
0.52819164	0.49679682	0.31953966	-0.53037	-0.126773	-0.0308188	-0.1692892
-2.3200511	-2.8412208	-1.5139197	-0.3592942	-0.1355417	0.28241614	-0.9332954
0.07500371	-0.0976483	0.09654758	0.12766959	-0.0727089	-0.273948	-0.0422508
1.2672208	1.3349562	-0.0360018	0.09193361	0.07564391	0.03164342	-0.0780578
0.04179142	0.05920684	-0.0203447	-0.1906725	-0.0353537	-0.1184644	-0.116532
-0.2914359	-0.3455108	0.09674951	-0.1580554	-0.0266572	-0.0147907	-0.2200726
0.57326294	0.3883518	0.10885167	0.36942187	0.16498017	-0.5615426	-0.0659
-1.8320662	-1.8238028	0.16648375	0.26884893	-0.0086856	-0.0171526	0.11063629
0.57107722	0.82682133	-0.2298277	0.1405983	0.17920815	0.08328294	-0.1074589
0.01936349	-0.1081342	0.30338093	-0.1137633	0.07634186	-0.1169651	0.04994557

-0.004343	-0.1396769	0.33780225	-0.0867393	0.18522893	0.16230807	0.04809813
0.22479	0.1982	-0.20769	-0.16049	0.12619	0.040337	-0.088989
-0.908239	-1.1648487	-0.0253291	-0.1780077	-0.0538096	0.12676514	0.04618976
-0.5463751	-0.4130871	0.14436843	-0.0582196	-0.1342199	-0.0808509	-0.1547113
1.3253791	1.3251363	-0.0518802	-0.5853989	-0.1037335	-0.0143657	-0.0759977
-0.1796399	-0.3806291	0.19746694	-0.0414573	0.08145472	-0.4209676	-0.1429663
1.4324534	1.0942884	0.22294736	-0.3818946	-0.0087564	-0.1297131	-0.0440151
0.98933	0.49534	0.45284	-0.29215	0.061121	-0.17996	-0.07613
0.11618927	0.08117495	-0.3321789	0.2218376	0.1580941	0.01787196	0.23265639
-0.1824824	-0.1247831	0.03678693	-0.30574	0.03657319	0.0661213	0.08552193
-0.4439004	-0.4009872	-0.112649	0.3732543	0.00201706	-0.0873591	0.0073147
-0.5177731	-0.2375875	0.01123251	-0.1083389	0.16838541	0.09591993	0.10461218
-0.5169979	-0.6862848	0.08820684	0.05652373	-0.091472	-0.2716942	0.14437364
-0.1546568	-0.1175303	-0.1110544	0.48030783	0.05221665	0.11787952	-0.0006551
-0.2278583	-0.1790388	-0.1897511	-0.1384526	0.12466165	0.15580819	0.04779357
-0.0821651	-0.0585321	0.08395724	0.10219986	-0.112708	-0.0752892	0.12094048
0.97513862	0.98540477	0.06647377	-0.0901739	-0.1372175	-0.3199423	0.00355035
-0.3806518	-0.5336043	-0.1702888	-0.0845636	-0.0853154	-0.0176035	-0.1605114
-0.8107749	-0.9747309	0.00487013	-0.9720848	-0.3071297	0.00618155	-0.0271191
0.50827851	0.42177408	-0.0089335	-0.1230924	0.16110869	0.08030071	-0.2833456
-0.2294173	-0.4546146	-0.2022754	-0.3219254	0.18004537	0.06788763	-0.06631
0.55098632	0.43029144	0.10051828	-0.2657564	0.02911448	-0.0584322	-0.0224659
-0.6768633	-0.5671098	0.08705123	0.16334	0.19372866	0.21457543	-0.021524
-0.5090293	-0.5926295	-0.4840645	-0.6125834	0.03550913	-0.0065044	-0.0428753
-0.0445116	0.04481764	-0.1829384	0.20578615	0.10051502	-0.0129954	0.07965122
0.17315299	-0.1211115	0.08766939	-0.2137172	-0.0224373	0.00599567	-0.2395285
-0.8078609	-0.7687951	0.05079746	-0.3455781	-0.0928788	-0.0213845	0.01824761
0.04022326	0.12924226	0.01764683	0.13982	-0.1395733	-0.0825431	-0.0755124
0.73039048	1.2385812	-0.0052845	2.4226683	0.04347269	-0.0920902	-0.0793171
1.1022842	1.5899112	0.25637542	0.00501135	-0.1473747	-0.0788174	-0.1022774
-0.026383	0.02833862	0.06914477	-0.0944671	-0.0339415	-0.2636575	0.02768885
-0.104442	-0.0964422	-0.4037339	-0.0189963	0.14824363	0.1554866	0.12889897
-0.0668939	-0.2142053	-0.1764765	-0.90196	-0.0108021	-0.1641483	-0.0560584
0.44376015	0.38486053	-0.2824863	-0.2753201	0.12562663	0.11165195	-0.1556161
-0.1682292	-0.2907754	-0.0986805	-0.1454988	-0.0671464	0.12853411	-0.1200888
-0.3906565	-0.2202185	0.01600669	-0.1111261	0.04032611	0.06371334	-0.223532
-0.2266514	-0.1095626	0.24290807	0.03542057	-0.0754974	0.10041812	-0.2943622
1.0740872	1.2789987	0.27648971	-0.1461436	0.18619889	0.04670302	-0.3101541
-0.5035567	0.00895989	0.08085352	-0.0743965	-0.117431	-0.1471095	-0.0530791
0.56496575	0.43284756	-0.1860504	0.04450311	0.0362951	0.07007949	-0.0327432
-0.3950202	-0.5880433	-0.0676075	-0.4508357	-0.1293967	-0.076304	-0.1754976
-0.2141462	0.08819597	0.26581175	-0.2215532	0.15718061	0.13391749	0.02622839

1.8416443	1.9717191	0.33006241	-0.0861903	-0.1406661	-0.1058455	-0.2289408
0.11801797	0.5076035	0.277349	0.27031247	0.0106034	-0.0073936	-0.0053946
-0.2680077	-0.338545	-0.0034407	-0.0186507	-0.2907159	-0.254446	0.13909023
-0.7651447	-0.6256453	-0.4838193	0.12054466	-0.0419579	0.04352828	-0.1248872
0.27079207	0.25565417	-0.1089954	0.06623353	0.06783514	0.08390677	-0.0483355
0.30858693	0.41403348	0.25855212	-0.1290893	0.00127251	0.02940986	-0.050082
0.07165119	0.0920194	-0.2115557	0.03225534	-0.1440844	-0.1547769	-0.0537962
0.07552809	0.01334343	-0.0808883	-0.0903466	0.0491156	0.0548881	-0.0179807
-0.1307999	0.04149997	0.02546927	-0.2048095	0.01992123	0.02269539	-0.0287387
0.50855991	0.42826155	-0.0942026	0.13626	-0.0178581	-0.0339275	0.09179172
0.15327185	0.01604543	-0.0690071	0.18244003	0.08407617	-0.0424747	-0.0549818
-0.0002921	-0.1411963	-0.2795108	0.16496227	-0.122578	-0.2438399	-0.0166458
0.09460859	0.05402984	0.1120408	-0.0487033	0.04655323	-0.0577783	0.16733249
0.3384917	-0.0215407	-0.0906999	0.093113	0.09355805	0.25434199	-0.3162006
-0.2662331	-0.3943454	-0.1170304	0.13221705	-0.0856106	-0.2384893	-0.0576657
-0.2514136	-0.375988	-0.0299098	0.15741935	-0.0630314	-0.0561345	-0.0005502
-0.3611703	-0.4245783	0.12840296	-0.1856143	-0.1126343	-0.1613769	0.05184302
0.08020951	0.20509928	0.23496415	-0.1857551	0.07267463	0.0675489	-0.1273395
-0.0015903	-0.4177119	0.4138535	0.10145573	-0.2502649	-0.0649228	-0.0989129
0.51140331	0.77841426	0.26552037	0.01734731	0.46561253	0.18641541	-0.0453393

test_GSE732	ref_GSE7326	test_GSE732	test_GSE732	test_GSE739	test_GSE765	test_GSE765
ref_GSE7326	ref_GSE7326	ref_GSE7326	ref_GSE7326	ref_GSE7398	ref_GSE7656	ref_GSE7656
GSE7326	GSE7326	GSE7326	GSE7326	GSE7398	GSE7656	GSE7656
GEO	GEO	GEO	GEO	GEO	GEO	GEO
affymetrix	affymetrix	affymetrix	affymetrix	affymetrix	affymetrix	affymetrix
,PaeR7I R:+1	TIME:110,TEMPERATURE:		PaeR7I M:+1	fis_b3261:-1,	NaCl:0.3,GLY	TEMPERATU
1664	1669	1670	1673	1688	1711	1712
0.23939502	-0.7025853	0.23915956	-0.1089158	-0.3245487	-0.016783	0.02920942
0.00828335	0.57432491	0.11095362	0.04884363	0.12906926	-0.4470147	-0.2586741
-0.1122627	2.314372	0.08192906	0.11021993	-0.1062897	0.29741225	0.91336378
-0.0018323	-0.1355059	0.04744897	-0.0444934	-0.2759602	0.0651347	0.05105765
-0.0471852	-0.1250333	0.15468666	-0.045242	-0.3342586	0.16301278	-0.2655721
0.09199612	0.06595102	0.21500406	0.27583809	-0.2785281	0.14704485	-0.3746674
0.1815438	-0.8017303	0.16180554	-0.2754695	-1.9431862	-0.215742	1.2502764
0.23743597	-0.3817709	0.45687044	0.73004184	2.2803541	0.37419388	0.23269809
-0.0865982	0.21353458	-0.0726485	-0.1095165	-0.037913	0.13425221	-0.1063808
0.01532393	0.11241397	0.14032317	0.17257633	0.17838968	1.1308899	0.43841399
-0.0126914	-0.292674	0.21462569	-0.0125616	0.54142077	0.41081306	0.29010353
-0.1303032	0.5950751	-0.0189366	-0.2580618	2.3650188	0.81854199	0.72648262
-0.0048041	-0.4889773	0.12478896	0.09978383	-0.3538026	0.26656878	-0.1178294
-0.2641875	-0.0787219	0.08716229	-0.409039	-0.4841603	-0.0472824	0.22620794
-0.0044848	-0.0797108	-0.1177005	-0.0046618	-0.1809039	0.58159406	0.19384777
0.11223344	0.44011886	0.16507075	-0.067745	-0.0470721	0.34550487	-0.0077531
0.04977885	0.44211163	-0.0087144	-0.1517866	0.13701003	0.60437	1.8012
-0.0691335	0.29706183	-0.0730699	-0.0971857	-0.0251074	0.27725776	0.71861333
0.01673565	0.16519869	-0.0044722	-0.2054785	0.22482958	0.39378258	-0.0181705
0.03890657	0.37877245	-0.0344653	-0.1211809	-0.2862255	0.01506115	-0.1010577
0.19097709	0.27919443	-0.0887502	0.04866465	-0.2290531	0.01507663	0.05246454
0.14364393	0.50714442	0.28488705	0.46857551	-0.1661013	0.15265	0.24216
0.14010638	2.1868392	0.0047276	-0.2564482	-0.4742018	0.28449437	0.36133717
-0.0107177	0.30019763	0.1367695	-0.2553175	0.55279547	0.37699	-0.2312
-0.0899728	-0.497903	-0.0454758	-0.2060585	-0.0156877	0.96347332	0.57443602
0.12176797	0.09106601	-0.0784984	0.09933635	-0.7133609	-0.0400853	-0.0792556
-0.0429699	0.51188086	-0.0853973	-0.066178	2.5968301	0.49419	0.77824
-0.0043834	0.38217346	0.14730943	-0.1816444	-0.2258343	-0.0456312	-0.0258095
0.2841187	-0.4681676	0.03811292	0.21745302	-0.6491409	0.51876	0.057861
0.06078466	-0.9757825	0.30936898	0.09042842	-0.2093475	0.60891911	0.74301984
0.10965672	-0.1354618	0.12727038	-0.146668	-0.0170144	0.05859466	0.13162733
0.18332913	-0.7335277	0.1270759	0.28227988	0.58867296	0.71056906	0.10379683

0.31288131	0.6057658	-0.1703935	0.07959048	0.42526381	-0.0339439	0.74842854
-0.0209102	0.05264842	0.03029912	-0.0843165	0.03328279	0.04345323	-0.1114399
-0.040225	1.0638822	-0.0712368	-0.2496973	1.2616207	0.53132504	0.47398021
-0.1417808	-0.0247994	-0.0165238	-0.091445	-0.0973873	-0.1171671	-0.6362865
0.03619386	0.18011011	0.04288269	-0.1355251	0.87075187	0.13178901	0.40184054
0.03475986	0.3399515	0.01263933	-0.0054707	1.087534	0.26885607	1.5652974
0.03942629	0.14273864	0.05286842	0.07451449	-0.0253024	0.16825704	1.8978997
0.02451778	0.03545736	0.07538791	-0.0367854	-0.0773177	0.10370093	0.07958331
0.15265584	-0.1112135	0.0698107	-0.0058731	-0.2591614	0.05084283	-0.1299899
0.10451221	0.06443362	-0.0261928	0.04553627	-0.0845105	0.217052	0.18587743
-0.0980978	-0.0474135	0.05997722	-0.084216	0.15352096	-0.0352389	0.05890407
-0.0455838	-0.368543	0.05283798	-0.0960574	2.6597714	-1.0388039	-0.8882991
0.01945345	-0.3631371	0.0753689	0.07656232	-0.1458046	0.16197724	0.02957496
0.07602184	-0.0962961	0.11472403	-0.0528476	-0.1260503	-0.4784553	-0.0898734
0.34063632	-0.2305303	0.07788118	0.12632894	0.09300658	-0.0028637	0.14835451
0.41119251	-0.87618	-0.02711	0.11997662	-1.7588962	0.44606424	-0.1177322
-0.0214154	0.0256301	-0.0119644	-0.0084592	-0.1583745	-0.2000056	0.2880711
0.141759	-0.2370837	-0.0320473	0.21538261	-0.0841538	0.39954252	0.48623617
0.13580507	-0.1356121	-0.033049	0.2297464	-0.0635038	-0.0933401	0.7052156
0.05481433	-0.0822379	0.04066809	-0.0208323	-0.0712194	0.0479562	1.8390549
0.17181905	0.75735732	-0.0504425	0.27711755	0.01555945	0.23566513	0.75998537
0.11169599	0.2084267	0.1462337	0.07017443	0.93181653	-0.12973	1.0043545
0.03452351	-0.2720179	0.00132826	-0.3563772	0.38760393	-0.344945	-0.1106706
0.15028857	-0.3190656	0.21806044	0.0628786	-0.6942848	-0.0217476	0.55649841
0.07824268	0.11878142	0.18754876	0.44891743	0.22453078	0.03457285	0.66697999
0.03670315	3.0789304	0.01420703	0.36624524	4.9468119	0.19027801	1.7594312
-0.1956658	-0.4492039	0.0238647	-0.1535542	-0.3035912	0.47160147	-0.4171992
-0.0905577	-0.0014198	0.15315527	0.14794785	-0.0185249	0.50165902	0.85907271
0.36834447	0.52243116	-0.1147891	0.12857991	1.0838934	0.03209943	1.1257956
0.12227714	-0.3117049	-0.1473894	0.04708033	-2.5565355	-0.1515957	-0.0203269
0.38126825	-0.53114	0.20073146	-0.174261	1.2229309	0.51741792	-0.0949605
0.0224801	0.13951658	0.12695151	0.01584121	-0.103394	-0.1549532	0.49140312
-0.0296376	0.09883508	0.15028816	0.21188766	0.13730268	0.6182023	0.19427203
-0.0050175	-0.3766863	0.01749786	-0.1069374	-0.4460366	-0.0924842	-0.8476203
-0.0119011	-0.0484349	0.03418839	-0.0905737	0.5117443	-0.108669	0.46703461
-0.0769829	-0.1769903	0.02512817	0.01971753	0.38923015	0.11845288	0.54328945
0.41785054	-0.4117235	-0.1010881	0.10396411	0.10589756	0.45064767	0.05822702
-0.141402	0.1524125	-0.0904315	-0.1537952	-0.0605635	-0.3304223	0.33742264
0.24685165	-0.4394287	0.24280706	0.3859866	0.5301694	0.61965874	0.54401459
-0.0140125	-0.2298608	0.02301143	-0.2272783	-0.1367122	-0.3191172	-0.6189252
0.08116162	-0.2402891	0.09488666	0.05429502	0.1225047	0.44757326	0.70593323
-0.1223541	1.2021918	-0.0577256	-0.2026211	-0.1088951	-0.2370888	0.75235627

0.14113894	-0.2969872	-0.0395586	0.20418414	0.71449934	0.61728745	0.10711258
0.0851205	-0.6179443	0.10728379	0.22072486	0.36814345	-0.6413418	-1.370093
0.19839269	-0.1289947	-0.0124341	0.22056504	-0.0822619	0.67150443	1.0158835
-0.0786864	0.29093612	-0.0814761	-0.0990041	0.11573205	0.74354921	1.3259195
0.02838226	-0.0547735	0.01885217	-0.1421974	0.14563687	0.48240207	0.18260696
-0.0354789	0.00367489	-0.0063087	-0.1809928	1.7262079	-0.3448538	0.19226749
0.15659693	0.12708893	0.02355772	0.13643453	-0.3058521	-0.1978293	-0.1276551
0.13991071	-0.0744692	-0.0121736	0.13811111	-0.2181507	-0.2514409	-0.2424181
0.28367111	0.0703722	-0.0644293	0.27225505	0.24612441	0.53129198	0.90907718
0.0573654	0.1269905	-0.1226784	-0.15889	0.36057277	-0.4424559	-0.4296463
0.03069018	0.05898103	0.00863375	-0.1090833	0.54225153	0.07082941	-0.0223213
0.09329007	0.24860083	-0.1048486	0.01253894	0.25593198	-0.3582078	0.55304345
0.04576863	0.74114247	0.27340136	-0.0174916	1.5226732	-0.3799471	-0.5089233
0.12309229	0.26164995	0.11337136	0.1741648	0.55566713	0.14297694	0.76684764
0.09852808	1.0031504	0.11743362	-0.179021	1.2889302	-0.37819	-0.72977
0.31973859	-0.2468357	0.1593943	0.03703859	0.12713362	-0.1505225	0.36560677
-0.0710695	0.25698234	0.0289038	-0.2041158	0.88463124	-0.2468533	0.89742681
-0.1036683	0.01839186	0.02257419	-0.075914	-0.0124394	0.1504597	0.66308498
0.13715143	0.09725423	0.09369042	0.26524431	1.658243	-0.084835	0.22327
0.10528231	0.04801769	-0.071031	0.12256467	1.0954891	-0.0203733	-0.0473267
-0.0090719	-0.0969478	0.06691269	-0.0670146	0.07129972	-0.1393976	0.01259127
-0.1462862	0.31191935	-0.0798677	0.21419607	0.47321934	-0.054227	0.18824
0.39425813	0.02540889	0.1002676	0.57142388	0.20016373	0.30156986	0.37411636
0.04013059	0.9368331	0.13356274	-0.2237966	1.3244675	-0.0381067	0.26699996
-0.0147301	0.23261421	0.06092061	-0.0799629	0.06723187	0.33077347	0.38667506
-0.0971989	-0.6093157	-0.0118781	0.37663901	-0.1317429	0.09273102	1.2247134
0.18958084	0.86658924	0.15475103	0.45675272	-0.4543643	-0.1860884	1.6131034
0.15187851	-0.389788	0.16564017	0.09793079	0.26602595	-0.4603754	-0.0990166
-0.058712	-0.21393	-0.047694	-0.079578	2.431	0.44312	-0.10096
0.014154	-0.51481	-0.090542	0.0009991	0.060104	0.45482	0.1585
0.2599	-1.1532	0.040766	0.063658	-1.9203	-0.0044973	-0.17893
-0.36759	0.017292	0.15271	0.081812	1.4515	0.10517	0.88676
-0.15468	0.27276	-0.02301	0.048009	1.2656	0.12073	1.0489
0.013812	-0.037984	0.15281	0.18377	0.02001	-0.078241	0.46846
0.20556	-0.41695	0.040761	0.51381	1.178	0.0062292	0.57277
0.05680302	0.45204767	-0.1760402	0.19807822	0.24799757	-0.0489812	-0.2090932
-0.2595064	0.19719764	-0.0993068	0.06368354	0.30785443	-0.0717185	0.0092929
0.14423795	-0.1605342	0.20834967	0.29847765	0.63111903	-0.329676	0.50688466
0.14201405	0.23536768	0.04657403	-0.005997	0.57461557	-0.1306796	0.54300298
0.12559487	0.23464113	-0.155849	0.07382742	0.04640021	0.07126084	0.13734714
0.06771727	-0.5099327	0.17647922	0.25197153	-0.6891981	1.2472874	1.2892955
0.50405597	0.16734744	0.07412711	0.3637334	0.53737841	-0.4154426	0.97367656

-0.0189138	-0.3300728	0.00544681	0.10613414	-0.3091672	-0.1940436	0.72591803
-0.022634	-0.0681885	-0.0499057	0.07354169	-0.8797284	0.06440451	-0.2314062
0.07618831	0.20955387	0.09454129	0.12059548	0.14512283	0.17590394	0.55650138
0.13766955	0.15833618	0.02605888	0.08832321	0.06731237	-0.0103789	0.53557438
-0.1733229	0.40679851	0.0076111	0.06217982	0.84648502	0.34340714	-0.0990493
-0.031296	0.26356843	0.06702661	0.19754087	1.6460565	-0.2124928	-0.0878022
0.06069744	-0.3361302	0.00618492	0.05897944	0.15491561	0.23999712	0.30324831
0.05847695	0.73731157	0.07826947	0.31919941	-0.149667	0.22559992	0.63392664
0.22258481	0.37452922	0.23228936	0.54587906	0.08025041	0.31668541	1.3599935
-0.1095567	1.4798313	0.15297193	0.336865	1.607346	0.30716543	0.9522423
0.13847293	-0.290111	-0.0086624	0.04553703	0.96989972	0.09646484	0.2231917
0.42163007	0.28574035	0.22101627	0.73476533	1.0750241	-0.2438951	0.0670604
0.01104073	0.22206811	-0.042603	-0.1899603	0.77239447	-0.4609784	-0.1753703
-0.0018833	-0.0223095	0.08937658	-0.0531081	0.74855075	-1.7649698	-0.6897631
0.07588735	-0.201375	0.00087522	-0.1651693	0.4524961	-1.8895322	-0.6213878
0.16660929	-0.2640181	0.02526631	0.16244205	-0.390809	-0.014748	-0.2666862
0.14006421	-0.5738384	0.15381994	0.30228003	-0.3749044	-0.3997976	-0.8291795
0.07375855	-1.0886559	0.13415118	0.28689267	-0.0141238	0.04634187	-0.0507194
-0.0605235	-0.1259253	-0.0223295	-0.0364899	0.30073384	0.36592463	0.69578714
0.00820783	-0.03423	-0.0578011	0.09379771	0.35941812	-1.6563899	-1.6687085
0.0824418	0.10970578	-0.0714784	0.09427577	0.1204755	0.03335607	0.43878078
-0.0583172	-0.0997191	-0.1305699	-0.1553969	0.80456647	0.15110001	-0.0463255
0.28393	0.11697	-0.028623	0.22496	0.58412	0.19314	0.55901
0.17975751	0.01556769	0.00745542	-0.1410062	0.81817922	0.24635229	0.69551082
0.16449719	0.53386268	0.09011713	0.02056736	0.87146404	0.16972	0.78262
0.13166382	0.44816921	-0.2289543	0.11045193	-0.0488837	0.39798937	0.9817463
0.08737482	-0.1876751	0.03937909	0.17639204	-0.0312965	-0.4795466	-0.8044557
0.04429982	-0.0306099	-0.1767435	0.03408092	0.03586463	-0.0124658	-0.2833727
-0.0025908	0.31313982	0.15417254	-0.0331116	0.31357574	-0.5170382	0.17768766
0.06505692	0.53873084	-0.1080024	-0.173416	1.9135151	-0.22558	0.55812
-0.0343954	1.2240732	0.24249755	0.08104698	1.1239784	-0.305956	-0.4991931
0.0055883	-0.3534867	-0.0487786	-0.0827915	0.33499314	0.72076971	0.09880995
0.02837859	-0.2922358	0.06313282	-0.1330477	0.24154383	0.1834656	0.00996024
-0.0155525	0.18149282	0.0808243	-0.1935855	0.32046771	0.08311807	-0.1388521
0.00830054	0.83178877	-0.1185413	-0.0195095	-0.3920044	-0.0416415	0.6041047
0.01742958	0.05637546	-0.0223378	0.02115354	-0.0916682	-0.2990314	-0.1315823
-0.0465458	-0.273094	0.02423591	0.08840777	1.7559112	0.03566046	0.13602013
0.01611486	0.74582541	-0.0564156	0.07076698	0.03057854	-0.2459839	0.32522753
0.08525497	0.27290984	-0.0198247	-0.0700368	0.19708572	0.08618246	-0.1399424
-0.1295484	0.91794627	0.05121729	0.11501376	-0.0342937	1.018913	1.0904892
-0.0004852	0.20920406	0.12883479	0.12948538	0.22314325	-0.0718463	-8.67E-06
0.28559014	0.69861791	-0.0733173	0.32772623	-0.0007272	0.01458318	-0.4921268

0.12890273	-0.0756925	-0.0204437	-0.1040228	0.38021171	-0.0541331	0.10209792
0.16482339	-0.0767517	0.04481261	0.29524233	0.02053491	0.21612787	-0.0345606
0.01112778	0.05717889	-0.1323864	-0.0517377	-0.1280724	-0.0054257	0.17935402
0.20078322	-0.1583462	0.12066996	-0.1339585	-1.0181891	1.030622	-0.4122623
-0.1507088	0.3658948	-0.168269	0.11824381	0.10189681	-0.2237733	-0.3535466
-0.0676441	-0.0597574	-0.0533569	-0.0453494	-0.0996491	-0.0634777	-0.2732813
0.01884461	-0.2321496	0.02176936	0.23537023	0.0832787	-0.0880543	-0.364236
-0.195887	-0.2320482	-0.2426217	0.03690494	1.0364506	-0.4630725	0.46350439
0.07957336	1.8655466	-0.0775609	-0.0518681	1.6855064	0.11394442	0.07417798
-0.0383271	0.08379549	-0.0220101	0.15275892	0.3914929	0.3244475	0.42758945
0.11106964	-0.0773816	-0.0422302	0.02727739	-0.2803939	-0.1734869	0.23797264
-0.0285758	0.18110514	0.01052029	0.01007475	-0.3521953	0.22690375	0.55990396
-0.0947091	-0.2217721	0.18755355	0.35042703	0.19064123	0.52768172	0.52310464
-0.1195118	-0.023716	-0.0912402	0.12014952	0.42723305	0.16997289	0.12334953
0.04166348	0.31361289	-0.2603019	0.04284528	0.68541152	0.48303157	0.63716874
0.18312473	-0.216188	-0.0820089	0.07745764	-0.1008432	0.44200043	0.15619172
0.07862247	1.571652	0.16331608	0.01226001	-0.5921444	1.4521856	2.2992598
0.03192048	0.26542221	0.01065619	-0.0342555	-1.4442645	0.37968513	0.21802448
0.12244961	-0.4334479	0.2345068	0.68959682	0.61420358	-0.1358017	-0.1049477
-0.0770648	-1.5177547	0.20065219	0.23045532	0.11291335	-0.0677757	-0.0926112
0.08570812	-0.0858774	0.14852327	0.14835682	0.10011361	-0.499044	-0.8529693
-0.0621639	0.57435018	-0.0446968	0.07578967	-0.081383	0.10877612	0.41958715
0.02460668	0.06442256	-0.0172624	-0.1688453	0.03264037	0.11824782	-0.4645056
-0.0505343	-0.0352971	-0.0101508	-0.1957379	-0.1829222	-0.0310949	-0.783367
0.02492668	0.03708281	-0.0067683	-0.1879205	-0.2188096	-0.1079991	0.32499176
0.03018333	0.24958289	-0.0522863	0.35436666	0.47899178	-0.1319103	-0.320068
0.12430032	0.27122227	0.01100915	-0.0031065	0.38932915	0.09795365	0.40715417
0.27748417	1.266497	0.44505234	0.39437704	1.2496075	0.37920601	0.15538866
0.0199363	0.14030908	0.18251875	0.54157274	-0.3642247	-0.2529588	-0.2893678
0.07251973	-1.0022216	-0.0211751	-0.0739007	0.06091694	0.31218731	-1.0017858
-0.0031848	-0.111051	0.01897	-0.198018	0.00877507	-0.0764664	-0.1909433
-0.0770106	0.03706851	0.15796725	0.12821614	-0.0531236	-0.1685128	-0.5310644
0.09142374	-0.0167854	0.02835132	0.16988558	0.12548264	0.02692739	0.41322905
-0.0255965	0.04098352	0.01746521	-0.0241152	0.15990392	0.13157807	-0.2176296
0.03570715	1.8034313	-0.0882505	-0.0123701	1.6389562	0.30849933	-0.5722397
0.10118421	0.05011548	0.03766991	-0.0105729	0.28304949	0.80459137	-0.0764938
0.03659905	0.18073856	0.19522202	0.19302753	0.25786775	-0.15801	-0.054668
0.007825	0.08676144	0.05548591	0.27257829	0.17726716	0.30091546	0.40969026
0.02441968	2.7836113	0.13794958	0.11574292	0.95351936	-0.0587844	-0.3691274
0.11570403	0.55370726	-0.0867948	-0.0210541	-0.2114214	-0.25321	0.40565
-0.099506	0.36128557	-0.0531444	0.32791206	1.0097448	-0.0671085	0.03965044
0.12047193	0.33249297	0.09145836	0.55402798	1.075326	0.22577418	0.93973208

0.00472789	0.42888578	-0.0507144	-0.2226335	-0.0020781	0.20454885	0.06794513
-0.1034254	-0.0043832	-0.074903	-0.0561919	0.13872012	0.0224716	-0.1377948
0.41930925	0.16169059	0.22054517	0.43750736	-0.5716418	0.91821665	0.53765155
0.0031564	-0.1478795	0.02344935	0.26469886	-0.3840078	0.08627403	-0.4725927
0.07991184	0.22500813	0.05749107	-0.1786918	0.03018024	-0.0639894	-0.1943484
-0.0003924	0.34774079	-0.1093486	-0.2859051	0.5223638	0.37790515	-0.3113783
0.15498721	-0.2712486	0.22795017	0.18339619	-0.2838203	0.57832228	-0.3508562
0.04753497	0.60955259	0.18157371	0.09319815	0.21944984	-0.0662461	-0.2190627
-0.1314669	0.12949438	-0.0714599	0.0836365	0.07877951	0.01219783	-0.201015
-0.0411443	0.04987721	-0.0321445	-0.146067	0.29832596	0.14871	0.14737
-0.0333814	-0.0476242	-0.0072406	0.14462894	-0.4488987	0.01845926	-0.237838
0.1706493	0.19526739	0.2305833	0.12561712	-0.1002837	0.94578042	0.10561262
0.09212874	0.03752278	-0.0642759	-0.0973774	-0.3566599	-0.9002532	-0.3813933
-0.0759921	0.06128613	-0.0322269	-0.0927013	0.16730724	0.80223537	0.40427701
0.10634825	-0.841525	0.35193634	-0.1703404	-4.800259	-0.9903615	-0.4717075
-0.0542895	-0.130482	0.05339241	0.02967142	-0.0504234	0.44124027	-0.2018739
-0.0572699	0.07954783	-0.2389625	0.12833991	1.1105381	0.14345971	0.63714639
0.14973836	0.31216936	0.02331156	-0.1598202	-0.0299914	0.08542441	0.23641626
0.11860312	0.09934101	0.18606925	0.14578792	-0.1499602	0.04678573	-0.3395699
0.05296447	0.06794761	0.52082666	0.43443842	-0.146304	0.56260553	-0.0224752
-0.0001195	1.1147805	-0.0964716	-0.0110805	0.62007769	-0.0008835	-0.2787294
-0.022108	0.06866333	-0.0844863	-0.1003608	-0.1082314	0.09942204	-0.0011537
-0.0342218	0.01531186	-0.1274645	-0.0709594	-0.3162624	-0.1676341	0.07989903
-0.0085314	0.12382953	0.12091786	0.09862843	0.18343579	-0.2644365	-0.631179
0.11125115	-0.0139325	-0.0007197	0.00608555	-0.2163123	0.16070966	-0.0730863
-0.0334751	-0.0567348	-0.0027375	-0.0321113	-0.4908522	0.27177112	1.0913094
-0.1758829	0.13266465	0.05669055	0.0491111	0.76100854	-0.1179051	0.02076757
0.0230322	1.8373533	0.09545412	0.03612048	2.6525576	-0.7992009	3.0152131
0.05593837	1.4812278	0.10576778	0.08201355	1.435618	0.10896707	0.42094039
-0.0244109	2.2192075	0.04181783	0.13796717	2.1729834	-0.1231923	1.6351296
-0.186605	-0.0930614	0.0453279	0.07014233	0.44348831	0.11920096	-0.4425623
-0.0883127	0.45890474	-0.0370767	0.06781916	-0.2222342	0.51764237	-0.3589717
-0.0350591	1.2493699	-0.1897037	0.12258027	4.4079563	0.17190579	0.38636964
0.09248347	-3.1741562	-0.0072806	-0.336319	1.264695	0.45567093	-0.5525792
0.08415725	0.26577223	0.1063104	0.39535336	0.11653017	0.13781088	0.17686186
0.06227165	0.38975759	0.09111733	0.06886291	0.10175984	0.05885241	0.21536657
-0.1127902	-0.2473572	-0.030902	-0.0433033	0.32753161	0.31030081	-0.3284077
0.01118122	0.34795557	-0.1742095	0.3003176	0.15096434	-0.1824909	-0.0782924
-0.0133063	0.83996916	0.08541128	0.38750672	0.73317539	-0.0007978	-0.4658242
-0.1355438	0.26374158	0.15866411	0.74662956	-1.1091717	0.21352553	0.31554127
-0.0107166	0.2975069	-0.1186182	-0.0945585	-0.3207502	0.49289002	0.31728155
-0.1019598	0.04639912	0.13291953	0.03688459	-0.0116066	0.21450657	-0.1871721

-0.0849358	0.23962226	-0.0049163	0.03699456	0.04518249	0.14515196	-0.4562714
-0.009196	0.48746	-0.07584	0.034009	0.10355	0.32866	-0.058895
0.05519865	-0.3692492	-0.1564906	-0.0963345	-0.2520595	0.59982898	0.26642065
0.12544621	-0.0617079	0.00760644	0.0904218	-0.2204388	-0.0916996	-0.4010613
0.34501515	0.00476968	0.02545012	0.04614582	-0.5447021	0.59000861	0.89156414
-0.0622591	0.64653475	0.09711326	0.36202866	0.34586609	-0.8994546	-0.4191743
0.05967658	0.01123387	0.07449846	0.20276534	0.03429483	0.70590125	0.00650413
0.0046212	0.24898	0.087263	0.10402	0.27125	1.9718	0.98235
-0.2231817	0.23937564	-0.153553	-0.2255173	-0.1478059	0.02161499	-0.0340162
0.15970424	-0.1061765	0.02840167	-0.1461524	-0.1932553	0.64532	0.43088
0.08971784	-0.1350274	-0.0203487	-0.2395427	-0.3496394	0.81125658	0.22916883
-0.0005013	0.3228729	0.15990526	0.0340953	0.08444344	-0.0922854	-0.037467
-0.0367269	0.60642627	0.17291928	-0.0732315	0.02686374	-0.0187192	0.11649317
-0.1062482	0.06474283	-0.0878384	-0.0156684	0.04711988	0.47763247	0.41554215
-0.0059689	0.3662654	-0.0581188	-0.1472309	0.14991212	0.2518682	-0.1913916
0.04355239	0.30726413	-0.1055947	-0.0448888	0.08529437	0.36317465	0.37814447
-0.049132	0.47976795	0.18361789	0.76247508	0.30813271	0.78525825	0.602839
-0.1488051	0.58436888	-0.0641282	-0.0732347	0.5427713	-0.3073791	0.22136963
0.13368613	-0.0529264	-0.0456331	-0.0385225	-1.0489569	-0.5000274	0.23681569
0.04123495	0.33259812	0.18505192	0.04972895	0.13242346	0.59593002	0.65456081
-0.2313368	0.29955238	-0.1707517	-0.1234995	-0.4627048	0.09896605	-0.008069
-0.0349701	0.77284303	-0.0678102	-0.1045781	0.62078976	0.21298988	0.46149602
-0.0447761	0.02094042	-0.2950055	-0.0854029	-0.0656469	-0.083215	-0.13005
0.09044325	0.29095349	-0.0828919	0.06282147	-0.3427523	0.24634828	0.21041348
-0.2001149	0.2761809	-0.036604	-0.0724019	-0.2086072	0.23880539	0.62691014
0.08846614	-0.042873	-0.1740251	0.06494468	0.14214706	0.66212182	0.44761435
-0.0556287	-0.4804592	0.13598118	-0.0796589	-0.7910134	0.46800193	-0.1023121
0.13954503	-0.2120246	0.06255094	0.02020397	-0.2116466	0.20399	0.40951
0.37710756	0.5361673	-0.1293751	0.39636839	-0.2009093	2.6654171	1.6771241
-0.0288682	0.11003909	0.00778934	0.07426028	0.28843276	0.12525877	0.03773277
0.07718572	1.2493641	-0.0462156	0.08025584	-0.042984	0.11070958	0.25035185
-0.0345309	0.07395573	-0.089567	-0.0782657	0.0783103	-0.1259542	-0.3225635
-0.1159646	0.40993242	-0.039473	0.01406767	0.46680681	-0.47494	-0.33773
0.06305055	0.64809766	0.06068804	0.01820872	-0.0259946	0.12994855	1.4979069
-0.0163841	1.6639466	0.05801767	0.01231287	0.65344504	0.09151343	-0.1550544
0.05685654	0.70772874	-0.0755502	0.20158755	0.04358078	-0.1744719	-0.1564603
-0.0315301	-0.1827643	-0.0218275	0.06034492	0.19079299	-0.0786206	-0.5839858
0.15686037	0.7862198	-0.2524567	0.13173367	1.2612548	0.30769218	1.0123392
0.09696942	0.01531815	0.138083	0.10472094	0.30291019	0.45538989	0.1542394
-0.0703518	0.46315757	-0.1096368	0.02196646	0.8692281	0.07308824	0.97353078
-0.0411619	0.04586914	0.09421924	0.02167054	0.23515856	0.11894301	-0.4213066
-0.0233718	0.15571659	-0.0111231	-0.1239305	0.21018716	0.19130837	0.05584972

0.08302354	0.70488538	0.17605816	-0.0478099	-0.0390042	0.70766728	0.40611759
0.11188819	0.07316864	-0.0234175	-0.0252605	-0.2821437	-0.4543172	-0.0819868
0.04922237	-0.3820624	0.1578507	0.25823735	-0.1457304	-0.0836894	-0.1373603
-0.1755218	0.25910188	-0.0301974	-0.0329785	0.04153988	0.78485342	-0.010174
0.00283696	0.41800135	0.04327536	0.0685124	0.23205981	-0.1531385	-0.2686005
-0.106182	0.14881368	0.12131324	0.34213377	0.3665708	-0.1898234	0.20852737
-0.0877426	1.0054093	0.17978326	-0.0878401	0.60970926	-0.1445332	-0.700708
0.00582353	0.33633983	0.07064813	0.02121086	0.38123329	-0.1901463	-0.5142914
0.22227088	0.06264161	0.04896492	-0.0281719	-0.2799291	-0.0784751	-0.6845565
-0.0015637	-0.1128336	0.14509331	0.42586003	-0.0336135	-0.022059	-0.45078
-0.1108308	0.20735585	0.08691289	-0.0318093	-0.0878016	0.73926003	0.13852616
-0.1134752	2.4667524	0.13528702	-0.0004366	0.98393439	-0.0703453	-0.0755567
-0.0427064	-0.1071153	0.13015415	0.03228566	-0.049898	-0.07295	-0.4738507
-0.2059777	-0.2343112	-0.0104641	0.10180301	0.44653623	-0.14596	-0.80001
-0.015031	0.01566449	0.01722954	-0.1044611	-0.1551423	0.18712125	0.21479215
0.00420883	0.31541243	-0.0843134	-0.1193824	0.61320391	0.11535468	0.19565519
0.20211648	0.09635449	-0.0429933	0.01449416	0.08955048	0.34746012	0.17789651
-0.1091027	0.57888889	-0.1206806	0.03159311	-0.0868835	-0.3468394	0.03578139
0.04526644	0.36942053	0.0245703	-0.0168358	0.27938623	0.07050481	0.03491144
-0.1227957	0.70701366	0.06055162	0.30166856	0.54798882	0.29673722	0.37911228

test_GSE765	_GSE7885_rţ	_GSE7885_rţ	_GSE7885_W	_GSE7885_W	_GSE7885_rţ	_GSE7885_rţ
ref_GSE7656	_GSE7885_W	_GSE7885_W	_GSE7885_W	_GSE7885_W	_GSE7885_W	_GSE7885_W
GSE7656	GSE7885	GSE7885	GSE7885	GSE7885	GSE7885	GSE7885
GEO	GEO	GEO	GEO	GEO	GEO	GEO
affymetrix	affymetrix	affymetrix	affymetrix	affymetrix	affymetrix	affymetrix
TEMPERATUI	rpoS_b2741:	rpoS_b2741:	BIOFILM:-1,P	BIOFILM:-1,P	rpoS_b2741:	rpoS_b2741:-
1713	1726	1727	1732	1734	1736	1737
-0.3696801	-0.2728391	-0.2847971	-0.324741	-0.3465017	-0.2610384	-0.5711684
0.40367438	0.09236002	0.37543132	-0.1880129	-0.1619428	0.44944226	0.17750057
1.3427468	0.31946029	0.8038677	0.31776065	0.28514341	0.81961761	0.6787743
0.01621095	-0.1378806	-0.2590203	-0.2960815	-0.0143933	-0.0589436	-0.2889729
-0.1058447	0.00909141	0.14288948	-0.1754326	-0.0765917	0.14206476	-0.0690416
-0.5737349	0.07797575	0.01182253	-0.226667	-0.1406714	0.29268898	0.18516505
1.679859	0.04446744	-0.2410737	-0.1597747	-0.1232435	-0.0634008	-0.072632
-0.1361402	0.91318793	0.48765047	1.048512	1.0648082	0.99358541	0.92236803
0.07853714	0.20274596	0.03232084	0.0731113	0.02594285	0.02734498	0.26595632
0.33167065	0.19039011	0.31296759	0.16638274	0.2872733	0.21406562	0.19970241
0.51613343	0.29309336	0.55369525	0.25756052	0.26289483	0.07327895	0.28026419
0.67248475	0.09590216	-0.3713384	-0.3881517	-0.3089438	0.0023749	0.04385146
-0.114313	0.01544395	0.14101935	-0.0597079	-0.0199051	0.00722427	0.057168
0.08991146	-0.0426036	-0.580163	-0.318896	-0.3187961	-0.2061301	-0.0347214
0.7846686	-0.1063614	-0.0670005	-0.066261	-0.0966035	0.09606063	-0.0825022
0.1189983	-0.3295371	-0.088348	-0.4207325	-0.2149149	-0.2429166	-0.5140766
2.24	0.45044796	-0.1451026	0.17597368	0.16449913	0.4101351	0.54422944
1.9441603	0.18611155	0.10210877	-0.2849894	-0.2555625	-0.1554457	0.08841239
-0.1400405	-0.026795	0.0729292	-0.0975164	-0.0673258	0.03751016	-0.0329021
-0.1644317	0.11626946	-0.0962793	0.17414884	0.14574644	0.15338428	0.284369
0.26366653	0.04508124	-0.0666463	-0.1163892	-0.0275427	0.17097148	-0.0098877
0.33183	0.08272104	-0.1288882	-0.1203364	-0.1833799	0.11435486	0.20540284
0.66656628	0.38136544	-0.5409921	-0.011074	0.07330855	0.42527894	0.43414363
0.23983	0.1972967	0.04179939	0.34862802	0.22571006	0.60044196	0.49801305
0.09586327	0.05558301	0.03832174	0.26847091	0.1519126	-0.089207	0.35830763
-0.0529462	-0.2293093	-0.3518003	-0.5494878	-0.3946703	-0.1813661	-0.2340612
0.071749	0.29714471	0.77289214	1.2867433	1.2897152	0.45836601	0.2625751
-0.0561568	0.62826091	0.36729751	-0.1722846	-0.1818077	0.74394745	0.72862166
-0.16181	0.1201674	-0.1451108	0.30096661	0.23278335	-0.0247168	0.00906466
0.12197598	0.77771877	1.1684618	1.4107559	1.1555505	0.44493693	0.81574153
0.42028221	0.09025226	0.21918169	0.12388048	0.0854768	0.20805637	0.27054413
-0.1786161	0.29312975	0.45082583	0.64706892	0.94816053	0.27725959	0.31621914

0.25198288	-0.3223737	-0.4422414	-0.1394625	-0.3122313	-0.2816117	-0.2562846
-0.330995	-0.1983897	-0.0658403	-0.071918	-0.2203957	-0.0316844	-0.2177478
0.20618895	0.18421618	-0.1023482	0.20418218	0.29137109	0.00581299	0.0385234
-0.3771244	0.10126397	0.07419178	0.02448956	0.02139073	0.082653	0.09745794
0.08614161	0.00286064	0.09779865	0.15349627	0.10350206	0.14018341	0.09454059
0.68560977	0.59052983	0.84298076	1.0535824	0.68177163	0.58635894	0.70890162
0.0087372	0.07691889	0.41685268	-0.0717412	0.02987787	0.08154926	-0.0555564
-0.1904306	-0.0188717	0.17067597	0.04132873	0.03603126	0.01495612	0.0535147
-0.1404898	-0.1227614	-0.0485466	-0.1674286	-0.1779589	-0.2100526	-0.2884799
-0.2946129	0.03188516	0.01229827	0.02603442	0.02867285	0.03081925	0.02536821
0.14674816	0.2710062	0.52180327	0.26573886	0.21965648	0.31284891	0.31471676
-1.5191685	2.3203385	-0.1721463	0.93890652	2.1974508	0.89671091	1.7080113
-0.1898991	-0.0652378	-0.047899	-0.0300871	-0.0549862	-0.0406228	-0.091135
-0.5375427	-0.1797348	-0.1718234	-0.2211658	-0.1396012	-0.1847524	-0.2638166
-0.349681	0.52242936	0.3252552	0.30025874	0.3850332	0.49033757	0.54833197
-0.0511812	-0.1101096	-0.1970751	-0.2184745	-0.1425817	-0.3013496	-0.042599
0.98332109	-0.1851889	-0.2185676	0.12886087	0.13639723	-0.2122047	-0.1924591
0.07530995	0.17633275	0.58551846	0.1510876	0.1837913	0.27254262	0.32666022
0.01769912	0.06032344	0.439934	0.03007325	-0.0325621	0.04241462	0.01989089
0.08651395	-0.0001841	-0.0605816	-0.050846	-0.0379961	-0.0514993	0.06028438
0.18957428	0.21325114	0.49958893	0.41321192	0.24802486	0.24282457	0.15876194
0.3833871	-0.0712609	0.15319633	0.83453702	0.91492463	-0.0671449	-0.1783409
-0.1512961	-0.2693683	-0.6642815	-0.4210952	-0.3212485	-0.3457591	-0.2619186
0.02874003	0.13211452	0.62226237	0.20747409	0.10875908	0.30328114	0.25532704
0.45514774	-0.0300224	0.0859626	0.01816676	0.06956633	0.17657921	0.10496226
1.1393758	1.9242409	0.03980209	0.78803134	1.2612092	2.4262924	1.9262065
-0.176116	1.1394644	1.1584946	0.33418129	0.45762617	1.361538	1.3060764
0.34480361	-0.1061766	0.05645241	-0.1097195	-0.0334319	-0.065054	-0.0378468
0.38040482	0.83423896	0.78048283	0.55876842	0.54784179	0.78142549	0.76992368
-0.2892382	-0.1611128	-0.0215642	-0.0127445	-0.0100675	-0.2162279	-0.1133715
-0.3458882	0.01119722	0.43341523	-0.1158884	-0.0712169	0.01431184	0.03182328
0.73339482	0.00018824	0.20279917	0.02951097	0.01543479	0.05383757	0.19101251
0.65600539	0.18578739	0.1163319	0.21778101	0.23488722	0.35528385	0.06703762
-0.1152888	-0.258715	-0.5790519	-0.2387329	-0.1147871	-0.2639787	-0.4316595
0.16413004	0.31075283	0.15467458	0.04618019	0.08512371	0.01421106	-0.0756242
0.36819674	0.0669261	0.20886272	0.49648471	0.53494398	0.24238132	0.06591752
0.14496907	0.09753992	0.06542449	-0.1370742	-0.0599066	0.08799328	-0.0396857
0.26138933	-0.0218383	-0.0028061	0.16181512	0.05811276	-0.0708194	0.10300339
-0.0016813	0.29654395	0.51812393	0.41690147	0.26490748	0.13367876	0.12082872
-0.2552622	0.40151629	0.13595917	0.09192164	0.18131918	0.38210311	0.42138745
0.79198903	-0.0490573	0.01075643	0.11576992	0.09929794	0.15471823	0.07502106
0.89536996	0.28747835	0.38968351	0.23907476	0.01557697	0.02461249	0.20240367

-0.0399016	0.71666077	0.29474157	0.92899105	0.33594816	0.99994031	0.96523185
-0.950029	0.166529	0.40809561	-0.0806707	-0.0405171	0.18121574	-0.0494869
0.20385735	0.24286424	0.6563698	0.07403964	0.20048731	0.49023424	0.37349891
0.09927187	0.26385361	-0.2641708	0.13798232	0.07931613	0.53039061	0.607444
0.42210347	-0.0505555	0.25858946	-0.0647435	-0.026527	0.13528145	0.14706311
0.23361477	0.03418195	0.26020023	0.20553442	0.66141385	0.28555495	-0.1441815
0.18726868	-0.0624294	0.11541404	-0.0695543	-0.1128473	-0.0504169	0.13896583
0.06136079	0.1316603	0.27383063	0.45268252	0.40090658	0.20230222	0.23363108
0.65372172	0.04738722	0.32368823	0.00237804	-0.0142326	0.0072509	-0.0313236
-0.2232054	-0.5870513	-1.038193	-0.7915946	-0.7220223	-0.6760598	-0.7003019
0.1893404	0.03704528	-0.0125585	0.12719257	0.02927256	-0.0054398	0.12941092
-0.0385409	-0.1004734	0.02436764	0.07474258	0.06703259	0.05416408	-0.0589487
-0.2299852	0.40169814	0.02376832	0.05477866	0.01057314	0.60142426	0.79369319
0.62934061	0.01516273	0.19614545	0.39311442	0.23476266	0.38190364	0.35718394
-0.40734	0.33941333	-0.0916082	-0.2505236	-0.1803284	0.26214903	0.43744752
0.27926696	0.1336448	0.29291236	-0.1755838	-0.1718328	-0.0564396	-0.067935
0.4041991	0.16315885	0.08205182	0.23341014	0.15945192	-0.0645826	-0.0225501
0.1295124	0.03119313	-0.1252968	0.03180093	-0.0580726	-0.0821031	0.02420758
0.18234	0.13277254	0.00272972	0.52388354	0.2084756	0.216993	0.2693753
-0.155605	0.43711862	0.18594385	0.69537796	0.78690935	0.23985596	0.1150827
0.25989396	0.0739407	-0.0220575	0.09572357	0.00615842	-0.0286506	0.07157666
0.1554	-0.0055646	0.00465928	0.01311492	-0.0163992	-0.1907665	-0.013357
0.41077495	0.08740899	0.52998689	0.42505578	0.18731802	0.35837559	0.10089264
0.29451148	-0.0720846	0.04490279	0.25623127	-0.114139	0.11250181	0.50074167
0.30681413	0.07520086	-0.0658471	-0.1436661	0.04944999	0.00096977	-0.1321676
1.0581914	0.21321416	0.21822646	0.0753641	0.05361647	0.21860961	0.27669596
1.4776981	0.37386602	0.41319728	0.08202147	0.01548015	0.33657009	0.30335047
0.0886641	0.39094085	0.82762494	0.29270097	0.1976156	0.2418479	0.46467933
-0.031123	-0.027764	0.0084524	-0.0028939	-0.021197	-0.13507	-0.098597
0.33861	0.0021843	0.29728	-0.016666	-0.018262	0.040189	-0.0013236
-0.53652	-0.078401	0.0059176	0.097503	0.038121	-0.0012946	0.076206
0.65604	1.0192	0.61401	1.4006	1.4686	0.61663	1.2645
0.68583	1.0617	0.69402	1.4633	1.5323	1.1087	1.1714
0.58815	-0.10432	-0.0012533	-0.064923	0.036573	0.074596	-0.12055
0.47463	-0.39756	-0.71139	-0.6755	-0.58819	-0.59786	-0.47981
0.44809086	0.02395314	0.39882046	0.27670812	-0.11069	0.24601	0.14183431
0.27209828	-0.4176274	-0.5420669	-0.4295831	-0.3521489	-0.386728	-0.5878245
-0.4418607	0.03727163	0.54463145	-0.2647024	-0.2330014	-0.0324462	-0.1692457
0.50592143	0.06703777	0.22897344	0.36449209	0.28906851	0.17338228	0.19042867
0.22831503	-0.5751373	-0.5781616	-0.5933406	-0.5591071	-0.4139772	-0.7101634
0.88374091	0.11887475	0.64914985	0.08318062	-0.0258229	-0.0359503	0.33532605
0.13812187	0.20463924	0.01891652	-0.0625136	0.02284287	0.20664388	-0.0005667

0.21658372	0.18541131	0.31299996	0.34820071	0.59375825	0.15002409	0.17764969
-0.0829071	-0.3944165	-0.7996772	-0.5673056	-0.4948061	-0.3190559	-0.3207218
0.66461022	-0.2264325	-0.0924177	0.01429072	0.01715639	-0.009055	-0.170234
0.34179636	0.15294432	0.21645449	0.13396733	0.09713265	0.24648384	0.20893812
-0.2199707	0.27276091	0.4272313	0.46648354	0.36108181	0.19627277	0.32961903
-0.1163483	-0.5515138	-0.8578233	-0.0252857	-0.1596743	-0.5967922	-0.7060886
0.4767389	-0.1550758	-0.1358794	-0.1760153	-0.2279819	-0.0074375	-0.264237
1.094525	0.22098803	0.22031217	0.19191833	0.08104309	0.18446412	0.43615108
1.221788	-0.0035417	0.02751427	0.10007879	0.04890413	0.07628457	0.11932928
1.0000363	0.66045302	0.73211007	0.295512	0.17840735	1.0755703	1.1447732
-0.281864	1.0675204	0.88045068	0.76943472	0.39909684	0.60502484	0.78613632
0.11707697	0.2868376	0.18530127	0.50059375	0.32751898	0.34566006	0.42126962
0.16317907	0.65878959	0.67190969	0.6101029	0.53515893	0.9408783	0.93047854
-0.3311002	0.66780854	0.60186218	0.31255616	0.13637381	0.67149121	0.75693492
-0.2989318	0.94341084	1.0718989	0.35372762	0.15801893	1.0733081	0.40664697
-0.20103	0.34681368	0.56755085	0.47912224	0.36087734	0.38830065	0.35969664
-0.2047465	0.15551172	0.33537402	0.45002162	0.20354982	0.0247651	0.24044951
0.44723788	0.19692545	0.1783334	0.21970386	0.18753931	0.08407385	0.23101692
1.2181672	0.30372378	0.30519076	0.15492226	-0.0158761	-0.0372657	0.3262229
-1.2099457	0.09957141	0.05823567	0.05796469	0.0288434	-0.1624879	-0.0649831
0.01186758	-0.035878	0.40086589	0.03625399	0.09136857	-0.0629156	-0.1636777
0.19478052	-0.2951207	-0.2348658	0.39980524	0.43098231	-0.3266079	-0.5457002
0.32317	-0.54339	-0.50325	-0.61196	-0.23928	-0.46712	-0.85098
0.87367366	-1.9907694	-2.3429618	-2.4244066	-2.2449529	-2.0615213	-2.0971768
0.57524	0.10959454	0.31565647	0.64454349	0.38232671	-0.0117654	0.02505335
0.89829619	0.01246601	-0.155825	0.02348675	-0.0175236	-0.0290003	-0.018994
-0.6777152	0.26240732	0.66970543	0.21962758	0.22160455	0.46381818	0.3315959
-0.0196973	-0.1991424	-0.5020862	-0.3734919	-0.2993631	-0.3164176	-0.2690104
0.05342466	0.43651705	0.56805642	0.18727838	-0.0169935	0.58495302	0.43021235
0.036253	-0.1560646	0.0222449	0.37927228	0.47417963	-0.0817499	-0.1801519
-0.0843632	-0.2215844	-0.4121051	-0.3487916	-0.4054231	0.08506026	-0.0206987
0.19419081	-0.3236146	-0.2732174	-0.2360296	-0.2032668	-0.3809447	-0.3399123
-0.0302914	-0.1867941	-0.0367245	-0.0887467	-0.249678	-0.1401453	-0.3177417
-0.0070503	-0.1085694	-0.0914331	-0.1741525	-0.0921583	0.02656456	-0.1133033
1.1834171	-0.0233096	-0.1502671	0.16591959	0.08369136	-0.0129395	0.08505331
-0.1252607	-0.1445121	-0.2643427	0.12326153	0.20577321	-0.3157217	-0.1862155
-0.1354339	0.64882099	0.77810802	0.93369933	0.95783481	0.88582233	0.70668961
0.36542719	-0.0785933	0.02087142	0.0580276	-0.0400486	0.38428724	0.22863248
-0.21995	0.02061916	-0.1228735	0.17910144	0.15470168	0.09688184	0.1317524
0.55871477	0.00545535	-0.2499702	0.39258638	0.49932539	-0.1420913	-0.0815655
-0.1950708	-0.0502916	-0.3076244	-0.1405411	-0.0991793	0.05710882	-0.2490415
-0.2705678	0.55281639	1.1954284	0.30177356	0.13905275	0.13864793	0.55013076

0.06656123	-0.1812918	-0.2425414	-0.2835624	-0.1626504	-0.4610969	-0.3954924
-0.0617818	0.1126114	0.34169927	0.09996839	0.14080903	0.08213275	0.18786915
0.1484425	0.00865595	-0.2807439	0.14759663	0.03644517	-0.1805161	0.08610609
-0.273925	-0.4229534	-0.3603813	-0.4798173	-0.3901556	-0.1367824	-0.4918024
0.26582696	-0.0825899	-0.3033149	-0.2033732	-0.1189005	0.00176353	-0.1082103
-0.1118374	-0.1057965	-0.2531194	-0.0615244	-0.039033	-0.046884	-0.0915074
-0.5154693	0.22570326	0.0615385	0.22149318	0.12662763	0.26685018	0.17801292
-0.1424347	0.33251702	-0.4994861	-0.1823704	-0.0595917	0.06266156	0.37655022
0.69923676	0.35611813	-0.1188889	-0.0893568	0.09858413	0.71909838	0.712087
0.18794773	0.02521988	0.21341271	0.27362695	0.19766991	0.02314097	0.04674066
0.15047564	0.04996918	0.0650907	0.21796039	0.19934329	-0.0631554	-0.1042796
0.42002281	-0.2130018	0.10326285	-0.0174322	-0.1699708	-0.1126389	-0.0599592
0.43549884	-0.0026523	-0.0943805	-0.0879106	-0.0146251	0.24574439	-0.0103398
0.13171485	-0.0636354	0.12772367	-0.0522035	-0.1013037	-0.1162928	-0.1014979
0.24010705	-0.7006543	-1.0104917	-0.99348	-0.9166625	-0.7568876	-0.8232597
-0.1853283	0.00846251	-0.0091785	0.16511932	-0.0361618	-0.0565901	0.14952137
2.5389651	-0.0865148	-0.0937981	-0.2339252	-0.3691273	-0.0388217	-0.1270396
0.17101794	0.48021339	0.17850118	-0.2080681	-0.299066	0.02671744	0.13695758
-0.1781387	0.55525169	0.43194906	0.11128541	0.23924249	0.51312566	0.40063138
-0.181867	-0.1790404	0.33124352	0.00295741	-0.1501347	0.05032358	-0.0336475
-0.7833272	0.08704819	0.17017485	-0.0845387	-0.1773121	0.40622389	0.28925441
0.59382856	-0.1202856	-0.2783612	0.00881728	-0.0530334	-0.2109221	-0.0504673
-0.0389073	-0.0789468	0.28351449	-0.0745741	-0.0511895	-0.1690256	0.18576832
-0.6060133	-0.221765	-0.1924248	-0.1449334	-0.0806191	-0.1794942	-0.0921346
0.01822852	-0.0081483	-0.0032482	0.21113831	0.30714629	-0.155414	-0.1257449
-0.2823184	-0.0669493	-0.0748723	-0.027884	-0.0161237	-0.1080361	-0.0648292
0.33773466	-0.0102394	-0.1375677	-0.0854832	0.00229011	0.15208229	0.02705974
-0.0293236	0.43041029	0.81005961	0.07552743	0.14975563	1.1285762	0.96464233
-0.2326623	0.21895707	0.42813983	0.39684865	0.52838916	0.27444882	0.24328484
-0.5151081	-0.1925583	-0.2080081	-0.0945136	-0.1750535	-0.1805104	-0.2849241
-0.2180482	-0.0325953	0.04607899	0.01791015	0.02835223	0.02819015	-0.0106657
-0.5415967	0.09387072	0.1711808	0.01544377	0.04634969	0.06027255	0.03265296
-0.1511015	-0.0265463	0.343069	-0.1208573	-0.0175671	0.10159156	-0.0844723
-0.3703439	0.07723763	0.0820915	0.05500563	0.01829736	0.14176023	0.07531007
-0.2643262	0.40204842	0.28299999	0.4711825	0.52381507	0.75212626	0.6521356
0.03212254	0.02490198	0.29048097	-0.0312496	-0.0440792	-0.1541172	0.04103592
-0.34362	0.01676992	0.13610832	-0.0315729	-0.0080819	0.07088383	0.18351453
-0.0435957	0.15146623	0.69735807	0.39164495	0.18042924	0.23396534	0.39223529
0.13681169	0.85111975	0.09813165	0.57548765	0.44138623	1.2151502	1.2386987
0.52361	-0.0190436	-0.1359431	0.28947825	0.26240029	-0.1520642	-0.1876176
0.13553767	0.13697477	0.086046	0.38091741	0.95888812	0.35016076	0.29860325
0.77582662	0.4412528	0.1278732	0.74395951	0.96028548	0.61881737	0.58010332

0.00458625	-0.2501173	-0.6030086	-0.2811854	-0.2908692	-0.3993385	-0.4385376
-0.0380386	0.22721571	0.73393254	0.18572827	0.13815305	0.06463961	0.11324927
0.79332207	0.00295059	0.12827474	0.04568214	0.11960285	-0.0061699	0.05581356
-0.3869448	-0.2192367	-0.04299	-0.3293403	-0.2314947	-0.1877479	-0.3352365
-0.1425176	-0.127951	-0.0224619	0.04656281	-0.0745781	-0.1782263	0.04479385
-0.3538633	-0.5075285	-0.7217315	-0.6760516	-0.594536	-0.5125434	-0.5928735
-0.0560374	0.08318532	0.3009409	-0.0164417	0.02543729	0.0527897	0.18462671
-0.4610898	-0.1098585	0.03126251	-0.0846535	-0.0763053	0.08236199	0.03813485
-0.364961	-0.0342919	0.08067301	0.04192098	0.03296905	0.07951013	0.02261596
-0.015948	0.42943095	0.2462764	0.19362438	0.21925277	0.29981949	0.49201095
-0.2064551	-0.3278042	-0.0735822	-0.2557155	-0.2531538	-0.0698278	-0.3815712
0.26971556	0.37206467	0.85257356	0.20501219	0.11316773	0.27219966	0.39021128
-0.7079198	0.45416313	0.24964737	0.19954748	-0.0072988	0.47748179	0.41584996
0.07827625	-0.0061877	0.23472133	0.15412515	0.03638572	0.09834089	0.07743349
0.03242171	0.23195793	0.06213734	-0.2493836	-0.1656013	0.43495279	0.29584591
0.04979482	0.00015819	-0.0153499	-0.2421923	-0.219836	-0.0729661	-0.2423893
0.07029313	-0.041122	0.02266272	0.3223193	0.40995881	0.09383073	-0.1705363
-0.4457884	0.16785545	0.22316963	-0.2855159	-0.2105591	0.06063356	0.10157463
-0.2384908	0.34843417	0.85816995	0.61404748	0.33316523	0.29342524	0.37315862
0.34358475	0.04343062	-0.0793033	0.14518663	0.16751721	0.21943504	-0.0332257
0.79353318	0.57541226	1.097331	0.41982235	0.30056304	0.85674065	0.84390095
0.12258147	-0.1411445	-0.4257511	-0.0330674	-0.0963809	-0.2748319	-0.158856
0.19047031	0.20716595	0.11858945	0.07970217	0.11126553	-0.0942719	0.1465356
-0.2123198	-0.7249811	-1.1186049	-1.0126713	-0.9334153	-0.8363305	-0.892031
-0.2020938	-0.1221355	0.15617356	-0.2184196	-0.2302562	-0.3905283	-0.254861
1.0864717	-0.1672205	-0.2232699	-0.2761504	-0.0994905	-0.0505294	-0.2358536
-0.556691	-0.0493811	0.08530759	0.20477264	0.33834446	-0.0833789	0.09965048
-0.0268351	0.64231337	0.90836323	1.2844578	1.3668614	1.0149493	0.91879308
0.09099052	-0.0532389	0.25596614	0.56620131	0.23974838	-0.1405624	-0.0387182
1.0943725	-0.0541105	0.50447321	1.1662073	1.1754858	-0.3276371	0.01780229
-0.215455	-0.192037	-0.2553604	-0.1853512	-0.0476603	-0.1603891	-0.2127486
0.1104053	-0.250378	-0.1140295	-0.2691126	-0.1480504	-0.2464226	-0.211109
0.07757932	-0.1986564	-0.1240008	1.1780277	1.2418646	-0.0832107	-0.40744
-0.597798	0.33059481	0.04972046	-0.0454427	0.17419782	-0.2729877	-0.1972463
-0.1036909	-0.0704537	0.17007082	-0.1072535	0.023629	0.26625697	0.05082173
-0.0221953	0.08511345	0.48215565	-0.0015171	0.2453035	0.00268299	-0.1046145
-0.3828154	0.04284723	0.0276156	0.44260216	0.24435072	-0.1253494	0.10589519
0.08802127	0.05125216	0.53401714	0.4834776	0.71672492	0.29308709	0.29526644
0.26931102	0.54402496	-0.2379847	0.20976919	0.24422051	1.3069541	1.0231893
0.55479173	0.44866212	0.49069354	-0.0611607	0.02791929	0.46215437	0.29221706
0.56664586	-0.0237056	-0.404447	-0.1497115	-0.1239629	-0.1826599	-0.0712398
-0.0288831	-0.1173826	0.04538048	-0.1236529	-0.0314712	-0.1839624	-0.0464319

-0.0608586	-0.078016	-0.0323491	-0.0426025	-0.0572673	-0.0232643	-0.1145709
0.072929	-0.082354	-0.2048	0.036309	-0.055437	0.14676	-0.0029056
0.52178259	0.04174672	-0.2124889	0.05450902	0.09370742	0.08059439	0.05247121
-0.2442478	-0.1253418	0.20422202	-0.2333011	-0.1747375	-0.0259102	-0.1756299
0.64056782	-0.0112173	0.07768713	0.04892008	-0.0580501	-0.0528126	-0.1295676
-0.2161889	-0.1520736	-0.0661276	-0.3280251	-0.1456611	0.00774603	-0.2427076
0.60595243	0.17491322	0.91269701	0.06078181	0.00502027	-0.1074008	-0.0210754
1.4685	0.11188	0.5656	0.08229	0.050072	0.019845	0.034029
0.74097219	-0.1311348	-0.239779	0.13242703	0.07180195	-0.2475771	-0.0772098
0.86678	-0.0520513	-0.398713	-0.1206019	-0.1013226	-0.3302361	-0.1571227
0.33153107	-0.2067791	-0.2585602	-0.0197038	0.00947094	-0.3023409	-0.0931446
0.37776796	-0.0255485	0.40889244	-0.2439662	-0.1236009	0.15378649	-0.052982
0.26127644	-0.0173691	0.16496706	-0.0463819	-0.0751364	0.26412776	0.36677795
0.7157396	-0.6777667	-1.083977	-0.7269154	-0.6322751	-0.7002786	-0.8251418
-0.3598856	-0.0788999	-0.2750418	-0.1162721	-0.053182	0.07067026	-0.07623
0.31115593	-0.0541608	0.08060764	0.00323815	0.0129315	0.2484973	0.14925503
0.55269207	0.3081504	0.45934677	0.53198718	0.54196328	0.39252154	0.41941067
0.58305982	0.10339663	0.46651763	0.25399379	0.09171205	0.30277465	0.25857531
0.13562681	-0.1404566	-0.4808296	-0.3755378	-0.3318556	-0.2612101	-0.318788
0.32810465	-0.1018135	0.29030836	-0.0294785	-0.0231915	0.02299519	0.03668858
0.09108755	0.09642352	0.52714358	0.09022017	-0.0131306	0.08406273	0.24513343
0.30663476	0.52288216	0.15199741	0.22919986	0.28881536	0.51268692	0.54627963
-0.1559	-0.1318572	-0.1142282	-0.1276642	-0.1138988	0.01753363	-0.1834875
-0.0689887	1.6819925	1.6510663	1.1166658	1.0247078	1.7249184	1.7324373
0.26441714	-0.3294569	-0.5136013	-0.3486794	-0.3463132	-0.2727663	-0.3237635
0.10037324	0.06123165	0.01090917	-0.1397633	-0.0968743	0.1000421	0.14181489
-0.3531366	0.63911528	0.83510183	0.99902078	1.0910247	0.56895438	0.60072764
-0.13835	0.08950138	0.33268556	-0.0161886	-0.1414479	-0.0812708	0.06556604
1.8560713	1.6182054	0.35372183	1.2670074	1.3620044	0.40844425	0.91187855
0.13467994	0.01094958	-0.195961	0.37182705	0.54840752	0.00043618	0.0499151
0.21226466	0.16377029	0.48463036	0.12800432	0.04847244	0.69895702	0.3950868
-0.5565874	-0.0755126	-0.0040576	-0.1485503	-0.039501	-0.1105494	-0.1511154
-0.75541	0.39613849	0.53325385	0.78140021	0.39253391	0.04714905	0.34618021
-0.3751516	-0.0984358	1.3595179	0.07533869	-0.0772043	0.02368735	-0.1567087
0.05615695	-0.6636476	-0.5290628	-0.4901825	-0.5759478	-0.2375208	-0.6692342
0.18845937	0.19135936	0.65093259	0.14644403	0.18406874	0.11490282	0.56981971
-0.5296453	-0.1044202	0.38394366	0.00453674	-0.0185593	0.13660727	-0.0926033
0.58522471	0.39390597	0.52800386	0.93054242	0.89091426	0.19256506	0.51667189
0.29389956	0.53230087	0.56014506	0.71545519	0.9186547	0.50868344	0.34010322
-0.026715	-0.2885472	-0.5522972	0.01542651	0.10008441	-0.4476554	-0.7257644
0.01354542	-0.005229	0.03899889	0.05339107	0.00235965	0.10306112	0.17480866
0.17106679	-0.2928301	-0.681146	-0.4200725	-0.2715337	-0.3421087	-0.4590425

0.42178136	0.24688272	-0.0743863	0.08761845	0.22302391	0.29895677	0.49154536
-0.2563188	0.01325401	0.13768169	-0.026985	-0.0404549	-0.1401019	-0.1694895
-0.308398	0.09403066	0.29708068	-0.007213	-0.0017637	0.01945224	-0.0967661
0.01874135	0.02466416	0.00972254	0.18430629	0.20536634	0.07551367	-0.1455241
-0.491534	-0.0502098	0.10452718	-0.146104	-0.1585521	-0.0666026	-0.2000023
-0.1580467	0.10069045	0.3835617	0.07100423	0.19023635	0.24625324	0.05298898
-0.4361371	0.4215233	0.41683446	0.13537842	0.09848745	0.58997214	0.98708951
-0.6279032	0.02925085	0.05908299	0.24303004	0.27935175	0.16171182	0.28343059
-0.4445794	0.08027007	0.10909269	0.30264769	0.36562446	0.01645081	0.13282375
-0.57758	0.03325269	0.26664129	-0.0459429	0.16970307	0.2652699	0.06456828
-0.0969895	-0.1855492	-0.2505518	-0.1238422	-0.1488034	-0.1184806	-0.1664421
0.15905791	0.04022021	0.26358436	-0.3057474	-0.3371564	0.29332453	-0.0172489
-0.294993	0.05752484	-0.0374204	0.04801529	0.07656452	-0.0126946	0.11293869
-0.61821	-0.0132257	0.00777315	0.0406174	0.0538084	-0.1060858	-0.0516589
0.10574591	-0.1723171	0.02172267	0.04123578	0.05105049	-0.1300577	-0.2402809
0.00786864	0.19529061	0.20184032	0.55795723	0.6386303	0.14317481	0.16824611
0.9091343	-0.1029484	-0.2907424	-0.2217062	-0.2255752	-0.1066029	-0.0046245
-0.467426	0.04972574	0.14162243	0.37615154	0.46336065	0.15144761	0.17001186
0.48228904	0.0251356	0.01716423	-0.1468808	-0.0547714	0.09031519	0.01393596
-0.577845	0.2554891	-0.1860343	-0.035904	0.16124269	0.26908579	0.21680921

test_GSE958	test_GSE101	test_GSE101	test_GSE101	test_GSE101	test_GSE101	test_GSE101
ref_GSE9587	ref_GSE1015	ref_GSE1015	ref_GSE1015	ref_GSE1015	ref_GSE1015	ref_GSE1015
GSE9587	GSE10158	GSE10158	GSE10158	GSE10158	GSE10158	GSE10158
GEO	GEO	GEO	GEO	GEO	GEO	GEO
affymetrix	affymetrix	affymetrix	affymetrix	affymetrix	affymetrix	affymetrix
-1,BIOFILM:-1	TIME:25	TIME:25	TIME:25	TIME:45	TIME:25,CEF	TIME:25,CEF
1752	1777	1778	1779	1783	1788	1789
-0.1862031	0.07949911	0.15905425	0.47375611	0.75368654	0.22664174	-0.0809609
0.06406427	-0.1107921	-0.3547498	-0.178612	0.16442197	-0.0773316	-0.4139553
-0.0340009	0.6453874	-0.0977549	-0.1483955	0.30925168	0.30046038	-0.0884495
-0.1861967	-0.2383689	-0.331083	-0.5350607	-0.3780416	-0.2413609	-0.1502843
-0.3423889	0.05211022	-0.0291682	0.08021028	-0.353982	0.04592963	0.22555348
-0.1260806	1.0386151	0.7605463	0.4022377	0.57247966	1.3511322	0.64370249
-0.0353066	-0.672172	-0.2568672	0.33155513	-0.3488583	-0.7514523	-0.6369541
0.38307202	1.0757643	0.95144227	0.76620735	1.2112951	1.0827466	1.0380883
-0.0048369	0.1085187	-0.2841797	-0.1938175	0.10058695	0.02324008	-0.2947671
0.04965943	0.26039295	0.46945252	-0.001005	0.05399809	0.1118962	0.59419481
0.04635406	0.20849848	-0.0612596	0.21828717	0.43563589	-0.0975969	-0.1277322
0.33247003	1.0709266	1.1640106	0.18386784	1.2151766	0.69152124	0.75606742
-0.3137735	0.01394273	0.15328742	0.11039443	0.04833588	0.03571394	-0.0582374
0.020165	-0.0876764	-0.4064133	-0.1472635	-0.0961497	-0.1257543	-0.1838787
-0.0002142	-0.0639982	-0.060776	-0.0105848	0.13769269	-0.0219964	-0.0739571
-0.0143902	0.29341471	0.34989058	0.21072201	0.15068123	0.4370791	0.36187282
-0.10551	0.4630444	0.33810524	0.11151343	-0.1494035	0.41730098	1.0085126
-0.070598	-0.1664989	-0.7125645	0.4904228	-0.2922984	-0.2193775	-0.4286963
-0.1092863	0.07987819	-0.2391474	-0.2316224	-0.173763	0.19654746	0.02236585
-0.0482018	-0.1873041	-0.5097928	-0.1712862	0.00864746	-0.3350565	-0.5833641
-0.1864371	0.22088028	0.10230031	0.39745645	0.41881389	0.05003534	0.02020684
-0.0656817	0.2180199	0.02134808	0.56332593	0.68673538	0.21497185	0.23291763
-0.1002937	0.18572452	-0.0991384	0.51721622	1.149699	0.1749165	0.04392041
0.0221477	-0.0633475	-0.1051732	-0.2165333	0.16264933	-0.0213814	0.02331754
-0.1472585	-0.5684043	0.1554084	-0.3742079	-0.0799961	-0.4262281	-0.005101
-0.145899	0.13722313	0.29951824	0.25932875	-0.118303	0.18859088	0.3647109
0.08772878	-0.1570395	0.74080156	0.02866896	-0.2974428	0.07694488	0.77609779
0.50643598	0.58989863	-0.5859217	0.23297234	0.41644806	-0.0532334	-0.0320035
-0.1194746	-0.0439128	0.21514798	-0.1230191	-0.0507928	0.2142432	0.05899475
0.29915972	0.59734777	1.1243158	1.1593981	0.22985841	0.4670325	0.97161499
0.01966953	-0.2468412	-0.0212783	0.03690741	0.05394996	-0.2190731	-0.4050942
0.00723081	0.3116193	0.27544151	0.01146958	-0.212696	0.10072791	-0.0356124

-0.0982958	0.35186045	0.32536305	0.06455085	-0.2840363	0.31244039	0.38196374
0.030751	-0.1441154	-0.1722787	-0.2845075	-0.0438699	-0.0963246	-0.1430186
-0.1461709	-0.2028101	0.25331567	-0.2903683	0.11209283	-0.3089544	0.08188196
0.00046028	0.0086472	0.11089848	0.06716588	0.06482569	0.10515539	0.1378904
-0.0978509	0.06915052	0.00968052	0.10534245	-0.0024434	0.07506151	0.09693892
0.03434018	-0.0706942	0.17759334	0.02849948	0.32926608	-0.183104	-0.0256647
-0.0973306	0.31696467	0.17840925	0.26394334	0.06175233	0.00660566	0.12173089
-0.0162524	0.05521634	0.13813021	-0.1058035	-0.3204069	0.07626378	0.02759108
-0.0528401	0.21013886	0.27906708	0.39308666	0.27510995	0.23708969	0.29194561
-0.0008805	0.08292779	0.11686144	-0.0741766	0.02253581	-0.0193313	0.00553566
0.03740936	-0.266339	-0.2798495	-0.0423174	0.1120245	-0.2898857	-0.3827014
0.55506472	0.34720065	-0.1122511	0.52940688	0.80479878	0.63315384	-1.4416571
0.01417574	-0.3919412	-0.2023552	-0.2557704	-0.1529994	-0.3395237	-0.2815159
-0.0579002	0.14624547	0.03855521	-0.4446907	-0.0613891	0.02827618	0.04895555
-0.1870622	0.26523267	-0.4157899	0.27612458	-0.6817955	-0.1171988	-0.6058407
-0.1600218	-1.0184586	-0.810413	-0.0258778	-0.8434449	-1.0949742	-0.9931208
-0.0715019	0.15825	0.34628985	0.15176364	0.24787627	0.20452126	0.36628797
-0.0366967	0.33903432	0.37671017	0.37661655	0.47812097	0.318795	0.2207571
-0.1158452	-0.1098611	-0.1743733	-0.4345263	-0.386456	-0.3302542	-0.1660939
-0.2298872	0.10140055	-0.0242612	-0.0504471	-0.0812796	0.00221065	0.1275572
-0.107701	0.54260333	0.38001395	-0.3118467	-0.192335	0.73797215	0.63938097
-0.1336308	0.15390338	0.05849573	0.01309319	-0.2841561	0.13794364	0.03742145
0.06536759	-0.1701869	-0.6182802	0.00325775	0.14072643	-0.3110679	-0.6287333
-0.1345278	-0.09184	0.15325325	-0.1608641	0.1411513	0.06225615	-0.0335322
0.10828006	0.54788403	0.38528133	0.37401682	0.24657984	0.51944768	0.32898379
0.21216122	3.217639	2.4852305	1.8167458	2.6268481	3.1870071	2.9718373
-0.045573	0.29608204	0.54745958	0.09046376	-0.2762433	-0.1921468	0.37482225
0.06142036	-0.1170747	-0.1748969	-0.1252514	-0.1667868	-0.0994339	-0.1034345
-0.1704259	-0.1059968	0.4959078	0.11296726	-0.5212576	0.13845093	0.2123733
0.00232161	0.10002048	-0.0918196	0.19964646	-0.1153032	0.05056371	-0.0043397
0.14299622	-0.1725327	-0.2051622	0.05428483	-0.3379552	-0.1428984	-0.2019501
0.10366249	0.28873482	0.51006993	0.47692306	0.63841748	0.46285924	0.43692019
-0.0319955	-0.0439465	-0.0241379	-0.1841465	0.00642414	-0.0978439	0.02736498
-0.1643859	0.07330486	0.70763528	-3.10E-05	0.89478285	0.14031557	0.70460343
0.02372524	0.15281316	0.13058797	-0.0268543	0.01282932	0.06590869	0.21721993
0.21180878	0.10522866	0.30871662	0.14536457	-0.1256	0.21914465	0.00928553
0.11814745	-0.0557958	0.32320815	-0.2551999	-0.5735867	0.11859136	0.78358474
0.07904854	0.16145778	0.08075472	0.29756026	0.36283142	0.1221901	0.11310992
-0.0944572	0.25236666	0.44422107	0.00353734	-0.2142663	0.30174502	0.43687498
0.06894582	-0.1249333	-0.4335632	-0.232181	-0.1068082	0.19190304	-0.5034794
-0.1034436	0.06651166	0.40894537	-0.2841354	-0.3170101	0.01728814	0.47757121
0.11496888	-0.3095883	0.21052871	-0.0674621	0.25344236	-0.0910139	-0.0798175

0.50870448	-0.1672511	0.99721416	0.01960723	0.41962634	-0.2287437	0.92226026
-0.0955829	-0.1924516	0.34475543	-0.2286121	-0.7705352	-0.2177886	0.11828405
-0.0846235	-0.0699803	0.29511363	0.07029778	-0.6636946	-0.0791347	-0.071105
0.31647768	-0.2712025	-0.1307472	-0.1298331	-0.0101023	0.03883381	-0.0645373
-0.0678394	-0.0175306	0.11947866	-0.0635325	-0.0679389	0.06753076	0.18008663
-0.1721682	-0.3138099	0.17648531	-0.3279923	-0.099338	-0.1174182	0.10560379
-0.121334	0.29275977	0.17339015	0.06458127	0.14787355	0.20673927	0.19627257
-0.051117	0.00897126	-0.1316934	0.11379056	-0.2926464	-0.0233351	-0.0868424
0.05886909	0.32627546	0.63231797	0.37462929	0.30345403	0.40730399	0.53104804
0.10080584	0.1478286	-0.0822674	0.06395806	0.13685856	0.22668035	-0.3265446
-0.1232365	-0.0189965	-0.2592055	-0.1215673	0.34955246	-0.0828892	-0.2252071
-0.2175364	0.25692859	0.45084815	0.37288648	-0.2118817	0.21354311	0.51071601
0.01852055	0.8594867	-0.0970411	0.0304172	0.75441372	0.54446204	0.2216936
0.05249987	0.44444618	0.68324011	0.25901114	0.51631327	0.36947035	0.64385989
-0.1695436	1.1257322	0.27585607	0.23437804	0.91688407	1.1567192	0.42258358
-0.0190951	-0.0633498	0.0087754	0.10890801	-0.3167033	-0.0285582	0.04912791
-0.0320561	0.19242209	-0.0335165	0.30921408	0.06567965	-0.0229996	-0.0376369
0.06504236	-0.3241388	-0.3739677	-0.1627955	-0.1322902	-0.2078596	-0.2843708
-0.1101434	-0.0890692	-0.0017099	0.03190136	0.06238983	-0.2360256	-0.0292927
0.33161081	0.41079384	0.35478557	0.35781065	0.1535299	0.3849015	0.39708123
0.00130332	-0.0892463	-0.1578796	-0.1712337	-0.0393169	-0.2655832	-0.1810631
0.06332365	0.16602327	0.24913928	0.05154361	0.32222059	0.10617425	0.16041037
0.12047727	0.45989912	0.32859378	0.19403869	0.15567931	0.30866963	0.48678124
0.24943478	1.1458632	0.64822525	0.5900843	0.82232142	1.1505809	1.1717774
0.05074118	-0.1072538	-0.4145422	-0.2179093	0.00745179	-0.1376491	-0.2236295
0.05693671	-0.4924632	-0.4801668	0.03200787	-0.1810533	-0.6973982	-0.4842952
0.39335809	-0.7051628	-0.7729316	-0.1271514	-1.056261	-0.9940139	-0.8734698
0.0552678	-0.0903385	-0.0912611	0.15796723	-0.2688808	-0.0685068	-0.4022816
0.15321	0.095738	0.12929	0.13343	0.1359	0.070474	0.062892
0.2026	-0.035814	0.12263	0.34729	0.16766	0.022798	0.084893
0.061005	-0.62327	-0.38991	-0.0027229	-0.53861	-0.72928	-0.65939
0.27733	-0.47259	-0.035008	-0.071633	0.67445	-0.47862	-0.19949
0.41967	-0.37625	0.20074	0.024942	0.32697	0.0070142	0.34703
0.016963	-0.26552	-0.35405	-0.091049	0.052791	-0.18438	-0.29158
0.63768	-0.23625	-0.21531	0.30401	0.4732	-0.38899	-0.5644
0.08046503	0.67751354	0.5547574	0.18697491	0.1627364	0.66900494	0.64927073
0.20744938	0.14695709	0.39762874	-0.0112906	-0.1795892	0.36112076	0.46029117
0.01780046	0.09616699	0.37115532	-0.2243668	0.21736072	0.49028657	0.39289129
0.17085747	0.45769277	0.0765169	0.36052307	0.02865002	0.42155587	0.04922184
0.01702086	0.2429572	0.04632473	0.23808723	0.07556419	0.29055496	0.12078428
0.29045513	-0.6170181	-0.5850969	0.03493989	-0.325722	-0.7972747	-0.823809
0.4043373	0.27172982	0.06956182	0.3910988	0.07641803	0.04631784	0.18806283

-0.006681	-0.1648496	-0.4507991	-0.3104196	-0.5252536	-0.3307334	-0.3682697
-0.0013688	-0.3641014	-0.4609075	-0.1688508	0.10698512	-0.2422176	-0.583448
0.09896034	0.07967816	0.22163609	0.0532586	0.11742093	0.22922176	0.1381921
0.02399776	0.148902	0.16402789	0.0244969	-0.0024109	0.10527035	0.20611794
0.32644654	0.24457996	0.79673651	0.17181059	0.59337835	0.56246358	0.81816232
0.26007309	-0.0922693	-0.2672369	0.02797587	0.1587846	-0.1237789	-0.6563455
0.22381054	0.56316109	0.211252	0.4329283	0.42700481	0.27843108	0.32713363
0.16112861	1.0717212	-0.0531939	0.74073383	0.68721112	0.6693999	-0.0437729
0.22576555	0.0783481	0.13657529	-0.0070758	0.29017344	-0.0139707	0.10271671
-0.0231616	1.0769915	0.15225383	0.11300105	0.90689383	0.82202575	0.35673432
0.15711083	1.5235149	1.6568976	1.279602	1.7345972	1.2617422	0.96726171
0.02000669	0.54846772	-0.1609755	0.49370988	0.40182874	0.28230644	-0.2928446
0.18334373	1.8058836	0.56226874	0.95053293	1.9812451	1.6727627	0.87651332
-0.1245903	-0.077368	0.08742083	0.20089185	0.04357776	0.20266176	-0.080028
0.25729129	0.27736799	0.27513639	0.56612429	0.14125041	0.40621947	0.26366902
0.27513585	0.45546352	0.31552679	0.26193924	-0.2891013	0.27804185	0.28062684
0.23335966	0.92777262	0.4466661	0.49918707	0.34284604	0.37529056	0.59294797
0.20370092	0.18540601	-0.0450662	0.05078738	-0.2503228	0.07601741	0.04352737
0.28423026	-0.1010305	-0.0970982	-0.0787956	-0.0136126	-0.1583268	-0.3573361
0.05017528	-0.1264184	0.07328466	-0.145342	-0.153138	-0.1568958	-0.0956523
-0.1085018	0.13200417	0.22456236	0.04824955	-0.1022193	0.03138619	0.1495194
-0.0712637	-0.1581135	-0.1524859	-0.1427401	-0.0954893	0.01489984	-0.0933724
-0.16641	-0.12371	0.14778	-0.058895	-0.052092	0.0099724	0.0038317
0.55698508	0.23861251	0.23746151	0.24539606	0.74306091	0.14497182	0.13693193
0.0481194	0.3938248	0.74208789	0.03153997	-0.2938412	0.69701912	0.87296937
0.194856	-0.0081715	0.45147993	0.27404507	0.09646757	0.27404784	0.39876414
-0.1554529	0.03446872	0.01421929	-0.2234238	0.04447575	-0.0474646	0.13537217
-0.0481846	0.40486848	0.10650115	0.43074847	0.43658786	0.17795874	0.12708384
0.11759134	1.2029047	0.39169139	0.89557631	0.91702612	1.2024838	0.37415272
0.03241614	0.14888455	0.226095	0.20589723	0.08412067	0.27556406	0.17444875
0.13220011	2.3429408	0.42071103	0.51211185	1.5713344	2.0234751	0.7154906
0.00944111	-0.046106	0.09483316	-0.2237189	0.15815674	0.25072435	0.22908709
-0.1040061	-0.3511317	-0.0980392	-0.0880582	0.00661003	-0.1761595	-0.107794
0.05151663	0.12484129	0.24863058	-0.1095748	0.38035531	0.23007858	0.20110188
0.38060614	0.28640906	0.07433955	0.54009721	0.32602324	0.12921821	0.05621078
-0.1440763	0.15598733	-0.1005101	-0.0259431	-0.1598377	0.01407382	-0.0776113
-0.0643852	0.41396081	0.76204338	0.32134478	0.11756273	0.25509672	0.6942929
0.1700149	0.32288637	0.22652308	0.27082996	0.32894765	0.33044313	0.19851655
-0.1387499	0.32742952	0.11999063	0.16903214	0.15002099	0.10891666	0.2777604
0.04205669	0.96094699	1.1414453	0.68168289	0.48839037	1.0147257	1.2509457
-0.0460788	0.45448278	0.54404307	0.0500989	0.2366475	0.31584919	0.58487429
0.24262204	0.56925009	0.78693442	0.55641032	0.39689368	0.25060412	0.84061685

-0.0222403	-0.1017345	-0.0850491	-0.0754415	0.03368858	-0.0589034	-0.1546404
-0.0487874	-0.0685402	0.1226632	0.22066766	-0.2432752	-0.1723015	0.13418051
0.18415104	-0.0032921	-0.1202747	-0.0826283	0.03682871	-0.025303	-0.0830364
0.06022523	0.27673643	-0.0169018	0.00363419	0.06814542	0.30831152	0.37176351
-0.1278606	0.11626705	0.07588807	0.0784886	-0.0654966	-0.0417435	-0.0614269
0.10908183	-0.1714765	-0.0622275	-0.227883	-0.3209784	-0.0645293	-0.0796829
0.1096277	0.12650355	0.12972453	-0.1093622	-0.2077249	0.39989973	0.33012795
-0.066174	0.49960122	0.6865683	0.36326912	-0.0510656	0.48985518	1.0492293
-0.06344	1.3120653	0.84739868	0.43306747	1.1647932	1.6253157	1.1765692
0.00409682	0.06720944	0.25387347	0.05127379	-0.1549013	-0.0170883	0.24375041
0.13178662	0.2093155	0.18241433	0.25414857	-0.098071	0.08587945	0.35080815
0.10896636	-0.2202388	-0.1558794	0.02345156	-0.1974183	-0.1870487	-0.1833202
-0.0178752	0.07914936	0.23938859	0.40264449	0.1092358	0.14334222	0.17029828
0.03748145	0.1207117	0.07479407	0.13982511	0.12117664	0.0214151	0.08853949
-0.1668532	0.23451516	-0.1416695	0.11707224	-0.1574752	-0.0823953	-0.0859105
0.06382267	0.04450069	-0.2302637	-0.010084	-0.3452156	-0.1985706	-0.0140218
0.23881867	-0.2453288	-0.9661493	0.3191728	0.26655206	-0.4235074	-1.0853698
0.40823151	-0.3644056	-0.3026465	-0.0815396	0.66233025	-0.516031	-0.7846477
0.0539706	0.59810963	0.93597181	0.51203121	-0.0266201	0.35252693	1.0230495
-0.1915023	0.88763796	1.8010989	0.10421212	0.9148868	0.9387917	2.401746
0.01448756	1.0884639	0.95810421	0.144302	1.2708145	1.0083635	1.5056406
-0.0274236	0.10622416	0.06977051	0.00329622	0.05291081	0.15056137	0.11180732
0.14280545	0.11932459	0.29043551	-0.0678881	0.28618884	0.09293284	0.14006774
-0.0507564	-0.1499472	-0.1274131	-0.0020903	0.16472204	-0.0127566	-0.0191171
-0.0325462	0.2185257	0.181697	0.10481624	0.29958472	0.27695746	0.11430913
0.01561428	0.43437892	0.27243683	0.3018972	0.25971333	0.38475006	0.42519264
0.01169534	0.08327694	0.2732559	0.19779622	-0.1527645	-0.1659423	0.36781351
0.16647089	2.3810493	1.0589111	0.86099345	1.6707338	1.9355909	1.6174448
0.18484977	0.22193147	-0.0752032	0.33434127	0.0491051	0.28781043	0.09120638
0.00484112	0.54343308	0.89290651	0.19442431	0.3262271	0.70065495	0.81246813
-0.0992446	0.31729943	0.35048383	0.16377889	0.16715152	0.33383438	0.22715106
0.10004412	0.33520283	0.32573355	0.26137136	0.29709537	0.2539467	0.24127321
0.03811565	0.37360949	0.06702513	0.36809232	0.11439924	-0.0314945	0.10051278
-0.114346	0.0449559	-0.0779851	0.02827047	0.03769134	-0.0430733	0.01349697
0.07369023	1.1591715	0.75655822	0.14855356	0.99721128	1.0996024	1.0505708
-0.0530716	0.21703753	0.73716059	-0.1092502	0.13750615	0.34404657	0.49308385
-0.0788199	0.27267607	0.10130408	0.02131726	0.20748126	0.13187253	0.14253432
-0.0368945	0.35823579	0.20718436	0.40217444	-0.2312913	0.13608588	0.11926219
-0.1386996	1.9827295	0.54996946	0.37651703	0.92195661	1.7197593	0.82062921
0.01880474	0.17249216	0.02358547	0.00485442	-0.4543529	-0.0011912	0.22305612
0.17978734	0.15206529	0.19240093	0.14430259	0.45110456	0.15901003	0.16238178
0.25166591	0.57909137	0.30829508	0.39583678	0.34502593	0.58418335	0.43136169

0.03788212	0.18286147	0.35052881	0.16623413	0.52370025	0.27072175	0.37455239
-0.0325068	0.00407398	0.04159174	0.19270564	0.32613337	-0.0443582	-0.1083554
0.1981659	-0.0108468	-0.1092094	0.09886611	0.02136728	-0.2931616	-0.1645411
-0.123792	0.16952218	0.42024885	0.14384819	-0.075337	0.19065466	0.3575798
0.20090334	-0.0759603	-0.1884648	-0.0578133	-0.2120742	-0.1524577	-0.2135594
0.18253419	0.10314213	-0.1044125	-0.1504244	-0.2284902	0.35415609	-0.0948325
-0.1327101	0.42669714	0.91631131	0.26286728	0.26319515	0.34718117	0.7153565
0.08518761	0.57081719	-0.2323734	-0.1083859	0.59638956	0.40333897	-0.1497704
0.00745314	0.07925985	0.02966298	-0.155499	0.0160453	-0.0143521	-0.0824645
0.4149838	0.23090698	0.23067805	-0.1231485	-0.0334016	0.10088895	0.09344594
0.04854816	-0.3754759	-0.1042414	-0.1106514	-0.1362929	-0.4209648	-0.2695116
0.01023797	-0.0292711	0.08847691	-0.1570662	-0.2374393	0.08569341	-0.0282506
0.43549722	-0.4730833	-0.5780848	-0.3187486	-0.7720209	-0.4519885	-1.0860102
0.08234004	-0.2526372	0.06595287	-0.3324964	-0.2124101	-0.2014186	-0.2144132
-0.0203914	-0.6534319	-3.054195	-0.1500566	-0.4160819	-0.9168437	-3.6308125
-0.179306	-0.1380277	-0.1972539	-0.1376853	-0.025444	-0.1194512	-0.1210428
0.05048013	0.58746993	1.3163633	0.58819942	0.54083547	0.53992652	1.6910188
0.29360596	1.1049837	0.92543495	0.53211226	0.90392085	1.2340634	0.72574901
-0.0718161	-0.1782142	0.02404166	-0.1571938	-0.4583857	0.09688607	-0.1038078
0.16020113	0.03497838	0.25196381	-0.2960494	-0.4258499	0.16487176	0.48653116
-0.0347131	0.54251274	-0.0421899	0.29648816	0.18568711	0.45100185	0.0485694
0.05284453	-0.1067197	-0.2896371	-0.0729858	-0.1876946	-0.2511271	-0.2751874
0.20473591	-0.2876432	-0.1032716	-0.3213709	-0.0232338	-0.1887855	-0.2541692
-0.0828858	0.13587257	-0.0279624	0.20048606	0.10672427	-0.0037468	-0.0151374
0.05223857	-0.1782796	0.00815065	-0.3161705	-0.6540024	-0.0921675	0.09272478
0.00345639	0.27585658	0.19852286	0.29403902	-0.1146054	0.33147326	0.22981393
-0.25142	-0.0957788	-0.1808821	-0.0852558	-0.1288587	-0.1364386	-0.0451067
-0.0013113	-0.0629973	0.09732811	-0.0786071	-0.0622645	0.01709509	0.14944984
-0.1020669	-0.0199231	0.45020299	-0.0173102	0.11859256	0.02760564	0.25806298
-0.0125142	0.19277343	0.50583738	0.09001491	-0.1011024	0.03477077	0.53995487
0.19118988	-0.001525	0.0201471	0.22513454	0.23075905	-0.0390471	-0.168807
0.11441163	0.30531073	0.31122097	-0.4166885	-0.5131044	0.44755732	0.76441575
0.16751293	-0.1431425	0.64504629	-0.0866466	0.25227255	-0.0935048	0.15781001
0.58917475	-2.0460983	-1.4303751	0.03583693	-1.7349385	-1.8498347	-2.5382439
-0.070302	0.19545098	0.69422094	0.01517346	0.16138885	0.16751365	0.67948211
-0.0011116	0.0277022	0.40223879	-0.1693637	-0.3851878	-0.0468214	0.27835333
0.04213285	-0.2967429	-0.302334	-0.2646703	-0.1218264	-0.3912535	-0.3405976
-0.1654415	0.1051531	0.74278635	0.46748234	0.54646055	0.18987267	0.3186286
-0.1287988	3.8889703	3.961585	1.7862014	3.0540399	3.9685848	4.9085432
-0.161099	-0.1885954	0.02957882	0.04621412	0.40572079	0.11922998	-0.0924839
0.02569518	-0.1789942	-0.2799749	-0.0887737	-0.3984608	-0.072504	-0.5120135
0.08394801	-0.1584297	0.05911598	-0.0810005	0.04455288	-0.1136565	-0.0180535

-0.0736677	-0.056757	-0.1401966	0.00386454	-0.2376832	-0.1940561	-0.1509863
0.15933	0.19023	0.043106	0.0071522	-0.12615	0.13134	0.10879
0.1006732	-0.7922127	-0.7689361	-0.223546	-0.2776976	-1.0134968	-1.0168046
-0.1863668	-0.049294	0.23600558	-0.0619552	-0.0011155	0.0747287	0.0438341
0.11279342	-0.191051	-0.3167409	-0.0472439	0.03434793	-0.1684355	-0.5132827
-0.1425828	0.87510913	0.4514709	0.66039197	0.44978014	0.6724768	0.6039108
0.09608922	-0.0607944	0.25256227	0.06797236	-0.0502589	-0.1022439	0.40653942
0.25049	0.30285	0.68091	-0.047353	0.21861	0.18146	0.76651
0.11464049	-0.1550997	-0.4641134	-0.3170629	-0.1427735	-0.3575345	-0.2918856
0.06002681	0.36847672	0.26815689	0.5186663	0.47897427	0.51160183	0.22572067
0.08685893	-0.0467713	0.13941396	-0.1583973	0.05485946	0.1071813	0.30925191
0.06468788	1.0150637	0.10888073	0.42659282	0.73361701	1.1523263	0.26122299
0.07472425	0.58431125	-0.2578819	-0.1086906	-0.2550166	0.54424899	-0.1878504
0.1250531	-0.1310235	-0.2419512	-0.0986646	-0.0314038	-0.179264	-0.2734262
0.06065732	0.15699737	0.11409365	0.13220443	0.38565116	0.31320577	0.22593366
-0.0546105	0.48545413	0.18450375	0.20373453	0.35968808	0.46036058	0.42477824
0.04920037	0.24296401	1.0037338	-0.126777	0.18629586	0.42257645	1.3195123
0.07494833	2.2231768	1.5729223	0.95598253	1.1492255	2.5652827	1.8369968
0.43368676	-0.39612	-1.0244292	-0.2090821	-0.1362784	-0.5332523	-1.1580562
-0.1439096	-0.0802013	-0.0674054	-0.3143136	-0.4317129	0.12358016	0.18137963
0.10499446	-0.4612055	-0.1365982	-0.5205427	-0.3017152	-0.2640588	-0.1565302
0.25522089	1.0401582	0.85911844	0.41368156	0.90668425	0.8379509	0.94045118
0.10996115	-0.1067331	0.02341624	-0.0296119	-0.0815663	-0.1023792	-0.1355522
0.4018885	0.01281157	1.4175439	-0.0827614	0.15252278	0.12997179	1.083683
0.01439631	-0.0312166	-0.4826541	-0.1745838	-0.1756157	-0.27107	-0.3745441
-0.0112982	0.07177357	-0.0631739	0.20806027	-0.2927484	-0.1241845	-0.0664526
0.34961761	0.1961541	-0.3932263	0.10468268	-0.1718185	-0.124692	-0.3654563
0.41740309	-0.2934992	-0.2312192	-0.1970241	-0.1854647	-0.304805	-0.2972819
0.16876292	0.53427797	0.36842531	0.43128505	-0.5223823	0.59236919	0.51509084
0.15146967	0.18447831	0.33536005	0.11891839	0.4281182	0.23102875	0.0490898
0.24003359	1.6754211	0.47100381	0.16685216	0.87394125	1.7962894	0.72664682
-0.0789811	-0.0395848	-0.108169	-0.071702	-0.3132489	0.04372094	0.06719037
0.05472734	-0.0601265	0.04399248	-0.3015804	-0.3565842	0.0626676	0.24671148
0.19541897	-0.0762795	-0.0293777	-0.1173331	-0.5107995	-0.0924987	0.07352267
0.09532894	1.3037358	-0.0282115	0.11822089	0.17568161	1.4090822	0.1303997
0.16078126	0.17219451	0.1573692	0.00347813	-0.0861426	0.28402701	0.13624457
-0.0324629	0.24343288	0.19723153	0.29177847	-0.0559057	0.26621696	0.1156756
-0.1393037	0.20396073	0.67225657	-0.1920247	-0.0512025	0.17181435	0.68243409
0.03453404	0.69365733	0.13149213	0.391987	0.20886389	0.44414991	0.26373503
0.02944681	-0.1258677	0.15831645	-0.1274933	-0.0909451	-0.1750844	0.15576172
0.10325063	-0.0413639	-0.3660244	-0.2817766	-0.5267976	-0.3356256	-0.3184111
0.06315451	0.09650736	0.18250937	0.18811482	0.19123187	0.13334398	-0.0082562

0.22274907	0.44277632	-0.186459	0.20393338	0.22381711	0.24923061	0.09863554
0.09939836	-0.2831729	-0.2677136	-0.3410654	-0.5138641	-0.1683768	-0.3079521
0.09117801	-0.0330654	0.38683303	-0.0059393	-0.0605202	0.03435399	0.35574301
-0.1983501	0.2540351	0.77888872	0.13790309	-0.601383	0.53458711	1.2666431
-0.0155462	0.00909941	0.12729789	-0.1326066	-0.279533	-0.0617265	0.05115281
0.01695631	0.2987007	0.45695919	0.35658845	0.25364439	0.28046965	0.42469466
0.02396472	0.71069988	0.25337155	0.17566994	0.55322086	0.40900727	0.56066696
-0.0767142	-0.1165683	-0.0555968	-0.1139336	0.00948585	-0.138621	-0.0855652
0.0776989	0.11291384	0.37283557	0.17215996	0.26811215	0.18017466	0.12228674
-0.0983889	0.39455754	0.3106739	0.24277312	0.03282942	0.31583327	0.31222588
0.00397815	0.0173509	-0.0202133	-0.1567441	0.02889738	0.03806947	0.0810885
0.09737214	1.6004761	0.5401023	0.18390801	1.0497358	1.5948388	0.87530582
-0.0347339	-0.0821953	-0.1410467	-0.1069318	-0.1563577	-0.0629597	-0.0175228
0.058175	-0.0406369	-0.0417662	0.0011327	-0.0577065	0.06323418	-0.0343226
0.03247516	-0.3001802	-0.2752369	-0.184639	-0.3518802	-0.1769938	-0.1863373
0.18295837	0.06084581	0.31164586	-0.159573	0.22966646	-0.0028537	0.24543501
0.19774921	-0.1612681	-0.439194	0.24906784	0.0854032	-0.1871079	-0.4318753
-0.0795303	0.16664738	0.69992218	0.16227951	0.26885814	0.50966171	0.84264898
-0.0054771	0.25628091	0.73582156	0.16970773	0.38974118	0.43907502	0.78385209
0.10778762	0.90353024	1.3034392	0.71021351	0.6199846	0.91722007	1.3779792

test_GSE101	test_GSE101	test_GSE101	test_GSE101	test_GSE101	test_GSE101	test_GSE101
ref_GSE1015	ref_GSE1015	ref_GSE1015	ref_GSE1015	ref_GSE1015	ref_GSE1015	ref_GSE1015
GSE10158	GSE10158	GSE10158	GSE10158	GSE10158	GSE10158	GSE10158
GEO	GEO	GEO	GEO	GEO	GEO	GEO
affymetrix	affymetrix	affymetrix	affymetrix	affymetrix	affymetrix	affymetrix
TIME:25,ME(	TIME:10,CEF!	TIME:10,CEF!	TIME:10,CEF!	TIME:25,CEF!	TIME:25,CEF!	TIME:45,CEF!
1797	1805	1806	1807	1808	1810	1813
0.12758736	0.29127424	0.48721694	0.59873075	0.12510383	0.44374078	0.27611798
-0.3384121	-0.4330493	-0.3059282	-0.3499652	0.04906172	-0.2666178	0.06896237
0.59016448	0.0772756	0.05215103	-0.4455519	1.2176764	0.22050364	0.32849785
-0.1232036	-0.2206823	-0.1380626	-0.3486786	0.04167789	-0.3049724	-0.4614775
0.03093919	0.11425054	0.09532575	0.13314363	0.33058151	0.16005319	0.14986242
1.3000435	0.56498672	0.68138598	0.31820938	0.92548633	0.93817135	0.89984754
-0.6397299	1.1849228	1.1449464	0.30059236	-0.7406661	-0.3708229	-0.555719
1.1238979	0.3303679	0.48478504	0.82377311	1.40507	0.73021098	1.5851234
0.17393052	-0.2159868	-0.1256656	-0.3653793	0.35842432	0.07545712	0.31148308
0.15937789	-0.0053371	-0.0852821	0.03319744	0.60246895	0.10784915	0.01999197
0.05262703	-0.4104015	-0.0885894	0.03958531	-0.1210424	0.13223194	-0.2487952
0.87006785	-0.317694	-0.089734	-0.1432661	1.3239989	0.31703483	0.38588581
0.09156912	0.22112228	0.22253501	0.05669138	0.12380948	0.06064849	0.04056037
-0.1200665	-0.0658546	-0.136317	-0.1821871	-0.1869885	0.04250236	-0.0879547
-0.0409724	-0.0375321	-0.1140316	-0.1860722	0.14395204	-0.0058156	-0.1649469
0.42024797	0.35213634	0.24513447	0.17981036	0.48044223	0.11638067	0.37603061
0.51316543	0.01827158	-0.0615525	0.09898767	1.2350971	0.23737477	-0.1895306
-0.2976103	-0.0065596	0.15803987	0.16958461	-0.3435029	0.22250023	-0.0665471
0.21197927	0.00436473	0.02113087	-0.1665476	0.14949637	0.07260374	0.05763473
-0.3722477	-0.3313646	-0.2457176	-0.3713926	-0.3400398	-0.1961927	-0.2251467
0.06397785	0.08891442	-0.0266961	0.40563538	0.07613971	0.38069959	0.47752201
0.25580476	0.23432628	0.23865933	0.57292096	0.16152413	0.5683354	0.58827437
0.05649028	0.13434322	0.14664262	0.34290286	0.13489142	0.93309528	0.95737835
-0.0574641	-0.3431068	-0.1325586	-0.3029597	-0.0327177	-0.150944	0.00864643
-0.5805016	-0.4244182	-0.4678816	-0.3113155	-0.3829317	-0.3067184	-0.339106
0.24903571	0.26523759	0.38785795	0.20411669	0.21417516	0.03938182	0.00381318
0.00413315	-0.3083647	-0.1616086	-0.2653409	0.30845529	-0.109882	0.37100008
0.1254808	-0.2496565	-0.2782707	0.12016442	0.51260888	0.43318541	0.3523795
0.28356417	0.39977438	0.28644487	-0.0183748	0.18816932	0.18528394	-0.1574535
0.5493321	0.87050074	0.82182518	1.1830168	0.65769985	1.2321766	1.0641427
-0.1251159	-0.0223678	0.06628048	-0.0267084	-0.2511561	-0.1168878	0.00239544
0.09460513	-0.0981119	-0.2776942	-0.1021335	0.75081918	0.08340697	-0.3291094

0.28837611	0.31295238	0.10757302	0.12560881	0.51085564	0.35166986	0.28543987
-0.1713072	-0.206293	-0.0989908	-0.269397	-0.0905249	-0.1499385	-0.1882386
-0.0391472	-0.1831951	-0.1587797	-0.1987139	0.24156034	-0.3767724	-0.19381
0.08709489	0.05067928	0.1444166	-0.0292382	0.04848698	0.08655655	0.08908788
-0.0407879	-0.0322147	0.12015198	0.09972996	-0.0532957	0.04656191	0.13416042
-0.0935008	-0.205614	-0.1396609	-0.360253	0.0265782	-0.1856496	-0.0181215
0.11151523	0.02809893	0.04257668	0.23692871	-0.001188	0.28247106	0.04486306
0.05596039	0.00636129	-0.0072186	0.04493863	0.02620567	-0.1290861	-0.1443644
0.19471038	0.30572948	0.19488735	0.32416324	-0.1183583	0.22945683	0.26960294
0.06227656	-0.0391752	-0.0284388	0.05563202	0.06638233	0.05166682	0.01448618
-0.3435245	-0.3064935	-0.0645451	-0.1692044	-0.4037492	-0.2646709	-0.1754123
1.0205005	0.84737094	0.52974259	-0.2250718	0.92208296	1.0545874	0.84941491
-0.345561	-0.0714415	-0.1542626	-0.2334269	-0.2982744	-0.3147827	-0.4022693
0.05703161	-0.2488129	-0.2256562	-0.280359	0.23003116	-0.1048286	-0.0672886
-0.2436921	-0.3488267	-0.2981155	0.16865116	-0.0671513	0.11792132	0.28888497
-1.144272	-0.2145912	0.02566158	-0.062271	-1.3782501	-0.5368023	-0.8241449
0.3652063	0.22479687	0.25265476	0.23687779	0.02328947	0.17474986	0.34407448
0.26777168	0.34963309	0.36178254	0.10100872	0.23626097	0.24305315	0.30588762
-0.1638915	-0.2355038	-0.2147294	-0.4207822	-0.1778182	-0.3412159	-0.4396592
0.0423434	0.0098963	-0.0273845	0.03727191	-0.0610228	0.08033447	0.00604379
0.966324	0.16863414	-0.081341	-0.1062451	1.4382206	0.06558997	0.20373392
0.0692149	-0.0728073	-0.0846381	-0.2356036	-0.0021916	-0.2303418	-0.0967936
-0.3234409	-0.6409195	-0.444199	-0.3794048	-0.5800686	-0.074636	-0.2884193
0.18421408	-0.0818048	0.1750016	-0.0854115	-0.0345203	-0.0037066	0.04071306
0.60429934	0.39941232	0.32738241	0.2569461	0.49173048	0.57309378	0.56137526
3.2805431	1.2980196	1.3994657	0.44777265	3.8844914	2.6985576	3.5271775
0.05911619	-0.1790077	-0.1444671	0.33210976	0.41358561	-0.2285498	-0.1840352
-0.2069467	-0.1334082	-0.3126948	-0.3526781	0.24515691	-0.313751	-0.337913
0.01301108	0.14498136	0.08970312	-0.048773	0.43854444	0.22818878	0.52221761
0.02129385	0.07231112	0.12566126	0.03895187	-0.0176026	0.04076605	-0.0398002
-0.1855909	-0.0579599	0.20186068	0.18643628	-0.1792096	-0.0304235	-0.0253903
0.51514589	0.42096323	0.46173834	0.37641306	0.3336248	0.34309827	0.53124206
0.00639289	-0.1916836	-0.1957705	-0.1777632	0.17107222	-0.1400389	-0.1656525
0.19734511	-0.0408828	-0.1094108	-0.2020919	0.573205	0.0695789	0.88544125
0.03088064	-0.0679235	0.01090427	-0.0911346	0.19556492	0.01063033	-0.02215
0.20989002	0.09640591	0.19072838	0.1878857	0.08983001	0.06599659	0.14144607
0.0010878	-0.4120147	-0.2332438	-0.2872328	0.23167972	-0.4172279	-0.416847
0.23985978	0.14450642	0.10060292	0.16357448	0.0578224	0.11541259	0.2247593
0.57382283	0.15022145	0.04239635	-0.0983127	0.72658728	0.10022867	0.13309785
0.09936006	-0.2040504	-0.110356	-0.3495587	-0.1763776	0.06341637	-0.0818671
0.10845702	-0.3590551	-0.1839816	-0.0781478	0.42739505	-0.2091108	-0.1581289
-0.0191987	-0.0076803	0.14098301	-0.0106311	-0.2019113	-0.1513588	0.01659246

-0.1425228	-0.0305011	-0.148975	-0.3516417	0.48412076	0.02442951	-0.0129779
0.04316411	0.26154895	0.02349665	0.08063912	0.37135108	-0.3330852	-0.4781796
0.01573203	0.21530813	0.18581378	0.141324	0.09658564	-0.1779099	-0.33337
0.01331331	-0.1482303	-0.1078139	-0.1398123	0.08452265	-0.0979472	0.0013469
0.2001461	-0.1578301	-0.1393163	-0.0022456	0.40636474	-0.0938238	-0.0001827
-0.2503784	-0.1812569	-0.2204553	-0.4429773	-0.1095682	-0.3475368	-0.2367044
0.31178773	0.16462	0.02709876	-0.026976	0.1301991	0.18325214	0.30117557
0.08223604	0.26052437	0.10960195	0.2066992	-0.0771553	0.07619285	0.10466476
0.40171153	0.46616079	0.42846124	0.39854658	0.19723505	0.28545867	0.22026624
0.30519241	0.39094646	0.27257002	0.24493707	0.03693728	0.09815691	-0.0300736
0.05257727	-0.2757801	-0.161955	-0.1549873	0.05065965	-0.0538801	-0.0021526
0.20844001	0.0820168	0.13849904	0.21164289	0.18431981	0.23076335	0.48448743
0.62778003	0.00996031	-0.0003836	-0.1680047	0.49307793	0.44412368	0.34199701
0.46425633	0.31137945	0.25753386	0.45852419	0.42581425	0.26236035	0.4810047
1.4458767	0.13299927	0.27506629	-0.1681056	1.2966991	0.63709473	0.66829125
-0.0422234	0.09285379	0.20078072	0.17631124	-0.1382373	-0.0805814	-0.1672338
-0.0561385	-0.3022658	-0.0797952	0.12721377	-0.3000206	-0.0434439	0.05170825
-0.2236997	-0.321196	-0.0941237	-0.1677575	-0.3221956	-0.7995898	-0.3026172
-0.1779185	-0.0626977	-0.0568685	-0.0388396	-0.031911	-0.1233754	-0.2003384
0.38358721	0.2740584	0.26261079	0.1422772	0.85139138	0.15025469	0.08832051
-0.0990013	-0.1848936	-0.034215	-0.0904379	-0.1657361	-0.1343514	-0.1143055
0.16927588	0.08330342	-0.0408436	-0.1118386	0.18724775	0.07064634	-0.0240136
0.48700474	0.02602889	-0.2020891	-0.0422747	0.55834403	0.18517298	-0.0656833
1.2993249	0.0703723	0.35881176	0.2867334	1.2670799	0.79267767	0.84019892
-0.1915001	-0.2589385	-0.1373677	-0.4021613	-0.0214219	-0.2270662	-0.185086
-0.7914301	-0.1773122	-0.0650904	-0.2422373	-0.6982949	-0.482283	-0.5159317
-1.0193569	-0.4163582	-0.2084563	-0.4308557	-0.9132503	-0.803355	-1.0229171
-0.0009189	0.1925791	0.21729556	0.29226909	-0.1741488	0.02026338	-0.1234922
0.14608	0.20629	0.081981	0.20175	0.014015	0.21404	0.23704
-0.0040937	0.12599	0.12566	0.27255	-0.12414	-0.094702	-0.13385
-0.63679	-0.12743	0.097088	-0.0019857	-0.78595	-0.38023	-0.57586
-0.22565	-0.29722	-0.21347	0.043525	-0.47455	-0.21152	-0.24485
-0.1006	-0.014853	-0.086996	0.0029832	-0.079745	-0.020218	0.045425
-0.31693	-0.21579	-0.20586	-0.32005	-0.17726	-0.26699	-0.39967
-0.42385	-0.036682	-0.23048	-0.21864	-0.10204	0.0099349	-0.23995
0.89062877	0.78951965	0.56001371	0.25069815	0.88382254	0.5554563	0.13955156
0.28657215	0.17716476	0.24145054	0.00367595	0.2498417	0.00731457	0.15450178
0.18530952	0.05849221	-0.1809718	-0.1353508	0.2034711	0.06239574	0.31789121
0.53166256	0.32115875	0.38753341	0.06488284	0.49709297	0.567381	0.35516394
0.36403538	0.21106868	0.25855406	0.12808649	0.42425345	0.18459796	0.18250166
-0.8607914	-0.1696884	0.00244651	0.5146458	-0.7884978	-0.1517144	-0.3830514
0.10143196	0.1808448	-0.0249463	-0.0114386	0.50956347	0.55460217	0.33206642

-0.2541209	-0.2475716	-0.2157418	-0.2853808	-0.2675443	-0.1498955	-0.1863397
-0.1689564	0.31301875	0.16719254	-0.0732183	-0.7147614	-0.0993519	0.06892352
0.19512502	0.20731448	0.19735593	0.1006188	0.11733356	0.03757197	0.09154295
0.23039213	0.01234981	-0.0205903	-0.0246317	0.19506676	-0.0412656	0.33860031
0.66908541	0.24663196	0.18822343	0.13403051	0.68495452	0.16244914	0.78078726
0.10945229	0.23234235	0.14969385	0.06873526	0.20334098	0.03782163	-0.1298083
0.35976568	0.17972151	0.12833038	0.1976473	0.24221575	0.25174178	0.42783398
0.83715755	0.10720161	0.32681219	0.12281302	0.01687253	0.72066456	0.93575704
0.01492608	0.0091852	0.04069645	0.0301885	0.19138808	-0.0439248	-0.0867705
1.2070911	0.09167797	0.13924458	0.01762399	1.2922134	0.60846869	0.92159055
1.3664466	0.30036913	0.54640244	1.0331932	1.4372191	1.4297812	1.6815214
0.09871104	0.19177905	0.07429981	0.24317213	0.17358287	0.43680734	0.32522211
1.6853068	0.48573425	0.87069909	0.04441563	1.6895517	1.2833144	1.4596474
0.17057783	0.09859496	0.13875162	0.05200708	-0.2891163	0.01906158	-0.3071065
0.3344447	0.33769023	0.65637283	0.17843223	-0.2650341	0.18155528	-0.7278446
0.16061037	0.08415956	0.12978009	0.25086154	0.41932812	0.14619271	0.28865037
0.86438361	0.24726695	0.24196403	0.64032531	0.99045067	0.48000866	0.39059471
0.04830069	-0.0471069	-0.0253207	0.11779567	0.29071443	0.40084657	2.333828
-0.1548199	-0.6303919	-0.3676333	-0.1603579	-0.6096142	-0.2484486	-0.3176515
-0.1492489	-0.2333374	-0.1662125	-0.1582986	-0.1892879	-0.3399492	-0.1733358
0.09039025	0.02879672	0.01418359	-0.0320047	0.09658343	-0.0347585	0.02987582
-0.163316	-0.1324946	-0.1117894	-0.0866285	-0.0203784	-0.1236476	-0.0809139
0.0050903	0.13843	0.078481	-0.11206	0.00066348	-0.22063	-0.14522
0.20868346	-0.1617198	0.20448261	0.03112744	0.04723137	0.0475302	0.25530608
0.7438902	0.26332499	0.36884145	0.65645467	0.59631088	0.51647419	0.59282082
0.39477738	0.48703927	0.30155554	0.05384769	0.61177753	0.39150672	0.4166307
0.1192735	-0.0354036	0.01161195	-0.1583076	0.1123345	-0.2427985	-0.1171965
0.2168765	0.09154738	0.20084972	0.09625718	0.43224566	0.22547259	0.05132531
1.1653995	0.58008435	0.81535282	0.16604727	0.93575441	1.0847658	1.2182276
0.17677493	0.0655028	0.27888116	0.20548894	0.23663727	0.10780581	0.18275486
2.420125	0.39373885	0.31926207	0.18514041	1.9279799	1.4477495	2.4272736
0.17286494	-0.0549308	0.07329241	0.13349743	0.06941802	-0.1060148	0.05408678
-0.1990124	0.0208181	0.03993788	-0.0347916	-0.1208037	-0.1826469	-0.2615697
0.13012843	0.06599543	0.04198645	-0.1388975	0.12417595	0.07522646	0.14059071
0.04311522	-0.0024155	0.38188398	0.34958292	-0.1980727	0.39095443	0.15626085
-0.0107867	0.15344981	0.06439773	0.09450447	-0.0749489	0.21683426	0.03932138
0.29435507	0.23605046	0.25660785	0.24039698	0.44398259	0.14858595	0.8347231
0.28833972	0.02016447	0.14323587	-0.0147371	0.36146341	0.00749346	0.41162401
0.10368534	0.047589	0.07817357	0.14979435	0.2526904	0.12381329	0.18022819
1.2230956	0.73246039	1.0806188	0.51178312	0.23179259	0.96586251	1.0428785
0.46729508	-0.0506818	0.01834685	-0.0848691	0.34085525	0.12369863	0.24110765
0.36799536	0.10152206	0.27506518	0.70884097	0.24715474	0.33519348	0.6334637

-0.0743739	-0.1040239	-0.1358446	-0.1886338	-0.0334925	-0.2487187	-0.1959454
0.01196408	0.30319492	0.11410958	0.21994129	0.05882501	0.16169122	0.10492532
-0.117779	-0.0017984	-0.0980235	-0.0895935	-0.1841534	0.05141977	0.01447785
0.21515246	0.20707633	0.41271559	0.2703391	0.35657223	-0.0389512	0.05923848
-0.0132208	-0.0273392	0.06273884	0.05423435	0.02913035	0.02350361	0.03714961
-0.1698203	-0.0834478	-0.2032293	-0.0959267	-0.0516749	-0.1507475	-0.1412738
0.28588551	0.16981004	0.04620603	-0.0657623	0.2655449	0.05556956	0.15681984
0.4057474	0.1997947	0.22445385	0.213474	0.43673178	0.27165991	-0.2764606
1.7858732	0.45268674	0.54521312	0.28098682	2.0463117	0.70146508	1.1924284
0.04755159	0.05152285	-0.1216443	-0.2037593	0.13054916	-0.2602947	-0.1225532
0.08076649	0.07725997	0.01555251	0.07708654	-0.0338355	0.17442957	0.26948787
-0.1668634	-0.1443187	0.01962195	-0.1711743	-0.3534057	-0.1261997	-0.2388176
0.05631691	0.26452325	0.15767247	0.13327669	0.24776168	0.19156191	-0.1392385
0.06983853	0.00331347	-0.0062114	0.09037111	0.23494996	0.07250127	0.1465847
-0.1669386	-0.1360653	-0.2704901	-0.1042502	0.16684219	-0.0278013	-0.0636194
-0.1495331	-0.0804357	-0.3433257	-0.0692786	0.08503016	-0.0302783	-0.3194281
-0.4393561	-0.0485792	0.15416329	0.28094261	-0.6509164	0.25795227	-0.401784
-0.6620104	-0.1369997	-0.0571999	0.39176427	-0.8875002	-0.3990411	0.03128454
0.23408766	0.18018029	0.30511413	0.54843911	0.25182889	0.29436699	0.36940347
1.2849995	0.12893342	0.20174455	0.19429481	1.5284782	0.43662295	2.3184215
1.1055664	0.17066422	0.11501551	-0.1112617	1.3555284	0.62987075	1.5229186
0.14824286	0.18126999	0.02316661	0.04829299	0.28287499	0.07751909	0.037992
0.16248502	0.00485773	0.0050412	0.06683773	0.18632175	0.04329703	0.24368726
-0.1143051	-0.0079585	-0.125675	0.09868809	-0.0762073	-0.1046096	-0.11382
0.07859074	0.18417526	0.18495758	0.28652762	0.24316541	0.17804165	0.27292108
0.37482164	0.36855292	0.26235282	0.09080512	0.64221032	0.42552236	0.20379077
-0.1155203	-0.118435	-0.2935952	-0.0177216	0.15475591	-0.031758	0.03758318
2.1018051	0.62531841	0.51784165	0.4191228	2.7031941	1.5911579	1.9033688
0.27958284	0.28675457	0.31112283	0.23704967	0.16038478	0.17133145	-0.110944
0.90962457	0.33900833	0.33203556	0.24985323	1.1044154	0.36241169	0.39126409
0.2742771	0.32300568	0.26988801	0.13892627	0.22630773	0.03985379	0.09236103
0.27779826	0.24632304	0.22297661	0.33816358	0.38265087	0.3229266	0.14069927
0.01361258	0.04544147	0.07631765	0.34217794	-0.0380705	0.32744605	0.33600537
-0.0049915	0.02655289	0.00315437	-0.0125327	0.14994613	0.13682924	0.12426928
1.444189	0.03347193	0.08344701	-0.0156845	1.7590838	0.72012827	1.1789326
0.55173362	0.1956637	0.20000064	0.2741809	0.56074426	0.05951684	0.12080456
0.25630716	0.17330734	0.29053338	0.17357027	0.14111717	0.28046735	0.2625755
0.17336114	-0.0287184	0.04232565	0.14949564	-0.1177303	0.12805566	0.00320394
2.155365	0.17510287	0.26030149	0.00846843	2.3163761	1.0846959	1.0307158
0.00674657	-0.1414576	-0.2051581	-0.0639307	0.04021489	-0.217332	-0.1862375
0.39098477	0.03094988	0.03314176	0.03166617	-0.0199053	-0.1256167	0.05166295
0.59077545	0.15346781	0.28528602	0.29750783	0.54825499	0.30559056	0.35294659

0.3310973	0.06258407	0.11148221	0.30907444	0.54291603	0.15317676	0.06737337
-0.0359587	-0.0030971	-0.1175841	0.15261358	-0.1279721	-0.0507211	-0.1149674
-0.1319831	-0.0395832	-0.0849619	0.12371337	-0.1521213	0.08101765	-0.2159515
0.31395633	0.12863773	0.06433125	0.15137919	0.71508471	0.13536234	-0.009751
-0.0504558	0.08012201	-0.1087086	0.07990919	-0.1058124	-0.0641667	-0.1603239
0.16094831	-0.4068572	-0.1856798	-0.1660939	0.15292829	0.00658111	-0.1558196
0.76246318	0.48867361	0.28761155	0.20827467	0.9614376	0.24755753	0.17725427
0.67562896	-0.1830439	-0.2055179	-0.3590734	0.12962045	0.40319742	0.7392274
-0.0176092	-0.0592311	-0.05682	-0.0670804	0.043759	-0.1471204	-0.0623581
0.24858215	-0.4735279	-0.2476178	-0.0137011	0.22984209	-0.1430208	-0.0784798
-0.2271517	0.16915918	-0.0765733	-0.0946237	-0.0458672	-0.165611	-0.2171402
0.14224619	-0.0370187	-0.2398159	-0.0397601	0.15593614	0.04491821	-0.1809972
-0.6134707	-0.3721903	-0.4480181	-0.106321	-1.0932359	-0.0872841	-0.2482222
-0.1164254	-0.0830713	-0.2175619	-0.0356092	-0.2304146	-0.2954366	-0.3444576
-0.7883245	-0.0596802	-0.113637	-0.0574176	-1.4501072	-0.4182406	-1.163
-0.0607579	-0.0361213	-0.0571131	-0.1620723	-0.0294385	-0.0234314	-0.11061
0.67151166	-0.3531502	0.423755	0.39626098	0.90280424	0.39605757	0.22026651
1.2345751	0.22435994	0.08577237	0.16441907	1.249062	0.97508241	0.98490387
-0.0199687	-0.3721081	-0.1828464	-0.2001159	0.01828922	-0.0998564	-0.1202595
0.30733406	-0.0414255	-0.0750867	-0.0855273	0.39083795	0.01434429	0.06385318
0.61785348	0.93334145	0.7944171	0.19431726	0.93854146	0.50512852	0.47613479
-0.2806057	-0.2149987	-0.2685032	0.04146944	-0.3135062	-0.0087453	-0.1049562
-0.2003795	-0.3022487	-0.2519526	-0.2114197	-0.440815	-0.3602176	-0.2478013
0.11808121	0.2189663	0.05472276	0.04579037	0.15260388	0.19743129	-0.0637207
-0.0503474	-0.0480979	-0.2223969	-0.2180884	0.2521809	-0.213622	-0.16595
0.17831907	0.33321962	0.31980953	0.38103737	0.2082989	0.3545483	0.15381411
-0.1329364	-0.1758168	-0.0691163	-0.0810443	-0.0974265	-0.0696844	-0.0514876
0.03317659	0.10899724	-0.0949303	0.00736417	0.06300514	0.06587976	0.15479226
0.11240461	0.00308737	-0.1567997	0.0446558	0.30904355	-0.0129755	0.35189316
0.07209714	-0.0077227	-0.155682	0.30859968	0.23071882	0.01831118	0.3797421
-0.0101254	-0.097557	-0.0220581	0.08258338	-0.2683135	-0.0650223	0.02229696
0.55319363	0.06707381	-0.2159074	-0.0737895	0.77053141	-0.1982481	-0.5628779
-0.1780453	-0.0281224	-0.1002221	-0.0460942	-0.0821493	-0.0365441	0.38627321
-2.0298043	0.31638516	0.04550263	0.23310269	-2.1878421	-0.2718101	-1.4547864
0.33338695	0.20917913	0.25293081	0.24992247	0.44574886	0.14173074	0.21957969
0.19958526	0.15579177	0.04263215	0.0704424	0.18548388	-0.1127086	-0.0088506
-0.3442283	-0.2346756	-0.4468473	-0.2556332	-0.3870336	-0.172032	-0.2713265
0.3374577	0.59657318	0.5059205	0.33479208	0.35793124	0.17967287	0.4718577
4.0869077	0.46293192	0.9363506	0.16063489	4.4816473	2.5857864	3.6766634
0.05081157	0.4311959	0.41070008	0.26436162	0.03172267	0.1916994	0.12344598
-0.240129	-0.0175391	-0.1085846	-0.1597234	-0.4123238	-0.1327459	-0.2575884
-0.075219	-0.0746841	-0.1399408	-0.163919	0.06437915	-0.0950967	-0.0806934

-0.1166317	-0.1096564	-0.1565172	0.09261204	-0.0528298	-0.0843617	0.00862494
0.099859	-0.067921	-0.26338	-0.11594	0.079623	0.096407	0.0024717
-1.03208	-0.4493709	-0.2077981	-0.3058216	-1.3707117	-0.4205481	-0.7486563
0.12371298	0.17964809	0.16107401	0.22597221	0.09653776	-0.0201864	0.03765937
-0.1786577	0.23960037	-0.0440311	0.11520723	-0.1384869	-0.0491511	-0.2284864
0.64323636	0.3550579	0.38668589	0.12703922	0.50949155	0.88659072	0.33906342
-0.0393761	0.0684108	0.01742766	0.16371979	0.2210616	-0.1515388	-0.0457435
0.43928	0.13071	-0.043623	0.24603	0.70583	0.062945	0.11811
-0.302674	-0.2827114	-0.6113836	-0.3046641	-0.35434	-0.2746769	-0.4452087
0.48171074	0.4207024	0.34452197	0.2342892	0.4471623	0.29155006	0.20279386
-0.107123	0.04535529	-0.0775638	0.03809598	-0.0289764	-0.0523401	-0.0558083
0.97440575	0.95280531	0.63382113	0.36917291	0.80985801	1.1619106	0.8319516
0.68656648	-0.3477286	-0.1431495	-0.248834	0.40830064	0.1133291	0.01456431
-0.2610494	-0.1083128	-0.4244541	-0.1257095	-0.2340702	-0.155082	-0.3362498
0.26362487	0.21998976	0.15007045	0.21514498	0.26998413	0.09019851	0.11991924
0.44984755	0.36152547	0.33183073	0.3762169	0.37922242	0.42064493	0.36967842
0.48139788	0.25355273	0.27839811	0.3318995	0.60743349	0.43902553	0.40063634
2.2473897	0.87565845	0.95401671	0.30378066	1.812102	1.7892255	0.96811267
-0.8417471	-0.5546344	-0.3341433	-0.3161109	-0.968947	-0.2915491	-0.5469204
0.08688029	0.0305344	-0.2212985	-0.03909	0.29915271	-0.0865183	-0.0650998
-0.1491996	-0.0813072	-0.4535717	-0.4318984	-0.0526091	-0.2368186	-0.3328175
0.88255397	0.0005621	-0.1378838	-0.1548828	1.2549788	0.68212594	0.90578438
-0.1894777	0.08636266	-0.0858131	-0.1686416	-0.2576965	-0.1613828	-0.2286064
0.30592809	-0.0552314	-0.1336111	-0.0092609	0.82087163	0.16665252	0.15032901
-0.2406097	-0.3967523	-0.4963916	-0.3030766	-0.1475558	-0.2010498	-0.1956747
-0.1288355	-0.1280472	-0.0366526	0.1407474	-0.410072	-0.0170906	-0.1079404
0.05142094	-0.2002442	-0.2250591	-0.0503181	0.19367153	-0.1505727	-0.3017948
-0.0896833	-0.1551411	-0.3157808	-0.3157248	-0.3414841	-0.2552695	-0.377235
1.0703177	0.46956235	0.47005811	0.44515174	1.128348	0.43708181	0.31899717
0.23180332	0.23364654	0.33826701	0.24016942	0.15609311	0.29682029	0.15500356
1.7013033	0.11550318	0.06435326	-0.1962045	1.9519303	0.78175163	0.76474568
0.10203972	0.09257779	0.00432797	0.05911485	0.03403655	0.10068916	-0.0253575
0.13728708	-0.0340806	0.0326712	-0.1051188	0.31864824	-0.1311977	-0.1006806
-0.0726604	-0.0701489	0.00348038	0.06480994	-0.0692293	-0.1231107	-0.1665124
1.6198545	0.09955016	0.16408282	-0.1512944	0.88266613	0.82347249	0.66487329
0.27380231	0.13982907	-0.1060351	0.02069535	0.59300072	0.27406411	0.17164824
0.3453294	0.23487958	0.11694802	0.34382981	0.09071198	0.36662326	0.03000939
0.33243804	0.10151755	0.01512677	0.2926972	0.55631193	0.15347815	0.38597592
0.43504398	0.05207354	0.41562763	0.4829936	0.34451691	0.28167549	0.04016195
-0.1034712	-0.1167035	-0.1589287	0.02512868	0.02500822	-0.069574	-0.0965449
-0.158996	-0.1856023	-0.2380948	-0.0889175	-0.07881	-0.276195	-0.5960662
0.04069988	0.13263396	0.18607657	0.04326045	0.15434034	0.10854067	0.00195296

0.36125378	-0.0660907	0.07177077	0.00848023	0.38061586	0.25069562	0.16145772
-0.1772608	0.02467969	-0.2903025	-0.2483664	-0.0835127	-0.2223355	-0.123335
0.00507745	0.08564513	0.08382176	0.1249	0.07156976	-0.0341448	-0.2312795
0.42248461	0.28918339	0.34454212	0.32782539	0.56892217	0.09506614	0.13397132
-0.047601	-0.0547392	-0.1968262	-0.1275867	0.15615006	-0.1727935	-0.1853111
0.2887236	0.23979298	0.1909529	0.09391207	0.17384372	0.15605333	0.09850869
0.48733406	-0.0805103	0.00947665	0.13661841	0.41308146	0.30895948	0.60237314
-0.0439339	-0.0689484	-0.1262271	0.00427484	-0.0714255	-0.0292071	0.01310665
0.22806651	0.3169601	0.25897614	0.28249625	0.34314402	0.32820926	0.07014694
0.34793223	0.32555776	0.36749756	0.52098206	0.19964364	0.30136925	0.3027662
0.05788609	-0.2357106	-0.1947573	-0.0588407	0.26200634	-0.0412938	-0.1853512
1.6979486	0.09694424	0.17557842	0.11214694	1.7031563	0.95763139	1.2395699
-0.0463769	-0.1677895	-0.153309	0.02199127	-0.1180682	0.08224176	-0.0712344
0.10512026	0.10820937	0.19471093	0.16735509	0.08578915	0.16919282	0.12243271
-0.2819841	-0.1176997	-0.2182522	-0.2057748	-0.152279	-0.2100931	-0.4346379
-0.0031227	-0.0497329	-0.1096046	-0.0315761	-0.0212043	-0.0785301	-0.0012085
-0.2584522	-0.1528519	-0.0670737	0.15991632	-0.2604172	-0.0644602	-0.2405701
0.82425679	0.33792822	0.29472605	0.2447944	0.9367574	0.32334967	0.54361321
0.44695882	0.36158048	0.35932966	0.33801245	0.57302786	0.42058905	0.14001694
0.97604371	0.88817384	0.59533282	0.87874225	1.5266361	0.91561578	0.92719181

test_GSE101	test_GSE101	test_GSE101	test_GSE101	test_GSE110	test_GSE110	test_GSE110
ref_GSE1015	ref_GSE1015	ref_GSE1015	ref_GSE1015	ref_GSE1108	ref_GSE1108	ref_GSE1108
GSE10159	GSE10159	GSE10159	GSE10159	GSE11087	GSE11087	GSE11087
GEO	GEO	GEO	GEO	GEO	GEO	GEO
affymetrix	affymetrix	affymetrix	affymetrix	affymetrix	affymetrix	affymetrix
TIME:5	CEFSULODIN	CEFSULODIN	TIME:5,CEFSU	relA_b2784:-	relA_b2784:-	lrp_b0889:-1
1817	1822	1823	1826	1835	1836	1845
0.33254706	0.09959402	0.10315852	0.05104196	1.3174696	0.74944708	0.22399456
0.2779309	-0.2356615	-0.2621426	0.32645493	-2.1439408	-2.7423394	-1.0554894
0.22437935	-0.213467	-0.0034176	0.630834	0.50625473	0.59636209	0.25949412
0.03108202	-0.1030877	-0.073067	0.00284389	-0.4989903	-0.3054268	-0.6072487
0.22435406	-0.0106985	-0.148193	-0.1238548	0.29788199	0.28512404	0.13572142
-0.0616418	0.12790909	0.21589727	0.68138518	-0.2599299	-0.0091624	-0.2225515
0.0461099	-0.088294	0.06680273	0.15079391	-0.7595054	-1.6413395	-1.4692397
0.07062812	-0.1615194	-0.0012794	0.00976894	-0.6052705	-0.5264786	1.311808
0.34332702	0.26924875	0.14338273	0.91886396	0.58803353	0.27436264	0.37022346
0.47480812	-0.0447571	-0.1820299	-0.3057609	-0.3210634	0.243756	0.25061251
0.42524806	-0.0523007	-0.0524197	0.2774532	1.2221019	0.97323707	-0.3155162
0.12306831	-0.0769935	-0.1761313	0.42437514	2.1252396	1.9167424	0.16905167
-0.0110663	-0.2782673	-0.0893048	0.18962342	-0.044875	-0.114387	-0.1210625
0.26395363	0.05646246	0.07254764	-0.2171161	1.7101917	0.80014651	-0.9779417
0.21796696	-0.0919025	0.02377074	0.20951791	NaN	NaN	NaN
0.17792145	-0.0988279	0.10999778	0.29469617	0.03316809	-0.1236364	-0.0842334
0.10673	0.19386	0.080707	0.12541	1.3086436	1.6113178	-0.1266237
0.23712408	-0.3667557	-0.476928	0.27749476	1.9394477	1.3064771	-1.1723325
-0.1144075	0.03340426	0.02593774	-0.0220023	-0.6074529	0.12554677	-0.2636339
0.10836359	0.08025562	-0.0471478	0.11562087	-0.2288046	-0.0864794	0.15732369
0.24374458	0.38138267	0.33741788	-0.0069404	0.00933336	0.6542859	-0.1999824
-0.21893	-0.17358	-0.11557	-0.056327	-0.5180105	-0.5563821	-0.3027421
0.21290811	0.31537575	0.25737115	0.46070891	-0.0111167	-0.236327	0.57579688
0.11112	-0.097182	-0.15754	-0.014898	NaN	NaN	NaN
-0.136526	-0.5701654	-0.606475	-0.3597047	0.60778436	0.26838161	-0.2635277
-0.1404731	-0.0831751	-0.1694524	-0.2566616	0.23517052	0.14498859	0.87563578
0.33535	0.043689	-0.070554	0.34252	-0.2800598	0.70177946	4.1648211
0.0693308	-0.2663198	-0.3335711	0.47472851	-0.8653812	-0.6938629	0.95292262
-0.29484	-0.025692	0.08069	-0.082526	0.43749966	0.36679427	-0.5330886
0.16306067	-0.0684706	-0.1886463	0.24680775	1.8474129	1.0106884	1.5106966
0.30141477	-0.1716335	0.03661513	-0.0711307	1.1202038	1.3156909	-0.2974162
0.11252627	-0.5021895	-0.3694327	0.26268175	-0.0312157	-0.3234154	1.0867335

0.10265093	-0.057717	-0.2286209	-0.1940171	0.60038828	0.40619503	1.2478368
0.22507624	0.05270361	-0.0359575	0.17610465	0.10668382	0.12716808	0.48921646
-0.10606	-0.1236011	-0.2283713	-0.117302	-0.1274946	0.46779394	0.66542991
0.06810236	-0.078444	-0.1152726	0.007171	0.22501264	0.13064491	0.31755146
-0.0354668	-0.093768	-0.2235787	0.23397199	0.72203275	0.45589731	0.67508296
0.01457565	0.00255945	0.0614461	0.62526227	1.3068957	0.53632321	2.3266843
0.09480894	-0.61504	-0.4434182	0.65179284	-0.2197706	-0.6583462	-0.0562623
-0.0891069	0.00249482	-0.005034	0.02183026	-0.2707152	-0.1915696	0.06372552
-0.0109405	-0.1068342	-0.1480023	-0.1612828	0.11921203	0.27725375	-0.2052132
-0.1857025	-0.1512992	-0.0439062	-0.1392657	0.04004267	0.09078592	0.11940944
0.33188823	0.12463027	0.00189729	-0.0840334	0.97701523	0.75778621	1.0952063
-0.3675877	-0.4362018	-0.4462033	0.80046415	-1.9653137	-0.2435796	-0.5633007
-0.0625586	-0.0285372	-0.0133067	-0.0270847	0.22344376	0.19321563	-0.0281538
-0.0392494	0.24076549	0.19551468	-0.4612177	-1.5961552	-1.9809434	-0.716065
0.08118816	-0.2064503	-0.1236428	0.18761574	0.08710565	-0.0923829	0.94596826
-0.045248	0.0999856	0.12245893	0.05001791	1.2175218	0.96194003	-0.5611932
-0.1139774	0.03520753	0.11675114	-0.3442108	-0.0348804	-0.1653106	-0.3069703
-0.0425412	0.01454755	-0.1688272	-0.1769633	0.52117474	0.3459705	0.38460636
-0.256338	0.17319657	0.10411549	-0.0928263	0.32613668	0.46202935	-0.4064756
-0.1316498	-0.0934541	-0.0794635	-0.0884302	0.36202304	0.2355997	0.15426898
-0.0813214	-0.1381499	-0.168656	-0.4588025	0.50099061	-0.1101148	0.04932991
0.15468025	0.02321494	0.15231847	0.47286591	-0.6923054	-0.3185299	0.49148417
-0.0681687	0.15864032	0.04472487	0.15239422	0.89399151	0.39023703	-0.0401759
-0.0041707	-0.0975323	-0.1565123	-0.1500498	-0.0242703	0.1039477	0.36577037
0.11115052	-0.0362842	-0.0330237	0.40804738	0.162188	0.45657697	-0.3044952
0.71376652	-0.4248843	-0.242004	1.6458079	-0.3108743	0.86647301	3.3552381
-0.1623431	-0.1444438	-0.0624515	-0.0960187	-0.5708863	0.08451541	-2.0521587
-0.0254475	-0.1260034	-0.1213532	-0.1771127	0.08552179	0.33519927	0.63477457
0.16593001	0.0388622	0.03414308	0.28825127	1.0854731	0.78286952	2.7915226
0.11544318	-0.0690077	0.02594029	-0.1241823	2.4030974	2.0930286	-0.4043717
0.1103913	-0.0340194	0.17687915	0.5397629	2.5849924	2.2474524	0.62941799
0.21693237	-0.332511	-0.1046129	0.18470884	0.63171496	0.54204355	-0.1722923
0.21319024	-0.1007629	-0.1133989	0.23518053	-0.4154093	-0.5132361	0.6062678
0.49311445	1.1713133	1.1321851	1.4646385	-0.5142713	0.07891175	0.68903899
-0.1207437	0.00986758	-0.0995566	-0.1821145	0.5891288	0.74024797	1.9838997
0.06068128	-0.1048308	-0.190318	-0.0659742	0.36311301	-0.0360582	0.44768171
0.17429846	0.04844596	-0.030748	-0.0731303	-0.4555556	-0.102159	1.1911458
-0.028266	0.020534	-0.0430972	0.07442429	-0.2150758	-0.4758403	0.03687348
-0.274727	-0.2940727	-0.162402	-0.08401	-0.1289776	-0.5380163	1.0119127
-0.31071	0.29947866	0.26560757	0.36130494	0.3958874	1.2863022	-0.082594
0.00414487	-0.037697	0.04091266	-0.1713623	0.74911348	0.75065925	0.10410136
0.23346313	0.0991778	-0.1026549	0.01985292	0.74172156	0.6928348	1.1405659

0.11593941	-0.0448816	-0.0711051	0.16169292	1.0541806	0.771164	1.1565112
-0.3666632	0.07891189	-0.1695827	-0.0679822	0.92344837	0.6048727	0.84833075
0.14687266	-0.0766783	-0.0430319	0.2644574	-0.009339	0.60607102	-0.0223732
-0.2288231	-0.023365	0.03226721	0.19270075	1.3810056	1.0767848	1.0757009
-0.0753574	-0.0493208	-0.1958659	-0.2660634	0.27892586	0.30805326	0.28847222
0.07538115	0.04077598	-0.00199	-0.2949242	1.4267783	1.1232124	1.3284254
0.10015714	-0.034968	0.03197925	0.52983189	-0.1694637	-0.2193724	-0.4963493
-0.0411482	0.24789739	0.18406782	0.23594542	0.29154772	0.45169417	0.27726091
0.15785301	-0.0806972	-0.0165464	0.06473437	0.24793834	0.21852576	-0.0614487
-0.0602017	-0.0376186	-0.0342649	0.15079013	0.57395713	0.42476032	0.15765056
-0.0690865	-0.0956145	-0.1008402	0.29110867	-0.1255183	-0.0480725	0.10541502
0.21456584	-0.1476537	-0.1369311	0.13964407	0.88144536	0.06330517	0.3190284
0.07497314	0.01468437	-0.0627452	0.73252788	-0.1274102	-0.1271072	0.26373662
-0.105665	0.15159599	0.11952965	0.16193957	0.17292796	0.29461431	0.0161568
0.098866	-0.069297	0.2019	1.2056	NaN	NaN	NaN
-0.3948274	-0.4291346	-0.1812247	-0.123006	1.1496731	0.65863832	0.08299026
-0.1482441	-0.1608746	-0.2142006	0.21111676	0.26579524	0.70748458	1.07709
-0.0342182	0.07420094	0.15196408	0.13784533	0.8765478	0.31317538	-0.2399915
0.36674	-0.16233	0.02298	0.10608	2.0304177	0.79339414	1.5361396
-0.0122601	-0.0093202	-0.1159612	0.09041219	1.0942759	1.0452299	2.0162425
-0.0606752	-0.0143356	-0.1404808	0.11944756	0.43036262	0.02897837	0.21359959
-0.48545	-0.38543	-0.38954	-0.0001073	NaN	NaN	NaN
0.24718315	-0.507109	-0.5126712	-0.183024	0.95276523	0.73892202	0.47700134
0.01347871	0.04642601	-0.1492222	0.42609461	0.26319799	0.71621239	0.62571733
0.20947447	0.16828849	0.11829992	0.08616536	0.16931769	0.19764615	-0.1478967
0.18003258	-0.1841467	-0.0869193	-0.0828385	0.45040463	0.37496733	-0.8357255
0.34175707	-0.1614263	-0.1490682	0.4954652	0.65992345	-0.0856856	-1.4044685
-0.2270491	-0.1976938	-0.0882736	0.26947682	0.83992502	0.84822596	-0.1533334
0.14304	0.029983	-0.168	-0.05681	1.0272951	0.94787123	0.44730636
0.10473	-0.10342	0.27026	0.38327	1.9274812	2.0575166	1.5694362
-0.113	-0.0022933	0.22352	0.35256	2.9647525	2.6382047	-0.4234227
0.17974	-0.10697	-0.24433	0.59591	2.8331912	2.9782694	2.9518305
0.2547	-0.20098	-0.2397	0.33578	2.6916395	2.453233	2.8965949
0.22012	0.2753	0.15526	0.44548	0.91993512	0.71122617	-0.1273266
0.24774	0.010692	-0.14387	0.43983	-0.3811908	0.29625089	0.90931988
0.61057948	-0.0375422	0.27887546	1.0482595	0.22297604	-0.5545671	-0.3608897
0.10125044	0.23092868	0.093048	-0.3148453	0.90167504	0.17221194	-0.0643767
0.11126628	-0.1238845	-0.0912387	-0.0397343	-2.028618	-1.2536742	-0.6738592
0.02079874	-0.2666449	-0.2220919	1.0851849	0.92436533	1.0666683	0.79678399
0.42439437	0.0856848	0.31635014	0.28328328	0.93462032	0.96877004	0.83070769
0.27757434	-0.2631338	-0.2709847	0.23646702	1.4184317	1.2898069	0.33613645
0.06176014	-0.2835817	-0.4522324	0.02457778	1.2700147	1.2668143	2.3506078

0.26857655	0.35714666	0.19174317	-0.200006	0.01305182	-0.1636683	-0.1814415
0.02030087	-0.1070416	-0.2566258	-0.2592182	1.7518517	2.3631242	-1.1155699
0.01993235	-0.0301367	-0.088506	0.00573233	-0.039518	-0.25653	-0.108281
-0.0547916	-0.0313873	-0.1740823	-0.0831491	0.03240674	0.04408672	0.32629367
-0.3764081	-0.3617738	-0.5400513	-0.2705515	-1.8776927	-1.3738009	0.05361008
-0.0591277	-0.0828867	-0.2241201	0.29955624	0.3580289	0.31731565	1.2329358
-0.0615673	-0.2827212	-0.1344869	0.18519664	0.3902846	0.0025791	0.11693783
0.66853069	-0.1200792	-0.2298272	0.58941885	1.8191924	1.1487885	-0.1839787
0.06363636	-0.1136608	-0.0071648	0.09834058	0.50895503	-0.0818139	0.57692934
0.23709659	-0.0921238	-0.1704487	0.6465658	-0.5021692	-0.0551331	0.34391232
0.05161165	0.02562758	-0.0172713	0.50941391	NaN	NaN	NaN
0.22819201	-0.2330989	-0.219723	0.63851401	0.64742973	0.54646987	0.63391119
0.48831077	0.17660162	0.41416558	1.6063707	-0.8124513	-0.2082716	-0.5038792
0.27728361	-0.0603579	0.18776261	0.72449621	-2.23372	-2.0214083	-1.8770441
0.29034597	-0.1797995	0.08875315	0.39477809	-2.6048054	-2.5073968	-1.9831834
-0.0653408	-0.4549012	-0.1879498	0.14326324	-0.4132986	-0.7166078	0.98053729
0.11960233	-0.1448425	-0.2811216	0.10558525	1.4240326	0.87178506	1.3158538
0.16028608	-0.0692541	0.14760564	0.56222829	0.50147063	-0.5732843	-0.6437119
-0.0638209	-0.4393435	-0.5727468	-0.011171	1.3517751	0.60235264	0.51388708
0.10152482	0.14473098	0.02332729	-0.1399063	0.15246807	0.44727331	1.6708954
-0.1027588	0.18282987	0.43188499	-0.069309	0.2187059	0.01133149	0.2058691
0.10995496	0.00381661	0.12208973	0.28068858	2.2925109	1.8622167	1.8839554
-0.048598	-0.10878	-0.047945	-0.071629	NaN	NaN	NaN
0.42351969	0.00993559	-0.0861712	-0.0167165	1.5188221	1.4073027	2.4520802
-0.22563	0.11745	0.093457	-0.23422	-0.4807343	-0.1650197	0.57524287
0.26666611	-0.3112091	-0.2150456	0.27472532	0.04017034	-0.4614406	-0.0397642
0.03907545	-0.1263741	-0.0923769	-0.1080915	1.18291	0.27007473	0.21256274
0.20206285	-0.1706207	-0.3315436	-0.226589	1.2348671	0.35366189	1.1972848
0.19239071	-0.2380787	0.23150933	1.4275083	-0.7176648	-0.5065046	-0.0993909
0.084127	-0.097982	-0.2431	-0.11835	0.13071765	0.12548862	0.65959385
0.15221233	0.23533108	0.34989049	1.6470422	0.62964956	0.17823687	-0.1297697
-0.3301405	-0.0198709	-0.1033909	-0.2811621	-0.7573858	-0.5365828	0.48733486
-0.4020391	-0.1316185	0.02773231	-0.0899636	-0.7796886	-0.6344876	-1.1529587
0.17726688	0.12674606	-0.1674873	0.23069532	0.05271862	-0.0208966	-0.0763388
0.57777231	0.00856817	-0.0224569	0.18489065	1.818992	1.8913379	1.5933123
-0.172967	0.05452396	0.02793851	-0.0578712	0.61390524	0.71964501	-0.0605777
-0.0441229	-0.1635217	-0.0967447	-0.0729738	0.0764581	0.53235243	0.34038764
-0.0256681	0.07451046	0.02923737	0.22002687	-0.7874282	-0.5793692	-0.2127808
-0.1178078	-0.159163	-0.0472255	-0.1095499	0.09046027	-0.0291339	0.03601717
0.09404545	0.03861524	0.0586701	0.34357013	1.4231897	0.96010037	-0.0529757
-0.3232099	-0.1736374	-0.1053512	0.29356052	-0.338071	-0.2472966	-0.0754917
-0.2224905	-0.2242023	-0.2434339	-0.0758232	-0.1842665	-1.3381713	0.01678081

-0.4228958	-0.2096978	-0.2815071	-0.366155	-0.0870588	0.11556143	0.05014886
0.00443015	-0.1870285	-0.2320918	0.31927481	1.1843731	0.9983126	0.20161973
0.06736972	-0.1069857	-0.1952328	-0.2404922	0.58102891	0.43784534	0.34027612
-0.0443483	-0.1228199	0.15934527	0.57754704	0.51333822	0.23422421	-0.5889153
0.28476265	0.09448601	0.01236406	-0.2048807	-0.4469883	-0.301013	-0.2332163
0.07906815	0.07988988	0.06443577	0.10349042	-0.0131016	-0.1095451	0.04806347
-0.0076395	0.20269112	0.39944219	-0.1567329	0.63206373	0.29231964	0.54102672
-0.1460871	-0.1236618	0.07680276	-0.2678321	1.1830436	1.5087984	1.6139958
0.01798085	-0.2654343	-0.030933	0.87879641	-0.7556829	-0.2797987	0.13216291
-0.3950414	0.16274711	-0.1714295	-0.3184885	0.09512884	0.27491155	0.36548374
0.03788482	0.18795111	0.08549563	-0.5110285	0.21164447	-0.1451291	0.13757419
0.17017825	0.02557728	-0.1203125	0.10812449	0.96221629	0.28271131	0.70761818
-0.2030279	-0.0301877	-0.1040587	0.01438259	-0.1256537	0.22106645	-0.2477386
-0.1379371	-0.1920729	-0.2092305	0.02894982	-0.0768339	-0.2394923	0.13115529
-0.0642341	-0.2288222	-0.0979296	0.00077603	-0.1377103	0.17596558	0.27555284
-0.0533956	-0.1023838	0.14705899	0.20348365	NaN	NaN	NaN
0.3739148	-0.1793517	-0.1260883	0.09636047	0.13347177	-0.3728102	-1.0783542
0.04460987	-0.0985818	-0.1064029	0.02571419	-0.8335397	-0.6967384	-1.4748911
0.0314108	0.20957548	0.14116012	-0.0892016	-0.0046146	-0.2046509	-1.2041669
-0.1307573	-0.1043545	-0.1287852	0.75624983	-0.3292976	-0.4807806	-0.1490923
-0.1929207	-0.1267216	-0.0284473	0.80302334	-0.5826597	-0.3048055	-0.3324634
0.33811081	0.14156282	-0.0929697	-0.1220929	0.73642704	0.79302025	-0.3082866
0.26411112	0.25908403	0.20983237	0.14798658	-0.4966266	-0.2993476	0.41437775
0.07352688	-0.0429775	-0.1255893	-0.1259379	0.17627341	-0.0989778	0.12851449
0.11373957	0.16334489	-0.0107144	-0.271311	-0.6390723	-0.5355392	0.07165589
0.30495166	0.03654657	0.00075814	0.31437628	0.35170074	0.29021867	0.36245864
0.22290781	-0.2466256	-0.1442809	0.07496221	1.3951216	1.3241007	1.7411228
0.39892042	-0.2715741	-0.1878188	1.0371843	-0.4081135	0.00704141	0.56055402
0.32119393	-0.0251454	0.07443232	0.19986754	0.73978854	-0.0340945	0.8765107
0.03581268	0.240708	0.12442639	0.12891282	0.56441768	0.78505796	0.27067352
-0.2277813	-0.1463492	0.02779528	-0.0627605	0.16392716	0.13262054	-0.3526512
-0.066421	-0.4221991	-0.1451164	0.20143865	0.51724767	0.32259124	0.13768581
-0.0996855	-0.5046979	-0.2273063	0.3277516	-0.6277399	-0.501327	-0.203671
0.11087275	-0.0904158	0.02965661	0.05952918	-0.1390266	-0.2322609	0.0567476
0.20105036	0.02475867	0.09701945	0.46735198	0.19679561	0.32811254	0.30621852
-0.0107248	-0.2013319	-0.1589136	-0.0045133	-0.9694044	-0.5785818	1.0016365
-0.2115	-0.16761	-0.046599	0.10518	0.79322778	-0.1577989	-0.3679732
0.25565136	0.08179169	0.04793169	0.16953906	-0.2930067	-0.4048803	-0.3085844
0.35754475	0.19227519	0.16986903	0.59811733	0.76606424	-0.0084647	0.55442247
0.2704	-0.0001465	-0.15024	-0.23307	-0.0776603	-0.0570416	0.26671661
0.26279005	-0.1327919	-0.2659916	0.03607636	3.920373	4.0523754	2.327945
0.1882278	-0.3673753	-0.3491807	0.06085715	3.7366943	3.5613971	3.3021022

-0.0331807	-0.0429937	-0.1097834	-0.2907934	NaN	NaN	NaN
0.03265023	0.05342458	0.02981869	-0.0705082	0.19848368	-0.0306836	0.05935409
0.32335303	-0.3456454	-0.1232978	0.01988809	0.68474878	0.29558247	0.20883193
0.01847046	0.1123579	2.46E-05	-0.3791657	-0.1442937	-0.2590051	-0.2930118
0.03554645	-0.3413898	-0.4045551	0.18491967	3.7350035	3.3770553	-0.2222397
0.1213839	-0.2025764	-0.1298677	-0.0422566	0.6482376	0.30018435	1.143181
-0.0148148	0.046078	-0.1241278	0.06392918	-0.0330781	0.07964173	0.47626219
0.18331732	0.08070082	-0.0227932	0.78484213	-0.0939692	-0.067945	0.18796365
0.145691	0.27342554	0.17626841	0.088287	0.1068494	-0.0423519	0.18637558
0.46689	-0.30983	-0.1875	0.30951	0.32667897	0.67042159	0.6856892
-0.0839664	-0.0955236	-0.0759714	0.22928818	0.83674361	0.3041838	0.41352277
-0.0217658	-0.1205728	-0.0857837	-0.1318034	-0.6978888	-0.1620179	-0.0658612
0.45975735	-0.1606332	-0.0841991	0.4364421	0.59729557	0.90861928	-0.2987967
-0.0628015	-0.0401904	-0.1344273	-0.4104337	NaN	NaN	NaN
-0.1313236	-0.0939585	-0.0758078	0.06717261	-0.3782858	-0.6879783	-2.1222525
0.16235797	-0.0052428	-0.3481476	-0.1358524	0.26074683	0.00073505	0.03872728
-0.1790797	-0.6017195	-0.1241097	0.23212544	NaN	NaN	NaN
0.142123	0.04402701	-0.0064966	0.18106369	-0.5521326	0.16994084	2.1342733
0.15241384	0.20237084	0.10945005	0.01160571	0.60078066	0.58024234	0.44821428
-0.1108826	0.0251445	-0.0550128	-0.4348254	-0.4109643	-0.0750661	0.53495136
0.69515081	0.23693342	0.45126808	1.5483465	-0.8826888	-1.2418412	-0.2669771
-0.2602036	0.05293119	-0.1135669	-0.039757	-0.1634941	-0.0498119	-0.4369903
-0.318971	0.13859152	0.08809459	-0.2552297	1.1297849	0.60047846	-0.4106739
0.24577179	0.10313009	2.28E-05	0.16535395	-0.4322699	-0.3838077	0.63026408
0.29536161	0.35116819	0.24014657	-0.1360748	-1.3654032	-1.0699554	0.12778993
-0.0347364	0.11639238	0.08250159	-0.5374713	0.12560516	-0.1413051	-0.5256999
0.13411858	0.09003231	0.24862612	0.12338679	0.18677963	-0.0750391	0.19662564
0.12303984	0.11899854	0.22455782	0.12200076	-0.3068809	-0.3816202	1.3828483
0.04053359	-0.3464185	-0.4267684	-0.0375242	-0.3225252	-1.1609044	-0.4602738
0.09490497	-0.1831	-0.5416461	0.00426045	-0.4935326	-0.2002703	1.1159939
0.15701271	-0.0526377	-0.2767039	-0.0666807	0.27269272	0.14321866	-0.3202221
-0.0510042	0.02753505	-0.2877324	-0.5439674	0.16360323	0.19967079	-0.5103465
0.33452803	-0.0291848	-0.201202	0.40978836	-0.6232925	-0.6344819	4.2316275
-0.3836438	0.05236463	0.01949846	0.90066576	1.7087101	2.5102102	-0.0561053
-0.311737	-0.0461473	0.04904469	0.02075321	0.0513553	-0.1826506	0.02018323
-0.1904936	0.01381457	-0.0322222	-0.4250937	-0.2202067	-0.0642573	-0.0772536
0.13576899	-0.0332034	-0.1016196	0.0442733	0.11032565	-0.2324114	-0.0855821
0.11299755	-0.178045	-0.1015475	0.12228845	1.0037939	0.48092617	-0.5314832
-0.0082132	0.45380522	0.53765902	1.629074	-0.752273	-0.7663672	-0.3611358
-0.0635265	0.00384638	-0.0700557	-0.1096954	0.12773967	0.53148546	-0.3298696
0.16794324	-0.1259512	-0.1339594	0.17327108	0.61531127	0.54140442	-0.4703224
-0.0463267	-0.0244156	-0.3223011	0.08089069	-0.0836685	-0.1717723	-0.1418852

-0.0572715	-0.1869398	-0.1384021	-0.0965953	0.0493291	-0.169424	-0.1696897
-0.037102	0.0059333	-0.1518	-0.012348	-0.6856535	-0.3473419	-0.0573864
-0.0645095	-0.1724809	0.11728337	-0.4166809	1.0600641	0.94539317	0.37607441
-0.2162092	-0.068429	-0.0953786	-0.3441644	-0.6892848	-0.5410397	-0.5802861
-0.384924	-0.193996	0.00368431	-0.0392479	-0.1036277	-0.1790113	-1.1923676
0.23540516	0.4362698	0.3165208	1.0167931	-0.8148739	-0.5915481	-0.3162929
0.13567153	-0.1666059	-0.5006207	-0.4806506	0.02715149	0.19657333	0.0223738
0.14428	-0.051	-0.15876	0.025547	-0.8198174	0.09513972	0.18024576
0.19226863	0.24938015	0.08829802	-0.2733884	0.9255163	0.96922659	-0.2047458
-0.61335	-0.12824	-0.2694	-0.56586	-0.2054965	-0.9861413	-0.1981717
-0.2416457	0.07358845	-0.1021791	-0.5881758	-0.4787439	-1.3965654	-1.6616858
0.33261017	0.07073117	0.17958824	1.145569	-0.3707959	-0.6274632	0.00859349
0.13534776	-0.0427085	0.00885537	0.23807059	0.44892447	0.19490578	0.19842978
0.11625119	0.30459437	0.04855252	-0.2146559	-0.4240777	-0.3994273	-0.120301
0.24175274	0.19009726	-0.0473122	-0.0599743	-0.1649943	-0.1083428	0.17512918
-0.0923004	-0.2758499	-0.1152935	-0.1474244	-0.0123778	-0.2003156	0.0495113
-0.0849448	0.02279259	0.06216061	-0.3347815	-0.4842605	-0.2617578	0.77113812
0.47472581	0.20355254	0.4667475	1.104844	-0.6885225	-1.0070236	-0.6330486
0.08818747	-0.1620746	-0.3637007	-0.0321278	NaN	NaN	NaN
-0.3607461	-0.09949	-0.0675338	-0.5279655	-0.5831127	-0.385202	-0.044569
0.07359113	0.05911969	-0.1271595	-0.2310301	-0.0888232	-0.1789645	0.26878305
0.18595993	-0.017174	-0.0825429	0.19542838	-0.1050641	0.64501575	-0.0476592
-0.1671	0.063636	-0.00436	0.24155	0.58969439	-0.0802271	-0.3842597
0.05791326	-0.1303777	-0.0841219	0.14736148	0.37889205	1.0568398	0.09190528
-0.0703191	-0.1355373	-0.109831	-0.2127611	NaN	NaN	NaN
-0.1006702	-0.0288413	-0.1321221	-0.0403663	1.1683997	0.29035313	-0.1134843
-0.2030292	-0.189134	-0.1098115	0.07391932	1.3616832	2.0488761	0.50424362
-0.12509	-0.13633	0.014564	0.26703	NaN	NaN	NaN
0.22640035	-0.37087	-0.3387803	-0.0441994	-0.7110531	0.11827906	-0.9464483
0.03390474	-0.2605343	-0.4483242	-0.0106088	1.4859866	1.2929505	-0.1280165
0.24059895	0.30366504	0.13517998	0.46034719	-1.034124	-0.5752034	-0.289672
0.0880564	0.16365803	0.09781645	0.01832076	-0.2704184	0.0092432	0.01093554
-0.17145	-0.10419	-0.041073	-0.32586	-2.8323349	-2.4907785	-1.3823376
-0.151411	-0.2868325	-0.2731424	-0.1594805	-0.0801777	-0.0637373	0.33523751
0.45021262	0.22796636	0.31716745	1.0917153	-0.7889795	-0.3287516	-0.1587716
0.25131401	-0.0488577	-0.1275447	0.05767012	-0.9288016	-0.9310273	-1.1586435
-0.1419847	-0.0529197	0.0135158	0.45001425	0.3874481	-0.1786075	-0.0571373
0.05890847	0.18909227	0.03945973	-0.3195136	-1.4796009	-1.1172439	1.5324007
0.24000773	-0.1903465	-0.1619925	0.2149897	2.5802047	2.1461517	0.89667441
-0.0593066	-0.0839701	-0.1138977	-0.2925297	0.38905331	0.03190369	0.75435272
-0.0415928	-0.1892432	-0.4174879	-0.4803178	0.48757581	0.34322036	0.68959048
-0.0436705	0.00175576	-0.0516111	-0.1403226	0.05025342	0.1839093	0.04296127

0.31058635	-0.0176553	0.01632125	0.22672142	0.71506295	0.75341116	1.4646394
-0.1592358	-0.2195605	-0.1276826	-0.0680412	-0.4358771	-0.9594944	0.36584965
-0.1983943	0.01219476	0.02600683	-0.0365718	2.5226901	0.06167923	-0.5699179
0.11481209	-0.0609761	-0.1162173	-0.4295495	0.16813509	0.41330745	0.32117012
0.10515325	-0.0867852	-0.1085571	-0.3576329	-0.4233171	-0.4004374	-0.7177498
0.06382968	-0.181983	-0.22795	0.1547162	0.88533506	0.65690726	0.26944401
0.03520911	-0.0379711	-0.0583988	0.33895203	0.8958306	0.43701017	0.15556686
-0.1026228	-0.1693686	-0.0050588	-0.0025738	-0.2433919	-0.2179281	0.11279657
0.35329689	0.15576752	-0.1543193	0.17467502	0.0486379	0.33361511	-0.1454711
-0.20887	-0.10201	0.039883	0.13649	-0.1224624	-0.3638072	-0.7327189
-0.1735361	-0.1539166	-0.064378	0.07462868	0.04826234	0.49248977	0.00632853
0.01953259	-0.0695591	-0.0326679	0.59053253	0.24783604	-0.0808974	-0.0137417
0.05816376	0.17929273	0.18984596	0.03035667	0.72154987	0.09165676	-0.0645518
-0.13933	0.0081901	-0.075732	-0.081182	0.3536613	0.1620289	-0.1705501
0.16961542	-0.0372736	-0.0689048	0.06899665	0.54079793	0.39422223	0.24096147
-0.0760096	-0.0122848	0.01581825	0.05218605	-0.4343117	-0.2623244	0.33641463
0.37356469	-0.3803518	-0.2231516	0.18561034	0.31897129	0.16819917	0.1232058
0.09734705	-0.1109767	0.03698358	0.24980347	0.03258335	-0.4460123	-0.5737538
0.20368041	-0.0193176	0.08931038	-0.4475235	0.73017579	0.32737103	-0.1180711
0.21888046	-0.2892279	-0.4279758	0.68437435	0.3658581	0.6395531	1.320008

test_GSE110	test_GSE110	test_GSE110	test_GSE135	test_GSE135	test_GSE135	test_GSE135
ref_GSE1108	ref_GSE1108	ref_GSE1108	ref_GSE1358	ref_GSE1358	ref_GSE1358	ref_GSE1358
GSE11087	GSE11087	GSE11087	GSE13589	GSE13589	GSE13589	GSE13589
GEO	GEO	GEO	GEO	GEO	GEO	GEO
affymetrix	affymetrix	affymetrix	affymetrix	affymetrix	affymetrix	affymetrix
,ISOLEUCINE:	ISOLEUCINE:	ISOLEUCINE:	BIOFILM_DEI	BIOFILM_DEI	BIOFILM_DEI	BIOFILM:-1,P
1848	1850	1853	1890	1891	1892	1898
0.11005009	-0.0771666	-0.0044097	-0.2797689	-0.3325472	-0.3182262	-0.2941839
0.05944938	-0.7150895	-1.420558	-0.274855	-0.2625086	-0.2474131	-0.9813838
0.03630006	0.3799447	0.40675834	0.20851954	0.23996516	0.25454205	1.5032211
-0.1340726	-0.0860761	-0.2459078	0.25079674	0.24393218	0.26620863	-0.3154006
0.07281566	0.21950046	0.36647874	-0.2169291	-0.1716727	-0.163866	-0.0948043
0.18564319	0.47275191	0.21990139	0.08289288	0.09496896	0.01366964	-0.2466828
0.09483752	-0.8502831	-0.9553219	0.35834776	0.30055333	0.32186444	-0.5611844
0.13658693	0.52699114	0.57731552	-0.1735016	-0.1183084	-0.1947635	1.6797773
0.03596863	-0.0016419	0.06874121	-0.1784417	-0.1542641	-0.1989733	0.12374447
0.05521769	0.40927516	0.66196762	-0.2688771	-0.2786884	-0.3356618	0.26272393
-0.0301137	0.13576601	0.07423796	0.69601587	0.58711088	0.7352614	1.4900669
-0.0560239	-0.025742	0.12544786	0.01919319	-0.0644698	-0.0546447	0.63293183
0.08632785	0.06925731	0.0686691	-0.4682115	-0.4351501	-0.4326859	-0.6464687
0.05920532	-0.2966742	0.35378859	0.53925584	0.63099659	0.54665468	-0.6653792
0.07741018	0.01485876	-0.0146539	-0.1156688	-0.0713787	-0.116345	-0.1693147
0.01355237	0.14664005	0.17748486	-0.311533	-0.288454	-0.2929404	-0.206288
-0.17866	0.034192	0.021668	0.33447	0.44861	0.3821	1.844
-0.0892699	-0.5176721	-0.7631341	0.31892085	0.28150834	0.25369782	-0.3470763
-0.1231561	0.17122311	0.03355222	0.20795657	0.18245836	0.18800786	0.38689793
-0.1284632	-0.056731	0.12468195	-0.082213	-0.0069404	-0.1522225	1.2826189
0.07817882	0.14045542	0.15941272	0.15554356	0.20386534	0.17828076	-0.0569343
0.031721	-0.009069	-0.056505	0.23987	0.040023	0.032243	3.1364
-0.0233222	0.09281439	0.38137971	-0.0940557	-0.0586311	-0.0531845	2.5141772
0.070014	0.035383	0.8132	-0.21969	-0.20364	-0.20155	0.89184
0.02545089	0.29764828	0.12422533	0.39887398	0.37202684	0.43488669	0.59438634
-0.0699438	-0.4427606	-0.4475352	0.26787001	0.31885353	0.30950819	-0.5252925
-0.010716	0.23368	1.1797	-0.027288	-0.061942	-0.0091679	2.3266
-0.2304347	0.26058131	0.44453697	-0.0328402	-0.055523	-0.0741486	-2.163115
0.13932	-0.1258	-0.50465	0.14042	0.13686	0.15977	0.90957
-0.0020157	0.27284399	0.09472591	0.39025851	0.36963197	0.44780795	1.0409412
-0.028653	-0.0140091	-0.2411286	0.05922348	0.1112324	0.08138313	-0.4304095
0.12423741	0.36823429	0.67696859	0.43512733	0.39490382	0.41353876	0.8781783

0.06053105	0.34439888	0.44855895	-0.2808502	-0.2146943	-0.3111858	-1.1906566
-0.1063233	-0.1869683	0.04982855	-0.1459516	-0.1473658	-0.1313748	-0.1453716
-0.0290112	0.77526953	0.70047987	0.19377512	0.13782112	0.16487642	0.21033079
0.10165798	-0.1097241	-0.0627279	-0.2530363	-0.3068485	-0.2832664	-0.2047049
0.02171316	0.15539255	0.09162933	0.01130938	-0.0214604	-0.0844734	0.90484789
-0.0809639	0.25713197	0.32801922	0.87890475	0.85270566	0.91377678	1.7361803
0.1270208	0.4194793	0.98639185	0.54148325	0.56599845	0.57993693	0.16334525
0.03069856	0.37766188	0.36179558	0.18069973	0.15628556	0.17590617	0.41521654
0.08740616	0.05525707	-0.054246	-0.4517231	-0.359878	-0.3341242	-0.721624
0.20405536	0.12801695	0.13780362	0.03539107	-0.0540952	-0.0942407	-0.2430873
0.12844343	0.14695542	0.32470805	-0.2404271	-0.2108212	-0.2196025	0.05369704
-0.1350036	0.21121264	-0.0206576	0.50713688	0.43358469	0.42743116	1.2820733
-0.1392485	-0.1623052	-0.1544888	0.00677791	-0.0107333	-0.0205057	-0.1255802
-0.0351149	0.51319955	0.67636386	-0.155738	-0.1363085	-0.1467266	-0.3513119
-0.3315806	0.57900646	-0.0121293	0.0191743	0.10893369	0.11404549	0.24934737
-0.0165249	-0.3770477	-0.7573925	0.31272521	0.28884426	0.30865185	-0.6484581
0.14299754	0.0822296	0.18356593	0.18931477	0.20427494	0.20532705	1.5498282
0.00112744	0.25109755	0.08389196	0.4697558	0.49465461	0.50068271	-0.1418096
0.09604872	0.13539832	0.20221029	0.81513217	0.81051947	0.89491044	0.07997934
0.01093394	0.00134831	-0.0865324	-0.1647603	-0.2024701	-0.1918862	-0.4534954
-0.0604656	0.28847168	0.32966678	-0.4568324	-0.4072626	-0.4421939	-0.038205
0.16336584	-0.0585532	0.37024011	0.19986849	0.24896659	0.26253547	2.5569988
0.13911318	-0.2392411	-0.0847667	0.34381624	0.34092571	0.33245014	1.4512981
0.13978945	-0.0020029	-0.0307154	0.45845288	0.44965544	0.42787847	0.20060817
0.12707548	0.14894981	0.00857367	0.33038441	0.39212464	0.36660752	1.00017
0.20848782	0.92212414	1.6762404	-0.0415075	-0.1776144	-0.1905822	2.1525959
-0.0550987	0.56910789	0.71009837	-0.4725786	-0.4275783	-0.4267445	-1.9977121
0.02683116	0.72584385	0.9679012	0.0012222	0.03680129	0.03003409	-0.3052293
-0.0609246	0.15127179	0.15554666	0.03953777	0.02310404	-0.0047683	-0.1734733
0.29614697	0.39283942	-0.2281363	-0.0663988	0.01639229	-0.0023169	-0.5441601
0.33625862	1.1533732	0.67598191	1.2255613	1.2573228	1.1940441	-0.0624956
-0.0903262	0.05024422	0.0340066	0.13384038	0.08454538	0.0789028	-0.1101621
0.15884672	0.24547662	0.72622683	0.29903735	0.29257724	0.31983693	1.056495
-0.1200454	0.93483525	1.4321926	0.16797179	0.08604669	0.11106048	1.5134928
-0.1282536	0.6837393	1.1942437	0.43443545	0.44841841	0.44166027	1.3192089
0.09795926	0.09969712	0.09850715	0.24627726	0.2412452	0.29248151	0.5021667
0.10562561	0.22429873	0.55854928	-0.4937532	-0.423568	-0.4498345	-0.5141922
-0.146893	-0.2194827	-0.2206236	-0.0132663	-0.0294198	-0.0608108	0.04324305
0.14911712	0.48757278	0.61260023	0.37106137	0.33512045	0.32283354	0.27686227
0.09382246	0.13489714	0.00037646	0.13514378	0.06885212	0.11191379	-0.1096824
-0.0234793	0.3645325	0.21389285	0.13630032	0.17663482	0.1957952	-0.0167269
0.01238337	0.1669182	0.38752661	-0.2303796	-0.1736589	-0.1820458	-0.1609492

0.09541174	0.52121033	0.76701438	-0.3984199	-0.3995157	-0.3877482	-0.8257407
-0.0815065	0.37522549	0.12799832	1.3830428	1.3325867	1.3504626	0.03166039
0.18525223	0.69610937	0.85182916	0.84758445	0.92554716	0.87871963	-0.0750295
0.0033844	0.57935457	0.48025078	0.19024353	0.10540293	0.17968474	1.2717034
-0.0965146	0.47367583	0.71753848	0.20216049	0.2000615	0.22383941	0.14105719
-0.1412075	0.30278375	0.29370854	1.346807	1.4183709	1.3918025	1.0232001
0.01334478	-0.1036618	-0.214405	0.06125124	-0.0136377	0.0399459	-0.1262057
-0.0560981	-0.3184948	-0.1647796	0.65172907	0.50606901	0.59137624	1.8661253
-0.0538794	0.18143719	0.10199774	0.31620839	0.31805471	0.29138302	0.54411247
0.04019376	0.25070165	0.07132425	0.5375031	0.48759459	0.43450738	0.24082319
0.03229312	0.35573308	0.14555502	0.11379372	0.11028804	0.1030835	0.43883907
0.07553692	0.26709489	0.29058089	0.1588233	0.12813197	0.17704568	0.37238145
0.01057407	0.0148073	0.00362256	-0.2917939	-0.1349199	-0.1918712	0.74409904
-0.0739926	0.20069537	0.31178706	0.38404505	0.43591742	0.50950349	1.0829967
0.015127	0.31583	0.67228	-0.046049	0.081329	-0.046809	0.64296
-0.0945699	-0.0867956	-0.1920975	0.64800307	0.68225673	0.66088088	-0.3165769
-0.067648	-0.0183006	-0.030532	0.78851408	0.77876945	0.77237414	1.7886339
-0.2303051	-0.1747746	-0.2542825	0.34525681	0.31784279	0.28514477	0.47806254
-0.025926	0.12871	0.62285	0.090173	0.086147	0.016031	0.70469
-0.0084432	0.10474912	-0.008893	0.21429235	0.27347456	0.23308292	0.53313213
0.07660201	0.17527534	0.49470277	-0.1338399	-0.1586584	-0.2124693	0.29898404
-0.069744	0.099144	0.028353	0.25979	0.21866	0.30132	0.7463
-0.1276829	0.07216732	-0.1627497	0.15256513	0.09637659	0.20743294	1.0484782
-0.0910454	0.32239177	0.55836447	0.08489322	0.11527024	0.0939222	0.72133226
0.30862328	0.316019	0.45612556	0.6898942	0.66738438	0.65721345	0.37180988
-0.1265021	-0.6222389	-0.835249	1.0578403	1.0829851	1.1408735	0.52674983
-0.0432836	-0.6517523	-1.0749014	0.91019773	0.88959381	0.91622143	-0.010366
0.04807382	0.02759139	-0.0112159	0.5558745	0.49266729	0.5311388	-0.6575352
NaN	NaN	NaN	0.53096	0.46094	0.45507	-0.23747
NaN	NaN	NaN	1.4933	1.4628	1.4411	0.28099
NaN	NaN	NaN	0.43852	0.39191	0.45178	-0.44204
NaN	NaN	NaN	0.47518	0.46662	0.43133	1.7203
NaN	NaN	NaN	0.44891	0.46492	0.51946	1.537
NaN	NaN	NaN	-0.2424	-0.089374	-0.17638	0.67029
NaN	NaN	NaN	0.55117	0.48211	0.49804	0.29817
0.0584033	-0.0736567	0.15386626	0.37863156	0.40138465	0.39207794	0.89948654
-0.2256093	0.08100852	-0.0488446	-0.0155724	-0.0200599	-0.016761	0.2285075
0.08126061	0.5744011	0.71725493	-0.2910518	-0.2865113	-0.2764636	-1.2154134
0.05115115	0.11385358	0.21842583	0.21478741	0.20871051	0.2145785	0.57825614
-0.0078638	0.23230091	0.42531446	-0.0951253	-0.0939973	-0.0816753	0.1862622
-0.2384371	-0.0789292	0.01534596	-0.2124346	-0.2935963	-0.1327235	-1.138953
-0.0015427	0.42520909	0.73268743	-0.1700735	-0.167601	-0.1265329	-0.0315803

-0.3473464	-0.0820426	-0.2073234	1.1454818	1.1999793	1.2767435	0.84419178
-0.2641242	0.20791542	0.02459676	0.39930763	0.41407778	0.36947109	-1.1101999
0.09325393	0.32731548	0.2631157	0.15680691	0.2821468	0.27047228	0.90862103
-0.1447565	0.28534245	0.33039356	0.02737242	0.0365326	0.01297106	0.14341883
0.01482875	0.14086545	0.61354807	-0.2721596	-0.2655877	-0.1707368	0.25500039
-0.0581405	0.39871112	0.64541397	-0.2469612	-0.2895352	-0.2313878	0.54688225
-0.0027326	0.02014962	0.23747987	0.17465669	0.2448341	0.22680159	0.28399993
-0.0978805	-0.2158572	-0.1304536	0.25740397	0.26933362	0.29523157	0.77744889
-0.0297466	-0.015247	0.07289386	0.36198928	0.35751783	0.38825576	0.3659864
-0.0648693	0.45206293	0.56081142	0.13827571	0.16227334	0.21706549	1.2939245
-0.0118905	0.41129273	0.25350301	0.32622869	0.33061062	0.27838982	0.68807652
-0.0519837	-0.1444431	-0.0096943	0.23438856	0.20572483	0.12904705	0.68575493
0.03317655	-0.1458297	-0.3620377	0.05472831	0.01584858	0.01138981	0.39040345
0.18676845	-0.0156512	-0.5842466	0.12599495	0.10489073	0.12329686	0.86382636
0.12177997	0.05612151	-0.5680892	0.26643748	0.19890574	0.17249386	0.37163145
0.01087959	0.49507624	0.31939408	0.57406645	0.55828936	0.59064422	-0.8493853
0.14127832	0.25977683	0.42971211	0.37389091	0.34457053	0.3555419	1.2741935
-0.1644319	-0.429078	-0.7703438	-0.0116261	-0.2374122	-0.062663	0.05844121
-0.039048	0.23636598	0.55082633	0.55449954	0.54264993	0.58006878	0.10867996
-0.0652094	-0.0930861	-0.0833809	1.5468544	1.4181814	1.4353249	-0.7424586
-0.1745066	-0.3068299	-0.5296657	1.0011802	0.9488386	1.0510509	-0.2064914
-0.0826005	-0.2945906	-0.0432055	-0.2222928	-0.2933963	-0.2388966	1.279499
-0.030968	0.14306	0.052575	1.1488	0.96065	1.0348	1.1721
0.07872644	0.17849036	0.46654042	-0.2507661	-0.1824755	-0.1272558	-0.2143563
-0.038401	0.3651	0.71033	0.32741	0.32516	0.29771	0.75316
-0.1062718	0.00039784	-0.0299579	0.09215967	0.05746187	0.08607826	0.4795399
0.08732756	0.07669895	-0.0669048	-0.0896226	-0.0154495	-0.0432297	-0.4135749
-0.1096142	0.34762052	0.4113201	-0.1620876	-0.1608858	-0.1669314	1.1879492
0.26144012	-0.1765957	-0.2683976	0.49792019	0.50707651	0.48761952	0.42781358
-0.097033	0.10531	0.44436	-0.10806	-0.12124	-0.056345	1.3204
-0.0043557	-0.2181058	-0.2104065	-0.1790243	-0.2800858	-0.2231896	0.3113497
0.06436396	-0.0786819	0.10124298	0.21682936	0.18404874	0.19496088	0.45999904
0.02977565	-0.1419841	-0.2718097	0.12205935	0.11866815	0.11158852	-0.4044248
-0.0094238	0.04244431	0.1315368	-0.1164278	-0.1630346	-0.1642612	-0.095218
-0.0642904	0.37600173	0.65140023	0.07424413	0.07749459	0.07369973	0.01125658
-0.0505469	0.09480753	-0.0869083	0.23599889	0.26312668	0.20344678	1.0816726
0.05716886	0.29076272	0.35101715	0.06355585	0.06362491	0.09033283	0.17858825
0.1462939	0.26981132	-0.0016412	0.23122511	0.24217184	0.22625761	0.63412927
-0.0494631	-0.1425894	0.03570364	-0.0697295	-0.1242052	-0.1304512	1.4234357
-0.1335298	0.24962308	0.06965257	0.49053927	0.4958927	0.44768607	0.82944962
0.03263502	-0.1022975	-0.2766485	0.26293502	0.19467586	0.25575101	0.63891494
-0.0789539	-0.069406	-0.3677473	0.55795947	0.61717515	0.54625757	-0.763142

-0.0417594	0.04874362	-0.0092851	0.16389707	0.18682192	0.21968064	0.55376389
0.13043744	-0.1036178	-0.0249212	0.18089655	0.16053482	0.12947927	0.38472319
-0.0230857	0.06824149	-0.0171275	0.24978282	0.30169443	0.27369424	0.36117645
-0.0223601	0.40543206	0.44385448	-0.1259945	-0.1784041	-0.1080435	-0.0349051
0.07838465	0.06196827	-0.0010902	-0.3652444	-0.3642916	-0.3728276	0.16155227
0.10058293	0.11755865	0.13899858	-0.0756889	-0.0421879	-0.0790161	-0.0198402
-0.1998033	-0.2563143	-0.2724774	0.43162655	0.4641311	0.42794617	0.54022875
-0.1833539	-0.0893959	-0.2747969	0.83525433	0.85788953	0.84759535	0.31364571
-0.0791585	0.25102259	0.45969604	-0.1834573	-0.0772498	0.06542041	1.3625376
0.0829336	0.22280113	0.29666646	0.3272081	0.32294908	0.48565979	0.79256593
-0.0486419	0.14170903	0.12740403	0.11829321	0.12005496	0.10294677	0.44481386
0.1575423	-0.0516265	0.15808694	-0.1427585	-0.1388735	-0.1255449	-0.2257232
0.09779525	0.08867333	0.01991617	0.56630675	0.59438074	0.62064212	0.58036349
0.25098422	-0.0062043	0.10546574	-0.2089333	-0.2594373	-0.211145	-0.5853222
-0.0656361	0.13534651	0.28588671	0.12446145	0.20480304	0.18681381	0.51420484
0.02569412	0.06588879	0.0942063	0.57841712	0.52059373	0.58691734	0.36575853
-0.1165248	-0.231736	-0.7040781	0.44827568	0.45424079	0.50069432	0.04379043
0.01592215	-0.0896915	-0.0995811	-0.1501954	-0.1231614	-0.1208168	-1.2201208
0.03522943	-0.1504871	-0.08609	0.36551209	0.27541848	0.24346172	-0.2496237
-0.0239375	0.06108977	-0.0150993	0.1665472	0.17599717	0.27692963	0.31395051
-0.0353084	-0.0461128	-0.0400638	-0.1455452	-0.1822545	-0.1841357	0.3210596
0.07345767	0.22122051	0.05430242	-0.2056844	-0.1705608	-0.1776834	0.21394106
0.1401717	0.40135791	0.43392951	-0.0488396	-0.031259	-0.0136708	-0.0353023
0.11036891	-0.1432666	-0.1151723	-0.2836098	-0.2935701	-0.265003	-3.1564429
-0.0772856	0.01412256	-0.177458	-0.1161382	-0.0866292	-0.0939804	0.33617479
-0.0073641	-0.0717631	0.26868576	-0.0872857	-0.1502516	-0.121315	0.30478158
0.11217848	0.25366872	0.45717777	0.14766497	0.12754252	0.15484145	0.19762568
-0.0170827	0.51640682	0.70826442	-0.0729484	-0.0477517	-0.0523424	-0.0910973
0.07431218	0.05751208	0.14046642	0.31137858	0.29482892	0.2929641	0.26253583
-0.0999385	0.2295467	0.39354227	0.10360635	0.14176846	0.10089151	0.0696071
0.19409031	0.1516879	-0.0998485	-0.0901313	-0.0899468	-0.050519	-0.0167857
-0.0444037	-0.00387	-0.1061681	-0.1378197	-0.2132915	-0.1340581	-0.2362565
0.38481122	-0.0420159	-0.2866076	1.0519611	1.0874906	1.1611506	0.41381069
0.07526543	-0.0994583	-0.0375937	0.0778023	-0.0191166	-0.0061326	-0.2716953
0.0173653	0.26976773	0.37007441	-0.7142096	-0.7079461	-0.7335704	0.7503752
0.15898101	0.64903333	0.93622045	0.25117194	0.25398474	0.26909355	-0.7921096
0.22948	-0.38109	-0.47853	0.21436	0.17899	0.1471	0.47345
-0.0319766	-0.139157	-0.2206499	0.26804359	0.24354378	0.23468173	-0.006068
0.02823377	0.18631683	0.30140126	0.07693494	0.07070541	0.03351975	1.4102032
-0.070605	0.016765	-0.16303	0.34159	0.34382	0.29574	1.1541
0.16993084	-0.0068283	0.17313811	1.126106	1.1017507	1.0920554	2.1698226
-0.0820136	0.10063183	0.24265744	1.1631144	1.1578155	1.1355046	1.7681546

0.04231629	-0.2650798	-0.2360219	0.24391001	0.21436654	0.22969021	-0.0534844
-0.0512247	0.08608167	0.16989066	0.10247859	0.15195692	0.16329428	0.19387803
-0.0400307	0.05867804	0.08785769	-0.2133941	-0.2316573	-0.2047448	0.61791214
0.13368102	0.3156296	0.22651327	0.08316118	0.0651173	0.02113112	-0.4596291
0.00453861	-0.0811819	-0.1459672	0.32012829	0.33391914	0.26054819	0.07611525
0.0961058	0.00918916	0.15542507	0.16822446	0.13810544	0.11391822	0.09262476
-0.0348311	0.33387344	0.38847026	0.30329023	0.34005904	0.30383998	-0.3077154
0.05443813	-0.221845	-0.1495532	-0.1231973	-0.1525785	-0.1815655	-0.1978995
0.03594237	-0.1521357	-0.263659	-0.0564255	-0.2711229	-0.1328113	-0.20647
0.048748	-0.092077	0.27671	0.17513	0.23327	0.2049	-0.55437
0.16237732	-0.1276359	-0.0375258	0.26307351	0.17507236	0.22633046	0.04709533
0.09215597	-0.2318182	-0.1816299	0.27127368	0.24948766	0.27237084	-0.510079
0.06064279	-0.1446074	-0.2756315	0.12799549	0.06773239	0.0733057	-0.1466509
0.20231092	0.37950255	0.66603644	0.02622055	-0.062142	-0.0321284	-0.031241
-0.1023538	-0.6148646	-1.7679298	0.31828615	0.23326671	0.19196602	-1.85375
0.01037462	-0.2047581	-0.0657919	-0.3893543	-0.2793144	-0.3331666	-0.5883854
0.24519906	0.26640489	0.55351879	0.42700928	0.40629937	0.42051357	-0.0203067
-0.006568	0.43079289	0.68129154	0.26658513	0.33211939	0.31209299	-1.5844956
-0.0908826	0.11908847	0.18782548	-0.1512493	-0.1914057	-0.1580028	-0.013127
-0.0743551	-0.195277	-0.3069873	-0.0593164	0.00422113	0.01132455	0.63022768
-0.1141752	-0.4479392	-1.022071	0.49217413	0.47743996	0.58333537	0.59638189
0.08582319	0.14802038	0.07883143	-0.0430512	-0.0958372	0.00613838	0.29852427
0.09318466	-0.0942649	-0.0849207	0.01056381	-0.0595634	-0.056077	-0.187098
-0.0235262	-0.1328885	-0.1441363	0.22910245	0.31093164	0.30499524	0.50888288
0.27144446	0.06003378	0.58425355	-0.0330449	-0.0736417	-0.1076125	-0.2950849
-0.0357736	-0.2715417	0.06962676	0.09757678	0.17550686	0.11771301	0.09975816
0.29121255	-0.154541	-0.0951491	-0.3655761	-0.371345	-0.4164701	-0.9235106
0.11664113	0.41745955	0.71545416	-0.472697	-0.4247038	-0.5315047	-1.19429
0.23681716	0.2578797	0.51719837	-1.0120558	-1.0393633	-1.0242109	-2.0753113
-0.0194806	0.00886684	0.42724153	-0.2881192	-0.341216	-0.3532632	-0.3095067
-0.0021328	-0.0379452	0.12266621	-0.2846466	-0.2457851	-0.2666218	-0.2613297
-0.093452	-0.0343863	-0.2488532	-0.1023909	-0.0791067	-0.0700527	-0.3004654
0.13820142	0.53916434	1.6738725	-0.5242308	-0.5867397	-0.6461559	1.3588778
0.47455529	-0.0517785	-0.0415578	1.4824294	1.4115242	1.4099524	0.11168877
0.16898962	0.63176613	0.55624224	0.30170181	0.17553968	0.19006423	-0.0782593
0.05316955	-0.0144305	0.27040229	-0.2780363	-0.2580755	-0.2912879	-0.2884614
0.23145705	-0.0602266	-0.209527	0.31700852	0.27937008	0.24024109	0.55611283
0.0069066	-0.3996622	-0.3704752	-0.3157162	-0.336408	-0.345671	0.69201075
0.15981758	-0.1183452	-0.0808168	-0.32434	-0.3591662	-0.3342667	0.49812865
0.16971135	-0.1458581	-0.4405065	0.10468825	0.14539212	0.06628665	-0.2447207
-0.0385331	0.0456872	-0.0573453	-0.1987773	-0.2222722	-0.2519646	-1.5520554
0.15701359	-0.141017	-0.0142619	0.03943115	0.05249311	-0.0516129	0.22496933

-0.0347077	-0.2475192	-0.0185724	-0.3652197	-0.3460503	-0.347143	-0.2173265
0.098901	0.41026	0.53346	0.23878	0.21321	0.22297	1.692
-0.0347793	-0.2391087	-0.4853129	0.14287604	0.19828441	0.2152057	-0.0014058
0.18849133	-0.1198716	-0.0791514	0.00187783	0.09015492	0.07710219	-0.4357975
0.06436326	-0.246868	-0.1729135	0.58989303	0.48907854	0.45499945	-0.0650632
0.0980367	-0.4076	-0.295157	0.10578404	0.00390117	-0.0054527	0.04940164
0.04667644	0.14477161	0.21792716	-0.2900526	-0.2681112	-0.2466788	-0.8180394
0.21492	0.22254	0.36386	0.254	0.24473	0.27996	0.26249
-0.0301645	-0.384951	-0.3308196	0.10818922	0.10710297	0.14447307	1.1280219
0.042846	0.22007	0.10791	1.2098	1.2659	1.3003	-0.86025
0.02008237	-0.5041361	-1.5143218	0.5146061	0.51560924	0.58056269	0.24959574
-0.1296426	-0.5773457	-0.47456	0.09442275	0.20201171	0.20584267	0.14017471
-0.0264451	-0.2243272	-0.1047448	-0.0106568	0.00801647	0.0198638	0.63748448
-0.0316431	0.06095398	-0.0738595	-0.0781773	-0.1100678	-0.1025687	0.22621118
0.02726764	0.16866914	0.0451143	-0.0783525	0.04631896	0.0846713	0.18315442
0.0203319	0.03979634	0.01376584	-0.2414401	-0.3264441	-0.2506554	-0.2714366
-0.0810138	0.16708721	0.09925728	-0.2426085	-0.1632122	-0.1817002	-0.513265
0.26423367	0.09664477	0.06226412	0.03425017	0.0352292	0.02821368	-0.0470915
-0.00301	-0.3092774	-0.3580817	0.40675099	0.34155429	0.36542339	-0.6415815
0.06003789	0.40827182	0.56768402	0.03328575	0.03811977	0.03333676	-0.4446853
0.03013006	0.19462827	0.34875798	0.51167275	0.47832956	0.49077864	0.08865763
0.03192479	-0.0045633	0.01923757	-0.1474062	-0.1204031	0.02145031	-0.2008057
0.08285	-0.083432	-0.18651	0.4158	0.41912	0.40184	0.23327
0.04138932	0.38179959	0.54714082	-0.1721629	-0.1592188	-0.1648675	-2.2423055
0.12835046	-0.1475235	-0.1447873	0.1213336	0.23503383	0.19013821	0.11682273
0.10605044	-0.2733428	-0.2291306	0.43425394	0.41304682	0.43423112	-0.8591539
0.14376822	-0.1440632	-0.061038	0.80799511	0.70909538	0.71188922	-0.1622213
0.19592	0.070333	0.045069	1.3449	1.3071	1.3734	-0.11191
0.00766899	-0.4247622	-0.3735801	-0.0875065	-0.0781269	-0.0675663	1.3950682
-0.130555	-0.0474775	-0.131978	0.07146053	0.06946092	0.09654493	0.52356331
-0.0414241	0.02905596	0.1684229	0.432776	0.40924597	0.36707216	0.56265847
0.03783736	0.1668308	0.09292429	-0.3068172	-0.3237555	-0.3062956	-0.3929385
0.29407	-0.6943	-0.40104	0.69541	0.54478	0.61892	-0.09468
0.37451216	0.0799448	0.15668557	-0.4597639	-0.5115191	-0.5090653	-1.9981363
0.12550201	-0.0980764	-0.1574357	-0.2760556	-0.3093309	-0.3650093	0.97593642
-0.1162932	0.0026147	-0.2556693	0.31323482	0.41521419	0.44994791	-0.0055685
0.06956092	-0.2026175	-0.2658428	0.37115099	0.39970604	0.35589343	0.19268416
0.03327288	-0.0958027	0.33155395	-0.6652064	-0.6484431	-0.6424009	0.25794471
0.13618585	0.09992239	0.12825854	-0.0112127	-0.0309952	0.02378385	0.12425758
0.03384669	0.14079501	0.26927869	-0.9802456	-0.9653501	-0.8965957	-0.6740379
0.17721397	0.12552192	0.19808963	0.22728376	0.15558334	0.14714179	0.00552068
-0.0913493	0.01810451	-0.1152817	0.68578164	0.68610004	0.77226707	1.0266247

-0.0179533	0.24962206	0.56791485	0.29015428	0.24767653	0.22388618	1.1092754
0.08758537	0.01281604	0.38460295	-0.0840495	-0.117239	-0.0651171	-0.5653535
-0.0045554	0.143263	0.18524707	0.5366985	0.51247511	0.56335801	0.17550062
-0.063162	0.1707601	-0.1626205	0.0445902	0.01141923	-0.0151998	-0.3939802
0.1147297	0.03457249	0.0224347	-0.1364544	-0.1247993	-0.1144808	-0.3026016
0.27380662	-0.0792753	-0.1069449	0.30955143	0.33369558	0.35719725	0.99721102
0.18178574	-0.2050335	-0.0842418	-0.4481018	-0.4989547	-0.4766516	-0.1273273
0.23494275	-0.000715	0.1112563	0.03704834	-0.025097	-0.0340099	1.0703894
0.11689691	-0.3651618	-0.3144752	-0.0592254	-0.0738672	-0.0206507	1.1542862
0.14534	-0.021601	-0.21718	-0.1863	-0.27657	-0.3173	-0.76722
0.02914074	0.17868422	0.3304222	0.23799991	0.27006056	0.27061903	0.55184589
0.10006854	-0.0151708	0.0342005	-0.2884568	-0.2601222	-0.2944969	0.0942436
0.02352832	-0.0713331	0.09377275	-0.2377451	-0.2327958	-0.2320621	-0.0704772
0.35442	0.012164	0.30501	-0.14199	-0.043747	-0.059323	-0.06072
-0.0543349	0.00269159	0.04295669	0.35533945	0.34176221	0.3506898	0.84787955
-0.09933	0.30742099	0.10124491	0.4793018	0.47978677	0.4758689	1.8577406
-0.0402203	-0.2731268	-0.3572553	0.19324459	0.15410247	0.16687546	-0.4893317
0.17486277	0.02663952	-0.0780394	0.1986645	0.3085744	0.27167646	0.6879076
0.03971632	0.422501	0.59252476	0.09585936	0.10314578	0.14778853	-0.0647252
0.04847369	0.35071465	0.60842909	-0.4190553	-0.3866202	-0.3813894	0.03617627

test_GSE135	test_GSE135	_GSE14238_	_GSE14238_	ref_GSE1742	test_GSE174	test_GSE174
ref_GSE1358	ref_GSE1358	ref_GSE1423	ref_GSE1423	ref_GSE1742	ref_GSE1742	ref_GSE1742
GSE13589	GSE13589	GSE14238	GSE14238	GSE17420	GSE17420	GSE17420
GEO	GEO	GEO	GEO	GEO	GEO	GEO
affymetrix	affymetrix	affymetrix	affymetrix	affymetrix	affymetrix	affymetrix
BIOFILM:-1,P	BIOFILM:-1,P	HEp-2:+1	HEp-2:+1		rpoS_b2741:	rpoS_b2741:
1899	1900	1911	1921	1973	1976	1977
-0.3268265	-0.3096342	-0.2009713	0.21948634	-0.5449628	-0.0567567	-0.0513024
-0.9369647	-0.8967798	-1.7824763	1.065559	1.5412593	1.1138448	-0.2404629
1.5281284	1.5723261	-1.9633506	0.53168092	1.5933744	-2.2377847	-1.0994493
-0.2065937	-0.2508283	-0.0663809	-0.219997	0.09576696	0.10444337	0.01506344
-0.244101	-0.2258384	0.11369552	0.26493467	0.74192578	-0.6922819	-0.7037746
-0.2383053	-0.2622791	-0.6087157	0.30295788	0.76406982	-0.1766252	-0.4429324
-0.5332064	-0.5215038	0.33910392	1.9006928	-2.1972386	2.2337378	1.4330208
1.7221044	1.7182689	-2.0307784	-0.6795524	0.93087827	-0.8430877	-0.8497769
0.16491507	0.18722055	-0.3643273	-0.2433842	1.2818167	-1.3307491	-0.8888465
0.2731388	0.28880866	-0.3781944	1.1203996	0.03715494	-0.7057965	-0.283285
1.3297004	1.4583798	-0.2740303	1.6159237	0.3639477	-0.3202917	-0.7946176
0.66551526	0.61695616	0.37052251	0.57957115	1.4415418	-0.5152709	-0.6724538
-0.8211284	-0.6957382	-0.1292954	-0.2224293	-0.0160505	0.0742441	0.00614273
-0.7021296	-0.862164	-0.0417308	-0.025396	-0.1700312	0.19975913	0.29569863
-0.0819235	-0.5413495	0.59107381	0.44533963	-0.9194474	0.62689651	0.12990858
-0.1778381	-0.2182979	-0.1218585	0.29216712	0.51551762	-1.3242478	-0.7151599
1.8558	1.8413	-1.2242	-0.21036	0.60778	0.33684	-0.20194
-0.1440656	-0.1376162	0.01147584	0.02756303	-0.1927755	0.17836133	0.13106779
0.22059002	0.33711352	-0.756525	-0.6344788	1.1591172	-0.5375271	-0.8453899
1.3700083	1.3942879	-1.4835672	-1.7713987	1.2485066	-1.1919864	-1.2147798
-0.1631227	-0.0279469	0.56766251	0.92293459	-0.0697413	0.3893666	0.01936483
2.8366	3.2273	0.073409	0.0095227	0.69201	-0.15965	-0.32984
2.5193667	2.490257	-1.1745333	0.25269433	2.4595335	-1.7089621	-1.8889938
0.92169	0.84878	-1.944	-0.34216	1.6956	-2.5712	-1.9803
0.61249607	0.59027693	-1.4607237	-1.103583	-2.5265803	-0.3979905	1.1585501
-0.5711884	-0.6301652	-0.6529916	0.26563219	0.13051035	-0.0077127	0.11388903
2.2647	2.3084	-1.2915	0.88539	1.4259	-2.2484	-1.6168
-2.1203723	-2.1199013	0.51760848	-0.9916958	0.52281148	-0.4133524	-0.2326974
0.86064	0.90522	-0.15067	3.0814	-0.20445	0.076827	0.22355
1.0365667	1.0455576	0.55644046	2.0827369	0.55460506	-0.2154784	-0.665272
-0.4061042	-0.3860282	0.13552087	0.57794636	-0.168933	0.06437662	0.16391809
0.86466345	0.90669763	0.16528166	0.56342088	-0.2840425	-0.0639796	0.25279904

-1.1449288	-1.1573567	-0.3561783	-0.213363	-0.5477642	-0.0257848	0.21098613
-0.1495427	-0.0294365	0.08052898	0.61515743	0.56635217	-0.3795147	-0.3371235
0.20688734	0.21305888	-0.0478253	0.46897174	0.37318839	-0.2466618	-0.0576646
-0.2239307	-0.2765794	0.54395024	0.86403602	0.1992809	-0.067446	-0.3853748
0.89178861	0.84440181	0.0306728	0.06700861	-0.049049	0.06883942	-0.0045412
1.6312185	1.7719507	-0.089471	-0.0997049	-0.055811	0.08978789	0.02562737
0.09249785	0.19403975	-0.1714755	-0.1608378	-0.1185487	0.15206112	0.03602248
0.2944562	0.25614787	0.04966391	0.3084989	0.65408451	-0.5289059	-0.7379706
-0.6076321	-0.7001827	0.21225181	0.45787828	-0.0323915	-0.1389521	-0.1435964
-0.1887512	-0.336799	0.72461347	0.77701606	-0.0154111	0.26937076	-0.2027564
0.05009397	-0.0271659	0.34076806	0.60533302	-1.2219145	0.31316995	0.58754375
1.3219078	1.2788793	-0.4624736	-0.0445031	0.13521669	0.29015958	0.293259
-0.2457743	-0.1725813	0.01862975	-0.0220149	-0.5032501	0.69864027	0.33976841
-0.3791509	-0.3742654	-0.7785773	-0.771288	0.09450687	-0.161636	-0.1219121
0.28523229	0.31430624	-0.4547162	0.0523114	-0.0523531	0.2506354	0.05050519
-0.766447	-0.7231855	0.43719614	0.41178283	-2.0409245	0.76186073	1.2639281
1.4501411	1.5030931	-0.1594375	0.18497489	0.11423045	0.37122007	-0.1709421
-0.2622653	-0.2540028	-0.0093712	-0.0560127	0.18558849	-0.1045062	-0.1321602
0.2692155	0.39003229	0.02849712	-0.5559234	-0.9942056	0.58642739	0.34471218
-0.4000608	-0.4132754	0.28482367	0.39239544	-0.273134	0.14401984	0.3113859
-0.1305817	-0.0602573	-0.9847579	1.66544	1.8545853	-1.8319799	-1.8334851
2.5491556	2.5617556	-2.7720205	-1.8960666	0.76499302	-2.6803244	-1.4265015
1.447383	1.4564119	-0.1691923	0.65344774	0.00895756	0.38413293	0.41617975
0.18342704	0.18509851	-0.3450697	-0.681232	-0.6341586	0.51354919	0.4264595
1.0405832	1.149276	-0.0329337	0.71388266	-0.5215842	0.10460556	0.09122249
2.1392916	2.109758	-0.9894145	-1.0115884	3.6051649	-2.9509918	-2.2641912
-1.978546	-2.0523817	1.0266367	0.09700505	0.78877629	-0.8454819	-0.6567435
-0.291619	-0.3291444	0.17277374	-0.0492634	0.08891132	-0.0484233	0.05705724
-0.1394741	-0.2048562	-0.5129076	-0.9218084	-0.3686441	-0.6566616	-0.0451111
-0.4917658	-0.3236458	0.44646024	0.42583883	-0.6596618	1.2004956	0.55665234
-0.0339759	0.01944586	0.72157259	0.11915016	-1.0692929	1.3097805	0.80088755
-0.068958	-0.0793958	-0.0710343	0.94449674	-0.4670038	0.21434273	0.32128001
1.0753922	1.1647053	-0.0940771	0.60638074	-0.0144964	0.09646997	0.26776343
1.4445652	1.3951201	0.04744469	-1.6028006	3.8892272	-3.3555406	-2.6929424
1.3008545	1.3284976	-0.6647188	-0.9922473	1.4361916	-1.4453219	-1.4884959
0.52037141	0.54868875	-0.1859034	1.5454516	0.09524523	-0.1339574	-0.032788
-0.5404602	-0.4954989	-0.8496281	0.12455775	0.09842686	-0.237653	-0.4150536
0.23289911	0.10569159	0.13034517	0.13410962	0.10098159	-0.0812562	0.00230972
0.23220606	0.31516728	0.06742996	0.08158824	-0.0774747	-0.0800714	-0.0384614
-0.1244097	-0.2040763	0.26149707	0.26204141	-1.0533289	0.44866162	0.34379297
0.02362386	0.03688701	0.21104159	0.1604541	0.07572776	-0.0331001	0.0412814
-0.1694579	-0.162366	0.35472819	-0.2727327	-0.3806749	0.39470778	0.30251517

-0.9408493	-0.9114627	0.45708555	-0.001912	-0.023545	-0.2901939	-0.0361915
0.00287328	-0.1205031	0.46461525	0.78387857	-0.2974382	0.18488577	0.3271919
-0.1606597	-0.2190137	0.21391476	-0.8814185	-0.6049182	0.84770033	0.33516021
1.3043876	1.3270604	0.40195188	0.21167448	2.4374495	-2.0110822	-1.8381011
0.11498265	0.11860647	-0.5691103	0.32281597	1.0306413	-0.7736726	-0.7532291
1.0317437	0.99542636	-1.2090089	-0.4176215	-0.6340525	-0.4647449	0.18282709
-0.0226529	-0.2009034	0.3285455	0.28727644	0.36589099	0.28697238	-0.0819445
1.7850893	1.7604541	0.15227298	0.2491755	0.12249498	-0.1448713	0.02087281
0.51239463	0.38114664	-0.6360022	-0.2664051	1.2574204	-1.2331754	-1.1570794
0.25458618	0.20073541	-0.1560872	0.49270864	0.62397873	0.51213225	0.15637841
0.09749017	0.28254424	0.0047568	-0.5763421	0.80730892	-0.9057286	-0.5454678
0.28170674	0.38728047	-0.0621206	-0.006573	0.0951711	-0.0465507	-0.1884204
0.80790514	0.7087296	0.13382674	0.07251775	-0.097695	0.03570546	0.10080629
1.0182083	1.1307079	0.03298475	0.12423204	0.0898653	-0.1446755	-0.1745151
0.55979	0.72135	0.14161	0.55423	1.9642	0.027275	-0.24413
-0.0262022	-0.4155215	-0.2143873	0.02573253	0.16287938	0.47682346	0.37518375
1.7643567	1.7640662	-0.209093	0.5036047	0.17887737	0.20882116	-0.0847308
0.44475173	0.49947603	-0.0698569	-0.4843822	-0.0679884	0.38180076	0.07310214
0.71728	0.82365	-0.0041766	-0.12554	2.4309	-2.7262	-2.4432
0.64233608	0.61115826	0.53766872	0.60407086	-0.3657245	0.16032699	0.18363808
0.29636085	0.2256849	0.43424334	0.40618884	0.23519737	0.02554119	0.06046079
0.69545	0.63798	-0.53621	-0.40619	0.22008	-0.05818	-0.51316
1.0240521	1.1151589	-0.1369813	-0.1518415	0.13238729	-0.054128	0.05851339
0.65320554	0.77660314	-0.452393	-0.1655947	1.6948449	-2.037284	-1.3295281
0.4019741	0.54322573	-0.8848571	-0.4545916	0.20583223	-0.2265696	-0.097903
0.52347551	0.44379691	-0.308332	-0.4808692	-1.8173169	0.37426502	1.2000665
0.04726491	0.03561042	-0.3107361	-0.035365	-1.3650009	0.58737356	1.1512187
-0.7092206	-0.6583383	0.43263628	0.10868432	0.09706551	1.7469982	0.19854188
-0.23367	-0.079616	0.052261	0.047642	-0.15502	0.22187	-0.029242
-0.060763	0.23629	-0.0019109	0.095888	-0.13795	0.034139	0.17957
-0.27547	-0.27717	-0.113	-0.14749	0.039353	-0.041824	0.026706
1.7594	1.7281	-0.13001	-0.058223	-0.16732	-0.0039994	-0.048859
1.5351	1.534	-0.0066304	-0.19389	-0.10047	0.078756	0.092122
0.52363	0.63472	-0.11447	0.16841	0.13374	-0.12136	0.062969
0.32702	0.32631	0.040922	0.010228	-0.070347	0.13891	-0.071109
0.88903407	0.89894599	-1.3795734	-0.0028659	1.7066791	-1.0975816	-0.8177235
0.25979275	0.17527782	-0.1975267	-0.2380998	0.95027805	-0.8081448	-1.0419892
-1.2540609	-1.2595164	-1.2775676	-0.8374311	1.0346318	0.53732207	-0.4293003
0.65847424	0.70475823	-0.0306625	0.6195824	0.61126541	-0.2362634	-0.2006834
0.18252696	0.33434786	-1.3633274	4.0093337	-0.2840941	-0.6559148	-0.0807772
-1.1852442	-1.2670788	-0.0317907	0.20939355	-1.5730643	-0.0165012	1.3038004
-0.0129071	-0.0083226	-0.2499255	-0.3548191	0.0302944	0.11178904	0.03542333

0.87741144	0.83602657	-0.3626326	-1.1656619	-0.7177963	0.04006078	0.90522694
-0.8207212	-1.0617584	0.05120467	-0.6822017	-2.2946512	1.4258238	2.329385
0.70281697	0.89489702	-0.5038383	-0.1238085	-0.6113045	0.0761783	0.38263394
0.19523558	0.21991453	-0.1356367	-0.1253432	1.0510033	-0.8533677	-0.8336333
0.27145317	0.38386574	0.35968572	0.40539392	2.2365469	-0.5174788	-0.7767376
0.55828993	0.61604675	0.08609087	-0.3903972	0.66239022	-0.7318741	-0.5263811
0.24474671	0.36606445	0.08086075	0.30486723	0.10735602	0.11589316	-0.0660735
0.71045279	0.85372031	-0.8469564	1.1893063	-0.3055407	-0.3918904	0.13744683
0.49455051	0.37178535	-0.277782	0.59152838	0.3564045	-0.1958138	-0.5982554
1.3525035	1.334426	-0.9850502	0.16782595	2.4489287	-2.397524	-2.105985
0.76570363	0.72897069	0.24650898	-0.1970702	-0.1319027	0.38628098	0.48793148
0.71473955	0.7065924	0.11652068	1.0306182	-0.0649187	0.41105539	0.27137293
0.40529814	0.42223465	-1.1351445	-0.3457339	1.0869062	-0.8364896	-0.5676622
0.7808267	0.84007431	-0.1060628	0.07973819	-0.1427017	0.27557626	0.46131241
0.3735331	0.4616259	0.10573405	0.21262307	-0.025892	-0.0295705	0.48264446
-0.8202443	-0.8337308	0.45925551	1.157398	0.3755998	0.63527929	0.16298806
1.1935825	1.2460844	0.32080141	0.48670631	0.21289107	-0.2655992	-0.4172901
-0.0573664	-0.0940889	0.14086624	0.14990355	-1.0728477	0.66800573	0.59580991
0.09452803	0.06244989	0.25656123	0.42282127	-0.0891488	0.68727128	0.19540365
-0.8284308	-0.7812543	-0.062551	-0.0693372	0.15791585	-0.1499633	-0.2608794
-0.2156355	-0.2144896	-0.3936322	-0.6536736	1.1118971	-0.7586123	-0.8818827
1.2833765	1.2587079	-0.7351804	0.12735932	0.53681215	-0.6811347	-0.7495516
1.2211	1.1513	0.0025538	0.011072	-0.028693	0.19321	0.10165
-0.2742838	-0.2412217	-0.0975713	0.12702885	-0.1950997	0.11920856	0.07448271
0.80795	0.7462	-0.53749	0.10512	1.1988	-0.58741	-0.96944
0.43097839	0.46766838	0.02492742	0.08628145	-0.0992612	0.06752134	0.20971931
-0.4272555	-0.4333592	0.50545506	0.91331542	0.45489972	-0.3683196	-0.187954
1.1878535	1.2065535	0.21645658	0.64269581	-0.6379095	0.01034741	0.57447405
0.30369042	0.35933061	-0.2718858	-1.3339799	0.38289202	-0.0128946	0.0721881
1.2385	1.3408	0.36151	0.10354	0.40708	0.057284	-0.31358
0.30526747	0.31363662	-2.262382	1.2717446	2.8464788	-2.6447743	-2.0018851
0.50581345	0.44189553	-0.035591	-0.1206491	0.02583696	0.06820305	-0.1471357
-0.406784	-0.3695715	0.22407288	-0.4093408	-0.7293953	0.25492975	0.51396451
-0.2871662	-0.0830765	-0.3170032	0.04722162	1.4086435	-0.3875606	-0.9927271
0.01008216	-0.023039	-0.5354143	-0.2325397	-0.039744	-0.0978979	0.04449832
1.1697742	1.0938908	-0.5932765	-0.6697704	0.51967799	-0.2826667	-0.4833421
0.19669953	0.17656277	0.15345502	0.05245203	1.0204781	-0.0871243	-0.3333663
0.56252804	0.57382335	0.35417574	0.22297863	-0.0110282	0.00901347	-0.0088284
1.3244419	1.3785431	-1.5349101	-0.9879849	0.73831438	-0.8199243	-0.7550232
0.8071892	0.82696206	-0.503124	-0.0750727	0.17416621	-0.0068108	0.33686492
0.7376229	0.59073695	0.03409233	-0.0652747	0.02112401	0.15466156	0.02697857
-0.7590974	-0.7563427	0.80497528	0.35783608	1.6002525	0.69104092	-1.2442702

0.54978938	0.49456829	0.31197502	0.24044375	1.0776536	0.07018845	-0.8711133
0.28390412	0.2601138	0.24799714	0.21326414	-0.1849761	0.2473646	0.1653459
0.33398655	0.35290825	-0.8288615	0.28558119	-0.3847339	0.25872656	0.2079571
-0.043382	-0.0447656	0.34945669	0.20878771	0.23782707	-0.1023977	-0.0951723
0.18837764	0.11724135	-0.2477575	-0.2138478	-0.1676605	0.19988973	0.25579185
0.03855607	-0.0304927	0.10546152	0.30963845	0.24433915	0.00568757	-0.1042477
0.55128969	0.48483153	0.01136779	-0.1038438	0.34838653	0.0869479	-0.1708372
0.30957533	0.32121251	0.46556499	0.03440197	-0.3375972	0.30668886	-0.5025482
1.3923168	1.3116089	-0.9611282	-0.8104742	3.5956239	-3.2426012	-2.5370068
0.74809954	0.71993815	0.01717148	0.9566041	0.3642636	-0.1944798	-0.5125518
0.45314394	0.45519678	0.46304648	-0.1591887	0.36762076	0.37853411	0.06608319
-0.1144503	-0.2070079	-0.2907996	-0.0991574	-1.0069275	0.83300184	1.2052644
0.59411625	0.60904872	1.42E-05	0.14122268	-0.007395	0.0989286	-0.0261151
-0.5392212	-0.423819	-0.0302646	0.00774363	0.07587862	-0.062067	0.00483464
0.58899498	0.51214715	-0.4580352	-0.4400578	-0.2348212	-0.4698064	-0.0237073
0.44595844	0.47365465	-2.5740946	0.43686946	-0.2775539	-0.4557763	-0.395227
-0.0337694	0.11778865	0.05328231	0.40222775	-1.2465499	0.4503179	1.2623271
-1.2784254	-1.3615318	1.7015083	0.99244972	0.39529438	0.72270377	-0.1395169
-0.200929	-0.3675532	0.63756323	-0.6078066	-0.7084728	0.51949261	0.62692367
0.32051225	0.43598258	-0.2792126	-0.2352711	1.1401322	-1.0183428	-1.2276215
0.26273913	0.31917807	-0.2791116	-0.4429254	-0.0441652	-0.0783697	-0.0637015
0.28727714	0.23539093	0.01915029	1.6950137	-0.7977906	-0.4158988	0.32476329
-0.0548513	0.02382302	-0.5565893	0.26547659	0.23366478	-0.7765977	-0.1487676
-3.2668883	-3.1414552	-0.5404598	-0.1110039	-0.8416712	-0.1192267	-0.008183
0.32778287	0.26214469	-0.207198	-0.3811423	0.03779465	0.67353234	-0.083199
0.38423502	0.31305888	0.04413739	0.14068191	-0.4028608	0.34275665	0.51887337
0.23143096	0.19704172	0.05542049	0.44397381	0.36979694	-0.1381055	-0.1631657
-0.1243134	-0.1920627	-0.6994618	-0.3357945	2.6156353	-2.7117237	-1.6684968
0.1956705	0.20372649	-0.3912372	0.72456274	-0.2354317	0.56369787	0.46404358
0.06760006	0.07436444	0.25021168	1.0943513	-0.1700542	-0.2923217	-0.1259832
-0.1238452	-0.0673097	-0.1167966	0.56522337	1.0342401	-0.6293429	-0.9517756
-0.2437052	-0.5030157	0.33121153	0.52591869	-0.3395111	-0.4503546	-0.2193815
0.22376769	0.14733153	0.78221002	0.79570864	0.07480557	0.81289854	-0.4158938
-0.2412342	-0.2243139	0.7954935	0.82641674	-0.9622978	0.42303947	0.36315955
0.78493014	0.78229259	-0.4201244	-0.4035877	-0.3735487	0.21407851	0.31294711
-0.7015001	-0.8472339	-0.2506525	0.59284078	0.73959823	-0.39411	-0.40083
0.4191	0.39416	-1.0427	-0.53087	0.13925	0.057662	-0.29082
-0.0184986	-0.0673886	-0.7580107	-0.2956901	0.10000003	0.16721122	0.21473364
1.4233377	1.4366502	-0.6090686	-0.5120804	0.05489896	0.33261073	0.23901201
1.203	1.2145	0.051783	1.364	0.55388	-0.96479	-0.48268
2.0581567	2.1605863	-0.2148819	-0.0793399	0.04583894	0.01075056	0.0385197
1.79366	1.7912181	0.16894563	0.18770848	0.02109886	-0.0264582	0.06661866

0.03304883	-0.0515991	-0.2365508	0.2560172	0.34705911	-0.3174875	-0.1232288
0.04601449	0.32359336	0.09280402	0.29365323	-0.004398	0.28254767	0.13690045
0.59426591	0.67622784	-0.6016844	-0.1085207	-0.7857742	0.09080647	0.39680975
-0.5363611	-0.5309992	0.10979657	0.01002142	-0.0174639	0.24327512	0.0853371
0.08390267	0.17029934	-0.6072243	0.66104292	-1.8893473	-0.6156253	0.84897234
0.10546443	0.06551884	-0.4497317	1.873355	0.55489432	-0.5673711	-0.1411163
-0.2350238	-0.3783998	0.05366729	-0.197938	-0.8225211	0.06365746	0.29108967
-0.3966436	-0.1899319	-1.5089261	0.47070528	0.86578229	-0.7595131	-0.7518231
-0.4509216	-0.00797	1.3132852	1.7071386	0.11802395	-0.0408072	-0.1997634
-0.49316	-0.50882	-0.19521	0.29715	-0.279	0.2957	-0.089287
-0.0168293	-0.0363136	-0.0087807	0.05879372	-0.9311456	-0.2896805	-0.2915518
-0.5722974	-0.4819867	-0.1236594	1.0717258	0.99133698	0.06469272	-0.4291004
-0.1398634	-0.089256	-0.9551086	0.37982369	-0.2905183	0.06357315	0.09436559
0.01134853	-0.1044672	-0.0488985	0.54093052	-0.6796371	0.11307328	-0.0383295
-1.8202957	-1.8349514	1.5334109	-0.5167254	-2.4010941	1.9877339	2.2649635
-0.3368794	-0.3792074	0.53107286	0.74534515	-0.0690925	-0.0109985	-0.0034208
0.04380079	0.06497275	-2.2809057	-0.567404	0.65289446	-0.9038619	-0.7898113
-1.5784764	-1.6246993	0.26760771	0.83516291	1.3849488	-0.438381	-0.5029705
-0.10988	-0.009774	-0.2982131	0.47956261	0.07162392	-0.4511445	-0.0391257
0.64384735	0.60946469	-0.9143388	0.44632923	0.48887257	-0.7454839	-0.676786
0.56312606	0.61866187	0.09722502	-0.0142175	0.136487	-0.1009433	-0.0846122
0.31613861	0.31175263	0.17547739	0.00281422	-0.1238726	0.04090866	0.04866962
-0.0146643	-0.135904	-0.0689733	-0.1601489	-0.3386499	-0.3278271	-0.005397
0.38461948	0.47466394	-0.1806626	0.78914429	1.2415928	-0.6779971	-0.7482099
-0.3410683	-0.1531347	-0.2249542	0.25243223	-0.0400697	0.11960371	0.14627401
0.26597535	0.15316496	-0.1968129	-0.1350469	-0.0067036	0.08214214	-0.0053361
-0.9368876	-0.9906405	0.58304737	-0.2844581	2.7609257	-0.9046334	-2.8990353
-1.1297775	-1.173	-0.7389585	-2.0684397	4.1565561	-1.5554523	-2.2511235
-2.0982165	-2.0131914	-0.8683021	1.2097714	3.1268058	-0.8505265	-1.4912117
-0.3452012	-0.3681481	-0.3758415	0.17865945	4.0899834	-3.3561366	-4.0646817
-0.229001	-0.1870375	0.18600793	0.85780734	-0.8598215	0.36621218	0.65745602
-0.2217498	-0.279992	0.245102	0.95639303	1.1648632	-1.6380109	-1.2375237
1.3479719	1.3804733	-0.1070989	0.77508874	3.9259368	-4.6949427	-4.5031984
0.01775001	0.11687511	0.54721965	0.85343043	-1.2140985	1.2078942	0.13391099
0.01216585	-0.1841286	-0.1624764	-0.3607866	0.58261283	-0.7084583	-0.876613
-0.3144338	-0.3072965	-0.5069536	-0.4577226	0.00314962	-0.1858651	-0.0350205
0.58865753	0.5185841	0.07642155	0.23280858	0.90941388	0.61142164	-0.565382
0.71489295	0.73473776	-1.1457784	-0.5015663	-0.5227148	-0.7768735	0.31461167
0.3900806	0.37882945	2.4444648	-0.5259661	2.3424976	-2.3772546	-1.7436741
-0.4078849	-0.1353937	0.07176379	-0.2069732	-0.8953691	0.39796572	0.90324226
-1.4952859	-1.5781935	0.15190107	0.00402828	-0.0205384	0.06641621	0.04758513
0.32702679	0.24452243	-0.1483812	0.0464916	0.72786848	-0.6122203	-0.7213481

-0.2238076	-0.2478875	0.16049927	0.49635371	-0.4271299	-0.1204891	-0.0524062
1.6736	1.6515	0.097327	0.03486	0.14104	-0.073725	-0.053407
0.02734904	-0.1743382	-0.0401178	-0.1942589	-0.1280838	-0.3411942	-0.0024506
-0.3990263	-0.5601321	0.20414962	0.49151638	0.11119091	0.36470789	-0.1437339
-0.0952099	-0.0223087	-0.0231736	-0.0853791	-0.0346649	0.12108367	0.07729483
0.21766191	0.22056723	0.33349761	0.35869331	0.57216767	-0.1205293	-0.5512039
-0.7565538	-0.8018846	-0.4158858	1.9691593	0.49861986	0.40277572	-0.0595553
0.26141	0.27708	0.39968	0.45669	0.49361	0.18932	-0.23892
1.1205917	1.1165339	0.07903687	0.19386459	0.24089486	-0.2983728	-0.0765599
-0.96263	-0.96863	-0.42609	1.8483	-0.61534	0.16375	0.86456
0.134786	0.21177877	-0.7147432	-0.5933061	0.17789167	0.21767418	-0.1944504
-0.10625	-0.0092562	-1.5718948	0.31582052	0.5899017	-0.5575582	-0.6351274
0.72630585	0.73857749	-1.0845407	0.61579455	0.62040097	-0.9785937	-0.8335514
-0.0255823	0.1877544	0.19250236	0.22165119	-0.0291599	-0.1290759	0.00217864
-0.4069772	0.00488314	-0.1712224	-0.1003671	-0.0744762	-0.2504106	-0.108161
-0.3184232	-0.1738409	-1.0539025	-0.2917923	0.41754499	-0.6693123	-0.5202211
-0.5314106	-0.5337571	0.0187344	-0.1800695	1.0892105	-0.4921826	-0.8541902
-0.0304601	-0.0365994	-0.0075086	0.36298913	1.5147651	-0.4294401	-0.7324897
-0.6658221	-0.6277752	-0.5992807	1.9906956	-0.9621891	0.77798169	1.0105951
-0.3713869	-0.3647063	0.14899076	0.10538605	1.0193219	-0.5632811	-0.9048364
0.05361975	0.02686033	0.42662661	-0.1306392	0.40125972	-0.3565226	-0.1831606
-0.1953326	-0.1309234	-0.8768657	-0.3479365	0.39109948	-0.6970014	-0.3310785
0.24739	0.1874	-0.23973	-0.60024	-0.99285	0.80473	0.6535
-2.2460015	-2.260263	0.25684995	-0.468113	0.37552101	-0.4388139	-0.2975307
0.10263836	0.12897783	-0.7350595	-0.4735502	-0.4176658	-0.0457287	0.1364512
-0.8697118	-0.8469119	0.22723109	1.9221479	-0.1715976	-0.0827583	-0.0892983
-0.1592021	-0.187519	-0.3627142	0.83033196	-0.642666	-0.1595881	0.20091476
-0.016306	0.056117	-1.1265	-1.0906	-0.35173	0.30769	0.52729
1.4126131	1.4360118	-0.5830418	0.25298291	2.3236528	-0.4304351	-0.1596069
0.54163361	0.4925273	-0.9864302	1.0078989	-0.8387375	-0.2263614	0.12472458
0.48770711	0.63030251	-0.0968989	-0.0502416	-0.0304384	0.03942493	0.24130792
-0.4442942	-0.4341009	-0.0219642	0.31641062	0.01078401	0.01376429	0.04152326
0.015998	-0.10053	-0.11726	-0.31613	1.0609	0.27327	-0.52754
-1.902662	-1.9626351	-1.3084498	-0.3633979	1.6787498	0.47016486	-1.2200374
0.99382708	0.98735407	-1.7896519	1.90659	1.5491157	-1.0717406	-1.3475889
-0.142953	-0.4026979	-0.2588719	-0.5641533	1.6326148	-0.3722506	-0.9984227
0.05630893	0.11493965	-0.2142025	-0.1014811	0.09132918	1.1202088	-0.1625247
0.18011502	0.18452039	0.82413353	0.47774029	2.7015948	-2.2468203	-2.3331329
0.13653729	0.13410287	-0.2891941	0.41672541	0.11347653	-0.3481008	-0.1203483
-0.6258583	-0.6434075	-0.3881889	-0.0425058	3.4284282	-3.618187	-3.4789083
0.06105744	-0.0311813	0.06171119	1.3576839	-0.8750851	0.11182712	0.62939029
1.0316754	1.0748047	-0.4633748	1.2357288	-0.5536184	-0.3651922	0.29642936

1.1079885	1.0970553	-1.8935901	1.1176521	-0.181268	-1.0101631	-0.4126332
-0.5242079	-0.5240901	-0.3513864	-0.105509	0.95334278	-0.9281945	-0.4360878
0.08688462	0.31519861	0.13706678	-0.0289044	-0.7325015	0.04348983	-0.2384796
-0.4589404	-0.4281782	0.47725928	-0.0121008	0.42697372	-0.7740217	-0.6866354
-0.4168237	-0.3330708	-0.0812116	-0.1115059	0.00134177	0.08324992	0.05084089
1.0003264	1.0591267	-0.1610441	-0.2117929	-0.0546098	-0.02154	0.02862592
-0.0194799	-0.1208872	-0.0325499	0.07764909	-0.1058908	0.12387499	-0.0073737
0.92482373	1.0075406	0.0232026	0.0596205	-0.1549114	-0.094159	-0.018989
1.1982513	1.1986372	-0.2175375	2.1132317	0.05702612	-0.24369	0.11872284
-0.89862	-0.67197	0.22585	0.40593	-0.33197	0.11902	0.18564
0.6077755	0.59138856	-0.0526173	-0.0219199	0.4168038	-1.101173	-0.6235741
0.07043567	0.10217354	-1.4844682	0.87534659	2.6825182	-1.8701603	-1.4514644
-0.1617973	0.06988987	-0.0184513	0.27037178	-0.0361292	-0.0670204	0.00414562
-0.047222	-0.10977	0.34884	0.80283	-0.12152	-0.51594	-0.31913
0.88458883	0.87254891	0.14038796	0.08953963	-0.0134925	0.05207649	0.04427422
1.8701101	1.7994065	0.27620447	-0.2857	0.32545329	-0.3376593	-0.2091927
-0.5037602	-0.6398774	-0.1653726	0.41359304	-0.4887274	-0.1086168	-0.0517576
0.66481066	0.66296925	-0.285388	0.39143969	1.6214719	-0.8448127	-0.7428481
-0.175309	-0.1427762	-0.1699056	-0.0182847	0.39321902	-0.9730106	-0.6042708
0.03167122	0.07974678	-0.5897269	0.41251526	1.0238782	-1.0660122	-0.8552594

test_GSE174	test_GSE175	test_GSE175	test_GSE175	test_GSE200	test_GSE218	test_GSE218
ref_GSE1742	ref_GSE1752	ref_GSE1752	ref_GSE1758	ref_GSE2009	ref_GSE2186	ref_GSE2186
GSE17420	GSE17526	GSE17526	GSE17584	GSE20095	GSE21869	GSE21869
GEO	GEO	GEO	GEO	GEO	GEO	GEO
affymetrix	affymetrix	affymetrix	affymetrix	affymetrix	affymetrix	affymetrix
rpoS_b2741:-1					PaeR7I M:+1	PaeR7I M:+1
1978	2021	2022	2042	2052	2071	2072
-0.1916943	0.0405197	0.22763083	-0.1223972	0.00427727	-0.0587766	0.76896838
1.0978353	0.36870301	1.0177062	0.63727737	-0.9705446	-0.046465	-0.0550025
-1.1481387	0.01203179	0.33912575	1.6766413	0.48751142	0.15410766	-0.6192309
-0.0007803	-0.1600856	-0.2182989	0.75597892	-0.0105985	0.30977224	0.11269697
-0.8259136	3.62E-06	0.24758908	-0.2058275	-0.0803615	-0.0360317	0.19020169
-0.3478828	0.06356705	-0.1847137	0.67713272	-0.1601265	0.25864342	0.30090077
1.9463602	0.03603952	-1.6610779	-1.0679667	0.29021239	0.10817469	0.27737268
-0.9360426	0.13001566	0.27777844	0.09022173	0.13445207	-0.6228913	0.44206891
-1.1727538	0.06517247	-0.1128405	1.2298699	-0.1251193	0.11554051	-0.0970583
-0.3314914	-0.0818901	-0.147478	0.63244048	0.14232977	-0.1084941	0.19293809
-0.7873722	-0.01218	-0.0560123	0.12349343	0.14899532	-0.0171125	1.3539481
-0.7603315	0.3612406	-0.2291919	0.31988857	0.16591209	-0.5027403	0.04742694
0.1299939	0.19475066	0.22369088	0.26438345	0.14234044	-0.2855421	0.33369782
0.11640783	0.14071853	0.10599868	-0.5162506	-0.3287536	-0.1787983	0.10772109
0.26187031	0.16623915	-0.2099223	-0.058692	-0.0348097	-0.1276034	0.03800309
-0.9868535	-0.0770129	0.13555834	0.16550796	0.06866675	-0.0229868	0.2885311
-0.13294	-0.4665	-0.8517	-0.9761	0.23163	0.16041526	0.6131482
0.11156356	0.31138467	0.07898149	-0.023498	-0.0978543	0.2183774	0.80838774
-0.7505619	0.00729303	-0.0390146	0.68629277	0.04317823	0.24708067	0.17395951
-1.2698939	0.05729325	0.0881709	-0.0530667	0.00078915	-0.010764	0.25350308
0.23197294	-0.0512487	-0.5219096	-0.1347781	0.04251225	-0.1998384	0.05724847
-0.2645	0.15536	0.070059	-0.19674	0.13887	0.29831269	0.43287036
-2.1100519	-0.0475951	-0.0271441	-0.311722	-0.0008311	-0.0046731	-0.3655182
-2.1172	0.035421	0.39123	-0.025828	0.068152	-0.1449896	0.26922553
0.8413735	0.15691878	0.34610596	0.32930201	0.21858346	-0.0693769	-0.1327966
0.08628829	0.05476815	-0.0969861	-0.0662114	0.0119478	-0.2401496	-0.0874258
-1.6283	0.2753	0.865	0.46874	0.38118	0.10065402	0.95114538
-0.2733268	0.03419703	0.11165125	0.36565404	-0.1644911	0.19707106	0.68703926
0.18863	0.033752	-0.1624	0.073066	0.15215	-0.2069054	0.22734388
-0.7800726	0.02686601	0.13496047	0.07704206	0.06050821	-0.3819598	1.0333248
0.00569811	0.23514007	0.44103369	-0.3341691	0.11937437	-0.052338	0.69851628
0.25401402	0.19958928	0.28769193	-0.2488487	0.31997907	-0.1154851	0.30500339

0.18009663	-0.1068398	0.19136662	0.28163261	0.20913865	-0.2006261	0.11826379
-0.379402	0.0059801	0.1502223	0.19998787	-0.0105957	0.02458531	-0.0235393
-0.0283772	0.11914826	-0.1765599	-0.0144902	0.01092989	0.12984587	-0.1021043
-0.1965589	-0.0312863	-0.226578	-0.2415659	0.0546521	-0.0703706	0.03326904
0.11431334	0.27514832	0.18644636	0.03273402	0.02226682	0.06065582	-0.0369051
-0.0532865	0.01208468	0.24349736	0.22526229	0.4892061	-0.2281116	0.2513767
0.09838569	0.07013271	0.52944148	-0.1383701	0.70219987	-0.041938	0.06749299
-0.6221595	0.14715529	0.25704175	0.117264	0.28762426	-0.1115422	-0.1130845
-0.3103683	0.38335923	0.20874209	-0.332144	0.02184585	-0.3345211	0.08176614
-0.1401913	0.36390097	0.62496781	0.07890695	-0.1260164	0.02549035	0.09496554
0.36944677	0.06855082	-0.1692321	0.09629471	-0.0718946	-0.0585967	0.17434656
0.20646873	-0.0061299	-0.0626748	-0.6405327	-0.2494158	0.28663532	1.1614172
0.435092	-0.0314402	0.29403293	-0.2152234	0.03226672	0.08544506	0.0397588
-0.1252476	-0.0777589	-0.255974	-0.2547678	0.07215039	-0.206834	-0.1785017
-0.0162927	0.40056736	0.25444706	0.03474364	0.10670719	-0.6210076	0.5017899
0.83251103	0.26479066	-0.0017833	0.11657099	-0.0788783	-0.3132734	0.0633471
-0.1200146	-0.1237091	-0.0628386	0.25297921	0.11203199	0.13855923	0.18332342
-0.0590979	0.26573957	0.20251248	-0.1223091	-0.1004629	-0.0177298	0.26046675
-0.011297	-0.0045605	-0.0641174	-0.311137	0.03738552	0.02545883	-0.0378782
0.33070625	0.25543746	0.32846869	0.0800145	0.09484498	-0.0153016	0.22618868
-1.687422	-0.3578034	0.44623627	0.48357895	0.09872353	0.18668731	-0.3790043
-1.0276773	-0.1582333	0.39910814	0.05348933	0.10430018	-0.2287299	0.55611751
0.34699897	0.37065233	-0.2000661	-0.5999793	0.10602442	-0.0238385	0.45318522
0.34886432	0.2507839	-0.0682547	-0.5613922	-0.0463537	0.00822954	0.29940643
0.12510454	0.16059336	0.01324751	-0.0378952	0.01045437	-0.0789248	0.30149214
-2.552662	0.10430124	0.29209554	1.1368929	0.52271537	-0.324261	0.5688253
-0.6978292	0.08023754	0.05266941	-0.037941	0.00886091	0.06467237	0.58295407
-0.0091872	0.27886765	0.22508765	0.42846554	0.24664679	0.048188	0.02023351
-0.6456152	-0.0658694	0.09087526	-0.3636668	0.17316643	-0.3534456	1.3179741
0.664557	0.14067677	-0.0471546	0.49701858	-0.2363143	-0.109483	-0.1090572
0.71612714	0.01246618	-0.9216609	0.72660259	0.04658201	-0.0365133	-0.0449337
0.33285666	0.07602428	-0.018574	0.30611064	0.12473195	0.03373377	0.16934835
0.34803841	0.35089328	0.06848941	-0.3570512	0.02542662	-0.2083235	-0.3244935
-2.666005	-0.7951976	0.27371212	-1.3888685	-0.1143398	-0.0116163	-0.7762351
-1.7845174	0.07058111	0.20269454	0.13893677	0.53919688	-0.118386	0.10771718
-0.2093813	0.03506859	0.03323488	0.43693855	0.21584925	-0.1441657	0.13599724
-0.3012167	-0.0197757	0.11701403	0.26602033	0.0368725	-0.3453701	0.16812197
-0.0432339	0.13911099	-0.0549758	-0.1761737	-0.132504	0.06981733	0.6632319
-0.0761877	0.03781844	0.0423257	0.69104691	0.26622444	0.04151915	0.10397348
0.1639549	0.23408129	-0.1164362	-0.0539931	-0.215458	-0.2824032	-0.4151543
-0.0570613	-0.137875	-0.3127432	0.23310397	0.19278192	0.00343173	-0.1636446
0.34196262	-0.1314443	-0.1245133	-0.9320436	0.14745046	-0.3436553	-0.1132855

-0.1246739	-0.0622392	0.08373909	-0.3013865	0.059559	-0.5186904	0.38078019
0.4680497	-0.1920713	-0.4880888	0.52606701	-0.2943712	0.17940612	-0.1646051
0.01940269	0.19726029	0.03969839	0.64996276	0.09304105	0.16760439	-0.0988695
-1.9995372	-0.1618482	-0.2684962	0.25322068	-0.5565528	-0.5169267	-0.3849033
-0.6847136	0.07849395	0.1354577	-0.094449	0.16932789	-0.1145804	-0.2848923
0.49198943	-0.1333652	-0.1858456	0.25089803	-0.016191	-0.387586	-0.1807105
-0.0020339	0.30578715	0.16205183	0.12246427	0.02758737	-0.0199836	-0.1008517
-0.05705	-0.0134443	0.07469027	-0.0753602	0.01858193	0.06141401	0.26906324
-1.3649198	0.19737659	0.23468887	0.16448204	0.12201233	-0.0097421	0.00179979
0.16772507	0.00145584	-0.105254	-0.2205853	-0.032899	-0.179921	-0.2130911
-0.762932	0.17695625	0.38686351	0.51567342	-0.1624577	0.13233408	-0.2174973
-0.0652811	-0.0830804	0.11518041	0.17676762	0.05953863	-0.0530351	0.27483015
-0.0263031	0.4038985	0.39860673	0.00848524	-0.1734296	0.2873238	0.35284211
-0.227025	-0.2012962	0.25541992	0.43372439	0.05157255	-0.2954591	0.44233133
-0.4556	0.13397	-0.1218	-0.61729	-0.199	0.35216995	0.2130469
0.29817218	-0.0093056	-0.0857571	0.17381384	0.09320652	-0.1970225	-0.0566556
-0.0812514	0.15952127	0.05490318	-0.3459862	0.08720411	-0.5714771	-0.0328121
0.13989932	0.23106448	-0.2268057	-0.1541396	0.00709672	-0.217202	-0.25315
-2.4551	0.0926	0.83742	-0.0003811	0.12967	-0.2199461	0.70785802
-0.0459366	-0.2090166	-0.1070886	0.16611246	-0.1788801	-0.2724503	-0.133772
-0.0257531	0.35541887	0.03269595	-0.3424882	-0.0370119	-0.1173459	-0.2207269
-0.084784	0.053885	0.19917	1.195	0.22031	-0.1225237	0.0996205
-0.1302816	0.18516526	0.29743265	0.71396063	0.27064668	-0.0785034	0.3046015
-1.6420494	0.02184871	-0.008695	0.66535789	-0.0036498	-0.3527615	-0.0627049
-0.135988	-0.0828937	0.16092334	0.31285899	0.25151266	0.25707653	0.23169086
1.3342489	0.2969022	0.31857261	0.17974049	-0.0290698	-0.4007999	-0.3531108
1.1861523	0.45739132	0.34976065	0.17724337	0.15699469	-0.812706	-0.766258
0.51508107	0.19545222	0.1162858	0.06629815	-0.0020087	0.04513407	0.20458277
0.085134	0.18104	-0.15978	1.5536	0.065153	-0.070282	-0.22179
0.17448	0.10432	-0.21592	0.80772	-0.272	-0.15599	-0.043741
-0.038497	0.31775	0.35709	1.1703	-0.083137	-0.18935	-0.25478
-0.1066	-0.025707	-0.11858	0.51403	0.14235	-0.37845	0.45369
0.058079	0.013713	0.063819	0.24146	0.16386	-0.1521	0.62677
-0.1002	-0.10474	-0.14544	0.051515	0.11647	0.047206	0.10504
0.052001	-0.0019953	-0.080365	-0.041965	0.19945	0.0388	1.0263
-0.9518362	0.07542623	0.28181622	0.51405218	0.12380655	-0.111529	-0.2891484
-1.0150569	-0.2723438	-0.2197763	-0.1316024	-0.0705151	-0.0122146	-0.1643725
-0.5129377	0.32224731	0.20176826	-0.1524411	0.05870012	0.43947265	0.17252774
-0.2771192	0.00124675	-0.385284	0.29011187	-0.0132207	-0.128296	0.15925076
-0.3296156	-0.181623	-0.1427354	-0.0682616	0.11710324	0.08063209	-0.148859
0.65845086	-0.2177655	-0.0275428	-0.9838002	-0.0858547	-0.5615354	-0.0037129
-0.1085216	0.1578022	0.21097951	0.17801913	0.05362116	-0.369686	1.1675071

0.65257873	0.01653717	0.10166795	-0.9895777	-0.0983289	-0.2727036	-0.2380058
1.4655144	0.22713367	-0.0318148	-1.352755	-0.1539408	0.2293794	-0.0716377
0.29563247	0.04674011	0.16262923	0.08477172	0.26241972	0.15571061	-0.0951776
-0.7472042	0.05790736	0.22569706	0.43195563	0.05750586	-0.0462199	0.08129984
-0.6139246	-0.0942951	-0.1247391	-0.1679755	0.06023714	0.18376754	0.04681678
-0.5792685	0.03273309	0.34310975	-0.1463693	0.20564256	0.048144	0.72790322
-0.3118531	0.28355228	0.22539745	0.17043889	0.03580491	-0.0441646	-0.0606637
0.12948868	0.27841276	0.28821368	-0.0226626	0.02664742	-0.0987275	0.06335334
-0.5173048	-0.1095	-0.1788973	0.31057596	0.27507639	-0.1165562	0.28060562
-2.3471785	0.10525342	0.15325257	-0.0189196	0.13481851	-0.2617495	-0.2207926
0.25206326	-0.2469066	-0.3272212	-0.5990469	-0.2809149	-0.2597077	0.83563754
0.26914012	0.18452602	0.12487386	0.35786783	0.05674113	0.13390192	0.61747655
-0.8605853	-0.0676073	0.22586906	0.03418443	-0.1371539	-0.4329627	-0.5154143
0.53763476	0.40536899	0.18307851	-0.2911839	-0.0222067	0.16051487	0.36102378
0.36131015	0.54579894	0.43570664	-0.3551227	0.07622233	0.16207742	0.30848425
0.38227883	0.19061553	0.32180275	0.25047436	0.0588146	-0.5573447	0.27827335
-0.4751088	0.16106227	0.33298173	-0.2581257	0.06347416	-0.3722326	0.23847771
0.50832035	-0.028189	-0.072605	-0.796522	0.08233183	-0.0539752	0.03440293
0.07242427	0.06466279	-0.3487864	0.27729796	0.13681022	-0.3493722	0.15578695
-0.2507547	0.38038847	-1.0334256	-0.419759	-0.0858785	-0.2749679	-0.1410157
-0.8578642	0.16056283	-0.113144	0.17444259	-0.9239773	0.19434321	-0.0016201
-0.858489	-0.09583	0.01869035	0.51206775	0.1051793	-0.2112438	0.45419586
-0.013066	-0.24452	-0.57311	0.18669	0.025773	-0.094968	-0.14694
0.05452043	0.05756306	-0.1914887	0.20093767	0.08429916	-0.7137297	0.34845689
-0.90456	0.12225	-0.014968	0.19196	0.16015	0.07402532	0.20360484
0.08631194	0.10584241	0.39767206	-0.0392072	-0.0203691	-0.150552	1.0747725
-0.315048	0.1162307	-0.0057605	-0.0644523	0.05448624	0.01153265	0.01003369
0.35338051	0.03529944	-0.2473064	-0.4101301	0.13239223	-0.2586433	0.14488156
-0.2045643	0.30419709	0.16910787	0.23315599	-0.2369674	-0.0353018	0.20081531
-0.17684	0.20214	0.9714	-0.33455	0.5712	0.03547757	0.2091629
-2.5458651	-0.0731152	-0.1128805	0.17653529	-0.2359954	0.28766232	-0.1395379
0.08901657	-0.2569226	0.45033285	0.37613541	-0.0256059	-0.151953	-0.4570315
0.11666818	-0.1524942	-0.1814292	-0.1071365	-0.0976084	0.16466634	-0.1855475
-0.9729553	0.25428785	0.06909066	0.28409635	-0.0882014	-0.0710882	0.00370028
-0.1652798	0.21714597	-0.0354137	-0.3086278	-0.2295209	-0.0878396	0.01031934
-0.3740219	-0.1655431	-0.2903958	0.01291655	0.03184891	0.05291596	-0.1007908
-0.429469	0.08003617	-0.2467329	-0.1994954	0.04851075	-0.4219231	0.01252559
0.04753894	-0.1028629	-0.0514268	0.88024282	0.06463016	-0.0447163	-0.4763428
-0.68988	0.00728203	0.16958111	0.40236237	0.07341671	-0.0797042	0.01912819
0.00772524	0.03767213	0.07153755	0.50345283	-0.2467344	-0.1402305	0.01025019
0.0877422	0.36364983	0.08392349	0.89180362	0.07723779	-0.0479641	-0.233892
-0.7236292	-0.1112808	-0.3360008	0.00543855	-0.5034575	0.06568235	0.14283201

-0.7317324	-0.180373	-0.118551	-0.0248671	0.16857324	0.17456853	0.16140368
0.02098322	0.01222846	-0.016324	-0.1031763	0.05899263	-0.0266251	0.15473116
0.2239333	-0.1705524	-0.1227724	0.24637453	0.11710808	-0.110096	-0.1337704
-0.1181015	0.30344756	0.509924	0.45776435	-0.1437736	-0.2019327	0.02811737
0.09619003	-0.114809	-0.1872272	0.11931298	0.21554622	0.13190372	-0.1097847
-0.1117204	-0.0401853	-0.046804	0.01358308	-0.0506077	0.15892507	0.00603317
-0.2521898	-0.1331713	0.16722196	0.18433008	0.0402569	-0.1087527	0.0417089
-0.6767751	-0.123246	-0.1822222	-0.576561	0.1849918	-0.3500889	-0.8778141
-2.7362618	0.24097076	0.04995403	0.03024422	-0.1835109	0.04946554	0.17161583
-0.2993778	0.2732844	-0.1554416	0.01493058	0.24376925	0.02351725	0.1830502
0.3436852	0.11538229	-0.4112872	-0.6602087	-0.121464	-0.0921381	-0.1197614
0.95663818	0.2351475	0.22749017	0.05599585	0.09248615	-0.0252674	-0.1733839
-0.0095216	0.32521096	-0.0454126	0.09552378	-0.0033858	-0.3035018	0.10268139
-0.0898504	0.48835373	0.45415716	0.16304979	-0.0356953	-0.1981595	-0.0442246
-0.0871563	-0.1534935	0.23305641	0.21810957	0.08432949	0.28836669	0.40322423
-0.7291434	-0.3004835	0.00694526	0.84005288	0.16485476	0.08883928	0.08233425
0.96805251	0.19956326	0.35649116	-1.6895008	0.30910946	-0.1956012	-0.223506
-0.1409133	0.26025787	-0.3935321	-0.704977	-0.0681651	-0.2651032	-0.3711171
0.57279572	-0.2169338	-0.4295608	-0.9073914	-0.1644664	-0.3765729	0.23269359
-1.0062012	0.13936895	-0.0405203	0.34546069	0.01492531	0.17973297	-0.2258019
0.01856097	0.05637037	0.3892263	1.2439087	0.2202843	-0.4073215	-0.4333604
0.37639829	-0.3765732	-0.55108	0.53539847	0.10436821	0.12380887	-0.1888897
-0.2742198	0.18487623	-0.1257207	-0.2551854	0.03348237	0.27070439	0.33695305
0.15392553	0.20087172	0.21320932	0.26529698	-0.0277958	-0.0551904	-0.2299968
0.0567752	-0.0825332	0.25580325	0.3385186	0.08433021	0.17894345	0.39658142
0.1863796	0.1237966	0.17581508	-0.0276777	-0.0006496	-0.3519797	-0.2103591
-0.0960848	0.0153357	-0.1884987	0.47124089	0.14234339	-0.3307619	-0.0926935
-2.1397135	0.51802543	0.14653324	0.31876729	0.076981	-0.4116576	0.65242917
0.70210885	-0.1164939	-0.0550609	-0.3125103	0.10338336	-0.1484565	0.49794172
-0.4013021	-0.1677597	0.08781092	0.75880087	0.03449446	0.09755808	-0.1950166
-0.8597738	0.21750675	0.07953755	0.72358983	0.10255331	0.08294003	0.039662
-0.6255084	0.18774593	0.37372937	0.06781251	0.20630705	0.03297543	0.17648437
-0.2593573	-0.3264105	0.88957478	0.31890591	0.18337703	-0.0276583	0.13277351
-0.0110476	0.48265214	1.011361	-0.0822714	-0.1252711	-0.0547732	0.02437213
0.22808549	0.31527207	0.7251934	2.0500856	0.04017688	0.07010431	-0.1593687
-0.4436063	0.24459802	0.05391438	0.59580872	0.04095744	-0.0741445	0.08684527
-0.25416	-0.026884	0.031354	0.4048	0.071334	-0.1430216	0.13515899
0.18771082	0.06050866	0.03832013	0.63895968	0.08102119	-0.291508	-0.0471173
-0.0134552	-0.0095568	-0.2597897	-0.0911953	-0.3826879	0.04940794	-0.2860207
-0.64208	0.030712	-0.10229	0.35862	-0.28016	-0.1308997	-0.2143493
-0.066461	-0.1458513	-0.363017	0.6256182	0.30815977	-0.490964	0.00107811
0.0068404	-0.1318426	-0.0400112	1.1094635	0.32366837	-0.6175828	0.23953959

-0.3069938	0.16874167	-0.0226885	-0.2211431	0.0576875	0.08971022	0.06558145
0.23708278	-0.1820725	0.38379124	0.56430953	-0.1559008	-0.2608155	-0.0100869
0.56908029	0.2043642	0.05736063	0.90485996	-0.0428473	0.02310092	0.48622122
0.29556473	0.0953149	0.46032418	0.85865046	-0.0260351	-0.1125308	-0.0891206
0.47697212	0.21681006	0.05273443	-0.0584702	0.10957218	-0.4191772	-0.0058928
-0.1784011	0.15894877	-0.0001564	0.46612333	0.10347852	-0.0974378	0.01255651
0.3564329	0.07105057	0.112574	0.18935178	0.0934011	-0.2422053	-0.1285439
-0.9844737	0.02343824	0.45391609	1.4164482	-0.1046127	-0.4031939	-0.2655828
-0.122971	0.38433738	0.33979738	0.5159297	0.04065455	-0.0859251	-0.0283786
0.048443	0.25813	0.21797	0.57053	0.17227	-0.1280784	0.08979943
-0.0598228	0.12002992	-0.1811089	0.15325841	-0.0418514	0.14503596	-0.0435856
-0.5734247	-0.0372106	-0.0710144	0.63384558	0.09370211	0.05947763	0.05266435
-0.1026823	0.26838985	0.53565281	0.00178396	-0.1057392	-0.2082604	0.06534313
0.09273324	0.03613215	0.11474066	0.81108074	0.25557924	0.06183767	-0.1812771
1.865137	0.2081363	0.22306245	-1.1777539	-0.0811247	0.11099606	0.47536331
0.09860324	0.34574222	0.45057602	0.14554552	-0.0470908	0.00962417	-0.0039362
-0.535546	0.2013084	0.52958559	0.69938465	0.10750903	-0.0192838	0.76352774
-0.5889174	0.02808972	-0.0609558	-0.0548114	0.13970394	0.00844522	0.37488886
-0.3208074	-0.0481658	0.14087066	0.20148342	0.05486032	-0.246217	0.21038048
-0.5703204	-0.1622032	0.08286639	0.20654311	0.0146616	0.01351541	0.11624663
-0.061207	0.05621161	0.0806866	1.292915	-0.1924045	0.01967584	-0.2707436
0.14583934	-0.3305588	-0.1110996	0.65817466	-0.0762531	0.26922721	-0.1314612
0.00370739	0.07087925	-0.2750594	-0.4461128	0.13308546	0.15509982	-0.3902587
-0.8999476	0.0527629	0.12887594	0.09765501	0.02968572	-0.1645457	0.33984894
-0.0431065	-0.086923	0.06938872	0.08420632	-0.0697903	0.08556253	0.0216797
-0.0258449	-0.0140093	-0.0959334	0.00095975	-0.2052124	0.22204157	0.39356088
-2.6237344	0.16623966	0.15221608	-0.0795214	0.17167783	-0.0954332	-0.1167477
-2.1181282	0.16901919	0.48146444	0.64856891	0.88738755	-0.1643608	0.08634439
-1.2161931	0.11022032	0.37732967	-0.131516	0.36184558	-0.2631084	0.41877957
-3.9605294	0.22988577	0.27244853	0.18685848	0.36018411	-0.175634	0.28908228
0.43088316	0.05377023	0.11089429	-0.0368551	0.26160647	0.02459609	-0.0689419
-1.2252639	0.3019976	0.26223354	1.1718234	0.15243886	0.27989682	-0.2652472
-4.4447801	0.19939953	0.2635508	0.0441602	0.56787585	-0.3358905	1.4314663
0.28600381	-0.2174671	-0.9336826	-0.2895548	-0.5690458	-0.2125624	0.09925885
-0.7526142	0.19287313	0.39762683	0.632709	0.03653825	-0.1534097	0.26462494
0.0021211	0.15420503	-0.1392584	-0.1946065	0.55149151	0.25942631	0.26346638
-0.2385575	-0.1286326	0.16383099	0.05305234	0.11641257	0.15068461	-0.2117485
0.09882805	-0.1876518	0.64564806	0.13952413	0.17351992	-0.1708895	0.06837781
-2.1547224	0.04406023	0.42168674	-1.6426581	0.01754262	-0.5193122	-0.1193013
0.78694449	0.09909426	-0.0469882	-0.2062818	0.08517569	-0.5626694	-0.0909545
0.0472453	0.17416455	0.02589306	-0.1635959	0.3634308	0.29454193	0.14983642
-0.6901142	0.23192663	0.11065007	0.11586066	-0.0001477	-0.354662	-0.0934018

0.05556516	0.05430172	0.0767297	0.07290803	-0.0095044	0.11219517	-0.042772
-0.034565	0.043285	-0.062665	1.124	-0.058113	0.10279	-0.15201
-0.1433247	0.2006108	0.22498526	0.21363193	0.17912178	0.21288314	-0.0333456
-0.0935166	-0.0214603	0.15713972	-0.2107581	-0.1078486	-0.1449752	0.15972393
0.12564742	-0.0728595	0.45889499	-0.2095521	-0.0706791	0.10315492	0.35855306
-0.5010758	0.30149885	0.11544711	-0.3337833	-0.2632433	-0.1828655	0.07899791
0.13267177	0.29147032	0.47418338	1.1017719	-0.1850167	-0.2120001	0.11643316
0.098931	0.40902	0.58626	0.7582	0.017729	-0.28769	0.056155
-0.2807513	0.33130069	0.03945326	-0.0779684	-0.0152238	-0.2119972	-0.43005
0.94847	0.062272	0.040382	-0.78071	0.38087	-0.1080622	0.12234619
0.39288779	-0.2743926	-0.1271304	0.72632269	-0.7469948	0.06815303	-0.0111084
-0.8736026	0.27607943	0.50590592	0.46170508	-0.1606641	-0.1050942	0.24098016
-0.9917246	0.19022719	0.0708639	0.43142824	0.15398515	-0.3859614	-0.3420723
-0.1617286	-0.0941817	-0.2595904	0.05313652	-0.1567276	0.12542387	-0.0885158
-0.0812505	-0.212454	-0.1536838	0.44864064	0.08926957	0.11505366	0.03736792
-0.6773342	-0.1177557	-0.0564078	0.30350472	0.05748887	-0.1133059	0.33945162
-0.6245194	-0.0767112	0.11686294	-0.0449267	0.13665393	-0.0808156	0.12207338
-0.7554217	0.06291665	0.18463531	0.03499336	0.02732759	0.01652548	-0.0357
0.80771196	0.41581342	0.35654887	-0.1542367	-0.0625021	-0.3794627	0.00716138
-0.8746436	0.21548073	0.20251821	0.4213022	-0.0342609	-0.0419361	-0.2517809
-0.1239702	-0.0813671	-0.2510015	0.16682858	-0.0453726	-0.1548653	-1.1305804
-0.4896054	0.19108352	-0.0016237	-0.0102072	-0.0080918	-0.164556	0.30161584
0.84316	-0.16654	-0.075368	-0.12455	-0.11998	-0.0459185	-0.4108203
-0.308229	0.06416219	0.17060001	0.35822412	0.19087572	-0.3891163	0.65026324
0.15680756	0.07114867	-0.1229462	0.93944913	-0.0851613	-0.014129	-0.2495842
-0.1397355	0.21433445	-0.1965584	0.26995439	-0.0639158	-0.121271	-0.0077772
0.18071973	-0.1444414	0.04112424	0.35765549	-0.2572528	-0.1810888	-0.1169416
0.39124	-0.24294	0.28249	1.6355	0.22328	-0.2402966	-0.3325132
-0.2651386	0.26454625	0.52194613	0.24059336	-0.0498453	0.12453701	0.62104058
0.04004227	0.03991523	0.04351816	0.24378536	0.27259272	-0.2243505	0.17797774
0.17265752	0.32217676	0.3033717	0.84180125	0.01607423	-0.1210836	-0.1030876
0.03586787	-0.0179462	0.194994	-0.1748511	-0.0772266	0.07082823	0.12914077
-0.27128	-0.046443	0.42876	0.77709	0.32285	-0.5378427	-0.918276
-0.76168	-0.1777747	0.29427785	1.4434514	0.40470379	0.29300249	-0.2248466
-1.3384913	0.00922014	0.52300567	1.7040803	0.32544611	-0.0495929	-0.7041326
-1.0484339	-0.21337	-0.0912719	0.37193054	-0.0623907	0.25689201	0.07417921
0.30428131	0.29335271	0.99280677	0.33659335	-0.1221866	-0.0021029	0.02023936
-1.896599	-0.0264048	0.25686603	0.07269126	0.33947033	-0.1717224	0.31414247
-0.1445561	0.01601666	0.06977144	0.30752112	0.00970915	0.2003076	0.76279214
-3.4107356	-0.2604554	0.75985876	0.079243	0.15686121	-0.2551995	-0.1443606
0.59705534	0.16182301	0.12158705	0.57782599	0.07738797	-0.3648238	-0.2867139
0.01608652	-0.0148707	-0.1564093	0.14972609	0.16245717	0.16570315	0.35665283

-0.5259695	-0.1358313	-0.0420801	1.2270712	0.24414032	-0.2901411	0.33033293
-0.8061095	0.13556898	0.24049111	0.61421457	-0.0494572	0.35184565	0.23545655
-0.4525475	0.17209211	-0.0920637	0.41117395	0.02660742	0.21251344	0.26087242
-0.5668678	0.12340512	0.15293153	0.48279371	0.18767305	0.62278575	0.2990765
-0.0192901	0.18537464	0.24566935	0.1369243	0.28154152	0.18940628	-0.1419384
0.08993829	0.1029386	0.03246253	0.4250962	0.12821321	-0.1393408	0.30579527
0.08062063	0.34228698	0.14233671	0.31754366	-0.0166089	-0.6192121	0.23290026
0.04847529	-0.1963525	-0.0023728	0.25349672	0.12316582	-0.1039345	-0.1427275
-0.0429547	-0.1345476	-0.0602355	0.42817043	0.01321576	0.00026185	0.20233781
0.15436	-0.15866	0.0076975	0.35035	0.016141	0.17431537	0.56254024
-0.7676699	-0.0141667	-0.6535606	0.15631063	0.05993827	0.18753633	-0.1993079
-1.5956424	-0.1853875	0.44832823	1.1139233	-0.0728822	0.05477499	0.05431413
-0.1499136	0.22201669	0.07845924	0.11148315	-0.0324523	-0.0900953	0.01165198
-0.37042	0.25703	0.24151	0.060361	0.032957	0.01575042	0.10493637
-0.1035292	0.08628487	0.20674101	0.6034174	0.02908066	0.09865001	-0.125954
-0.1995544	-0.1277972	-0.0477905	0.32215654	-0.0239689	0.08131516	-0.0272464
-0.5127733	0.11073343	0.14086772	-0.1472471	0.09873614	-0.1828216	0.15898691
-0.8514219	0.0471728	-0.0437939	0.35361097	0.13842256	-0.0262083	-0.3183976
-0.904584	-0.016026	-0.4517207	-0.1906663	0.10188218	-0.0481441	0.15997822
-1.0513897	0.28357864	0.26616414	0.38398129	0.2489462	0.1482851	0.90633417

test_GSE218	test_GSE218	test_GSE218	test_GSE218	test_GSE218	test_GSE218	ref_GSE2841
ref_GSE2186	ref_GSE2186	ref_GSE2186	ref_GSE2186	ref_GSE2186	ref_GSE2186	ref_GSE2841
GSE21869	GSE21869	GSE21869	GSE21869	GSE21869	GSE21869	GSE28412
GEO	GEO	GEO	GEO	GEO	GEO	GEO
affymetrix	affymetrix	affymetrix	affymetrix	affymetrix	affymetrix	affymetrix
PaeR7I M:+1	EVO_PaeR7II	EVO_PaeR7II	EVO_PaeR7II	EVO_PaeR7II	EVO_PaeR7II	GROWTH_RA
2075	2088	2089	2090	2093	2094	2105
0.83535291	-0.2225052	-0.0085718	0.13815896	0.04460614	-0.0536999	0.15639274
-0.0902604	0.3739357	0.244536	0.07150206	-0.0877707	0.31453443	-0.2647106
-0.3381392	-1.7122758	-1.906681	-1.9345734	-2.0307274	-1.8961937	0.07437072
0.0726366	-0.0524389	-0.0224059	-0.0266379	-0.1070382	-0.3750995	-0.0600948
0.17475676	-0.1454685	-0.0005792	0.06000785	0.00284866	-0.1170795	0.05658798
0.50769619	-0.441551	-0.4231986	-0.4426821	-0.5469635	-0.6735485	-0.0286588
0.10849702	1.201975	1.6160752	1.4182014	2.033695	2.2566934	0.02862273
0.06074133	-3.1690491	-3.1291136	-3.2509299	-3.8211357	-3.8399172	0.2024372
-0.0089835	-0.6786647	-0.7157418	-0.60153	-0.6975844	-0.7136782	-0.0335697
0.14290756	-0.5270604	-0.4702645	-0.5655657	-0.5751873	-0.8235495	0.12353702
0.95339869	-0.0124205	0.14429546	0.01660451	0.16958867	0.03709339	0.15201713
0.01494075	-1.5339036	-1.6630459	-1.7921679	-1.9048345	-1.8014209	-0.4120903
0.02465334	-0.0191792	0.24128146	0.05661591	-0.101888	-0.2331644	0.15516003
0.26644101	0.03416161	0.13555736	0.34266886	0.25544961	0.21073439	-0.2346122
0.06947594	0.03225143	0.04159334	0.21360198	0.11792785	0.06611995	0.28558674
0.32101761	-0.4998856	-0.2972449	-0.4087482	-0.2138339	-0.3433201	0.19782828
0.66691728	-0.1554134	-0.1415329	0.08509514	0.18428388	0.30586446	0.29024
0.74586001	0.893061	0.83294374	0.57773017	1.3232885	1.4034609	0.03972276
0.3688123	0.29017285	0.16696415	0.04418887	0.23790244	0.21246476	0.23787932
0.06732013	-2.3575451	-2.3820839	-2.0704083	-2.8113199	-2.7439997	-0.3021643
0.0482622	0.31683263	0.57674212	0.397206	0.46996546	0.51229604	-0.121274
0.36179887	0.09448239	0.18687742	0.16528018	0.42881396	0.17022989	-0.35978
0.0383527	-2.0802189	-2.3169711	-2.3230148	-2.3542694	-2.5962131	-0.1947361
0.26024646	-0.7088751	-0.8064804	-0.6874254	-0.8381483	-1.0683498	-0.24502
-0.0092327	-0.3503949	-0.3325533	-0.0916565	-0.036183	-0.1312918	0.01935697
-0.2365891	0.26521834	0.49135162	0.51816714	0.38005413	0.15773177	-0.2235204
0.69773127	-1.573699	-1.4815325	-1.6540152	-1.5475654	-1.5615803	-0.17105
0.61507617	0.04311452	-0.096825	-0.2044519	-1.1729553	-0.9217142	-0.0648151
0.24887495	0.23477013	0.42407164	0.24420789	0.10305094	0.02625937	-0.31439
0.83364032	0.70786175	0.83994683	0.76644765	0.73183504	0.66262924	-0.0407013
0.53836151	0.4978215	0.57128312	0.32357044	0.56703645	0.5917696	0.48850782
0.1362953	-0.2542124	-0.0761714	-0.0258659	0.04859556	-0.1622957	0.02958555

0.12405625	-0.4435455	-0.2144687	-0.1769736	-0.2505412	-0.2743256	0.0344786
0.05176287	-0.0176411	-0.1661247	-0.1390421	0.04995271	-0.0676212	0.33653437
0.12887836	0.00284896	-0.0562118	-0.1552081	0.37829111	0.2524417	0.21864901
0.07149671	0.00951421	0.03509979	0.04942274	0.04701508	0.04601508	0.0229225
-0.042776	-0.2753887	-0.3452823	-0.2307683	-0.032342	-0.079525	0.25777103
0.11201066	-0.586562	-0.6020142	-0.6079901	-0.3798227	-0.1706093	0.39009263
0.11179354	0.04203278	0.12202316	0.16820708	0.07255329	0.12429485	0.11383073
-0.0123896	-0.0520974	-0.0863897	-0.0043407	-0.058077	-0.1081462	-0.1211695
0.19096314	-0.0815293	0.18582072	0.21077976	0.3527914	0.37663866	0.05215549
0.07948391	-0.0815849	0.09732247	-0.0202705	0.08354711	0.0053521	-0.0309897
0.21520289	0.24062908	0.35588866	0.31020023	0.41050009	0.3289999	-0.3421803
1.0399621	0.6496732	0.76608136	0.44669275	0.21083867	0.15547083	-0.0613887
-0.0241478	0.15990972	0.11488951	0.06184025	0.13238362	0.18731586	0.36116159
-0.2484895	0.13247452	0.09628124	0.13727489	-0.0162617	0.1810499	-0.603974
0.32473811	0.01391344	0.43328003	0.43506074	0.04980499	0.13800234	-0.0802146
0.34407078	0.93358556	1.1799177	1.2187366	1.6821798	1.5903459	0.01003223
0.2961383	-0.0008813	0.07429399	0.35158315	0.32613899	0.13996386	0.03459869
0.27009025	0.04689478	0.51410522	0.447483	0.61053668	0.50295808	0.45876767
-0.034212	0.09025825	0.10573807	0.10299978	0.04600849	0.27937607	-0.0233939
0.18587077	0.0331109	0.23818618	0.24052305	0.20259907	0.20188682	0.36202599
-0.2980043	-0.7662041	-0.7346656	-0.8041632	-1.1792188	-1.0229685	-0.2030557
0.29943362	-0.6598165	-0.6667379	-0.6776172	-0.4733664	-0.6437133	-0.1705598
0.51574634	0.46543926	0.40965641	0.67221551	0.56950594	0.61690987	-0.3297419
0.18180298	0.63521612	1.0603056	0.80466047	1.0408634	1.0470013	-0.0131902
0.21688155	-0.2406645	-0.1218384	-0.0390394	-0.1814337	-0.3275705	0.15381303
0.57546894	-2.2587678	-1.7615626	-1.9578387	-2.9959107	-3.0687864	0.15121397
0.49643543	-0.0765613	-0.1081093	0.01486041	-0.0951973	-0.0929491	0.01633196
-0.0108077	-0.0748681	-0.0689116	-0.0605419	-0.4435869	-0.0966447	0.36785735
0.81664983	-0.9190765	-0.6236156	-0.5164557	-0.770005	-0.8707321	0.05181034
-0.2188979	-0.0521706	0.12261185	0.00684031	-0.0511663	0.03309757	0.4292432
0.097688	-0.1246828	0.19585513	0.02383349	-0.0413687	0.03533021	0.4574914
0.14042438	0.40527715	0.46262823	0.32865255	0.40326611	0.34841636	0.00449893
-0.51311	-0.380717	-0.4287819	-0.523362	-0.7077006	-0.5555667	-0.2679797
-0.8810214	-1.9629064	-2.2600255	-2.3177503	-2.7520908	-2.5999461	-0.1576835
0.12621424	-0.2831718	-0.2792923	-0.2502249	-0.1462428	-0.2417796	0.17981718
0.04552354	-0.9039756	-0.8542329	-0.9271487	-0.5972939	-0.7810924	0.29176369
0.1002911	-0.8372063	-0.8231686	-0.5839624	-0.9140316	-0.8883121	-0.107178
-0.1691305	-0.5982495	-0.6150539	-0.389134	-0.470124	-0.5143717	-0.2419568
0.04596802	-0.5467774	-0.6189766	-0.5399826	-0.3152641	-0.3374082	0.66903637
-0.1876804	0.19573014	0.15133803	-0.0131807	0.26682853	0.17772059	-0.2013303
-0.2275099	-0.4996621	-0.448149	-0.466899	-0.4923045	-0.3937469	-0.1479573
0.09974377	-0.1348175	-0.0699084	-0.082703	-0.1011866	-0.1748802	-0.1193876

0.37596101	-0.43054	-0.4137332	-0.5406441	-1.2585451	-1.2103266	0.0295485
-0.1503931	-0.0488606	-0.012569	-0.2604345	0.01404823	0.03018587	0.42605915
0.0196524	0.05414925	0.03132854	-0.1113878	-0.0301972	-0.0684091	0.30725013
-0.2934998	-0.7488645	-0.7248929	-0.7044618	-0.6820836	-0.7422816	0.29481164
-0.1781553	-0.5583662	-0.5803327	-0.4965963	-0.2696315	-0.2966071	0.50661482
0.38290642	-0.5008557	-0.5135018	-0.5936119	-0.2006189	-0.0984918	-0.5256518
-0.1379108	0.2451612	0.34489239	0.13546772	0.37792797	0.33951383	0.14141103
0.3317836	-0.03386	0.31209963	0.23769452	0.21063297	0.28251617	-0.4969372
0.06201349	0.00745227	0.10832597	-0.1160658	0.33073335	0.26193562	-0.2213058
0.13499183	-0.1258051	-0.0541385	0.1308773	0.06322824	0.12786298	0.0777981
-0.1616463	-0.2889773	-0.3029259	-0.299076	-0.1723016	-0.2291478	0.1692241
0.14506611	-0.2089704	-0.0709497	-0.0629467	-0.1335779	-0.1495303	0.09283356
0.33311672	-0.5307291	-0.6541155	-0.6103185	-1.0769249	-0.7775811	0.45606406
0.26770329	-0.8524011	-0.5139337	-0.5659933	-0.526959	-0.4540974	0.00810093
0.53454349	0.32463884	0.24566874	-0.0324586	0.59759295	0.76481787	-0.10323
-0.0832004	-0.1995375	0.14698701	0.00344629	0.14180928	0.37770537	-0.1619015
-0.3358596	-0.3381671	-0.3326461	-0.2796796	-0.3144717	-0.3304605	-0.095037
-0.3052056	-0.2105808	-0.0705764	-0.2198881	-0.0920188	-0.1445962	0.22399807
0.26602409	-0.9545747	-0.900677	-0.8212264	-0.990212	-1.0418333	0.16891
-0.1975227	-0.2155501	-0.2140909	-0.3824482	-0.4413698	-0.5663413	0.0207276
-0.2466408	-0.1530201	-0.131047	-0.0810812	-0.2428661	-0.2124113	0.40407304
-0.0061075	-0.3048953	-0.0897317	-0.134373	-0.1297569	-0.1963519	-0.13289
0.08549928	-0.8388849	-0.7416651	-0.8212177	-0.7445671	-0.8361372	-0.0346555
-0.1855106	-2.2441355	-2.2110419	-2.0793346	-2.6079359	-2.6054194	-0.0596929
0.39181083	0.22876948	0.21265827	0.22217372	0.29559528	0.4988985	0.15009694
-0.1672153	0.0675396	-0.0249695	0.01205472	-0.168557	-0.0408475	-0.3247996
-0.2545769	0.0240559	0.00859227	-0.1878706	-0.5307678	-0.0038879	0.00310592
0.03015336	0.60914209	0.64388488	0.45824726	0.73798309	0.67823628	0.19485999
-0.19446	-0.10205	-0.13138	-0.15107	-0.3725	-0.24002	0.49255
0.023429	-0.17242	-0.069737	-0.15247	0.005411	-0.026528	-0.0068896
-0.26054	0.35676	0.3132	0.24555	0.4565	0.51483	0.24604
0.37504	0.13327	0.23025	0.047928	0.084653	0.27942	0.10525
0.59518	0.15927	0.27934	-0.056603	0.1321	0.15545	0.064426
0.0013661	-0.12105	-0.13272	0.066838	-0.19217	-0.26407	0.12412
0.60737	0.54573	0.68833	0.67071	-0.081573	-0.0083615	-0.065723
-0.125543	-0.6872153	-0.5970861	-0.7361997	-0.5200682	-0.7192123	-0.0544087
0.05187577	-0.1989394	-0.2500351	-0.263818	-0.2636471	-0.2183504	-0.5778255
0.22249739	0.64277815	0.53353803	0.682329	0.41442008	0.49107825	0.23477037
0.06661907	0.30755859	0.26663684	0.02980942	-0.0476104	-0.1103931	0.16558748
-0.1600583	-0.3270522	-0.3277891	-0.2777228	-0.1424132	-0.3204414	-0.3355544
0.03980442	0.1655486	0.5320823	0.28992366	0.23244106	0.42773061	-0.2265825
0.69256392	0.07764391	0.07572605	0.18833943	-0.9357534	-0.4888896	0.16253061

-0.1756372	-0.1894118	-0.1996132	-0.220536	-0.5719809	-0.3481021	-0.7264532
0.09639444	0.8474526	1.0623436	0.90888489	1.2853792	1.3667624	-0.3014961
0.05170848	-0.1440474	0.05589423	-0.0664523	-0.0575296	-0.0320414	-0.0629994
0.13543464	-0.3758599	-0.1857789	-0.2721072	-0.2257104	-0.1933439	0.0224771
0.28089919	-0.8061708	-0.6447005	-0.4092272	-0.5800485	-0.6241853	0.51795033
0.58681889	-0.6041614	-0.5080027	-0.9031405	-0.6950811	-0.7303866	0.1882628
-0.0264708	-0.2550605	-0.0989282	-0.1169393	0.02469673	-0.053198	-0.0596792
0.0819047	0.32648378	0.46063101	0.2252448	0.25595019	0.27933042	-0.0338279
0.20801603	-0.0552373	-0.1395471	-0.1989657	-0.6341735	-0.3811241	-0.0288717
-0.1814133	-1.4820823	-1.5276401	-1.486328	-2.2267303	-2.1965568	-0.0666999
0.7100195	0.19142536	0.15192465	0.21638496	0.19481142	0.16217292	-0.1383174
0.14173556	0.47958496	0.30256061	0.29028597	0.1097257	0.32829538	0.26663836
-0.4669278	0.47326346	0.37560165	0.34831138	0.19798294	0.10417697	-0.0400862
0.46404555	-2.4795724	-2.4522625	-2.1761815	-2.3522813	-2.4521216	0.09964107
0.28174444	-3.645701	-3.6335137	-3.5114656	-3.5630484	-3.5433408	0.19659797
0.04916881	0.17555336	0.36335023	0.32196118	0.04552751	0.22631814	-0.0366151
-0.0018551	0.05909847	0.20643771	0.02253138	-0.1063208	0.03899721	-0.1076812
-0.0676357	-0.1385167	-0.146033	-0.0688666	-0.1800994	-0.2688666	0.23313432
0.0617546	0.58034068	0.69511126	0.38906984	0.46647149	0.43922504	-0.2109525
-0.3488743	-0.2333432	-0.2628516	-0.2909851	-0.4181525	-0.3949119	-0.0694271
-0.0245443	-0.2992047	-0.2560202	-0.305062	-0.1301523	-0.0770842	-0.2214889
0.2757964	-0.4109771	-0.4702033	-0.4065136	-0.1918336	-0.3108272	-0.2788875
0.0057005	-0.1057	-0.26084	-0.53178	0.090372	0.086901	-0.05148
0.29078773	-0.1833267	-0.0387745	-0.0618209	-0.2248128	-0.2897105	0.10604459
0.16632536	-0.3647108	-0.3257485	-0.2277964	-0.1711095	-0.2465486	0.16278
0.73469698	-0.0105283	0.15914391	-0.0646987	-0.001172	-0.0183574	-0.2338561
-0.0617841	0.04203926	0.15048469	0.16137605	0.04506431	0.273992	-0.0869764
0.14105839	-0.0501993	-0.0165914	0.10784433	-0.0189901	-0.3980691	0.10405943
0.2961993	0.62704353	0.69642675	0.422187	0.7897681	0.69009267	0.37887649
0.16758473	-0.8802602	-0.8516927	-0.9705902	-0.8031753	-0.8450955	0.047571
0.09727615	-0.3750363	-0.5669851	-0.4970618	-0.8475033	-1.0543535	-0.0105942
-0.3130058	-0.2862553	-0.3275342	-0.5102556	-0.264943	-0.2375152	-0.1006377
-0.0539916	-0.0269247	-0.0230258	-0.2189643	0.14578354	0.14327208	-0.1693457
0.1018026	-0.1474909	-0.133237	-0.1877056	0.00757116	-0.0491281	0.25492768
0.01873968	0.42377469	0.69769327	0.635668	0.5419607	0.66073739	0.30229532
-0.1939361	-0.2457808	-0.2681278	-0.0759537	-0.3429911	-0.2564444	-0.1039953
0.12610258	-0.0072501	0.14336176	0.28357922	-0.2118489	-0.1767621	0.02850406
-0.3103548	-0.7784034	-0.6853668	-0.7872517	-0.6230481	-0.8355695	0.09389916
-0.0582708	-0.5007579	-0.4568127	-0.344753	-0.5753901	-0.5985753	0.10340645
0.23744891	0.15758418	0.33753654	0.05264413	0.05269874	-0.0815218	0.40120959
0.05958289	0.03097201	-0.1898169	-0.1864151	-0.1198878	-0.2880286	0.239803
0.09247984	0.79545822	0.9165981	0.63941296	1.2358053	1.3448746	0.64034077

0.1959207	-0.1222816	-0.0193035	-0.0647564	0.09753667	0.14910207	0.01731456
0.1022118	0.09515833	0.17360714	-0.0699074	-0.0193953	0.04332953	0.13102631
-0.0622835	-0.0768971	-0.0221113	0.04649001	-0.042301	-0.1176946	0.03468613
0.03945229	-0.0485338	-0.1307836	-0.1495254	0.07416429	-0.1096865	0.03344311
-0.0944732	-0.1446802	-0.1902152	-0.1768834	-0.3647594	-0.1860361	-0.351492
-0.0277124	0.19313029	0.13227903	0.19795885	0.29699636	0.33354668	-0.1539969
-0.1346547	-0.0292995	-0.08837	-0.0788971	-0.2319565	-0.2879546	-0.0041241
-0.5027753	-0.6258943	-0.6527012	-0.572978	-0.4914952	-0.4821482	-0.019218
0.56516314	-0.9050273	-0.8572	-0.9515627	-0.8143657	-1.0426221	-0.2336126
0.1851743	-0.0545089	-0.0724816	-0.1725833	-0.0903689	-0.1079978	-0.4128934
-0.2806485	0.13159739	0.0159201	0.17075754	0.28603222	0.04643614	-0.2217077
-0.1059738	0.56191556	0.57303393	0.47437296	0.62553757	0.53874233	0.3316476
-0.0489693	-0.0427696	-0.0488659	-0.0582261	-0.2303625	-0.1927525	-0.0561109
-0.0886299	-0.1398472	-0.1017823	-0.0786095	-0.3461948	-0.1798462	-0.1985473
0.37173219	-1.4623739	-1.501558	-1.2549115	-1.3014314	-1.3622917	0.36226544
0.160148	-0.0559976	-0.1403881	-0.0650823	-0.1052492	-0.0842462	0.54643701
-0.0333682	1.5018799	1.2477497	1.3240905	1.072218	0.87214037	-0.087822
-0.3589329	1.4733419	1.3542189	1.3393807	1.305958	1.3897924	0.04529049
-0.0427871	0.37892472	0.22818338	0.05655048	0.12102267	-0.0358747	-0.0100969
-0.2915019	-0.5634128	-0.7305557	-0.7530513	-0.8442149	-0.9007526	0.00760692
-0.3925065	-0.4382909	-0.6116831	-0.7042383	-0.6510637	-0.8885792	0.17618209
-0.125637	-0.1234965	-0.1146254	-0.1344386	-0.1098718	-0.1338242	-0.3540704
0.39570059	0.38741269	0.31655609	0.42398656	0.49287439	0.35850145	0.03326526
-0.1318951	-0.0449324	-0.1339463	-0.047177	0.03934738	0.0062163	0.12734625
0.50055444	0.22236537	0.19761119	0.15934079	0.49084973	0.57423975	0.19819193
-0.1159008	-0.1891003	-0.1948932	-0.19935	-0.4986208	-0.3620124	0.0755772
-0.236897	-0.295219	-0.4633276	-0.5401559	-0.7280357	-0.7498062	-0.1676577
0.39496445	-0.7922211	-1.0048715	-0.8208722	-1.6788176	-1.6507635	0.10367428
0.23879813	0.11627485	0.15864396	0.07890534	-0.0967127	0.26509383	-0.128722
-0.033466	-0.3389227	-0.3708086	-0.3651948	-0.1481986	-0.3171083	0.11350077
0.34232163	0.23465169	0.28817203	0.24057248	0.43110928	0.42774216	0.17387419
0.00909469	0.13957953	0.09335187	0.05098385	-0.0445358	-0.137466	0.16293094
0.10831311	0.08168479	0.12847481	0.24879686	0.0704207	0.27549383	0.05486931
-0.1828236	0.10106373	0.09625096	0.1429594	0.06037519	0.08521375	0.09255717
0.12270429	-1.8020625	-1.8734594	-1.801246	-1.9398442	-2.1803365	0.52889972
0.04472645	0.15322043	0.13500132	-0.0645337	0.33126522	0.19272314	0.21507137
-0.0222538	0.14615794	0.08874031	0.22682941	-0.0230732	-0.0282274	-0.090497
-0.1241647	-0.0692805	-0.1376842	-0.2548296	-0.0522715	-0.1203774	-0.0069757
0.01658125	-1.5399045	-1.7359395	-1.6551012	-1.7326663	-1.6966927	0.10021888
-0.1842469	-0.3759593	-0.4092838	-0.3991287	-0.3428315	-0.3764543	-0.26896
-0.0140537	-0.2487917	-0.3258771	-0.719257	-0.406314	-0.4465287	0.15107125
0.13578237	-0.2128827	-0.2489649	-0.6209929	-0.5649792	-0.6629381	0.5408218

0.05342162	0.39251288	0.28078808	0.21265145	0.28488197	0.37074338	-0.1493324
-0.033133	-0.0180882	-0.1007267	-0.0592083	0.07967099	0.12144019	-0.1447249
0.47210622	0.56050022	0.4139978	0.62366759	0.3133812	0.41321226	0.6941695
0.01080832	0.32864195	0.25273699	0.33727068	0.16806866	0.46187931	0.55704149
-0.1124166	-0.3463229	-0.3924856	-0.4471379	-0.6480295	-0.5400938	0.06396298
-0.0377685	-0.2848398	-0.3501243	-0.4015845	-0.4697117	-0.505717	0.03992835
-0.1001069	0.57382033	0.58103005	0.31573853	0.47349537	0.63579915	-0.043949
-0.0773656	-0.5840573	-0.6926407	-0.8497464	-0.7893917	-0.8186136	0.15870386
-0.0071222	-0.122568	-0.1378295	-0.0500073	-0.0021256	-0.0045052	0.58223866
0.05163331	0.07726941	0.0571659	-0.1837992	-0.0074018	-0.1600762	0.31634
0.06236232	0.42408328	0.36981636	0.12736209	0.25659897	0.39078777	-0.1340528
0.17222614	0.09042538	0.09340015	0.08461619	-0.2378143	-0.0118149	-0.0113768
0.20613942	0.42487727	0.55746366	0.3436189	0.1937314	0.27940845	0.17445047
-0.0710528	0.00696643	-0.1510369	-0.1267982	0.18954852	0.17851996	-0.018027
0.41896016	2.9863875	2.8570236	2.6677932	2.8939228	2.7825254	0.4117532
-0.0182463	0.15001915	0.18583409	0.0775741	0.20263423	0.22085214	0.11630041
0.83887724	-0.6106799	-0.6381951	-0.7160013	-0.5244183	-0.3799249	0.13740368
0.24345345	-0.0664247	0.04733557	-0.109958	-0.1662296	-0.2480523	0.46882476
0.13488916	-0.3799736	-0.4450564	-0.3279715	-0.4692359	-0.4707251	0.35513104
0.2849275	-0.2906645	-0.474522	-0.5010789	-0.8017765	-0.7394485	-0.0946858
-0.2475612	-1.5619298	-1.674372	-1.5805715	-1.7868063	-1.8156329	-0.1706083
-0.0329197	0.15120882	0.01126306	0.06698001	0.14435615	0.07953161	-0.2191917
-0.1455868	0.31838238	0.26820403	0.33480079	0.61136015	0.50213592	0.09562911
0.37085221	0.22890297	0.13473404	0.19434049	0.67006685	0.4035311	0.04496389
-0.089159	0.04016562	0.0420408	-0.1547668	-0.3124199	-0.3781806	0.26993984
0.5798914	0.6028374	0.49125347	0.5036398	0.90290837	0.82839246	-0.5411891
-0.0854995	-0.0179122	-0.0258796	0.03390627	-0.0284454	-0.0877124	0.46951784
0.08419149	-0.6408197	-0.675749	-0.6805098	-0.5478396	-0.712801	0.32586264
-0.0489435	0.54155359	0.6134816	0.60523822	0.79388195	0.6343128	0.30075332
-0.1236159	0.08056488	0.13023747	-0.0193724	0.03189131	-0.0967784	0.29513581
-0.1763048	0.07855448	0.05707552	-0.1198745	-0.0771796	-0.0844936	0.07392446
-0.1135379	0.08284524	-0.1389976	-0.0329488	0.05753065	0.00074702	0.02453967
0.90127711	-2.622965	-2.8087663	-2.5270839	-2.9074354	-3.137767	0.3369571
0.19835428	0.88222222	0.94661493	0.70597737	1.0649922	0.90939293	0.16261819
0.24438713	-0.4371298	-0.3417099	-0.3702246	-0.0825398	-0.1962182	0.40725318
0.15632501	0.57556728	0.583967	0.31807453	0.0822665	0.50539107	-0.0466662
-0.1381185	0.16581384	0.03545068	-0.0642912	-0.0502476	0.08184821	0.19575017
0.08475635	0.41339164	0.32450223	-0.1066544	0.68205054	0.69542751	-0.2792404
-0.018653	-1.4548839	-1.82256	-1.4989262	-1.7442282	-1.6428596	-0.0901733
-0.2152278	0.9608776	1.0512633	1.1856068	0.96758079	0.97607596	-0.043228
0.15052294	0.552537	0.42849459	0.51679824	0.75397016	0.59160559	0.06826557
-0.1746907	-0.3354619	-0.4419872	-0.2883278	-0.4428864	-0.5267362	0.18439917

-0.1549303	0.14554685	0.02407902	-0.028581	-0.0588243	0.06800524	-0.0380184
-0.1398	-0.10375	-0.22965	-0.28029	-0.43529	-0.51101	0.11455
-0.2401811	0.53380957	0.49975243	0.50134766	0.71248625	0.64382884	-0.0311764
0.24721973	0.37801592	0.37129427	0.2881544	0.81099087	0.94448425	0.09867234
0.29380611	0.81356073	0.79362412	0.59148684	0.84793769	0.84666261	0.16943317
0.0772874	0.13014065	0.10902183	0.10843082	-0.1057389	0.01478467	-0.0918439
0.13730447	-0.0862547	-0.0490981	-0.1930429	0.00802228	0.0577134	0.18550024
-0.20177	-0.59614	-0.61612	-0.76414	-0.7635	-0.75963	0.66391
-0.4250396	-0.5288101	-0.6789682	-0.3649483	-0.771295	-0.665278	-0.7587382
0.22894046	0.18814446	0.00364893	0.17756348	0.38626098	0.42669641	-0.11679
-0.0569309	0.26396973	0.17915101	0.08384562	0.05516276	0.17449446	-0.2517203
0.31008788	0.32918762	0.28943194	0.20443613	0.50988375	0.41744104	0.05897312
-0.2937261	-0.571428	-0.5822849	-0.6645156	-0.8525362	-0.8784041	-0.0547193
-0.0652339	-0.0802931	-0.1713777	-0.082442	-0.0434681	-0.16311	0.02778894
0.14408535	-0.0822321	-0.1750282	-0.1226146	0.02825704	0.09119184	0.33843724
0.30182536	-0.0646094	-0.1328381	-0.0458746	0.35766646	0.30263739	0.20073991
0.29506405	-0.3540095	-0.13148	0.00335432	-0.2388713	-0.2522244	0.02353008
-0.0194073	0.56538644	0.37107643	0.4359438	0.58310783	0.52752716	0.24047757
-0.1558213	0.9219515	0.82611381	0.71041524	0.71430642	0.64862229	0.07097203
-0.194095	-0.2830359	-0.3631517	-0.3870364	-0.5663165	-0.2618474	0.05565899
-0.6413506	0.00294298	-0.2473691	-0.1943582	-0.2116553	-0.3321748	0.06948671
0.28575744	-0.4791871	-0.6680928	-0.703362	-1.0651655	-1.0719601	-0.0556873
-0.4329334	0.65009962	0.46527304	0.27923366	0.87748	0.51343088	0.077246
0.54421459	-0.5956977	-0.7168107	-0.7941728	-1.3349971	-1.3683674	0.08797013
-0.2575209	-0.2121899	-0.207854	-0.01593	-0.2895282	-0.3156765	0.02674443
-0.0549839	0.37951386	0.23293118	0.29426084	0.54424963	0.49508986	0.08117869
-0.1389087	0.02169634	0.13245953	-0.2606685	-0.3176848	-0.2180789	-0.1013988
-0.1696132	0.02077593	-0.0691039	-0.1544884	-0.1403815	-0.1676657	0.76592
0.62114027	0.3774384	0.33952038	0.148317	0.59790418	0.81963805	0.17705984
0.21775132	0.32326459	0.29760431	0.11183543	0.46784196	0.4859861	-0.0407471
-0.2106831	-0.6418606	-0.9000178	-0.7110285	-0.7351631	-0.9799261	0.99782997
0.1449486	0.25080847	0.22764094	0.16644379	0.44688299	0.5428762	0.4434253
-0.9220881	-1.389827	-1.5732815	-1.5110901	-1.6697286	-1.6038129	0.10313
-0.1175097	-0.038854	-0.1583166	-0.353429	-0.3056791	-0.1878977	0.42614636
-0.4467378	-0.5867411	-0.8568165	-0.9700625	-0.9585902	-1.158588	0.19043099
0.02874188	-0.0133475	-0.132507	-0.2931469	0.0094208	-0.0679827	0.04890764
0.13598762	0.12366757	0.08331072	0.06352406	0.16778643	0.11301927	0.71380986
0.44362863	-0.9659065	-1.0069362	-0.8909963	-0.8384526	-0.7252075	0.31701618
0.81608036	0.14463132	0.0298042	0.10546209	0.44965636	0.4375906	0.34484142
-0.1901684	-1.0182708	-1.1956128	-1.049797	-1.1683007	-1.181916	-0.005585
-0.27122	0.04433562	-0.1336322	-0.3487023	-0.325424	-0.3231567	-0.327995
0.34015379	0.35663233	0.27041686	0.31231685	0.48316003	0.46745333	0.02151363

0.31641176	-0.6572781	-0.8669167	-0.9147637	-1.4101117	-1.2659775	0.01056693
0.31241216	0.16379051	0.22474691	0.13331767	0.27512329	0.30310787	0.5753131
0.33610624	0.21754734	0.08807191	0.06337545	0.36090084	0.32284524	-0.2625555
0.06941446	-0.0835841	-0.1478023	-0.0325974	-0.0611438	-0.1267388	0.15386539
-0.0648511	-0.1947905	-0.363156	-0.3230315	-0.2531368	-0.2957225	0.02513025
0.20222141	0.2833871	0.28219322	0.15733985	0.51385465	0.43436354	-0.0036887
-0.0789366	-1.8964705	-2.024081	-1.9908557	-2.3364725	-2.2996576	0.32154645
-0.2979058	-0.9752267	-1.0759153	-1.0014026	-1.2815571	-1.2151221	-0.0467564
0.23986589	0.27056343	0.27843475	0.19473016	0.35471061	0.43035157	-0.0250704
0.60948905	0.52341467	0.49433999	0.30093554	0.66337532	0.49497189	0.15491
-0.1977507	-0.4473437	-0.658777	-0.713209	-0.7763207	-0.7733285	0.1257214
0.21507738	-1.0777523	-1.4549979	-1.4307447	-1.5612823	-1.5807127	0.3919253
-0.0283999	0.10760255	-0.0088245	0.16352101	-0.0767887	-0.048677	0.56107898
0.11452897	0.19003622	0.26903513	0.00084287	0.22548587	0.27911613	0.033157
-0.2378419	-0.3337974	-0.4798233	-0.4740979	-0.3843275	-0.3542517	-0.2164668
-0.1296457	-0.5839103	-0.6971935	-0.8131933	-0.5932847	-0.8291491	-0.1509722
-0.0565117	-0.2704047	-0.4708963	-0.3067369	-0.4622509	-0.559026	-0.1446845
-0.0219513	-1.2619743	-1.3541097	-1.2543483	-1.1582655	-1.2084797	0.58198787
0.21902415	-0.2767989	-0.329982	-0.268773	-0.0092569	-0.0648439	-0.0132825
0.95237625	-1.199174	-1.2534781	-1.3018772	-1.4272337	-1.4827022	0.77550852

test_GSE284	test_GSE306	test_GSE306	test_GSE311	test_GSE311	test_GSE331	test_GSE331
ref_GSE2841	ref_GSE3063	ref_GSE3063	ref_GSE3114	ref_GSE3114	ref_GSE3314	ref_GSE3314
GSE28412	GSE30639	GSE30639	GSE31140	GSE31140	GSE33147	GSE33147
GEO	GEO	GEO	GEO	GEO	GEO	GEO
affymetrix	affymetrix	affymetrix	affymetrix	affymetrix	affymetrix	affymetrix
TIME:120	ara_L2:+1,EV	EVO_ARABINOSE:+1,EVO_		PMX10070:1	EVO_LACTAT	EVO_LACTAT
2106	2147	2165	2193	2196	2227	2238
-0.2058849	-0.5123015	-0.2926789	-0.1649803	-0.0004158	-0.1638625	0.26184258
-0.0951643	0.20420084	0.62783644	0.69765305	0.1313726	-0.9742302	0.49045435
-0.2322989	-0.5849147	-0.6884068	-0.1370802	1.1133607	-0.3545226	-0.1339526
0.16104898	-0.2774486	-0.2478787	-0.0293722	0.28210288	-0.1195086	-0.138079
-0.1653502	0.00346128	-0.1138256	0.03597248	-0.0190974	-0.1165201	0.03653823
-0.2698566	-0.1008564	-0.100218	-0.0991587	0.7502589	0.00040976	0.74076572
0.74134939	0.92021669	1.0601252	-0.1778304	-0.5266526	0.03045233	0.42601087
-0.4688159	-0.9224155	-1.1505912	-0.3940647	0.79913645	-0.9505234	1.0572412
0.01194546	-0.4421093	-0.6237861	-0.0252808	0.46561625	-0.1037947	-0.7123562
-0.2299963	-1.5003991	-1.2606559	-0.1984334	0.12672239	-0.1320886	-0.1724728
-0.2045816	0.28483063	0.38185631	0.08225823	0.21166387	-0.0611905	0.78769009
-0.0295491	-1.0631067	-0.5092101	-0.0816124	0.36396507	0.10498634	-0.0416336
-0.1182322	-0.0615079	0.10360667	-0.3436235	0.24407647	-0.2039632	-0.0304921
0.01976716	0.26592044	-0.0020986	-0.6556535	-0.5503011	0.19865236	0.03152637
-0.7340764	-0.7585074	0.25918977	0.22090538	-0.0750481	-0.1827677	-0.1958295
-0.1212062	-0.2138804	-0.3100504	-0.0805299	-0.138895	0.10923486	0.24962329
0.33276	-0.49082	-0.62695	0.18987	0.225	0.16089955	0.14961538
-0.1969884	0.43462824	1.0872861	0.42788614	0.01665102	0.41488625	1.0210982
-0.1689403	0.68922004	0.51338814	0.1073254	0.10153356	0.12259821	0.21262019
0.12113849	-0.0934565	-0.3481112	-0.0243988	0.00422233	-0.2919097	-0.1130912
0.11555216	0.18795623	0.42164976	-0.2807246	0.07044841	0.13921128	0.05288805
-2.0024	-0.15828	-0.091137	0.092065	0.11289	0.1372657	0.05975068
-0.3422479	-2.1555603	-1.9185253	-0.3926094	0.27551501	-0.0605496	0.24449007
0.16213	-0.52931	-0.35911	-0.26912	0.21618	0.19729284	-0.3514915
0.10310699	0.41176678	0.42276747	0.16544856	0.01645398	0.02229919	-0.4512744
0.14967093	-1.309948	-0.7810129	-0.1362833	0.10374613	-0.1345344	0.12763841
-0.091714	-0.52335	-0.37458	0.12922	0.49447	-0.6925008	0.78550187
-1.3859049	-0.3791935	-0.3583097	-0.926865	-0.2137602	-0.4198499	-1.3526042
0.16303	0.30605	0.42693	-0.1657	0.40335	-0.2891113	0.46749137
-0.2043988	1.5658101	1.4773466	-0.4078635	-0.0639039	-0.6360061	3.0032475
-0.1135205	-0.1277374	-0.0695763	-0.2036037	0.93612714	0.10348486	0.0313439
0.00325154	-0.4388416	-0.0555878	-0.4507803	0.25896541	-0.1890885	0.22227249

-0.0411863	-0.8799013	-0.7826133	-0.4480982	0.21778998	-0.0575405	0.26714865
-0.1087518	-0.1651088	-0.1552479	0.2638681	-0.0762663	0.09712243	-0.1036545
-0.0995925	-0.5565445	-0.3880617	0.05028312	0.05039933	-0.1224482	0.08296553
-0.2696326	-0.8865088	-0.5220798	-0.0083569	-0.1377639	0.00981001	-0.0120848
-0.4258181	-0.4239166	-0.3949239	-0.1264677	-0.2325688	0.05512627	0.05807592
-0.9420617	-0.4873038	0.02259076	-0.0741376	0.07396814	-0.2227296	0.06538341
-0.0664815	-0.1122488	-4.619196	0.43200728	-0.2848783	-0.079913	-0.0456304
0.22322241	-0.3102243	-4.3115652	0.18550845	-0.2932553	-0.0719088	-0.0164474
0.12376883	-0.5894038	-4.4345045	0.05462774	-0.0219389	0.43132044	0.60170611
0.18093044	0.01575382	-0.2258794	-0.3594567	-0.1290423	-0.0193878	-0.0759457
0.40320144	0.29922179	0.05898133	0.04253948	-0.3277735	0.09692785	0.12376125
-0.1032665	0.06787604	0.07324808	-2.7306346	-0.6262133	-0.6431784	0.55997845
-0.3021884	-0.37692	-0.0786562	0.088347	-0.3141666	-0.0144415	0.00385015
0.43896869	-0.1848539	-0.4507641	-0.0070662	0.0785942	-0.1848793	-0.0111284
-0.1978781	-0.4304001	-0.3608795	-0.3807377	-0.1114877	0.03123365	1.5787346
0.04440947	0.77316643	0.74769927	0.72112949	-0.2818262	0.18899537	0.06028853
0.01521254	0.22554445	0.22554763	-0.0037594	0.05190466	0.14022612	0.44740188
-0.2680249	0.04919321	0.0885908	-0.5097026	-0.1780411	0.26419487	-0.0749815
0.07261749	0.73867919	0.79216285	-0.1155263	-0.1530543	-0.178759	-0.037495
-0.1492253	-0.4983033	-0.1407805	-0.0448593	-0.2964192	0.07349888	0.09380572
0.18435519	-0.9553137	-0.871269	-0.5163197	0.24938285	-0.4425152	0.56182724
-0.2102179	-0.1697283	0.09265749	0.11665739	-0.0590538	-0.5802265	1.0834781
0.56188518	-0.2458428	-0.1379618	-0.4568079	-0.2527407	0.1932806	0.32702875
-0.0760342	0.10142334	0.27985473	-0.1435977	-0.1167281	0.37734401	0.25349225
-0.3183879	-0.4682463	-0.408465	-0.2106304	0.54631748	-0.2172714	0.11375334
-1.6174795	-2.7560544	-2.4910463	-0.2928096	1.5490604	-0.1283706	-0.186334
-0.3872499	-0.8709353	-0.663532	-0.2557557	-0.0141574	0.05504877	-1.6592647
-0.346567	-0.5776809	-0.4216528	-0.0351955	-0.2523194	-0.1552943	-0.1232875
-0.5355292	-0.0452347	-0.2956879	0.00647732	-0.1181542	-0.304026	0.74977727
-0.4489742	1.0417992	1.4113855	-0.4371646	-0.5969834	0.17118682	0.26189893
-0.2431647	0.05780454	0.27015398	-0.8707626	-0.4266308	-0.1713118	0.53135161
-0.0030215	0.018229	0.04366402	-0.0967694	0.01289523	0.16415665	0.07332273
0.16393982	0.39258775	0.35028993	-0.0478011	0.23320371	-0.1626168	-0.0897526
0.63385141	0.1381099	-0.1191888	0.17989287	-0.3740717	-0.022032	0.19041505
-0.2660232	-0.2924184	-0.2186816	-0.2222366	-0.1025618	-0.8891908	-1.9258432
-0.398364	-0.677144	-0.6158807	-0.0733863	0.18957946	0.1100804	0.13648244
0.01258051	-0.1481129	0.31473907	0.03891715	0.21442532	-0.0964475	0.13310802
-0.1924421	0.03943821	0.22334496	-0.3398147	-0.0692258	0.19077389	0.21589465
-0.661982	0.54451575	0.91654718	0.05248951	-0.0324817	-0.1329071	0.2571994
-0.0501954	-0.0244703	-0.4067414	-0.1228969	0.0919603	0.06530039	0.42505165
0.13572531	0.08812285	-0.0736918	0.06566335	0.1160611	-0.205593	-0.2863018
-0.1841974	-0.4956905	-0.3839015	-0.2615485	0.79995179	-0.3010619	0.09088768

-0.9851494	0.13265683	0.19461583	-1.0117361	0.2036059	-0.1033237	0.4448766
-0.3719997	1.0921385	1.2118602	0.22744038	-0.1033338	-0.0258909	0.095963
-0.1201808	0.02720509	0.19495556	-0.0262377	0.00205279	-0.0018311	0.60102153
-0.1101494	0.32765966	-0.0961184	0.13886218	0.23332038	0.070346	0.14247489
-0.134972	0.46277887	0.28457284	0.06181057	0.22678305	-0.0005746	-0.0528576
1.0908893	-0.4957493	-0.561814	-0.1721271	0.40664621	0.06325637	0.03864945
-0.2572104	0.30879796	0.67027875	-0.1821075	0.218608	0.17534468	0.12196902
-0.4246448	0.23412952	0.56886061	-0.1221618	0.56499489	-0.0986017	0.04510798
-0.0374591	0.03921244	-0.0594529	-0.1134142	0.21533489	-0.0139682	0.11165743
-0.0868443	-0.2908694	-0.422156	0.30249378	0.29583511	0.06456297	0.29551623
-0.2349808	-0.2023184	-0.3488125	-0.2256618	0.2771266	0.0638572	-0.0893658
0.10408391	0.12422462	-0.1697939	0.05281682	-0.1329786	-0.0539411	-0.0763914
-0.4149129	0.13023667	-0.0671838	0.08352483	-0.0700781	0.17620947	0.10830743
-0.008953	-0.0922901	-0.1452808	-0.0444535	0.12101273	-0.11914	-0.0544678
-0.10701	-1.4846	-1.2413	-0.027668	-0.011312	0.04525585	0.23372564
-0.0029847	-0.2495664	-0.1990588	0.37341624	-0.0951666	0.00869041	0.24564698
-0.4836989	-0.0198001	0.54188497	-0.1472534	0.14833832	-0.0315021	0.24752318
-0.3066927	0.96670289	0.78868655	-0.1035235	0.02535545	0.25167387	0.0398321
-0.35194	-0.73315	-0.29214	-0.08783	-0.1045	0.13332048	0.30496999
-0.0714404	-0.2278186	-0.415839	-0.4157201	-0.1179338	-0.0510339	0.02720237
-0.6448231	-0.7885409	-0.5324574	0.02452504	-0.5266411	-0.1599792	-0.127273
-0.12942	-0.28988	-0.19618	0.32518	0.10576	-0.0247929	0.34915331
-0.1211356	-0.019353	-0.0516996	0.05990578	0.04152135	-0.5539746	0.29214902
0.11228774	-0.0913631	0.00050283	0.20890849	0.1746427	-0.488695	-1.8124155
-0.1588961	-0.1716273	-0.098149	-0.118366	-0.0962683	0.09617082	0.13946592
0.64801485	1.067878	0.70879297	-0.1217926	0.59699952	0.04579522	0.08343947
-0.0247544	0.16432049	0.03362856	-0.1650989	0.56534222	-0.1478382	0.01470901
-0.1765875	0.24476469	0.16278925	0.11779702	-0.2528066	-0.1084887	0.39834733
0.097798	-3.8028	0.57557	-0.34177	0.38801	0.051021	1.0309
0.051639	0.04794	0.047366	-0.7282	-0.10806	0.1629	0.30479
-0.12909	-0.26788	-0.02569	0.0083782	-0.40012	0.0038838	0.2143
-0.18152	-0.051126	-0.11395	-0.054043	0.1916	-0.36198	0.17553
-0.11203	0.08239	0.16311	0.046825	0.10274	-0.42749	0.37204
0.16134	-0.21529	-0.2124	0.080962	-0.087864	-0.034605	0.073487
0.09436	0.12372	0.11165	-0.91332	-0.08946	-0.30981	1.1986
-0.0824101	0.19617335	-0.2837025	0.02659252	0.61388383	-0.3782833	-0.334104
0.35388057	0.06383664	-0.0663134	-0.247755	0.04845106	0.01592733	0.11755945
-0.1050188	0.41533965	0.49125508	0.36587197	0.05299619	0.96301048	0.47874161
-0.2967941	-0.9460583	-1.3104913	-0.1119389	0.59500704	-0.1072275	0.68643859
0.092664	-0.8027505	-0.7251112	-0.1388405	0.6600361	-0.0182734	0.63005376
-0.1497931	0.82127697	0.55486221	-0.0354969	-0.1935962	0.14620193	0.68886806
-0.3082723	-0.5601646	-0.7545572	-1.329102	-0.1308954	-0.4983806	0.31021158

0.5062658	-0.6148553	-0.7177853	-0.7353695	0.17285676	-0.1026223	0.09143538
0.62642505	1.2281591	1.1798724	-0.1572597	-0.1015081	0.0978047	-0.4319214
0.13513281	-0.1896102	-0.0579119	0.13033927	0.16328862	0.1347809	-0.0496752
-0.0822243	-0.3231223	-0.1262165	0.17227019	0.05574746	-0.0461469	0.04708454
-0.625455	-0.9455801	-0.7935321	-0.2537359	0.37795136	0.01190936	-0.2815568
-0.5651452	-0.7225439	-0.8446506	-0.3959096	-0.0109689	-0.3565591	0.04172832
0.01679455	-0.0712445	-0.0344312	0.39902555	-0.0460606	-0.0007807	0.09846328
-0.2680368	-0.4519653	-0.2712073	-0.2533748	0.33049781	0.10657467	-0.0114602
0.04267594	-0.0110272	0.01255753	0.048279	0.21768865	0.1486418	0.32502882
0.05727609	-1.3614675	-1.3640965	-0.2782657	1.2468949	-0.1158392	1.0477173
-0.7761554	0.2906793	0.24372676	-0.1537861	-0.014177	-0.5704213	-0.5798499
-0.3746735	0.46909278	0.43950569	-0.3017372	0.34899174	0.03715275	0.67176527
-0.2010749	-0.3184928	-0.5955064	-0.1357055	0.92280189	-0.1667731	0.06090466
-0.0945334	0.74201708	0.17434979	0.05804803	-0.103268	0.1159133	-0.0088872
-0.0966283	0.82305298	0.23065381	0.21146349	-0.1837554	0.09152241	-0.1739428
-0.0409695	0.04744224	-0.3963575	0.19600866	-0.0736411	-0.085894	0.22692907
0.12404511	-0.11911	-0.2163172	-0.5785296	-0.2837662	0.03843767	-0.23799
-0.2640618	0.1250561	0.18595316	0.02896902	0.60091162	-0.1960939	-0.1586897
0.16752658	-0.0240847	-0.2493484	0.28166438	-0.036812	0.44165514	0.90230534
-0.1370502	0.48521207	0.99796405	0.06349847	-0.3172516	0.25049784	0.1381042
0.01111194	0.22900882	1.2733124	0.37417952	-0.2421676	0.01668825	-0.0555305
-0.1138023	-0.2900334	-0.04785	-0.1302261	-0.1527583	-0.2017123	0.46957557
-0.11992	0.035585	0.078565	-0.18157	0.084148	0.072111	0.066592
-0.4909118	0.07613276	-0.0214123	-0.3531175	-0.0310913	-0.1338751	0.16003409
-0.060907	-0.13101	-0.40292	-0.063287	0.23706	-0.2463694	0.04304708
0.23175763	-1.0129878	0.27439977	-0.0127827	0.04392303	-0.314234	-0.0329549
0.10213643	-0.2284199	-0.1503499	0.04148647	0.07165248	-0.0064989	-0.1513399
-0.1454581	0.10996396	0.18060923	-0.4896085	-0.1827285	0.06812607	0.00728022
-0.3129916	0.71641444	0.39804987	0.11506409	0.38285095	-0.1932017	0.4154885
0.14182	-0.027232	-0.047296	-0.57054	0.1736	0.37478211	0.07953554
-0.8114801	-0.9978594	-1.135854	0.26320076	0.52423659	-0.1822344	0.3558043
0.17218759	-0.2517867	-0.3474426	-0.4699983	0.27108271	0.06629842	0.3388377
0.09982904	-0.0337061	0.12209914	0.03159252	0.29629647	0.00787413	0.06398444
-0.3282255	-0.2452489	-0.0369656	-0.0279104	0.19838698	0.24033954	0.11252687
-0.1825411	0.18334692	0.05448318	-0.5122882	0.11349768	0.42158423	-0.445126
0.09778387	0.36866395	0.07513882	-0.0672126	-0.0643748	-0.112062	0.12303615
-0.2347841	-0.1405081	0.09597714	0.06579318	0.27903952	0.13011595	-0.3388984
-0.1775049	-0.1588286	-0.4013768	0.00517592	0.43327328	0.00733978	0.14818316
-0.2284372	-0.4775943	-0.2620178	0.17702567	-0.0939797	-0.1215383	-0.00039
-0.3646167	-1.6151932	-1.7042718	0.22185709	0.25360606	0.06048195	1.9587655
-0.3911279	-0.0646488	-0.2141597	0.39322353	-0.0444718	-0.0568285	0.11136473
-0.2663384	-0.1752725	0.27134232	-0.0699783	-0.2720083	-0.2325905	0.91792311

-0.1753255	0.58164829	0.53850103	0.43995186	-0.3488266	0.31099399	0.21166869
-0.0064118	-0.2635554	-0.2124137	0.1324194	0.02782559	-0.0178949	0.2317688
0.08864691	0.36360227	0.1334253	0.11265355	-0.2335046	-0.0418104	-0.0864025
0.32932119	-0.5155137	-0.1385736	-0.0598531	0.15935615	0.43440441	0.16294479
0.20506182	-0.5029105	-0.3801673	0.08518483	0.11439307	-0.1951347	0.00117664
0.00568006	-0.0018683	0.05341486	-0.3360364	0.01829641	0.15041844	-0.0243695
0.00575119	0.31763025	0.20172143	-0.4849589	0.01661791	-0.1562684	-0.0839696
-0.7281891	-0.8359556	-0.5930081	-0.0836917	-0.0804723	0.14508752	0.26760697
-0.4401307	-2.5000602	-2.7909372	0.13980941	0.08017552	0.20648392	0.1141742
-0.0478269	0.87531817	0.03342104	-0.2644919	-0.1332894	-0.0512177	-0.1920676
0.26821595	1.1395681	0.98316808	-0.1988595	-0.1044598	0.15825268	-0.1091995
-0.0926946	-0.017764	-0.4720117	0.31247961	-0.3780016	0.00858649	0.02782077
-0.2786557	0.30340902	-0.0227591	0.0226011	0.20954218	0.07807698	0.12598543
0.06930087	-0.0185138	-0.0003782	0.55938603	-0.2945223	0.03320479	-0.0067832
-0.0783987	0.41258209	0.00501508	0.02413799	0.02625126	-0.1049539	0.03080018
-0.406535	0.87479219	0.80970692	-0.1294145	0.236365	-0.2225227	-0.0232611
-0.139262	1.2102165	1.0044975	-0.881374	-0.4653565	-0.031486	-0.0560872
0.09274803	0.36205458	0.93678533	0.12030836	0.2597347	0.19963621	0.43768239
-0.1269614	0.41730727	0.3243675	-0.2892665	-0.2383553	0.52587092	-0.5224902
0.06534331	0.22451526	0.07468347	0.18823853	1.1376049	-0.1711218	0.41227541
0.20412204	0.16666021	0.04081229	0.46859821	0.89119396	-0.6031679	0.6278463
0.25967842	0.36895653	0.21647998	-0.2349223	0.01067639	0.01661419	-0.1245055
-0.020747	-0.1685629	-0.3297952	-0.027112	0.40182319	-0.017446	-0.0378961
-0.020417	-0.1645572	-0.1605348	0.02718982	-0.0610882	0.06665285	-0.1213622
-0.0965245	0.46929972	0.33051178	-0.1930394	0.00956625	0.37692127	0.22386118
0.04379401	-1.4006711	-1.2774913	-0.1428617	0.30122626	0.24173734	0.30833919
0.12529898	-0.1749761	0.29571011	-0.0392587	0.32603886	-0.1955117	0.14668966
-0.4632988	-1.2921932	-1.3045548	-0.2119307	0.96820207	0.32520934	-0.6101041
0.10952699	0.73900816	0.62615565	-0.1627992	-0.2600866	0.07645688	-0.0159911
-0.0668047	-0.5086878	-0.5673306	-0.1619258	0.1021137	0.14075636	0.41598766
-0.0214029	-0.3429628	-0.3156989	-0.0345851	-0.3402781	0.2178942	0.00432314
-0.321605	-0.0980192	0.09537082	0.47675438	-0.5682419	-0.0417541	0.06765205
-0.0771043	0.25388927	0.03140023	0.26509758	-0.2203811	0.11386085	0.0845424
-0.1678945	0.1911705	0.12624583	0.48734218	-0.4864119	0.03795143	-0.032869
-0.0733785	-1.254582	-1.2398041	0.75562675	0.45300711	-0.5069983	0.78908405
0.06410302	-0.1216389	-0.3926681	0.14593933	0.25646513	0.16255454	-0.0477502
0.076873	0.18349	0.065677	0.25026	0.24787	-0.0102039	0.28250117
-0.0002018	-0.0166877	-0.1348936	-0.0661245	-0.0129163	-0.3242813	-0.0966611
-0.1161101	-1.4634679	-1.4854591	-0.3035994	0.33312467	-0.0998264	0.76786809
0.11341	-0.33274	-0.48939	-0.041456	0.042126	-0.0728905	-0.1897534
-0.2067755	-0.1379669	-0.1074793	-0.2859511	0.52915741	-0.1080154	0.57663332
-0.3403896	0.56254237	0.29962499	0.04378598	0.58782328	-0.5225712	0.47223335

0.22203649	0.11235035	0.10922707	-0.2201415	-0.0517837	-0.0004392	-0.2925954
0.16785993	0.28234299	0.0959596	-0.5431897	0.11258059	0.20952017	0.36461181
-0.1024276	0.26450965	0.24518064	-0.0617981	0.61862736	-0.1824165	-0.45425
-0.2252774	0.2102861	-0.0974461	-0.1801529	0.16861273	0.10449484	0.15356465
-0.1128163	-0.1325921	-0.0227919	-0.2061554	0.2137876	-0.0192709	0.12258305
0.16674876	-0.7614197	-0.8297565	-0.14714	0.31277999	-0.3020377	0.965976
0.04021084	0.39993023	0.36631494	0.1693829	0.26230985	0.1298673	-0.0343972
-0.3156823	-0.101904	-0.1472944	0.46253783	1.4787133	-0.0264385	1.0730295
-0.8019037	-0.4345103	-0.0235213	0.13696697	0.20086466	-0.0310462	0.18670045
-0.10882	0.32779	0.28544	-0.25302	0.072381	-0.2674519	-0.101254
0.06391773	0.09402806	-0.0070182	-0.2473134	0.17031771	-0.1623732	0.05230245
-0.0688946	-0.8070051	-0.6816195	-0.0644792	0.14054198	-0.0178322	0.58524679
-0.192793	-0.451168	-0.5839217	-0.5414166	-0.2013425	-0.1754474	0.02253416
-0.0814739	0.14518605	0.14788868	-0.1071903	0.11456666	-0.0896188	-0.1528479
0.38517392	1.3751001	1.6119462	-0.0013359	-0.5929984	-0.3933503	-0.0813626
-0.0169905	-0.288571	-0.5713398	0.13980884	-0.1064369	-0.0384326	0.0431138
-0.2574324	-0.7617523	-0.5456789	0.43427064	-0.0939941	0.19782286	0.87176342
-0.8087314	-1.1478441	-1.1767698	-0.5338386	0.42816527	0.48555567	-0.1721806
0.11705329	-0.3916676	-0.2777098	0.08897657	0.31498305	-0.1620586	0.25282573
0.13403129	-0.5157482	-0.8759582	0.15776482	0.75065132	-0.0949256	0.00072957
0.19609526	0.1802386	-0.1574232	-0.3759752	0.92677004	-0.2910584	-0.3785954
0.2257349	0.30287972	0.27103916	-0.3748386	0.15610132	0.10673276	0.02305068
0.14820978	1.1774977	1.1745868	0.22583463	0.01125569	-0.0058332	-0.1431617
0.06776116	-0.2647155	-0.2057031	-0.4764098	0.01201255	-0.0880238	0.12713554
-0.0405315	0.34190116	0.33629176	-0.4650991	0.30082218	0.09791252	0.07272259
0.28825051	-0.2843172	-0.1332183	-0.3572385	-0.3443862	-0.0466389	-0.0978549
-0.3963796	-0.7436144	-0.6188251	0.03370125	-0.0038946	-0.1222776	0.01849434
-0.3576325	-0.659962	-0.2446001	-0.2649265	0.11357735	-0.688903	-2.1472862
-0.1401151	-1.0869917	-0.7958006	0.12972641	-0.3615947	0.03354319	-0.3267743
-0.2806954	-0.1524568	-0.1130652	0.20249028	-0.0810238	-0.0461918	0.10385618
0.12021556	-0.1990426	-0.0043757	0.05154255	-0.0891188	0.11994277	0.27751946
0.10371913	-0.3917666	-0.3624733	0.20085018	-0.1100032	0.04190597	-0.0707531
-0.2253214	-1.3605172	-1.0330024	-0.0391447	-0.2224611	-1.2728644	0.91079359
0.28348954	0.16818242	-0.1815393	0.18193356	-0.2208652	-0.2126858	2.259439
-0.1527485	0.28963892	0.12798111	0.12726802	-0.3903709	-0.2365333	-0.1789144
0.14972426	0.07328909	0.10333784	-0.0617628	-0.2594362	-0.167881	0.01174378
0.04018476	-0.0253618	-0.0267232	0.07265339	-0.1961992	0.08916694	0.31342807
0.32921419	-0.3635772	-0.1812587	-0.2833521	-0.3930642	-0.3558998	0.97220734
0.04258396	-1.314106	-1.398319	0.14261829	-0.4695744	-0.0094101	2.0068779
0.19128907	0.18924934	0.06704121	0.41639506	-0.3218087	0.23425182	-0.1238863
0.46623824	1.0394795	0.83089038	0.27527974	-0.3221945	-0.0945775	0.04427538
-0.0099587	-0.2765394	-0.2140998	0.11504505	-0.176973	0.06975015	0.10789714

0.20525705	-0.5419301	-0.4439586	-0.1104448	-0.2581737	-0.1315351	-0.270491
0.0062285	0.043831	-0.11455	-0.0068776	0.16927	0.013334	0.28099
0.85430857	1.5110149	1.3501346	-0.1672488	0.19499732	-0.1426455	0.00971076
-0.1238487	0.00611305	0.43362831	-0.0887935	-0.3482749	0.11662233	-0.1567678
-0.1152484	0.20139256	0.19093944	-0.1247862	-0.1641488	-0.1640812	0.06145454
-0.0601624	-1.009381	-0.9697846	0.26663181	0.04750254	0.07357493	0.22969265
-0.0811111	-1.1164428	-0.9867972	0.0811982	-0.0670037	-0.0587734	0.24197072
-0.41775	-0.49336	-0.51788	0.10802	0.11871	0.10783	0.041803
0.29769008	-0.0408272	0.00311404	-0.7873212	0.56167109	0.0041093	-0.0042142
0.35033	0.034366	-0.021611	-0.36234	0.19876	-0.0968423	-0.4776029
0.29996748	0.88584497	1.4635365	0.11019964	0.10201184	0.02799481	-0.0788487
0.1649335	-0.3596787	-0.0494791	0.2552199	0.03536213	0.16365717	0.08869691
-0.0219908	-0.2886254	-0.333001	0.04077585	0.28506924	-0.4449904	-0.2240234
0.15288141	0.19838199	-0.0586792	-0.0703106	-0.0248313	-0.0367512	0.04130249
-0.1382624	0.11013759	0.02690453	0.11760004	-0.139786	0.20783096	0.1913627
0.10554788	-0.6862521	-0.7015008	-0.2155819	-0.0667977	-0.0615814	0.16885985
0.18804325	-0.4073473	-0.3428639	-0.2443004	-0.1436158	-0.0371962	-0.6138614
0.07721796	0.1515476	-0.1116315	-0.1286804	0.55217969	-0.1948273	-0.5139144
0.07287005	1.1236282	1.1267185	-0.7873351	-0.1326108	-0.558274	-0.4999813
0.17233325	0.49651303	0.21168085	-0.0701582	0.20759742	-0.0742806	-0.2143108
-0.0922905	-1.1926244	-1.3666973	-0.1596922	0.15418119	0.28918317	0.1445687
-0.0900678	-0.2871441	-0.4120504	-0.2682131	0.58393415	0.04530002	0.14235218
-0.083075	0.49535	0.43307	0.66466	-0.040237	0.04950149	-0.1309364
-1.3427069	-0.9580594	-1.2364803	-0.1001787	-0.0647259	0.04920107	-0.151845
0.16390344	0.34765631	0.17561338	-0.0748268	0.1266066	0.0486212	-0.0688316
-0.0151657	-0.5757563	-1.1829638	-0.2923253	0.14408887	0.00170548	-0.0455832
0.09675449	-1.2086016	-0.7663852	-0.5818745	-0.0362409	-0.017072	0.65649756
-0.3067	0.0092493	0.22948	0.42929	0.00058949	0.21603992	0.69827878
0.04649094	-0.6717306	-0.5627106	-0.2108358	1.2492714	0.37699661	0.53209172
0.00704081	-0.0997451	-0.1588181	0.03371195	-0.1191334	0.12620558	0.08586879
-0.4337832	-0.8565761	-0.8101609	-0.1899009	0.33962383	-0.1887727	0.09981119
0.11909777	-0.0442918	0.07798107	-0.5244305	0.22137026	0.13527168	-0.0503927
-0.28403	-0.29111	-0.29673	-0.0046862	0.34558	-0.2449371	0.51914188
-0.2156647	-0.4886848	-0.2546229	0.2028371	-0.5252326	-0.7177529	1.5363697
-0.0796342	-1.6084404	-1.6791143	-0.2270151	0.83868603	-0.620225	1.3102361
0.03661226	-0.7429396	-0.4323875	-0.2690755	0.12040568	-0.1814932	0.07372889
-0.566382	-0.1457262	-0.1190778	0.18713363	-0.3123236	-0.0263086	0.04316276
-0.4241278	-0.5433886	-0.7790148	-0.0838323	0.13511669	0.13139211	-0.1725338
-0.0593925	0.05962874	0.35518866	0.07994725	-0.3438732	-0.3717416	0.16388013
0.22269023	-0.0729261	-0.1394787	-0.064022	-0.0001682	-0.5198193	-0.7198115
0.28397442	-0.3711226	-0.3706634	-0.4422	0.0278233	-0.1378369	-0.2655688
0.09482148	0.62642708	0.4102828	-0.281007	-0.0387534	-0.1147144	-0.1727274

0.02916801	-0.0043924	-0.1611231	-0.1328096	0.20567081	-0.6644686	0.53485977
-0.0815379	-0.1906796	-0.1630992	0.18346184	0.02795177	-0.0954017	-0.0686481
0.03538658	0.35208874	0.36649118	0.12453649	0.03148629	-0.0074867	-0.0640718
0.29097676	0.3024678	0.16463149	0.1007909	0.1233707	-0.8081317	-1.0723171
0.11435042	0.12271556	0.05208165	-0.0323336	-0.0320313	-0.1688966	-0.1158917
-0.0493648	-0.0846544	-0.1237537	-0.1117372	0.20811686	-0.1545871	-0.3702868
-0.0777365	0.04913338	-0.0197185	0.15697038	0.20959827	-0.4624457	-0.3782212
0.0596522	-0.0878047	-4.4771744	0.17393925	-0.1988346	-0.3143458	-0.195818
0.08211625	-0.0600604	-0.1051147	-0.3186846	0.07252433	0.14779787	0.13069059
-0.0044848	0.14299	0.33025	0.32752	0.057293	-0.0822847	-0.0868269
0.00126098	0.05323438	-0.1653363	0.21115445	-0.2132094	0.05030318	0.04651716
-0.3906117	-0.4641251	-0.6561775	0.03225805	0.95583939	-0.416159	1.2341135
-0.1429856	-0.2532527	-0.2621731	0.09896894	-0.2111664	0.02453877	-0.1177751
0.12448	-0.65388	-0.65544	0.11351	-0.34382	-0.0656826	-0.1820674
-0.0463276	7.62E-05	0.28518871	-0.4205873	0.11138896	0.06008897	-0.0790036
-0.0456226	0.18357064	-0.1814457	0.14722055	0.22132898	-0.3073138	-0.1045441
0.03086145	-0.8074045	-0.7101522	-0.3583451	-0.2394464	-0.1083498	-0.0103964
-0.3289612	-1.1499742	-0.6965871	0.30610541	-0.1947702	-0.2893226	0.01308081
0.01210319	0.92180661	0.76864875	-0.1947931	-0.1620807	-0.014973	-0.4147232
-0.4323686	-1.1202011	-0.7740205	0.32052387	-0.1765669	0.07907426	-0.0757023

ref_GSE3314	test_GSE331	test_GSE331	test_GSE331	test_GSE331	test_GSE331	test_GSE331
ref_GSE3314	ref_GSE3314	ref_GSE3314	ref_GSE3314	ref_GSE3314	ref_GSE3314	ref_GSE3314
GSE33147	GSE33147	GSE33147	GSE33147	GSE33147	GSE33147	GSE33147
GEO	GEO	GEO	GEO	GEO	GEO	GEO
affymetrix	affymetrix	affymetrix	affymetrix	affymetrix	affymetrix	affymetrix
GLYCEROL:-2	EVO_LACTAT	EVO_GLYCER	EVO_GLYCER	EVO_GLYCER	EVO_GLYCER	EVO_GLYCER
2243	2246	2252	2256	2259	2260	2262
-0.3714473	0.1252802	0.01347041	0.40033136	0.20376342	0.40582405	-0.0152091
1.0463532	0.32877004	-0.2617632	0.22742948	-0.0333122	0.14555869	0.79203676
0.85134845	0.20886281	-0.2409626	0.15432157	-0.1523113	0.02723198	0.05978836
0.0968848	-0.155252	-0.279713	-0.2881206	-0.3117846	-0.4155801	-0.1097267
0.07684507	-0.1067348	0.07231418	0.06687572	0.02956341	0.18276884	-0.0398864
0.5748135	0.15058702	0.16826282	0.24973983	0.14874797	0.17159671	0.19597713
-0.1695769	-0.1085453	-0.1968095	-0.2305458	-0.0985279	-0.1446794	0.15950185
0.83281971	0.18147869	-0.214561	2.0931512	0.93562224	1.1270078	-0.1495501
0.09933341	0.10509761	-0.4624318	-0.408328	-0.3528556	-0.4164219	-0.050257
0.23255277	0.13909344	-0.0138978	-0.312911	-0.2509809	-0.4512898	-0.0582661
-0.1559339	0.26619501	-0.092733	0.30672555	0.0251208	-0.0054671	0.18658486
-0.9910423	-0.0504374	-0.363427	-0.4563115	-0.2921909	-0.0766108	0.16713102
-0.1444422	-0.2084113	0.15085499	-0.1500788	-0.025551	-0.2222164	-0.2319757
-0.0159307	-0.1438883	-0.1163023	-0.0221136	0.00577179	-0.2912286	0.23523844
-0.0169239	-0.1163132	-0.0160724	-0.1195758	-0.1090597	-0.3955814	-0.1226162
-0.2922519	-0.0180725	-0.1457216	-0.2993742	-0.0759967	-0.1967004	-0.1152049
-0.4799195	-0.0652198	-0.3178036	-0.3365331	-0.4537604	-0.3959037	-0.113156
-0.2811103	-0.0597431	-0.0230048	-0.2386896	-0.1902127	-0.1051463	-0.0355576
-0.0228587	-0.132459	-0.2517959	-0.1647009	-0.3994297	-0.3009971	0.06296877
-0.0297252	-0.188684	-0.1997298	-0.2433009	-0.1996173	-0.3253366	-0.2728911
-0.1777009	-0.0288149	0.28936147	-0.0379975	0.0182076	-0.0174212	-0.059434
-0.3130489	-0.0106249	-0.2191176	-0.1821216	-0.1473523	-0.0952973	-0.0173391
-0.5678983	-0.2826124	-0.1143216	-0.0088962	0.17141601	0.15364113	-0.2411604
0.20454584	0.43684032	-0.3643392	-0.0192061	-0.227173	-0.262051	-0.1519331
-0.1629339	0.01436338	-0.4782918	-0.1339046	-0.4346711	-0.3281894	-0.0641765
-0.2105401	0.25353629	-0.2415883	-0.4712968	0.17410105	0.07777313	-0.2873503
0.35982357	0.50011945	-0.7035535	2.2439199	0.69383546	0.79267272	-0.2376405
1.1495287	0.21757515	0.44126268	-0.0922592	-0.9360555	-1.2286842	-0.2361552
-0.0494874	-0.0075394	0.30785359	0.35835606	0.26847979	0.18537332	0.39091668
0.43024496	-0.267618	0.88181742	1.456836	0.8352714	0.90228802	2.2652478
-0.0691	0.0364467	-0.0380289	-0.1713726	-0.1100779	-0.08669	0.03147659
0.1675746	-0.0356684	0.25808291	0.07668275	0.26012121	0.23413842	-0.0964325

0.55760974	0.07522222	0.57172308	0.16040104	0.27267882	0.14459165	0.47180329
0.16015523	0.12509178	0.06668413	0.05691583	-0.0502421	-0.1107375	0.12853543
0.30562898	0.15692234	-0.0177678	0.46868335	0.05946139	0.10898969	0.21894571
-0.0534151	-0.1500967	-0.0416365	-0.24866	-0.0997022	-0.171512	-0.0572488
0.0073558	0.0193515	-0.3617484	-0.2556367	-0.4241408	-0.0759579	-0.1285589
0.14074321	0.22839321	-0.2378695	0.20106949	-0.0786707	-0.031542	0.01946522
0.16840918	0.03376266	0.0508158	0.03619237	0.09733667	0.0946054	0.0496529
-0.0123487	0.03743422	0.00139085	0.1180738	0.04414443	0.07696631	0.05600141
0.08785007	0.12540141	-0.3054042	-0.1328341	-0.0798364	-0.1287586	-0.3823236
-0.169395	-0.0660201	-0.0818753	-0.1299063	-0.1857482	-0.1848937	-0.1566235
-0.0715532	0.2102768	-0.0320392	-0.1605863	-0.109472	-0.2435225	0.150419
2.2900923	0.26325613	1.0625246	2.73066	3.2838467	3.6305242	2.6555812
0.04693343	-0.0739875	-0.0352422	-0.0606896	-0.0733832	-0.1666272	0.03718157
0.12936527	-0.1740055	0.38353014	0.09240394	-0.143809	-0.512598	0.17182789
-0.2498175	0.08380028	-0.4378359	0.1934374	-0.1648635	-0.1238682	0.49600662
-0.073474	-0.1659107	0.06632727	0.00738402	-0.0636107	-0.0926757	0.08036089
-0.0928805	0.05896856	-0.1037241	0.23877117	-0.0456286	0.07285799	0.01367382
-0.1707021	0.02532714	-0.1371649	-0.3119878	-0.1696197	-0.3318233	-0.0884686
0.32412754	-0.0559145	-0.0158184	-0.1945457	-0.1132698	-0.3710413	0.01452113
-0.0832076	0.10467627	0.11649985	0.06917107	0.10574395	-0.0402371	-0.029516
0.51526375	0.09774453	0.0100156	0.07132227	-0.2771294	-0.5182041	0.16925299
0.06304649	0.14401454	0.02282783	0.7027506	0.32906619	0.12592584	-0.3032644
-0.5709073	0.07294878	-0.3016697	-0.0264047	0.09015243	0.02396246	0.09770661
-0.1928257	0.304261	0.27059777	-0.0786148	-0.1040995	-0.0604538	0.08168896
0.28212103	-0.104283	0.11061307	0.0428409	0.03967831	0.1805294	0.08276789
2.4348094	0.40595975	0.78931203	1.8596816	2.2719291	3.2313701	-0.9691011
1.1583792	-0.038347	0.02258887	-0.6984065	-0.5361867	-0.5865466	0.11727321
-0.0172237	-0.1292181	-0.2836209	-0.456247	-0.3495856	-0.6287118	-0.0142166
-0.2313003	0.09849805	-1.2961591	0.10425979	0.31072167	-0.1780941	-1.0496812
0.04442006	0.00056283	0.18258552	0.00549157	0.12520589	0.0155772	0.26033162
0.19679149	-0.4062644	0.09310169	0.0831047	0.06837624	-0.033412	1.1161068
0.03328331	-0.0773844	0.44646629	0.09512792	0.14970928	0.04788639	0.20701891
-0.3513699	0.04335759	-0.1789203	-0.2299286	-0.254365	-0.3148246	-0.313771
-0.1844772	-0.4521842	-0.1295477	-0.1089913	-0.0751451	-0.4013945	-0.1353401
0.58101672	-0.0927876	-0.9328056	-0.7059495	-0.5375808	-0.4294208	-0.8352318
-0.0981982	-0.0020104	-0.1121685	-0.0692901	0.1126624	0.30015184	0.09238386
-0.1834683	0.14699787	0.21747421	-0.2789179	-0.1885336	-0.6862928	-0.4074709
-0.2459009	0.03485524	-0.1359135	-0.0797482	-0.0298843	0.05957749	-0.1334739
-0.0100681	0.01595819	0.04983141	-0.2613805	-0.2796432	-0.2420507	0.56300619
0.00205862	-0.0196103	-0.0135835	0.22497358	0.24065073	0.18193968	0.13944627
0.1906338	-0.1341894	-0.0638003	-0.2441283	-0.0405613	-0.153744	-0.0418198
-0.2170748	0.07104472	-0.1347572	-0.3510093	-0.3803132	-0.5325895	-0.391487

0.1849309	0.4023789	0.54606948	-0.5256669	1.513691	0.93772778	-0.2416892
0.19635291	0.0283208	0.16990277	-0.0930054	-0.071273	-0.3187336	0.64898895
-0.0827274	0.18708052	0.0126302	-0.0382178	-0.0367349	-0.0999996	0.1106486
-0.5785116	-0.0769791	-0.4791605	-0.0621754	-0.3180963	-0.1938868	-0.0380054
0.23197605	-0.0472666	-0.1411126	0.18262881	-0.1726193	-0.0013818	0.17735448
0.08890401	0.32175657	-0.1692275	0.32368822	0.27501512	0.16390176	0.1261077
-0.224	0.04361252	0.12979624	-0.0803607	0.03705306	-0.0055115	-0.0785606
0.50762129	0.03366207	0.37688827	0.16298997	0.24942557	0.12243048	0.48770891
0.11488151	0.01739851	0.13143787	0.09737944	0.01045088	0.09532835	0.12788295
-0.4096006	-0.3498323	-0.0968599	0.10797973	0.16581673	0.23135711	0.30275646
0.14818455	-0.1416778	-0.1261389	0.04442369	-0.1164252	-0.038155	0.02790972
-0.0134055	-0.0513786	-0.16971	-0.1070354	-0.0540179	-0.1505229	-0.1865108
-0.3047313	0.03978	-0.1784722	-0.2586481	-0.2606083	-0.1520629	-0.124556
0.07437458	0.00896209	-0.1795036	0.08495137	-0.0386509	-0.013888	-0.0859534
-0.2998394	0.12182469	-0.2510275	-0.1598041	-0.2985283	-0.1493289	0.27328448
0.09212213	0.15618424	0.30901918	0.01852191	0.06813233	-0.0004835	0.24626971
0.27350095	0.00736346	-0.1806737	-0.0808361	-0.0281578	0.06211035	0.02071549
-0.1861634	-0.0552658	-0.2680384	0.1158961	0.03282782	0.00155177	0.04157395
-0.1326569	0.37449758	0.03724647	0.07231241	-0.0300804	-0.1812846	0.0312278
-0.0211922	-0.0963558	-0.0275008	-0.0911608	0.32218192	0.32943389	0.12630876
0.03866195	-0.1560259	0.01871201	0.08040637	0.05348851	-0.0727456	-0.0262517
-0.0069084	0.03593554	0.3519891	0.63927026	0.20762546	0.30277683	0.06875074
0.46429112	-0.2522046	0.1175705	0.43098123	0.0163615	0.04154603	0.1314012
1.9763482	-0.0517373	-0.4713191	2.0859047	2.0936573	1.0026203	-0.1167492
-0.3458535	0.16183041	-0.2807377	-0.0329253	-0.3605487	0.08805359	-0.009755
-0.0388621	0.04717311	-0.0412904	-0.0924434	0.01670753	-0.1044455	0.05431124
0.0072014	0.0068413	0.01845568	-0.2768305	-0.0943614	-0.3345841	0.03355293
0.04301884	0.0299444	0.09946202	-0.0177402	-0.025471	-0.0437249	0.41235855
0.070416	0.024178	0.20844	0.12639	0.27855	0.11939	0.58726
-0.034977	0.067835	-0.21811	0.083085	0.19668	0.24003	0.56555
0.13696	-0.12312	-0.06174	0.10909	0.12126	0.12774	0.27973
0.56098	0.087151	0.17244	2.4487	2.4431	2.7471	0.16048
0.38949	0.19653	0.00027455	1.916	1.8626	1.6473	-0.10527
-0.15636	0.033941	0.041003	-0.084775	0.02488	-0.16258	0.066521
-0.53048	0.20537	0.18442	0.057581	0.30199	0.032431	0.662
0.96357901	0.17730306	0.11672307	0.17844794	0.29851239	0.1589236	0.1110174
-0.186149	0.05917944	-0.1247267	-0.0540105	0.00254718	-0.0634787	-0.1327472
-0.2291277	0.51866193	0.14132885	-0.621466	-0.4994589	-0.5959912	0.07313244
-0.1463243	-0.0938126	-0.0078114	-0.1746208	-0.1429773	-0.2477716	0.45803351
0.10067094	0.01039363	0.2964574	0.26453438	0.08855756	0.10617919	0.36140236
0.02765307	0.27258662	0.35053292	-0.1208428	0.26369491	0.33956049	0.38880194
-0.0734741	0.17196001	0.08746157	-0.4546359	0.00845643	-0.160667	0.52489648

-0.0599047	-0.0413979	0.17314634	0.08551371	0.20080198	-0.0943907	0.04282881
-0.1595114	0.08168612	-0.0665192	-0.2636754	-0.2091865	-0.1974372	-0.1396731
-0.2139564	0.06012691	-0.0444626	0.00966664	0.11954064	0.03514049	-0.1064392
0.18634149	0.0905537	-0.0319348	0.05595362	-0.0351398	-0.0053144	0.1061244
0.36110647	0.07541669	-0.3475263	0.05546164	-0.1108811	-0.1071195	0.50562296
0.81218093	0.11766913	0.3391074	1.4029195	1.4218948	1.6303753	0.29882951
0.1467372	-0.0179425	0.14090223	0.10540867	0.10169746	0.28430943	0.19710226
0.05723777	0.02143858	-0.0526396	-0.012596	0.08230882	-0.0065558	-0.0833887
-0.3839441	0.31815651	-0.0961557	-0.4428425	-0.2320464	-0.3652747	0.01050707
0.27014325	0.45444789	0.28540267	0.50718642	0.13682346	-0.0222711	0.18743962
0.25365646	-0.3615047	-0.9937982	-0.3814245	0.47658367	0.59768296	0.37722359
0.25389474	0.30647382	0.11359722	0.05778607	0.05072174	-0.2293407	0.08550252
0.12701156	0.19280608	-0.1672504	-0.1634178	-0.0765946	-0.0267751	-0.2692821
-0.103859	0.26059556	-0.3200227	-0.0990619	-0.1241992	0.11627173	-0.3092296
0.16099147	0.34357024	-0.4992754	-0.4214284	-0.3368075	-0.2437512	-0.2719107
-0.01443	0.15762953	0.47242714	0.25005165	0.74142186	0.4145147	0.76920722
-0.0321889	-0.0181376	0.15150431	0.25373903	0.19152292	0.10948242	-0.0437943
0.3079252	-0.0927596	1.3499316	-0.1115533	-0.1309413	0.01672176	-0.019111
0.0441241	0.1544176	0.15934356	0.2426765	0.20158613	0.36221306	0.19589084
-0.0550475	-0.0621485	0.15492178	0.03889747	0.19987768	0.00956625	0.12381963
0.14196264	0.01481604	0.0886049	0.2015663	0.22862769	0.06864699	0.10492999
0.23818943	0.04511967	0.20198571	0.43751997	0.41894658	0.31540159	0.34049973
0.38775	0.084873	-0.0093656	0.20104	0.012929	0.76972	0.2349
-0.1520653	0.00129142	0.5941623	-0.3691534	0.82995902	1.3951933	0.86756306
-0.0055208	-0.0842035	-0.1648598	0.10742644	0.28439663	0.22234184	0.48293555
0.27720851	-0.1699031	-0.0236226	0.04184684	-0.1161335	-0.1773441	0.22033428
-0.0968958	0.16949554	0.12987811	-0.2077453	-0.0764141	-0.4399968	-0.1585176
-0.6606495	-0.0086547	-0.0143237	0.04214105	0.13393711	0.06028937	-0.0250643
0.3152431	0.08015614	-0.232526	-0.1282044	-0.1128373	0.06480519	0.05053182
0.34298444	0.09210555	-0.3111488	0.26549307	0.30537142	0.40706648	-0.0143777
0.4776339	-0.0131934	0.09953703	0.25434033	0.09865714	0.00329617	0.24929305
-0.0022087	0.04457967	0.03958712	0.20732376	0.00517471	0.17055992	0.20476638
0.13765767	0.07718862	0.05300455	-0.0630462	-0.000801	0.07212623	-0.0780632
-0.053632	0.12986974	0.01038972	0.37690942	0.26905312	0.39081253	0.06494865
0.34206203	0.20541398	-0.084025	0.33181975	0.24506826	0.27576837	0.45392304
-0.034843	-0.0319933	0.04612168	0.18051357	0.12212509	0.05833412	0.11788509
0.05907349	-0.1825247	0.0981858	-0.3442845	-0.1443374	-0.1789304	0.33552809
0.41501474	0.11097579	0.04283995	0.31473213	0.19843413	0.03627428	0.05486378
0.13326768	0.00817014	0.01688293	-0.0719821	0.07240889	-0.0962826	-0.1046294
0.70633611	0.30095642	0.20541245	0.29570172	0.24361362	0.03382185	0.14485757
0.20887626	-0.1428793	0.00120495	0.15154383	0.06708441	0.0840902	-0.0366872
-0.5517456	0.35999651	-0.1504325	-0.3996255	-0.4681414	-0.5309799	0.21135003

-0.096486	0.07549132	-0.084458	0.30233314	0.12283656	0.2607689	0.22676644
0.21645613	0.23903016	0.42419587	0.14458392	0.18974132	0.0757392	0.12082261
-0.4051804	-0.0174981	-0.0031397	0.1557142	0.15648622	0.14184753	-0.0827225
-0.1437332	0.2217799	-0.0970412	0.13327372	0.13151579	0.13804654	0.09229563
0.24572731	-0.1578364	0.22670697	0.04111561	0.15052692	-0.0244715	-0.0809711
0.07607127	-0.066948	0.01299455	0.03429382	0.08985623	0.12553616	0.08308212
0.17993009	0.08259555	0.10809645	0.24876438	0.51322863	0.22706772	0.17329001
-0.4696819	-0.1219078	0.32314774	-0.0048117	-0.0037962	0.15211352	0.04369204
-0.2910891	0.22313902	-0.4169349	-0.1816647	-0.2810098	0.15277185	-0.1450401
0.11683569	-0.0720121	0.45683648	0.17844152	0.40456775	0.1735412	0.1457704
0.03581319	-0.235217	0.1210772	0.06323754	0.03149091	-0.0305977	0.13801841
-0.1375619	0.08911206	0.02870335	0.06310445	0.05404032	0.36391407	-0.0643016
-0.1769433	0.27732482	0.06644927	-0.2474516	-0.3017052	-0.4698074	-0.0943835
-0.3470754	-0.1159231	0.1374719	-0.1046789	-0.0300332	-0.2283141	-0.2853774
-0.4602953	-0.3031985	-0.0576679	0.49761664	0.45941097	0.18280757	-0.3791548
-0.0126662	0.06072665	0.10485054	0.06083153	0.20761775	-0.0602366	0.04636022
-0.1131238	0.07930686	0.038281	-0.0726726	-0.1302829	-0.3531599	-0.084894
0.0482894	0.09862192	0.13925317	-0.6193674	-0.7658261	-0.5171833	0.00181479
0.23633801	-0.1310216	0.37938545	-1.0440412	-0.5971784	-0.9532542	-0.2861218
0.18789499	0.26774098	0.1871387	0.24274464	0.32326145	0.37138907	0.00919133
0.43298371	0.21225575	-0.1193658	0.24602399	0.31165537	0.19738319	0.03301452
0.00931334	-0.0315867	0.23756714	0.20547625	0.28225966	0.21824767	-0.0377036
0.0615929	0.12049059	0.15312291	0.27776365	0.18977882	0.14790098	0.18174962
-0.0604967	-0.0955962	-0.1214805	0.08658207	-0.0550845	0.00640055	-0.0231083
-0.1281858	0.42344934	-0.0647592	0.34828742	-0.0400053	0.25918792	0.1818852
-0.1832198	0.07223523	0.14856851	-0.2316102	-0.2582578	-0.4294933	0.04530349
0.57666913	0.07387463	0.59403151	0.34484873	0.50131289	0.3975776	0.40943285
0.61844308	0.24022896	-0.2125357	-0.5798831	-0.504427	-0.6754763	-0.2798661
-0.0218158	-0.0898316	0.00796894	0.04423885	0.06515528	0.03978652	-0.0190795
0.48540786	0.07960448	0.27314224	0.36460749	0.21403405	0.20679497	0.53090571
0.02455264	0.08052441	-0.085846	0.18454645	0.09899405	0.13665539	0.23425395
-0.1129198	0.04756488	0.0969602	-0.1241452	0.00386624	-0.1190522	-0.0659177
-0.0626114	0.04967172	0.07339706	-0.0240091	0.04527794	0.01301994	-0.0752488
-0.0260964	-0.0424425	0.21891732	-0.0428499	-0.0500264	-0.13331	-0.0563753
0.96592223	0.38947676	0.00478142	0.82647747	0.34323403	0.35987772	0.08937754
0.26439219	0.1034907	0.25360029	-0.0095108	-0.134102	0.00092955	0.34968986
-0.0231693	0.08495687	0.22693628	-0.0463245	-0.0055976	-0.2148672	-0.0099321
0.29545625	0.200915	0.20701352	-0.0075088	0.13796971	-0.0388613	0.12902383
0.22742061	-0.2417736	0.1830699	-0.0756737	-0.0296461	0.0313106	0.56172486
-0.1628466	0.12884974	-0.0809465	0.15273444	0.12821968	0.20196031	-0.0650753
0.26497178	0.29900699	1.1284267	3.5979443	2.343875	2.2954457	0.79114612
1.0292053	0.27473292	1.9683508	3.1845903	1.9834879	2.0267758	0.90962739

0.04246136	-0.0099825	0.14887074	0.22887977	0.20709009	0.1958717	0.13483941
0.09199662	0.1958819	0.1245106	0.09889699	0.08654756	0.15380738	0.22618266
0.20027281	0.11036435	0.14337459	-0.2616016	-0.3805938	-0.620458	-0.2655889
-0.5670725	-0.1471177	-0.0237616	-0.1126097	0.02497187	-0.1819668	0.07995852
-0.2267296	0.11626304	0.13905269	0.21777149	0.25185213	0.18498515	-0.0733327
-0.0098611	-0.0128237	0.57520258	0.83975706	0.7353699	0.8066882	0.83477914
-0.1493947	0.20376677	-0.1277505	-0.2369617	-0.3198825	-0.1529007	-0.0676153
-0.3040707	0.13870031	0.16618355	0.40825062	0.31961912	0.30535239	-0.182979
-0.1365875	0.02111785	0.00553399	0.00725548	-0.0394996	0.0111878	0.01988251
0.02551457	-0.0108246	0.22677586	-0.1699318	0.07511109	0.30380853	-0.055345
-0.0172568	-0.1318613	0.22327059	0.10547011	0.07370993	0.18342167	0.01073598
0.11696603	0.22356224	0.56396166	-0.0703947	0.15685118	-0.0240525	0.99983639
0.29867269	0.16629977	0.60718336	-0.3100334	-0.1625009	-0.1031748	0.64170475
0.27700308	-0.0232264	0.13388132	0.02906305	0.04807904	0.04939493	0.13725386
0.34369022	0.35258513	0.35183985	-0.7732357	-0.1127705	-0.4545678	0.34581892
-0.0733749	0.08755415	0.10978837	0.04232257	-0.0188322	0.08949448	-0.0967172
-0.0373027	0.64587147	0.08838536	0.38716101	0.72678898	0.79571538	0.10813019
-0.4981857	0.22029999	-0.0752397	-0.9738727	-0.7056325	-0.2519468	-0.1794026
-0.2304106	0.28448199	0.1695168	-0.2101912	0.07428465	-0.2434324	0.00531787
0.50011057	0.12863654	0.14896267	0.27227493	-0.2723678	-0.4147363	0.35041617
1.1964427	0.25269788	0.05589905	0.65168295	-0.0233281	-0.0202659	-0.0264591
0.10974223	-0.0557686	0.23514831	0.11581393	0.26225867	0.2734665	0.15484951
-0.0051837	-0.0514397	0.11253291	-0.1270522	-0.2149167	-0.1935482	0.41766345
-0.2653539	-0.0316929	-0.0093826	0.24776301	0.38569264	0.01701227	0.02761259
-0.265825	0.08082578	0.16233134	0.08123877	0.05545155	-0.0162043	-0.0748074
-0.0931307	-0.190095	-0.0771213	0.15309263	-0.0578607	0.19657383	0.01704554
-0.0429604	-0.0175433	0.54565555	-0.045778	0.12197205	-0.0885694	-0.2372025
1.9994399	0.66339582	1.6971797	0.46756279	1.5221625	1.4588785	0.0214208
0.62001527	0.53001624	1.635178	1.3508661	0.93782351	0.78893315	0.49602606
-0.4358495	0.71385004	0.61579996	0.74528464	1.3087265	1.2822011	0.08871308
-0.2610292	0.15038171	0.71621332	-0.1027261	0.11048744	0.15580527	-0.2870463
-0.1560189	0.01433405	0.27352894	-0.0086371	-0.0925775	0.0488844	-0.1149215
0.25384013	0.38570604	-0.6697479	1.5765681	2.4811061	1.9956647	-1.1581988
-0.3405089	-0.1414146	0.8362111	-0.120597	-0.2050454	0.19608494	1.3161231
0.13569497	0.08578182	0.27344863	-0.2039117	-0.2610442	-0.1799273	-0.1589916
0.15145927	-0.0757149	1.0884677	-0.1201419	0.09111554	0.13652105	0.13255091
-0.2047294	0.11515464	0.2961839	0.17472944	-0.0194712	-0.0791254	-0.1294382
0.54308889	0.1891298	0.08719869	2.0045083	0.49247147	0.64005482	0.04573494
0.21861251	-0.4975745	-0.1831846	-0.4586633	-0.2097281	-0.3811144	-0.2295122
0.1229602	0.15109209	0.46105699	-0.3499994	-0.2061504	-0.1269001	0.51829802
0.00545156	-0.0563988	0.16682742	0.3342983	0.1659024	0.3085801	-0.0470598
-0.1776708	0.01798498	0.37218782	0.1280045	0.12899009	0.07564668	-0.0103747

0.1992818	-0.2438153	0.48556344	0.05526302	-0.0992094	0.01091244	0.04247407
0.031821	0.031568	0.027993	0.085446	0.1036	0.17219	0.073959
0.2650521	0.04819844	0.02179565	0.27671271	0.17989294	0.26160843	0.19908956
0.09490683	-0.0390015	0.07343302	0.38739058	0.25938517	0.52734416	0.16317086
0.15128155	0.10896698	0.19663469	0.0310753	0.03744803	0.11708751	0.30190212
0.02031233	0.08990372	0.44151105	-0.0305186	0.16870077	-0.014119	0.12496908
0.10255574	0.22736769	0.31144313	0.22431511	-0.0929727	-0.1560418	1.1462703
-0.12766	0.31437	0.11699	0.021835	-0.18831	-0.053124	0.047221
-0.3674946	-0.0004136	-0.0313601	0.49317074	0.28416545	0.45131362	-0.0232364
0.43380069	-0.408725	0.18757042	0.26977465	0.01334622	0.42346099	0.04568712
-0.0986351	-0.040351	0.03499623	0.48731497	0.18560388	0.54571569	0.0458742
0.10553792	0.0699433	0.10626493	0.3973743	0.24392146	0.53388911	0.28304185
0.2394785	0.26882455	0.24769402	0.08400263	0.04122202	0.15361673	-0.0113036
-0.4516745	-0.1529429	-0.0071144	0.30268314	0.37522434	0.69099242	-0.1682527
-0.2014011	0.16429507	-0.1972734	0.43219749	0.26126698	0.41258614	0.00471752
0.08325043	0.07019929	0.0542724	0.18751269	0.28181126	0.4219628	-0.0081133
-0.0880128	0.20564531	-0.1935117	-0.356973	-0.3127817	-0.4823594	-0.9188953
-0.020293	-0.2644287	-0.4279034	-0.0071859	0.13529981	0.23172486	-0.2043302
-0.6859016	-0.2855158	-0.208961	0.95536612	-0.164114	-0.2898287	-0.5161136
0.36339083	-0.0284881	0.28048171	0.24952085	0.10641581	0.03878788	0.27716517
0.25092183	0.11387527	0.3685191	-0.18277	-0.0798779	-0.068358	0.33242486
0.33768454	0.26335897	0.03560028	0.06653494	0.19253169	0.37827608	-0.0371336
-0.0568724	0.12576887	0.1525079	0.00393311	0.21359671	0.43394871	0.04835064
1.0852671	0.28818824	1.5465769	-0.7046753	-0.6742793	-0.7795562	0.01478078
-0.0109349	-0.0584687	0.44021714	0.40651777	0.6161391	0.56731735	0.08606948
-0.1175584	0.10680674	-0.0111582	0.11539352	0.07996674	0.19451568	0.29463589
0.3694381	0.17899425	0.65918561	0.88106419	0.5171582	0.52906436	0.65166995
-0.0227956	0.35361461	0.07166042	-0.0047451	0.14002064	0.29762903	0.4217357
1.1078175	-0.6431388	0.38874967	0.03643894	0.0596309	0.14369892	-0.0218529
-0.0648613	-0.0340241	0.18118912	0.27548667	0.26772456	0.55299546	-0.0108152
0.14426731	0.32407316	0.01728827	-0.1363345	-0.0779746	-0.1551071	0.36126813
0.21426151	-0.1324937	0.23741853	0.23144355	0.18079704	0.4287572	0.24845745
0.20443777	0.07743645	0.14425471	0.12150617	-0.0313477	0.1464053	-0.0866764
1.0293375	0.75916724	0.37730637	0.42080776	0.26818616	0.3386023	0.42060789
1.0838711	-0.1492425	0.96427799	1.2973798	0.39223946	0.47590779	0.66864496
0.39805373	0.13562572	0.00774853	0.03614374	0.35628441	0.09402644	0.24062299
-0.0146414	0.04610174	0.01882108	0.14746822	0.04419643	0.21450188	-0.0178518
0.0333409	-0.1356594	-0.3502196	0.36894786	0.28882588	0.66034613	-0.2208724
0.08797079	0.03878279	-0.1713863	0.73949599	0.00432191	0.51117772	0.1919335
0.07068577	0.13553877	-0.0309943	0.85938079	1.2756948	1.6415912	-0.5935998
0.48766365	0.00125042	0.52164413	0.18058188	0.24742153	0.37818439	0.13687696
-0.1173122	-0.1441033	-0.1804546	0.1688495	0.02911623	0.30667492	-0.0041867

0.16634548	0.04281447	0.56095295	1.009498	0.26241805	0.3307828	0.41670407
-0.0087508	-0.0636064	0.01659982	0.06959115	0.10478751	0.25063749	0.15810452
-0.0961826	0.24353611	-0.2492547	0.096907	-0.0735297	0.23159667	-0.0691521
0.70078662	-0.435111	-0.1927566	-0.5546342	-0.3415542	-0.5637552	-0.362906
0.14108588	-0.015848	0.19499326	0.19533244	0.08993445	0.09669121	0.06777094
0.4637557	0.21634479	0.60957045	0.04407217	0.02238979	0.16953375	0.30655375
0.52740025	0.38336167	0.14764994	0.16903452	0.1181359	0.15877358	-0.0558306
0.26877504	0.19287896	0.39524651	0.8209302	0.22959619	0.27598924	-0.1446152
0.12657936	-0.0152654	0.2010754	0.27947648	0.14817828	0.31343628	0.11933893
-0.0543394	-0.1385046	0.06527992	-0.0752165	-0.0185169	0.01515877	0.05528469
-0.2708206	-0.0290643	0.23095606	0.20077807	0.14876232	0.26223669	-0.0916232
0.65273708	0.07465096	1.0184278	0.25863807	-0.021473	0.20147938	0.53519682
-0.0687119	-0.1178719	0.20596982	-0.0680677	0.13063527	-0.0362287	0.00301609
0.00615713	-0.0250798	0.69054409	0.13942971	0.23279953	0.06444656	-0.0799993
-0.1014902	0.08855483	0.09582155	0.18384699	0.01860231	0.21455517	-0.0617044
0.38196738	-0.1311369	0.25816936	0.75427484	0.35916549	0.52056169	-0.2862721
-0.0694526	0.150649	-0.1154816	0.30967353	0.17882611	0.37592392	0.17016454
0.62104879	0.03062659	0.50343016	0.28661026	0.14368121	0.36008443	0.54853944
0.23919928	0.04823782	-0.1594373	0.09179573	-0.0565529	0.5282283	-0.0096763
-1.0187942	0.24236001	-0.5552385	-0.2047304	0.56758114	0.68085485	-0.61867

test_GSE331	test_GSE331	test_GSE331	test_GSE331	test_GSE331	test_GSE331	test_GSE331
ref_GSE3314	ref_GSE3314	ref_GSE3314	ref_GSE3314	ref_GSE3314	ref_GSE3314	ref_GSE3314
GSE33147	GSE33147	GSE33147	GSE33147	GSE33147	GSE33147	GSE33147
GEO	GEO	GEO	GEO	GEO	GEO	GEO
affymetrix	affymetrix	affymetrix	affymetrix	affymetrix	affymetrix	affymetrix
EVO_GLYCER	EVO_GLYCER	EVO_GLYCER	EVO_GLYCER	EVO_GLYCER	EVO_GLYCER	EVO_GLYCER
2264	2265	2266	2270	2271	2272	2274
0.19523588	-0.2197676	-0.1473153	-0.0272792	-0.0584691	-0.1119592	0.01682531
0.25456393	0.24850608	0.46229148	0.28605735	0.21177643	0.34573989	0.5578311
0.37959763	0.29202445	0.32382641	0.34997625	0.23389188	0.28856325	-0.0043755
-0.2367338	-0.0389193	-0.0365775	-0.1178088	-0.1030393	-0.2286953	-0.1627712
-0.0848471	-0.0326542	-0.0507781	-0.0688608	-0.1511355	-0.1038062	-0.0703437
0.44123999	0.37957769	0.3385687	0.31210013	0.36512447	0.32061417	0.08733032
0.25456853	0.36425208	0.29170231	0.19107411	0.24160025	0.13060303	0.21732577
0.03761166	-0.2464646	-0.1972743	0.08019336	0.30737177	0.15600767	-0.2630605
0.0013045	-0.1868096	-0.0030618	-0.1173883	-0.2327438	-0.1263251	-0.2978995
0.26740196	0.29770428	0.32938545	0.08139579	0.15868506	0.06758406	0.01962134
0.39073687	0.3557482	0.2989402	0.19263286	0.50333033	0.40304647	0.1537018
0.15590948	-0.0131952	-0.1848919	0.05942604	-0.2921955	-0.0869321	-0.3741267
0.03420542	0.02700445	0.01356335	-0.1511243	-0.1474532	-0.1578607	-0.0808915
0.01340589	0.23436271	0.07831495	0.03190328	0.21714106	0.11016156	-0.0012614
-0.0057941	-0.0694006	-0.0702194	-0.1659125	-0.1997801	-0.208606	-0.1495564
-0.1540288	-0.2020134	-0.1021703	-0.1746357	-0.3098324	-0.2563725	-0.313989
-0.2848326	-0.312202	-0.2739812	-0.2379708	-0.1105323	-0.1952364	-0.2953716
0.4806848	0.80087746	0.6392994	0.07966691	0.31930173	0.39118832	-0.3251817
0.06305075	0.18220238	0.10569644	0.05538101	0.03788888	0.09999734	-0.0324078
-0.2808868	-0.1475758	-0.1764515	-0.3601089	-0.3018985	-0.3434388	-0.2177378
0.04423966	0.04650853	0.07200744	-0.0319331	-0.0505747	-0.0327382	-0.0836587
-0.3277764	-0.1451112	-0.2037865	-0.3193536	-0.1796406	-0.2497437	-0.3883658
-0.2579477	-0.4936531	-0.3122189	-0.2375805	-0.3263562	-0.2855458	-0.662214
-0.2164302	-0.1662296	-0.2221646	-0.324592	-0.2754841	-0.2272646	-0.2257872
-0.2792139	-0.1387554	-0.2789418	-0.1820691	-0.3230018	-0.2345713	-0.3432938
0.08104239	-0.4554121	-0.1061054	-0.0545409	-0.1622587	-0.0765145	-0.3676823
-0.0291855	-0.1272292	-0.2019404	-0.3511777	-0.4293301	-0.3415739	-0.4589319
0.11478972	-0.1772481	0.12336924	-0.2708902	-0.1449232	-0.2508587	-0.2418306
0.91691932	1.4897571	1.1616804	0.84027695	0.99231155	0.79479607	0.33682424
2.3583308	1.9755265	2.1723499	2.3448614	2.4540791	2.5130552	1.6796164
-0.0408215	0.12826307	-0.024445	-0.1124376	0.05920907	0.01587783	-0.0019577
0.27978762	0.145049	0.2677553	0.02168925	0.02383485	0.08926532	0.06238725

0.63485502	0.76276718	0.81074964	0.62138078	0.72804504	0.6573129	0.42464323
0.0589393	0.21605251	0.05585374	-0.0497665	0.09141912	0.05222447	0.04243803
0.42995427	0.60678323	0.34295704	0.06798275	0.10049713	0.2773975	0.11276328
-0.0726104	-0.0815361	-0.0967954	-0.1170986	-0.0629961	-0.0597668	-0.1663391
-0.1419978	0.02922657	-0.1665765	-0.1648172	-0.1204266	-0.1034453	-0.020226
0.28193522	0.51333689	0.36619857	0.16507041	0.29152379	0.26272333	-0.0672741
0.06023107	0.11907173	0.08382334	0.00247349	0.10194864	-0.0006263	0.0876029
0.06550113	-0.0751815	-0.0206486	-0.1921783	-0.0286553	0.00790282	-0.0762232
-0.3443429	-0.2199237	-0.3657133	-0.4643725	-0.4165612	-0.4593198	-0.257544
-0.1298802	-0.151728	-0.1226973	-0.1802326	-0.1904832	-0.2015832	-0.1749211
0.30411451	0.35587776	0.26179495	0.17675289	0.21202684	0.27644239	0.03802762
1.4396095	1.362528	1.3390989	1.1861778	1.364605	1.4368413	1.2383009
0.15126723	0.05639822	0.07383505	0.16728921	0.05503888	0.03332101	-0.0026593
-0.0401842	-0.0462888	-0.024323	-0.047613	-0.1500823	-0.1779581	0.13280186
0.11913023	-0.0976646	0.15396664	0.13406629	0.07496844	0.07011178	0.38069249
0.35101382	0.12759771	0.29961574	0.31255652	0.13871956	0.34216289	-0.0358523
-0.1012388	-0.1618027	-0.1786143	-0.1880026	-0.1737105	-0.1972309	-0.1779769
-0.0461763	-0.0908872	-0.0120466	-0.235169	-0.2430245	-0.1697487	-0.0761318
0.26177074	0.29520462	0.35095026	0.16799462	0.26574808	0.17273489	0.21061153
-0.0600846	-0.0817686	-0.1267238	-0.1010186	-0.1299387	-0.1577232	-0.1679654
1.0706063	0.55447956	0.91077702	0.88046745	0.81829481	0.79723483	0.35719992
-0.201299	-0.5067487	-0.3909535	-0.4411333	-0.4815256	-0.4193089	0.54153569
-0.335631	-0.2510795	-0.3380222	-0.353806	-0.3922338	-0.3097588	-0.408485
0.4234345	0.25777675	0.32829578	0.15989251	0.15357643	0.20972321	0.18679566
0.04326215	0.10146106	0.03997616	0.02674464	0.03189144	-0.015303	-0.0556464
-0.2225394	-0.5607369	-0.6485892	-0.7022742	-0.7763429	-0.5292608	-1.3471336
0.53800281	0.71731082	0.70873145	0.27206471	0.53316797	0.28227293	0.48014163
-0.0624057	-0.0382143	0.0433288	-0.0651498	-0.2328335	-0.1651088	-0.0827287
-0.4935849	-1.1140764	-0.6931705	-0.793674	-0.7817833	-0.5936148	-1.4286795
0.18814972	-0.0263993	0.06517334	0.04926397	0.17939066	0.08599375	0.05283298
0.60996543	0.34572504	0.39242096	0.3061939	0.31219748	0.50065444	0.27345672
0.09959342	0.15294157	0.15922829	0.09214605	0.17246196	0.07409164	0.07053671
-0.1515239	-0.2754202	-0.126508	-0.2565413	-0.3682811	-0.319808	-0.2181596
-0.2655012	-0.2813739	-0.1809007	-0.318183	-0.3436834	-0.3901635	-0.3877917
-0.7067212	-1.010839	-0.8794077	-0.8408131	-0.895472	-0.8082388	-0.9001313
0.27682251	0.16968869	0.15036481	0.2884296	0.35503003	0.45579876	-0.2017371
0.04600841	-0.1612409	-0.1315079	-0.239411	-0.3573197	-0.119742	-0.4400155
-0.2224977	-0.2080901	-0.198351	-0.1661662	-0.1584403	-0.1694528	-0.0751806
0.30461611	0.37192797	0.32837558	0.2754289	0.20669774	0.22871845	0.34183683
0.28760401	0.31753184	0.22666965	0.2560184	0.1762029	0.29520242	0.18047264
0.17942455	0.2254623	0.17651679	-0.0409219	0.0919091	0.09042347	-0.0042653
-0.0558924	-0.0940838	-0.1079937	-0.0928342	-0.0889436	-0.0844168	-0.2015551

0.07247411	0.34397001	0.53519005	0.11041386	0.1208395	0.22223126	-0.3049393
0.93457805	0.77858499	0.93692853	0.71687587	0.63633676	0.6578554	1.1706588
0.49163762	0.49429284	0.47898464	0.20801435	0.20842305	0.23636362	0.03891644
-0.353592	-0.3602784	-0.3639722	-0.3553544	-0.2618157	-0.3220613	-0.3680818
0.22951467	0.18383659	0.17047366	0.11835663	0.36286768	0.20250249	0.2192859
0.36211735	0.53235945	0.24168544	0.20720329	0.24826175	0.3104768	0.15413256
-0.1733282	-0.025367	-0.1243547	-0.2391974	-0.0945168	-0.2547767	-0.0079568
0.31175909	0.13528021	0.30997978	0.11055989	0.20149978	0.22474153	0.14842977
0.48539286	0.32259842	0.39014651	0.34557746	0.45545582	0.2877977	0.08981168
-0.1705936	-0.1261735	-0.1164239	-0.0249579	0.03157086	0.00184768	-0.2175315
0.17343381	0.15600392	0.05125369	0.07418661	0.13128759	0.15009868	0.0061203
0.22625586	0.05075259	0.15480651	0.01143233	0.23544066	0.11789696	-0.2404136
-0.0283376	-0.2008558	-0.0364765	-0.2041244	-0.3276689	-0.268246	-0.2647336
0.15057102	-0.0909984	0.00412117	-0.103969	-0.1098464	-0.0557518	-0.1500715
0.43415497	0.64392008	0.28426653	0.17464673	0.34024035	0.43059053	0.18264036
0.44662439	0.63968658	0.55487183	0.3230535	0.45723651	0.38413132	0.30405478
0.37991894	0.15071229	0.26909452	0.28697626	0.4514019	0.45731596	0.23895322
-0.0791327	-0.1313326	-0.1152634	-0.0849637	-0.0289947	-0.000183	-0.0337727
-0.0115632	-0.0790919	0.08201949	-0.064272	-0.1109368	-0.1165257	0.13360729
-0.0863476	-0.2420911	-0.1283214	-0.0315555	-0.0175552	0.02564256	-0.0349939
-0.1022775	-0.0967141	-0.1158748	-0.090476	-0.1363229	-0.0933435	-0.1484637
0.22020951	0.11655966	0.16961344	0.12753408	0.10397892	0.20252834	-0.041821
0.50949018	0.25214465	0.30859097	0.44333328	0.5501905	0.58507655	0.20259241
-0.5724721	-0.5605185	-0.48871	-0.4557483	-0.3918222	-0.4153867	-0.547877
-0.0861377	-0.1081702	-0.1581109	-0.2449122	-0.3327404	-0.2890898	-0.259734
0.04053556	0.12448151	0.10865952	0.16335864	0.1461665	0.07942854	0.27982287
0.47927699	0.52885743	0.6080218	0.59138778	0.54971206	0.57499468	0.64043471
0.5703708	0.76310671	0.79526002	0.70978625	0.78694878	0.63110513	0.5437981
0.35209	0.24139	0.1738	0.30723	0.29939	0.25162	0.3282
0.32386	0.20975	0.26428	0.28788	0.30654	0.49218	0.1709
0.21157	0.13043	0.16418	0.35424	0.36049	0.36434	0.34526
0.25777	-0.028489	-0.0053467	-0.14298	0.0029164	0.078874	0.40809
0.072027	-0.29036	-0.12627	-0.44177	-0.15392	-0.15518	0.075822
0.039921	-0.13306	0.083018	-0.067917	-0.19452	-0.14759	-0.03215
0.010466	0.16313	0.14391	0.098632	-0.0069327	0.022916	0.021968
0.74875428	0.76916891	0.48590629	0.73740945	0.84801946	0.83362812	0.26177681
-0.096588	-0.1676247	-0.2000774	-0.0610161	-0.1241927	-0.064688	-0.2973943
-0.0473734	0.094493	0.10096154	-0.1171438	-0.1281496	-0.0626162	0.25809521
0.27533587	0.25805578	0.30295704	0.24460444	0.26541716	0.33150109	0.04764418
0.57505303	0.31554191	0.51451642	0.56123957	0.52202218	0.60409017	0.14931448
0.68704062	0.67138683	0.65070215	0.65072812	0.85769329	0.66809916	0.55728612
0.04354493	-0.0775187	0.09026445	-0.1010979	-0.2250471	-0.0284746	-0.2522759

0.00578182	-0.0304666	-0.0127099	-0.1655993	-0.1793546	-0.0331469	-0.0802921
-0.2206699	-0.3194372	-0.2864575	-0.3210455	-0.3065595	-0.4327795	-0.4979737
-0.1548291	-0.207644	-0.1905394	-0.2166719	-0.2470505	-0.188631	-0.3920938
-0.0724308	-0.0591605	0.01560302	-0.0598579	0.15923762	0.06973638	0.02953362
0.12999849	0.12744958	0.17954783	0.30003077	0.24605018	0.22039827	0.38479477
0.2090973	0.04764627	0.14947693	-0.0326272	0.14349576	0.22178603	0.01851766
0.34741608	0.15534533	0.18701817	0.31954753	0.32685168	0.31657817	0.18860965
0.27106189	-0.1242169	0.13382879	0.27238716	0.27855444	0.22645971	0.15014134
0.08177958	0.15026092	0.12126843	-0.0084846	-0.0028627	0.02542256	-0.2003568
0.45379213	0.31970943	0.31101926	0.38290541	0.37122873	0.33427523	0.40052092
-1.0543519	-0.7133204	-0.6608973	-1.2129731	-0.4519376	-0.7746525	0.00718233
0.68174742	0.62833934	0.75219584	0.48841068	0.39559335	0.48337664	0.44444485
0.11862922	-0.0873389	-0.0621916	0.03467928	-0.0300455	-0.0011916	-0.2371696
-0.0543377	-0.1288358	-0.16827	-0.2241296	-0.1819587	-0.1319191	0.11485959
0.2219353	0.12196354	0.14070387	0.03182368	0.09941347	0.12570177	0.64823916
0.61651992	0.74399029	0.49667867	0.70490303	0.64166549	0.61490452	0.44634561
0.28631341	0.25767297	0.20627645	-0.045391	0.20174009	0.15133308	0.30122361
0.04317566	0.10796613	-0.0076044	-0.0149737	0.09398934	0.12903572	0.08539334
0.30082776	0.40458301	0.32435532	0.37295868	0.40251952	0.37202876	0.46227172
0.20494072	0.32661372	0.18781978	0.07420323	0.05968834	0.07095833	0.83044017
0.36109724	0.31293724	0.2679027	0.22284299	0.1155081	0.17387809	0.24432493
0.47348571	0.51447333	0.50116573	0.37167805	0.66004491	0.55861534	-0.0520185
0.68713	0.38531	0.51813	0.71316	0.64467	0.5945	0.2775
0.60366315	0.56549523	0.57660145	0.54825241	0.87885696	0.99474613	-0.3478206
-0.0493546	-0.0395308	-0.0466894	0.13305015	0.22418376	0.16842563	0.19460321
0.45092888	0.31909634	0.32830377	0.42460555	0.54782012	0.44557236	0.25804162
0.22299198	0.20545007	0.15838728	0.20238645	0.13091582	0.238641	0.12534577
-0.389039	-0.4964896	-0.4811689	-0.5491399	-0.7160143	-0.5132893	-0.6870633
0.20326215	0.19428578	0.21991289	0.24860906	0.38716392	0.33828951	0.20816973
-0.1423818	-0.1180618	-0.1793573	-0.0318507	-0.0209807	-0.0280931	0.03334913
0.47611247	0.29781251	0.3809659	0.53213928	0.62405894	0.63587423	0.36110936
0.1138941	0.05043077	0.04447016	0.0658051	0.12356373	0.15181482	0.19449586
0.12379215	0.11624707	0.03551496	0.05780541	0.15646963	0.10911813	0.17049043
0.05221869	-0.0100067	0.0049588	0.13335079	0.05901474	0.19863812	0.11429978
0.42484633	0.41578912	0.50039093	0.46517615	0.54886538	0.52590149	0.10956271
-0.06474	-0.0285829	-0.0014642	-0.0633121	-0.1329557	-0.1326798	-0.1836791
0.12699845	0.02193607	0.16084968	0.11273819	-0.0622866	0.07136194	-0.1268634
-0.0092632	0.00945302	-0.043285	-0.0044795	0.06299412	0.15797584	0.15932338
-0.0612752	-0.2146054	-0.0518457	-0.0977503	-0.2001391	-0.1488022	-0.1706659
0.67576551	0.5618043	0.60991035	0.64392062	0.64910056	0.65768574	0.2548612
0.1114408	0.11807216	0.25848512	0.13980026	0.249697	0.09833978	-0.015671
0.23573732	-0.0964153	0.04747917	0.12349614	0.16263753	0.08972475	1.468037

-0.0557761	0.07214118	0.00424328	0.1288436	0.12226559	0.12948965	-0.0010292
0.45389	0.33620972	0.36000522	0.28486627	0.33111214	0.32681107	0.22391966
-0.3242126	-0.322548	-0.3217088	-0.334537	-0.3042372	-0.3024421	-0.4202985
0.04986092	0.03478914	0.01082189	0.07229996	0.13572594	0.17620444	0.16350588
-0.0334015	0.0075388	-0.0175193	-0.0900937	-0.1464683	-0.0425352	-0.1062272
-0.0159331	-0.105447	-0.0005411	0.06595328	0.05333368	0.12271866	0.10560464
0.22270454	0.28176457	0.22119537	0.3956411	0.43773156	0.3052564	-0.1782877
-0.3472151	-0.2131906	-0.3046488	-0.0020227	-0.1551341	-0.0303676	0.33341829
-0.1167023	-0.0534345	-0.1619227	-0.1618848	-9.08E-05	-0.0101824	-0.2902847
0.38777202	0.24699464	0.22852833	0.29370312	0.28177734	0.19598104	0.04977068
-0.0872076	-0.1216823	-0.1112836	0.03122784	-0.004293	-0.0304761	-0.0392056
-0.0106829	-0.0147968	-0.1341165	-0.0225282	-0.0360135	0.04356148	-0.0829103
0.1048232	-0.0644061	0.02519221	-0.0682275	-0.1585351	-0.0698818	-0.1699628
-0.1603221	-0.2760498	-0.2457563	-0.268381	-0.3496089	-0.3162816	-0.2911228
-0.5078357	-0.6039912	-0.613782	-0.5478174	-0.8006398	-0.6794198	-1.0429771
0.13496491	-0.1445812	-0.0439911	0.34996563	0.08116416	0.17318328	-0.1337745
0.03210443	0.06418995	0.17241042	0.09154801	0.17221167	0.08375811	0.26830772
0.73958331	1.1444282	1.116647	0.904467	1.3238568	1.0181051	1.4287023
-0.2121957	-0.0671695	-0.1444226	-0.2227661	-0.4040479	-0.3079883	-0.0383949
0.13798428	0.14792525	0.13913914	0.12180404	0.18123734	0.10298109	0.06831492
0.10115596	0.07259113	0.06632043	0.14959626	0.08904216	0.06080133	-0.1454453
-0.0377924	-0.067924	-0.0767274	-0.0429929	0.01484546	-0.0613224	-0.1183391
0.2811378	0.13200609	0.25292762	0.33133111	0.32519774	0.37367132	0.11588479
-0.2365269	-0.1360227	-0.1849503	0.0374016	-0.1201622	-0.0598046	-0.0263614
-0.0871379	0.00775834	0.02889992	0.12495305	0.20477846	0.18929767	0.11694726
0.25859112	0.30469609	0.33279748	0.23141457	0.17210071	0.17770729	0.01818039
0.95912402	0.6355535	0.79897268	1.1290849	1.0280041	1.0211019	0.33505367
-0.0263427	-0.0868444	-0.0307547	0.06243436	-0.026354	-0.0539301	-0.1041935
0.15128811	0.21890648	0.19091111	0.24335346	0.2362722	0.23712436	-0.0233605
0.71808923	0.6980025	0.62066855	0.77625809	0.91642882	0.86436748	0.60027646
0.15252698	0.10849364	0.10467824	0.14558556	0.24845925	0.27897036	0.10756842
-0.0836039	-0.1207209	-0.0778442	-0.0998856	-0.121045	-0.1054629	-0.0713644
0.0568434	0.04174357	0.08630157	-0.0681196	0.01064909	-0.0284661	0.0836143
-0.0851675	-0.038747	0.00524364	-0.0946021	-0.0536102	0.00089696	-0.0223576
0.25600563	0.26751152	0.1708454	0.38869639	0.42765411	0.46122653	0.35191297
0.26194308	0.32315766	0.2946895	0.28701054	0.51836642	0.45292226	0.42593822
0.07466278	0.0837764	0.0867058	0.01365909	-0.0177155	0.02075728	0.01344676
0.4077778	0.37183702	0.24938315	0.47084441	0.56673318	0.41225595	0.38701475
1.7680147	1.3079378	1.6025127	1.9967206	1.8785036	1.8744706	0.76316384
0.01904659	-0.1773052	-0.0924031	0.04166906	0.07527005	0.03265297	0.00651154
0.9648482	0.97220512	0.98485349	1.0801617	1.3241696	1.2128356	0.64549116
1.2611035	1.2259742	1.4033642	1.3655425	1.6616658	1.4616046	1.1098279

-0.0549991	-0.0284762	-0.0602029	-0.0066803	0.15622473	0.04558994	0.05526087
0.64531669	0.45028893	0.43578902	0.62777273	0.69106849	0.67723999	0.32074543
-0.1527225	-0.1064332	0.02795763	-0.1661183	-0.2587696	-0.2008456	0.25353868
-0.2219272	-0.2001037	-0.1554619	-0.1637323	-0.2239797	-0.1060193	-0.0018634
0.28345248	0.17518744	0.20794764	0.17404354	0.19983455	0.14672472	0.19934908
0.48726771	0.53419311	0.35082798	0.67018773	0.81181689	0.78321903	0.51072685
-0.0609759	0.17284174	0.15280918	0.07367207	0.08983408	0.01227991	0.33870171
0.13221998	-0.0993824	-0.1641959	0.13564816	0.01218652	-0.0291993	0.03841081
0.0365485	0.04288907	-0.0041386	0.01804449	-0.0436715	-0.0644658	0.03827871
0.28339198	0.19993179	0.0549943	0.32149547	0.172101	0.20093419	0.46463736
0.81488588	1.1297475	0.90096902	0.6185681	0.75892279	0.58170434	0.42805586
0.79329579	1.0167337	0.92362608	0.88964716	0.98970879	0.78971398	1.2086334
0.4503673	0.68328659	0.58889836	0.6105095	0.78962369	0.84987103	0.74649342
0.35268501	0.35435693	0.27397539	0.27459253	0.39295003	0.31363279	0.4532152
1.0214231	1.1506415	1.0563209	0.97773222	1.0117691	1.1470341	0.84175413
-0.0729825	-0.0798576	-0.0936144	-0.0431249	-0.0675083	-0.0821517	0.07939147
1.0634371	0.95286531	0.92007222	1.1801825	1.2065951	1.2823623	0.57306006
-0.2453032	-0.3420148	-0.1364228	0.07257694	0.18344008	0.13557393	0.12935722
-0.0689821	0.16212006	0.09494908	0.26727457	0.04904406	-0.0242436	0.49775853
0.52586065	0.38213952	0.44613387	0.43807193	0.37992264	0.2470311	0.55893313
0.21664924	0.23419795	0.09717214	0.21560156	0.32645192	0.2070641	0.30992435
-0.0035552	0.0842157	0.04946973	0.19790749	0.11960616	0.18565279	0.15161341
0.25620125	0.48300215	0.38146276	0.44121658	0.50466781	0.44252593	0.50795365
0.00427006	-0.1883477	-0.1000311	0.09052886	-0.1976015	-0.0867327	0.00050148
-0.0816874	-0.1135856	-0.130268	-0.0050622	-0.0329678	-0.083379	0.06076429
-0.2625376	-0.188371	-0.2313695	0.06589517	-0.123676	-0.113001	-0.1375552
-0.1976509	-0.1891548	-0.2550531	-0.3160468	-0.3011859	-0.2849484	0.32256584
-0.3628319	-0.5797255	-0.4548759	-0.5044331	-0.6513911	-0.5566436	1.6714466
0.67031076	0.50097038	0.55122318	0.31057298	0.39921919	0.3631025	1.720872
-0.211545	-0.349618	-0.2987638	-0.3164381	-0.2254077	-0.2861891	1.0671923
-0.2430594	-0.1902413	-0.2095939	-0.0736647	-0.2643664	-0.1874719	-0.0528265
0.02259029	0.06955112	0.0153182	0.14670239	0.07780302	0.12407323	-0.0247642
-1.236827	-1.3627198	-1.4063051	-1.3594575	-1.358686	-1.3363282	-0.4022614
0.67810428	0.52706655	0.44612093	0.72947416	0.72430927	0.90834894	1.6168112
0.11336511	-0.0232795	0.01644322	-0.0028392	-0.0928348	-0.1213426	0.03911274
0.45428997	0.54374122	0.39572635	0.31435295	0.38461416	0.29603733	0.7134219
0.03957318	-0.0592987	-0.0492272	0.19503884	0.1427719	0.14967449	0.26715134
0.59008533	0.4456563	0.64355614	0.80287398	0.82751365	0.78940742	0.53704696
-0.1383042	-0.1535636	-0.1594046	-0.1046421	-0.263343	-0.3917372	0.10528419
0.93104503	0.90345587	0.94530631	0.76349271	0.71777057	0.57488521	1.0362724
0.14769986	0.25232737	0.12829595	0.22890085	0.30015665	0.29739609	0.22910077
0.03999565	-0.0494541	-0.0489016	-0.0560028	-0.0610971	-0.1097547	1.45E-05

0.12083605	0.18563641	0.09301464	0.12994763	0.11328339	0.12230091	0.01069011
-0.023654	-0.064188	0.050981	0.094027	0.061234	0.067088	0.055352
0.21299371	0.32372558	0.26748371	0.33279841	0.48284746	0.43781367	0.40107107
0.00169248	0.1896001	0.02637559	0.09704623	0.20009489	0.20088516	0.30405884
0.58239505	0.7288071	0.44485454	0.79373796	0.85577856	0.87885321	0.72100581
0.34372395	0.34112753	0.31574641	0.2872176	0.36840451	0.37198852	0.1994924
1.3193195	1.2370199	1.350809	1.1180816	1.4057434	1.3585921	1.2911965
0.15335	0.10617	0.11468	0.061119	0.15384	0.15509	0.12264
-0.4001519	-0.4136174	-0.4060634	-0.2796122	-0.3268061	-0.3772743	-0.3446036
0.29782251	0.39483615	0.28759808	0.45301839	0.57145279	0.56022826	0.50767224
-0.3321419	-0.1765931	-0.2550168	-0.0952152	0.03934663	-0.0179111	0.21726365
0.13945191	0.08928161	0.12257656	0.16428338	0.17438366	0.19117649	0.31256233
0.31399883	0.37413992	0.22899406	0.39726821	0.39527832	0.25664993	0.30659304
-0.4807603	-0.4531446	-0.4654955	-0.3264526	-0.4164756	-0.395227	-0.4230142
-0.207824	-0.1721218	-0.2060637	-0.0067217	-0.1129669	0.00782044	-0.1580836
-0.0760882	0.04157031	-0.0493615	0.13656791	0.2000256	0.2037484	0.02114751
-0.4937221	-0.5229164	-0.568921	-0.4906558	-0.5610988	-0.5681811	-0.0194924
-0.3004202	-0.1713418	-0.2589826	-0.0972497	0.00641694	-0.0580865	0.1799263
-0.274487	-0.3179591	-0.4495685	-0.1503417	-0.1400382	0.03315149	-0.4181947
0.34636472	0.44457985	0.36401355	0.3093023	0.43231922	0.23604105	0.36558378
0.59466506	0.44570445	0.473066	0.47144861	0.46067896	0.43259491	0.56712826
0.28758832	0.18260525	0.21822644	0.49662101	0.42936994	0.43034222	0.23572296
0.34969022	0.25420586	0.22677004	0.40390537	0.30417473	0.26130569	0.36324566
0.48676125	0.4060089	0.57300276	0.57161173	0.4658607	0.62076078	1.2655427
-0.1104893	-0.0849249	-0.0840157	0.04549831	-0.0976538	-0.0637822	-0.0015338
0.15712974	0.40208487	0.22783108	0.31539578	0.36006349	0.38950739	0.34237295
1.217258	1.0026036	1.0052324	1.103899	1.0750981	1.0566742	1.4395369
0.5382396	0.3657217	0.32822574	0.4624752	0.49114089	0.47975713	0.70533166
0.34417926	0.307064	0.39411637	0.95417251	0.92776399	0.85298643	1.8574029
0.06680511	0.02788925	0.05648483	0.36707083	0.37096741	0.36579416	0.15893871
0.3230703	0.14626082	0.23813439	0.37608652	0.4917585	0.42401537	0.27274014
0.08800974	0.16774748	0.0301077	0.23944315	0.16222039	0.12409582	0.19729385
0.25571358	0.20815087	0.11918101	0.28064404	0.34987924	0.23167001	0.34421097
0.4066204	0.2256283	0.31348105	0.38623543	0.38378185	0.43432463	0.41694313
1.3891234	1.116114	1.1937021	1.6731103	1.6454068	1.5873557	1.204624
0.36251112	0.29698773	0.31267899	0.53852059	0.53860773	0.53555694	0.5433173
-0.0011725	-0.0552794	-0.1270368	0.0121766	0.07511294	-0.0085989	0.29215965
-0.4545539	-0.3444116	-0.4963938	-0.2793829	-0.2883968	-0.2824853	0.11831085
0.34195162	0.8782338	0.49250979	0.82543698	0.90419581	0.92125803	0.38380783
-0.6143656	-0.7358555	-0.695487	-0.6539725	-0.9077648	-0.6865267	-0.5408461
0.49881867	0.61661303	0.45726209	0.52525843	0.55923402	0.54520798	0.51631709
-0.3998251	-0.293582	-0.3652311	-0.221521	-0.2566275	-0.1644803	-0.2999189

0.27078607	0.46128803	0.44303161	0.12378302	0.30569414	0.22145922	0.02570411
0.12326722	0.16669026	0.1070524	0.42718651	0.38683247	0.31426232	0.15999038
-0.0377861	-0.133299	-0.0755252	0.03544241	0.03315998	0.01508105	0.16327134
-0.2041774	-0.1817967	-0.2124949	-0.1245698	-0.1000877	-0.2114165	0.18849897
0.1788773	0.18631771	0.03806642	0.26024356	0.31744805	0.18688744	0.25318689
1.1001349	1.2518108	1.1349847	1.1508598	1.1226057	1.0998272	0.60269325
0.08946037	0.06741872	0.02109562	0.05352225	0.21368551	0.23155756	-0.1773245
0.04389616	0.10458477	0.03061456	0.03082789	0.16991774	0.04963658	-0.0829981
0.33535533	0.23652609	0.21561042	0.35735248	0.49418542	0.43753942	0.23543345
0.04438574	0.28749079	0.22446017	0.16391422	-0.0242475	0.06655354	0.03016666
0.02126606	-0.0256855	0.0067815	0.08298009	0.05801143	0.04046634	-0.2276924
0.51689659	0.40297398	0.55888089	0.8234458	1.3167767	1.1667659	1.3252603
-0.1181353	-0.0906458	-0.1187459	-0.0930574	-0.2272641	-0.1811735	-0.219198
-0.0383502	-0.0181479	-0.0988173	-0.0471852	-0.1654095	-0.1198082	0.03147201
0.05038277	0.00787048	0.05451639	0.11539282	0.10286745	0.08435426	-0.0754671
-0.171674	-0.2417834	-0.2024315	0.00564983	0.04958647	0.05686663	0.09192176
-0.2535319	-0.2583861	-0.2648405	-0.1591408	-0.0777748	-0.0158645	-0.014299
0.41305396	0.67669576	0.48121114	0.60084133	0.84844585	0.77453722	0.33817345
-0.0953116	-0.0101662	-0.0297526	0.08233434	0.16428386	0.19513117	0.04036942
-0.3881247	-0.6524566	-0.3738619	-0.3929294	-0.6593888	-0.4960672	-1.3436687

test_GSE331	test_GSE331	test_GSE331	test_GSE331	test_GSE331	test_GSE331	test_GSE353
ref_GSE3314	ref_GSE3314	ref_GSE3314	ref_GSE3314	ref_GSE3314	ref_GSE3314	ref_GSE3537
GSE33147	GSE33147	GSE33147	GSE33147	GSE33147	GSE33147	GSE35371
GEO	GEO	GEO	GEO	GEO	GEO	GEO
affymetrix	affymetrix	affymetrix	affymetrix	affymetrix	affymetrix	affymetrix
EVO_GLYCER	EVO_GLYCER	EVO_GLYCER	EVO_GLYCER	EVO_GLYCER	EVO_GLYCER	TIME:8,ELEC
2275	2276	2282	2286	2288	2292	2306
-0.121136	0.21687004	-0.1218467	-0.102801	0.12118492	0.11851232	-0.0725264
0.16667242	0.29144991	0.47444113	0.49889469	0.49506717	0.03553752	-0.3091645
0.00653549	0.17215238	0.38906883	0.43225891	0.21823566	-0.0595436	-0.548813
-0.1363073	-0.2087261	-0.2043547	0.00471911	-0.1651489	-0.1875317	-0.1953332
-0.1814054	-0.0458892	-0.0286335	-0.0262685	-0.0502638	-0.000814	0.07339489
0.21351199	0.25635915	0.38744833	0.21858497	0.10501111	0.1908536	-0.1430113
0.548935	0.47462012	0.36418882	0.26037026	0.28260177	-0.071352	-0.1851264
-0.528116	-0.172091	-0.1832559	-0.0147262	0.06669364	0.36349148	-0.0523619
-0.2790394	-0.2360964	0.06906559	-0.0082547	0.02807577	0.03061475	-0.5949498
0.29121665	0.32193578	0.40269048	0.22350836	0.09331147	0.1692749	0.07072463
0.10388352	0.13214984	0.35582414	0.17112746	0.27664738	0.17143718	0.01204283
-0.0660483	-0.4047743	-0.1192162	-0.263491	0.3616261	0.0769394	0.07905531
-0.1095468	-0.0366585	-0.0286995	-0.1568247	0.09411105	0.06855582	-0.0016844
-0.1147026	0.04337667	0.07338719	-0.010963	0.02606846	-0.1561342	-0.0631442
-0.2113674	-0.1891986	-0.1198343	-0.1395612	-0.0889412	-0.0029866	-0.0353671
-0.384922	-0.1814144	-0.3697179	-0.1037507	-0.3317741	-0.1366718	0.04345236
0.01359408	-0.1918881	-0.0875795	-0.100101	-0.1925594	-0.0365029	0.0038039
-0.4552758	-0.2053894	0.32517618	-0.1372062	-0.1481202	-0.293607	-0.0007724
-0.0688358	0.13999854	0.13243912	0.01508118	-0.018039	-0.078634	-0.0531522
-0.2829021	-0.202681	-0.2179484	-0.399967	-0.295559	-0.1374096	-0.2898873
-0.1245793	0.0801059	0.02950754	0.15504283	0.24072024	0.0476806	-0.0562455
-0.350974	-0.1907036	-0.2421389	-0.2050356	-0.2167671	-0.1332636	-0.12247
-0.8313574	-0.5995834	-0.6712605	-0.3276171	-0.2398826	-0.1513782	-0.4952917
-0.4264702	-0.2823129	-0.1439309	-0.2868529	-0.0695148	0.17241223	-0.066919
-0.4282612	-0.4073296	-0.2292161	-0.2532425	-0.0652104	-0.1535691	0.09558723
-0.291126	-0.3823828	-0.2533738	-0.3461842	-0.0783402	0.11052868	-0.0364524
-0.6208743	-0.2526941	-0.3924626	-0.0305404	-0.2844775	0.28346479	-0.028065
-0.0286482	0.23507029	0.6962269	0.72002763	0.66164877	0.10767347	-0.0327843
0.21564142	0.40966584	0.3481803	0.22316565	0.30373013	0.00575539	-0.070509
1.230725	1.8379035	1.4617948	2.0143575	1.7497992	0.61642108	0.07599186
0.30821041	0.12686454	0.4066146	-0.1010643	-0.0250853	0.04403344	-0.0402512
0.50832446	0.30722613	0.41992883	0.18581297	0.2390647	0.04708524	0.12734045

0.52637245	0.73727321	0.64512345	0.69851695	0.24676194	0.03924023	0.23077386
-0.016969	-0.0007998	0.03432783	0.09527797	0.03157324	0.01997972	-0.0762783
0.45042658	0.32497809	0.38763007	0.43320891	0.35655315	0.49843476	0.07563285
-0.1594926	-0.1245196	-0.1829531	-0.0265287	-0.0732681	-0.0450119	0.08609095
-0.1148389	0.16591795	-0.1349727	-0.0826318	-0.1571754	0.03588659	-0.0466786
-0.064584	0.04703229	-0.0315081	-0.0106029	-0.0680153	0.19545187	0.16264706
0.22386669	0.19211731	0.12427435	-0.050434	-0.0005492	0.00021707	0.25103389
-0.0175913	-0.0643156	0.02055022	0.01587665	0.06132108	0.09442463	0.15510354
-0.1203586	-0.2952015	-0.1194424	-0.3260103	-0.0412401	-0.1437518	-0.1535002
-0.1953681	-0.2013784	-0.1518565	-0.1100641	-0.0226448	-0.20021	0.00380693
0.00408429	0.15890263	0.25906921	0.20950026	0.23752023	0.11536166	-0.0086023
1.302555	1.6021352	1.0557261	1.5037472	1.2388065	0.80269742	-0.1512594
0.11637154	0.01496414	0.01804491	0.06317182	0.01557617	0.06897796	0.16272918
0.17546191	0.20306019	-0.0766637	-0.0102243	0.01581552	0.10652743	0.23814937
0.03397068	0.1614852	0.32505857	0.55053015	0.51806727	0.23952294	-0.1049784
0.16209172	0.18799912	0.31591527	0.13101608	0.09093812	0.0795008	-0.1041632
-0.1985802	-0.1425719	-0.2400985	-0.0283529	-0.0841539	0.12549929	0.07907103
0.19823765	0.16024037	0.16891566	-0.0733873	0.07917778	0.08180762	0.02632973
0.25219592	0.35500743	0.2094181	0.3020356	-0.0329692	-0.1732903	-0.0195838
-0.1575492	-0.0965377	0.00757439	-0.0885799	0.0106677	0.01547636	0.00420431
0.59410328	0.6073499	0.20968182	0.11168238	0.01462054	0.02659313	-0.0774687
0.65669932	0.65808627	-0.3894332	-0.3584932	-0.4122058	-0.2084316	-0.1094051
-0.5567424	-0.3447547	-0.5153711	-0.2524755	-0.3042859	-0.0248317	-0.042044
0.32718119	0.2804062	0.32501069	0.12679339	0.22661656	0.14692145	0.10429032
0.06956522	-0.0066023	0.17476095	0.17166072	0.01954239	0.05313455	-0.2428277
-1.1034173	-0.8663463	-0.6312776	-0.6401933	-0.9171055	0.45399605	-0.2547219
0.8860416	0.75374492	1.3530469	0.70360604	0.80657033	0.158547	0.06104781
-0.1011901	-0.0946932	-0.0339449	-0.1650695	-0.1076919	-0.1117223	0.16487314
-1.2465878	-1.2848133	-0.7037017	0.02882168	-0.7786242	-0.4387308	0.24704003
0.30393906	0.39091301	-0.1123771	0.08837138	-0.0422479	0.10907089	0.06833903
0.31534621	0.87095603	0.34024405	0.20294374	-0.0177135	0.05306216	-0.0723339
0.20692208	0.15870675	0.16862686	0.06494641	0.05838549	-0.0792087	0.07165368
-0.2514988	-0.3075226	-0.1562604	-0.3005505	-0.193646	-0.0828981	0.00352744
-0.4862961	-0.3455008	-0.3902609	-0.3130085	-0.2005227	-0.2406009	-0.4107935
-0.2562779	-0.7779633	-1.0197675	-0.8984883	-0.9572295	0.5025163	0.28085944
-0.2478195	-0.1800975	0.00388975	0.20471452	-0.0800791	-0.0504532	-0.0543053
-0.3979713	-0.2988021	-0.45245	-0.4171555	-0.4507678	0.21968815	-0.0344229
0.011813	0.08074774	-0.3502721	-0.3864373	-0.0412722	-0.0245249	-0.0102342
0.66309096	0.49921282	0.453603	0.33084683	-0.1910137	0.12593615	0.11068596
0.09463749	0.31505858	0.1287087	0.12156899	0.20946548	0.06005421	-0.1983919
0.14137686	0.13235093	0.34612921	0.05996891	0.11789997	0.04184662	0.12713653
-0.4470501	-0.1016357	-0.3243861	-0.0762027	-0.0903823	-0.0695179	0.05100638

-0.3497138	-0.2260187	0.23261464	1.3198629	0.61511822	0.3462732	0.04141308
0.82067875	0.98962621	0.85433095	0.92830245	0.54709348	-0.0236365	0.0126337
0.34348201	0.30223582	0.5223086	0.36659566	0.35827913	0.03165488	0.0973965
-0.4659732	-0.4605278	-0.3129414	-0.23669	-0.2987582	0.34576559	-0.0323873
0.1921479	0.30063258	0.14184039	0.28360064	0.43997863	0.12640509	-0.0276036
0.22768434	0.26980204	0.22089581	0.1544999	0.35682124	0.13700417	0.0682691
0.27036566	0.06429265	0.04310784	-0.132183	-0.1075263	0.0749579	-0.1170979
0.15626905	0.32678239	0.405244	0.3894286	0.18472528	0.0951029	0.00896859
0.64144294	0.31134847	0.33608567	0.13430498	0.07898203	0.15424941	0.11175121
-0.3045709	-0.0973356	-0.3468678	-0.3393324	-0.0987228	0.10254519	-0.0560694
-0.0725653	0.08234254	0.33647094	0.18540795	0.18619031	0.19151934	-0.5455519
-0.1463389	-0.1634962	0.08260242	1.1387569	0.45091555	-0.0341234	0.0059808
-0.365046	-0.339374	-0.1006326	-0.116645	-0.1174127	0.12577995	-1.0781463
-0.0994261	-0.0984059	0.17247443	0.09687195	0.00040302	0.08642834	-0.0170891
0.44124708	0.36182181	0.60828155	-0.0319877	0.03327904	0.15928877	-0.3873
0.58989024	0.26253915	0.44991607	0.34689169	0.193539	0.15391987	-0.0396815
0.17769528	0.23950905	0.45851018	0.13410799	0.09431445	0.04328217	-0.1006795
-0.054857	-0.0713199	-0.0712343	-0.0630121	-0.0404945	0.00630539	0.33327458
0.14904283	-0.0118522	0.26104464	0.09399855	0.08245078	0.12721424	-0.10342
-0.0660639	0.1028811	0.02783127	-0.2237754	-0.0148579	-0.0488269	0.14307546
-0.1682113	-0.1563221	-0.0585865	-0.0997366	-5.80E-05	-0.0167195	-0.083822
-0.0103909	0.18426173	-0.1498801	0.10678338	0.15613314	0.0449698	-0.083149
0.22216754	0.25988979	0.25506613	0.39356187	0.09989814	-0.0179624	-0.0705002
-0.8968081	-0.7058197	-0.7513476	-0.307238	-0.3530228	-0.1205724	-0.2825556
-0.2289546	-0.2136689	-0.250451	-0.1138706	-0.0631282	-0.058486	0.0248837
0.44999863	0.31776095	0.31671788	0.03346682	0.00727442	0.00928998	-0.1512059
0.61678016	0.65716804	0.74726489	0.10158454	0.03267421	0.10326581	-0.1160653
0.86737187	0.70785476	0.58532522	0.39829798	0.21204311	0.2143254	-0.1335119
0.31618	0.73814	0.22814	0.29594	0.13993	0.11331	-0.018898
0.026032	0.6028	0.084664	0.092205	0.17259	0.12188	0.070381
0.44286	0.55946	0.13639	0.17602	0.07291	-0.057487	-0.091095
0.17491	0.30964	0.52642	0.056097	0.41394	0.24708	-0.12311
0.06473	0.14049	0.30119	0.1871	0.2068	0.19459	-0.07636
-0.26193	-0.24827	-0.14026	-0.069491	0.056556	-0.0060051	0.070269
-0.22484	-0.037287	-0.29539	0.023405	0.16538	0.26552	-0.095313
0.36938059	0.335814	0.55252339	0.1911361	0.02410837	-0.0089592	-0.6925525
-0.2629997	-0.2508977	-0.2577643	-0.2385884	-0.1787912	-0.18368	-0.0022915
0.62489004	0.19219969	0.43525049	0.08952833	0.09367387	0.18179222	0.0967923
-0.1990934	0.11762373	-0.0052099	0.26974674	0.10552391	-0.1071458	-0.1328253
-0.0091692	0.24355362	0.0881999	0.56702211	0.16035332	0.11728607	-0.0808787
1.0667093	0.6122332	0.9408241	0.2339199	0.05654438	0.16829434	0.04206266
-0.5081301	-0.229039	-0.2975444	0.6040459	0.01301679	0.24644133	0.01244698

0.11679907	-0.0740845	0.08702992	-0.0017263	-0.0677298	0.02978836	-0.1575038
-0.1230537	-0.3821543	-0.1527847	-0.0414349	-0.1851285	0.16137044	-0.0037644
-0.185678	-0.2823551	-0.2004274	-0.1405568	-0.0438789	-0.0012287	0.05288661
0.21874135	0.08886761	0.09871461	0.07409662	-0.0480608	0.10885029	0.06838259
0.2542431	0.28959676	0.3584989	0.23509324	-0.1166726	0.09419296	-0.0061137
0.00595339	0.1895282	0.08565535	0.20949515	0.16234292	0.4314126	-0.0268457
0.24689804	0.24811702	0.11119262	0.27689016	0.18721875	0.14996741	0.12782263
0.08726748	0.26733489	0.15404614	0.10497441	-0.0030114	0.03107512	-0.3082828
-0.253872	-0.2420508	-0.0103761	-0.1183391	-0.2022549	-0.2110713	0.10327755
0.27997254	0.37231372	0.34012377	0.20963771	0.16498591	0.12756569	-0.7985769
-0.091686	0.25096987	-0.0567544	1.0415431	-0.2880071	-0.0062266	0.33778366
0.37833527	0.44712515	0.56085303	0.37537155	0.06072185	-0.0110082	-0.0905686
-0.1916029	-0.2949212	0.06875219	-0.2905137	-0.0316016	0.15992432	-0.931976
0.23733786	0.20011755	0.08185521	-0.1500919	-0.1885542	0.14732604	-0.338935
0.84290496	0.49850651	0.70528722	0.05371434	-0.0677093	0.32329618	-0.3500721
0.64335518	0.42681349	0.18441972	0.2157904	0.16864266	0.25960222	-0.1193954
0.14850024	0.11491903	0.59972194	0.1700645	0.31765723	0.06621198	-0.0223118
0.5195818	0.14591903	0.25095138	-0.0150274	0.03167588	0.0852213	0.06515963
0.7221401	0.35256755	0.37147521	0.12287541	0.07127946	0.25669888	0.07048498
0.39866466	0.15026906	0.60520107	0.00375147	0.21927772	0.16506272	-0.0576937
0.01933097	0.3103956	0.12696204	0.31391422	0.07449325	-0.0050553	0.32194256
-0.1748621	0.03281805	-0.0729836	0.07023453	0.08946568	-0.0100812	0.01462867
0.23147	0.39952	0.36489	0.43421	0.077142	0.10463	0.12002
-1.0130992	-0.5392462	-0.2779743	0.08616202	0.30063481	0.1929483	-0.1358134
0.03774825	-0.0018998	0.00577499	0.41971371	-0.138872	0.05411706	0.01367
0.30373092	0.46459264	0.25249849	1.1890931	0.17445882	0.17775498	-0.0326807
0.18873058	0.2124842	0.13104728	0.24311872	0.09207927	-0.0291067	-0.0099637
-0.9516808	-0.7117097	-0.6227342	-0.3046807	-0.0792491	-0.1689653	-0.0445155
0.23084916	0.2005536	0.38929492	0.10065919	0.07275156	0.10046981	-0.5490016
0.14872157	-0.0269827	-0.1440774	-0.1127471	-0.2215252	0.19195268	-0.085903
-0.1246144	0.28376031	0.08811938	0.34493978	0.13483389	0.16666057	-1.1920882
0.037044	0.18595078	-0.0341594	-0.0153165	0.14922084	0.00319734	0.17554697
0.44280303	0.230615	0.16691255	-0.054354	0.03923859	0.11455707	-0.0930179
-0.09526	-0.0945076	-0.0100832	-0.0003974	0.12409837	0.22188082	0.02207516
-0.0452777	0.01810431	0.39787038	0.37737219	0.29711701	0.23962556	-0.117699
-0.2047947	-0.184193	-0.2298141	0.05295257	-0.1473933	-0.0044065	0.18298933
0.05122874	-0.1328292	0.03234313	-0.2194355	0.15529459	0.2912392	0.02218319
0.04009304	0.22406157	0.0717286	0.00283945	-0.1620605	0.05785619	-0.2245127
-0.1868808	-0.225582	-0.1254612	-0.0854689	-0.0090491	-0.1087016	-0.1074437
-0.011126	0.17850891	0.55719258	0.78932582	0.30704462	-0.0601247	-0.0670226
0.05001122	0.08876785	0.16621985	0.0721639	-0.0240453	0.02500332	0.01120301
1.6361159	1.4389191	0.26561815	0.04974133	0.0006614	0.03690061	-0.0377295

0.01009592	0.09189455	-0.0579286	0.13623604	-0.0459668	0.04844687	0.03408581
0.54183264	0.27729369	0.35441565	0.32755406	0.12469344	0.11829252	-0.027095
-0.4589198	-0.4176945	-0.3930138	-0.2776343	-0.3174349	-0.0940516	0.07554886
-0.1028031	-0.0050859	-0.0820856	0.04925111	-0.0779801	-0.0365891	-0.3334993
0.00338929	-0.1227768	-0.0649815	0.03749497	-0.0336957	-0.0403088	-0.1048109
0.03503298	0.031636	-0.0274	0.10801642	0.05665963	0.09315764	0.03486747
-0.1700187	-0.1848734	-0.2112826	0.39772875	-0.0006166	-0.1089778	0.28670357
0.94217878	0.43255324	0.37065545	0.06663397	0.20891236	-0.0141978	0.13429464
-0.3093448	-0.3207109	0.07983382	-0.2150807	-0.2062999	0.11602803	-0.9931558
0.06296726	0.10471502	0.17852638	0.17588923	0.15981338	0.09625824	-0.0201745
-0.0627903	-0.146227	-0.1572431	0.04493655	-0.0938181	-0.0033476	0.02324878
-0.0725698	-0.2142143	-0.1151575	-0.0364933	0.0785327	0.1600108	-0.149693
-0.2487212	-0.298381	-0.1550402	-0.3058934	-0.1376511	0.01105025	-0.0919845
-0.3879443	-0.3560223	-0.2639035	-0.2689461	0.01019592	-0.1300488	-0.1583314
-1.2017358	-1.0153434	-1.1177456	-0.5655833	-0.5836218	-0.2666725	0.12696451
-0.0609474	-0.1269379	0.07097322	-0.0272007	-0.1102631	0.03032619	-0.0145925
0.90225886	0.1326566	0.5490899	0.27832429	0.18513598	0.26007168	0.03455052
1.3895588	1.3701731	1.1492736	0.76178995	0.97622054	0.42356155	-0.1462794
0.19269747	0.00837944	0.29135409	0.12356937	-0.2499649	-0.1019777	0.08462388
0.04938866	0.04418367	0.16328207	0.0326061	0.11401285	-0.1099306	-0.2454614
-0.402909	-0.2620784	-0.0660994	0.01879969	0.03382768	0.09078087	-0.1791858
0.03144329	-0.1771483	-0.0115628	-0.0452785	0.04009252	-0.0090422	0.29667148
0.23545134	0.01636613	0.19896803	0.26404182	0.21283683	0.0486003	-0.2647314
-0.2143163	-0.1943138	-0.2634347	-0.0257493	0.03765402	-0.0913006	0.02355252
0.03381224	0.04782748	0.05998066	-0.0305066	0.11041144	0.12145308	0.08548393
-0.1481454	-0.0811784	-0.0495191	0.15829053	0.00110254	-0.1823908	-0.1040962
0.52167323	0.4949928	0.5572797	0.62118064	0.60340948	0.1912761	-0.1196837
-0.3998908	-0.2705148	-0.0180154	0.02887079	-0.2890266	0.08162001	-0.4527721
0.17574069	-0.1132423	0.38416537	0.14440698	0.07563441	0.13275896	0.0027225
0.48591127	0.55519941	0.66483418	0.73233831	0.21450066	0.13364429	0.08134046
0.08656002	0.20377148	0.12772459	0.05108251	0.08500649	0.07632995	-0.0622298
-0.0785515	-0.1334961	0.01839576	-0.0661027	0.03811263	-0.0447196	0.03442276
0.09772932	0.01497524	0.00565367	-0.1202642	0.10802697	0.06699116	-0.135433
-0.0038451	-0.1179406	0.08821668	-0.1030195	0.01094312	-0.0807716	-0.0320598
-0.3697013	0.35165104	-0.2592811	0.15870836	0.09617117	-0.0435604	-0.0823518
0.69191614	0.54855446	0.99707609	0.61696414	0.57923534	0.33613721	0.12460495
0.03577152	0.04063879	0.00821792	-0.0213819	0.02884534	0.0177196	-0.12843
0.52117908	0.26437739	0.77079895	0.41080021	0.19221051	0.11192378	-0.1001591
0.3942325	0.80142951	0.53767148	0.67494982	0.32632169	0.12432307	-0.6419976
-0.0059342	-0.0264115	-0.1136066	0.03379658	0.06593543	-0.0395275	0.073782
0.35488682	0.71764998	0.60739625	0.84603419	0.89300205	0.07176615	0.02629688
0.73677393	1.0881638	1.2393754	1.5950531	0.81596795	0.04422621	0.20744181

0.02372473	-0.1126022	0.13429807	0.12522185	0.25610949	0.11286846	0.03178518
0.15656947	0.28208069	0.11986851	0.33499177	0.3893609	-0.1240995	0.1199372
0.08104734	0.07984913	0.07052682	0.16777303	-0.0464438	-0.1997525	0.14677336
-0.1586941	-0.1053849	-0.1483505	-0.0886227	-0.2152612	-0.2963864	0.10775982
0.19931727	0.23637874	0.13704793	0.07189456	0.1649645	-0.0192758	0.04734612
0.02108189	0.52653569	0.1013725	0.55940675	0.26127489	-0.0804831	-0.0386319
0.61049228	0.35469443	0.47827807	0.1760016	0.09810511	-0.0704188	0.18263086
-0.4524841	-0.0665951	-0.3100118	-0.1263361	-0.0087936	-0.1061303	-0.2476111
-0.0231113	0.00946827	-0.0345027	-0.0728579	-0.0023395	0.03601931	0.00996221
0.53795646	0.42384537	0.29396319	0.00582608	0.24508519	0.08197679	0.13767
0.48678396	0.50248052	0.30112108	0.22308313	0.12186692	-0.0029189	0.00218166
0.78578034	1.0603457	0.83363713	0.94440611	0.4318548	0.01509465	0.00672537
0.68148499	0.86566493	0.53975491	0.52914547	0.43782873	-0.0409308	0.05347965
0.72682949	0.6032619	0.31948289	0.21784084	0.05853194	-0.0932905	0.28790152
1.0159524	0.42232503	0.57021509	0.25161319	0.32579048	0.32880657	0.00770186
0.02836488	0.00521753	0.12870744	-0.0083305	-0.0346613	-0.0190079	-0.1065762
0.58332704	0.76956221	0.70300826	0.74936883	0.2883549	0.1285291	0.14423001
-0.0511338	-0.0369136	-0.5096712	-0.0086715	0.18568118	-0.0571581	-0.0130266
0.57496905	0.23534135	0.34513771	0.24019062	0.21381696	-0.1680201	-0.0458957
0.30123793	0.39745971	0.35539508	0.4392913	0.2538795	0.05936319	-0.1856493
0.19626718	0.43962309	0.42573041	0.52912886	0.07881818	0.03274929	-0.963476
0.16285775	0.11352629	-0.0651434	0.1762245	0.04843778	-0.0620663	-0.0666024
0.320143	0.41662566	0.15834631	0.32509246	0.21049565	0.13372088	0.00884742
-0.436863	-0.0702074	-0.4051332	-0.139488	-0.2450298	-0.2645156	0.01210995
0.23266154	-0.0149635	0.10974181	0.22172864	0.1979629	-0.0085578	0.16953309
-0.1606902	-0.2417197	-0.2715915	-0.0546304	-0.0313461	-0.2733397	0.02651007
0.69261181	0.36160943	-0.3009641	-0.2730669	-0.1898183	-0.1675333	0.0513485
1.1793838	1.6520821	-0.6999181	-0.5453289	-0.6227051	0.12449677	0.1097669
1.744648	1.8801736	0.26055932	-0.0610697	-0.1174766	0.20756662	-0.0361579
0.78265839	0.98818472	-0.6260464	-0.5998797	-0.6868714	0.01828667	0.03334928
-0.0333716	-0.0569122	-0.1131991	-0.2348641	0.05035613	-0.0214132	0.18966949
0.138571	-0.0869776	0.08255151	0.05977345	-0.1128332	-0.2108659	0.11386451
-0.4415904	-0.1499201	-1.2938831	-1.2839564	-1.3211172	0.4481153	0.11864927
1.1045443	2.1213829	0.8752985	1.570852	1.1581867	0.07039847	0.39388588
0.28144249	-0.021597	0.1610746	0.15244524	-0.0844272	-0.1390807	0.06366777
0.90309202	0.89241679	0.54122549	0.21592387	0.14252569	-0.0970816	0.07396324
0.09533838	0.22752551	-0.1333878	-0.1590008	-0.071467	-0.1950738	-0.0497164
0.96923343	0.68070125	0.93046364	0.26561264	0.44187271	0.10992855	-0.1608008
0.17043517	-0.0237857	0.24755559	-0.1220358	-0.3183879	-0.2834559	-0.4948793
0.80695908	0.70265497	0.76890435	0.5811925	0.68065865	0.14495334	0.08741962
0.47715751	0.27995882	0.15258676	0.1769555	0.04118137	-0.1316143	-0.0346237
-0.1955129	-0.0502998	-0.1265509	-0.0610645	-0.0352174	-0.0328799	-0.2540472

0.18778809	0.07901077	0.05956576	0.03570925	0.14571676	-0.041736	0.04110039
-0.02735	0.014957	-0.040278	0.24228	0.12698	-0.11674	-0.078194
0.43989796	0.25511574	0.17472955	0.0530276	0.15177375	0.13895582	0.02428482
0.61071283	0.51294299	0.20121476	0.27203221	0.23452586	-0.0520493	-0.0449153
0.76765781	0.73479535	0.73204886	0.55768746	0.42254063	0.07217029	0.52101551
-0.0035157	0.24174138	-0.0519696	0.18655792	0.12058014	0.01055845	-0.3880165
0.62456621	1.1311531	0.56153166	1.1531438	0.89450537	0.25062607	0.11552807
0.23236	0.14307	0.28555	0.37968	0.3047	0.0014893	0.0527
-0.3916375	-0.44939	-0.5681681	-0.3759678	-0.0581791	-0.2526753	-0.155583
0.13037603	0.43829666	0.43999612	0.37536871	0.16332001	-0.0327837	0.17211
-0.1448549	-0.0197134	-0.2776058	-0.0554639	-0.0274578	0.01480138	0.18498511
0.24643648	0.28954836	0.31062549	0.26259663	0.19204441	0.08388663	-0.8305093
0.23212176	0.15260617	0.27238655	0.14287176	0.29766316	0.15546481	-0.169646
-0.5699253	-0.6629702	-0.6456154	-0.1602393	-0.1264273	-0.2299001	0.08073443
-0.2048335	-0.2185317	-0.2626266	0.03283786	0.04177124	-0.0818	-0.1082337
0.04180846	0.11038811	-0.0349602	0.04832641	0.05043456	-0.1817558	-0.1129327
-0.2929413	-0.3225581	-0.6762071	-0.7228666	-0.6122162	-0.2538589	0.03030279
-0.198725	-0.1230774	-0.1669219	0.19886838	0.00142182	-0.3039434	-0.496876
-0.715791	-0.4684746	-0.9081036	-0.9619666	-0.4939885	0.14161175	0.05454121
0.72695349	0.53445254	0.36495593	0.35287728	-0.0430582	0.12039983	-0.0550403
0.66973833	0.62322614	0.56051044	0.52075278	0.57563233	-0.0087607	-0.0583816
0.28055416	0.12957004	0.21174906	0.46768246	0.39619888	0.18218976	-0.1922088
0.44582483	0.32324669	0.12401198	0.16170936	0.01965699	-0.0247093	0.0092416
1.10996	1.1758511	1.1794419	1.5076748	0.21985753	-0.1210762	-0.0637145
-0.1364754	-0.1173969	-0.1609089	0.08618712	0.1578711	-0.0459991	-0.0967915
0.45252371	0.41332389	0.23202453	0.45111687	0.29216519	-0.047837	0.00923671
1.2503966	1.3779689	0.94848486	0.72433434	0.93169837	0.28088371	0.08234017
0.54555137	0.72317368	0.3850374	0.41312097	0.17463105	-0.1089194	0.13029
1.7679175	1.6802159	1.5398	1.7070845	1.1894857	0.12702526	0.09569128
0.09492335	0.13412102	0.02326505	0.02431829	0.12047898	-0.0818102	0.00872288
0.14504757	0.16224262	0.18850339	0.18998399	0.21042662	0.09997419	-0.1304662
0.16267508	0.26595037	0.06603068	0.28390544	0.13782114	0.11316454	-0.0092962
0.25201837	0.24202155	0.17062343	0.05770567	0.05267055	-0.0522704	0.049925
0.19051353	0.34452378	0.02171035	0.31953243	0.03204591	-0.0160215	0.16823228
0.54950038	1.4891874	0.45165012	0.57150502	0.2249918	0.06219677	-0.799254
0.71797868	0.5138103	0.28574703	0.28568035	0.04583594	0.07725087	-0.3517933
0.28905246	0.27388439	-0.0494832	-0.0011146	-0.0439088	-0.0174897	-0.0083765
0.14980192	0.23079046	-0.5734178	-0.4500004	-0.6999845	-0.1692428	-0.057389
0.34266077	0.51309329	0.32688094	0.11610661	0.23920863	0.09325399	0.16721035
-0.4100835	-0.3698179	-0.9136969	-0.683988	-0.4737872	-0.0568409	-0.0080517
0.73026136	0.58899812	0.67162516	0.41890272	0.3723134	0.10812462	0.11464412
-0.349347	-0.3171983	-0.3844711	-0.2871631	-0.1410815	-0.144127	0.01833649

-0.3164314	-0.0108255	0.05780279	0.47543044	0.26251286	-0.0250079	-0.3143368
0.09294187	0.05995498	-0.0094027	0.33580498	0.00594388	-0.1227067	0.02958517
0.22084038	0.10060764	0.27100383	0.05673498	0.07533931	0.05068161	-0.0085715
0.42453886	0.10148103	0.06111645	0.07150923	-0.1651598	-0.0755382	0.11735703
0.56747156	0.3322085	0.14410103	0.21285605	0.05548894	0.0630104	0.13314899
0.59593979	0.56147706	0.79016175	0.74286566	0.63913461	0.18706475	-0.090285
-0.2144178	-0.2367533	0.03366945	-0.082206	-0.0906411	-0.0556767	-0.5979252
-0.0198494	-0.1092404	-0.0827936	-0.0290616	-0.072435	0.02918694	-0.0052304
0.31111136	0.24445169	0.30952861	0.18289619	0.26278712	-0.1235766	0.12308517
0.02862366	0.01852859	-0.0129437	-0.0385165	-0.0742477	-0.1014359	0.10237
-0.0011452	-0.2715086	-0.1017392	-0.0582861	-0.0078648	-0.0157604	-0.0353035
-0.0424	1.290148	0.36703846	0.93334002	0.62665339	0.05285034	-0.9223193
-0.1310899	-0.1513278	-0.0920308	-0.1320735	-0.0550399	0.02170842	-0.0299435
0.84369432	0.25037109	0.11098082	-0.0723057	0.00521503	-0.0253831	-0.14605
0.00861539	-0.1816628	0.07032324	-0.055793	-0.1032176	0.11718796	0.06450505
0.00046771	-0.1161836	-0.0270632	-0.2554646	-0.2118146	0.26709998	0.02082173
-0.1870008	-0.2696825	0.04776594	-0.1175424	0.09075381	-0.104819	-0.026565
-0.0941731	0.14112224	-0.0201446	0.2251464	-0.0293908	0.02723004	-0.0319787
0.10191715	-0.2155372	0.08382338	-0.0445742	0.01967806	-0.0212294	-0.0125731
-1.7264031	-1.2724805	-1.4676681	-0.3808524	-0.4850653	-0.035469	-0.0123103

test_GSE353	test_GSE353	test_GSE353	test_GSE353	test_GSE353	test_GSE353	test_GSE353
ref_GSE3537	ref_GSE3537	ref_GSE3537	ref_GSE3537	ref_GSE3537	ref_GSE3537	ref_GSE3537
GSE35371	GSE35371	GSE35371	GSE35371	GSE35371	GSE35371	GSE35371
GEO	GEO	GEO	GEO	GEO	GEO	GEO
affymetrix	affymetrix	affymetrix	affymetrix	affymetrix	affymetrix	affymetrix
TIME:8,ELEC	TIME:8,ELEC	TIME:150,ELI	TIME:150,ELI	TIME:900,ELI	TIME:150,ELI	TIME:900,ELI
2310	2311	2312	2317	2320	2326	2335
-0.0658243	-0.117764	-0.0477705	-0.0196845	0.21359407	0.08719224	0.00411768
0.04803173	-0.6490308	0.08796387	-0.2734116	0.55576147	0.27300069	0.08045641
0.25680332	-0.8298635	-0.6977734	-1.0293439	-0.0144734	0.06252803	-0.339035
-0.0265656	-0.0646578	-0.0356412	0.03930013	-0.0193527	0.14716782	-0.037564
-0.0149121	0.14929851	-0.1386201	0.07519702	-0.1192245	0.16716135	0.03908391
-0.0183733	-0.3015839	-0.3182363	-0.3373225	-0.0650027	0.04997729	-0.1264961
0.01187001	-0.1277313	-0.0370946	0.05565473	-0.0803044	0.12329272	-0.0958806
0.09379841	-0.0441233	-0.1533839	-0.0778189	0.1650423	0.1824915	-0.0272432
0.15471304	-0.9968615	-0.6214013	-1.0123632	0.02775709	0.00657786	-0.1457545
0.0029375	-0.0176747	-0.1726354	0.02006606	0.27567082	0.00708396	-0.0500926
-0.0578841	-0.1549955	-0.0893272	0.07741157	0.32003525	0.12850523	0.01288957
-0.0367155	-0.1009339	0.10094195	-0.0323808	-0.1287367	0.21363879	0.05880152
0.17130758	-0.0328478	0.01993959	0.05680455	0.14689008	0.15292098	-0.0516781
0.12237423	-0.0733893	0.33155331	0.1454558	-0.0069643	-0.0815661	-0.0484987
-0.1078094	-0.0356765	0.17088314	0.21291354	0.10278389	0.12461376	-0.0445754
0.03979417	-0.0015542	-0.3042321	-0.2301232	-0.0291331	-0.0226945	0.01670717
0.12438	-0.031672	0.010583	0.034964	0.10311	-0.17347	-0.078133
0.05067722	-0.2713663	0.08659421	-0.0117947	-0.0414886	0.79780364	0.49534495
-0.05135	-0.1268738	0.02954522	-0.1165077	-0.1092844	-0.0958638	-0.0844237
-0.09503	-0.2545258	-0.3684675	-0.5127749	-0.0285389	0.10206776	-0.2159174
-0.0335916	0.12428352	0.04358488	0.12413344	0.09107918	0.00920448	0.01504381
0.089364	-0.14853	-0.25266	-0.35961	-0.040566	0.017914	-0.138
0.32215447	-0.6057007	-0.9114405	-1.2849478	-0.0166998	0.2124748	-0.5049841
-0.0027109	-0.13401	-0.2427	-0.19836	0.045392	0.0081753	-0.048913
0.05516582	0.21537982	-0.1087836	-0.0654285	-0.0258918	0.04139954	-0.0768838
0.06564885	0.00495521	-0.0038913	-0.0590754	-0.0181982	-0.0970785	-0.0987526
-0.090347	0.079483	-0.24873	0.039623	0.042584	0.52394	0.10354
0.18553505	-0.082036	-0.1522632	-0.1179499	-0.1228335	-0.020888	-0.1143723
-0.053038	0.03434	0.1573	0.012058	-0.15019	0.14534	-0.2703
-0.1475271	0.01428622	0.07462727	0.27715984	0.23610951	0.10609716	0.00399554
0.10706446	-0.1494898	-0.2099771	-0.2031123	0.20674308	0.09638299	-0.2410774
0.09817073	0.02803543	0.16020938	0.02715644	0.14110315	0.07660478	0.05247862

0.13336576	0.03159375	-0.0336907	-0.1285831	0.1649079	-0.025165	0.05339452
-0.0074374	-0.1692378	0.2135979	0.09700491	0.22941184	0.02813545	0.13707218
-0.0847266	0.06238313	0.02894731	0.04947986	-0.0433684	-0.0327772	0.02158035
-0.0291531	-0.0597018	-0.140367	-0.0534755	0.01101271	-0.0302682	-0.2141905
-0.1368662	-0.1496384	-0.0387283	0.06515012	0.10976181	0.08706926	-0.0993976
-0.095845	0.08191182	-0.1540062	0.01221842	0.32639911	0.15962648	0.00616867
0.09420211	0.59078608	0.13377959	0.57965347	0.24534364	-0.0766847	0.30806856
0.08470597	0.08284014	0.06441068	0.08481466	0.02073142	0.09711363	0.03318224
0.05697082	-0.199661	0.01982731	-0.0346014	0.04055228	-0.0631792	-0.1101943
0.05619798	-0.0300457	0.09878204	0.10250127	-0.0238732	-0.1345135	-0.1030126
0.00475276	-0.1765121	0.07343719	0.02326472	0.33477933	0.13014131	0.01889866
-0.0192379	-0.2121622	-0.1076452	-0.1829538	-0.2055049	0.09655662	0.03225216
0.01002935	0.11393841	0.10349494	0.01917336	0.05875506	0.02461429	-0.1227283
0.091852	0.23702411	-0.0075007	0.07939107	-0.270888	-0.1391913	0.01096966
0.03115718	-0.1794354	-0.0734502	-0.1052765	0.185492	0.09872317	-0.0820433
0.02456013	-0.1290846	0.14799098	0.11423685	0.1771093	0.0237603	0.00530145
-0.0500553	-0.0242199	-0.0475278	0.02295001	-0.0641188	-0.0724063	-0.0242138
0.08910412	-0.0208966	-0.0278998	0.00471607	0.13893953	0.0735549	-0.0458784
0.10884197	0.05099044	-0.0239132	-0.0799462	-0.0572273	0.05366365	-0.0756927
0.01522887	-0.0959312	0.06580341	-0.0917102	0.14786664	0.11329088	-0.0745917
-0.1140205	0.0424321	-0.3245683	-0.1395155	0.11897991	0.15888731	0.07058647
-0.0396146	0.05108632	0.39092947	0.13356843	0.282515	-0.2302944	0.00333806
0.08265455	-0.1840804	-0.0249208	-0.1009834	0.13231956	0.29351478	-0.1052358
-0.0309341	-0.1131283	0.17478231	-0.0175423	0.02690885	0.09591539	0.05011803
-0.0621706	-0.43354	-0.1017741	-0.5982501	0.16238316	0.1505479	-0.1508792
0.23601405	-0.4226416	-0.5006027	-0.6473368	-0.1929386	0.12838866	-0.1558762
-0.0096694	-0.0664352	-0.0195576	0.00284435	0.04376301	0.05281647	-0.0183586
0.16754482	0.14276807	0.07630622	0.15549563	0.21325597	-0.0647497	-0.0798882
0.03464035	0.23707548	0.14569093	-0.043996	-0.0777817	0.14457591	0.17986569
0.36642587	0.08572623	0.09437685	0.4057112	0.30288255	0.13435802	0.05182936
0.00080106	0.1435991	0.26472722	0.60679952	0.17354009	-0.1090994	-0.0192096
0.12653996	-0.1021192	-0.0475356	-0.1222032	0.05239097	0.04073945	-0.0415022
-0.0208416	-0.1149367	0.04505081	-0.0500375	0.05967038	0.00642295	-0.1057951
0.32052074	-0.7963636	-0.466977	-0.8839064	-0.0485267	-0.0775878	-0.0969539
-0.0519986	0.54784946	0.17287256	0.91517838	0.61442343	0.05003545	0.5339242
0.13784931	-0.1701938	-0.1017439	-0.3173731	0.21065993	0.30426225	-0.0086467
0.02445712	-0.0199629	-0.0494425	-0.0542416	0.14693781	0.07981802	-0.0290254
-0.0960126	-0.0708519	-0.1067667	-0.2476244	0.08387986	0.06505727	-0.1743552
0.10216347	-0.0109212	-0.0350966	0.05614095	0.08120996	0.11319352	-0.118414
0.06647061	-0.1826671	-0.082238	-0.2491716	-0.0523651	-0.0859271	-0.1228352
0.06072292	0.1390219	-0.0750273	0.05656778	0.04603808	-0.1074861	-0.0018081
-0.0231553	0.05483287	0.1103391	0.19527842	0.08467576	0.07965063	0.06849563

0.07442714	0.05456877	-0.0822482	-0.0730893	-0.0592923	0.05907339	-0.0143206
-0.0286165	-0.1476643	-0.0606893	-0.1866313	0.01472574	-0.0977048	0.0217707
0.19189354	0.13462716	0.0052998	-0.2572922	-0.0713548	-0.0248377	-0.0998866
0.06889363	0.05007381	-0.1051457	0.05330889	0.02774104	0.11591088	-0.1513154
-0.0180112	0.0421758	0.05287577	0.01278227	0.08736027	-0.0406241	0.04304961
0.01368815	0.21210384	-0.2613224	-0.2338119	-0.2841995	-0.134301	-0.0599301
-0.0176623	-0.0033002	0.21288303	0.20886789	0.00847107	-0.0543714	0.13254523
-0.1040438	-0.0082078	0.04074089	-0.0627651	0.39072705	1.1008332	-0.0988013
0.06232329	0.05765924	-0.0409081	0.15904291	0.15577645	0.01681752	-0.0650514
0.01696898	-0.1031999	-0.0137987	-0.0413226	0.12736863	0.08906534	-0.037655
0.05408249	-0.8600246	-0.4345994	-0.8018682	0.08767538	0.12319539	-0.2035962
-0.1612833	-0.0291171	0.02501292	-0.1338498	-0.0146016	-0.0508683	-0.0456583
0.05897987	-1.7649398	-0.9477418	-1.6398703	-0.0997156	0.2630326	-0.4381689
0.15643051	0.06955828	-0.341657	-0.51941	-0.2703139	0.19064685	-0.2421146
0.040554	-0.38961	-0.37429	-0.6439	-0.013938	0.1931	-0.29291
-0.0069069	-0.1944447	0.12346502	-0.0510577	0.09260061	0.00880019	0.05674039
-0.1293553	-0.1476854	-0.1761553	-0.128722	-0.0120912	0.23416571	-0.0532249
-0.0052688	-0.0244536	0.0456492	-0.1092002	0.09362446	0.09409311	0.00688005
-0.022037	-0.16771	-0.20489	0.24822	0.22486	0.049058	-0.10224
0.03992607	0.40468828	0.41421897	0.06975276	-0.1066464	-0.2442678	0.15003994
-0.1467108	-0.228047	-0.0090954	-0.078225	0.03352991	-0.1935295	-0.0293819
-0.064835	-0.12163	-0.35534	-0.13876	0.10784	0.20643	0.025852
0.03490215	-0.1090226	-0.249301	-0.10833	0.28553241	0.1597543	0.08172954
-0.1045667	-0.5225844	-0.5202359	-0.6999348	0.03127334	-0.1045988	-0.2551425
-0.0588941	0.01856782	-0.1062608	-0.079341	0.04301974	-0.1023988	-0.0812561
0.00937077	-0.3993524	0.02809421	-0.1121508	0.26219831	0.12665848	0.03259734
0.05522651	-0.308649	0.06237599	-0.1750694	0.37724363	0.13095345	0.00991137
-0.0035905	-0.2147336	0.14081947	0.09555223	0.03487785	-0.1262582	-0.098225
-0.043134	0.032818	0.035916	0.055766	-0.17331	0.40059	-0.58846
-0.01063	0.28086	0.33067	0.5329	-0.038874	0.0043623	-0.19839
0.15141	-0.13477	0.38067	0.50207	0.082262	0.30224	-0.17409
0.08534	-0.12205	-0.07157	-0.053374	0.12309	0.18621	-0.12471
0.018881	-0.11217	-0.25932	-0.12038	0.0026552	0.12549	-0.10247
0.036809	0.16006	-0.011401	-0.070455	0.096261	0.24199	-0.083576
0.085112	-0.20915	-0.06888	-0.048244	0.30726	0.51809	-0.090576
0.0237514	-1.0698403	-0.5277685	-0.8835571	0.09819942	0.19949622	-0.1736235
-0.1660235	0.08651492	-0.1305397	-0.0930093	-0.0276931	0.18010982	0.02323685
0.06715388	-0.1358904	-0.0846507	-0.0863767	0.05098465	-0.0224541	-0.1629211
0.19704896	-0.1050749	-0.0716957	-0.4889302	0.0177688	0.20828987	-0.0829614
0.06797474	-0.24984	-0.1725397	-0.4307283	-0.0157713	0.04048551	-0.1235144
0.0092609	-0.0446938	-0.1264628	0.22407193	0.50612531	-0.014927	-0.048595
0.07143291	-0.0862726	-0.0929732	-0.1114782	-0.002649	0.34303675	0.09693668

-0.1343386	-0.1556107	-0.0407748	0.18884014	0.00734283	0.00838311	0.17043951
0.00862519	-0.0326954	-0.1310487	-0.0830767	-0.1405208	-0.0386691	-0.066218
0.12446766	0.0652162	0.09900804	-0.0841503	0.04075388	-0.0290936	0.06061904
0.04637752	0.10249276	-0.0629931	0.06340585	0.02640046	0.01837984	0.01852364
-0.0239852	0.08467229	-0.2860635	-0.015496	0.23524311	0.11731322	-0.0128189
0.0225221	0.04086058	-0.0286062	0.00364617	-0.0273926	0.02267825	-0.1017042
0.11384573	0.07883782	-0.0130962	-0.1106737	0.0691953	0.05619881	-0.0396146
0.06957151	-0.473819	-0.2697688	-0.6131355	0.2585437	0.11278741	0.16444881
0.05789582	-0.1241941	-0.0489485	-0.1384143	0.16024842	0.17771591	-0.0591524
0.03886836	-0.9941064	-0.5834338	-1.1502569	0.01257806	-0.0874149	-0.1377974
0.20419317	0.22808998	0.28419559	-0.1467132	-0.4587775	0.17869655	0.17127976
0.12709179	-0.3183681	-0.0460178	-0.1463175	0.10385652	-0.0571226	0.05010573
0.08069602	-1.4580179	-0.7050627	-0.9428255	0.13982985	0.11940572	-0.1154682
0.10576864	-1.1001695	-0.1925468	-0.4053943	-0.0136988	0.00631464	-0.1294014
0.03804238	-1.1292093	-0.148156	-0.4077714	0.09539175	0.08356942	-0.1154815
0.03864508	-0.0928058	-0.0578463	0.08574816	0.2125575	0.02159525	-0.0160168
0.01878266	-0.0997449	-0.1494635	-0.0332068	0.17747619	-0.1780028	-0.1537229
-0.1784383	-0.0737323	0.11761525	-0.0530276	-0.0089121	0.17459035	-0.0322607
0.08343254	-0.0655426	-0.0276876	0.01809383	0.18960977	0.10626267	-0.1840575
-0.0316495	-0.4906546	0.35158526	-0.1177912	0.28544198	0.15324013	0.03704355
-0.3092816	0.66053885	0.56152827	0.89601613	0.1703464	-0.1115155	0.423982
0.04058122	0.04609411	0.09009645	0.17690637	0.07351372	0.08355699	0.13270647
0.0015409	0.20474	0.24391	-0.16405	-0.15724	0.048	0.015988
-0.0062444	-0.2530482	-0.2208837	-0.1691914	0.37280709	0.24652253	-0.1703034
-0.068786	-0.0021033	0.0070754	0.082013	0.203	0.041419	-0.032627
0.06450773	-0.309427	-0.1785603	-0.0971373	0.16748945	0.11592482	-0.0665124
0.08578524	-0.1010765	-0.0495899	-0.0890462	0.16522987	0.00797685	-0.0958939
0.09667962	-0.1030203	-0.0290327	-0.004452	0.32934444	0.32807085	-0.0685806
0.23409704	-1.139784	-0.3403709	-0.6633003	0.24939308	0.00216031	-0.274531
0.053033	-0.043992	-0.10825	-0.011353	0.018164	-0.015172	-0.02125
-0.0615928	-1.7034458	-0.690516	-1.7103895	0.0699843	0.07437759	-0.0153356
0.0087346	0.42118448	0.03502801	0.32030691	-0.2585689	0.04002422	-0.0498965
-0.13676	0.03446625	0.04084947	0.01748511	-0.0163129	-0.1838084	-0.0462054
0.00116146	-0.1512031	-0.0969024	-0.2654807	-0.1178381	0.01013787	-0.1278544
0.19651277	-0.1091227	-0.0107691	-0.1732032	-0.0136008	-0.0892484	-0.0551989
0.05490154	0.21874649	0.15538943	-0.0071757	-0.3249973	-0.0039631	0.08871978
-4.73E-05	-0.0562649	0.06348957	-0.0871943	0.1390628	0.03854425	-0.0120638
0.26709484	-0.2161692	-0.4476103	-0.7236136	-0.0118824	-0.0990911	-0.1975483
0.14625436	-0.1104135	-0.4207738	-0.5974025	-0.213376	-0.0647747	-0.2841767
0.0490105	-0.2507863	-0.4131787	-0.6004325	-0.1711147	-0.0970634	-0.2847728
-0.0148346	-0.0809629	-0.2437291	-0.339042	-0.0633518	-0.0739211	-0.0785159
-0.0878839	0.18219259	0.31077439	0.5658622	0.28893535	-0.0367347	-0.0142252

-0.1236916	0.17963934	0.0268589	0.10359072	-0.0790501	-0.1147291	0.01049694
-0.0144353	-0.1536616	0.01975393	0.05793152	0.02466834	-0.0145303	-0.0248929
0.02618547	0.00322557	0.00478978	0.00113135	0.05626674	-0.0669808	0.01691256
-0.0009372	-0.2810397	-0.2282389	-0.3168876	0.1030193	-0.0526239	-0.0857206
-0.0358047	-0.001651	0.00733512	0.01256171	0.01067085	0.03661642	0.019698
-0.0100306	0.13818006	0.24138837	0.09401581	-0.1001383	0.20160605	0.12097145
0.12278438	0.02640504	-0.0779611	-0.0344693	-0.0411457	-0.071667	0.02153122
0.08086564	0.15105974	-0.0939965	-0.1935192	-0.1628029	0.17267381	-0.0844278
0.05593049	-1.7264283	-0.83921	-1.7394168	-0.1617449	0.11843927	-0.3563303
0.00792456	0.05401013	-0.0561305	-0.1275559	-0.2008893	-0.1695534	-0.2613682
0.05843656	0.02061128	0.00124928	0.02506528	0.13072866	0.02994288	0.04332984
0.07517821	-0.0109571	-0.0527323	-0.1970366	0.07347187	-0.0397394	-0.1449546
-0.068653	-0.02015	0.1120624	-0.0163806	-0.022436	0.06492191	0.08105704
-0.002807	-0.1618904	-0.0870059	-0.1045197	0.00320277	0.05762631	-0.1092696
0.1669616	0.02263534	-0.355708	-0.3077326	-0.0839022	0.04043276	-0.3205977
-0.0588987	0.19546292	-0.2350997	-0.1223803	-0.0702178	-0.1686462	0.09949798
0.01013942	-0.1584431	0.03908369	0.1263531	0.28584036	0.28744663	0.00261731
-0.0498936	-0.0232435	-0.0836969	-0.0113938	0.13010953	0.00297544	0.00329834
0.06263318	0.5506589	0.39859351	-0.0595562	-0.2203185	0.10773622	0.24261517
-0.0416593	-0.1495631	-0.3303085	-0.2003895	-0.0998275	-0.1333516	-0.125024
0.05318106	-0.4588646	-0.4616639	-0.2917591	0.31067044	0.23261829	-0.0726163
0.05177808	0.20231531	-0.0156025	-0.0493422	-0.0242325	0.05725421	0.03423072
0.07413227	-0.436836	-0.1103733	-0.2284835	0.32399652	0.00426256	-0.1376481
-0.019845	-0.0993205	-0.0406589	-0.1034678	0.07563874	-0.0613454	0.02362158
-0.1143831	0.0508572	-0.0848192	0.09770941	0.12749142	-0.1058088	0.23076216
0.03211833	-0.3331996	-0.0748046	-0.4866466	0.15571872	0.27759328	0.0158007
-0.0589074	-0.1915848	-0.0824209	-0.0826357	0.30495341	0.06017459	0.07404608
0.10786328	-0.7414706	-0.572117	-0.9666157	0.2545079	0.01743322	-0.2518038
0.06333051	-0.008399	-0.0297252	-0.1191221	0.06561172	-0.0361835	-0.0100532
0.1312004	0.147989	-0.1021219	0.00820909	0.19790391	-0.030513	-0.0102325
-0.0662182	0.07721441	0.05233929	-0.0660105	-0.2032976	-0.1198241	-0.0264895
0.16298259	0.18656812	-0.0254731	0.15372781	0.0964823	0.02480062	-0.1408889
0.01630653	-0.1633034	-0.0594305	-0.0064053	0.16167894	-0.1069163	-0.11325
0.1241013	0.02510295	0.10686673	0.08779502	0.08573291	0.16706491	-0.0317728
0.25747315	-0.2324103	-0.6321007	-0.9408714	-0.2062797	-0.1464324	-0.3280779
0.04317572	0.0990378	-0.0298863	0.06551199	0.06627689	0.01012625	0.00867421
-0.11705	-0.21997	-0.11352	-0.11548	0.067611	-0.2016	-0.15736
-0.0108496	-0.063868	0.07395337	0.01619733	0.18975193	0.22293129	0.09766686
0.2617215	-0.7655269	-0.6449932	-1.0333986	0.1946636	0.10829219	-0.28997
0.037579	0.094321	0.030877	0.042808	0.24648	0.061627	0.061378
0.15529209	0.03627319	-0.0936224	-0.2672576	-0.0427369	0.13914461	-0.0575123
0.16662074	0.09634397	-0.1676695	-0.1901434	0.13475065	0.15504006	-0.0873857

-0.1007381	-0.0627486	0.1534915	0.08267401	-0.0465652	0.16022507	-0.1423094
0.0059483	-0.0558088	0.23973153	0.09617288	0.09201314	0.08156832	0.01032207
-0.0454102	-0.0186115	0.01501718	-0.0874828	0.40301803	0.09886567	-0.0135951
0.13984932	-0.0343213	-0.0828722	-0.0295736	0.33741712	-0.0045256	-0.0200996
0.26848104	-0.0157488	-0.0372561	-0.1719662	0.24991766	0.35643794	0.0604451
0.04506216	-0.1266801	-0.0776318	-0.0827658	0.31156991	0.02658208	-0.0239898
0.11431971	0.24827683	-0.0054694	-0.0033205	0.01454729	0.1039624	-0.0108584
-0.1075807	-0.3841163	-0.3243317	-0.8375565	-0.015374	0.11531584	-0.0049115
-0.0136769	-0.0345012	0.1013912	-0.0298121	-0.0579845	0.00298947	0.20987094
0.071957	0.066492	-0.10483	-0.16229	-0.029154	0.21766	-0.0565
0.09582371	9.72E-05	0.30131958	0.05266811	0.1703155	-0.0234525	0.14723937
-0.1110854	0.00623026	-0.0473608	-0.0408955	0.13961264	-0.1205504	-0.0426854
0.28752054	-0.1301499	0.13045036	-0.0161354	0.14903902	0.25373941	-0.0147565
-0.0923749	0.21410066	0.06340335	0.179145	0.04583569	-0.1164858	0.03657758
-0.0260779	-0.1154167	0.04562734	0.02145753	0.05128663	0.10386354	-0.0198265
-0.1060633	-0.0444339	-0.0226058	-0.0628108	0.04620777	0.08068096	-0.0273452
0.11253256	-0.0052457	-0.1271205	-0.2531911	0.15686314	0.16026161	-0.1140816
0.04222201	-0.0515987	-0.1674554	-0.0543051	0.14557727	-0.0819604	-0.0818803
0.03767036	-0.0524917	-0.0014969	0.05902006	0.14762387	0.24113595	-0.0436219
0.03977873	0.10959733	-0.2770176	-0.2151561	-0.1462059	-0.0592231	-0.1346975
0.27331729	-2.0680104	-0.7716195	-1.150914	-0.1061297	0.04565625	-0.3080848
-0.1898147	0.0461172	-0.0979552	0.06972432	-0.016177	-0.0267166	0.14407661
0.00670094	-0.0157044	0.05899496	0.10808043	0.04064581	-0.1790277	-0.0139069
0.05200531	-0.0627503	-0.1258717	-0.0739003	0.16346537	0.24169795	0.00328828
0.0848106	0.15700564	0.03330903	0.07315677	-0.0192594	-0.1715914	0.16576477
0.00434834	-0.016017	0.00032631	-0.0485554	0.03108039	-0.1571362	0.18256596
-0.0095049	0.22325952	0.14002433	0.455123	0.10281479	0.01804072	-0.097324
-0.310294	0.25242734	-0.0348716	0.70447996	0.59924906	0.16680605	0.20335854
-0.1646695	0.19904717	0.31213646	1.0187694	0.80044629	-0.0288791	0.42690821
-0.4596722	0.18141483	0.08301724	0.66308315	0.07482303	-0.1053036	0.09561758
0.02015347	-0.0251946	-0.1327618	-0.0051522	-0.0222306	-0.0319643	-0.001013
-0.0549589	0.09632531	-0.0862547	0.11975686	0.16278588	-0.1852131	-0.1778183
-0.0369121	0.17413746	0.12775893	0.35613602	0.23962371	0.22507862	0.48572261
0.00848204	0.33074691	1.0647338	0.20532057	-0.4281121	-0.0472803	0.02980955
0.07953962	0.11304504	-0.0946995	0.05512429	0.05602346	-0.2094255	-0.1165496
0.02772279	0.02924124	0.08597488	0.03990573	0.03336469	-0.0052708	0.11793056
0.20422436	-0.0998483	0.08097966	0.12696056	0.0988976	0.03957768	-0.0941666
0.15335306	-0.2474828	-0.2674947	-0.2483602	0.18439693	0.24049615	-0.0115889
0.45569801	-0.7261379	-0.6661375	-1.184374	-0.141117	-0.1528312	-0.3384336
0.04311663	0.05682215	-0.0098691	0.0073653	-0.0322165	-0.0006513	-0.1485665
0.03961715	0.00239876	0.0924002	-0.1663741	-0.2520445	-0.1177881	0.03959447
-0.0247559	-0.2619345	-0.1288071	-0.1709822	0.23421713	0.18853439	-0.0690368

0.13100323	0.09519203	0.0101996	-0.0142437	0.07698614	0.1517602	-0.0984541
0.17775	-0.15708	-0.51281	-0.44754	0.025133	0.080394	-0.085262
0.07174052	0.11139629	-0.0264463	-0.020403	-0.163646	-0.0604538	0.04344706
0.06967826	-0.0075724	0.21583421	0.14443333	0.03835074	-0.0805429	0.04298595
0.18605749	0.46949982	0.3705009	0.08301984	0.18285441	0.19534121	0.15145171
0.33303462	-0.6829661	-0.1025638	-0.2844549	0.11653541	-0.0740905	-0.1548944
0.02976417	0.21818533	0.29950863	0.25045545	0.03235528	-0.0683855	0.16976783
-0.019143	0.055402	-0.21894	0.017191	0.075375	-0.022135	-0.023722
-0.0093841	0.07739179	0.24284659	0.08491664	-0.3353281	-0.0205891	0.05591403
-0.22218	0.073197	-0.058186	-0.0538	-0.11209	-0.039794	0.043332
-0.2119508	0.44046104	0.33547042	0.5079727	-0.3135884	-0.2547034	0.2551097
0.0357806	-1.3822904	-0.0748265	-0.6701164	0.2067692	0.06126522	-0.0224777
0.03227797	-0.4587002	-0.2114777	-0.3593199	0.1654253	0.16307202	0.01093231
-0.0475529	-0.0767647	-0.0231965	0.08678457	-0.0948623	0.0973594	0.18401609
0.09778186	-0.4900644	-0.5377289	-0.7802527	-0.4587312	-0.3678983	-0.20356
-0.1258779	-0.1880943	-0.0929402	-0.326232	-0.3204371	-0.0200611	-0.1776806
-0.0680398	0.04437401	-0.0791235	-0.0548084	-0.1271851	-0.0398863	-0.0170038
0.11001878	-0.9768132	-0.1854745	-0.6125736	0.04804834	-0.1933503	-0.1161271
-0.0448033	-0.1948928	0.07874319	0.06662592	0.14607721	0.26208101	0.05818618
0.00360875	0.14276862	-0.0148453	0.07967383	0.17712596	-0.0714694	-0.0448406
-0.010586	0.03351774	0.02131804	-0.1397479	-0.1080854	0.00106909	-0.0509433
0.10803314	-0.3412151	-0.3819756	-0.3622626	-0.0100703	-0.0422011	-0.1822051
-0.10357	-0.10931	0.087182	0.10361	-0.013713	0.25758	0.15438
0.01310935	-0.0794134	-0.1519427	-0.0004899	0.12858974	0.02953499	-0.0644393
-0.0463131	-0.0620514	-0.0163774	-0.2394844	-0.1149352	-0.1998875	-0.0188508
0.20265628	-0.110493	0.02271909	-0.0722385	0.17887543	0.08579822	-0.0632058
0.06405008	0.02357341	0.00471182	-0.1280448	-0.1735394	0.09477516	0.01165052
0.11961	0.15529	0.12968	-0.023831	-0.30168	-0.18476	0.091441
-0.0856932	0.10533589	-0.0362295	0.01147096	-0.672824	-0.2093286	-0.4941395
-0.0033297	-0.0723398	0.00098996	0.0442126	-0.235447	0.08030421	0.15707351
0.06662941	-0.2533531	-0.312978	-0.5827501	0.29266781	0.09313479	-0.0973792
-0.0965744	0.11205234	0.20681947	0.08459016	0.05316412	0.21874056	0.23511082
0.061066	0.010335	-0.10234	-0.070803	0.099225	-0.063301	-0.021999
-0.0528115	0.20638907	0.14626747	0.48518262	0.2160386	-0.027864	0.12139131
-0.1748446	-1.2695979	-0.592715	-1.0896353	-0.0084551	0.11332364	0.09453507
0.08556692	-0.3922897	-0.3019728	-0.437671	0.10907502	0.12839403	-0.1222023
0.01927738	-0.0489751	0.25197243	0.23971834	0.16751069	0.18302997	0.06604398
0.00975003	0.02186931	-0.0199448	0.10269951	0.1766387	-0.1023849	0.06239049
0.11736465	-0.0017984	0.07744421	0.02439316	0.13460584	0.00114344	-0.0209282
-0.2192413	0.24524997	0.14672965	0.40346019	-0.1635845	-0.0012712	0.33143241
0.15549532	0.00911449	0.02376458	-0.0999232	0.09389843	0.10796155	0.06674187
0.03605622	-0.152204	-0.0539443	0.02471998	-0.1209244	0.15075205	-0.0833

-0.1409682	-0.5305738	-0.2089011	-0.6054664	0.12402887	0.35748371	0.10231446
0.07238264	-0.0681106	-0.0161809	-0.081417	0.08257431	-0.204116	0.00053853
-0.1567881	-0.0241633	-0.1771244	-0.0242751	0.0222268	0.07201932	-0.0367014
0.04291805	0.23784535	-0.1142176	-0.022166	-0.0713785	-0.1083576	-0.0251806
-0.0795438	0.2596553	-0.0995912	0.13865852	0.27422834	0.11583286	0.03180463
0.05632695	-0.0864636	0.10349202	-0.0852277	-0.0891763	0.0852387	-0.0153158
0.1432564	-0.9612464	-0.6343577	-0.9789189	0.22931174	0.05225079	-0.3816284
-0.0013955	0.04310085	-0.1372764	-0.2535242	-0.020801	-0.0210807	-0.1456122
0.22652382	-0.047586	-0.0521553	-0.1564971	0.10528556	-0.060994	-0.0234898
0.022937	0.10127	0.015441	-0.015509	0.1139	0.015465	-0.017835
0.02965248	0.13254069	-0.0190336	-0.0156096	-0.1278201	-0.0597701	-0.0567538
0.2802647	-1.8696716	-1.2119508	-1.8391272	0.05766626	-0.0644771	-0.3942979
0.05549632	-0.0067953	0.18898375	0.13084925	0.10670435	-0.0736986	0.13093946
-0.022746	0.35129	0.28675	-0.038537	-0.10401	-0.051639	-0.14173
-0.0968645	0.10172267	-0.0917438	0.06982036	0.1737294	-0.039463	0.08769599
0.00818372	0.02479488	0.02712109	0.0199275	-0.0823713	0.04519893	0.00859064
0.0031952	-0.1537768	0.00242358	-0.1487511	0.12564155	-0.0165936	-0.0528919
0.00453475	-0.0363146	0.03479775	0.01621383	0.07643183	0.11875728	0.03177966
-0.0507673	0.17357064	-0.1472124	-0.0960188	-0.286457	-0.0904087	-0.0933915
0.05564506	0.03921677	-0.1681651	-0.0118175	-0.0130281	0.08110834	0.01725262

test_GSE353	test_GSE353	test_GSE353	test_GSE353	test_GSE353	test_GSE353	ref_GSE3537
ref_GSE3537	ref_GSE3537	ref_GSE3537	ref_GSE3537	ref_GSE3537	ref_GSE3537	ref_GSE3537
GSE35371	GSE35371	GSE35371	GSE35371	GSE35371	GSE35371	GSE35371
GEO	GEO	GEO	GEO	GEO	GEO	GEO
affymetrix	affymetrix	affymetrix	affymetrix	affymetrix	affymetrix	affymetrix
TIME:8,ELEC	TIME:8,ELEC	TIME:150,ELI	TIME:900,ELI	TIME:900,ELI	TIME:900,ELI	TIME:900,ELI
2338	2340	2346	2348	2349	2350	2351
0.00874978	0.02981386	0.06037744	-0.0767366	-0.1601704	0.04029076	0.04134728
-0.1159581	-0.4001479	-0.5361335	-0.1641087	-0.2791212	-0.0550632	0.16565532
-1.0058872	-1.3501334	-1.1161899	-0.4595476	0.02036039	-0.2603577	0.21624485
-0.0779814	-0.1528736	0.02294902	-0.0395301	-0.1414906	-0.0059693	0.01336064
-0.0304225	-0.001074	0.14186905	-0.0889056	-0.2624804	-0.0229587	0.17797067
-0.3622345	-0.4219382	-0.3853135	-0.2514021	0.01572323	-0.1500927	0.13956943
-0.2446642	-0.1974378	-0.206724	-0.0947256	0.05948379	-0.0722496	-0.0919167
0.20591489	0.09213549	-0.1696754	-0.2009681	-0.1904378	0.05515138	0.08275346
-1.1273131	-1.4557226	-1.1796023	-0.563823	-0.1739392	-0.23681	0.16569334
0.23397753	0.13150961	0.02382531	-0.2687492	-0.3158376	6.11E-05	0.11861405
0.06148125	-0.0826219	-0.0428575	-0.1378497	-0.312774	0.11017461	-0.0280986
-0.1334394	0.04622448	-0.1172201	0.0359248	0.20825574	0.09768093	-0.1625497
0.16349307	0.15901948	0.20011405	-0.2596769	-0.2828264	-0.0163333	0.14196923
0.01387074	0.04061054	0.09991095	0.15746652	0.03244547	0.20251073	-0.081036
0.03078238	0.10982926	0.2845545	-0.0496646	-0.1568598	-0.0771047	0.01759774
-0.1022305	-0.0928345	-0.2363661	-0.151874	-0.0600301	-0.0536163	0.09140292
0.16851	-0.021436	-0.085028	-0.0046058	-0.093311	0.043874	-0.021131
-0.092192	0.03387106	-0.3232239	-0.2474276	-0.1240253	-0.2205602	0.26936774
-0.1434495	-0.2113803	-0.1022132	-0.1592705	0.00026058	-0.0390899	0.01547618
-0.2606972	-0.2882631	-0.550085	-0.316901	-0.3227013	-0.0924565	0.00883781
-0.0227927	-0.0535793	0.10786495	0.0863232	-0.0200609	0.00716064	-0.0679758
-0.1544	-0.1568	-0.32644	-0.15267	-0.031202	-0.077442	-0.087387
-1.1733811	-1.4934404	-1.406469	-0.5976644	-0.124388	-0.4688936	0.10942339
-0.1379	-0.2153	-0.36413	-0.36754	-0.27191	-0.058599	-0.033148
-0.0676162	-0.1399638	0.00828462	0.05385653	-0.1066141	0.02060076	-0.100566
-0.1881717	-0.150466	-0.0573283	0.05379885	0.01642894	-0.0425573	-0.0100281
0.08739	0.068749	0.067157	-0.1555	-0.23977	-0.011967	-0.029388
-0.1254579	-0.223609	-0.1759757	-0.0836337	-0.0018186	-0.0568179	0.09646075
-0.090981	-0.048702	0.13725	0.010148	-0.12194	-0.029829	0.045227
0.26642686	0.48485425	0.34600511	-0.1129285	-0.1936935	-0.0379169	0.11371975
-0.1098853	-0.0968664	-0.2151207	-0.2327837	-0.0930397	0.00230511	0.10715101
0.44585956	0.36072636	0.08796406	-0.0749732	-0.0838965	0.07749299	0.05908583

0.03221874	0.03007241	-0.0710887	-0.0365452	0.03352135	-0.0742259	0.1043534
-0.1420694	0.01307089	-0.1104898	0.07882453	0.14145032	0.22173762	-0.1235765
0.06265712	0.14861978	0.14981873	0.07466261	0.02865834	0.05804389	0.00449937
0.16900111	0.07779388	0.13635463	-0.0848392	0.08687551	0.00334974	-0.0490667
0.09103599	0.19290072	0.08894161	-0.1240071	-0.1664841	-0.0979812	0.19780194
0.12721828	0.16366212	0.07058499	-0.1076032	-0.200992	-0.2216857	0.24813467
0.39334572	0.50322609	0.49457549	-0.0779044	-0.0063419	-0.080201	0.05607032
0.06436802	-0.0760978	0.22974324	-0.0420713	-0.0072148	-0.0433747	0.04751747
-0.0334434	-0.1241511	-0.1557668	-0.0385993	-0.1384957	0.12896058	-0.1058102
0.03400995	0.08413274	0.00633536	-0.196499	0.08424382	-0.0307893	0.13886435
0.0948176	0.26461853	0.19429598	-0.1944203	-0.1629653	0.00688513	0.14039852
-0.2472508	-0.3088891	-0.2057513	0.04366602	0.09965173	-0.0520597	-0.0119786
0.04874383	0.17058949	0.14154557	-0.1025451	-0.0845123	0.01163273	0.08116874
-0.0453819	-0.1073541	0.02425504	-0.0038907	0.06540171	0.00096379	-0.0954528
0.10816106	0.09751728	-0.006452	-0.1212184	-0.1984894	-0.0224094	0.12329812
-0.0496405	0.11221763	-0.0365262	-0.1997586	-0.1649952	0.04338667	-0.016002
-0.2057117	-0.2992612	0.03880623	0.16206557	0.07819349	0.15692433	-0.2844491
0.09314349	0.13957646	-0.0762345	-0.1009795	-0.1629919	-0.0523283	0.11636559
-0.0471603	-0.0155962	0.03232289	-0.0812982	-0.1218614	-0.0970683	0.08279708
-0.1457263	-0.0662986	0.03513011	-0.0564426	-0.0083762	-0.0427795	0.04926424
0.11645962	-0.0696148	0.06805693	0.00766565	-0.04883	0.02748019	-0.042021
0.19756692	0.11531079	0.29539136	0.11415793	0.22200632	0.26776426	-0.3172629
0.00765906	-0.0150488	-0.13673	-0.0813916	-0.1367863	0.01905296	0.01681232
0.01608307	0.00081476	-0.0996664	-0.1115602	-0.0856111	0.0242411	0.06143428
-0.2069175	-0.3471101	-0.4633137	-0.2960713	-0.2201846	0.02191695	0.04238956
-0.7314783	-1.0029507	-0.7910242	-0.2355886	-0.117462	-0.1973836	0.17461102
0.07315214	0.13512726	-0.0574166	-0.0674688	-0.071112	-0.0297254	0.07924015
0.36789685	0.12446281	0.11506209	-0.0967626	-0.1141833	-0.1388381	0.22347511
0.1280554	0.50815197	0.13161921	-0.008845	0.18552292	-0.1009167	0.18208598
0.08908839	0.10748164	0.17609472	-0.1606034	-0.0587679	-0.0453429	0.20233271
0.29962561	0.44103549	0.3556844	-0.2075519	-0.1438072	-0.0636138	0.24494812
-0.105813	-0.0988183	-0.2106791	-0.1317102	0.01664092	0.01772414	0.06882398
0.08390149	-0.0369362	-0.0928816	-0.0770467	-0.1020766	0.05103896	0.01730331
-0.944322	-1.448152	-1.2361282	-0.4137487	0.13449532	-0.1157962	-0.1156628
0.64038461	0.77170948	0.85437744	-0.1688873	-0.0507319	0.11494974	0.03222529
0.01624434	0.01403229	-0.1098418	-0.05131	-0.1018453	-0.0216097	0.03241983
0.18876696	0.08696533	-0.0037541	-0.0136035	-0.0783683	-0.0135007	0.08404607
-0.0695396	-0.0620035	-0.3746857	0.13936331	0.00163346	-0.0351387	0.00217892
0.16848725	0.17660959	0.08008937	-0.0124141	-0.0944283	-0.1010876	0.12361163
-0.2442201	-0.2162689	-0.2017864	-0.1697936	-0.1490002	0.03115158	-0.008254
0.15467375	0.09188528	0.22590226	-0.0928486	-0.2091747	-0.0459423	0.15617844
0.0687349	0.20072078	0.13064917	0.02881979	-0.0455364	0.12470791	-0.0743758

0.04294011	0.1345332	0.0356868	-0.0406178	-0.0371232	-0.0126873	0.04311248
0.08685504	0.14401551	-0.0666389	0.12827232	0.10702613	-0.1819704	0.13795193
-0.1795965	-0.208553	-0.1632917	0.04850091	0.20133047	-0.1440116	0.07144692
0.17494518	0.03238005	0.12682838	-0.0181038	0.00673499	-0.0330736	0.17433446
0.04009683	-0.0411521	-0.008055	-0.1969909	-0.3064989	-0.1700093	0.1730704
-0.022642	-0.2044035	-0.1027664	0.16103196	0.0303289	-0.0260217	0.00304199
-0.1919253	-0.2036803	-0.0626298	-0.0411514	-0.0655923	-0.0370653	0.03209365
-0.3234788	-0.0888727	-0.050457	0.12908374	0.1179544	-0.1470732	-0.2252732
0.14304934	0.04727156	0.01704085	-0.1335065	-0.1140417	0.04241719	0.08297584
0.10584782	0.12627479	0.02712397	-0.0696046	-0.1100114	-0.0524919	0.13746526
-0.6397772	-0.7401478	-0.9177316	-0.4608752	-0.1594337	-0.1485199	0.14119251
-0.0510017	0.04415431	0.14600479	-0.0897617	-0.1284039	-0.0757682	0.20992271
-1.4545318	-1.9754357	-1.7121765	-0.5979849	-0.2691856	-0.197324	0.2187814
-0.290256	-0.4740862	-0.5852453	-0.0866951	-0.0475821	-0.0024384	0.05270684
-0.65474	-0.73968	-0.66345	-0.22338	-0.075807	-0.085551	0.083541
0.11333663	0.26988354	0.17043624	0.11637051	0.03310174	-0.0199366	0.02349932
-0.1887093	-0.0568085	-0.1250194	-0.126351	-0.0934702	-0.1465768	0.04458138
-0.2132143	-0.1382737	-0.2118312	-0.01551	0.1656732	0.04978113	-0.1058372
0.44374	0.60433	0.26199	-0.15901	-0.20498	-0.040477	0.056787
-0.0305564	0.40409078	0.06918407	0.48043048	0.47065339	-0.0622881	0.21871288
0.08526666	0.12345965	0.16933748	-0.2075718	-0.3146505	-0.1194261	0.14402395
-0.12539	-0.15312	-0.18967	-0.25093	-0.26347	-0.21781	0.088535
-0.0382962	-0.0958294	-0.0748517	-0.3221946	-0.2896804	-0.12613	0.05018664
-0.565591	-0.7732757	-0.6781466	-0.530682	-0.3044808	-0.3126786	0.02577219
0.12288082	0.08570529	-0.0374875	0.18871256	0.16846321	0.12937981	0.00134017
-0.0336034	0.12802462	-0.0816607	-0.0077044	-0.2000456	-0.1621045	0.06849401
1.45E-05	0.14629631	-0.1099199	-0.0278588	-0.1623629	-0.0118116	0.05683722
0.10684974	0.09884464	-0.1511399	-0.1127487	-0.1292395	-0.0923256	0.08438463
0.1254	0.22466	0.39525	-0.23956	-0.26868	-0.010454	0.22982
0.31244	0.21528	0.45807	-0.093848	-0.20146	0.03385	0.23161
0.20312	0.1849	0.15962	-0.038899	0.10203	0.030913	0.17597
-0.031015	0.089786	-0.020307	-0.041077	-0.064869	0.064248	0.013621
-0.066729	-0.023532	-0.044422	-0.17042	-0.28263	-0.081347	0.060037
0.12461	0.17678	0.092364	-0.073898	-0.28735	-0.022802	0.037515
0.12405	0.063402	-0.057756	-0.18468	-0.22972	-0.05171	0.12024
-0.9877497	-1.381215	-0.9123824	-0.4357028	-0.2608911	-0.1544762	0.16351903
-0.0920033	-0.195411	0.12653013	0.13133608	0.15311533	0.09593183	-0.2432963
0.12182462	0.03275434	-0.0013118	-0.0412087	-0.0996282	0.06603127	0.05967119
-0.4435063	-0.3699026	-0.3684847	-0.1196529	0.07340676	-0.0642077	0.14287459
-0.3314433	-0.3681246	-0.3960858	-0.19109	-0.089253	-0.0224135	-0.0257676
0.04577572	0.18970087	0.09623216	-0.3493128	-0.2983938	0.13716832	-0.0682896
-0.0706863	0.09679129	-0.0505944	-0.0975763	-0.0794065	-0.1722371	0.00011518

-0.4011232	-0.3876587	-0.4427714	0.21211908	0.02960779	0.2496977	-0.5496342
-0.1106832	-0.0913821	-0.0026617	0.03240462	-0.0252517	0.08167431	0.011809
-0.0314241	-0.1301825	0.03993531	0.06010704	-0.0677615	0.0290673	-0.0196542
-0.1244871	-0.1622559	0.2780695	-0.2754392	-0.270599	-0.3012679	0.32074199
0.06703786	-0.1709459	0.11910023	-0.1030368	-0.1767782	-0.050546	-0.0483501
0.08123726	0.15013011	0.10553806	0.01039158	-0.0203543	0.00147222	0.05064441
-0.0370616	-0.0220567	0.01078604	-0.1714479	-0.1939895	-0.0598275	0.18806603
-0.4424928	-0.5732654	-0.6970242	-0.4157395	-0.1758001	-0.0789522	0.15345479
0.13591133	0.00133606	-0.1572697	-0.1572394	-0.307132	-0.21674	0.08134597
-1.0770951	-1.4494003	-1.2323938	-0.6634223	-0.2536783	-0.0323564	-0.1423346
-0.4884026	-0.177507	-0.5340634	0.4283444	0.72970582	0.11923662	-0.2157637
-0.0708419	-0.0510213	-0.3234604	-0.2651021	-0.1869798	-0.0740156	0.23656654
-1.315358	-1.8512151	-1.0508161	-0.4776994	-0.1435174	-0.2983605	0.15491361
-0.5425742	-1.2728509	-0.4569121	-0.2043008	-0.0999861	-0.1585676	0.14511491
-0.5193504	-1.0856048	-0.4061076	-0.2320486	-0.1630216	-0.1207708	0.13127335
0.15092162	0.14811835	0.04687403	-0.2071394	-0.1670397	-0.0007906	0.08848243
-0.1429898	-0.1074986	-0.1769292	-0.0861656	-0.1918062	0.0813893	-0.0256663
0.25313455	0.44453915	0.29792331	0.18673432	-0.0011685	-0.044778	0.17698464
0.12778181	0.17173735	-0.0513161	-0.1825794	-0.1848549	-0.0348301	0.08014051
-0.1057416	0.25654763	-0.2672458	0.13846329	0.03862789	0.00832891	0.00261605
0.28010717	0.43375506	0.99592076	0.3641072	0.16497749	0.27383545	-0.2767132
0.22986352	0.17730914	0.17706758	-0.0600418	-0.1358439	0.03747252	-0.0636111
-0.20458	0.086727	0.15579	0.19107	0.33219	-0.25038	0.26294
0.01480962	-0.009055	-0.0732034	-0.2323192	-0.3308033	-0.017981	0.07781531
0.30054	0.25562	0.13727	-0.084084	-0.083573	-0.0029911	0.11633
0.00256424	-0.1009553	-0.2134971	-0.3404363	-0.3675995	-0.2421643	0.35719153
0.03417645	-0.0551738	-0.0920188	-0.0100769	-0.0797947	0.01474631	0.01765657
0.18743489	0.0549098	-0.0200926	-0.1896066	-0.3395525	0.12573991	0.16848523
-0.9081159	-1.3692099	-0.9476171	-0.3920058	-0.2028149	-0.302685	0.32996134
0.23591	0.32678	0.12647	-0.10731	-0.073019	0.074646	0.052878
-1.8274276	-2.1113541	-1.922797	-1.0341711	-0.3719696	-0.2870727	0.33639643
0.15711189	-0.1017065	0.29759656	0.14044083	0.19844162	0.04078817	-0.0668576
0.06202261	0.13493833	0.03829909	0.04133716	0.01196497	0.01016738	0.0364918
-0.1157839	-0.1514142	-0.1521125	-0.3901984	-0.3705106	-0.1968582	0.1204207
-0.0983564	-0.1228532	-0.2344234	-0.2379029	-0.1333731	-0.0475591	0.29050827
0.00884587	0.12655928	0.01628241	0.25376032	0.36514426	-0.0704688	0.10260297
0.01763805	0.11274844	-0.0257514	-0.0412036	-0.0084643	0.01140373	0.0408152
-0.5533932	-0.8050113	-0.823488	-0.3677937	-0.1051464	-0.4704239	0.23778122
-0.5652291	-0.2989125	-0.5494266	-0.2835605	-0.1638893	-0.2540483	0.19596697
-0.5841257	-0.9681964	-0.6950031	-0.2710206	-0.0622725	-0.2524826	0.11834505
-0.5114852	-0.4913476	-0.2792861	-0.224419	-0.0756545	-0.3245637	0.13748747
0.52014859	0.63794522	0.51451557	-0.0017612	0.00074708	0.01422399	0.12215603

0.13796453	-0.1364324	0.03971639	0.09862829	0.01147852	0.15305969	-0.1243495
0.10501683	0.11785901	-0.0637391	-0.1116876	-0.1374579	-0.0729224	0.11249775
0.13646152	0.02775016	0.03731179	-0.1167422	-0.0199051	0.06469716	0.03896562
-0.2411409	-0.3683928	-0.2325357	-0.3675291	-0.3108942	-0.2156064	0.15620469
-0.0823117	-0.2265616	-0.0987737	0.09215841	-0.0999685	0.05727337	-0.1925101
0.0970438	0.03525392	0.06044527	0.17925727	0.10369268	0.21988768	-0.0154193
-0.0336334	-0.0768986	0.03358165	0.0261983	0.1180456	-0.0005368	-0.0256046
-0.1828579	-0.3427663	-0.0339976	0.27122997	0.13565985	0.06766366	-0.195875
-1.5575898	-2.3158883	-1.6418484	-0.5961177	-0.3082778	-0.2232965	0.12382416
-0.1174179	-0.1460511	0.15172517	-0.0143387	-0.0686676	-0.0543544	0.26238711
-0.0246569	-0.0042256	0.02077512	0.10339858	0.05351814	0.10853338	-0.0097441
-0.2234958	-0.2548332	-0.2021154	-0.0983707	-0.0391854	-0.1051215	0.07520295
-0.0412766	-0.0069312	0.10326086	-0.0821192	-0.0287417	0.02427162	-0.0344927
0.05058473	0.01200858	-0.0483544	-0.2228202	-0.2636652	-0.0812573	0.14771079
-0.1332491	-0.1288401	-0.2494804	-0.1963288	-0.1939328	-0.2865065	0.17873598
0.06513551	0.21554297	0.07539473	-0.0032404	-0.0247134	-0.1313265	0.13747499
0.06712579	0.20529171	-0.0481202	-0.055181	-0.0905728	0.04778447	0.01831762
0.01920701	0.10618171	0.08592578	-0.0167588	-0.0913915	-0.1351874	0.03006239
-0.1671595	0.17464734	0.05405521	0.43906091	0.59489601	-0.0574194	-0.0672129
-0.2732055	-0.293184	-0.169852	-0.289637	-0.2308252	-0.0966093	0.02028078
-0.4811882	-0.6293395	-0.7064841	-0.6222199	-0.3429136	-0.3684061	0.16995342
-0.003636	-0.1200266	-0.2072259	0.0872086	0.19483457	0.02916983	-0.0574941
-0.1437958	-0.355529	-0.1778264	-0.1872253	-0.1656442	0.08036728	-0.0036335
0.11526688	0.01794321	-0.0241287	0.0056438	0.12484588	0.074375	-0.1340126
-0.0254438	0.2053924	0.15262587	0.25163721	0.2382829	0.30135216	-0.2976491
-0.22109	-0.3016753	-0.5707124	-0.2098013	-0.1679967	-0.0759651	0.20635386
0.00269653	0.09912942	-0.05786	-0.0482789	-0.0332273	0.0803457	-0.0094002
-0.6187811	-1.1037417	-1.0112604	-0.6601456	-0.4131795	-0.2453744	0.24676923
0.12021002	-0.045184	-0.0495887	-0.036928	-0.0538024	-0.0430102	0.09474046
-0.0057566	-0.2355913	0.04815751	-0.1271866	-0.1628956	0.05787191	0.04109609
-0.0297754	-0.1329474	0.07288871	0.11698945	-0.0817931	-0.0117754	-0.1415094
0.36713356	0.09039376	0.07126708	-0.2580138	-0.2329934	-0.0335577	0.28947903
0.07098903	0.20717244	-0.015611	-0.0891837	-0.0197722	-0.038914	0.12569996
0.10758249	0.03063512	0.06492802	-0.0021802	0.00148824	0.07015887	0.05910883
-0.7052708	-0.7044346	-1.0027023	-0.7278003	-0.3740676	-0.4632471	0.23013875
0.15988051	-0.0859861	0.19087887	0.00979422	-0.0457203	0.06958551	0.04141227
-0.25279	-0.048169	0.047381	-0.087498	-0.03124	-0.052527	0.048937
0.09890258	0.06585942	0.06597453	0.0194618	0.02471863	-0.0695739	0.17785746
-1.0116588	-1.4337178	-1.1853486	-0.530268	-0.1032485	-0.220672	0.15291428
0.081097	0.066052	-0.060818	-0.086871	-0.089667	-0.046721	0.051193
-0.1968126	-0.0150244	-0.0693646	-0.0190496	-0.0150925	-0.1518179	0.12212482
0.00607207	0.00511904	-0.0899359	-0.0612047	-0.0172719	-0.0831727	0.19656355

0.15289076	0.09865089	0.03237469	-0.0859894	-0.2517588	-0.0155945	-0.0025721
0.01409893	0.18628438	-0.0360787	0.06626112	0.04893256	0.09733004	-0.0181196
0.36395022	0.42505889	0.27138572	-0.0203176	0.0500527	0.18990642	0.01526314
0.03691258	-0.0829579	-0.2012706	-0.1385916	-0.1291513	-0.0979554	0.12106054
-0.0169646	0.02803684	-0.1166554	-0.1636644	0.00259194	0.01722238	0.05424577
0.13437803	0.08255485	-0.0541116	-0.1198726	-0.131676	-0.0447796	0.15571476
-0.0750442	-0.0496088	0.02253999	0.03666365	0.08840138	-0.0225853	-0.0100461
-0.4929375	-0.2718609	-0.635198	-0.3881521	-0.1645296	-0.2260109	0.25460455
-0.2203662	-0.2198553	-0.0978487	0.03893146	0.03607395	-0.0644264	-0.0039386
0.14645	-0.021746	0.0044018	-0.05163	-0.071615	0.13269	0.01356
-0.0548128	0.32626909	0.35018656	0.19751773	0.01694813	0.06872522	0.07891337
0.05651722	-0.056898	-0.0080345	0.05352051	-0.0049332	0.01927145	-0.027631
-0.1268965	-0.0245991	-0.0112589	-0.0687952	-0.1658894	-0.0703319	0.30286491
0.38339566	0.36817858	0.27305269	0.09599167	-0.000186	-0.0174201	-0.0292693
-0.0099954	0.17224157	-0.0358239	-0.0017798	-0.0318837	-0.061226	0.10073202
-0.115958	0.008181	-0.0474985	0.14358845	0.13191013	-0.1878764	0.11338701
-0.1256104	-0.0895987	-0.2001536	-0.1144637	-0.0207574	-0.174623	0.27022274
0.10678588	-0.0343232	-0.041729	-0.1808512	-0.1759278	0.00508509	0.1058919
0.09441591	0.04223338	-0.1239988	-0.0934118	-0.0572104	-0.0092159	0.08734376
-0.2242593	-0.3984797	-0.2652054	0.00056229	0.02623125	-0.002942	-0.0908455
-1.7367995	-2.6909496	-1.5395663	-0.5066991	-0.1745369	-0.3455144	0.14533231
-0.105497	-0.1389226	0.07064546	-0.209862	-0.1608973	0.01615973	-0.0646105
0.09420302	0.12850405	0.02016365	-0.0558797	-0.0527245	0.07067906	0.05728273
-0.0393156	-0.1101294	-0.1707361	-0.0034271	-0.038473	0.05892748	-0.1113681
-0.0048264	0.03345461	-0.0682244	-0.0101417	0.08449291	-0.0292342	0.08213019
-0.0151205	0.01917623	0.10573671	0.14965671	0.01824171	0.04906204	0.04902808
0.18843468	0.62903595	0.37142538	0.07889818	0.01938615	-0.0504562	-0.0015506
0.61061402	0.97247795	1.2311336	-0.2466853	-0.2410012	-0.0598716	0.22681396
0.67060427	0.95987578	0.8290867	-0.3229076	-0.2776537	0.19209826	0.18176587
0.51871016	0.8643934	0.5792252	-0.2418814	-0.3179616	-0.0568082	0.07419754
0.24243188	0.41431529	0.03150855	-0.0496255	-0.1749782	-0.0229046	0.10329608
0.10415019	-0.3542383	-0.1411803	-0.0895172	-0.0996218	0.04397314	-0.1311662
0.58366815	1.0990735	0.6196226	0.04827835	0.03113006	0.08358337	0.01769438
0.08344063	0.71085378	0.0707375	0.25116404	0.5274095	-0.0445682	0.09387447
0.30045933	0.32611119	0.1371056	-0.0600886	-0.0190208	-0.0785219	0.17023302
0.14486525	0.12461302	0.03967865	0.03288214	-0.0448746	-0.1040501	0.04417074
0.24210521	0.16866286	0.02829227	-0.1552883	-0.1896787	0.0482792	0.31626172
-0.2027014	-0.2287864	-0.3998743	-0.2591355	-0.1126972	-0.2465275	0.12579013
-1.1251538	-1.6910566	-1.5467179	-0.4396095	-0.0296492	-0.3037642	-0.037078
0.07259084	-0.0566849	0.08559826	0.02083831	0.012598	0.03690669	0.01158625
-0.1628428	-0.2467053	-0.0520429	0.27608604	0.37901983	0.03434777	0.0413157
-0.0366364	-0.1893095	-0.1377514	-0.2675452	-0.3294704	-0.1580739	0.1223049

0.0074655	0.06955425	0.01823771	-0.0756699	-0.1108619	-0.1202938	0.23827313
-0.3424	-0.4747	-0.55496	-0.35703	-0.14953	-0.081219	0.1031
-0.1419702	-0.1106561	-0.0796097	0.20408698	0.22115834	0.07213201	-0.163467
0.14517278	0.10146448	0.22739893	0.11274652	0.02530438	0.16852335	-0.1132447
0.17938	0.3752076	0.34325397	0.09381371	0.15058559	-0.3270544	0.28227954
-0.4819248	-1.0087978	-0.747743	-0.1108943	-0.0025362	-0.1768072	-0.0010945
0.31227617	0.2644902	0.27365437	0.15023388	0.08277483	0.08407868	0.04098051
0.1241	-0.022722	0.136	-0.090207	-0.19159	-0.023301	0.12703
-0.2416025	-0.3364697	-0.0819449	0.19892584	0.14461797	0.15185011	-0.2274962
0.16817	0.48403	0.093069	0.17821	0.039983	0.0067361	-0.1742
0.19587062	0.01280046	0.51198834	0.22834827	0.06140344	0.24266591	-0.2472935
-0.8787301	-1.1746387	-0.7990889	-0.5834349	-0.4774179	-0.1003776	0.02859716
-0.378124	-0.4818827	-0.5999282	-0.4393246	-0.1661892	-0.1686868	0.07002985
0.07327966	0.14142294	-0.0688094	0.09685595	0.07421267	0.05978369	-0.0677956
-0.7208243	-0.8739169	-0.6498447	-0.3942552	-0.2663704	-0.2594847	0.45056762
-0.3461884	-0.3952398	-0.4281704	-0.3226992	-0.0368794	-0.0663574	0.22155851
-0.0167767	-0.1718179	0.0015111	0.02736861	-0.0050996	0.00310724	-0.0842382
-0.8882273	-1.2642504	-0.7620626	-0.4399602	-0.0595787	-0.1049563	0.06856195
0.05242501	0.35805348	-0.0606442	-0.0046524	-0.0146944	0.11666576	0.0591559
0.05934212	-0.2321414	-0.021595	-0.0164165	-0.0612452	0.01453687	-0.0801501
-0.0017744	-0.0566447	0.00518454	0.14409298	0.09052217	0.1141146	-0.131711
-0.385419	-0.6136911	-0.4294361	-0.2817462	-0.1728226	-0.1218424	0.0828187
-0.014322	0.024175	0.08582	0.066792	0.089634	-0.097249	0.117
0.01842097	0.041896	-0.0416321	-0.1646471	-0.130694	-0.0011466	0.08309205
-0.1364747	-0.2126055	-0.1466913	0.06694284	0.12581802	-0.0261718	-0.038179
0.16523095	0.10699086	-0.0450761	-0.0565647	-0.0394483	0.01337523	0.1082724
0.07060804	0.01055696	0.0324927	0.10855644	0.08980082	0.04432165	0.10666215
0.2123	0.17635	0.17324	0.012863	0.090683	-0.11394	0.35278
0.33222627	0.20952273	0.22443612	0.06911911	-0.1729113	-0.1175386	0.01149996
0.17959922	0.05015279	-0.0686855	-0.1463004	-0.2264028	0.07556241	0.1456804
-0.1746779	-0.184819	-0.4554113	-0.3555918	-0.1734768	-0.0642288	0.14096356
-0.0601726	0.09318693	-0.118257	0.29304822	0.31595102	0.05532113	-0.1463367
-0.034421	-0.036689	-0.14713	0.077496	-0.012042	-0.19018	-0.053046
0.23790938	0.22104203	0.39605753	-0.3733867	-0.3357759	-0.18267	0.08327271
-1.317263	-1.3926043	-1.196552	-0.5860889	-0.1962589	-0.2890536	0.08948893
-0.4626391	-0.5789728	-0.6531687	-0.4988976	-0.2583391	-0.3205832	0.17642269
0.20766102	0.2446324	0.05108198	-0.0104749	-0.1156091	0.04326536	0.07911057
0.18019543	0.21203185	0.16681925	-0.0004096	-0.0310639	0.12356451	-0.089782
0.14635616	0.14603109	0.0123293	-0.1486639	-0.0760083	-0.0737186	0.14069173
0.1288897	0.22317583	0.42786187	0.26459252	0.20926826	0.10543491	-0.3516023
0.0364883	0.02441889	-0.2014715	-0.1485164	-0.0422872	-0.0222705	0.05858599
-0.0765568	0.0250998	-0.1345673	-0.1005999	-0.0952794	-0.0210833	0.06599504

-0.2812466	-0.4149205	-0.4852616	-0.4996225	-0.3579909	-0.1092279	0.11512345
-0.0269852	-0.0452287	-0.0683163	-0.0906597	-0.0701053	-0.0815618	0.17751115
-0.1941619	-0.1380944	-0.098678	-0.0374918	-0.1205089	-0.0677506	-0.0597966
0.06358983	-0.076812	0.1307558	0.13180857	0.17224911	-0.0836449	0.00641138
0.19937066	0.11435162	0.25936766	0.01145937	0.15594698	-0.0740753	0.02191661
-0.095285	-0.1244696	-0.0476601	-0.0168858	-0.057168	-0.0497905	0.09709425
-1.0214896	-1.1935886	-1.0753421	-0.8777358	-0.665733	-0.3340952	0.23855199
0.02627347	0.03574773	0.01828772	-0.0711614	-0.125513	-0.0934014	0.12530665
0.11435093	0.02454036	-0.0605708	-0.0223121	-0.0401991	0.07472169	0.03880068
0.074557	0.059767	-0.027425	-0.041101	0.021753	-0.17235	0.072422
0.0140307	-0.2995009	-0.0561799	0.07284773	0.12334349	0.22377635	-0.1120252
-2.0033775	-2.5855947	-1.9353439	-1.0218547	-0.4502183	-0.5017644	0.33028284
0.1369452	0.22341245	0.12816614	-0.0312411	0.03495392	0.00202277	0.03408262
0.029914	0.53593	0.21542	0.12466	0.35346	-0.056768	0.095104
0.09111912	-0.0561173	-0.1025905	-0.1459229	0.05728738	-0.0589196	-0.057224
0.05135956	0.17175195	-0.014409	-0.1079052	-0.0764025	0.01133414	0.01068046
0.09098204	0.0010207	-0.1577456	-0.1291733	-0.1331707	-0.0010748	0.14011491
0.17825021	0.02742514	-0.0277905	-0.025821	0.02929395	-0.0153799	-0.0026798
-0.2006044	-0.3285678	0.02328427	0.05517953	0.12508254	-0.0003943	-0.1366055
-0.0084491	-0.0309175	-0.0992391	-0.1950557	-0.1013773	-0.1778197	0.18037871

test_GSE353	test_GSE353	test_GSE353	test_GSE353	test_GSE353	test_GSE353	test_GSE353
ref_GSE3537	ref_GSE3537	ref_GSE3537	ref_GSE3537	ref_GSE3537	ref_GSE3537	ref_GSE3537
GSE35371	GSE35371	GSE35371	GSE35371	GSE35371	GSE35371	GSE35371
GEO	GEO	GEO	GEO	GEO	GEO	GEO
affymetrix	affymetrix	affymetrix	affymetrix	affymetrix	affymetrix	affymetrix
TIME:900,ELI	TIME:900,ELECTRIC_SHAM:+1			H2O2:2	H2O2:2	H2O2:2
2352	2353	2357	2360	2362	2363	2364
-0.0883027	0.04069619	-0.1200197	-0.1321434	-0.0955585	-0.2667369	-0.0782221
-0.4003124	-0.1830492	-0.5238449	0.18684958	-1.7601533	-2.4270327	-2.0938907
-0.1728342	-0.1418122	-0.9612073	-1.7615611	-1.8126548	-1.6970873	-1.763538
-0.0436203	-0.0294176	-0.0107949	0.00686838	0.32729218	0.17520513	0.36281797
-0.0298928	-0.014959	0.12166041	0.25378715	0.3232653	0.44115203	0.33043327
-0.0315006	-0.1382183	-0.3224708	-0.4220822	-0.322516	-0.2498523	-0.3101879
-0.0177867	-0.0225775	-0.3200714	-0.2568975	-1.3539537	-1.1172361	-1.3456537
-0.2230373	-0.0981224	-0.277375	-0.1451642	-0.1707191	-0.0782581	-0.1447531
-0.1978579	-0.1391082	-1.0666905	-1.8368055	-1.7440759	-1.5925503	-1.6943246
-0.3376004	-0.226763	-0.0215959	0.00680538	0.06151289	0.18437112	0.0819175
-0.4065766	-0.0651855	-0.0550095	-0.1188558	1.6865562	1.2378252	1.4791798
0.1469011	0.13504138	-0.082217	0.00995738	0.05637706	-0.0347544	-0.1245778
-0.2938327	-0.1489878	0.00958354	-0.0128212	-0.0043015	0.08568963	0.05451618
0.07466446	-0.0749204	-0.1429828	0.4341235	-0.2690489	-0.3198194	0.23244682
-0.2457594	0.02250284	0.04216669	0.0161967	0.30244506	0.09152061	0.2201676
-0.1203586	-0.0407954	-0.1590848	-0.2956415	-0.2564109	-0.0620425	-0.170714
-0.011095	-0.027784	0.13184	0.016522	-0.2918	-0.19885	-0.4181
-0.3975097	-0.1191082	-0.2137355	-0.3665886	-0.4014725	-0.1127489	-0.4152867
-0.0040435	-0.0186523	-0.1671762	-0.2359975	-0.2730739	-0.2458993	-0.2028039
-0.3167663	-0.148476	-0.5990337	-0.7629618	-0.8461514	-0.8741421	-0.8219896
-0.0752754	-0.0665698	-0.0007439	-0.1177147	0.086141	0.0105572	0.08652629
-0.117	-0.1619	-0.35123	-0.4712	-0.40996	-0.48578	-0.48422
-0.4209135	-0.3683954	-1.0732174	-1.9762899	-2.4959612	-2.4462128	-2.6709494
-0.30454	-0.1525	-0.36313	-0.52376	-0.27087	-0.3254	-0.34922
-0.1281385	0.01489419	0.01140871	0.04734024	0.86382601	1.1416191	0.92807434
-0.0981622	-0.0065364	-0.0170689	-0.002343	0.09352747	0.09906972	0.08248001
-0.29644	0.13543	0.026223	-0.093312	0.0066429	0.086683	0.13326
-0.0557638	0.07407634	-0.0154872	-0.3259419	-0.1860154	-0.293644	-0.0528069
0.017972	0.040925	0.10861	0.19146	0.95399	1.1801	1.1126
-0.2282082	-0.0324708	0.17367377	0.5046157	0.03486631	-0.1396582	0.34039505
-0.2348694	-0.1588079	-0.2800543	-0.1043817	-0.0848661	-0.1517831	-0.1818178
-0.0365518	0.0130238	0.09978803	0.08999177	0.04103616	0.01971629	0.22506307

-0.0475385	-0.0896483	0.13573443	0.05536902	-0.2014592	0.08375301	0.0272026
0.04424877	0.08086998	-0.0048973	0.03582699	-0.0960092	-0.1184297	-0.1001932
0.12363255	0.1325247	0.23599052	0.18090073	0.07921282	0.16281368	0.11598493
-0.093239	-0.011204	0.04044956	0.07058861	-0.0599083	-0.2790572	-0.1538431
-0.2914368	-0.0599983	0.03208615	0.14619952	0.1558791	-0.1022573	0.16851689
-0.3442021	-0.042807	0.11785763	0.17317031	0.11965927	0.01929785	0.26213711
-0.1482236	0.00462283	0.4125157	-0.0768606	-0.2419256	-0.4339751	-0.0648954
-0.1698453	-0.1113752	0.21865114	0.09568242	-0.1500099	0.11971142	-0.0172463
-0.1639443	-0.0329312	-0.2524433	-0.1410932	0.11143878	0.0566184	-0.1879834
-0.2434949	-0.0901093	0.0576317	0.09418937	-0.0542727	-0.156528	0.01362507
-0.2154686	-0.1947534	0.02999979	-0.0141928	0.45935581	0.65153446	0.44340511
0.09630482	0.11014015	-0.2701076	-0.3084976	-0.3858543	-0.6157468	-0.3874179
-0.0777785	-0.0869053	0.09376194	0.04041848	0.14334987	0.06623757	0.27132056
0.08167937	-0.1441367	0.06959416	-0.0143976	0.05339768	0.12900581	-9.99E-05
-0.2222681	-0.076027	-0.0353502	0.02863767	0.09798402	0.26238304	0.19039317
-0.1915196	-0.1433094	-0.2266691	-0.1737788	0.08769789	0.30172429	-0.0382101
0.12162949	0.05148754	-0.1039364	-0.1762277	0.06454928	0.12071895	0.06556876
-0.1112894	-0.0689826	-0.0603479	0.28458824	0.31741091	0.46328483	0.40679442
-0.1738135	-0.2304316	0.10941561	0.09630328	0.36034248	0.39121655	0.33049722
-0.1281398	-0.0872504	-0.0873618	0.05442097	0.00289223	0.23511498	0.08781745
-0.0804827	-0.1271917	0.07996461	0.02222797	-0.0819733	-0.1334332	0.0186026
0.15714355	0.25723338	-0.192716	0.12867153	-0.5075112	-0.8558044	-0.6180109
-0.2521576	-0.0366907	-0.1477115	-0.0338209	-0.2499569	-0.4054246	-0.2760573
-0.0761717	-0.076545	0.05105144	0.11218945	0.06350526	0.2767807	0.11692286
-0.0899561	-0.0506117	-0.514718	-0.5723403	-0.5363037	-0.4882212	-0.5425252
-0.1056497	-0.0672861	-0.4033971	-1.216505	-0.9616411	-0.915891	-0.8951163
-0.0364079	-0.0226772	-0.0244077	0.14494026	-0.0249674	-0.0050915	0.06263562
-0.1533447	-0.0468124	0.26858276	0.30994677	0.14513755	0.61804733	0.33260831
0.38221946	0.12506822	0.32061633	0.17802197	0.03881172	0.40323877	0.32451585
-0.1182889	-0.06504	0.22740255	0.33829776	0.01767932	-0.0178295	-0.0387248
-0.1041773	0.01364261	0.34558905	0.49164166	0.57674847	0.71057095	0.72313613
-0.051846	-0.1053152	0.04452869	-0.1353243	-0.2692457	-0.0429141	-0.1785097
-0.2370528	-0.0239139	-0.0430199	-0.0575515	0.03270783	0.20344063	0.044028
0.03540273	-0.1769324	-1.1074937	-2.181239	-2.0897953	-1.8504684	-1.9262394
0.07378279	0.10589834	0.66900983	0.34032425	0.06525854	0.06813146	0.00803018
-0.2129894	-0.2603196	-0.1916994	0.06484508	-0.0135412	-0.0252292	-0.0099411
-0.0715225	-0.0417797	-0.0406994	0.15857008	0.17550548	0.18290137	0.22266645
-0.0688423	0.03542283	-0.156455	-0.0205174	0.04099402	-0.0730337	0.09468118
-0.0795394	-0.0270997	0.1451339	0.23533079	-0.1472186	-0.1636912	0.05895474
-0.0994311	0.0660384	-0.2505503	-0.2402484	-0.1298646	-0.1971289	-0.1631868
-0.0052422	-0.0977701	0.22641207	0.28839395	0.18289598	0.32638078	0.31453572
-0.0540481	-0.0451719	0.03693313	-0.1700337	-0.20052	-0.3006133	-0.2172659

0.00935405	0.03022258	0.04144735	-0.0981216	-0.0210986	0.07825293	0.06623185
0.02263034	0.04647315	0.0768155	0.35596492	0.2572618	0.4362473	0.45143241
0.21549977	0.12583239	0.04935827	0.00461716	-0.1447729	0.24985882	0.07797709
-0.0638645	-0.0425732	0.17242825	0.10622864	-0.2377209	0.02895412	-0.3175965
-0.2014954	-0.2356319	0.035564	0.11431686	0.20027431	0.28264635	0.26557281
0.00215582	0.15171706	0.08797316	0.09944897	0.04738768	0.11016096	0.00489421
-0.1311536	0.09290784	-0.2388789	-0.2065306	-0.3754411	-0.7165236	-0.3613807
0.01339593	0.0814508	-0.2191889	-0.0826405	0.6189047	0.2972797	0.39201583
-0.0734508	0.05188907	0.04236807	0.2338842	-0.0071948	0.07809601	0.12449558
-0.1154708	-0.0706402	0.03480462	0.07118025	0.06605818	0.1787894	0.12794661
-0.191634	-0.1718732	-0.7705097	-0.9709134	-1.0589366	-0.952129	-1.1347961
-0.1099883	-0.0652454	0.10976366	-0.0039098	0.06934298	0.16290975	0.29018157
-0.4273944	-0.1720721	-1.4991948	-2.2944253	-2.7439958	-2.7342505	-2.9528033
-0.0307054	-0.0135055	-0.1495479	-0.4832027	-0.4763154	-0.3351291	-0.4380791
-0.10499	0.02781	-0.46635	-0.85658	-1.6517	-1.6008	-1.9049
0.0828854	0.07577641	-0.043548	0.09077145	0.12150289	0.10407837	0.24941958
-0.1881612	-0.0777119	-0.2719425	-0.2063947	-0.4112393	-0.8843197	-0.2495331
0.13301549	0.06328177	-0.150558	-0.0500971	-0.4007314	-0.2257294	-0.3086456
-0.23297	-0.030409	0.042515	-0.16762	-0.17296	-0.30991	-0.27992
0.78069227	0.55286601	0.56168455	0.46004808	0.31673442	0.51796143	0.49952053
-0.4046326	-0.302942	-0.0788576	0.00668242	0.21429798	0.08799783	0.20429996
-0.42799	-0.21964	-0.094256	-0.078555	-0.27067	-0.45025	-0.20373
-0.3645106	-0.2230298	-0.2780696	-0.236858	-0.300884	-0.6500413	-0.3320889
-0.3419054	-0.2637966	-0.8428222	-1.1658627	-0.9707329	-0.8395568	-0.9196217
0.19568301	0.23416982	0.10170228	0.09567866	0.23370593	0.40075557	0.27163661
-0.2073664	-0.1232251	-0.2801602	-0.1061387	-0.1227729	-0.2420432	-0.2505029
-0.2272287	-0.1127488	-0.2849059	-0.190179	-0.2165563	-0.1554942	-0.1775272
-0.1452847	-0.1687301	-0.0267829	0.18082772	-0.0931394	-0.2630553	-0.1674616
-0.10928	-0.17612	0.23746	0.52455	1.0247	1.0107	1.0535
-0.12486	0.076579	0.46212	0.61632	1.1425	1.6208	1.2485
0.21871	0.053749	0.2878	0.4087	0.049355	0.039336	0.092573
-0.20919	-0.057104	-0.083413	-0.10555	0.0016818	0.01393	0.06356
-0.3471	-0.038736	-0.10951	-0.14752	0.0008927	-0.039269	0.031498
-0.28952	0.035191	-0.084155	-0.07495	-0.19332	-0.20457	-0.010366
-0.2165	-0.05187	-0.23469	0.076735	-0.060952	-0.09876	-0.10023
-0.2936406	-0.2121155	-0.8530674	-1.59314	-1.5976183	-1.4792332	-1.5883445
0.11253931	0.11429813	-0.0559659	-0.0241555	0.10168107	0.08269787	-0.0095111
-0.1811432	-0.0954054	-0.0353908	0.30254063	0.40346904	0.52133001	0.52145745
0.18181643	0.12286242	-0.1716389	-0.2178663	-0.4217185	-0.2805014	-0.2319571
-0.0959703	-0.0096112	-0.3488968	-0.4354165	-0.3501891	-0.1003372	-0.2948485
-0.3260288	-0.0628164	-0.3398113	-0.0282407	0.6635788	1.0612693	0.56526901
0.01755725	0.02251409	-0.0474717	-0.1694106	-0.2787457	-0.4538754	-0.2061082

0.08349928	0.27541631	-0.4080627	-0.1313003	0.0255395	-0.1449224	-0.2030908
0.01714385	-0.0284044	0.00507883	-0.1022181	0.34517854	0.36199499	0.301859
-0.0085665	0.0116855	-0.0606642	-0.005972	0.03078005	-0.0301472	0.00639083
-0.2213441	-0.1957899	0.16197726	0.20700636	0.00050276	-0.1926445	-0.0205253
-0.3092551	-0.1216865	-0.0301821	-0.1282663	-0.0744502	-0.0616111	-0.2233544
0.02443046	0.03329552	0.05237013	0.06636895	0.26935745	0.16500611	0.28105472
-0.186696	-0.1736131	0.17985019	0.11040405	0.12120169	0.02718234	0.13577164
-0.1817896	0.01181973	-0.6417998	-0.8729646	-0.890484	-0.9023972	-0.8359104
-0.3486755	-0.1670646	-0.200537	-0.1256815	-0.1625225	-0.0740862	-0.1113093
-0.3422273	-0.075748	-1.3442495	-2.0465877	-2.148098	-2.0059394	-2.103838
0.94237763	0.46685433	0.00269558	-0.1115057	-0.4435071	-0.3664417	-0.2330466
-0.1929778	-0.0840489	-0.3025277	-0.3758531	-0.3694134	-0.1091638	-0.1597711
-0.2291624	-0.0781776	-1.1916682	-2.3259642	-2.4892151	-2.1937883	-2.321092
-0.1451873	-0.1545815	-0.4691865	-2.3085801	-3.2568685	-3.352423	-3.3345032
-0.1838219	-0.1135923	-0.3439724	-2.2949496	-3.1964884	-3.2238881	-3.1769891
-0.1618893	-0.0746733	-0.0371255	0.06209312	0.14130139	0.15791102	0.19952878
-0.2340011	-0.0409136	-0.0794183	-0.2125937	0.44452545	0.48166799	0.66977308
-0.0709598	0.05562508	0.18235568	0.42471188	-0.0352257	-0.3772448	0.01492463
-0.1248457	-0.1447454	-0.0038011	0.19461222	0.60155767	1.2547992	0.81285422
-0.016457	0.03045082	-0.4798669	0.77441371	-0.1422566	-0.1576933	0.03371748
0.19490578	0.34429133	0.48282586	2.3350417	-0.9931835	-1.0326504	-0.9255701
-0.059316	-0.1127715	0.09784157	0.22728699	0.31388031	0.27984442	0.20456751
0.44527	0.22468	0.40299	0.42737	0.060066	0.45469	0.44341
-0.4635752	-0.195057	-0.1710707	0.13135016	0.04372667	-0.1192875	0.00610791
-0.10059	-0.099932	-0.01947	0.18525	0.39514	0.4297	0.32338
-0.4164902	-0.2566475	-0.0945595	-0.1519436	-0.0275295	0.29299707	0.3462444
-0.0672674	-0.1352114	-0.1404213	0.05357501	0.02488616	0.11542743	0.05976733
-0.3014519	-0.0750645	-0.090669	0.21885602	0.11133241	-0.0666428	0.14597059
-0.14718	-0.2184358	-0.673904	-1.7074655	-1.7242445	-1.6518643	-1.6051166
-0.073516	-0.10582	0.070826	-0.12846	-0.50644	-0.68303	-0.49304
-0.4957318	-0.1428715	-1.8701697	-2.5709368	-2.4852118	-2.5254301	-2.5405908
0.16561536	0.15128533	0.37283286	0.49977051	0.61176985	0.95617307	0.49936814
0.02062615	-0.085048	0.04325706	0.03165444	1.0608193	1.0500608	0.98298445
-0.392163	-0.2585671	-0.347303	-0.3794104	-0.4702391	-0.543841	-0.4930607
-0.1910893	-0.1969408	0.00775124	-0.1171545	-0.3298425	-0.2522658	-0.2380651
0.29280089	0.11564359	0.18854398	0.21166357	0.12307693	0.21508131	0.30658608
0.04618118	-0.0104115	-0.0197728	0.0573248	0.1234701	0.13331097	0.13922288
-0.1980172	-0.2671538	-0.563543	-0.8709794	-1.1056435	-0.9763752	-1.042921
-0.2398756	-0.2032562	-0.2601596	-0.6308302	-0.6093749	-0.4195228	-0.3529615
-0.2340486	-0.0807059	-0.5473553	-0.6318093	-0.6466583	-0.3546598	-0.6683295
-0.1435408	-0.1423944	-0.524768	-0.6632447	-0.8539396	-0.8650623	-0.8758198
0.02757376	0.04103048	0.33647524	0.51032055	-0.1538982	-0.5979775	-0.2324235

0.12252568	0.07041557	0.03879284	0.17748212	0.26382454	0.19919429	0.21292424
-0.228965	-0.030425	-0.003365	0.1709184	-0.0549059	0.00224784	0.12716357
-0.0050798	-0.0396182	0.02497404	0.06951084	0.05018675	-0.0472173	0.13148922
-0.5218051	-0.0618405	-0.2757498	-0.5074174	-0.5643026	-0.4210378	-0.516732
-0.1762495	-0.0892169	-0.0780204	-0.2296306	-0.1582099	-0.3244758	-0.2449841
0.22338127	0.09499271	0.14719381	0.17618504	-0.1456118	0.08566608	0.00566837
0.1176306	-0.0123273	0.07441589	0.05603731	0.03234871	0.21375712	0.14893691
0.12633024	0.12883758	0.03508678	0.0272643	0.417798	0.666904	0.28346842
-0.5155307	-0.1454777	-1.4382584	-2.7783476	-3.032411	-2.8614324	-3.2466833
-0.0771927	-0.0631238	0.23715231	0.23783598	0.31822731	0.57418044	0.36473948
0.029724	-0.0930493	0.17440409	0.12325099	0.28863447	0.30792226	0.13717861
-0.1969032	-0.0671652	-0.0587426	-0.4585789	-1.002938	-0.9762042	-0.9973906
0.00688703	0.07568589	-0.0934949	0.01889074	0.2039231	0.18768513	0.15538208
-0.4166353	-0.0926136	-0.1370865	-0.0682148	-0.063011	-0.069693	-0.13249
-0.1968491	-0.0995217	-0.0506632	-0.1580412	-0.1634427	-0.1727477	-0.1576675
-0.1071184	0.01019586	0.26862073	0.22213348	-0.1886923	-0.0379507	0.13700164
-0.1936647	0.05446685	-0.0630688	-0.2614483	-0.2109582	-0.7343853	-0.4516871
-0.1325449	0.02261417	0.02677425	-0.0111538	-0.3118921	-0.7210152	-0.3301851
0.58194897	0.37323447	0.18840251	0.14313172	0.0488173	0.26328006	0.30295581
-0.228536	-0.1944077	-0.3098125	-0.4534055	-0.3970898	-0.5286832	-0.3643147
-0.5424911	-0.3693118	-0.7715199	-0.817926	-0.8452899	-0.8098985	-0.8783858
0.22022684	0.08065541	0.14972763	-0.1146498	0.2004802	0.24320496	0.01821122
-0.2443269	-0.1123158	-0.2316708	-0.4939111	-0.2333276	-0.2640808	-0.3588113
0.17622773	-0.0094727	-0.0259747	0.00407953	-0.0154281	0.04008754	-0.1149003
0.39346729	0.30250837	0.09809293	0.07420238	-0.2156234	-0.2583849	-0.0431506
-0.0919059	-0.0264045	-0.2830558	-0.3423188	-0.6158557	-0.5826196	-0.5055652
-0.0084896	-0.0067264	-0.0875954	0.14546969	-0.1885643	-0.279932	-0.1485422
-0.4915503	-0.3042753	-0.9725662	-1.3258423	-1.3536906	-1.3822752	-1.3280861
-0.0457085	0.0196975	0.02472248	0.04279978	-0.052983	0.05819174	-0.1064797
-0.1121287	-0.0588734	0.06115771	0.14301177	0.07018057	0.16507786	0.06187616
0.12194028	-0.0127275	-0.0504037	-0.009814	0.10768547	0.22212942	0.00756702
-0.402717	-0.1028783	0.03821863	0.14345146	0.02515389	-0.0368112	0.16357318
-0.0716923	0.03544764	-0.0671449	0.20681635	-0.0360289	-0.2798976	-0.0264982
0.02194594	0.02666634	0.01312021	0.11010476	0.17414574	0.16046289	0.06652179
-0.5442742	-0.34486	-0.747882	-0.8345652	-0.9397046	-0.6436699	-0.896536
-0.0139584	-0.0326554	0.13457561	0.10935323	0.12511717	0.22476086	0.12592714
-0.087377	0.13118	-0.17317	-0.21593	-0.26397	-0.37577	-0.092812
0.01065642	0.07665018	0.15961632	0.03903697	-0.1131451	0.00222854	0.12440957
-0.2947526	-0.2293935	-0.9426174	-1.4457722	-2.2956441	-2.0649628	-2.3611092
-0.086146	-0.15778	0.088068	0.0087806	0.2658	0.53855	0.29244
-0.2101534	0.11176236	0.14929124	0.02449358	-0.1611678	-0.0518815	0.09412237
-0.0176854	0.09280679	-0.0058752	0.12094314	-0.201242	-0.1731941	-0.0065155

0.02788477	0.01196076	0.13921775	-0.0096826	0.23888304	0.17958135	0.23537892
0.15947385	0.04028402	-0.0787367	0.00975112	0.00624919	-0.1718522	-0.0333041
0.00918676	0.02350893	-0.0909419	0.3147254	-0.1050911	-0.1939468	-0.0133208
-0.0969197	-0.2609619	-0.0234668	0.06485364	0.18945679	0.40652937	0.22145819
-0.0554559	0.06504876	-0.0460003	-0.0178308	-0.1752826	-0.1605126	-0.0940646
-0.1853412	-0.0389134	-0.0380313	-0.0132427	0.79481164	0.79249015	0.74146345
0.12321491	0.02500352	0.08727317	0.08150949	0.1596221	0.14814318	0.2361411
-0.1645458	-0.0948047	-0.3773675	-0.60052	-0.6681302	-0.5307786	-0.4109132
-0.043978	-0.0142022	-0.0121666	-0.0534771	-0.2277766	-0.1232767	-0.0055707
0.0010849	0.1242	0.088644	0.097856	-0.091925	-0.14661	-0.060421
-0.0521005	0.09377839	0.22345119	0.13166306	0.41511289	0.45073826	0.42240899
-0.1001337	0.00039541	-0.0111803	0.01940807	0.07187545	0.08283769	0.0395366
-0.2105792	-0.1265444	-0.0783309	0.21939135	-0.1746611	-0.0754158	0.3202055
0.05213068	-0.036399	0.34096043	0.57293365	0.68636718	0.86094722	0.70799597
-0.0435565	-0.0102792	-0.130136	0.03500073	-0.1020264	-0.1354343	-0.0813989
0.16869583	0.08304605	0.17407535	0.2055711	0.00380098	0.16261483	0.04325095
-0.0619334	-0.0066862	0.06920698	-0.0150314	0.01699786	0.23883362	0.09877795
-0.165597	-0.1214296	-0.0518665	-0.0976817	0.00996947	0.08501966	-0.0292295
-0.0451949	-0.1629058	-0.0068732	0.02705579	0.17830979	0.16007361	0.21652314
0.01719852	0.09007931	-0.1276026	-0.3220466	0.15306096	0.13682465	0.01558298
-0.2263916	-0.2759706	-1.194773	-3.6695527	-3.4993162	-3.4429586	-3.5064137
-0.1945695	-0.200619	-0.0901444	-0.1283338	0.15711659	0.03237255	-0.107649
-0.1148458	-0.1829342	0.13619894	0.08746301	0.11337452	-0.0080767	0.0793625
-0.0268053	-0.1064473	-0.1961933	-0.1692398	-0.1511562	-0.2790204	-0.0426798
0.15371619	-0.0046754	0.08138836	-0.0928358	-0.0771722	0.08927466	0.27646167
0.0561805	-0.033204	0.21925211	0.16144383	0.05840896	0.08165356	-0.0566897
0.08650877	0.06792037	0.19982472	0.17569845	0.00867667	-0.0049804	-0.0266374
-0.3705186	0.06565767	0.43747161	0.08753404	-0.2687826	-0.451265	-0.4021244
-0.1050425	0.03231314	0.61827924	0.33818456	-0.1884233	-0.4140652	0.06220124
-0.3289556	-0.0070329	0.34275165	0.05267758	-0.4262414	-0.9452138	-0.3664001
-0.0205378	0.00857511	0.05748236	0.03864476	0.12394478	0.28178237	0.26992865
-0.210742	-0.0804838	0.02132501	0.05945693	0.19586936	0.05294195	0.01187069
0.1557492	0.28936671	0.35319965	-0.4971478	-0.8016386	-0.9417194	-0.7940893
1.0342764	0.39175544	0.49562072	0.72352138	0.14411362	0.24502256	0.35801114
-0.0012196	-0.0250237	0.19186599	0.23647307	0.0547234	0.26524878	0.34587695
0.01537621	-0.049244	0.10158512	0.08501333	0.31920423	0.29293146	0.23973283
-0.1405937	-0.1217883	0.28402463	0.48294077	0.27743556	0.04372519	0.403961
-0.2220244	-0.1626546	-0.2733745	-0.6042126	-0.4205051	-0.4806629	-0.4309188
-0.1847224	-0.2982602	-1.1967689	-2.2292918	-2.064611	-1.8650376	-2.310342
0.05067289	-0.0375093	0.01279509	-0.0943212	-0.0965798	-0.0259297	-0.110164
0.46553505	0.25504231	0.23047068	-0.4115013	-0.4906519	-0.2073875	-0.4178167
-0.498547	-0.0703387	-0.1787849	-0.2262274	-0.2884624	-0.41346	-0.2349066

-0.1912235	-0.2122778	0.03656982	0.01755833	-0.0294494	0.0946762	0.05010767
-0.18181	-0.1727	-0.36921	-0.66669	-0.40666	-0.36117	-0.24289
0.15823234	0.02293513	0.03260005	-0.2442758	0.07859711	0.27989405	-0.009863
0.02199746	0.02063444	-0.0286992	0.05097306	0.29393863	0.30209908	0.26038641
0.12668375	-0.0250844	0.29946997	0.51270002	-0.5134618	-0.2623574	-0.2559853
0.03850972	-0.1664009	-0.548163	-1.1936041	-1.0840773	-1.0825304	-1.170177
0.138392	0.15502319	0.38463309	0.43768511	0.30687934	0.56528381	0.32268117
-0.22215	-0.039235	0.15691	0.2293	0.28621	0.37061	0.26172
0.04084178	0.1057839	-0.0626394	-0.176099	-0.0711681	-0.3631292	-0.1660442
0.047988	0.031505	-0.022294	0.11207	0.24723	-0.029796	-0.016642
0.08831788	0.10836802	0.27355196	1.312713	-0.5692649	-0.7486941	-1.0608619
-0.3723299	-0.2109037	-1.0466256	-1.6892139	-1.8551487	-1.7428173	-1.8645747
-0.2168659	-0.1586378	-0.5670278	-0.8474416	-0.751581	-0.7145191	-0.7606751
0.19486565	0.03567552	-0.098319	-0.0324467	0.05792715	0.0927819	-0.0711066
-0.5336939	-0.1398986	-0.3355377	-0.6173318	-0.775461	-0.6872865	-0.6163229
-0.0878961	-0.0352895	-0.4268807	-0.6142655	-0.6144883	-0.3769567	-0.3929647
-0.0475729	0.03207156	0.01766648	-0.0192472	0.00326406	0.02997677	-0.1145818
-0.0949951	-0.2169914	-0.7696219	-1.5078843	-1.7666102	-1.6309391	-1.8238958
-0.0839522	0.05034104	-0.0650278	0.08962291	-0.1880607	-0.6529952	-0.1433788
-0.1858803	-0.1837162	0.06907138	0.07740763	0.31014744	0.30259531	0.148881
0.2465747	0.07337128	0.01781916	0.07488512	0.17192896	0.30501348	0.02801822
-0.2392045	-0.0891667	-0.4258911	-0.4563251	-0.2387781	-0.1029484	-0.3573207
0.06252	0.13276	0.14906	0.16364	0.24821	0.32797	0.30962
-0.1751261	-0.0647924	-0.0114846	-0.0899293	-0.080257	-0.1080285	-0.0430675
0.14016632	0.08978944	0.00676433	-0.1660814	-0.1965755	0.0234691	-0.0688159
-0.1419936	-0.1906666	0.00027713	0.15610446	0.15739317	0.14552556	0.24115447
0.14760478	0.18710807	0.20880195	0.26261786	1.7211343	1.8923976	1.8131024
0.11736	0.019962	0.46661	0.44702	0.10261	0.36348	0.43585
-0.2266	0.10433198	-0.0234236	0.05852035	0.53032125	0.90328356	0.53701186
-0.1579317	-0.0586117	0.01988194	0.02587125	0.16569918	0.18832248	0.38594285
-0.214719	-0.1111098	-0.4755656	-0.5505386	-0.4293614	-0.3273885	-0.4149802
0.2955994	0.24892023	0.20386545	0.16760743	0.14479175	0.1766506	0.05123856
0.0058301	-0.071687	0.016411	-0.15015	0.095141	0.17587	-0.06532
-0.2653539	-0.3155137	0.06566882	-0.1293999	-0.0457224	-0.0214513	0.0168302
-0.2612834	-0.1733058	-1.227743	-2.0756373	-1.9886999	-2.0473192	-1.9835459
-0.4300069	-0.3406016	-0.6186932	-0.7277026	-0.8165948	-0.8044878	-0.8065737
0.04440528	0.00181222	0.16118516	0.26744531	-0.0590755	0.02562582	0.1249097
-0.0335016	-0.0181608	0.12675873	-0.044319	0.3681263	0.37382936	0.25093551
-0.0226768	0.02119236	0.05204332	0.09354794	0.10196358	0.21509388	0.23155521
0.40680276	0.32271299	0.22273021	-0.2059789	-0.1927749	-0.115645	-0.4770488
-0.0662882	-0.0219009	-0.0338212	-0.1294366	-0.2093288	0.10221698	-0.0513018
0.06034365	-0.0062291	-0.0091606	0.0220616	0.0435253	0.03894489	0.08791586

-0.5221742	-0.1883615	-0.7331988	-0.8091239	-0.889957	-0.9762764	-0.9262971
-0.1382153	-0.3246666	-0.0470344	-0.1576792	-0.200415	0.05177894	-0.0885959
-0.2811565	-0.0126547	-0.1676923	-0.136949	0.15217055	0.11451856	0.02048312
0.16925036	0.06228645	0.2630996	0.03555756	0.01458388	0.31144875	-0.0143917
0.11064101	-0.0058186	0.2332018	0.25078006	-0.1088959	-0.3687559	0.05335485
-0.0254174	0.01147131	0.10320017	0.0180687	0.00160009	0.16748959	0.12919261
-0.7575707	-0.546824	-1.150608	-1.670876	-1.4314301	-1.444464	-1.3103179
-0.1390674	-0.0252756	0.02289555	-0.2314781	-0.1751823	-0.2467562	-0.2529183
0.00605005	-0.0096064	-0.0103595	-0.0220708	0.54742928	0.67328702	0.55338953
-0.030001	-0.063347	-0.039382	0.083214	-0.020452	-0.014247	-0.022218
0.00985431	0.02369822	0.20755279	0.08135946	0.25963144	0.42216878	0.10244719
-0.6622823	-0.4551189	-1.7801217	-3.1580228	-2.9960972	-2.9200306	-3.019628
-0.0034873	0.00718952	-0.0118065	0.12942505	0.05631891	0.17147281	0.02407182
0.43682	0.36911	0.22304	0.43093	0.14217	0.075686	0.24698
-0.0713391	-0.0735676	-0.0927987	-0.1348488	0.07607085	0.06125638	0.11503611
-0.0363591	-0.0340885	-0.0724555	-0.0717523	0.26469217	0.2208089	0.32748394
-0.174236	-0.099377	-0.1400399	-0.2347312	-0.1318574	-0.3153212	0.14293747
0.01677896	0.00274208	-0.0245276	0.0332073	0.19936872	0.36083359	0.1526374
0.05812051	-0.0130786	0.05145401	-0.055221	0.03181384	0.12611854	-0.096173
-0.1069501	-0.0532048	0.09351196	-0.1696452	-0.1157354	0.11757482	-0.011877

test_GSE125	test_GSE125	test_GSE125	test_GSE125	test_GSE125	test_GSE125	test_GSE125
ref_GSE1255	ref_GSE1255	ref_GSE1255	ref_GSE1255	ref_GSE1255	ref_GSE1255	ref_GSE1255
GSE12554	GSE12554	GSE12554	GSE12554	GSE12554	GSE12554	GSE12554
GEO	GEO	GEO	GEO	GEO	GEO	GEO
affymetrix	affymetrix	affymetrix	affymetrix	affymetrix	affymetrix	affymetrix
	CRANBERRY:	CRANBERRY:	CRANBERRY:	CRANBERRY:	CRANBERRY:	CRANBERRY:
2365	2368	2369	2370	2371	2373	2375
0.16516642	0.31733338	0.36520287	0.26058955	0.30660507	0.08928513	-0.0341399
-0.2446811	-0.4101841	-0.3990258	-0.5527469	-0.4495137	-0.5127209	-0.5206856
-0.6816276	-0.8994785	-0.4946523	-0.5044237	-0.3243914	-1.1258061	-1.1914877
0.02816119	0.12766915	0.03445469	0.1155387	0.06969659	0.03733658	0.20664277
-0.1169041	0.00598406	-0.0238798	-0.0384412	0.03037106	-0.0861071	-0.0141573
0.04832851	0.07811417	0.04649914	-0.0018267	0.13011818	-0.1155767	-0.0609719
-0.1498498	-0.3633713	-0.3782444	-0.1135385	-0.2586153	-0.0100704	-0.0900429
-0.619447	-0.3299867	-0.2163848	-0.3150421	-0.1182501	-0.9841178	-1.0554579
-0.3074267	-0.0379337	-0.0268423	-0.0196638	-0.033454	-0.3267862	-0.4339745
-0.1824085	0.08707307	-0.0691686	-0.1547603	-0.0860513	-0.4027541	-0.2617026
-0.0673981	-0.2026694	-0.207046	-0.2125698	-0.1189023	-0.0903063	-0.0706853
-0.6035921	-0.6485734	-0.5428694	-0.290037	-0.5155836	-0.9317493	-0.9436056
0.03667981	0.21275884	-0.0027416	0.20838178	0.08833419	0.09129902	0.04297766
-0.0251969	-0.09319	0.1222057	0.11509814	0.01771445	0.05926921	0.03325651
-0.1038918	-0.112673	0.01551315	-0.1327365	0.10338969	0.01113645	0.02283731
-0.0616781	-0.0643631	0.0138092	-0.0209593	0.05180473	-0.0769402	-0.1106617
-0.50389	-0.79134	-0.67608	-0.69134	-0.67852	-0.75883	-0.73139
-0.0993155	0.06642793	0.05252799	0.0388005	0.06833229	-0.0036304	-0.108249
-0.2170285	-0.2340608	-0.2227844	-0.2472301	-0.2536227	-0.1555844	-0.0704605
0.20959032	0.02935094	0.18222329	0.24113061	0.21421492	0.07981012	-0.0001827
-0.071779	0.17950229	0.06769632	0.07162227	0.14471883	-0.0411511	-0.1235595
0.011998	0.00052689	-0.15529	0.00030871	-0.086632	-0.016494	-0.028035
0.18246456	0.27707295	0.23988739	0.21981264	0.16415678	0.06640183	0.15163805
-0.16717	-0.41353	-0.23031	-0.32507	-0.24094	-0.28322	-0.30371
0.07960953	-0.1559328	-0.0228486	-0.224829	-0.1110605	0.02822749	0.1618722
0.02918374	0.01070607	-0.2649498	-0.0485572	0.02874126	-0.1426017	-0.1225011
-0.36016	-0.33434	-0.41183	-0.24133	-0.21401	-0.62731	-0.58641
0.28236427	-0.3883486	-0.1745436	-0.1995193	-0.1306978	-0.5779112	-0.7857765
-0.0070407	-0.29372	-0.025432	-0.15262	-0.077759	0.15696	0.10151
-0.1055517	-0.2531527	-0.2206889	-0.2478675	-0.1577376	0.09977738	0.11718027
0.10776452	0.07249423	-0.040821	-0.0696943	0.03166287	-0.0816056	-0.1076932
-0.0767053	-0.3251705	-0.2216284	-0.2291489	-0.3853376	-0.1918962	-0.1397963

0.00870263	-0.0471252	0.00102063	-0.0076927	-0.3073532	9.99E-05	-0.0084248
-0.0865463	0.05662669	0.01559117	-0.0408476	0.00674596	-0.1750432	-0.1409511
0.42342466	0.11665122	0.16935313	0.19875928	0.0963957	0.17987648	0.26173512
-0.0003764	0.06315584	0.0448207	0.01789332	0.03431176	0.03622098	0.03350483
0.02055543	-0.033062	0.1136263	0.03505974	-0.0104409	0.0067091	-0.1373861
-0.0973261	-0.0329322	0.00662769	0.0070365	-0.0448488	-0.031682	-0.0269387
-0.0552616	0.06625617	0.0013396	0.00650729	0.05642142	0.04922949	0.05883684
0.13225504	0.14468958	0.075822	0.13101397	0.01198769	-0.0008078	0.00975357
-0.0995034	-0.0185464	-0.0756813	-0.0592294	0.01565438	-0.0796547	-0.0636162
0.11065705	0.1093134	0.07958856	0.11362915	0.08512439	0.12377937	0.02524319
0.18967987	0.36534203	0.29019521	0.31835461	0.54907082	0.42463328	0.44394056
1.4439367	-0.379624	-0.4699109	-0.3905136	-0.6732267	1.5144729	1.1388931
-0.0758911	0.03646459	0.0383733	0.00732255	0.01890009	-0.016544	-0.008205
0.06005912	0.02800531	0.07424602	0.03086761	0.0268534	-0.0634561	-0.027072
-0.1971768	-0.5835135	-0.5006247	-0.2688481	-0.429586	-0.1664785	-0.0978474
0.01739479	-0.1718537	-0.2774745	-0.1187043	-0.1017681	0.21038978	0.20374536
-0.0923927	-0.1333773	-0.0825821	-0.0829871	-0.0210115	-0.1493765	-0.0943123
0.19851978	0.30798628	0.17770498	0.2622065	0.23138388	0.05119802	-0.0719769
-0.3086754	-0.3889213	-0.417373	-0.3515092	-0.3091056	-0.1491818	0.09953374
0.02594994	0.02695448	0.08980033	0.14463956	0.11571679	-0.15315	-0.150706
-0.2101574	-0.3550525	-0.292982	-0.2273931	-0.0841108	-0.33028	-0.3307731
-0.2737687	-0.3073687	-0.3584729	-0.3327362	-0.3322431	-0.378678	-0.1914847
-0.1058801	-0.1298677	-0.0205265	0.00873319	0.02537482	-0.0073252	-0.0009176
0.02612711	0.05101607	0.04062337	0.03959295	0.01535462	0.03897154	0.00151059
-0.0514744	-0.1201743	-0.2954665	-0.2780071	-0.0358347	-0.0917856	0.06419532
-0.6417654	-1.010158	-1.0040285	-0.951771	-0.9455254	-1.3537456	-1.4665148
-0.1186566	-0.4717688	-0.3521593	-0.5604048	-0.5481325	0.38054095	0.11456866
0.04985121	0.00500655	-0.0548656	0.00521441	0.05580933	0.03030918	0.0285633
0.46561135	-0.1356472	-0.1536535	-0.1103678	-0.1894457	0.06726656	0.13984306
0.05557153	0.09153717	0.13566243	0.09241726	0.07663317	-0.0610029	-0.044006
0.5559722	-0.5209378	-0.4204288	-0.4240494	-0.6914465	1.4292504	0.94911025
-0.1366093	-0.077319	0.03534591	0.02194115	-0.0244746	-0.2559933	-0.2658301
-0.0090387	-0.4353398	-0.4070888	-0.2918682	-0.3520196	-0.0759003	-0.1361205
-0.0065287	0.06612537	0.0593268	0.16551874	0.00766015	-0.0100625	-0.064109
-0.2603036	-0.1158771	-0.0008587	-0.0275747	-0.0459225	-0.1056505	-0.1233819
-0.5768184	-0.5886259	-0.4779806	-0.3777909	-0.4507084	-0.0231652	-0.2156688
-0.3650745	-0.232706	-0.1130137	-0.0736198	0.06751458	-0.0689008	-0.0164667
-0.1427301	0.17141327	0.12318574	0.11834121	0.23987473	0.05336652	0.04493087
-0.2197852	-0.5776581	-0.28705	-0.3571288	-0.3560969	-0.1447894	-0.2921826
0.03456678	-0.0804451	-0.2597028	-0.2322258	-0.2096296	0.06472975	0.03162127
-0.0709503	0.05304182	0.00610168	0.15246873	0.0696471	-0.0654132	-0.1052374
-0.1974727	-0.279772	-0.2487431	-0.2171559	-0.2097378	-0.4381124	-0.5303657

0.30652201	-0.4246157	-0.3488253	-0.2611414	-0.067573	-0.5886756	-0.6988669
0.07420659	-0.0179243	0.03885332	-0.1113613	-0.1663289	0.07573003	-0.0267891
0.28001811	0.02621281	-0.0667327	-0.2914191	0.02049958	0.22069846	0.02539721
0.00500703	-0.0468443	0.05300411	0.05566524	0.02542287	0.01650087	-0.0896734
-0.1112193	-0.2051415	-0.0137451	-0.0868043	-0.1106896	0.0377225	0.01037509
0.00105866	0.04704538	0.16868854	0.13973291	0.02171335	0.07121763	0.05836694
0.12824851	0.0875915	0.15783903	0.08111071	0.0287691	0.0713019	0.12235649
-0.1337846	-0.0909489	-0.0936991	-0.1852814	-0.100213	-0.086197	-0.0692951
-0.0423468	0.05856523	0.06452823	0.11497134	0.0230026	-0.0245691	-0.1088754
0.29137188	0.01332769	0.07204798	0.13024207	0.00069877	0.23363863	0.07890744
0.07118127	0.34247234	0.24269611	0.30011907	0.2959599	0.03560871	0.04015717
0.04364777	-0.1036121	0.09789285	0.03325644	0.12956409	0.01480399	-0.0552051
0.01250604	0.20786314	0.09246283	0.16178537	0.13256953	-0.0320982	-0.0203826
0.13585403	-0.0101296	0.03107577	0.04764163	0.13942386	0.010782	-0.104786
-0.020232	-0.20408	0.019873	-0.013396	0.084559	-0.092714	-0.14062
-0.087895	-0.2222603	-0.220444	-0.2093737	-0.2448086	0.1173088	-0.0385154
-0.1590317	-0.1472278	-0.1456589	-0.1042825	-0.1159741	-0.0920268	-0.1478561
-0.0009692	0.22586479	0.01579436	0.10578415	0.0359334	-0.0279232	-0.0489679
-0.047933	-0.0096001	-0.037232	0.0053399	0.016579	-0.10533	-0.079022
-0.1000113	-0.1881715	-0.208628	-0.1730459	-0.1145454	-0.1316155	-0.0255247
-0.1533631	-0.1775732	-0.1938839	-0.2567911	-0.3306507	-0.1194651	-0.1711905
-0.11748	-0.31408	-0.21788	-0.20576	-0.13422	-0.28912	-0.28787
-0.2614591	-0.5150131	-0.4910752	-0.4645723	-0.3525866	-0.4467183	-0.3174898
-0.0380177	0.11161609	-0.0109472	0.01125583	-0.0040261	0.11141355	-0.0493473
0.01770718	0.05278679	-0.0753345	0.02866594	0.02314042	-0.0769871	-0.0300984
0.16781464	0.18194684	0.11400257	0.08807729	0.18447111	0.08718211	0.03970504
-0.2592404	-0.1504484	0.03329052	-0.0528994	-0.145661	0.00141455	-0.1299359
0.36020295	-0.0113234	-0.0550165	-0.0386619	0.01789365	0.37290305	0.38654758
-0.15168	0.056608	0.011856	0.10059	0.042071	-0.045398	-0.048514
-0.052555	0.064349	0.04195	0.062779	0.043101	0.1168	0.16743
0.040286	0.082505	0.043949	0.069264	0.0025624	0.056921	0.12673
0.035087	-0.044158	0.090806	0.00048709	-0.050263	-0.065376	-0.017475
0.0084432	0.036433	-0.067305	-0.0018576	0.035529	-0.0843	-0.081962
-0.092327	-0.033107	-0.033567	-0.0005428	0.0064003	-0.09545	-0.023707
0.084974	-0.01155	-0.034621	0.022628	0.0057017	-0.07942	-0.087702
0.52256614	-0.0497625	-0.1130341	-0.1167398	-0.1761397	0.18865761	0.02432711
0.17449183	0.31393284	0.24086412	0.28948475	0.14640236	0.02589908	0.14091199
0.1384811	-0.1750701	-0.0439267	-0.0722759	-0.045541	-0.024175	-0.0116522
-0.1467777	-0.3669162	-0.4320136	-0.2654223	-0.4195586	-0.1929547	-0.1737931
0.08518379	-0.0965365	0.05230986	0.00796298	0.03824252	-0.178278	-0.2312147
-0.0037094	0.04389395	0.05438399	-0.0390781	-0.028982	0.79869711	0.88968129
-0.3551067	-0.6370927	-0.5093715	-0.5745159	-0.3834342	-0.4634293	-0.600103

0.29414092	0.50088192	0.46344123	0.4610305	0.17212316	0.20748789	0.19731988
-0.1143123	-0.0864667	-0.2176945	-0.2017096	-0.2511344	0.12030257	0.00071318
0.01064632	0.18925684	0.10596772	0.12368408	-0.00204	0.02309783	0.0123025
-0.3236406	-0.2083461	-0.1182461	-0.1622986	-0.0607989	-0.2723275	-0.2936075
-0.3292035	-0.488335	-0.3806258	-0.3635489	-0.1687612	-0.5792031	-0.5063335
0.68774373	0.25674077	0.43059261	0.42967572	0.16566815	0.03323221	0.17199898
0.09938403	0.12011096	-0.0024703	0.05281417	-0.1964511	-0.1105523	-0.0465929
-0.1846375	-0.1164853	-0.1451404	-0.2207367	-0.0922082	-0.2142651	-0.3281022
-0.3120415	-0.2693463	-0.1679996	-0.1036214	-0.0488329	-0.1136315	-0.0362258
0.01342961	0.03219029	0.09514692	0.03119145	0.05502285	0.0848667	0.08231557
0.58773721	0.14804236	0.32823377	0.18843449	0.23329221	0.14091089	-0.0573457
-0.2442305	-0.6247611	-0.6049235	-0.3988952	-0.5385858	-0.0120249	-0.2441265
0.15019883	-0.0477191	0.21800273	0.08140623	0.27413743	0.04062936	0.01082036
0.26083927	-0.2079467	-0.125773	-0.1207647	-0.2185473	-0.0268619	-0.1123911
-0.0191916	-0.6539653	-0.6073098	-0.5948656	-0.5495232	-0.4605639	-0.6972925
0.76398914	0.60352457	0.5332006	0.60278151	0.57947734	0.43562258	0.35470024
-0.2610671	-0.2378577	-0.4015487	-0.2906509	-0.3810016	-0.0642469	-0.2189231
-0.1638315	0.09679602	0.05055183	-0.0250954	0.12706708	0.10328644	-0.032593
-0.0234301	-0.1333592	-0.1688336	-0.2575541	-0.1869303	-0.0771753	-0.1638318
0.24597126	0.43371537	0.49149962	0.38639043	0.40216593	0.20085788	0.07864376
0.0031629	0.22190066	0.17628901	0.2830968	0.15407985	0.05784201	0.0507861
-0.1176635	0.02284277	0.05815745	0.08055768	0.08707497	-0.0666585	-0.2279706
-0.15296	-0.15449	-0.12225	-0.13105	-0.092211	-0.15999	-0.087846
-0.7190048	-0.6469931	-0.1492687	-0.5609695	-0.4055163	-0.5407353	-0.7269894
0.060758	-0.18153	-0.23635	-0.12782	-0.20298	-0.022099	0.031364
0.04041947	0.05662318	-0.0890262	-0.0526813	-0.0319888	0.05104023	0.00154138
-0.1769285	-0.1880449	-0.1184807	-0.111315	-0.1692819	-0.0517723	-0.0780569
-0.0746056	-0.1334018	-0.1069071	-0.1021314	-0.2604999	-0.1321821	-0.2036178
-0.2368191	-0.6001535	-0.3458183	-0.4163298	-0.3169851	-0.5243309	-0.5471474
-0.16338	-0.26314	-0.054827	-0.082433	-0.12866	-0.23671	-0.12313
-0.6785986	-0.4089254	-0.3698687	-0.3713968	-0.1656702	-0.7978329	-0.8273101
-0.1479291	-0.0023113	-0.0775298	-0.0053437	0.09893072	-0.225644	-0.3310977
0.08652111	0.05295179	0.05815662	0.03065286	0.04838898	-0.0043986	-0.097089
-0.2897975	-0.1985441	-0.2417549	-0.3131757	-0.3012275	-0.4105713	-0.3622767
0.3200013	0.35342444	0.43547783	0.45269897	0.44151552	0.06075952	-0.0121768
0.00403769	-0.0478018	0.07881569	-0.0094168	0.03021642	-0.0409131	-0.0296207
0.06409503	0.12528865	0.010697	0.0001233	0.2694975	0.04773525	0.02571494
-0.5033964	-0.5829601	-0.4835366	-0.6104554	-0.5106947	-0.5532458	-0.5779189
-0.0719278	-0.1351549	0.00178908	-0.0826105	-0.0539515	-0.1597131	-0.1616475
-0.1689554	-0.1527927	-0.2144222	-0.1899819	-0.2360618	-0.079624	-0.128151
-0.1764025	-0.1642379	-0.0796297	-0.1638204	-0.0617829	-0.0973352	-0.1229076
-0.4187286	-0.5955129	-0.6003758	-0.5215049	-0.6626645	-0.01378	-0.2043287

0.05579236	-0.0341934	-0.1176251	-0.1664341	0.09781007	0.18611047	0.07070111
-0.1613139	0.09030437	0.01924153	0.02358227	-0.0210644	-0.0323292	-0.1051693
0.07785458	0.35322647	0.27683261	0.2866527	0.3366856	0.06146978	0.03734095
0.03602617	-0.0213147	0.08382353	0.08827998	0.0607691	0.03024576	0.15592019
0.30001191	0.25325359	0.49003169	0.39423813	0.48517779	0.2426282	0.10814161
-0.0787876	0.00502598	0.02568162	0.17935687	0.08521336	-0.0300162	-0.0052906
0.39309259	0.60710166	0.60797615	0.56506915	0.54855641	0.32599159	0.20977269
0.31020117	0.27107023	0.21922756	0.28896917	0.06912642	0.31601374	0.32976572
0.10588382	0.2057488	0.02647688	0.06578613	0.14083707	-0.0235968	-0.0250528
0.12687892	0.03551302	0.00801389	0.03635074	0.00376412	0.00722184	-0.0577075
0.06242897	-0.2886711	0.03851912	-0.1801212	-0.1582991	0.05980748	0.05177321
0.03803044	-0.1560057	-0.0774808	-0.1618869	-0.1010869	0.09710396	0.00610905
0.07777903	0.04757415	0.05101278	0.10034071	-0.0074555	0.00340625	0.01041922
0.0071949	0.03349042	0.02838231	0.01409603	0.03648784	-0.000357	-0.0458728
-0.1515193	0.08222936	-0.1097139	-0.0048026	0.10224064	-0.2539081	-0.2744699
-0.1447353	-0.1129757	-0.2082301	-0.0765996	-0.1458864	-0.1284169	0.0480878
0.11105586	0.03006324	0.25195903	0.13753625	0.02443025	0.05126199	-0.0772951
-0.0701425	-0.5500255	-0.5428809	-0.42592	-0.4198246	-0.1095292	-0.2097764
0.26122633	0.17111412	0.31329865	0.29550031	0.25658732	0.30078144	0.15522704
-0.2568534	-0.2092449	-0.3493201	-0.2469144	-0.3460733	-0.2145028	-0.0741719
-0.4172684	-0.1541911	-0.3112782	-0.1857813	-0.2690731	-0.5989324	-0.4265958
-0.0093346	0.08442653	0.05396068	0.01889858	0.12878701	0.03693237	0.05705082
0.10419432	-0.0182156	0.11878638	0.09031702	0.00107723	-0.0035949	-0.0470753
0.45206978	0.74420434	0.69067185	0.47352416	0.6820197	1.1073333	1.0860371
0.02705069	0.05430618	-0.0595658	0.06777411	-0.0488437	0.01003077	0.04423079
-0.1993457	-0.1703745	-0.2146113	-0.0708419	-0.072356	-0.3032069	-0.2772648
-0.2554989	-0.1722079	0.03444731	0.25824011	0.26151653	-0.3854902	-0.34567
-0.3240302	-0.6115838	-0.5744922	-0.4094809	-0.4932517	-0.4678136	-0.5854491
0.152787	0.26707839	0.22355026	0.28881144	-0.0614383	0.25693466	0.29309654
-0.4146931	-0.319251	-0.5567117	-0.4612811	-0.4406596	-0.2890152	-0.3476306
-0.0932255	-0.2434802	-0.0929838	-0.0426566	-0.1024653	-0.0704718	-0.1482655
-0.0285843	0.06394567	0.11811646	0.09986422	0.03993753	0.12117981	0.15619848
0.15154884	-0.023152	0.01773958	-0.0530199	-0.0434607	-0.0276603	-0.197059
0.06676421	0.08353571	0.07576467	0.17255665	0.13449462	0.10219748	0.06300069
-0.8100936	0.50735612	0.28581951	0.44341924	0.53096886	-1.005776	-0.7096636
-0.3256487	-0.2375862	-0.2924144	-0.1167075	-0.1759356	-0.1580138	-0.132255
-0.36782	-0.21722	-0.42766	-0.30774	-0.39274	-0.016398	-0.10561
-0.1755298	-0.4492638	-0.3348496	-0.3691071	-0.421995	-0.0307151	-0.0574547
-0.1850582	0.09661478	-0.0635526	0.02059688	-0.0886283	-0.2631853	-0.2340073
-0.18007	0.032433	0.071839	-0.082029	-0.046438	-0.22285	-0.099548
-0.0529025	-0.0007128	0.08272734	0.07667913	-0.0355391	0.01372369	-0.0666615
0.11398257	0.04429118	0.05170053	0.09128247	0.04667462	0.10065883	-0.0058522

-0.2769756	-0.1171476	0.04584342	-0.0478766	0.08479521	-0.2020871	-0.3335375
0.06320262	-0.0248729	0.01225908	0.01034716	0.07946821	-0.0924062	-0.0777939
-0.4753829	-0.8676345	-0.7405406	-0.6545977	-0.6671203	-0.5777321	-0.6623941
-0.1589303	-0.2751044	-0.2236885	-0.0918339	-0.2352551	-0.0173652	-0.0221083
-0.2759352	-0.1140948	-0.1909745	-0.1992204	0.1307483	-0.4454714	-0.3422251
-0.5012893	-0.6488516	-0.4903946	-0.5437939	-0.6301042	-0.4893358	-0.372471
0.04402429	0.42331936	0.33178027	0.37385046	0.5699412	0.08107693	-0.0296063
-0.6967467	-1.4498984	-1.3058813	-1.1527586	-0.9431656	-2.388661	-2.3739765
0.0212801	-0.1114016	-0.0717563	-0.0454265	0.09949377	-0.0099162	-0.1098687
-0.096456	-0.34524	-0.37681	-0.31929	-0.35636	-0.38184	-0.2153
-0.2844656	-0.0343633	-0.171791	-0.0866575	-0.0276324	0.05992463	0.03796945
-0.1042196	-0.219511	-0.1744131	-0.0762596	-0.3149359	-0.1668389	-0.1713701
-0.5803558	-0.7023821	-0.7338887	-0.6034095	-0.6934192	-0.2765118	-0.3845438
-0.3190795	-0.3608355	-0.1396559	-0.2312905	-0.2045645	0.01120204	0.09210443
0.09258519	-0.0851585	-0.0167215	-0.0515845	-0.2466108	0.35986812	0.09726877
-0.3087768	-0.0510831	-0.0222085	-0.1713752	-0.3363822	-0.0682397	-0.0758191
-0.1721686	-0.2896059	0.04611087	-0.2199168	0.11537719	0.0924798	0.19232579
0.25182536	0.04172072	0.17858746	0.12414047	0.43685433	-0.2954971	-0.0907745
0.06022899	-0.0679533	0.07177715	-0.0174538	-0.0441663	-0.0027886	0.02041739
-0.1473448	-0.1054467	-0.0020652	0.11726706	-0.013159	-0.1225603	-0.1215692
-0.0717714	-0.1177297	-0.1707591	-0.0950305	-0.1525243	-0.0604479	-0.0806336
0.08341207	-0.0154954	0.00754849	-0.0409681	-0.0387527	-0.0764615	-0.0494018
0.07507624	0.05806774	-0.1596954	-0.1193095	0.0270254	0.03799388	0.1113535
0.24846596	0.08549275	0.02042456	0.14662619	0.16978523	0.00306699	0.05611445
0.14871898	0.09391643	0.25637366	0.30891228	0.10851131	-0.0851995	-0.0827988
-0.0227177	0.0560493	0.14754237	0.12298069	0.08717947	-0.0018772	0.11618149
-0.584898	-0.2271159	-0.2711361	-0.3233069	-0.1947145	0.33732236	0.55525401
-0.3129302	0.11092081	0.29252873	0.01534927	0.26334143	0.9985875	0.66248096
0.17531475	0.0613372	0.05477646	0.03311512	0.07259336	0.11931717	0.01675386
0.09550442	0.10993783	0.04824782	0.00179867	0.04322271	0.07037952	0.01784817
-0.2353595	-0.0134976	0.015966	0.07529521	-0.065754	-0.1624658	-0.1387539
-0.0961275	-0.1338274	-0.1221737	-0.1482896	-0.0975323	-0.0430425	-0.0210389
-0.4600553	-0.1095547	-0.1176221	-0.274794	-0.3291082	-0.1297698	0.28868867
0.67072622	0.18323371	0.29982783	0.30469377	0.34380492	0.66048754	0.36706301
-0.1938269	-0.2718523	-0.4081625	-0.294825	-0.1657074	-0.2227869	-0.1128104
0.06053463	0.08069779	-0.2832552	-0.0202014	-0.0606623	0.06141523	0.10783825
0.11737433	0.0860512	0.0827645	0.17794088	-0.0646554	0.13066151	0.14239163
-0.2781027	-0.0956772	-0.1561685	-0.1277112	-0.1701053	-0.2140964	-0.0531659
0.08837879	0.08002532	0.14160507	0.13183236	0.15289175	0.12813664	0.08561833
0.11229215	0.04125718	0.17877105	0.09922472	0.08136914	0.17487474	0.116896
-0.1460827	0.00385065	0.17839613	0.13910924	0.05077551	0.13135909	0.18733157
-0.2101314	-0.1187338	-0.1995959	-0.1253438	-0.1702423	-0.0449851	-0.0529415

-0.0995494	0.11323003	0.24225876	0.06615261	0.01943118	-0.1344778	-0.1043842
-0.069346	-0.067092	-0.15225	-0.090137	-0.068803	-0.19147	-0.24726
-0.0766188	-0.1524434	-0.1471675	-0.1930366	-0.0761705	0.17454391	-0.0778862
-0.05838	-0.1326252	-0.0875389	-0.0265983	0.06171217	0.00955581	-0.1084889
-0.5646172	-0.5740044	-0.6905535	-0.4917375	-0.2895823	-0.3286042	-0.2588512
-0.0133358	0.21737666	0.17742261	-0.0506476	0.19035598	-0.1857543	-0.163252
-0.5226947	-0.4001908	-0.452998	-0.3676818	-0.1721509	-0.4319147	-0.2830765
-0.22225	-0.30903	-0.32414	-0.34967	-0.25307	-0.2216	-0.22286
-0.0941302	-0.021112	-0.171888	-0.0902541	-0.0446026	-0.0153309	0.05619133
-0.31035	0.12235	0.10251	0.17601	-0.0075819	0.057853	0.17508
0.1176743	0.06926598	0.09434419	0.04326428	-0.0359124	-0.0845343	-0.0620635
-0.1777643	0.1279212	-0.1260916	-0.0286826	-0.0471334	-0.2075595	-0.1621747
0.02758037	0.16534739	0.02233009	0.04524412	0.03188164	-0.0953423	-0.0542386
0.08430156	0.08305169	0.02803175	0.15470552	-0.0017941	0.00639655	0.03729993
-0.4782747	-0.6167637	-0.6103873	-0.6401012	-0.5001213	-0.2911893	-0.3137404
-0.3214813	-0.5532375	-0.2950216	-0.3530832	-0.2917157	-0.233564	-0.098611
0.08048531	0.02921622	0.21451053	0.04917443	0.0976862	-0.1426744	0.01963864
0.41981291	-0.0551385	0.08613023	0.18573856	0.33104926	-0.2867963	-0.2888979
0.38538515	0.11066097	-0.019209	0.00269183	-0.1509473	0.16962229	0.25562195
-0.2914055	-0.4162271	-0.376067	-0.4079946	-0.2591113	-0.3107641	-0.3312245
-0.17825	-0.5550069	-0.5304542	-0.3467566	-0.3970706	-0.3390549	-0.46681
0.03415585	-0.227264	-0.1835825	-0.4247695	-0.1917903	-0.2758797	-0.4407091
-0.15654	0.037237	0.050417	0.040306	0.00481	-0.015245	0.0051443
0.05195034	-0.2624986	0.02064722	-0.2050894	0.04414761	-0.3393933	-0.349256
-0.1528276	-0.1484483	-0.1917594	-0.1257193	-0.1412648	0.00282294	-0.0092381
-0.1179226	0.18632539	0.21576739	0.24101274	0.13008864	-0.0185457	0.10230441
-0.1819092	-0.3454807	-0.2909561	-0.1663322	-0.4145813	-0.1225742	-0.356916
0.043524	0.081583	0.0054213	-0.007755	0.046796	-0.057575	-0.056169
-0.2231482	0.20273777	-0.0698259	0.00263914	0.28779449	-0.4287915	-0.3848334
-0.0742809	0.01895639	0.099125	0.2049055	0.11812101	0.05779913	0.13643648
-0.3829877	-0.2827942	-0.3157338	-0.3685676	-0.2020888	-0.2136514	-0.2434009
0.00230626	0.14737788	0.0584733	0.1547593	0.21061761	0.13007811	0.05700931
-0.29362	-0.27833	-0.076513	-0.31761	-0.10049	0.026648	-0.036908
-0.3320557	-0.2500223	-0.2409042	-0.2796493	-0.2757834	0.58053011	0.42233711
-1.1064982	-0.796527	-0.75085	-0.6970465	-0.6986892	-1.401018	-1.4452388
-0.2394943	-0.5409148	-0.5733111	-0.4540284	-0.4822471	-0.2769473	-0.387272
-0.3836587	-0.4196723	-0.3128092	-0.4194255	-0.3755529	-0.4420095	-0.3196957
-0.2589115	0.0165253	0.13251562	0.04410071	0.079731	-0.2364422	-0.0176011
-0.3368369	-0.531163	-0.3807363	-0.0613971	-0.30357	-0.4269993	-0.1050526
-0.2276834	0.26360774	0.26764	-0.0310774	0.33039799	0.40971028	1.0457124
-0.2013713	-0.0420361	-0.1618819	-0.107915	-0.2233113	-0.2047994	-0.2384011
0.00756204	0.02848811	-0.1241673	-0.2019231	-0.2036677	0.08354978	0.0694302

-0.0236405	0.02757092	0.0836434	0.05328663	-0.0574498	-0.0217809	-0.0075012
-0.3543193	-0.388596	-0.463392	-0.269141	-0.3758559	-0.3157625	-0.3121134
0.05723675	-0.0616699	-0.0229178	-0.0136371	0.03029457	0.00332451	-0.1132068
0.00896428	0.01126795	-0.1895962	-0.1230179	0.09786784	-0.1778438	-0.1305172
-0.1159502	-0.178981	-0.2142843	-0.1777436	-0.150211	-0.1513877	-0.1523218
0.1256397	0.05684874	0.03241854	0.27084768	0.03918584	0.05023333	0.02883931
0.03382998	0.09978606	0.09097199	0.0867977	0.01824995	0.09648981	0.01043445
-0.0220176	0.17589497	0.06260624	0.12332468	0.13496466	-0.0414161	-0.0194426
0.09190951	0.10649271	-0.0175714	-0.0629064	0.00286054	0.0637759	0.02432006
-0.43745	-0.44858	-0.32802	-0.43648	-0.43562	-0.36644	-0.32614
0.14331715	0.05993948	-0.0144903	-0.0211869	-0.0217238	-0.0330994	-0.0451159
-0.0420604	0.04692606	0.09261237	0.04142141	0.07293272	0.07992073	0.11307705
-0.2113994	0.02641876	0.01507296	-0.038123	0.067707	0.00114579	-0.0633929
-0.26528	-0.12519	-0.083129	-0.11163	-0.14966	-0.18919	-0.06174
0.16729767	0.05330748	0.00574564	0.1091714	0.00222761	0.08718405	0.09945444
0.07540398	0.05421949	0.04895124	-0.0055976	0.03107363	0.05234231	0.08135696
-0.0980354	-0.1319297	-0.0263171	-0.0320735	-0.1757435	-0.0973165	-0.185947
0.03619511	0.04315562	0.22829046	0.11118924	0.26320105	-0.3033374	-0.084754
0.20082241	-0.0181862	0.29486219	0.17045927	0.28955228	0.01874371	-0.0452977
0.12280223	-0.295453	-0.1030566	-0.0016922	-0.109006	-0.0923937	0.12606074

test_GSE125	test_GSE125	test_GSE358	test_GSE399	test_GSE40313_hns_ydgT
ref_GSE1255	ref_GSE1255	ref_GSE3582	ref_GSE3992	ref_GSE40313
GSE12554	GSE12554	GSE35822	GSE39926	GSE40313
GEO	GEO	GEO	GEO	GEO
affymetrix	affymetrix	RNAseq [stra	RNAseq	RNAseq
CRANBERRY:	CRANBERRY:10,CRANBERRY_TIME:230			hns_b1237:-1,cnu_b1625:-1
2376	2377	2380	2391	2403
0.28183114	0.23609302	0.28102825	0.03733475	0.71917559
-0.6024616	-0.5161167	0.33361349	0.03218729	2.4979136
-0.337221	-0.5693629	-0.3985528	0.25291944	0.54991712
0.09027667	0.08876628	0.7673103	-0.0819037	-0.0813824
-0.0311858	-0.1073732	-0.1080107	0.00136391	3.7133491
0.24774803	0.10303069	0.45293053	0.14098206	-0.0515181
-0.1222419	-0.2756521	-0.2715298	0.09109826	-0.7781323
-0.1875327	-0.3108338	-0.5823373	0.10006298	1.1974018
0.08713958	-0.0412044	0.46778122	0.06598696	0.59479444
0.17576259	0.1209557	0.26025768	0.05577889	0.00191965
-0.2185593	-0.3247744	-0.2799115	-0.1197616	0.11496593
-0.4437764	-0.7206308	-1.2060713	0.25897788	-0.4149422
0.17230446	0.12781689	-0.6627862	0.39086544	-0.0650754
0.03512379	0.04191675	0.02633311	0.03102123	-0.3456047
0.03344382	-0.0855148	-0.5713063	0.3115881	1.5723934
0.24542232	0.03115036	0.65077746	-0.0885972	0.27355785
-0.71806	-0.58839	-0.1140074	-0.1861872	-0.8528172
-0.0119806	-0.1311066	-0.390483	0.3947678	1.8537136
-0.1866987	-0.22693	-0.2837434	0.3739234	0.09876137
0.16447243	0.18148539	0.99468399	-0.0242004	-0.1423203
-0.0508383	-0.063527	0.02633311	-0.2124294	0.14346711
0.021442	0.042378	-0.005002	-0.0903877	0.13363262
0.21583254	0.16064459	0.88598047	-0.2908727	-0.2277744
-0.23451	-0.26234	0.84115692	-0.1647616	0.36777549
0.0011945	-0.1132171	0.84599225	-0.2825801	-0.3927106
0.08959735	0.25283293	-0.3551839	-0.2494266	0.08064431
-0.60049	-0.56343	-0.4045138	0.44571594	0.71826089
-0.0028701	-0.3415984	-0.4615852	0.14063985	-0.3838132
-0.028394	-0.04431	-1.1190846	-0.1061053	-0.2895806
-0.128391	-0.1530009	-1.4480227	0.17897272	2.0622357
-0.0188057	0.00463279	-0.8006533	0.20268733	-0.1304939
-0.2507086	-0.2864115	-0.3422642	-0.0318049	0.53563877

0.09989021	0.01857081	0.61553197	-0.0654187	0.57916289
0.06800286	0.06492752	0.28949291	-0.114524	-0.2184785
0.1114916	0.06691704	-0.637302	-0.1097794	0.80759161
0.00894366	0.07160921	-0.9559676	0.50395694	1.4997201
0.00705946	0.05831137	-1.2553657	0.06188778	1.3849099
0.01393628	-0.0322782	-1.8842479	0.18998233	2.7566567
-0.0233836	0.02348507	-1.7217725	0.21320619	1.7495213
0.08036085	0.06883748	-0.351191	0.03733475	0.39646318
-0.0137657	0.02846774	-0.4308623	-0.0189387	-0.1506739
0.08601833	0.14500115	-0.6156675	0.35291029	1.1635769
0.28658209	0.57426022	-0.3524573	-0.1046584	2.6055585
-0.7611779	-0.7196867	-0.3844598	-0.4523667	-0.658681
0.0867516	-0.0011478	-0.527155	0.26762454	0.43680757
0.0024178	-0.0475456	0.3912448	-0.0340817	-0.2152581
-0.3602839	-0.5081685	-0.5046579	0.00903788	-0.4207155
-0.126	-0.1208709	0.02633311	0.06072977	-0.0496719
0.10324859	0.24546558	0.56993749	-0.0941067	0.0232493
0.17540122	0.06036577	-0.4468353	-0.1003338	0.23435521
-0.6691994	-0.4466337	-0.0203501	0.05632607	-0.1525278
0.14369905	0.11098518	-0.2510635	0.03702912	2.0368447
-0.2297956	-0.0268768	-0.9093519	-0.2064048	-0.4160608
-0.1660817	0.03682127	-0.1642758	-0.136609	-0.35645
-0.0635204	-0.0928449	0.18400549	0.12693564	0.07010671
0.06405374	0.11373188	-0.4882202	-0.0652448	-0.0240784
-0.1750981	-0.1119071	-0.3978661	0.13077406	-0.3341822
-0.7637216	-0.9497436	-2.1294837	-0.1864551	-0.2811206
-1.4815626	-0.8255249	-1.1665563	-0.0098462	-0.3199818
-0.0287454	-0.0019841	-0.9379571	0.48019929	0.42266055
0.01336216	0.12319109	-0.7797132	-0.2675114	0.35936306
0.06360905	0.12908838	-0.6371423	-0.00627	0.49740893
-0.3535654	-0.2881955	-0.1096097	-0.0008661	-0.0266731
0.15530459	0.15759344	0.12513916	-0.0034126	-0.5840936
-0.4629548	-0.2215199	0.63068673	0.07466954	-0.7693133
0.06471129	0.19172014	1.8746223	-0.0661599	-0.9940865
-0.101786	-0.0074562	-0.414071	0.10721851	2.5345056
-0.2760689	-0.2125713	-0.5186145	-0.1352609	0.07494502
0.03615914	0.0455836	-1.0210594	-0.0051758	0.63757062
0.33793545	0.09717523	2.5907004	0.24477917	0.11496593
-0.2904162	-0.3598226	-0.360086	0.17987498	0.38054579
-0.2222024	-0.0812372	-0.6605293	-0.0996123	-0.715151
0.01647116	0.05573185	0.23666284	-0.1410184	-0.2277744
-0.2528902	-0.2466909	-0.5672381	0.23933027	0.69531573

-0.1029049	-0.2362925	-1.0223262	-0.1491527	-6.2918187
-0.2153086	-0.0572712	-0.0883331	0.00653198	0.78247946
-0.0365213	0.02127783	-1.3301518	0.01106752	0.56232712
-0.0162638	0.02434084	-0.5023869	-0.0761344	-0.7331635
-0.044696	-0.0103275	-0.0590212	-0.1247551	0.06771983
0.06827032	0.1668441	-0.2823823	-0.2750218	0.77333205
0.17689071	-0.0028694	-0.1956269	0.54770526	0.31683599
-0.1237423	-0.1378493	0.18686472	-0.0721138	0.14334524
-0.0070431	0.02033498	-0.7743007	0.27746791	0.62286191
0.0028329	0.23533962	-0.4144418	0.30908293	-0.7843175
0.17759655	0.16169778	-0.2878451	0.15364714	-0.5946307
0.01640324	0.0738176	-0.555919	-0.00627	-0.1445672
0.18811578	0.0864916	0.37596267	0.23337942	-0.715208
0.11169095	0.02254473	-0.776597	-0.1022594	-0.1018653
0.031448	-0.043752	-0.8965899	-0.0073642	-1.1743964
-0.1776411	-0.2388824	-0.5886086	-0.0290452	-0.6018832
-0.1016696	-0.1379342	0.58372086	-0.0291295	0.07545795
0.03129174	-0.0722207	0.15262161	0.29590289	-0.6941025
-0.073815	0.018031	-0.1632702	0.27052271	4.157452
-0.0151845	-0.0265138	-1.3038901	-0.0121647	0.0232493
-0.2470185	-0.2071706	-0.3407238	0.27367667	2.8553126
-0.27908	-0.34162	-0.0452858	0.18677469	0.7688556
-0.5198838	-0.4414244	-0.7171331	-0.0413622	-0.3254854
0.05424019	0.0170142	0.14270381	0.27642683	0.55356649
0.02157002	0.17212498	-0.2040443	-0.0408039	0.17164075
0.24772268	0.22645313	-0.5915757	0.01823923	-0.1884099
0.10635442	0.1179324	-0.1968282	0.00182117	-0.0451086
-0.2068609	-0.210324	-0.6192689	-0.0044779	-0.907957
-0.084119	-0.11546	0.02633311	0.28755416	1.6608628
0.12073	0.057982	-0.8576656	0.14024732	-0.3317039
0.040138	0.02671	-0.6220143	0.08839555	0.00770989
-0.084089	0.072291	-0.5713063	0.08754744	0.07948884
-0.027804	-0.044962	-1.4869195	0.16622075	0.08378703
-0.045562	-0.039479	0.56071779	-0.0741325	-0.8949363
-0.014749	-0.0081913	-1.762509	0.18856859	-0.2894192
-0.1517601	-0.1126212	-0.1238307	-0.1448031	0.20196412
0.23756364	0.2715123	1.223318	-0.1134518	0.29848832
-0.1011703	-0.1518679	-0.4597333	0.23377654	-0.0559203
-0.3003692	-0.4093965	-0.8376303	-0.0225221	-0.4317581
0.11411522	0.06382665	0.43535719	0.15364714	-0.0373897
-0.0668935	-0.0438666	-0.953326	0.59593802	0.6903227
-0.3815021	-0.6768631	-0.8160478	0.06550666	0.19885158

0.44893359	0.50284493	-0.6087888	-0.086845	0.03020043
-0.0787766	-0.2677303	0.01159499	0.01303536	-0.8767194
0.24431524	0.08296118	-0.2511836	-0.1009662	0.07332798
-0.1024742	-0.2029168	-0.0471704	0.15588951	0.51169908
-0.131313	-0.5620104	-0.4789322	0.30167566	1.1872404
0.57410673	0.83120137	-0.3936504	-0.3623372	0.23255274
-0.0048845	0.05451569	0.02633311	0.36227592	1.4535356
-0.1300738	-0.1259869	0.62680986	0.20996005	0.90197324
-0.2097315	-0.0747486	-1.6718485	0.10518517	0.06771983
0.08486564	0.13453776	0.16659198	-0.1654523	0.18766982
-0.6184597	-0.4582061	-0.6502244	-0.6881441	1.0745428
-0.3222588	-0.5375179	0.53496431	0.13157701	0.44383134
0.13401997	0.12433408	-0.453214	0.2001261	0.20837874
-0.0961574	-0.3187972	-0.5681752	0.07893314	-0.8416582
-0.4487546	-0.8080644	-1.229748	0.20996574	-0.5675782
0.28372279	0.11108893	-1.9038377	-0.2585703	1.314086
-0.2972157	-0.2021037	-0.9179484	-0.0225221	-0.1136876
0.06978124	0.02925339	-0.5713063	0.02400855	3.0725642
-0.1973722	-0.3431339	-1.5273941	-0.0563695	2.827504
0.39873401	0.27169726	-0.8042174	-0.1432407	-0.128316
0.25555651	0.01893669	0.00774677	0.17354878	0.0232493
0.20808563	0.08288009	0.02633311	0.26675823	-0.1156038
-0.023055	-0.082023	-1.2076784	-0.2095676	0.15808382
-0.0114216	0.0986137	-1.6459561	0.21251017	-0.1318594
-0.28235	-0.25315	0.12044662	-0.0434418	0.08486776
0.00522193	-0.0521554	-0.9179484	-0.2534497	1.7208355
-0.1649298	-0.0053689	-1.0255066	0.09995051	-0.3794071
-0.01422	-0.1314909	-1.4644787	0.08561767	0.22279643
-0.3620014	-0.4526648	-1.3044312	0.12614547	0.66810542
-0.05661	-0.11192	-0.360086	-0.2167446	0.24900409
-0.1551315	-0.4468276	0.87984124	0.04793355	-1.0206767
-0.1233092	-0.2882219	-0.4229225	0.0414864	-0.0939014
-0.0197716	0.0259462	0.42683376	0.1452712	0.08163894
-0.3212385	-0.4049925	0.02633311	0.13215856	1.4241537
0.51196857	0.45035133	-0.0731923	0.22965657	-0.449052
0.12299417	0.09151381	0.37986045	-0.1463482	0.39341635
0.32483102	0.18596407	-0.8411253	-0.0924859	-0.1441998
-0.5543746	-0.5539718	0.16102159	0.16637095	0.47981312
-0.1582284	-0.0855905	0.28254929	0.1901269	0.07545795
-0.3317015	-0.2545274	0.00553302	0.03443284	0.02838355
-0.0921251	-0.0810493	-0.0703717	0.12601814	0.0232493
-0.696714	-0.7875986	-0.6535245	-0.0596233	1.2949086

-0.1559206	-0.0546157	0.31923003	-0.0857114	0.14749462
0.01644162	-0.0646698	0.39417971	0.0789774	4.0147635
0.30586101	0.39695852	-0.43635	-0.0044824	-0.0536828
0.00970832	0.00202933	0.63068673	0.02619905	-0.2518277
0.46974662	0.35327816	0.70008999	0.04295942	-0.6031929
0.0020144	0.05825225	0.78487446	-0.1506812	-0.9217374
0.62444573	0.40120442	-0.4221535	-0.0115962	-0.1779103
0.31874845	0.20919669	-0.043796	-0.4719884	-0.5917476
0.1293542	0.06596083	0.06385925	0.11746784	-0.3991794
0.07477756	-0.0055816	0.08342867	0.20662619	-0.214252
-0.136947	-0.0455899	0.44757985	-0.1409741	-0.7748239
-0.0229627	0.02838184	0.60022114	0.17761666	-0.0884911
-0.0531467	0.06228671	-0.3100897	0.0645297	-0.4129302
-0.0559638	-0.0370977	-0.3793491	-0.0479837	0.41943494
-0.0606319	-0.0240151	-0.0517572	-0.0590562	0.55108874
-0.1298385	-0.127465	-0.0794042	-0.1794694	-1.3918056
0.15707914	0.11871683	-0.8094906	-0.1597362	2.1321211
-0.3167978	-0.8015845	-0.5486272	-0.1296866	-0.9355205
0.14268084	0.1112282	0.36598843	-0.0125074	-0.2608056
-0.2597355	-0.1601819	-0.623699	-0.0990318	0.94143491
-0.2786333	-0.3203059	-0.2955859	-0.0961434	0.15216558
0.0745746	0.12049336	1.4418532	-0.0087471	-1.0553635
0.11378238	0.01685156	-0.0051966	0.14078116	-1.0574996
0.27974997	-0.0120382	-0.5248563	-0.0652448	0.04253214
0.06221333	-0.03707	0.11343837	-0.0331215	0.37001435
-0.0333598	-0.2156617	-0.4096237	0.11394487	0.1456622
0.1859129	0.03133914	-0.7872474	-0.0978477	0.19770652
-0.3623571	-0.395908	0.008496	0.0583558	-0.4904042
0.07603429	0.33660088	0.06928816	0.30572038	-0.660091
-0.5529437	-0.5799588	-0.703494	-0.0173991	-0.1115125
-0.0899918	0.02724664	-0.2765342	-0.0459971	-0.3613476
0.04793882	0.09687399	-0.7122227	0.22101095	2.8053375
-0.0847738	-0.1249589	-0.242948	0.40264587	2.1695434
0.05742342	0.16664396	-0.3818707	0.19903221	1.1434911
0.53888282	0.72206559	0.10867734	0.14498899	0.5882802
-0.143164	-0.0816761	-0.1528088	-0.0936752	0.15105661
-0.28825	-0.33931	-0.6583915	0.2619298	0.43093844
-0.3116711	-0.2545821	-0.2597776	0.45843139	0.28516029
0.09728611	0.16232725	0.498347	-0.030984	-0.8338365
-0.11606	0.0089967	0.28935419	0.01882122	-0.0536828
-0.0426624	-0.0418557	-0.5426212	0.29580979	-0.9028342
0.04145742	0.12079313	-1.6554955	0.12456665	-0.1098321

-0.0860562	-0.0700936	0.33281349	0.24967961	-0.057022
0.01051644	-0.0485875	-1.3335332	0.07442755	2.1506206
-0.5937457	-0.6390296	-0.0531096	-0.1521232	-0.1604184
-0.3374578	-0.2918379	0.35164489	-0.0725958	-0.2782021
-0.1522712	-0.2620151	-0.5371094	0.15049763	0.43722831
-0.6885744	-0.4422436	-1.7458113	0.1658652	-0.0069561
0.16919163	0.07801779	0.29597292	0.37309537	-0.8623539
-0.9922844	-1.2373389	-0.2986296	0.18938327	2.2410767
-0.0626049	0.10412318	-0.4204043	0.23514713	1.3450451
-0.31764	-0.31703	0.11574198	0.04427666	-0.1458368
-0.1629687	-0.3125151	-0.2986296	-0.170045	-0.6141249
-0.0401553	-0.0974895	-0.5897341	0.12686781	0.17851749
-0.8456393	-0.8526796	-0.6730474	0.21818905	-0.8077642
-0.1698018	-0.0478005	-0.4086015	-0.1795728	0.04676775
-0.1394	-0.3049562	-0.4253306	0.11645316	-0.9111338
-0.0464644	0.04195367	0.62680986	0.01046326	1.0629592
0.14555583	0.32444154	-1.0759046	0.28155696	0.07002332
-0.1635738	-0.009372	1.0800985	0.00687894	-0.5339912
-0.0252135	-0.0182944	-0.6192742	0.04422314	-0.3565953
0.09975963	0.07679593	0.2009758	0.01997193	0.01005222
-0.1204134	-0.1554568	0.99891491	-0.0048549	-0.2948909
-0.0133699	-0.0353587	1.6345444	-0.149993	-0.0654131
-0.1096082	-0.252866	0.51518677	-0.2072834	0.19978017
-0.0884327	0.13202839	-0.9918453	-0.0111251	-0.4820768
0.30734613	0.30601439	0.19325763	-0.0994157	-0.2928256
0.13509117	0.10799301	-0.4886966	-0.1794579	0.0232493
-0.4451099	-0.4211585	-0.0162609	0.29509546	2.1919495
0.62061758	0.60260103	-0.7259741	0.43609178	1.4416301
-0.022286	0.0002068	-1.2741802	0.34499059	2.9901636
0.0203186	0.01024445	-0.0919308	0.19772966	1.9168418
0.20055196	-0.1050739	0.26459285	0.23737825	0.40967941
-0.2074073	-0.0566123	0.66184698	0.04748792	-0.5254542
0.49418905	0.51293859	-0.8349175	0.28288714	1.3550602
0.03003549	-0.1144911	-0.5929131	0.32266044	-0.3130717
-0.311432	-0.2608311	0.02633311	0.12965696	0.64753569
-0.0924269	0.0473246	0.21049132	0.10467421	-0.3586518
0.1711834	0.12742449	0.20357253	-0.0129683	2.3794743
-0.1302526	-0.2513613	0.11159782	-0.129243	-0.2761292
0.08367528	0.04496591	0.29753856	0.20240848	0.0232493
-0.0239069	-0.0118643	0.17550013	-0.3243657	-0.8629682
0.12258943	0.14340713	-0.0278277	-0.2317553	-0.5320601
0.09445511	-0.2068336	0.02633311	0.16458561	1.9605269

0.06030823	0.10096332	0.43207554	-0.0235592	-0.8949363
0.01579	-0.20115	0.19504648	-0.1222324	0.40309983
-0.3898367	-0.5014056	0.38612457	-0.1319978	-0.6297382
0.03293574	-0.1131665	-0.1614661	0.41615201	-0.5365489
-0.4316423	-0.5786613	-1.0649238	-0.1032057	-0.1604184
0.19257053	0.03431165	0.46462173	-0.1534703	-0.4040324
-0.3337217	-0.3727903	-0.1643343	0.15133188	0.02930586
-0.39295	-0.3251	-1.0788508	0.22049126	0.32943045
-0.1366568	-0.1886271	1.0586742	-0.0559543	-0.0464569
0.022969	0.067093	0.66184698	-0.3345769	-0.2277744
-0.0015492	-0.0448369	0.92313122	-0.053295	0.39098928
-0.1089215	-0.0641936	0.53026356	0.27954144	-0.3627339
-0.0354272	0.02374513	-0.4412137	-0.0071381	-0.3843576
0.0102025	-0.008784	0.69092797	-0.0668968	0.65111663
-0.5532695	-0.6094046	-0.4309836	-0.1782906	-0.1604184
-0.1795029	-0.139894	0.22985818	0.39673511	-0.4343153
0.36529816	0.64223493	-0.1865089	-0.3273426	-0.3378059
0.34229587	0.02120891	0.28853415	-0.0640406	-0.1750959
-0.0771272	-0.2270186	-0.171639	-0.0242497	0.22500692
-0.4339751	-0.4341311	-0.5853423	0.03090872	-0.1924899
-0.6362928	-0.4749244	-0.539519	-0.0708134	-0.1183307
-0.2925157	-0.2577366	-0.1740679	-0.1320662	-0.5437488
0.040242	-0.022638	0.30765157	0.0609995	-0.2193151
-1.0965592	-1.0123525	-1.2119665	-0.0790287	0.3130755
-0.004871	-0.0345436	1.2533017	-0.0892563	0.45331899
0.15582536	0.13862831	-0.6606792	-0.0838583	-0.2453931
-0.3283467	-0.4807247	-0.5018129	0.0522278	-0.4744972
0.048354	-0.039833	-0.5953855	0.16172143	0.09890439
0.11828429	-0.0782305	-0.3024768	-0.1006535	-0.3371083
0.23931024	0.34788097	0.8695676	-0.3352525	0.7178299
-0.2681423	-0.193501	-0.4999193	0.34585211	0.77966162
0.15561564	0.28640873	1.5113645	-0.0769682	0.04253214
-0.30038	-0.35667	-0.3141244	0.02269363	0.36240656
-0.5434441	-0.5656795	-0.0496155	0.29768707	3.9461125
-0.5572512	-0.6135225	-0.5471556	0.15837236	0.16480236
-0.8108987	-0.6866154	0.12513916	0.216981	0.62331928
-0.3444362	-0.4396882	-0.4099373	0.17794058	4.2024751
0.22265379	0.27768615	-0.5685273	0.18653593	0.73003776
-0.339981	0.03052637	-1.1022145	-0.1534858	-0.3050137
0.40463755	0.56220305	1.0658476	0.11932042	1.5539107
-0.0940113	-0.204225	-0.5036188	0.18688693	-0.0683696
-0.0586175	0.08457027	0.98040431	-0.1222863	-0.0399528

0.04224366	0.0117457	-0.5237528	-0.1545482	0.0232493
-0.4713298	-0.4197314	0.47852612	-0.1298268	-0.2012402
-0.0330906	-0.0112392	-0.2633054	-0.1788552	0.0232493
-0.1335608	0.05122194	0.67183393	-0.0004514	-0.0681842
-0.2028257	-0.1432175	-0.5034163	0.07794699	0.25961593
0.11018891	0.02281565	0.10318325	0.20976147	-0.2813838
0.00547914	0.03353745	-0.2692093	0.10028395	2.4502525
0.08238073	-0.0032111	-0.4371196	0.33412675	1.3740305
0.15352361	0.19091717	0.46582989	0.11394487	0.06611561
-0.36971	-0.47778	-0.5064438	0.05077318	-1.5623891
-0.0726808	0.0005869	0.82506616	0.01997193	-0.3843576
0.04282208	0.14276227	0.19079451	0.23210151	0.75176062
0.07049268	0.07091704	-0.2282623	0.42657143	2.6543426
-0.293	-0.24871	0.15715563	0.00836894	1.5002665
0.12578738	0.0217696	0.48114174	0.01779452	-0.2368775
-0.0396747	0.08968648	0.02779611	0.09086574	0.06647005
-0.1469303	0.03175663	0.49891155	-0.2450857	-0.9960746
0.19312936	0.06576953	-0.3320681	-0.0136669	-0.0095613
0.29104126	0.14011832	-0.0410359	0.12751009	-0.2287037
0.12370723	0.12954974	-0.1072598	-0.3401807	0.06856682
