# Supplementary material for: Functional Prediction of Hypothetical Transcription Factors of Escherichia coli K-12 Based on Expression Data
Source: Comput Struct Biotechnol J. 2018 Mar 27;16:157–66. doi: 10.1016/j.csbj.2018.03.003 (PMC6055005; doi:10.1016/j.csbj.2018.03.003)
Supplement: Supplementary material Table S2 — Comparison of clusters identified in this work against previous analysis. [file mmc3.docx]

Table SII.

| Enriched Cluster | Module | P-value | palsson-dataset |
| --- | --- | --- | --- |
| 2 | 6 | 4.58E-07 | EcoMAC |
| 9 | 1 | 5.50E-90 | EcoMAC |
| 10 | 3 | 6.70E-271 | EcoMAC |
| 8 | 5 | 2.32E-142 | EcoMAC |
| 9 | 7 | 1.06E-14 | EcoMAC |
| 8 | 3 | 1.81E-114 | COLOMBOS |
| 5 | 6 | 1.84E-37 | COLOMBOS |
| 2 | 6 | 1.52E-26 | COLOMBOS |

**Therefore, to compare our clustering method, with previous works, an enrichment test using Fisher exact test was performed. We present the enriched clusters with a core biological function and a robust regulatory organization. The Palsson’s modules were used as benchmark because they used a similar expression compendium (EcoMAC) and compared with COLOMBOS, the same expression compendium we used, yielding similar results (normalized VI = 0.29). Overall, this results confirm a functional relationship between the expression patterns in the TRN, that can be verified by different approaches.**
